# Electrochemically driven regioselective C−H phosphorylation of group 8 metallocenes

**DOI:** 10.1038/s41467-022-31178-7

**Published:** 2022-06-17

**Authors:** Hao Zheng, Chang-Hui Liu, Shi-Yu Guo, Gu-Cheng He, Xiang-Ting Min, Bo-Chao Zhou, Ding-Wei Ji, Yan-Cheng Hu, Qing-An Chen

**Affiliations:** 1 Dalian Institute of Chemical Physics, Chinese Academy of Sciences, 457 Zhongshan Road, Dalian, 116023 China; 2 University of Chinese Academy of Sciences, Beijing, 100049 China

**Keywords:** Synthetic chemistry methodology, Electrocatalysis

## Abstract

Metallocenes are privileged backbones for synthesis and catalysis. However, the direct dehydrogenative C−H functionalization of unsymmetric metallocenes suffers from reactivity and selectivity issues. Herein, we report an electrochemically driven regioselective C−H phosphorylation of group 8 metallocenes. Mechanistic investigations indicate this dehydrogenative cross coupling occurs through an electrophilic radical substitution of the metallocene with a phosphoryl radical, facilitated by the metallocene itself. This work not only offers an efficient and divergent synthesis of phosphorylated metallocenes, but also provides a guide to interpret the reactivity and regioselectivity for the C−H functionalization of unsymmetric metallocenes.

## Abstract (web summary)

Metallocene-based phosphines are compounds with potential use in catalysis. Here, the authors report the electrochemical regioselective functionalization of group 8 metallocenes with phosphine oxides; over 60 examples of phosphorylated (benzo)ferrocenes and ruthenocenes can be accessed via this method without the need for a preinstalled directing group.

## Introduction

**Fig. 1:**
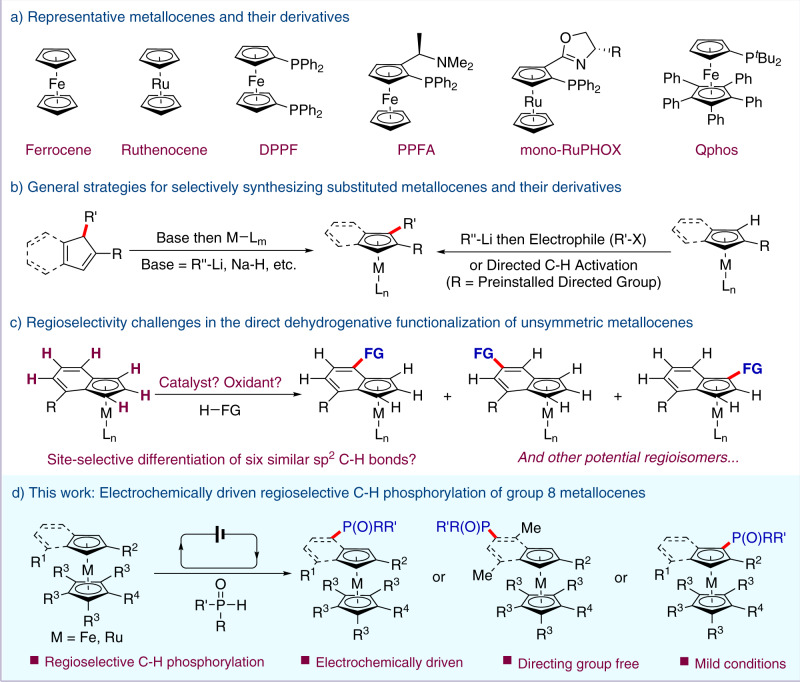
Synthesis of metallocene phosphine ligands. **a** Representative metallocenes and their derivatives. **b** General strategies for synthesizing substituted metallocenes and their derivatives. **c** Regioselectivity challenges in the direct dehydrogenative functionalization of unsymmetric metallocenes. **d** This work: Electrochemically driven regioselective C−H phosphorylation of group 8 metallocenes.

Since its discovery in the early 1950s^1–5^, ferrocene and its metallocene derivatives have received widespread attention owing to their broad applications in physics^6^, polymer science^7^, and medicine^8, 9^. With regard to organic synthesis and catalysis, metallocene-based phosphines have proven to be privileged ligands or catalysts (Fig. 1a)^10–20^. Although there are well-established methods for the synthesis of simple and unsubstituted metallocenes, the construction of substituted metallocene derivatives remains a challenge. Two general approaches have been used for the synthesis of substituted metallocenes. The first strategy is the coordination of substituted cyclopentadienyl precursors with the corresponding metal complex (Fig. 1b)^21–23^. While reliable, this approach usually requires the multi-step synthesis of substituted cyclopentadienyl precursors and stoichiometric amounts of strong bases. The second strategy involves the C−H functionalization of the metallocene. The latter exhibits a better step economy but relies on strong bases or preinstalled directing groups^24–38^. Therefore, it is of great importance to address the challenges in developing an efficient and concise protocol for the construction of substituted metallocenes.

Compared with the biscyclopentadienyl complex, the direct C−H functionalization of indenyl metallocenes poses an additional challenge in regiocontrol. Bearing six similar *sp*^2^ C−H bonds on indenyl moiety, the 4-substituted indenyl metallocene is a representative unsymmetric substrate for C−H functionalization (Fig. 1c). Theoretically, six possible regioisomers would be expected to form. We propose to develop a system to control the regioselectivity through site-selective differentiation of the six similar *sp*^2^ C−H bonds. Based on the principle of mass conservation, this direct C−H functionalization will generate one molar equivalent of hydrogen gas or an associated byproduct. Usually, an external oxidant is required as a hydrogen scavenger. Meanwhile, electrochemical synthesis^39–51^ is a powerful method to facilitate oxidative cross-coupling reactions^52–58^ under external-oxidant-free conditions through anodic oxidation. Specially, electrochemical phosphorylation^59–70^ has emerged as a complementary approach to conventional methods for forming C−P bonds.

Here, we report an efficient regioselective C−H phosphorylation of group 8 metallocenes (Fig. 1d). This oxidative cross-coupling protocol features broad substrate scope and mild conditions while avoiding the use of directing groups and external oxidants.

## Results

### Reaction optimization

Initially, under electrolysis conditions, benzoferrocene **1a** and diphenyl phosphine oxide **2a** were selected as model substrates to optimize the reaction. The reaction was conducted under constant current in an undivided cell equipped with a reticulated vitreous carbon (RVC) anode and a platinum plate cathode (Table 1). Using ^*n*^Bu_4_NOAc as the electrolyte, monophosphorylated product **3a** was obtained in high regioselectivity and 73% yield in MeOH (entry 1). It is notable that small amounts of over phosphorylated products (**4a**, **5a**, and **6a**) were obtained. No desired reaction occurred in the absence of current (entry 2). Other electrode materials, including Pt and graphite anode, were all found to be less effective in terms of reactivity (entries 3 and 4). The use of an alternative electrolyte, such as ^*n*^Bu_4_NBF_4_, or LiClO_4_•3H_2_O lowered reaction efficiency (entries 5 and 6). Increasing the current to 3.0 or 5.0 mA had a negative effect on selectivities and favored the formation of bisphosphorylated products **4a**–**6a** (entries 7 and 8). On the other hand, conducting the electrolysis without Et_3_N resulted in a lower yield (entry 9). Other basic additives such as NaOAc and K_3_PO_4_ failed to give better results (entries 10 and 11). Solvents such as TFE (Trifluoroethanol), DCE (1,2-Dichloroethane), and MeCN were screened as well, but lower yields were obtained (entries 12–14). Notably, the electrochemical reaction could also be performed at RT (room temperature), although with a slight decrease in yield (entry 15). And the use of stoichiometric oxidants (e.g., AgF, MnO_2_, and DDQ (1,2-Dichloro-4,5-Dicyanobenzoquinone), etc.) for this phosphorylation gave low yields (See Supplementary Table 1 in Supporting Information).

**Table 1:** Optimization of reaction conditions^a^.


Entry	Deviation from standard conditions	Yield (%)			
		3a	4a	5a	6a
1	None	79(73^b^)	1	3	8
2	without electricity	0	0	0	0
3	C rod as anode	59	2	3	6
4	Pt plate as anode	62	3	2	3
5	^*n*^Bu_4_NBF_4_ as electrolyte	47	0	0	10
6	LiClO_4_•3H_2_O as electrolyte	45	2	4	15
7	3.0 mA	32	5	6	33
8	5.0 mA	12	9	3	28
9	without Et_3_N	39	0	2	3
10	NaOAc as base	61	1	2	4
11	K_3_PO_4_ as base	49	0	7	3
12	TFE as solvent	16	3	1	4
13	DCE as solvent	24	3	1	1
14	MeCN as solvent	38	0	4	0
15	RT instead of 50 ^o^C	52	1	2	4

*TFE* trifluoroethanol, *DCE* 1,2-dichloroethane.

^a^Conditions: Undivided cell, constant current (2.0 mA), **1a** (0.20 mmol), **2a** (0.40 mmol), ^*n*^Bu_4_NOAc (0.20 mmol), Et_3_N (0.40 mmol), MeOH (4.0 mL), 50 ^o^C, under N_2_, 6 h, 2.2 F/mol. Yields were determined by ^1^H NMR spectroscopy using 1,3,5-trimethoxybenzene as the internal standard.

^b^Isolated yield.

### Substrate scope

**Fig. 2:**
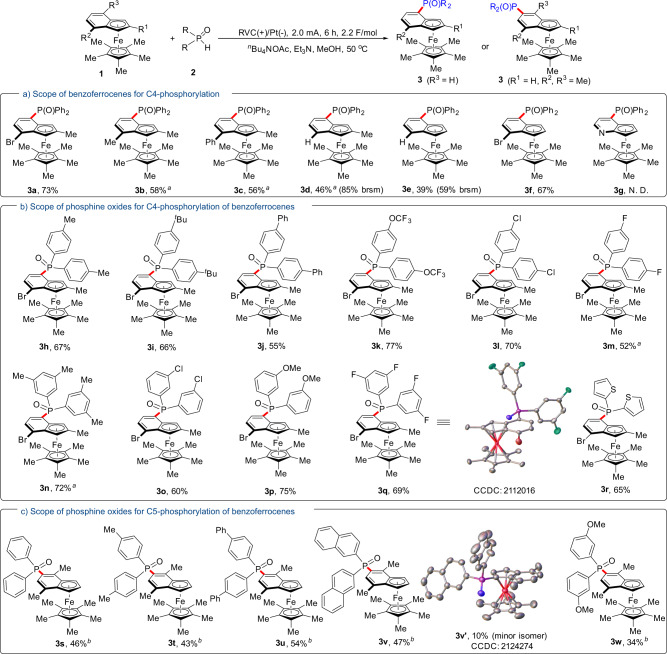
Substrate scope. **a** Scope of benzoferrocenes for C4-phosphorylation. **b** Scope of phosphine oxides for C4-phosphorylation of benzoferrocenes. **c** Scope of phosphine oxides for C5-phosphorylation of benzoferrocenes. ^a^Reaction with 2.5 mA, 6 h (2.8 F/mol). ^b^Reaction with 3.0 mA, 10 h (5.6 F/mol). Brsm refers to the yield based on the recovered starting material.

Having identified optimized conditions, we next evaluated the phosphorylation of different benzoferrocenes with diphenyl phosphine oxide **2a** (Fig. 2a). The presence of a Br, Me, or Ph group at the α-position of the aryl ring of 1-methylbenzoferrocences were all well tolerated to afford the phosphorylated products in decent yields (**3a**–**3c**). It should be noted that even when either Cp (cyclopentadienyl) or phenyl rings on the benzoferrocene substrates were naked, the phosphorylation still occurred at the *α*-position of phenyl ring (**3d**–**3f**). Some of the relatively low yields (**3d** and **3e**) were due to low conversion or decomposition of substrates rather than regioselective issues. These results suggest that the Br atom on model substrate **1a** does not act as an activating or directing group in this protocol. Due to its electron deficiency, the aza-benzoferrocene substrate was not applicable in this case (**3g**).

Subsequently, we turned our attention to the investigation of the scope of phosphine oxides with Br-substituted benzoferrocenes, which was good for further derivatization. As shown in Fig. 2b, phosphine oxides containing electron-donating or electron-withdrawing groups, such as -Me, -^*t*^Bu, -Ph, -OCF_3_, -Cl, and -F, at the para position of the aryl components, all resulted in good yields (**3h**–**3m**). Likewise, substrates with substituents at the meta-position of the aryl components also exhibited good reactivities and delivered the corresponding products in 60–75% yields (**3n**–**3q**). Delightfully, the reaction also demonstrated good tolerance for the phosphine oxide bearing a heterocyclic 2-thiophenyl group (**3r**) which is usually problematic under transition metal catalysis. To our surprise, the desired reactions also worked when benzoferrocenes with the *α*-positions of the aryl components blocked were subjected to the standard conditions (Fig. 2c, **3s**–**3w**). Less than 10% of the corresponding 2-phosphorylated products could be observed for these substrates. Meanwhile, over phosphorylations and decomposition of substrates resulted in the relatively low yields (**3s**–**3w**). The regioselectivity and molecular structures of **3q** and **3v**′ were unambiguously determined by single-crystal X-ray diffraction analysis.

**Fig. 3:**
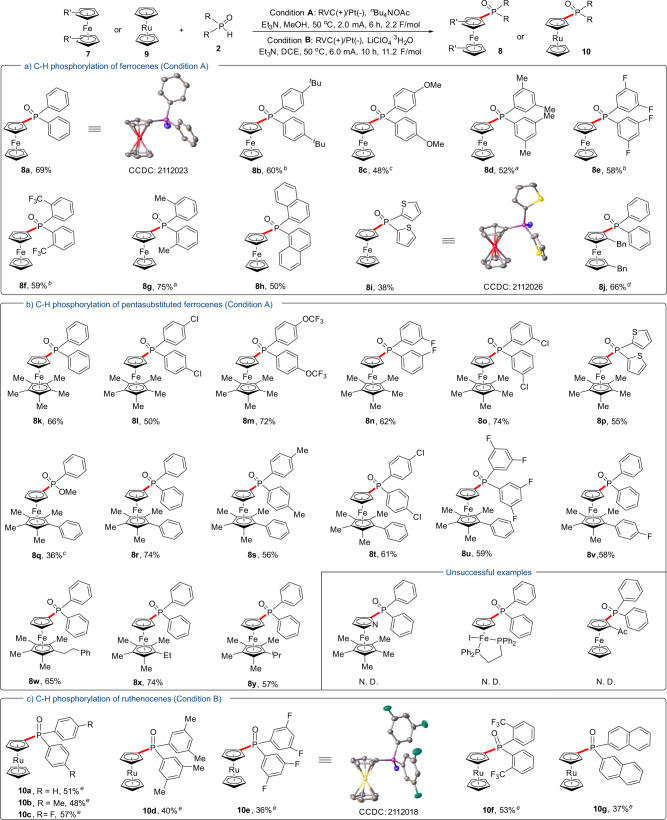
Substrate scope for C−H phosphorylation of ferrocenes and ruthenocenes. **a** C−H phosphorylation of ferrocenes (Condition A). **b** C−H phosphorylation of pentasubstituted ferrocenes (Condition A). **c** C−H phosphorylation of ruthenocenes (Condition B). ^a^Reaction with 2.0 mA, 12 h (4.5 F/mol). ^b^Reaction with 2.5 mA, 12 h (5.6 F/mol). ^c^Reaction with 4.0 mA, 12 h (9.0 F/mol). ^d^Regioselectivity *o*:*m* = 1:2.8. ^e^Reaction with 6.0 mA, 10 h (11.2 F/mol).

Additionally, the current electrochemical protocol was also suitable for the phosphorylation of simple ferrocene substrates (Fig. 3a). Substrates bearing either electron-donating or electron-withdrawing groups at the phenyl rings of phosphine oxides were all tolerated and gave the phosphorylated products in 48–75% yields (**8a**–**8g**). 1-Naphylphosphine oxide was applicable to the standard condition as well (**8h**), while subjecting 2-thiophenyl phosphine oxides to the reaction could only deliver the desired product in a 38% yield (**8i**). For the substrate 1,1’-dibenzylferrocene, the products of *o*-phosphorylation and *m*-phosphorylation were isolated respectively in 1:2.8 ratio. Next, pentamethylferrocene and their analogs were tested with various phosphine oxides (Fig. 3b). These reactions proceeded successfully and all occurred at the bare Cp rings, leading to products in 36–74% yields (**8k**–**8y**). Unfortunately, the phosphorylation of ruthenocene initially failed due to its low solubility in MeOH. To overcome this drawback, DCE was chosen as solvent and LiClO_4_•3H_2_O was selected as an electrolyte in this case. Under the modified condition, ruthenocene reacted with a series of phosphine oxides smoothly and delivered products in decent yields (**10a–10g**), which further highlights the generality of the current strategy. The structure of ruthenocenyl phosphine oxide **10e** was also further confirmed by single-crystal X-ray diffraction analysis.

### Mechanistic investigations

**Fig. 4:**
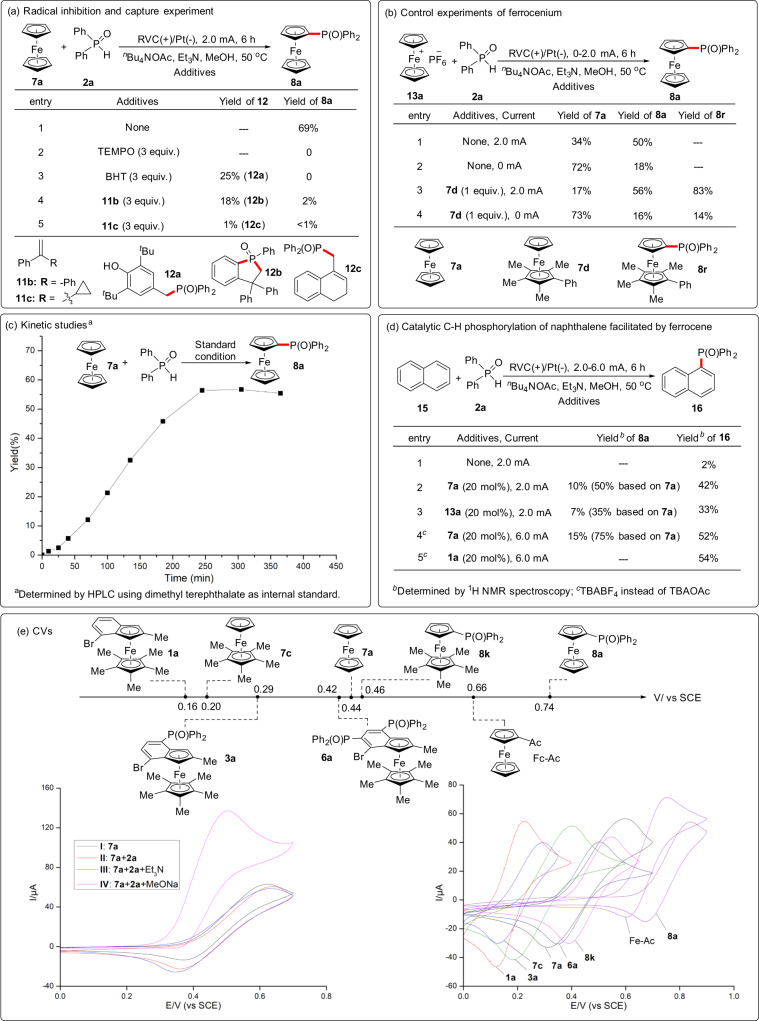
Mechanistic investigations. **a** Radical inhibition and capture experiments. **b** Control experiments of ferrocenium. **c** Kinetic studies. **d** Catalytic C−H phosphorylation of naphthalene facilitated by ferrocene. **e** Cyclic voltammograms (CVs).

To probe the mechanism of this electrochemically enabled C−H phosphorylation of metallocenes, preliminary mechanistic investigations have been conducted (Fig. 4). The addition of radical scavengers TEMPO (2,2,6,6-tetramethylpiperidinoxy) to the reaction mixture almost suppressed the reactivity (Fig. 4a, entry 2 vs 1). Using BHT (butylated hydroxytoluene) and 1,2-diphenylethylene as a radical scavenger, the phosphorylated products **12a** and **12b** were isolated in 25 and 18% yield respectively (entries 3–4)^71^. Besides, radical clock reaction with 2 equiv. of (1-cyclopropylvinyl)benzene (**11c**) led to the ring-opened product **12c** (entry 5)^72^. The above experiments are strongly suggestive of a phosphinyl radical species being involved in this reaction.

In order to get insight into this electrochemical process, control experiments of ferrocenium were carried out (Fig. 4b). Conducting electrolysis by replacing ferrocene with ferrocenium **13a** under the standard conditions gave a 50% yield of **8a** accompanied by **7a** (entry 1). Notably, an 18% yield of **8a** could be obtained without electricity. A similar phenomenon was also observed in the control experiments with **7d** (entries 3–4). Interestingly, some amount of phosphorylation product **8r** was still obtained in the absence of electricity. These results suggest that this phosphorylation may undergo through the ferrocenium species. Kinetic studies on the coupling of **7a** with **2a** showed phosphorylation product **8a** was generated continuously at the first 4h (Fig. 4c).

Surprisingly, a small amount of **16** was observed when the kinetic studies of phosphorylation of **7a** were performed using naphthalene as the internal standard (Fig. 4c, d). Only 2% phosphorylated naphthalene could be obtained without **7a** under standard conditions (Fig. 4d, entry 1). The addition of a catalytic amount of ferrocene **7a** or ferrocenium **13a** helped the formation of **16** (entries 2 and 3). Through rough optimization with ferrocene as a redox catalyst, the yield of **16** was improved to 54% (entries 4 and 5). Therefore, the above experiments suggested that the ferrocene could act as a redox catalyst in phosphorylation.

Next, cyclic voltammograms (CVs) of **7a** were conducted under different conditions to gain mechanistic insight (Fig. 4e). The oxidation potential of ferrocene **7a** was 0.44 V (curve I). Although compound **2a** exhibited no apparent peak from 0 to 1.6 V (Supplementary Fig. 1, curve V, Supporting Information)^50, 63, 69^, **2a** showed an oxidative potential at 1.25 V in the presence of the base NaOMe (Supplementary Fig. 1, curve VI, Supporting Information). No significant change on the CVs of **7a** was observed in the presence of **2a** (curve II) and adding Et_3_N had also no remarkable change (curve III). However, a catalytic current could be observed when **7a** was mixed with **2a** and NaOMe (curve IV)^39^. A comparison of curves IV and VI (Fig. S1), the voltammogram of the conjugate base of **2a**, suggested that the current increase of curve IV did not result from the oxidation of phosphine oxide anion. The above results suggest that the addition of NaOMe facilitates the oxidation of **2a** with ferrocenium. Notably, MeO^−^ is assumed to be generated at the cathode through the reduction of MeOH. Based on the CVs of the metallocene substrates and products, the observed oxidation potentials could account for the reactivity difference between electron-rich and electron-deficient metallocenes (Fig. 4e).

**Fig. 5:**
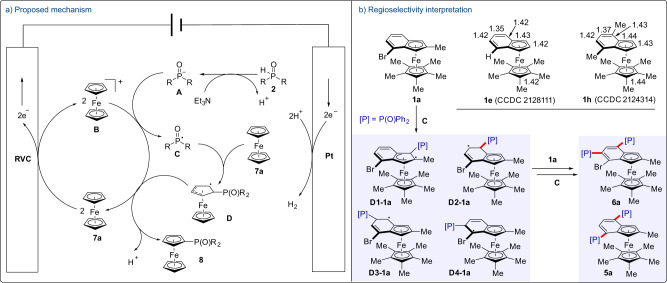
Proposed mechanism. **a** Proposed mechanism. **b** Regioselectivity interpretation.

Based on the experimental results above and literature precedent, a proposed mechanism is shown in Fig. 5. The electrolytic process begins with the anodic oxidation of **7a** that produces ferrocenium ion **B**. Meanwhile, the addition of Et_3_N facilitates deprotonation of **2** for the formation of anion **A**. A first single-electron transfer (SET) between anion **A** and ferrocenium ion **B** generates phosphinyl radical **C** and ferrocene **7a**. Subsequently, the in situ formed phosphinyl radical **C** adds to ferrocene **7a** to yield radical species **D**. A second SET between radical **D** and ferrocenium ion **B** delivers the desired phosphorylation product **8** and regenerates ferrocene **7a** for the next cycle. The hydrogen is released at the cathode. Therefore, an external oxidant is not required in this cross dehydrogenative phosphorylation.

In order to interpret the unexpected high regioselectivity in the phosphorylation of benzoferrocenes **1**, the structural properties of **1e** and **1h** have been shown in Fig. 5b. Some important bond lengths are given according to the single-crystal X-ray diffraction analysis. For these substrates, the bond lengths of C(5)=C(6) and C(7)=C(8) are between 1.35–1.37 Å which are shorter than that of benzene (1.39 Å). The bond lengths for the rest of the C−C bonds on the indenyl motif are ~1.42 Å. These results suggest higher electron density is located around C(5)=C(6) and C(7)=C(8) and makes them more nucleophilic. Taking **1a** as the example for regioselectivity interpretation, the four most likely radical species **D** (**D1–D4** of **1a**) will be expected to form via the reaction of phosphinyl radical **C** with benzoferrocene **1a**. Based on the distribution of electron density on the indenyl motif, **D1-1a** is the least favored species to generate. Owing to the steric hindrance of the bromine atom, the formation of **D4-1a** is not preferred. Stronger ability on the stabling the allylic radical species make **D2-1a** preferentially yielded over **D3-1a**. The electron density distribution on the indenyl motif also results in the formation of **6a** as the major bisphosphorylated side product.

**Fig. 6:**
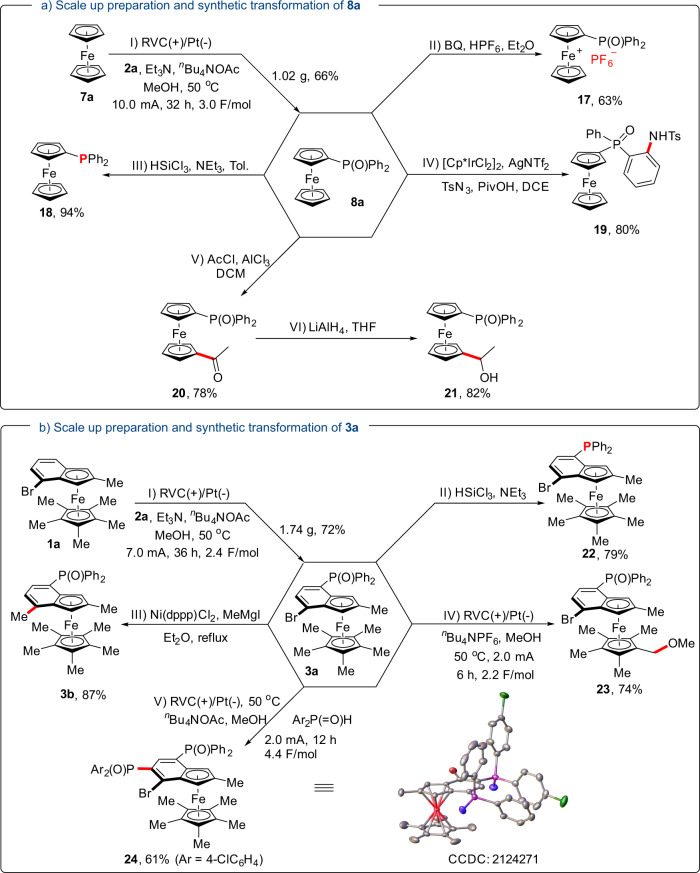
Scale-up preparations and synthetic transformations. **a** Scale-up preparation and synthetic transformation of **8a**. **b** Scale-up preparation and synthetic transformation of **3a**.

Finally, gram-scale preparations of **8a** (1.02 g) and **3a** (1.74 g) were carried out to demonstrate the efficacy of this protocol (Fig. 6a, b). Further synthetic elaborations of these materials were conducted to show the synthetic utility. For instance, a strong single-electron oxidant **17** could be synthesized through the oxidation of **8a** with BQ (1,4-Benzoquinone) in the presence of HPF_6_^73^. Meanwhile, ferrocene-based phosphine **18** could be prepared via reduction of **8a** with HSiCl_3_. Through a selective *sp*^2^ C−H activation, amination product **19** was produced under Ir catalysis^74^. Besides, 1,1′-disubstituted ferrocenyl phosphine oxide **20** could be obtained with high regioselectivity through Friedel–Crafts acylation of **8a**. Further reduction of **20** with LiAlH_4_ delivered alcohol **21**. For the reduction of **3a**, phosphine product **22** was isolated with a bromine atom intact. Additionally, **3b** could be obtained via Ni-catalyzed Kumuda coupling reaction. Interestingly, with ^*n*^Bu_4_NPF_6_ as the electrolyte, a selective *sp*^3^ C−H etherification of **3a** occurred efficiently in MeOH to deliver product **23**^75^. Furthermore, another different phosphinyl moiety can be installed on benzoferrocenes to obtain highly functionalized **24**, which would otherwise be challenging to synthesize via traditional means.

## Discussion

In this work, we have developed an electrochemically driven regioselective cross-coupling of group 8 metallocenes with phosphine oxides. Without preinstalled directing groups or pyrophoric alkyl lithium reagent, over 60 examples of phosphorylated (benzo)ferrocenes and ruthenocene are easily accessed through this intermolecular dehydrogenative C−H phosphorylation. Mechanistic studies suggest that the desired C−P bond is constructed through a radical substitution between phosphoryl radical and metallocene. Meanwhile, the metallocene acts as a single-electron transfer reagent for radical generation and quenching. The high regioselectivity for the C−H phosphorylation of benzoferrocenes is a consequence of the unequal electron density distribution on the indenyl motif.

## Methods

### General procedures for electrochemically driven C−H phosphorylation ferrocenes or benzoferrocences

(Method A): In an oven-dried undivided three-necked flask equipped with a stir bar, ferrocenes or benzoferrocenes (0.20 mmol), diphenyl phosphine oxide (0.40 mmol), Et_3_N (0.40 mmol), ^*n*^Bu_4_NOAc (0.20 mmol), and MeOH (4 mL) were combined. The flask was equipped with RVC (100 PPI, 15 mm × 10 mm × 5 mm) as the anode and platinum plate (10 mm × 10 mm × 0.3 mm) as the cathode under an inert atmosphere in a nitrogen glove box. The flask was capped with a septum. The reaction mixture was stirred and electrolyzed at a constant current of 2.0–4.0 mA at 50 ^o^C for 6–12 h. Upon reaction completion, the reaction crude was washed with water and the product was extracted with dichloromethane (10 × 3 mL). The organic layers were combined, dried over Na_2_SO_4_, and concentrated. The pure product was obtained after flash column chromatography on silica gel (petroleum: ethyl acetate = 1:1–4:1).

### General procedures for electrochemically driven C−H phosphorylation ruthenocene

(Method B): In an oven-dried undivided three-necked flask equipped with a stir bar, ruthenocenes (0.20 mmol), diphenyl phosphine oxide (0.40 mmol), Et_3_N (0.40 mmol), LiClO_4_•3H_2_O (0.30 mmol), and DCE (4 mL) were combined. The flask was also equipped with RVC (100 PPI, 15 mm × 10 mm × 5 mm) as the anode and platinum plate (10 mm × 10 mm × 0.3 mm) as the cathode under inert atmosphere in a nitrogen glove box. The flask was capped with a septum. The reaction mixture was stirred and electrolyzed at a constant current of 6.0 mA at 50 ^o^C for 10 h. Upon reaction completion, the reaction crude was washed with water and the product was extracted with dichloromethane (10 mL × 3). The organic layers were combined, dried over Na_2_SO_4_, and concentrated. The pure product was obtained after flash column chromatography on silica gel (petroleum: ethyl acetate = 1:1).

## Supplementary information

supplementary information

## Data Availability

The X-ray crystallographic data for compounds **1e**, **1h**, **3q**, **3v’**, **8a**, **8i**, **10e**, and **24** have been deposited in the Cambridge Crystallographic Data Centre (CCDC), under deposition number CCDC 2128111, CCDC 2124314, CCDC 2112016, CCDC 2124274, CCDC 21122023, CCDC 2112026, CCDC 2112018, and CCDC 2124271, respectively [www.ccdc.cam.ac.uk/data_request/cif]. The data that supports the findings of this study, including experimental details and compound characterization, are available within the manuscript and its Supplementary Information files. All data are available from the corresponding author upon request.

## References

[1] Kealy TJ, Pauson PL (1951). A new type of organo-iron compound. Nature.

[2] Eiland PF, Pepinsky R (1952). X-ray examination of iron biscyclopentadienyl. J. Am. Chem. Soc..

[3] Miller, S. A., Tebboth, J. A. & Tremaine, J. F. Dicyclopentadienyliron. *J. Chem. Soc*. 632–635 (1952).

[4] Dunitz JD, Orgel LE (1953). Bis-cyclopentadienyl iron: a molecular sandwich. Nature.

[5] Dunitz JD, Orgel LE, Rich A (1956). The crystal structure of ferrocene. Acta Crystallogr..

[6] Takahashi S, Anzai J-i (2013). Recent progress in ferrocene-modified thin films and nanoparticles for biosensors. Materials.

[7] Pietschnig R (2016). Polymers with pendant ferrocenes. Chem. Soc. Rev..

[8] Braga SS, Silva AMS (2013). A new age for iron: antitumoral ferrocenes. Organometallics.

[9] Sansook S, Hassell-Hart S, Ocasio C, Spencer J (2020). Ferrocenes in medicinal chemistry; a personal perspective. J. Organomet. Chem..

[10] Togni, A. & Halterman, R. L. *Metallocenes: Synthesis, Reactivity, Applications* (Wiley, 1998).

[11] Fu GC (2000). Enantioselective nucleophilic catalysis with “planar-chiral” heterocycles. Acc. Chem. Res..

[12] Dai LX, Tu T, You SL, Deng WP, Hou XL (2003). Asymmetric catalysis with chiral ferrocene ligands. Acc. Chem. Res..

[13] Fu GC (2004). Asymmetric catalysis with “planar-chiral” derivatives of 4-(dimethylamino)pyridine. Acc. Chem. Res..

[14] Siemeling U, Auch TC (2005). 1,1’-Di(heteroatom)-functionalised ferrocenes as [N,N], [O,O] and [S,S] chelate ligands in transition metal chemistry. Chem. Soc. Rev..

[15] Stepnicka, P. *Ferrocenes: Ligands, Materials and Biomolecules* (Wiley, 2008).

[16] Togni, A. & Hayashi, T. *Ferrocenes: Homogeneous Catalysis, Organic Synthesis, Materials Science* (Wiley, 2008).

[17] Dai, L. X. & Hou, X. L. *Chiral Ferrocenes in Asymmetric Catalysis: Synthesis and Applications* (Wiley, 2010).

[18] Schaarschmidt D, Lang H (2013). Selective syntheses of planar-chiral ferrocenes. Organometallics.

[19] Zhang W, Butt N, Liu D (2014). The design and synthesis of planar chiral ligands and their application to asymmetric catalysis. Synlett.

[20] Gao DW, Gu Q, Zheng C, You SL (2017). Synthesis of planar chiral ferrocenes via transition-metal-catalyzed direct C–H bond functionalization. Acc. Chem. Res..

[21] Ruble JC, Fu GC (1996). Chiral π-complexes of heterocycles with transition metals: A versatile new family of nucleophilic catalysts. J. Org. Chem..

[22] Kataoka N, Shelby Q, Stambuli JP, Hartwig JF (2002). Air stable, sterically hindered ferrocenyl dialkylphosphines for palladium-catalyzed C–C, C–N, and C–O bond-forming cross-couplings. J. Org. Chem..

[23] Thimmaiah M, Luck RL, Fang S (2007). Novel benzoferrocenyl chiral ligands: Synthesis and evaluation of their suitability for asymmetric catalysis. J. Organomet. Chem..

[24] Battelle LF, Bau R, Gokel GW, Oyakawa RT, Ugi IK (1973). Stereoselective synthesis. VIII. absolute configuration of a 1,2-disubstituted ferrocene derivative with planar and central elements of chirality and mechanism of stereoselective metalations of optically-active alpha-ferrocenyl tertiary-amines. J. Am. Chem. Soc..

[25] Rebiere F, Riant O, Ricard L, Kagan HB (1993). Asymmetric-synthesis and highly diastereoselective ortho-lithiation of ferrocenyl sulfoxides: application to the synthesis of ferrocenyl derivatives with planar chirality. Angew. Chem. Int. Ed..

[26] Enders D, Peters R, Lochtman R, Raabe G (1999). Asymmetric synthesis of novel ferrocenyl ligands with planar and central chirality. Angew. Chem. Int. Ed..

[27] Bolm C, Kesselgruber M, Muniz K, Raabe G (2000). Diastereoselective synthesis of ferrocenyl sulfoximines with planar and central chirality. Organometallics.

[28] Gao DW, Shi YC, Gu Q, Zhao ZL, You SL (2013). Enantioselective synthesis of planar chiral ferrocenes via palladium-catalyzed direct coupling with arylboronic acids. J. Am. Chem. Soc..

[29] Pi C (2013). Redox of ferrocene controlled asymmetric dehydrogenative Heck reaction via palladium-catalyzed dual C–H bond activation. Chem. Sci..

[30] Deng RX (2014). Palladium-catalyzed intramolecular asymmetric C–H functionalization/cyclization reaction of metallocenes: an efficient approach toward the synthesis of planar chiral metallocene compounds. J. Am. Chem. Soc..

[31] Shibata T, Shizuno T (2014). Iridium-catalyzed enantioselective C–H alkylation of ferrocenes with alkenes using chiral diene ligands. Angew. Chem. Int. Ed..

[32] Zhang QW, An K, Liu LC, Yue Y, He W (2015). Rhodium-catalyzed enantioselective intramolecular C–H silylation for the syntheses of planar-chiral metallocene siloles. Angew. Chem. Int. Ed..

[33] Zhu D-Y, Chen P, Xia J-B (2016). Synthesis of planar chiral ferrocenes by transition-metal-catalyzed enantioselective C−H activation. ChemCatChem.

[34] Cai ZJ, Liu CX, Gu Q, Zheng C, You SL (2019). Pd-II-catalyzed regio- and enantioselective oxidative C–H/C–H cross-coupling reaction between ferrocenes and azoles. Angew. Chem. Int. Ed..

[35] Chen H, Wang YX, Luan YX, Ye MC (2020). Enantioselective twofold C–H annulation of formamides and alkynes without built-in chelating groups. Angew. Chem. Int. Ed..

[36] Liu C-X, Gu Q, You S-L (2020). Asymmetric C–H bond functionalization of ferrocenes: new opportunities and challenges. Trends Chem..

[37] Liu L, Song H, Liu YH, Wu LS, Shi BF (2020). Achiral (CpIr)-Ir-x(III)/chiral carboxylic acid catalyzed enantioselective C–H amidation of ferrocenes under mild conditions. Acs Catal..

[38] Lou SJ, Zhuo QD, Nishiura M, Luo G, Hou ZM (2021). Enantioselective C–H alkenylation of ferrocenes with alkynes by half-sandwich scandium catalyst. J. Am. Chem. Soc..

[39] Zhu L (2016). Electrocatalytic generation of amidyl radicals for olefin hydroamidation: Use of solvent effects to enable anilide oxidation. Angew. Chem. Int. Ed..

[40] Yan M, Kawamata Y, Baran PS (2017). Synthetic organic electrochemical methods since 2000: On the verge of a renaissance. Chem. Rev..

[41] Tang S, Liu Y, Lei A (2018). Electrochemical oxidative cross-coupling with hydrogen evolution: a green and sustainable way for bond formation. Chem.

[42] Yang Q-L, Fang P, Mei T-S (2018). Recent advances in organic electrochemical C–H functionalization. Chin. J. Chem..

[43] Wang H, Gao X, Lv Z, Abdelilah T, Lei A (2019). Recent advances in oxidative R1–H/R2–H cross-coupling with hydrogen evolution via photo-/electrochemistry. Chem. Rev..

[44] Xiong P, Xu HC (2019). Chemistry with electrochemically generated N-centered radicals. Acc. Chem. Res..

[45] Yuan Y, Lei A (2019). Electrochemical oxidative cross-coupling with hydrogen evolution reactions. Acc. Chem. Res..

[46] Yuan Y (2019). Exogenous-oxidant-free electrochemical oxidative C–H phosphonylation with hydrogen evolution. Chem. Commun..

[47] Chang X, Zhang Q, Guo C (2020). Asymmetric electrochemical transformations. Angew. Chem. Int. Ed..

[48] Jiao KJ, Xing YK, Yang QL, Qiu H, Mei TS (2020). Site-selective C–H functionalization via synergistic use of electrochemistry and transition metal catalysis. Acc. Chem. Res..

[49] Meyer TH, Choi I, Tian C, Ackermann L (2020). Powering the future: how can electrochemistry make a difference in organic synthesis?. Chem. Sci..

[50] Kurimoto Y, Yamashita J, Mitsudo K, Sato E, Suga S (2021). Electrosynthesis of phosphacycles via dehydrogenative C–P bond formation using DABCO as a mediator. Org. Lett..

[51] Zhu C, Ang NWJ, Meyer TH, Qiu Y, Ackermann L (2021). Organic electrochemistry: molecular syntheses with potential. ACS Cent. Sci..

[52] Zhao Y (2006). Oxidative cross-coupling through double transmetallation: Surprisingly high selectivity for palladium-catalyzed cross-coupling of alkylzinc and alkynylstannanes. J. Am. Chem. Soc..

[53] Li CJ (2009). Cross-dehydrogenative coupling (CDC): exploring C-C bond formations beyond functional group transformations. Acc. Chem. Res..

[54] Chen M, Zheng XL, Li WQ, He J, Lei AW (2010). Palladium-catalyzed aerobic oxidative cross-coupling reactions of terminal alkynes with alkylzinc reagents. J. Am. Chem. Soc..

[55] Le Bras J, Muzart J (2011). Intermolecular dehydrogenative heck reactions. Chem. Rev..

[56] Liu C, Zhang H, Shi W, Lei A (2011). Bond formations between two nucleophiles: transition metal catalyzed oxidative cross-coupling reactions. Chem. Rev..

[57] Yeung CS, Dong VM (2011). Catalytic dehydrogenative cross-coupling: forming carbon-carbon bonds by oxidizing two carbon-hydrogen bonds. Chem. Rev..

[58] Yang Y, Lan J, You J (2017). Oxidative C-H/C-H coupling reactions between two (Hetero)arenes. Chem. Rev..

[59] Khrizanforov M (2016). One-stage synthesis of FcP(O)(OC_2_H_5_)_2_ from ferrocene and α-hydroxyethylphosphonate. RSC Adv..

[60] Yurko EO, Gryaznova TV, Kholin KV, Khrizanforova VV, Budnikova YH (2017). External oxidant-free cross-coupling: electrochemically induced aromatic C-H phosphonation of azoles with dialkyl-H-phosphonates under silver catalysis. Dalton Trans..

[61] Khrizanforova VV, Kholin KV, Khrizanforov MN, Kadirov MK, Budnikova YH (2018). Electrooxidative CH/PH functionalization as a novel way to synthesize benzo[b]phosphole oxides mediated by catalytic amounts of silver acetate. N. J. Chem..

[62] Liu Y, Yi H, Lei A (2018). Oxidation-induced C-H functionalization: a formal way for C-H activation. Chin. J. Chem..

[63] Fu N (2019). New bisoxazoline ligands enable enantioselective electrocatalytic cyanofunctionalization of vinylarenes. J. Am. Chem. Soc..

[64] Wu ZJ (2019). Scalable rhodium(III)-catalyzed aryl C–H phosphorylation enabled by anodic oxidation induced reductive elimination. Angew. Chem. Int. Ed..

[65] Deng Y (2021). Electrochemical regioselective phosphorylation of nitrogen-containing heterocycles and related derivatives. Adv. Synth. Catal..

[66] Sbei N, Martins GM, Shirinfar B, Ahmed N (2020). Electrochemical phosphorylation of organic molecules. Chem. Rec..

[67] Long H (2021). Electrochemical C–H phosphorylation of arenes in continuous flow suitable for late-stage functionalization. Nat. Commun..

[68] Lu L, Li H, Lei A (2021). Oxidative cross-coupling reactions between two nucleophiles. Chin. J. Chem..

[69] Wang S, Xue Q, Guan Z, Ye Y, Lei A (2021). Mn-catalyzed electrooxidative undirected C–H/P–H cross-coupling between aromatics and diphenyl phosphine oxides. ACS Catal..

[70] Zhu P-W, Yang Y-T, Li Y, Zhu J, Wu L (2022). Electrochemical oxidative C-H phosphonylation of thiazole derivatives in ambient conditions. Mol. Catal..

[71] Gou XY (2020). Visible-light-induced ligand-free RuCl3 catalyzed C-H phosphorylation in water. Chem. Commun..

[72] Lei T (2020). Cobaloxime catalysis for enamine phosphorylation with hydrogen evolution. Org. Lett..

[73] Khobragade DA (2012). Acceptor-substituted ferrocenium salts as strong, single-electron oxidants: synthesis, electrochemistry, theoretical investigations, and initial synthetic application. Chem. Eur. J..

[74] Gwon D, Park S, Chang S (2015). Dual role of carboxylic acid additive: mechanistic studies and implication for the asymmetric C–H amidation. Tetrahedron.

[75] Xiong P (2020). Site-selective electrooxidation of methylarenes to aromatic acetals. Nat. Commun..

