# Supplementary material for: Electrochemically driven regioselective C−H phosphorylation of group 8 metallocenes
Source: Nat Commun. 2022 Jun 17;13:3496. doi: 10.1038/s41467-022-31178-7 (PMC9206016; doi:10.1038/s41467-022-31178-7)
Supplement: Supplementary file 1 — supplementary information [file 41467_2022_31178_MOESM1_ESM.pdf]

Supporting Information For:

Electrochemically Driven Regioselective C−H Phosphorylation of Group 8 Metallocenes

Hao Zheng,[†,‡] Chang-Hui Liu,[†,‡] Shi-Yu Guo,[†] Gu-Cheng He,[†,‡] Xiang-Ting Min,[†] Bo-Chao Zhou,[†,‡] Ding-Wei Ji,[†] Yan-Cheng Hu,[†] and Qing-An Chen[†,‡,*]

Dalian Institute of Chemical Physics, Chinese Academy of Sciences
457 Zhongshan Road, Dalian 116023, China
E-mail: qachen@dicp.ac.cn

Table of Contents

1. Supplementary Note 1 .... S1
2. Supplementary Note 2 .... S2
3. Supplementary Note 3 .... S3
3.1. Optimization studies S3
3.2. Cyclic voltammetry studies S4
3.3. Radical capture experiment S5
3.4. General procedure for the synthesis of substrates S8
3.5. Typical procedure for electrochemical C-H phosphorylation of benzoferrocenes S14
3.6. Typical procedure for electrochemical C-H phosphorylation of ferrocenes S21
3.7. Typical procedure for electrochemical C-H phosphorylation of ruthenocene S27
3.8. Derivatizations and scale-up synthesis S29
3.9. X-ray crystal structures S35
3.10. Copies of NMR spectra S43
4. Supplementary References S183

1. Supplementary Note 1

Commercially available reagents were used without further purification. Solvents were treated prior to use according to the standard methods. Unless otherwise stated, all reactions were conducted under inert atmosphere using standard Schlenk techniques or in a nitrogen-filled glove-box. ^{1}H NMR and ^{13}C NMR spectra were recorded at room temperature in $CDCl_3$ or CD_2Cl_2 on 400 MHz or 700MHz instrument with tetramethylsilane (TMS) as internal standard. Flash column chromatography was performed on silica gel (200-300 mesh) or neutral aluminum oxide (200-300

mesh). All reactions were monitored by TLC or NMR analysis. HRMS data was obtained with Agilent 8890-7250 or Agilent 6540 Accurate-MS spectrometer (Q-TOF). Cyclic voltammetry data were measured with a CHI 600E electrochemical workstation. Ferrocene **7a**, phosphine oxide **2a**, bis(3-fluorophenyl)phosphine oxide, bis(3,5-dimethylphenyl)phosphine oxide bis(4-methylphenyl)phosphine oxide, bis(4-*tert*-butylphenyl)phosphine oxide, bis(2-chlorophenyl)phosphine oxide, bis(4-methoxyphenyl)phosphine oxide, bis(2-methylphenyl)phosphine oxide, bis(2-(trifluoromethyl)phenyl)phosphine oxide, bis(naphthalen-2-yl)phosphine oxide, bis(naphthalen-1-yl)phosphine oxide, bis(2-thienyl)phosphine oxide and methyl phenylphosphinate are commercial available.

2. Supplementary Note 2

Electrochemical phosphorylation was carried out using RVC (100PPI, 15 mm×10 mm×5 mm) as the anode and platinum plate (10 mm×10 mm×0.3 mm) as the cathode. The electrochemical reactions were performed in a 10 mL three-necked flask equipped with a RVC electrode, a Pt electrode and a septum. The two electrodes are connected to DC power supply (eTOMMENS, eTM-L603SP) with a current tuning range from 0.1 mA to 3000.0 mA.

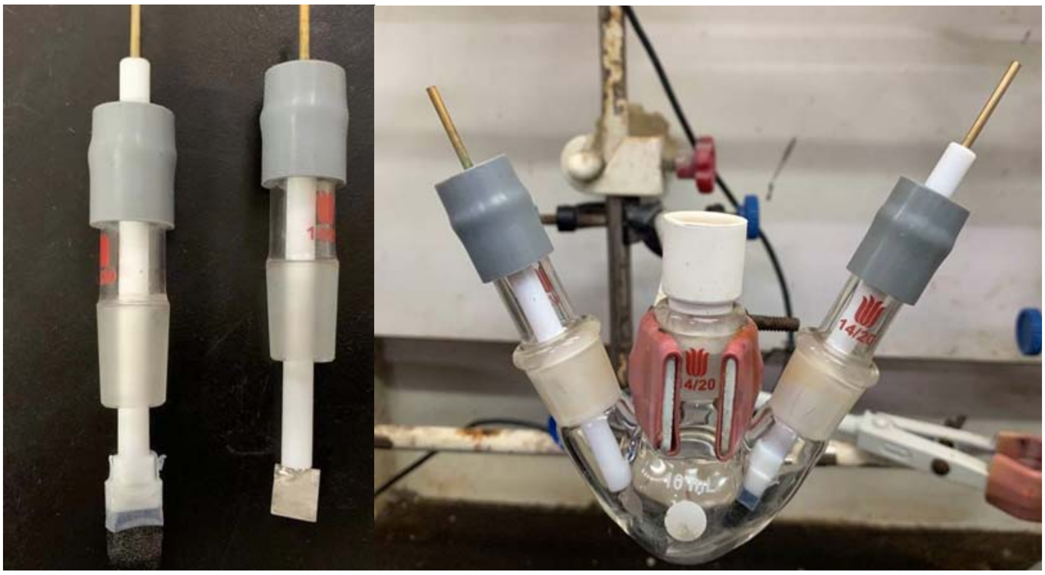

3. Supplementary Note 3

3.1. Optimization studies

Supplementary Table 1. Reactions under stoichiometric oxidants without electricity

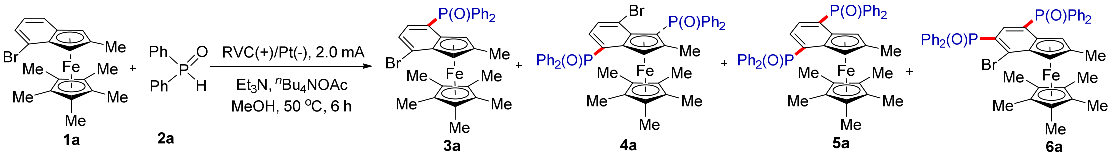

Entry	Deviation from standard conditions	Yield (%)				
		**1a**[c]	**3a**	**4a**	**5a**	**6a**
1	None	8	79(73[b])	1	3	8
2	CuO (2.0 equiv) as oxidant instead of electricity	88	0	0	0	0
3	AgF (2.0 equiv) as oxidant instead of electricity	60	32	0	3	2
4	MnO_2 (2.0 equiv) as oxidant instead of electricity	34	36	<1	1	5
5	$Mn(OAc)_3{\bullet}2H_2O$ (2.0 equiv) as oxidant instead of electricity	42	22	<1	0	5
6	DDQ (2.0 equiv) as oxidant instead of electricity	56	0	0	0	0

[a]Conditions: Undivided cell, constant current (2.0 mA), **1a** (0.20 mmol), **2a** (0.40 mmol), $^nBu_4NOAc$ (0.20 mmol), Et_3N (0.40 mmol), MeOH (4.0 mL), 50 °C, under N_2, 6 h. Yields were determined by ^1H NMR spectroscopy using 1,3,5-trimethoxybenzene as the internal standard. [b]Isolated yield. [c]Recovered yield of **1a**.

Supplementary Table 2. Reactions under stoichiometric oxidants without electricity

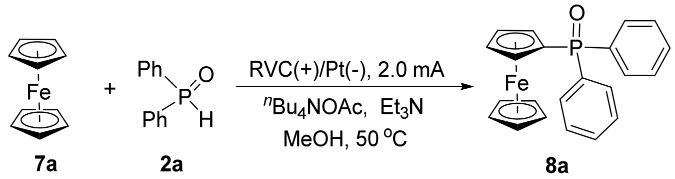

Entry	Deviation from standard conditions	**8a** yield (%)[a]
1	none	72%(69%)[b]
2	without electricity	N. D.
3	without Et_3N	58%
4	C Rod as +	38%
5	Pt plate as +	48%
6	3 mA, 5mA	64%, 50%
7	$^nBu_4NPF_6$ as electrolyte	57%
8	$^nBu_4NClO_4$ as electrolyte	63%
9	NaOAc as base	64%
10	DABCO as base	61%
11	MeCN as solvent	15%

12	TFE as solvent	N. D.
13	DCE as solvent	12%
14	TBHP (2 equiv.) instead of electricity	N. D.
15	DDQ (2 equiv.) instead of electricity	N. D
16	Ag_2CO_3 (2 equiv.) instead of electricity	37%
17	MnO_2 (2 equiv.) instead of electricity	18%
18	$Mn(OAc)_3$•$3H_2O$ (2 equiv.) instead of electricity	24%
19	CuO (2 equiv.) instead of electricity	N. D.

[a]Reaction conditions: Undivided cell, **7a** (0.20 mmol), **2a** (0.40 mmol), ^{n}Bu_4NOAc (0.20 mmol), Et_3N (0.40 mmol), MeOH (4.0 mL), 50 °C, under N_2, 2.0 mA, 6 h, 2.2 F/mol. Yields were determined by ^{1}H NMR spectroscopy using 1,3,5-trimethoxybenzene as the internal standard. [b]Isolated yield.

3.2. Cyclic Voltammetry Studies

The cyclic voltammograms were recorded in an electrolyte solution of ^{n}Bu_4NBF_4 (0.1 M) in MeOH using a glassy carbon disk working electrode (diameter, 1 mm), a Pt wire auxiliary electrode and a SCE reference electrode. The scan rate was 100 mV/s

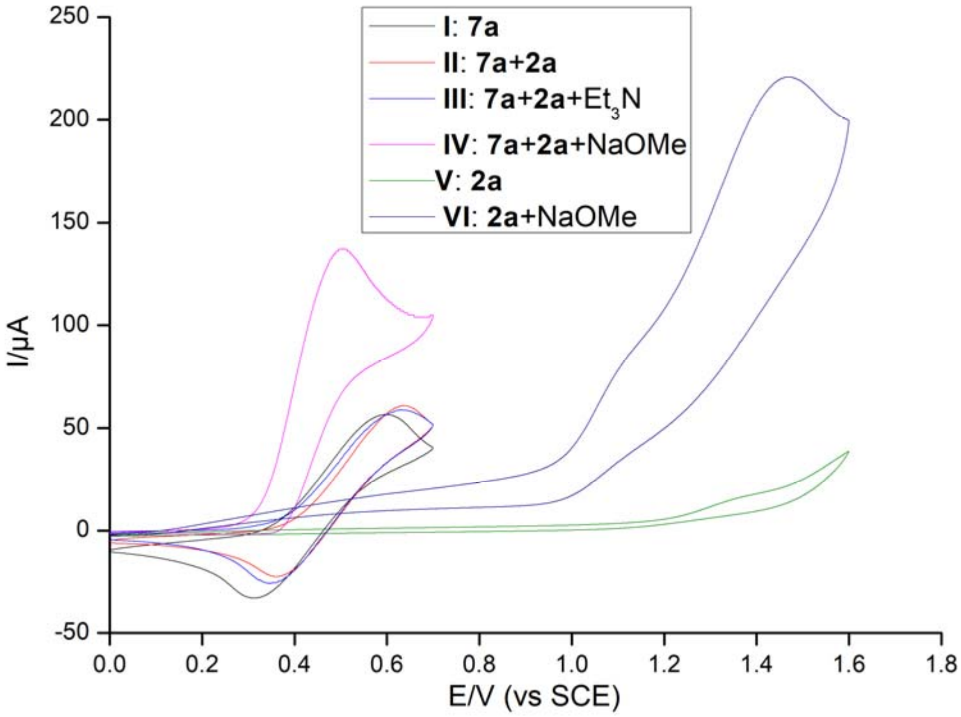

Supplementary Figure 1. Cyclic voltammograms in 0.1 M $TBABF_4$ MeOH solution. I) **7a** (3 mM). **II)** **7a** (3 mM) + **2a** (30 mM). **III)** **7a** (3 mM) + **2a** (30 mM) + Et_3N (60 mM). **IV)** 7a (3 mM) + **2a** (30 mM) + NaOMe (60 mM). **V)** **2a** (30 mM). **VI)** **2a** (30 mM) + NaOMe (60 mM).

3.3. Radical capture experiment

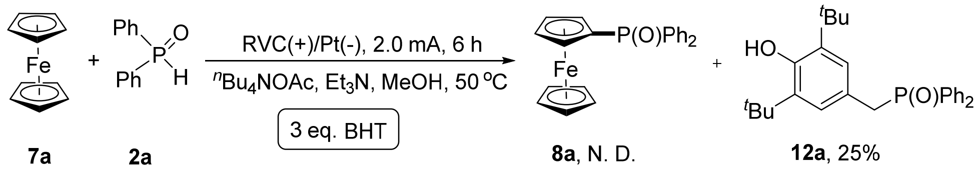

To an oven-dried undivided three-necked flask equipped with a stir bar, **7a** (0.20 mmol), diphenyl phosphine oxide (80.8 mg, 0.40 mmol), ^{n}Bu$_4$NOAc (60.2 mg, 0.20 mmol), MeOH (4 mL), BHT (1,2-butylated hydroxytoluene) (108.2 mg, 0.6 mmol) and Et$_3$N (56 µL, 0.40 mmol) were combined and added in a glove-box. The flask was equipped with RVC (15 mm×10 mm×5 mm) as the anode and platinum plate (10 mm×10 mm×0.3 mm) as the cathode. The reaction mixture was stirred and electrolyzed at a constant current of 2.0 mA under 50 °C for 6 h. When the reaction was finished, concentrated in vacuo, the crude product mixture was purified by silica chromatography (petroleum ether: ethyl acetate =3:4) to afford white solid, known compound, mp 171.6-173.1 °C, 42.0 mg, 25%, ^{1}**H NMR** (400 MHz, CDCl$_3$) δ 7.72 – 7.61 (m, 4H), 7.53 – 7.46 (m, 2H), 7.46 – 7.39 (m, 4H), 6.73 (s, 1H), 5.11 (s, 2H), 3.57 (d, J = 13.8 Hz, 2H), 1.28 (s, 18H). ^{31}**P NMR** (162 MHz, CDCl$_3$) δ 29.9.

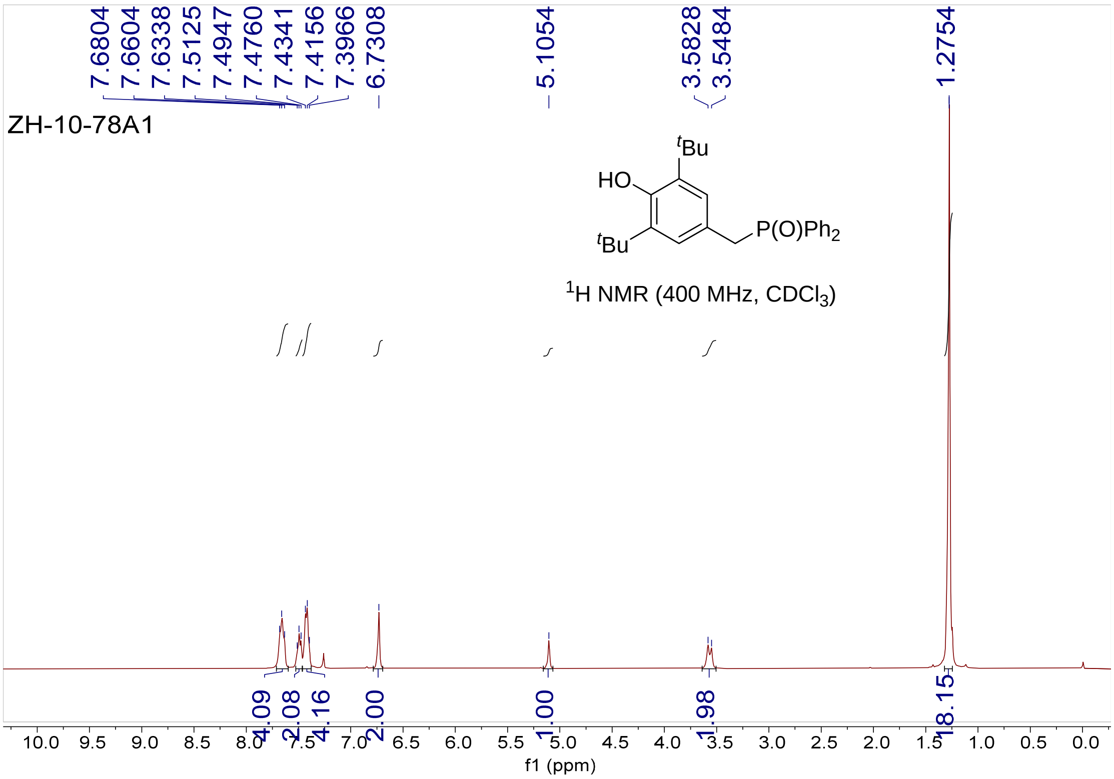

Supplementary Figure 2. ^{1}H NMR spectra of compound 12a

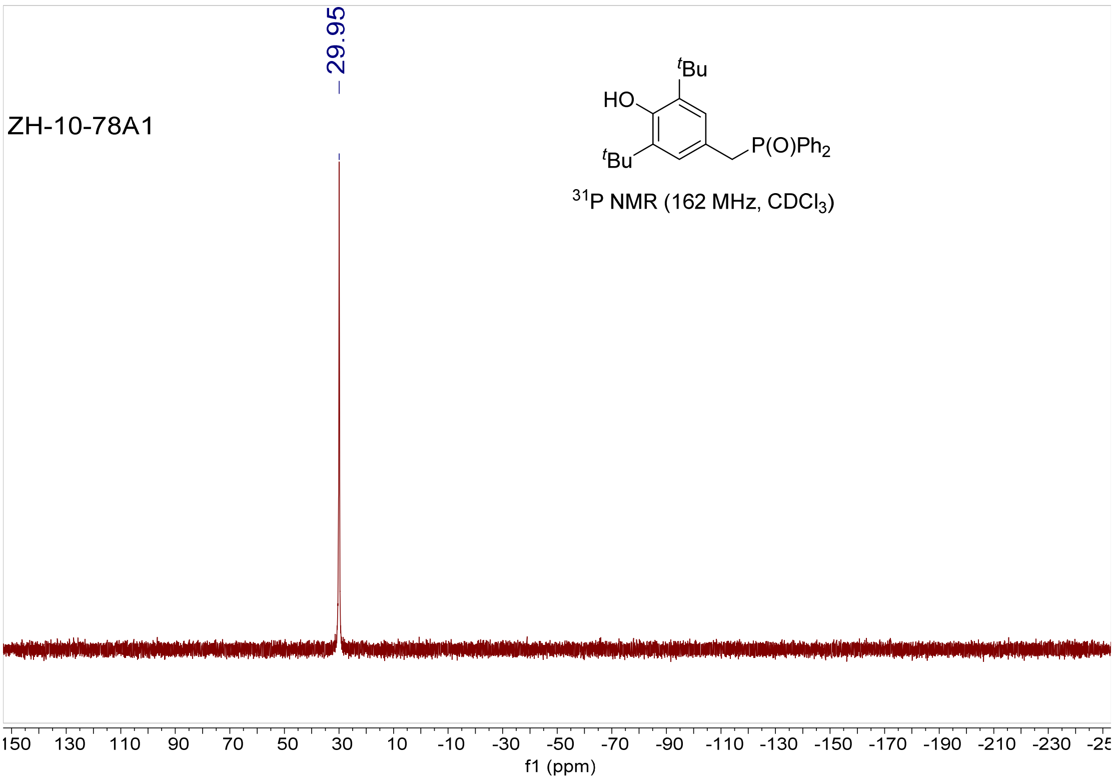

Supplementary Figure 3. ^{31}P NMR spectra of compound 12a

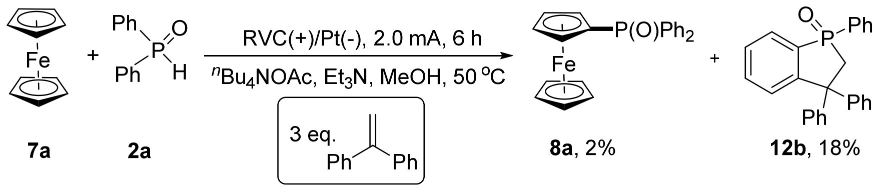

7a **2a** **8a**, 2% **12b**, 18%

To an oven-dried undivided three-necked flask equipped with a stir bar, **7a** (0.20 mmol), diphenyl phosphine oxide (80.8 mg, 0.40 mmol), ^{n}Bu$_4$NOAc (60.2 mg, 0.20 mmol), MeOH (4 mL), 1,2-diphenylethylene (108.2 mg, 0.6 mmol) and Et_3N (56 μL, 0.40 mmol) were combined and added in a glove-box. The flask was equipped with RVC (15 mm×10 mm×5 mm) as the anode and platinum plate (10 mm×10 mm×0.3 mm) as the cathode. The reaction mixture was stirred and electrolyzed at a constant current of 2.0 mA under 50 °C for 6 h. When the reaction was finished, concentrated in vacuo, the crude product mixture was purified by silica chromatography (petroleum ether: ethyl acetate =1:1) to afford **12a** as white solid (known compound,[1] 27.3 mg, 18%) and **8a** (1.2 mg, 2%). **^{1}H NMR** (400 MHz, $CDCl_3$) δ 7.54 – 7.46 (m, 4H), 7.45 – 7.39 (m, 2H), 7.38 – 7.32 (m, 7H), 7.13 – 7.02 (m, 5H), 3.39 (d, J = 9.6 Hz, 2H). **^{31}P NMR** (162 MHz, $CDCl_3$) δ 33.2.

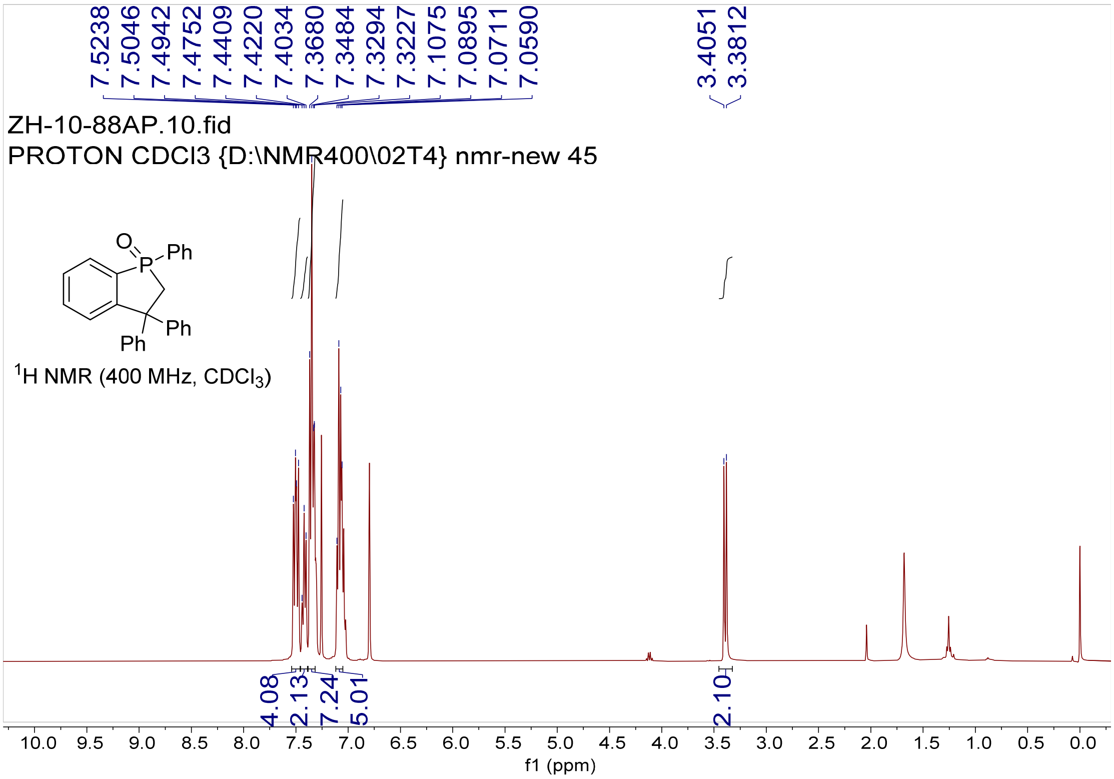

Supplementary Figure 4. ^{1}**H NMR spectra of compound 12b**

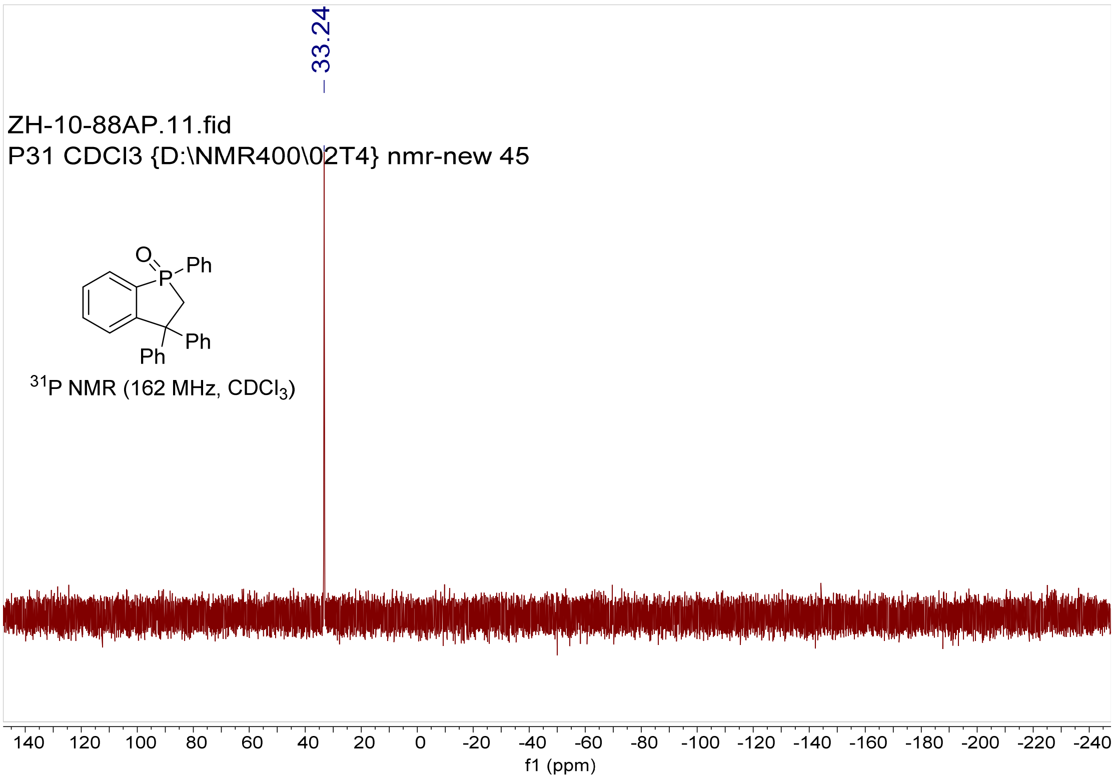

Supplementary Figure 5. ^{31}**P NMR spectra of compound 12b**

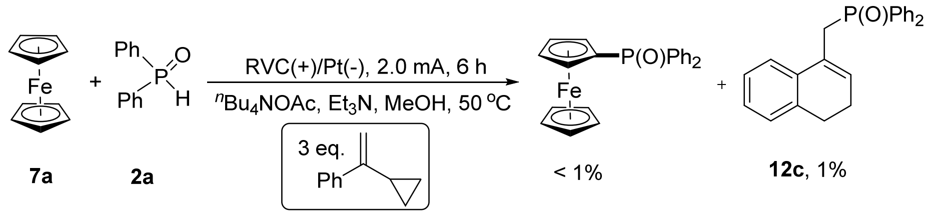

To an oven-dried undivided three-necked flask equipped with a stir bar, **7a** (0.20 mmol), diphenyl phosphine oxide (80.8 mg, 0.40 mmol), ^{n}Bu$_4$NOAc (60.2 mg, 0.20 mmol), MeOH (4 mL), (1-cyclopropylvinyl)benzene (86.5 mg, 0.6 mmol) and Et_3N (56 μL, 0.40 mmol) were combined and added in a glove-box. The flask was equipped with RVC (15 mm×10 mm×5 mm) as the anode and platinum plate (10 mm×10 mm×0.3 mm) as the cathode. The reaction mixture was stirred and electrolyzed at a constant current of 2.0 mA under 50 °C for 6 h. When the reaction was finished, concentrated in vacuo, the crude product mixture was purified by silica chromatography (petroleum ether: ethyl acetate =1:1) to afford the crude by-product. The by-products with cyclopropane-opening were detected by ^{1}H NMR (characteristic signals). Unfortunately, by-product is hard for further purification.[2]

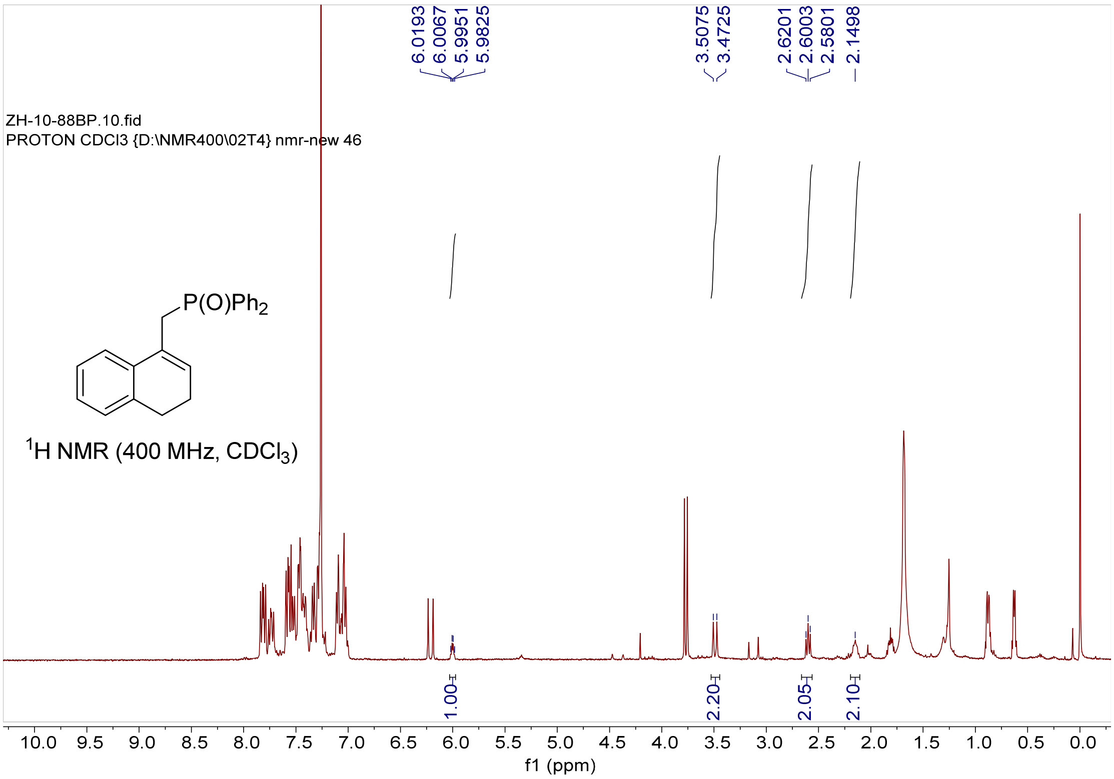

Supplementary Figure 6. ^{1}H NMR spectra of compound 12c

3.4. General procedure for the synthesis of substrates

3.4.1. Synthesis of phosphine oxides

A modification of synthetic protocol from the literature[3]: A three neck 100 mL bottle fitted with stirred bar, reflux condenser and addition funnel was evacuated/N_2 filled (3×), then charged with 4.0 equiv. Mg (0) felt, 15 mL dry THF and a catalytic amount iodine. To this addition funnel was then added a solution of 3.3. equiv. aryl bromide in 10 mL THF. After the addition of a little aryl

bromide, a Grignard reagent was formed and the remaining bromide solution was then added dropwise so as to maintain reflux. When the addition was complete, the mixture was then refluxed for 2 h. After cooling to 0 °C, 1.0 equiv diethylphosphite in 10 mL THF was added dropwise over 15 minutes. The mixture was then stirred 18 h at RT and cautiously quenched with 60 mL 2 N HCl at 0 °C. 100 mL EtOAc was then added, and the mixture was stirred vigorously for 30 min., then the phases were separated. The aqueous phase was extracted with EtOAc (2×20 mL), and the combined EtOAc phases was dried (Na_2SO_4) and the solvent was removed in vacuo to give a yellow oil, which was purified by flash column chromatography (SiO_2, petroleum ether: ethyl acetate = 1:2) to afford phosphine oxides.

Bis(4-(trifluoromethoxy)phenyl)phosphine oxide: Known compound. Colorless oil, 1.93 g, 52% yield. **^{1}H NMR** (700 MHz, $CDCl_3$) δ 8.11 (d, *J* = 489.1 Hz, 1H), 7.76 – 7.72 (m, 4H), 7.37 – 7.32 (m, 4H).

Bis(4-phenylphenyl)phosphine oxide: Known compound. White solid, 1.82 g, 51% yield. **^{1}H NMR** (700 MHz, $CDCl_3$) δ 8.18 (d, *J* = 480.9 Hz, 1H), 7.83 – 7.78 (m, 4H), 7.77 – 7.72 (m, 4H), 7.64 – 7.59 (m, 4H), 7.50 – 7.45 (m, 4H), 7.43 – 7.39 (m, 2H).

Bis(3-chlorophenyl)phosphine oxide: Known compound. Colorless oil, 2.21 g, 81% yield. **^{1}H NMR** (700 MHz, $CDCl_3$) δ 8.03 (d, *J* = 492.4 Hz,), 7.68 – 7.64 (m, 2H), 7.59 – 7.55 (m, 2H), 7.55 – 7.53 (m, 2H), 7.47 – 7.43 (m, 2H).

Bis(3-methoxyphenyl)phosphine oxide: Known compound. Colorless oil, 1.73 g, 66% yield. **^{1}H NMR** (700 MHz, $CDCl_3$) δ 8.03 (d, *J* = 482.8 Hz, 1H), 7.43 – 7.38 (m, 2H), 7.26 (dd, *J* = 14.9, 2.6 Hz, 2H), 7.25 – 7.20 (m, 2H), 7.10 – 7.08 (m, 2H), 3.83 (s, 6H).

Bis(3,5-difluorophenyl)phosphine oxide Known compound. White solid, 2.13 g, 78% yield. **^{1}H NMR** (700 MHz, $CDCl_3$) δ 8.04 (d, *J* = 499.8 Hz, 1H), 7.26 – 7.24 (m, 2H), 7.24 – 7.21 (m, 2H), 7.08 – 7.02 (m, 2H).

3.4.2. Synthesis of metallocenes

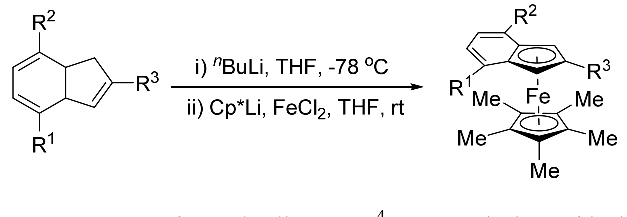

A modification of synthetic protocol from the literature[4]: To a solution of indene (1 equiv.) in THF at -78 °C, *n*-BuLi (2.5 M in hexane, 1.5 equiv.) was added via syringe in a dropwise manner. After addition, the solution was warmed to rt slowly. Pentamethylcyclopentadiene (Cp*–H, 1 equiv.) in THF in another round-bottomed flask was treated with *n*-BuLi (2.5 M in hexane, 1.5 equiv.) at -78 °C, the solution was warmed to rt, giving a white suspension after 2 h. This white suspension was added to a suspension of anhydrous $FeCl_2$ (1 equiv.) in THF with vigorous stirring via a cannula. After stirring at rt for 1 h, the solution of lithiated indene was added dropwise via a cannula, and the resulting deep purple solution was stirred at rt. After 2 h, TLC (SiO_2, petroleum ether)

indicated quantitative conversion. THF was removed under reduced pressure on a rotary evaporator, the deep purple residue was partitioned between 10% K_2CO_3 and petroleum ether and the organic layer was further washed with 10% K_2CO_3 and water. After drying over anhydrous Na_2SO_4, the deep purple solution was concentrated. The crude product mixture was purified by flash column chromatography (neutral Al_2O_3, petroleum ether) to afford product.

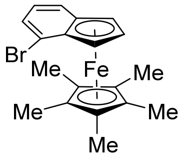

4-Bromoindenyl-pentamethylcyclopentadienyliron (1f): Known compound. Purple solid, 1.31 g, 34% yield. **^{1}H NMR** (700 MHz, $CDCl_3$) δ 7.55 – 7.21 (m, 2H), 6.91 – 6.69 (m, 1H), 4.67 – 4.48 (m, 1H), 4.47 – 4.26 (m, 1H), 3.95 – 3.73 (m, 1H), 1.67 (s, 15H).

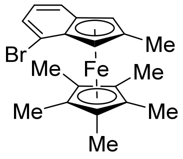

1-Methyl-4-bromoindenyl-pentamethylcyclopentadienyliron (1a): Purple solid, mp 105.9-107.8 °C, 1.93 g, 48% yield. **^{1}H NMR** (400 MHz, $CDCl_3$) δ 7.25 – 7.06 (m, 2H), 6.86 – 6.68 (m, 1H), 4.37 (s, 1H), 4.22 (s, 1H), 1.93 (s, 3H), 1.61 (s, 15H); **^{13}C NMR** (100 MHz, $CDCl_3$) δ 127.2, 124.1, 123.0, 122.4, 90.2, 90.0, 88.9, 77.9, 67.1, 66.6, 13.4, 9.7. **HRMS** calculated for $C_{20}H_{23}BrFe$ $[M]^+$ 398.0333, found 398.0337.

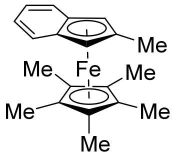

1-Methylindenyl-pentamethylcyclopentadienyliron (1d): Purple solid, mp 65.4-66.7 °C, 476 mg, 15% yield. **^{1}H NMR** (400 MHz, $CDCl_3$) δ 7.21 (d, J = 7.7 Hz, 2H), 6.92 (d, J = 7.1 Hz, 2H), 4.18 (s, 2H), 1.91 (s, 3H), 1.57 (s, 15H); **^{13}C NMR** (100 MHz, $CDCl_3$) δ 127.5, 122.6, 89.1, 88.6, 77.5, 65.7, 13.4, 9.7. **HRMS** calculated for $C_{20}H_{24}Fe$ $[M]^+$ 320.1227, found 320.1227.

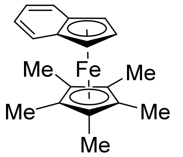

Indenyl-pentamethylcyclopentadienyliron (1e): Red solid, mp 58.8-60.2 °C, 391 mg, 13% yield. **^{1}H NMR** (400 MHz, $CDCl_3$) δ 7.23 (d, J = 7.6 Hz, 2H), 6.93 (d, J = 7.6 Hz, 2H), 4.34 (s, 2H), 3.78 (s, 1H), 1.59 (s, 15H); **^{13}C NMR** (100 MHz, $CDCl_3$) δ 127.3, 122.7, 88.7, 77.9, 76.0, 65.7, 10.2. **HRMS** calculated for $C_{19}H_{22}Fe$ $[M]^+$ 306.1071, found 306.1071.

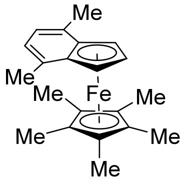

4,7-Dimethylindenyl-pentamethylcyclopentadienyliron (1h): Rose red solid, mp 115.9-117.1 °C, 1.44 g, 43% yield. **^{1}H NMR** (400 MHz, $CDCl_3$) δ 6.62 (s, 2H), 4.35 (s, 2H), 3.77 (s, 1H), 2.37 (s, 6H), 1.57 (s, 15H). **^{13}C NMR** (100 MHz, $CDCl_3$) δ 133.6, 121.07, 90.0, 77.8, 75.6, 64.6, 19.5, 9.9. **HRMS** calculated for $C_{21}H_{26}Fe$ $[M]^+$ 334.1384, found 334.1387.

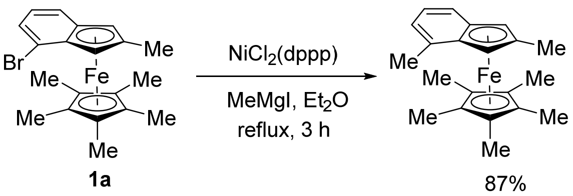

1-Methyl-4-methylindenyl-pentamethylcyclopentadienyliron (1b): A solution of the MeMgI (3M, 2 mL, 6.0 mmol) in Et_2O was added to a stirred solution of **1a** (399.2 mg, 1.0 mmol) and $NiCl_2$(dppp) (27.1 mg, 0.05 mmol) in anhydrous Et_2O (10 mL). The mixture was refluxed for 3 h,

then cooled to 0 °C and quenched with NH_4Cl. This solution was warmed to room temperature. The mixture was diluted with diethyl ether (10 mL) and washed with water (10 mL×3), and the organic layer was dried over Na_2SO_4. The products were concentrated under reduced pressure and the crude product mixture was purified by flash column chromatography (neutral Al_2O_3, petroleum ether) to afford the product as a rose viscous solid, 290.7 mg, 87% yield. **^{1}H NMR** (700 MHz, $CDCl_3$) δ 7.05 (m, 1H), 6.88 (m, 1H), 6.74 (m, 1H), 4.26 (s, 2H), 4.22 (s, 1H), 2.42 (s, 3H), 1.94 (s, 3H), 1.59 (s, 15H); **^{13}C NMR** (175 MHz, $CDCl_3$) δ 137.1, 124.8, 122.8, 120.9, 90.5, 89.5, 88.0, 77.5, 66.1, 64.0, 19.7, 13.5, 9.7. **HRMS** calculated for $C_{21}H_{26}Fe$ $[M]^+$ 334.1384, found 334.1388.

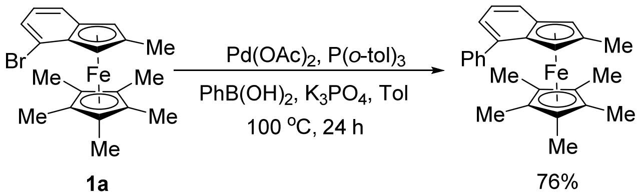

1-Methyl-4-phenylindenyl-pentamethylcyclopentadienyliron (1c): To a sealed tube was sequentially added **1a** (79.8 mg, 0.2 mmol), $Pd(OAc)_2$ (4.5 mg, 0.02 mmol), P(*o*-tol)$_3$ (12.2 mg, 0.04 mmol), K_3PO_4 (127.4 mg, 0.6 mmol), toluene (1.0 mL). The resulting mixture was stirred at 100 °C for 24 h. This solution was warmed to room temperature. The mixture was diluted with diethyl ether (10 mL) and washed with water (10 mL×3), and the organic layer was dried over Na_2SO_4. The products were concentrated under reduced pressure and the residue was purified by flash column chromatography (silica gel, petroleum ether) to afford product as a purple solid, mp 111.1-113.0 °C, 60.2 mg, 76% yield. **^{1}H NMR** (400 MHz, $CDCl_3$) δ 7.73 – 7.56 (m, 2H), 7.54 – 7.41 (m, 2H), 7.36 – 7.27 (m, 1H), 6.92 – 6.63 (m, 3H), 5.02 – 4.77 (m, 1H), 4.75 – 4.53 (m, 1H), 1.69 (s, 3H), 1.22 (s, 15H); **^{13}C NMR** (100 MHz, $CDCl_3$) δ 141.3, 140.4, 128.5, 128.3, 127.6, 127.1, 122.6, 121.9, 89.9, 89.3, 89.0, 77.9, 66.0, 65.2, 13.5, 9.7. **HRMS** calculated for $C_{26}H_{28}Fe$ $[M]^+$ 396.1540, found 396.1545.

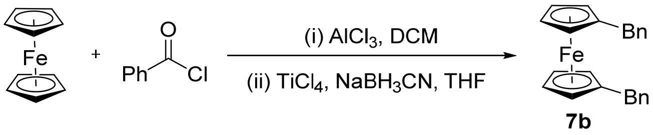

1,1’-Dibenzylferrocene 7b: A modification of synthetic protocol from the literature[5]: To a stirred solution of ferrocene (1.0 g, 5.40 mmol) in CH_2Cl_2 (10 mL) was added $AlCl_3$ (1.8 g, 13.0 mmol) in portions at 0 °C. A solution of the benzoyl chloride (1.7 g, 12.0 mmol) in dichloromethane (10 mL) was added dropwise over 30 min while keeping the temperature at 0 °C, and then the reaction mixture was warmed to room temperature. Upon consumption of the starting material (monitored by TLC), the reaction mixture was quenched with ice water. The organic layer was separated, and the water phase was extracted with dichloromethane (8 mL×3). The combined organic phase was neutralized with a solution of $NaHCO_3$, followed by washing with ice water and saturated brine, and dried over anhydrous Na_2SO_4. After the solvents were removed in vacuo, the crude product was used to reduction without further purification. $TiCl_4$ (0.57 g, 3.0 mmol) was added dropwise by a syringe to a magnetically stirred solution of the above crude product in anhydrous THF (15 mL) at 0 °C. $NaBH_3CN$ (0.38 g, 6.0 mmol) was then added and the resulting mixture was stirred for 10 h at RT. The mixture was then diluted with Et_2O (50 mL), quenched with aqueous ammonia

(30 mL, 2N), the resulting inorganic precipitate was filtered and washed with Et_2O (20 mL). And separation of the organic layer from the filtrate, drying over Na_2SO_4, solvent removal and flash chromatography (silica gel, petroleum ether: ethyl acetate = 1:20) to afford **7b**. Known compound. Yellow solid, 1.1 g, 54% yield. **^{1}H NMR** (400 MHz, $CDCl_3$) δ 7.36 – 7.22 (m, 5H), 7.21 – 7.10 (m, 5H), 4.04 (s, 8H), 3.66 (s, 4H).

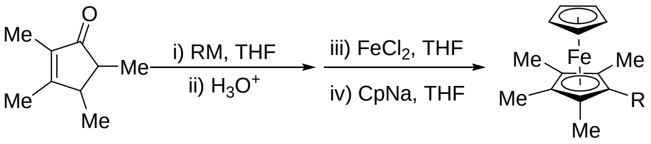

A modification of synthetic protocol from the literature[6,7]: A solution of the corresponding Grignard reagent (1.5 equiv.) in THF was added to a stirred solution of 2,3,4,5-tetramethyl-2-cyclopentenone (1.0 equiv.) in anhydrous THF. The mixture was refluxed for 24 h, then cooled to 0 °C and quenched with HCl. This solution was warmed to room temperature and stirred for 2 h. The mixture was diluted with diethyl ether and washed with water, and the obtained organic layer was dried over Na_2SO_4. The obtained mixtures were concentrated under reduced pressure and without further purification. And *n*-BuLi (2.5 M in hexane, 1 equiv.) was added to a stirred solution of the above dienes in anhydrous THF at -78 ^oC, were warmed to rt, stirred for 2 h and added to the slurry of $FeCl_2$ (1 equiv.) in THF. After 2 h, CpNa (1 equiv.) was added to the above mixture and stirred for 16 h. THF was removed under reduced pressure on a rotary evaporator, the residue was partitioned between saturated NH_4Cl and petroleum ether and the organic layer was further washed with saturated NaCl and water. After drying over anhydrous Na_2SO_4, the solution was concentrated. The crude product mixture was purified by flash column chromatography (neutral Al_2O_3, petroleum ether) to afford respective products.

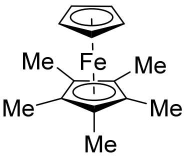

1,2,3,4,5-Pentamethylferrocene (7c): Known compound. Yellow solid, 0.81 g, 31% yield. ^{1}H NMR (700 MHz, $CDCl_3$) δ 3.70 (s, 5H), 1.94 (s, 15H).

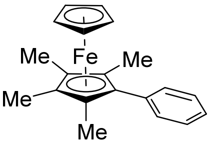

1-Phenyl-2,3,4,5-tetramethylferrocene (7d): Yellow solid, mp 68.7-70.1 ^oC, 1.11 g, 35% yield. **^{1}H NMR** (400 MHz, $CDCl_3$) δ 7.54 – 7.50 (m, 2H), 7.38 – 7.32 (m, 2H), 7.29 – 7.22 (m, 1H), 3.82 (s, 5H), 2.01 (s, 6H), 1.95 (s, 6H); **^{13}C NMR** (100 MHz, $CDCl_3$) δ 138.2, 131.4, 127.6, 126.1, 87.4, 81.3, 80.3, 72.2, 12.3, 11.8. **HRMS** calculated for $C_{20}H_{22}Fe$ $[M]^+$ 318.1071, found 318.1074.

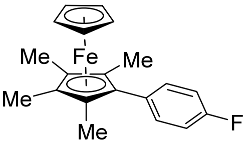

1-(4-Fluoro-phenyl)-2,3,4,5-tetramethylferrocene (7e): Yellow solid, mp 61.0-61.9 ^oC, 483 mg, 24% yield. **^{1}H NMR** (400 MHz, $CDCl_3$) δ 7.48 (dd, *J* = 8.4, 5.5 Hz, 2H), 7.04 (t, *J* = 8.6 Hz, 2H), 3.81 (s, 5H), 2.01 (s, 6H), 1.93 (s, 6H); **^{13}C NMR** (100 MHz, $CDCl_3$) δ 161.4 (d, *J* = 244.8 Hz), 133.9 (d, *J* = 3.2 Hz), 132.7 (d, *J* = 7.8 Hz), 114.5 (d, *J* = 21.1 Hz), 86.8, 81.4, 80.4, 72.3, 12.2, 11.7. **^{19}F NMR** (376 MHz, $CDCl_3$) δ -116.99. **HRMS** calculated for $C_{20}H_{21}F_2Fe$ $[M]^+$ 336.0977, found 336.0978.

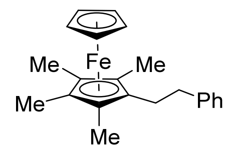

1-Phenethyl-2,3,4,5-tetramethylferrocene (7f): Yellow oil, 1.56 g, 75% yield. **^{1}H NMR** (700 MHz, $CDCl_3$) δ 7.25 (dt, J = 7.7, 3.8 Hz, 2H), 7.18 – 7.15 (m, 1H), 7.15 – 7.12 (m, 2H), 3.65 (s, 5H), 2.70 – 2.63 (m, 2H), 2.57 – 2.52 (m, 2H), 1.89 (s, 6H), 1.84 (s, 6H); **^{13}C NMR** (175 MHz, $CDCl_3$) δ 142.4, 128.5, 128.4, 125.9, 83.9, 80.7, 80.1, 71.3, 38.0, 29.9, 11.6, 11.4. **HRMS** calculated for $C_{22}H_{26}Fe$ $[M]^+$ 346.1384, found 346.1387.

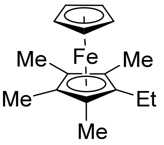

1-Ethyl-2,3,4,5-tetramethylferrocene (7g): Yellow oil, 473 mg, 35% yield. **^{1}H NMR** (400 MHz, $CDCl_3$) δ 3.69 (s, 5H), 2.43 (q, J = 7.6 Hz, 2H), 1.93 (s, 6H), 1.92 (s, 6H), 0.97 (t, J = 7.6 Hz, 3H); **^{13}C NMR** (100 MHz, $CDCl_3$) δ 87.6, 82.0, 81.2, 73.5, 20.0, 14.6, 11.5, 11.3. **HRMS** calculated for $C_{16}H_{22}Fe$ $[M]^+$ 270.1071, found 270.1073.

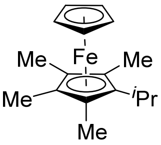

1-Isopropyl-2,3,4,5-tetramethylferrocene (7h): Yellow oil, 365 mg, 96% yield. **^{1}H NMR** (400 MHz, $CDCl_3$) δ 3.76 (s, 5H), 2.76 (hept, J = 7.1 Hz, 1H), 1.92 (s, 12H), 1.30 (d, J = 7.1 Hz, 6H); **^{13}C NMR** (100 MHz, $CDCl_3$) δ 91.4, 80.9, 79.4, 71.6, 27.1, 24.0, 12.1, 11.6. **HRMS** calculated for $C_{17}H_{24}Fe$ $[M]^+$ 284.1227, found 284.1230.

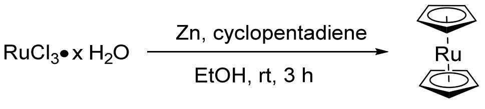

9

Ruthenocene (9): A modification of synthetic protocol from the literature[8]: A two-neck round-bottom flask (100 mL) equipped with a Teflon-coated magnetic stirring bar, was charged with ruthenium trichloride hydrate (2.1 g, 10.0 mmol, 1.0 equiv.) and absolute ethanol (30 mL). The reaction flask was placed in an ice bath, and the reaction mixture was cooled to 0 °C, then cyclopentadiene (8.3 mL, 6.6 g, 100 mmol, 10 equiv.) was added via syringe to the dark red solution. Zinc dust (6.5 g, 100 mmol, 10 equiv.) was added over 60 minutes in 10 portions to the stirred solution, and the temperature was kept between 0 °C and 10 °C during the addition. The reaction mixture was stirred at 0 °C for 30 minutes, then the ice bath was removed, and stirring was continued for 3 hours. The suspension was filtered and the metallic grey solid was washed with hot toluene (100 °C, 4 ×20 mL). The filtrate was concentrated on a rotary evaporation to dryness, and the brown residue was then dissolved in toluene (20 mL) at 23 °C and passed through a plug of silica gel, which was subsequently rinsed with toluene (20 mL). The resulting yellowish solution was concentrated in vacuo to dryness to afford ruthenocene **9** (known compound) as a pale yellow solid, 1.78 g, 77% yield. **^{1}H NMR** (700 MHz, $CDCl_3$) δ 4.56 (s, 10H).

3.5. Typical procedure for electrochemical C-H phosphorylation of benzoferrocenes (Method A)

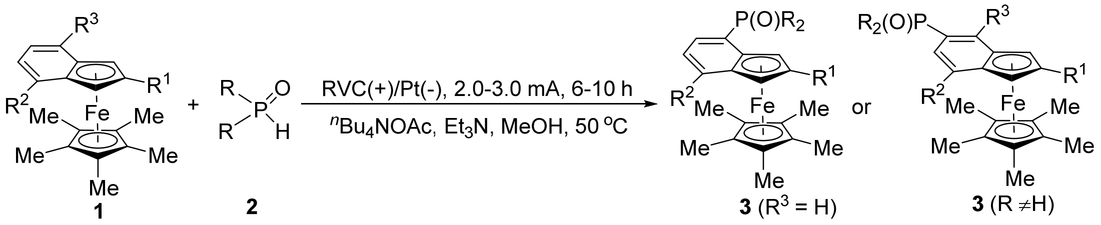

To an oven-dried undivided three-necked flask equipped with a stir bar, benzoferrocene (0.20 mmol), diphenyl phosphine oxide (0.40 mmol), $^{n}Bu_4NOAc$ (0.20 mmol), MeOH (4 mL) and Et_3N (0.40 mmol) were combined and added in a glove-box. The flask was equipped with RVC (15 mm×10 mm×5 mm) as the anode and platinum plate (10 mm×10 mm×0.3 mm) as the cathode. The reaction mixture was stirred and electrolyzed at a constant current of 2.0-3.0 mA under 50 °C for 6 h or 10 h. When the reaction was finished, concentrated in vacuo, the crude product mixture was purified by silica chromatography to afford the product.

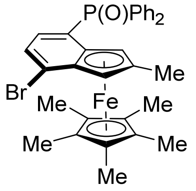

1-Methyl-4-diphenylphosphinyl-7-bromoindenyl-pentamethyl cyclopenta-dienyliron (3a): Purple solid, mp 227.2-229.1 °C, 87.5 mg, 73%. **^{1}H NMR** (400 MHz, $CDCl_3$) δ 7.73 – 7.65 (m, 2H), 7.60 – 7.53 (m, 1H), 7.52 – 7.41 (m, 5H), 7.38 – 7.30 (m, 2H), 7.09 (dd, J = 7.1, 1.9 Hz, 1H), 6.56 (dd, J = 15.5, 7.0 Hz, 2H), 4.83 (s, 1H), 4.28 (s, 1H), 1.86 (s, 3H), 1.68 (s, 15H); **^{13}C NMR** (100 MHz, $CDCl_3$) δ 133.0 (d, J = 104.6 Hz), 132.48 (d, J = 9.5 Hz), 132.46 (d, J = 103.7 Hz), 131.84 (d, J = 2.7 Hz), 131.82 (d, J = 9.9 Hz), 131.7 (d, J = 2.9 Hz), 131.3 (d, J = 3.5 Hz), 130.4 (d, J = 12.3 Hz), 130.0 (d, J = 105.9 Hz), 128.52 (d, J = 12.4 Hz), 128.50 (d, J = 12.1 Hz), 121.7 (d, J = 14.9 Hz), 91.9 (d, J = 9.9 Hz), 90.6, 89.3 (d, J = 8.8 Hz), 78.5, 69.2 (d, J = 2.0 Hz), 66.9, 13.6, 9.6. **^{31}P NMR** (162 MHz, $CDCl_3$) δ 29.4. **HRMS** calculated for $C_{32}H_{32}OPBrNaFe$ $[M+Na]^+$ 621.0616, found 621.0627.

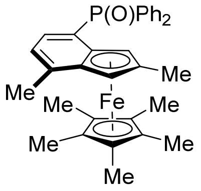

1,7-Dimethyl-4-diphenylphosphinylindenyl-pentamethylcyclopentadienyl iron (3b): Purple solid, mp 221.5-222.3 °C, 62.0 mg, 58% yield, **^{1}H NMR** (400 MHz, CD_2Cl_2) δ 7.61 – 7.55 (m, 2H), 7.45 (dd, J = 7.4, 1.7 Hz, 1H), 7.42 – 7.37 (m, 4H), 7.37 – 7.32 (m, 2H), 7.29 – 7.24 (m, 2H), 6.61 (dd, J = 15.7, 6.7 Hz, 1H), 4.58 (s, 1H), 4.04 (s, 1H), 2.26 (s, 3H), 1.73 (s, 3H), 1.50 (s, 15H). **^{13}C NMR** (100 MHz, CD_2Cl_2) δ 145.6 (d, J = 3.0 Hz), 134.3 (d, J = 103.1 Hz), 133.6 (d, J = 102.5 Hz), 132.6 (d, J = 9.3 Hz), 132.1 (d, J = 9.9 Hz), 131.8 (d, J = 2.8 Hz), 131.7, 131.6 (d, J = 10.0 Hz), 128.7 (d, J = 3.4 Hz), 128.6 (d, J = 3.5 Hz), 127.4 (d, J = 107.8 Hz), 119.0 (d, J = 14.5 Hz), 91.4 (d, J = 9.6 Hz), 89.8 (d, J = 8.5 Hz), 89.6, 78.1, 68.0 (d, J = 2.6 Hz), 64.6, 20.2, 13.7, 9.6. **^{31}P NMR** (162 MHz, CD_2Cl_2) δ 28.3; **HRMS** calculated for $C_{33}H_{35}OPNaFe$ $[M+Na]^+$ 557.1667, found 557.1668.

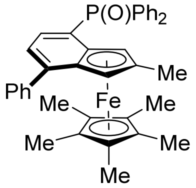

1-Methyl-4-diphenylphosphinyl-7-phenylindenyl-pentamethylcyclopenta-dienyliron (3c): Black green solid, mp 210.2-211.7 °C, 74.3 mg, 56% yield (accompanied by a small amount of ethyl acetate, the yield of the product has been adjusted accordingly). **^{1}H NMR** (400 MHz, $CDCl_3$) δ 7.78 – 7.69 (m, 4H), 7.57 – 7.45 (m, 7H), 7.41 – 7.33 (m, 2H), 6.92 (s, 1H), 6.83 (dd, *J* = 15.6, 6.7 Hz, 1H), 5.00 (s, 1H), 4.49 (s, 1H), 1.83 (s, 3H), 1.50 (s, 15H). **^{13}C NMR** (100 MHz, $CDCl_3$) δ 147.1 (d, *J* = 3.3 Hz), 140.3, 133.6 (d, *J* = 104.3 Hz), 132.9 (d, *J* = 103.2 Hz), 132.5 (d, *J* = 9.4 Hz), 131.9 (d, *J* = 9.8 Hz), 131.6 (d, *J* = 2.9 Hz), 131.5 (d, *J* = 2.5 Hz), 130.9 (d, *J* = 11.3 Hz), 129.3 (d, *J* = 106.8 Hz), 128.5, 128.4, 128.37, 128.3, 128.27, 128.0, 92.1 (d, *J* = 8.4 Hz), 88.5 (d, *J* = 8.7 Hz), 78.3, 67.9, 65.4, 13.7, 9.6. **^{31}P NMR** (162 MHz, $CDCl_3$) δ 29.4. **HRMS** calculated for $C_{38}H_{38}OPFe$ $[M+H]^+$ 597.2004, found 597.2009.

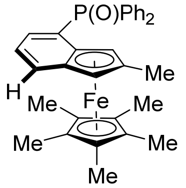

1-Methyl-4-diphenylphosphinylindenyl-pentamethylcyclopentadienyliron (3d): Purple solid, mp 190.4-191.6 °C, 51.7 mg, 46% yield (accompanied by a small amount of ethyl acetate, the yield of the product has been adjusted accordingly). **^{1}H NMR** (400 MHz, $CDCl_3$) δ 7.75 – 7.65 (m, 2H), 7.58 – 7.43 (m, 7H), 7.43 – 7.38 (m, 1H), 7.38 – 7.28 (m, 2H), 6.87 – 6.71 (m, 2H), 4.84 (s, 1H), 4.18 (s, 1H), 1.82 (s, 3H), 1.61 (s, 15H). **^{13}C NMR** (100 MHz, $CDCl_3$) δ 135.1 (d, *J* = 2.5 Hz), 133.5 (d, *J* = 103.8 Hz), 133.0 (d, *J* = 103.2 Hz), 132.5 (d, *J* = 9.2 Hz), 131.9 (d, *J* = 9.9 Hz), 131.6 (d, *J* = 2.6 Hz), 131.5 (d, *J* = 2.8 Hz), 130.9 (d, *J* = 11.3 Hz), 130.1 (d, *J* = 107.3 Hz), 128.4 (d, *J* = 12.0 Hz), 128.3 (d, *J* = 11.9 Hz). 119. 9 (d, *J* = 14.3 Hz), 90.8 (d, *J* = 10.3 Hz), 90.0, 88.0 (d, *J* = 7.3 Hz), 77.9, 67.6, 66.0, 13.5, 9.8. **^{31}P NMR** (162 MHz, $CDCl_3$) δ 29.5. **HRMS** calculated for $C_{32}H_{33}OPNaFe$ $[M+Na]^+$ 543.1511, found 543.1485.

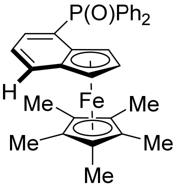

4-Diphenylphosphinylindenyl-pentamethylcyclopentadienyliron (3e): Purple solid, mp 157.9-158.7 °C, 43.2 mg, 39% yield (accompanied by a small amount of ethyl acetate, the yield of the product has been adjusted accordingly). **^{1}H NMR** (400 MHz, $CDCl_3$) δ 7.80 – 7.67 (m, 2H), 7.59 (d, *J* = 8.6 Hz, 1H), 7.56 – 7.44 (m, 5H), 7.43 – 7.38 (m, 1H), 7.36 – 7.28 (m, 2H), 6.89 (t, *J* = 7.7 Hz, 1H), 6.81 (dd, *J* = 15.8, 6.2 Hz, 1H), 4.92 (s, 1H), 4.33 (s, 1H), 3.79 (s, 1H), 1.70 (s, 15H). **^{13}C NMR** (100 MHz, $CDCl_3$) δ 134.9 (d, *J* = 3.1 Hz), 133.3 (d, *J* = 103.9 Hz), 132.7 (d, *J* = 103.4 Hz), 132.4 (d, *J* = 9.5 Hz), 131.8 (d, *J* = 9.8 Hz), 131.6 (d, *J* = 2.7 Hz), 131.4 (d, *J* = 2.8 Hz), 131.0 (d, *J* = 11.3 Hz), 130.1 (d, *J* = 105.8 Hz), 128.40 (d, *J* = 3.4 Hz), 128.36 (d, *J* = 12.2 Hz), 128.32 (d, *J* = 12.0 Hz), 128.28 (d, *J* = 3.0 Hz), 119.9 (d, *J* = 14.3 Hz), 90.1 (d, *J* = 9.2 Hz), 87.4 (d, *J* = 8.2 Hz), 78.4, 78.0, 67.4, 65.9, 10.1. **^{31}P NMR** (162 MHz, $CDCl_3$) δ 26.3. **HRMS** calculated for $C_{31}H_{32}OPFe$ $[M+H]^+$ 507.1535, found 507.1526.

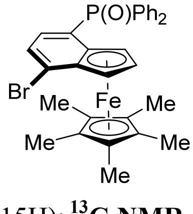

4-Diphenylphosphinyl-7-bromoindenyl-pentamethylcyclopentadienyliron (3f): Purple solid, mp 181.4-182.7 °C, 78.3 mg, 67% yield. **^{1}H NMR** (400 MHz, $CDCl_3$) δ 7.76 – 7.66 (m, 2H), 7.60 – 7.54 (m, 1H), 7.53 – 7.41 (m, 5H), 7.38 – 7.31 (m, 2H), 7.11 (dd, *J* = 7.1, 1.8 Hz, 1H), 6.58 (dd, *J* = 15.4, 7.0 Hz, 1H), 5.00 – 4.95 (m, 1H), 4.50 – 4.45 (m, 1H), 3.86 (t, *J* = 2.6 Hz, 1H), 1.73 (s, 15H); **^{13}C NMR** (175 MHz, $CDCl_3$) δ 132.9 (d, *J* = 104.8 Hz), 132.4 (d, *J* = 9.5 Hz), 132.6, 132.4,

132.3, 132.2 (d, J = 105.84 Hz), 131.8, 131.75, 131.7, 131.6, 131.0, 130.4(d, *J* = 12.1 Hz), 130.2, 129.6, 128.5, 128.4, 121.7 (d, *J* = 14.8 Hz), 91.1 (d, *J* = 9.9 Hz), 88.8 (d, *J* = 8.7 Hz), 79.00, 78.8, 69.0, 66.7, 9.8. **^{31}P NMR** (162 MHz, $CDCl_3$) δ 28.9. **HRMS** calculated for $C_{31}H_{31}OBrPFe$ $[M+H]^+$ 585.0640, found 585.0642.

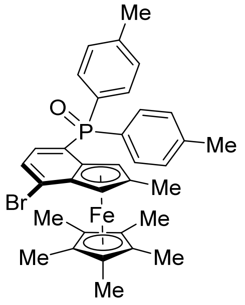

1-Methyl-4-bis(4-methylphenyl)phosphinyl-7-bromoindenyl-pentamethylcyclopentadienyliron (3h): Purple gum, 84.0 mg, 67% yield. **^{1}H NMR** (400 MHz, $CDCl_3$) δ 7.57 (dd, *J* = 11.6, 7.8 Hz, 2H), 7.37 – 7.27 (m, 4H), 7.13 (dd, *J* = 8.0, 2.6 Hz, 2H), 7.08 (dd, *J* = 7.1, 1.8 Hz, 1H), 6.55 (dd, *J* = 15.4, 7.1 Hz, 1H), 4.86 (s, 1H), 4.27 (s, 1H), 2.42 (s, 3H), 2.32 (s, 3H), 1.86 (s, 3H), 1.68 (s, 15H). **^{13}C NMR** (100 MHz, $CDCl_3$) δ 142.1 (d, *J* = 2.8 Hz), 141.9 (d, *J* = 2.8 Hz), 132.5 (d, *J* = 10.0 Hz), 131.8 (d, *J* = 10.5 Hz), 130.6 (d, *J* = 105.4 Hz), 130.9 (d, *J* = 3.5 Hz), 130.2 (d, *J* = 12.2 Hz), 130.0 (d, *J* = 106.9 Hz), 129.5 (d, *J* = 106.1 Hz), 129.3 (d, *J* = 2.5 Hz), 129.1 (d, *J* = 2.4 Hz), 121.7 (d, *J* = 14.9 Hz), 91.9 (d, *J* = 9.8 Hz), 90.5, 89.3 (d, *J* = 9.1 Hz), 78.4, 69.2, 66.8, 21.7, 21.7, 13.6, 9.6. **^{31}P NMR** (162 MHz, $CDCl_3$) δ 29.4. **HRMS** calculated for $C_{34}H_{37}OPBrFe$ $[M+H]^+$ 627.1109, found 627.1090.

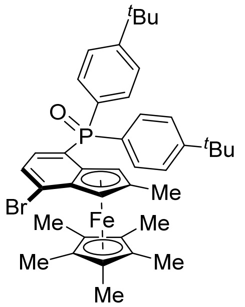

1-Methyl-4-bis(4-*tert*-butylphenyl)phosphinyl-7-bromoindenyl-pentamethylcyclopentadienyliron (3i): Purple solid, mp 168.0-168.8 °C, 94.1 mg, 66% yield. **^{1}H NMR** (700 MHz, $CDCl_3$) δ 7.61 (t, *J* = 9.4 Hz, 2H), 7.49 (d, *J* = 7.9 Hz, 2H), 7.39 (t, *J* = 9.8 Hz, 2H), 7.34 (d, *J* = 7.8 Hz, 2H), 7.10 (d, *J* = 7.1 Hz, 1H), 6.62 (dd, *J* = 15.5, 7.1 Hz, 1H), 4.87 (s, 1H), 4.28 (s, 1H), 1.87 (s, 3H), 1.68 (s, 15H), 1.35 (s, 9H), 1.26 (s, 9H); **^{13}C NMR** (175 MHz, $CDCl_3$) δ 155.1, 154.83, 154.82,132.4 (d, *J* = 9.9 Hz), 131.6 (d, *J* = 10.1 Hz), 130.79, 130.77, 130.6 (d, *J* = 105.4 Hz), 130.29, 130.28, 130.2, 129.7, 129.4 (d, *J* = 106.2 Hz), 125.5 (d, *J* = 12.1 Hz), 121.8 (d, *J* = 14.6 Hz), 92.1 (d, *J* = 10.0 Hz), 90.4, 89.4 (d, *J* = 8.8 Hz). 78.4, 69.2, 66.7, 35.1, 35.0, 31.3, 31.2, 13.7, 9.6. **^{31}P NMR** (162 MHz, $CDCl_3$) δ 29.1. **HRMS** calculated for $C_{40}H_{49}OPBrFe$ $[M+H]^+$ 711.2048, found 711.2048.

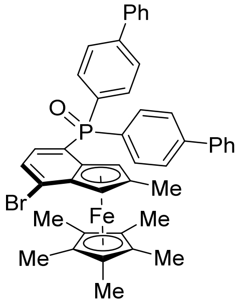

1-Methyl-4-bis(4-phenylphenyl)phosphinyl-7-bromoindenyl-pentamethylcyclopentadienyliron (3j): Purple solid, mp 222.9-224.5 °C, 83.2 mg, 55% yield. **^{1}H NMR** (400 MHz, $CDCl_3$) δ 7.89 – 7.69 (m, 4H), 7.69 – 7.52 (m, 6H), 7.51 – 7.32 (m, 3H), 7.15 (d, *J* = 7.0 Hz, 1H), 6.71 (dd, *J* = 15.8, 7.2 Hz, 1H), 4.93 (s, 1H), 4.33 (s, 1H), 1.90 (s, 3H), 1.72 (s, 15H); **^{13}C NMR** (100 MHz, $CDCl_3$) δ 144.6 (d, *J* = 2.1 Hz), 144.4, 140.1, 133.0 (d, *J* = 9.8 Hz), 132.3 (d, *J* = 9.9 Hz), 131.33, 131.30, 131.2, 131.1 (d, J = 104.89 Hz), 130.4, 130.3, 130.1 (d, J = 106.3 Hz), 129.0 (d, *J* = 9.8 Hz), 128.2 (d, *J* = 13.7 Hz), 127.4, 127.3, 127.1, 121.7 (d, *J* = 15.0 Hz), 91.9 (d, *J* = 9.8 Hz), 90.7, 89.4 (d, *J* = 8.9 Hz), 78.5, 69.2, 66.9, 13.7, 9.6. **^{31}P NMR** (162 MHz, $CDCl_3$) δ 29.1. **HRMS** calculated for $C_{44}H_{41}OPBrFe$ $[M+H]^+$ 751.1422, found 711.1421.

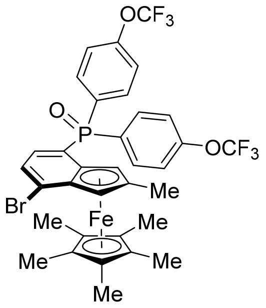

1-Methyl-4-bis(4-trifluoromethoxyphenyl)phosphinyl-7-bromoindenyl-pentamethylcyclopentadienyliron (3k): Purple solid, mp 172.5-173.3 °C, 118.4 mg, 77% yield. **^{1}H NMR** (700 MHz, $CDCl_3$) δ 7.62 (dt, *J* = 163.1, 9.7 Hz, 4H), 7.26 (dd, *J* = 120.5, 8.2 Hz, 4H), 7.11 (d, *J* = 7.1 Hz, 1H), 6.56 (dd, *J* = 15.8, 7.1 Hz, 1H), 4.76 (s, 1H), 4.30 (s, 1H), 1.88 (s, 3H), 1.67 (s, 15H). **^{13}C NMR** (175 MHz, $CDCl_3$) δ 152.1, 152.0, 134.4 (d, *J* = 10.6 Hz), 133.8 (d, *J* = 11.1 Hz), 132.2 (d, *J* = 3.3 Hz), 131.3 (d, *J* = 106.0 Hz), 130.5 (d, *J* = 105.2 Hz), 130.3 (d, *J* = 12.4 Hz), 128.9 (d, *J* = 108.4 Hz), 121.6 (d, *J* = 15.1 Hz), 120.63 (d, *J* = 7.8 Hz), 120.56 (d, *J* = 7.6 Hz), 120.4 (q, *J* = 258.9 Hz), 120.3 (q, *J* = 258.7 Hz), 91.5 (d, *J* = 10.2 Hz), 91.0, 89.4 (d, *J* = 9.0 Hz), 78.5, 69.0, 67.1, 13.6, 9.5. **^{31}P NMR** (162 MHz, $CDCl_3$) δ 27.2. **^{19}F NMR** (376 MHz, $CDCl_3$) δ -57.5, -57.6. **HRMS** calculated for $C_{34}H_{30}OPBrF_6NaFe$ $[M+Na]^+$ 789.0262, found 789.0260.

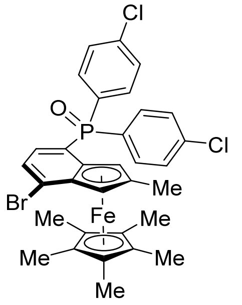

1-Methyl-4-bis(4-chlorophenyl)phosphinyl-7-bromoindenyl-pentamethylcyclopentadienyliron (3l): Purple solid, mp 235.4-235.9 °C, 93.8 mg, 70% yield. **^{1}H NMR** (400 MHz, $CDCl_3$) δ 7.61 (dd, *J* = 11.2, 8.1 Hz, 2H), 7.48 (dd, *J* = 8.4, 2.2 Hz, 2H), 7.42 – 7.30 (m, 4H), 7.08 (dd, *J* = 7.1, 1.9 Hz, 1H), 6.52 (dd, *J* = 15.7, 7.0 Hz, 1H), 4.77 (s, 1H), 4.31 (s, 1H), 1.87 (s, 3H), 1.64 (s, 15H); **^{13}C NMR** (100 MHz, CDCl3) δ 138.6 (d, *J* = 2.7 Hz), 138.4 (d, *J* = 2.5 Hz), 133.7 (d, *J* = 10.5 Hz), 133.1 (d, *J* = 10.7 Hz), 131.9 (d, *J* = 2.8 Hz), 131.2 (d, *J* = 105.9 Hz), 130.4 (d, *J* = 105.3 Hz).130.2 (d, *J* = 12.4 Hz), 129.0 (d, *J* = 107.6 Hz), 128.94 (d, *J* = 12.7 Hz), 128.91 (d, *J* = 12.4 Hz), 121.5 (d, *J* = 15.2 Hz), 91.4 (d, *J* = 10.4 Hz), 90.8, 89.2 (d, *J* = 9.2 Hz), 78.4, 68.9, 67.0, 13.5, 9.5. **^{31}P NMR** (162 MHz, $CDCl_3$) δ 27.9. **HRMS** calculated for $C_{32}H_{30}OPBrCl_2NaFe$ $[M+Na]^+$ 688.9836, found 688.9840.

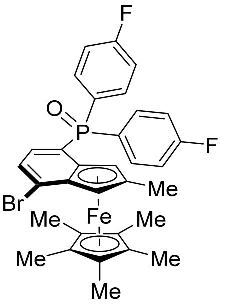

1-Methyl-4-bis(4-fluorophenyl)phosphinyl-7-bromoindenyl-pentamethylcyclopentadienyliron (3m): Purple solid, mp 224.0-226.0 °C, 65.7 mg, 52% yield. **^{1}H NMR** (700 MHz, $CDCl_3$) δ 7.71 – 7.63 (m, 2H), 7.48 – 7.41 (m, 2H), 7.22 – 7.15 (m, 2H), 7.09 (dd, J = 7.0, 1.9 Hz), 7.06 – 7.00 (m, 2H), 6.52 (dd, *J* = 15.7, 7.0 Hz, 1H), 4.77 (s, 1H), 4.29 (s, 1H), 1.87 (s, 3H), 1.66 (s, 15H); **^{13}C NMR** (175 MHz, $CDCl_3$) δ 165.0 (dd, *J* = 253.6, 3.1 Hz), 164.9 (dd, *J* = 253.0, 3.0 Hz), 134.7 (dd, *J* = 10.9, 8.6 Hz), 134.1 (dd, *J* = 11.5, 8.7 Hz), 131.7 (d, *J* = 3.3 Hz), 130.2 (d, *J* = 12.2 Hz), 129.5 (d, *J* = 107.9 Hz), 128.7 (dd, *J* = 107.3, 3.1 Hz), 128.1 (dd, *J* = 106.6, 3.2 Hz), 121.5 (d, *J* = 14.8 Hz), 115.90 (dd, *J* = 21.3, 13.4 Hz), 115.87 (dd, *J* = 21.2, 13.2 Hz), 91.5 (d, *J* = 10.1 Hz), 90.7, 89.2 (d, *J* = 8.9 Hz), 78.4, 68.9, 66.9, 13.5, 9.4. **^{31}P NMR** (162 MHz, $CDCl_3$) δ 27.9; **^{19}F NMR** (376 MHz, $CDCl_3$) δ -106.8, -107.1. **HRMS** calculated for $C_{32}H_{31}OPBrF_2Fe$ $[M+H]^+$ 635.0608, found 635.0611.

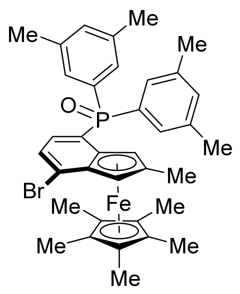

1-Methyl-4-bis(3,5-dimethylphenyl)phosphinyl-7-bromoindenyl-pentamethylcyclopentadienyliron (3n): Purple solid, mp 242.5-242.8 °C, 94.9 mg, 72% yield. **^{1}H NMR** (700 MHz, $CDCl_3$) δ 7.30 (d, *J* = 12.0 Hz, 2H), 7.17 (s, 1H), 7.10 (dd, *J* = 7.1, 1.7 Hz, 1H), 7.07 (d, *J* = 12.3 Hz, 2H), 7.04 (s, 1H), 6.59 (dd, *J* = 15.3, 7.1 Hz, 1H), 4.88 (s, 1H), 4.27 (s, 1H), 2.34 (s, 6H), 2.22 (s, 6H), 1.87 (s, 3H), 1.68 (s, 15H); **^{13}C NMR** (175 MHz, $CDCl_3$) δ 138.03 (d, *J* = 12.6 Hz). 137.99 (d, *J* = 12.7 Hz), 133.50 (d, *J* = 2.8 Hz), 133.47 (d, *J* = 2.9 Hz), 132.9 (d, *J* = 103.7 Hz), 132.5 (d, *J* = 102.7 Hz), 130.8 (d, *J* = 3.2 Hz), 130.4 (d, *J* = 104.4 Hz), 130.3 (d, *J* = 12.2 Hz), 130.1 (d, *J* = 9.3 Hz). 129.3 (d, *J* = 9.6 Hz), 121.8 (d, *J* = 14.7 Hz), 92.1 (d, *J* = 9.9 Hz), 90.4, 89.4 (d, *J* = 8.8 Hz), 78.4, 69.2, 66.7, 21.5, 21.4, 13.7, 9.6; **^{31}P NMR** (162 MHz, $CDCl_3$) δ 29.4; **HRMS** calculated for $C_{36}H_{41}OPBrFe$ $[M+H]^+$ 655.1422, found 655.1431.

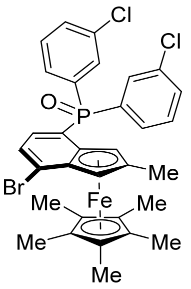

1-Methyl-4-bis(3-chlorophenyl)phosphinyl-7-bromoindenyl-pentamethylcyclopentadienyliron (3o): Purple solid, mp 192.2-193.9 °C, 80.1 mg, 60% yield. **^{1}H NMR** (700 MHz, $CDCl_3$) δ 7.68 (d, *J* = 12.0 Hz, 1H), 7.60 – 7.51 (m, 3H), 7.47 – 7.38 (m, 2H), 7.24 – 7.20 (m, 2H), 7.11 (d, *J* = 7.1 Hz, 1H), 6.58 (dd, *J* = 16.1, 7.1 Hz, 1H), 4.77 (s, 1H), 4.30 (s, 1H), 1.87 (s, 3H), 1.67 (s, 15H); **^{13}C NMR** (175 MHz, $CDCl_3$) δ 135.3, 135.2,135.1, 134.9 (d, *J* = 103.1 Hz), 134.2 (d, *J* = 101.8 Hz), 132.30, 132.28, 132.26, 132.25, 132.1 (d, *J* = 10.4 Hz), 131.6 (d, *J* = 10.5 Hz), 130.5, 130.45, 130.40, 130.0 (d, *J* = 12.9 Hz), 129.7 (d, *J* = 10.0 Hz), 128.5 (d, *J* = 108.1 Hz), 121.6 (d, *J* = 15.1 Hz), 91.4 (d, *J* = 10.2 Hz), 91.0, 89.3 (d, *J* = 9.1 Hz), 78.5, 69.0, 67.1, 13.6; **^{31}P NMR** (162 MHz, $CDCl_3$) δ 27.1. **HRMS** calculated for $C_{32}H_{30}OPBrCl_2NaFe$ $[M+Na]^+$ 688.9836, found 688.9843.

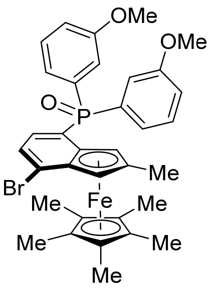

1-Methyl-4-bis(3-methoxyphenyl)phosphinyl-7-bromoindenyl-pentamethylcyclopentadienyliron (3p): Purple solid, mp 215.6-216.9 °C, 99.2 mg, 75% yield. **^{1}H NMR** (700 MHz, $CDCl_3$) δ 7.68 (d, *J* = 12.0 Hz, 1H), 7.60 – 7.51 (m, 3H), 7.47 – 7.38 (m, 2H), 7.24 – 7.20 (m, 2H), 7.11 (d, *J* = 7.1 Hz, 1H), 6.58 (dd, *J* = 16.1, 7.1 Hz, 1H), 4.77 (s, 1H), 4.30 (s, 1H), 1.87 (s, 3H), 1.67 (s, 15H); **^{13}C NMR** (175 MHz, $CDCl_3$) δ 135.3, 135.2,135.1, 134.9 (d, *J* = 103.1 Hz), 134.2 (d, *J* = 101.8 Hz), 132.30, 132.28, 132.26, 132.25, 132.1 (d, *J* = 10.4 Hz), 131.6 (d, *J* = 10.5 Hz), 130.5, 130.45, 130.40, 130.0 (d, *J* = 12.9 Hz), 129.7 (d, *J* = 10.0 Hz), 128.5 (d, *J* = 108.1 Hz), 121.6 (d, *J* = 15.1 Hz), 91.4 (d, *J* = 10.2 Hz), 91.0, 89.3 (d, *J* = 9.1 Hz), 78.5, 69.0, 67.1, 13.6; **^{31}P NMR** (162 MHz, $CDCl_3$) δ 27.1. **HRMS** calculated for $C_{34}H_{36}OPBrNaFe$ $[M+Na]^+$ 681.0827, found 681.0826.

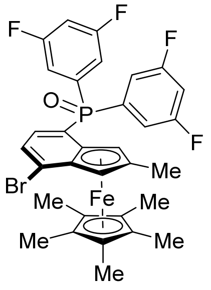

1-Methyl-4-bis(3,5-difluorophenyl)phosphinyl-7-bromoindenyl-pentamethylcyclopentadienyliron (3q): Purple solid, mp 183.0-184.2 °C, 92.6 mg, 69% yield. **^{1}H NMR** (400 MHz, $CDCl_3$) δ 7.22 (dd, *J* = 12.6, 5.3 Hz, 3H), 7.14 (dd, *J* = 7.2, 2.0 Hz, 1H), 7.09 – 6.96 (m, 3H), 6.96 – 6.89 (m, 1H), 6.60 (dd, *J* = 16.1, 7.1 Hz, 1H), 4.73 (s, 1H), 4.33 (s, 1H), 1.90 (s, 3H), 1.66 (s, 15H). **^{13}C NMR** (175 MHz, $CDCl_3$) δ 163.13 (ddd, *J* = 254.3, 19.8, 10.8 Hz). 163.06 (ddd, *J* = 254.3, 19.6, 10.8 Hz). 136.5 (dt, *J* = 104.0, 6.6 Hz),

135.6 (dt, J = 102.5, 6.6 Hz), 133.2 (d, J = 3.7 Hz), 130.5 (d, J = 12.6 Hz), 127.1 (d, J = 110.8 Hz), 121.5 (d, J = 15.3 Hz), 115.3 (ddd, J = 20.8, 10.0, 5.1 Hz), 114.7 (ddd, J = 20.6, 10.6, 5.0 Hz), 108.1 (td, J = 25.1, 1.8 Hz), 91.3, 91.2 (d, J = 10.3 Hz), 89.4 (d, J = 9.4 Hz), 78.6, 68.8 (d, J = 2.2 Hz), 67.4, 13.6, 9.6; **^{31}P NMR** (162 MHz, $CDCl_3$) δ 26.2 (p, J = 6.3 Hz); **^{19}F NMR** (376 MHz, $CDCl_3$) δ -106.5 (d, J = 6.6 Hz), -106.6 (d, J = 6.5 Hz). **HRMS** calculated for $C_{32}H_{28}OPBrF_4NaFe$ $[M+Na]^+$ 693.0239, found 693.0235.

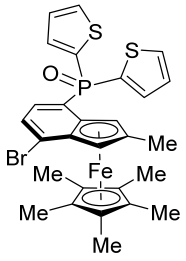

1-Methyl-4-bis(thiophen-2-yl)phosphinyl-7-bromoindenyl-pentamethylcyclopentadienyliron (3r): Purple solid, mp 216.8-218.8 ^{o}C, 79.6 mg, 65% yield, **^{1}H NMR** (400 MHz, $CDCl_3$) δ 7.79 (t, J = 4.5 Hz, 1H), 7.62 (t, J = 4.5 Hz, 1H), 7.49 (dd, J = 7.5, 3.5 Hz, 1H), 7.32 (dd, J = 7.8, 3.6 Hz, 1H), 7.24 – 7.22 (m, 1H), 7.14 – 7.10 (m, 1H), 7.09 – 7.05 (m, 1H), 6.77 (dd, J = 17.1, 7.1 Hz, 1H), 5.03 (s, 1H), 4.33 (s, 1H), 1.90 (s, 3H), 1.66 (s, 15H). **^{13}C NMR** (100 MHz, $CDCl_3$) δ 137.1 (d, J = 10.2 Hz), 136.2 (d, J = 11.1 Hz), 135.2 (d, J = 107.4 Hz), 134.04 (d, J = 110.8 Hz), 134.03 (d, J = 4.6 Hz), 133.7 (d, J = 4.4 Hz), 131.9, 131.0 (d, J = 118.8 Hz), 129.6 (d, J = 13.3 Hz), 128.2 (d, J = 14.5 Hz), 121.7 (d, J = 16.1 Hz), 91.5 (d, J = 11.4 Hz), 90.9, 89.4 (d, J = 9.1 Hz), 78.4, 69.1, 66.9, 13.6, 9.6; **^{31}P NMR** (162 MHz, $CDCl_3$) δ 15.2; **HRMS** calculated for $C_{28}H_{29}OS_2PFe$ $[M+H]^+$ 610.9925, found 610.9931.

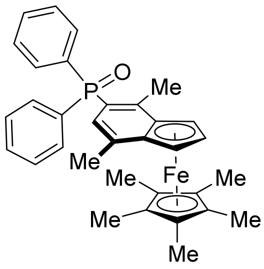

4,7-Dimethyl-5-diphenylphosphinylindenyl-pentamethyl cyclopentadienyliron (3s): Red solid, mp 217.6-218.7 ^{o}C, 49.2 mg, 46% yield. **^{1}H NMR** (400 MHz, $CDCl_3$) δ 7.80 – 7.65 (m, 4H), 7.56 – 7.36 (m, 7H), 6.42 (d, J = 11.3 Hz, 1H), 4.51 – 4.42 (m, 1H), 4.40 (d, J = 2.6 Hz, 1H), 3.93 (t, J = 2.7 Hz, 1H), 2.55 (s, 3H), 2.22 (s, 3H), 1.61 (s, 15H); **^{13}C NMR** (100 MHz, $CDCl_3$) δ 146.4 (d, J = 8.5 Hz), 135.2 (d, J = 101.08 Hz), 134.3 (d, J = 102.09 Hz), 132.9 (d, J = 13.8 Hz), 132.07 (d, J = 9.7 Hz), 131.96 (d, J = 10.4 Hz), 131.46 (d, J = 2.2 Hz), 131.43 (d, J = 2.2 Hz), 128.44 (d, J = 11.8 Hz), 128.38 (d, J = 12.0 Hz), 122.8 (d, J = 15.0 Hz), 121.6 (d, J = 106.8 Hz), 90.7, 89.6 (d, J = 14.7 Hz), 78.5, 77.5, 65.8 (d, J = 1.4 Hz), 65.7, 19.7, 18.6 (d, J = 6.1 Hz), 10.0; **^{31}P NMR** (162 MHz, $CDCl_3$) δ 31.5; **HRMS** calculated for $C_{33}H_{36}OPFe$ $[M+H]^+$ 535.1848, found 535.1848.

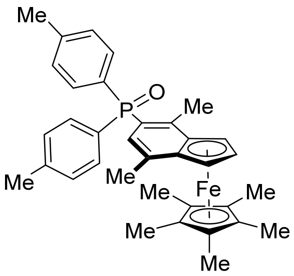

4,7-Dimethyl-5-bis(4-methylphenyl)phosphinylindenyl-pentamethylcyclopentadienyliron (3t): Red solid, mp 217.7-218.9 ^{o}C, 48.4 mg, 43% yield. **^{1}H NMR** (400 MHz, $CDCl_3$) δ 7.58 (dt, J = 12.6, 5.9 Hz, 4H), 7.30 – 7.25 (m, 2H), 7.21 (d, J = 8.0 Hz, 2H), 6.44 (d, J = 11.2 Hz, 1H), 4.48 – 4.42 (m, 1H), 4.42 – 4.37 (m, 1H), 3.92 (t, J = 2.6 Hz, 1H), 2.54 (s, 3H), 2.41 (s, 3H), 2.38 (s, 3H), 2.23 (s, 3H), 1.62 (s, 15H); **^{13}C NMR** (100 MHz, $CDCl_3$) δ 146.1 (d, J = 8.4 Hz), 141.71, 141.68, 141.65, 132.7 (d, J = 13.7 Hz), 132.1, 132.02, 131.97, 131.87, 131.6 (d, J = 103.43 Hz), 131.1 (d, J = 104.77 Hz), 129.2 (d, J = 4.5 Hz), 129.1 (d, J = 4.4 Hz), 123.0 (d, J = 14.4 Hz), 122.1 (d, J = 106.4 Hz), 90.7, 89.7 (d, J = 13.9 Hz), 78.5, 65.8, 65.6, 21.7, 19.6, 18.6 (d, J = 6.1 Hz), 10.0. **^{31}P NMR** (162 MHz, $CDCl_3$) δ 31.7. **HRMS** calculated for $C_{35}H_{40}OPFe$ $[M+H]^+$ 563.2161, found 563.2163.

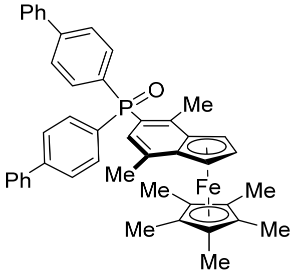

4,7-Dimethyl-5-bis(4-phenylphenyl)phosphinylindenyl-pentamethylcyclopentadienyliron (3u): Red solid, mp 203.8-205.1 °C, 73.8 mg, 54% yield. **^{1}H NMR** (700 MHz, $CDCl_3$) δ 7.85 (m, 4H), 7.74 (d, *J* = 7.7 Hz, 2H), 7.70 – 7.60 (m, 6H), 7.47 (m, 4H), 7.40 (m, 2H), 6.57 (d, *J* = 11.2 Hz, 1H), 4.49 (s, 1H), 4.44 (s, 1H), 3.96 (s, 1H), 2.64 (s, 3H), 2.28 (s, 3H), 1.65 (s, 15H); **^{13}C NMR** (175 MHz, $CDCl_3$) δ 146.5 (d, *J* = 8.5 Hz), 144.1 (d, *J* = 7.7 Hz), 140.2, 133.5 (d, J = 103.65 Hz), 133.00 (d, *J* = 13.6 Hz), 132.96 (d, J = 104.09 Hz), 132.6 (d, *J* = 9.6 Hz), 132.4 (d, *J* = 10.1 Hz), 129.0 (d, *J* = 5.7 Hz), 128.1 (d, *J* = 6.9 Hz), 127.35, 127.33, 127.2, 127.1, 127.0, 122.8 (d, *J* = 14.6 Hz), 121.6 (d, *J* = 106.8 Hz), 90.7, 89.7 (d, *J* = 14.6 Hz), 78.5, 77.6, 65.9, 65.7, 19.7, 18.7 (d, *J* = 5.9 Hz), 10.1. **^{31}P NMR** (162 MHz, $CDCl_3$) δ 31.3. **HRMS** calculated for $C_{45}H_{43}OPNaFe$ $[M+Na]^+$ 709.2293, found 709.2321.

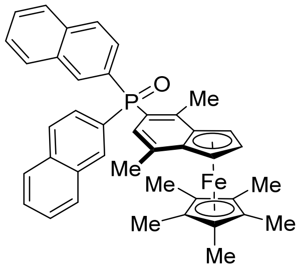

4,7-Dimethyl-5-bis(naphthalen-2-yl)phosphinylindenyl-pentamethylcyclopentadienyliron (3v): Red solid, mp 223.5-224.6 °C, 59.1 mg, 47% yield. **^{1}H NMR** (700 MHz, $CDCl_3$) δ 8.46 – 8.33 (m, 2H), 7.97 – 7.83 (m, 6H), 7.82 – 7.75 (m, 2H), 7.63 – 7.49 (m, 4H), 6.56 (d, *J* = 11.2 Hz, 1H), 4.49 (s, 1H), 4.44 (s, 1H), 3.96 (s, 1H), 2.63 (s, 3H), 2.23 (s, 3H), 1.65 (s, 15H). **^{13}C NMR** (175 MHz, $CDCl_3$) δ 146.7 (d, *J* = 8.4 Hz), 134.7 (d, *J* = 2.2 Hz), 134.6 (d, *J* = 2.1 Hz), 133.6 (d, *J* = 8.9 Hz), 133.3 (d, *J* = 9.5 Hz), 133.0 (d, *J* = 13.8 Hz), 132.7 (d, *J* = 5.0 Hz), 132.6 (d, *J* = 4.6 Hz), 132.2 (d, *J* = 102.2 Hz), 131.6 (d, *J* = 102.9 Hz), 129.1 (d, *J* = 6.5 Hz), 128.3 (d, *J* = 11.8 Hz), 128.1 (d, *J* = 11.6 Hz), 127.4 (d, *J* = 10.1 Hz), 127.2 (d, *J* = 10.4 Hz), 126.8 (d, *J* = 8.2 Hz), 90.7, 89.6 (d, *J* = 14.5 Hz), 78.5, 77.6, 65.9, 65.7, 19.6, 18.8 (d, *J* = 6.2 Hz), 10.1. **^{31}P NMR** (162 MHz, $CDCl_3$) δ 31.2. **HRMS** calculated for $C_{41}H_{40}OPFe$ $[M+H]^+$ 635.2161, found 635.2186.

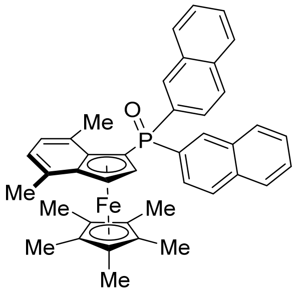

4,7-Dimethyl-2-bis(naphthalen-2-yl)phosphinylindenyl-pentamethylcyclopentadienyliron (3v'): Red solid, Decomposed: 108.5 °C, 12.3 mg, 10% yield. **^{1}H NMR** (400 MHz, $CDCl_3$) δ 8.73 (d, *J* = 13.4 Hz, 1H), 8.03 – 7.97 (m, 1H), 7.92 (d, *J* = 13.5 Hz, 1H), 7.88 – 7.83 (m, 1H), 7.81 – 7.72 (m, 2H), 7.72 – 7.65 (m, 2H), 7.63 – 7.53 (m, 3H), 7.47 (t, *J* = 7.4 Hz, 1H), 7.41 (t, *J* = 7.4 Hz, 1H), 7.29 – 7.22 (m, 1H), 6.83 (d, *J* = 6.5 Hz, 1H), 6.63 (d, *J* = 6.5 Hz, 1H), 4.63 (t, *J* = 2.2 Hz, 1H), 3.69 (t, *J* = 2.9 Hz, 1H), 2.44 (s, 3H), 2.32 (s, 3H), 1.61 (s, 15H). **^{31}P NMR** (162 MHz, $CDCl_3$) δ 30.1. **HRMS** calculated for $C_{41}H_{40}OPFe$ $[M+H]^+$ 635.2161, found 635.2153.

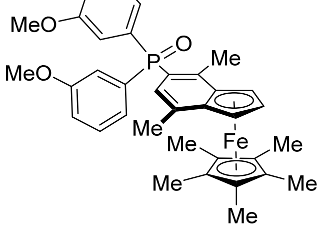

4,7-Dimethyl-5-bis(3-methoxyphenyl)phosphinylindenyl-pentamethylcyclopentadienyliron (3w): Red solid, mp 128.7-129.9 °C, 40.4 mg, 34% yield. **^{1}H NMR** (700 MHz, $CDCl_3$) δ 7.40 – 7.32 (m, 3H), 7.32 – 7.28 (m, 1H), 7.22 – 7.16 (m, 2H), 7.06 (d, *J* = 8.6 Hz, 1H), 7.02 (d, *J* = 8.8 Hz, 1H), 6.40 (d, *J* = 11.7 Hz, 1H), 4.48 – 4.43 (m, 1H), 4.42 – 4.37 (m, 1H), 3.95 – 3.90 (m, 1H), 3.82 (s, 3H), 3.78 (s, 3H), 2.56 (s, 3H), 2.23 (s, 3H), 1.61 (s, 15H). **^{13}C**

NMR (175 MHz, $CDCl_3$) δ 159.63 (d, *J* = 14.2 Hz), 159.59 (d, *J* = 14.6 Hz), 146.5 (d, *J* = 8.9 Hz), 136.1 (d, *J* = 101.2 Hz), 135.7 (d, *J* = 101.8 Hz),132.8 (d, *J* = 13.4 Hz), 129.6 (d, *J* = 6.4 Hz), 129.5 (d, *J* = 5.7 Hz), 124.4 (d, *J* = 9.5 Hz), 124.3 (d, *J* = 10.2 Hz), 122.8 (d, *J* = 15.3 Hz), 121.7 (d, *J* = 106.2 Hz), 117.8 (d, *J* = 3.2 Hz), 117.7 (d, *J* = 2.5 Hz), 116.8 (d, *J* = 10.8 Hz), 116.5 (d, *J* = 10.8 Hz), 90.7, 89.6 (d, *J* = 14.0 Hz), 78.5, 77.5, 65.8, 65.7, 55.52, 55.50, 19.7, 18.7 (d, *J* = 5.7 Hz), 10.1. ^{31}**P NMR** (283 MHz, $CDCl_3$) δ 31.6. **HRMS** calculated for $C_{35}H_{40}O_3PFe$ $[M+H]^+$ 595.2059, found 595.2053.

3.6. Typical procedure for electrochemical C-H phosphorylation of ferrocenes (Method A)

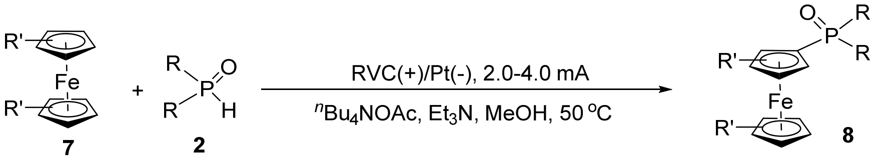

To an oven-dried undivided three-necked flask equipped with a stir bar, ferrocenes (0.20 mmol), diphenyl phosphine oxide (0.40 mmol), ^{n}Bu$_4$NOAc (0.20 mmol), MeOH (4 mL) and Et_3N (0.40 mmol) were combined and added in a glove-box. The flask was equipped with RVC (15 mm×10 mm×5 mm) as the anode and platinum plate (10 mm×10 mm×0.3 mm) as the cathode and. The reaction mixture was stirred and electrolyzed at a constant current of 2.0-4.0 mA under 50 °C for 6-12 h. When the reaction was finished, after filtered through a silica plug and concentrated in vacuo, the crude product mixture was purified by silica chromatography to afford the product.

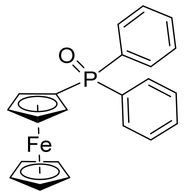

Diphenylphosphinylferrocene (8a): Known compound**,** yellow solid, 53.3 mg, 69% yield. ^{1}**H NMR** (400 MHz, $CDCl_3$) δ 7.71 – 7.63 (m, 4H), 7.48 (m, 2H), 7.41 (m, 4H), 4.46 (d, *J* = 1.8 Hz, 2H), 4.36 (d, *J* = 1.9 Hz, 2H), 4.19 (s, 5H); ^{13}**C NMR** (100 MHz, $CDCl_3$) δ 134.4 (d, *J* = 106.4 Hz), 131.6 (d, *J* = 2.8 Hz), 131.5 (d, *J* = 9.9 Hz), 128.2 (d, *J* = 12.1 Hz), 72.8 (d, *J* = 117.6 Hz), 72.3 (d, *J* = 12.9 Hz), 71.7 (d, *J* = 10.5 Hz), 69.7. ^{31}**P NMR** (162 MHz, $CDCl_3$) δ 29.0;

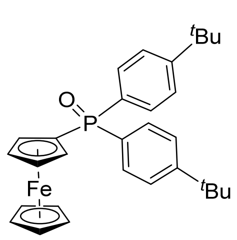

Bis(4-(*tert*-butyl)phenyl)phosphinylferrocene (8b): Yellow solid, mp 223.2-224.2 °C, 59.8 mg, 60% yield. ^{1}**H NMR** (400 MHz, $CDCl_3$) δ 7.60 (dd, *J* = 11.7, 8.1 Hz, 4H), 7.43 (m, 4H), 4.42 (s, 2H), 4.36 (s, 2H), 4.17 (s, 5H), 1.30 (s, 18H). ^{13}**C NMR** (100 MHz, $CDCl_3$) δ 154.8 (d, *J* = 2.8 Hz), 131.36 (d, *J* = 108.6 Hz), 131.36 (d, *J* = 10.1 Hz), 125.1 (d, *J* = 12.3 Hz), 73.7 (d, *J* = 116.9 Hz), 72.3 (d, *J* = 12.8 Hz), 71.4 (d, *J* = 10.3 Hz), 69.7. ^{31}**P NMR** (162 MHz, $CDCl_3$) δ 28.4; **HRMS** calculated for $C_{30}H_{36}OPFe$ $[M+H]^+$ 499.1837, found 499.1848.

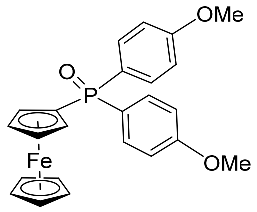

Bis(4-methoxyphenyl)phosphinylferrocene (8c): Yellow solid, mp 205.4-207.2 °C, 43.0 mg, 48% yield. ^{1}**H NMR** (400 MHz, $CDCl_3$) δ 7.57 (dd, *J* = 11.3, 8.6 Hz, 4H), 6.93 (dd, *J* = 8.6, 1.7 Hz, 4H), 4.43 (q, *J* = 1.7 Hz, 2H), 4.32 (q, *J* = 1.8 Hz, 2H), 4.21 (s, 1H), 3.83 (s, 1H); ^{13}**C NMR** (100 MHz, $CDCl_3$) δ 162.0 (d, *J* = 2.8 Hz), 133.3 (d, *J* = 11.3 Hz), 126.1 (d, *J* = 112.7 Hz), 113.6 (d, *J* = 13.0 Hz), 74.1 (d, *J* = 117.6 Hz), 72.2 (d, *J* = 12.9 Hz), 71.4 (d, *J* =

10.4 Hz), 69.6, 55.3; ^{31}**P NMR** (162 MHz, $CDCl_3$) δ 28.5; **HRMS** calculated for $C_{24}H_{23}OPNaFe$ $[M+Na]^+$ 469.0626, found 469.0645.

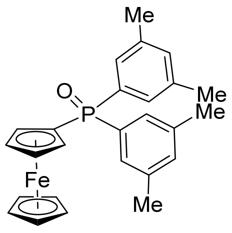

Bis(3,5-dimethylphenyl)phosphinylferrocene (8d): Yellow solid, mp 179.5-181.1 °C, 45.7 mg, 52% yield. ^{1}**H NMR** (700 MHz, $CDCl_3$) δ 7.30 (s, 2H), 7.28 (d, *J* = 2.7 Hz, 2H), 7.11 (s, 2H), 4.44 (q, *J* = 1.8 Hz, 2H), 4.36 (q, *J* = 1.9 Hz, 2H), 4.20 (s, 5H), 2.32 (s, 12H); ^{13}**C NMR** (175 MHz, $CDCl_3$) δ 137.7 (d, *J* = 12.6 Hz), 134.3 (d, *J* = 105.4 Hz), 133.2 (d, *J* = 2.8 Hz), 129.1 (d, *J* = 9.8 Hz), 73.5 (d, *J* = 115.9 Hz), 72.4 (d, *J* = 12.7 Hz), 71.4 (d, *J* = 10.6 Hz), 69.6, 21.4; ^{31}**P NMR** (162 MHz, $CDCl_3$) δ 29.2; **HRMS** calculated for $C_{26}H_{27}OPNaFe$ $[M+Na]^+$ 465.1041, found 465.1028.

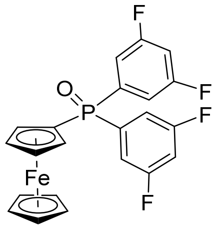

Bis(3,5-difluorophenyl)phosphinylferrocene (8e): Yellow solid, mp 163.5-164.7 °C, 53.1 mg, 58% yield. ^{1}**H NMR** (400 MHz, $CDCl_3$) δ 7.26 – 7.13 (m, 4H), 7.07 – 6.94 (m, 2H), 4.57 (s, 2H), 4.39 (s, 2H), 4.27 (s, 5H); ^{13}**C NMR** (100 MHz, $CDCl_3$) δ 162.80 (ddd, *J* = 254.0, 19.8, 11.1 Hz), 137.7 (dt, *J* = 105.6, 6.8 Hz), 114.42, 114.35, 114.32, 114.24, 114.16, 114.13, 114.06, 107.6 (dt, *J* = 25.0, 1.5 Hz), 72.5 (d, *J* = 11.2 Hz), 72.1 (d, *J* = 13.6 Hz), 69.9, 69.8 (d, J = 123.3 Hz); ^{31}**P NMR** (162 MHz, $CDCl_3$) δ 26.6 (t, *J* = 6.1 Hz). ^{19}**F NMR** (376 MHz, $CDCl_3$) δ -106.8 (d, *J* = 6.3 Hz). **HRMS** calculated for $C_{22}H_{15}OPF_4NaFe$ $[M+Na]^+$ 481.0038, found 481.0038.

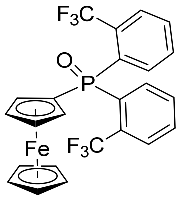

Bis(2-trifluoromethylphenyl)phosphinylferrocene (8f): Yellow solid, mp 163.1-164.0 °C, 61.6 mg, 59% yield. **H NMR** (700 MHz, $CDCl_3$) δ 8.19 (dd, *J* = 14.5, 7.3 Hz, 2H), 7.74 (d, *J* = 6.8 Hz, 2H), 7.65 (t, *J* = 7.7 Hz, 4H), 4.51 (s, 2H), 4.33 (s, 2H), 4.25 (s, 5H); ^{13}**C NMR** (175 MHz, $CDCl_3$) δ 135.8 (d, *J* = 8.3 Hz), 132.7 (d, *J* = 99.3 Hz), 131.7 (d, J =1.9 Hz), 131.5 (dq, *J* = 32.4, 6.1 Hz), 130.9 (d, *J* = 11.5 Hz), 127.26, 127.23, 127.20, 127.18, 127.16, 127.13, 123.6 (dq, *J* = 274.6, 2.8 Hz), 74.3 (d, *J* = 126.9 Hz), 73.4 (d, *J* = 13.4 Hz), 71.7 (d, *J* = 11.1 Hz), 70.2. ^{31}**P NMR** (162 MHz, $CDCl_3$) δ 31.0; ^{19}**F NMR** (376 MHz, $CDCl_3$) δ -56.2. **HRMS** calculated for $C_{24}H_{17}OPF_6NaFe$ $[M+Na]^+$ 545.0163, found 545.0158.

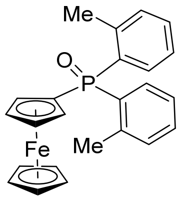

Bis(2-methylphenyl)phosphinylphosphinylferrocene (8g): Yellow solid, mp 222.7-223.8 °C, 61.8 mg, 75% yield. ^{1}**H NMR** (400 MHz, $CDCl_3$) δ 7.71 (ddd, *J* = 13.7, 7.7, 1.4 Hz, 2H), 7.35 (tt, *J* = 7.6, 1.5 Hz, 2H), 7.22 (tt, *J* = 7.6, 1.6 Hz, 2H), 7.14 (dd, *J* = 7.6, 4.5 Hz, 2H), 4.48 (q, *J* = 1.8 Hz, 2H), 4.38 (q, *J* = 1.9 Hz, 2H), 4.23 (s, 5H), 2.20 (s, 6H). ^{13}**C NMR** (101 MHz, $CDCl_3$) δ 141.14 (d, *J* = 9.3 Hz), 133.25 (d, *J* = 10.7 Hz), 133.17 (d, *J* = 103.0 Hz), 131.49 (d, *J* = 1.3 Hz), 131.42 (d, *J* = 9.2 Hz), 125.46 (d, *J* = 12.1 Hz), 73.78 (d, *J* = 116.8 Hz), 72.66 (d, *J* = 12.6 Hz), 71.50 (d, *J* = 10.4 Hz), 69.81, 21.49 (d, *J* = 4.4 Hz); ^{31}**P NMR** (162 MHz, $CDCl_3$) δ 30.1; **HRMS** calculated for $C_{24}H_{23}OPNaFe$ $[M+Na]^+$ 437.0728, found 437.0729.

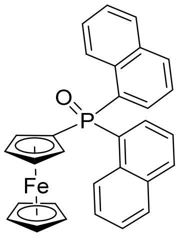

Bis(naphthalen-1-yl)phosphinylferrocene (8h): Yellow solid, mp 256.2-258.0 ^{o}C, 48.6 mg, 50% yield. **^{1}H NMR** (400 MHz, $CDCl_3$) δ 8.68 (d, *J* = 8.5 Hz, 2H), 7.99 – 7.95 (m, 3H), 7.92 (dd, *J* = 7.1, 1.3 Hz, 1H), 7.82 (dd, *J* = 8.1, 1.7 Hz, 2H), 7.52 – 7.45 (m, 2H), 7.44 – 7.38 (m, 2H), 7.34 (ddd, *J* = 8.4, 6.9, 1.5 Hz, 2H), 4.49 (q, *J* = 1.8 Hz, 2H), 4.44 (q, *J* = 1.9 Hz, 2H), 4.11 (s, 5H). **^{13}C NMR** (100 MHz, $CDCl_3$) δ 133.8 (d, *J* = 9.2 Hz), 133.5 (d, *J* = 8.9 Hz), 132.9 (d, *J* = 10.4 Hz), 132.8 (d, *J* = 3.0 Hz), 131.7 (d, *J* = 102.5 Hz), 128.7 (d, *J* = 1.4 Hz), 127.0 (d, *J* = 5.3 Hz), 126.9, 126.2, 124.5 (d, *J* = 13.9 Hz), 74.5 (d, *J* = 119.8 Hz), 72.9 (d, *J* = 12.7 Hz), 71.7 (d, *J* = 10.6 Hz), 69.9. **^{31}P NMR** (162 MHz, $CDCl_3$) δ 32.3. **HRMS** calculated for $C_{30}H_{24}OPFe$ $[M+H]^+$ 487.0909, found 487.0911.

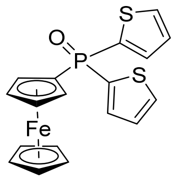

Bis(thiophen-2-yl)phosphinylferrocene (8i): Yellow solid, mp 176.8-178.4 ^{o}C, 30.3 mg, 38% yield. **^{1}H NMR** (700 MHz, $CDCl_3$) δ 7.68 (t, *J* = 4.5 Hz, 2H), 7.45 (dd, *J* = 7.8, 3.6 Hz, 2H), 7.17 – 7.12 (m, 2H), 4.50 – 4.46 (m, 2H), 4.46 – 4.43 (m, 2H), 4.28 (s, 5H); **^{13}C NMR** (100 MHz, $CDCl_3$) δ 135.98 (d, *J* = 121.8 Hz), 135.82 (d, *J* = 10.7 Hz), 133.26 (d, *J* = 5.3 Hz), 127.92 (d, *J* = 14.5 Hz), 74.22 (d, *J* = 131.0 Hz), 72.04 (d, *J* = 14.7 Hz), 71.78 (d, *J* = 11.6 Hz), 69.94; **^{31}P NMR** (162 MHz, $CDCl_3$) δ 15.8. **HRMS** calculated for $C_{18}H_{16}OPS_2Fe$ $[M+H]^+$ 398.9724, found 398.9732.

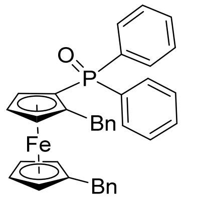

2,2'-Dibenenzyl-1-(diphenylphosphinyl)ferrocene (8j): Yellow gum, 19.3 mg, 17% yield. **^{1}H NMR** (400 MHz, $CDCl_3$) δ 7.80 (dd, *J* = 11.8, 7.3 Hz, 2H), 7.56 – 7.47 (m, 3H), 7.42 (dd, *J* = 12.1, 7.7 Hz, 2H), 7.35 (t, *J* = 7.5 Hz, 1H), 7.25 – 7.18 (m, 4H), 7.18 – 7.12 (m, 1H), 7.05 (d, *J* = 7.7 Hz, 2H), 7.01 – 6.94 (m, 4H), 4.36 (s, 1H), 4.33 (s, 1H), 4.28 – 4.20 (m, 3H), 4.15 (s, 1H), 4.03 (d, *J* = 15.0 Hz, 1H), 3.82 (d, *J* = 16.6 Hz, 2H), 3.47 (d, *J* = 15.1 Hz, 1H), 3.34 (d, *J* = 15.1 Hz, 1H). **^{13}C NMR** (100 MHz, $CDCl_3$) δ 141.4, 140.7, 134.8 (d, *J* = 105.7 Hz), 133.9 (d, *J* = 105.6 Hz), 131.7 (d, *J* = 9.7 Hz), 131.5 (d, *J* = 9.9 Hz), 131.5 (d, *J* = 2.6 Hz), 131.2 (d, *J* = 2.7 Hz), 128.9, 128.34, 128.31, 128.23, 128.16, 128.1, 128.0, 127.9, 126.0, 125.8, 92.7 (d, *J* = 10.9 Hz), 89.3, 74.4, 74.4, 74.3, 74.2, 71.91, 71.3 (d, *J* = 115.4 Hz), 70.9 (d, *J* = 11.3 Hz), 70.8, 70.4, 70.3, 35.2, 34.3. **^{31}P NMR** (162 MHz, $CDCl_3$) δ 29.0. **HRMS** calculated for $C_{36}H_{32}OPFe$ $[M+H]^+$ 567.1535, found 567.1510.

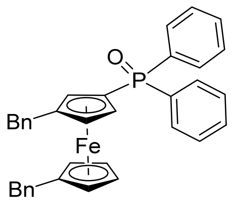

3,3'-Dibenenzyl-1-(diphenylphosphinyl)ferrocene (8j'): Yellow gum, 54.6 mg, 48% yield. **^{1}H NMR** (700 MHz, $CDCl_3$) δ 7.69 (td, *J* = 12.2, 7.3 Hz, 4H), 7.50 (q, *J* = 7.7 Hz, 4H), 7.45 (t, *J* = 9.0 Hz, 5H), 7.30 (t, *J* = 7.5 Hz, 2H), 7.23 (t, *J* = 7.7 Hz, 3H), 7.18 (d, *J* = 7.4 Hz, 2H), 7.15 (t, *J* = 7.4 Hz, 1H), 7.06 (d, *J* = 7.5 Hz, 2H), 4.34 (s, 1H), 4.29 (d, *J* = 7.8 Hz, 2H), 4.21 (s, 1H), 4.16 (s, 1H), 4.06 (s, 1H), 4.02 (s, 1H), 3.71 – 3.61 (m, 2H), 3.48 – 3.39 (m, 2H). **^{13}C NMR** (175 MHz, $CDCl_3$) δ 141.3, 140.9, 134.4 (d, *J* = 106.2 Hz), 131.6 (d, *J* = 2.8 Hz), 131.6 (d, *J* = 2.5 Hz), 128.4 (d, *J* = 11.0 Hz), 128.33 (d, *J* = 7.7 Hz), 128.27 (d, *J* = 3.9 Hz), 126.3, 126.0, 91.9 (d, *J* = 10.1 Hz), 89.8, 73.3, 73.2, 73.2, 72.6 (d, *J* = 12.4 Hz), 72.2 (d, *J* = 117.3 Hz), 71.2 (d, *J* = 2.5 Hz), 70.1, 69.8, 35.5, 35.2. **^{31}P NMR** (162 MHz, $CDCl_3$) δ 29.2; **HRMS** calculated for $C_{36}H_{31}OPNaFe$ $[M+Na]^+$ 589.1354, found 589.1381.

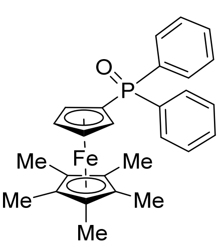

1-Diphenylphosphinyl-1’,2’,3’,4’,5’-pentamethyferrocene (8k): Yellow solid, mp 195.3-197.0 °C, 60.2 mg, 66% yield. **^{1}H NMR** (400 MHz, $CDCl_3$) δ 7.61 (dd, *J* = 11.9, 7.5 Hz, 4H), 7.46 – 7.33 (m, 6H), 3.99 (s, 4H), 1.82 (s, 15H); **^{13}C NMR** (100 MHz, $CDCl_3$) δ 135.6 (d, *J* = 104.6 Hz), 131.5 (d, *J* = 9.6 Hz), 131.1 (d, *J* = 2.7 Hz), 128.1 (d, *J* = 11.8 Hz), 82.0, 75.8 (d, *J* = 10.9 Hz), 74.2 (d, *J* = 12.9 Hz), 73.2 (d, *J* = 120.4 Hz); **^{31}P NMR** (162 MHz, $CDCl_3$) δ 28.0; **HRMS** calculated for $C_{27}H_{29}OPNaFe$ $[M+Na]^+$ 479.1198, found 479.1199.

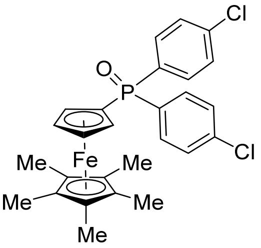

1-Bis(4-chlorophenyl) phosphinyl-1’,2’,3’,4’,5’-pentamethyferrocene (8l): Yellow solid, mp 132.2-133.6 °C, 52.7 mg, 50% yield. **^{1}H NMR** (400 MHz, $CDCl_3$) δ 7.51 (dd, *J* = 11.4, 8.1 Hz, 8H), 7.36 (dd, *J* = 8.4, 2.2 Hz, 8H), 4.02 (d, *J* = 2.0 Hz, 4H), 3.94 (d, *J* = 2.0 Hz, 4H), 1.82 (s, 15H); **^{13}C NMR** (100 MHz, $CDCl_3$) δ 137.8 (d, *J* = 3.5 Hz), 133.6 (d, *J* = 106.1 Hz), 132.7 (d, *J* = 10.6 Hz), 128.4 (d, *J* = 12.5 Hz), 82.1, 76.0 (d, *J* = 11.1 Hz), 73.9 (d, *J* = 13.3 Hz), 72.1 (d, *J* = 123.0 Hz), 11.3; **^{31}P NMR** (162 MHz, $CDCl_3$) δ 27.3; **HRMS** calculated for $C_{27}H_{27}OPCl_2NaFe$ $[M+Na]^+$ 547.0418, found 547.0432.

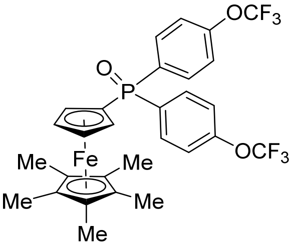

1-Bis(4-trifluoromethoxyphenyl)phosphinyl-1’,2’,3’,4’,5’-pentamethyferrocene (8m): Yellow solid, mp 136.9-138.4 °C, 90.0 mg, 72% yield. **^{1}H NMR** (400 MHz, $CDCl_3$) δ 7.70 – 7.62 (m, 4H), 7.25 (d, *J* = 8.2 Hz, 4H), 4.05 (q, *J* = 1.8 Hz, 2H), 3.98 (q, *J* = 1.9 Hz, 2H), 1.82 (s, 15H); **^{13}C NMR** (100 MHz, $CDCl_3$) δ 151.6 (dq, *J* = 3.2, 1.7 Hz), 133.7 (d, *J* = 106.0 Hz), 133.4 (d, *J* = 10.8 Hz), 120.4 (q, *J* = 258.5 Hz), 120.3 (d, *J* = 12.8 Hz), 82.3, 76.2 (d, *J* = 11.1 Hz), 74.0 (d, *J* = 13.3 Hz), 72.0 (d, *J* = 123.4 Hz), 11.3. **^{31}P NMR** (162 MHz, $CDCl_3$) δ 26.7; **^{19}F NMR** (376 MHz, $CDCl_3$) δ -57.7. **HRMS** calculated for $C_{29}H_{27}O_3PF_6NaFe$ $[M+Na]^+$ 647.0844, found 647.0842.

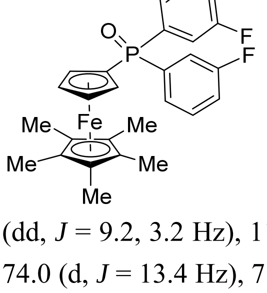

1-Bis(3-fluorophenyl)phosphinyl-1’,2’,3’,4’,5’-pentamethylferrocene (8n): Yellow solid, mp 179.9-181.4 °C, 61.1 mg, 62% yield **^{1}H NMR** (400 MHz, $CDCl_3$) δ 7.42 – 7.35 (m, 4H), 7.32 (ddd, *J* = 11.9, 8.8, 2.5 Hz, 2H), 7.15 (m, 2H), 4.04 (q, *J* = 1.9 Hz, 2H), 3.99 (q, *J* = 2.0 Hz, 2H), 1.83 (s, 15H); **^{13}C NMR** (100 MHz, $CDCl_3$) δ 162.4 (dd, *J* = 249.7, 16.6 Hz), 137.8 (dd, *J* = 103.9, 5.3 Hz), 130.2 (dd, *J* = 13.8, 7.3 Hz), 127.1 (dd, *J* = 9.2, 3.2 Hz), 118.5 (dd, *J* = 2.5 Hz), 118.3 (dd, *J* = 10.5 Hz), 82.3, 76.2 (d, *J* = 11.2 Hz), 74.0 (d, *J* = 13.4 Hz), 71.7 (d, *J* = 123.4 Hz), 11.4. **^{31}P NMR** (162 MHz, $CDCl_3$) δ 26.7; **^{19}F NMR** (376 MHz, $CDCl_3$) δ -111.7. **HRMS** calculated for $C_{27}H_{27}OPF_2NaFe$ $[M+Na]^+$ 515.1009, found 515.1010.

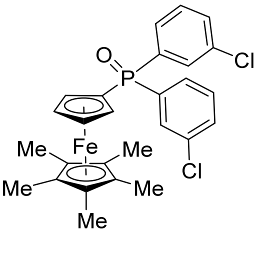

1-Bis(3-chlorophenyl)phosphinyl-1’,2’,3’,4’,5’-pentamethylferrocene (8o): Yellow solid, mp 124.5-125.7 °C, 78.1 mg, 74% yield. **^{1}H NMR** (400 MHz, $CDCl_3$) δ 7.61 (dt, *J* = 12.0, 1.7 Hz, 2H), 7.46 – 7.38 (m, 4H), 7.36 – 7.29 (m, 2H), 4.04 (q, *J* = 1.9 Hz, 2H), 3.96 (q, *J* = 1.9 Hz, 2H),

1.82 (s, 15H). **^{13}C NMR** (100 MHz, $CDCl_3$) δ 137.2 (d, *J* = 103.1 Hz), 134.7 (d, *J* = 15.4 Hz), 131.6 (d, *J* = 2.5 Hz), 131.3 (d, *J* = 10.6 Hz), 129.7 (d, *J* = 13.0 Hz), 129.5 (d, *J* = 9.1 Hz), 82.3, 76.3 (d, *J* = 11.4 Hz), 74.0 (d, *J* = 13.3 Hz), 71.5 (d, *J* = 123.5 Hz), 11.4. **^{31}P NMR** (162 MHz, $CDCl_3$) δ 27.1. **HRMS** calculated for $C_{27}H_{27}OPCl_2NaFe$ $[M+Na]^+$ 547.0418, found 547.0414.

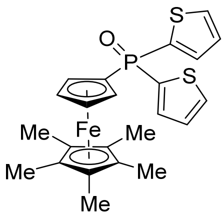

1-Bis(thiophen-2-yl)phosphinyl-1',2',3',4',5'-pentamethylferrocene (8p): Yellow solid, mp 140.5-142.1 °C, 52.0 mg, 55% yield. **^{1}H NMR** (400 MHz, $CDCl_3$) δ 7.62 (m, 2H), 7.44 m, 2H), 7.10 (m, 2H), 4.14 (q, *J* = 2.0 Hz, 2H), 4.00 (q, *J* = 1.9 Hz, 2H), 1.84 (s, 15H); **^{13}C NMR** (100 MHz, $CDCl_3$) δ 137.3 (d, *J* = 119.8 Hz), 135.4 (d, *J* = 10.2 Hz), 132.8 (d, *J* = 5.1 Hz), 127.7 (d, *J* = 14.2 Hz), 82.2, 76.0 (d, *J* = 11.9 Hz), 74.5 (d, *J* = 133.8 Hz), 73.7 (d, *J* = 14.8 Hz), 11.4; **^{31}P NMR** (162 MHz, $CDCl_3$) δ 16.0; **HRMS** calculated for $C_{23}H_{25}OS_2PNaFe$ $[M+Na]^+$ 491.0326, found 491.0331.

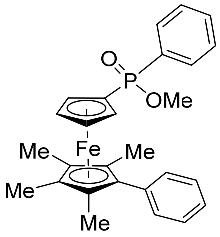

Methyl(1'-phenyl-2',3',4',5'-pentamethylferrocenyl)(phenyl) phosphinate (8q): Yellow solid, mp 64.8-66.6 °C, 33.7mg, 36% yield. **^{1}H NMR** (400 MHz, $CDCl_3$) δ 7.76 – 7.66 (m, 2H), 7.62 – 7.55 (m, 2H), 7.49 – 7.43 (m, 1H), 7.42 – 7.32 (m, 4H), 7.28 (d, *J* = 7.3 Hz, 1H), 4.21 (s, 1H), 4.13 (s, 1H), 4.06 – 4.02 (m, 1H), 4.00 (q, *J* = 3.0 Hz, 1H), 3.64 (d, *J* = 11.4 Hz, 3H), 2.05 (s, 3H), 2.04 (s, 3H), 1.99 (s, 3H), 1.97 (s, 3H); **^{13}C NMR** (100 MHz, $CDCl_3$) δ 137.1, 132.5 (d, *J* = 132.2 Hz), 131.6 (d, *J* = 2.8 Hz), 131.5 (d, *J* = 9.8 Hz), 131.2, 128.4 (d, *J* = 12.9 Hz), 127.6, 126.2, 88.1, 83.1, 83.0, 81.8, 81.7, 76.5 (d, *J* = 12.8 Hz), 76.4 (d, J = 11.8 Hz), 74.7 (d, *J* = 17.8 Hz), 74.3 (d, *J* = 12.0 Hz), 71.8 (d, *J* = 165.3 Hz), 50.9 (d, *J* = 5.9 Hz), 12.0, 11.9, 11.3; **^{31}P NMR** (162 MHz, $CDCl_3$) δ 38.6; **HRMS** calculated for $C_{27}H_{29}O_2PNaFe$ $[M+Na]^+$ 495.1147, found 495.1145.

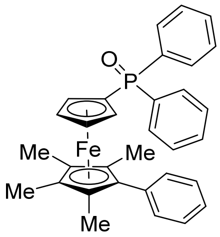

1-Diphenylphosphinyl-1'-phenyl-2',3',4',5'-tetramethylferrocene (8r): Yellow solid, mp 42.9-44.2 °C, 76.8 mg, 74% yield. **^{1}H NMR** (400 MHz, $CDCl_3$) δ 7.67 – 7.59 (m, 4H), 7.47 – 7.34 (m, 8H), 7.29 – 7.21 (m, 3H), 4.12 (q, *J* = 2.0 Hz, 2H), 4.08 (q, *J* = 1.9 Hz, 2H), 1.94 (s, 6H), 1.91 (s, 6H). **^{13}C NMR** (100 MHz, $CDCl_3$) δ 136.9, 135.3 (d, *J* = 104.9 Hz), 131.5 (d, *J* = 9.7 Hz), 131.2 (d, *J* = 2.9 Hz), 131.2, 128.1 (d, *J* = 11.9 Hz), 127.7, 126.2, 88.0, 83.0, 82.0, 76.7 (d, *J* = 10.7 Hz), 75.2 (d, *J* = 12.8 Hz), 73.4 (d, *J* = 119.8 Hz), 12.3, 11.5; **^{31}P NMR** (162 MHz, $CDCl_3$) δ 28.0; **HRMS** calculated for $C_{32}H_{32}OPFe$ $[M+H]^+$ 519.1535, found 519.1527.

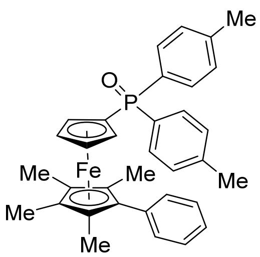

1-Bis(4-methylphenyl) phosphinyl-1'-phenyl-2',3',4',5'-tetramethyl ferrocene (8s): Yellow solid, mp 105.6-107.3 °C, 61.2 mg, 56% yield. **^{1}H NMR** (400 MHz, $CDCl_3$) δ 7.50 (dd, *J* = 11.7, 7.8 Hz, 4H), 7.41 – 7.36 (m, 2H), 7.25 – 7.21 (m, 3H), 7.17 (dd, *J* = 8.1, 2.6 Hz, 4H), 4.10 (q, *J* = 2.0 Hz, 2H), 4.06 (q, *J* = 1.8 Hz, 2H), 2.35 (s, 6H), 1.94 (s, 6H), 1.93 (s, 6H); **^{13}C NMR** (100 MHz, $CDCl_3$) δ 141.4 (d, *J* = 2.8 Hz), 137.0, 132.3 (d, *J* = 107.3 Hz), 131.5 (d, *J* = 10.1 Hz), 131.2, 128.8 (d, *J* = 12.3 Hz), 127.6, 126.1, 87.9, 83.0, 81.9, 76.5 (d, *J* = 10.7 Hz), 75.2 (d, *J* = 12.8 Hz), 74.0 (d, *J* = 119.5

Hz), 21.6, 12.3, 11.6; **^{31}P NMR** (162 MHz, $CDCl_3$) δ 28.1; **HRMS** calculated for $C_{34}H_{35}OPNaFe$ $[M+Na]^+$ 569.1667, found 569.1693.

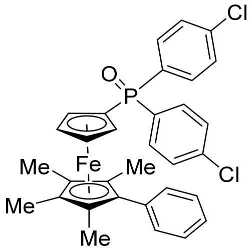

1-Bis(4-chlorophenyl) phosphinyl-1'-phenyl-2',3',4',5'-tetramethyl ferrocene (8t): Yellow solid, mp 128.7-129.3 °C, 71.3 mg, 61% yield. **^{1}H NMR** (400 MHz, $CDCl_3$) δ 7.50 (dd, *J* = 11.4, 8.1 Hz, 4H), 7.38 – 7.31 (m, 6H), 7.28 – 7.24 (m, 3H), 4.11 (t, *J* = 2.0 Hz, 2H), 4.07 (t, *J* = 2.0 Hz, 2H), 1.93 (s, 12H). **^{13}C NMR** (100 MHz, $CDCl_3$) δ 137.9 (d, *J* = 3.3 Hz), 136.5, 133.4 (d, *J* = 106.2 Hz), 132.7 (d, *J* = 10.6 Hz), 131.0, 128.5 (d, *J* = 12.5 Hz), 127.6, 126.3, 88.1, 83.1, 82.2, 76.9 (d, *J* = 11.0 Hz), 75.0 (d, *J* = 13.2 Hz), 72.4 (d, *J* = 122.3 Hz), 12.2, 11.5. **^{31}P NMR** (162 MHz, $CDCl_3$) δ 27.1. **HRMS** calculated for $C_{32}H_{29}OPCl_2NaFe$ $[M+Na]^+$ 609.0575, found 609.0578.

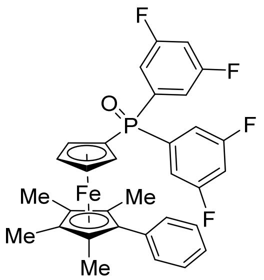

1-Bis(3,5-difluorophenyl)phosphinyl-1'-phenyl-2'3,4'5'-tetramethyl ferrocene (8u): Yellow solid, mp 121.8-122.2 °C, 69.7 mg, 59% yield. **^{1}H NMR** (400 MHz, $CDCl_3$) δ 7.44 – 7.38 (m, 2H), 7.31 – 7.24 (m, 3H), 7.17 – 7.06 (m, *J* = 5.0 Hz, 4H), 6.96 – 6.87 (m, 2H), 4.18-4.17 (m, 2H), 4.12-4.10 (m, 2H), 1.92 (s, 12H); **^{13}C NMR** (100 MHz, $CDCl_3$) δ 162.8 (ddd, *J* = 253.8, 19.6, 11.0 Hz), 138.6 (dt, *J* = 103.9, 6.5 Hz), 136.4, 131.0, 127.8, 126.5, 114.29 (ddd, J = 26.0, 10.2, 1.6 Hz) 107.4 (dt, J = 25.0, 1.7 Hz), 88.5, 83.4, 82.4, 75.0 (d, *J* = 13.4 Hz), 77.4 (d, J = 11.4 Hz), 70.7 (d, *J* = 125.5 Hz), 11.9 (d, *J* = 69.5 Hz); **^{31}P NMR** (162 MHz, $CDCl_3$) δ 26.2 (t, *J* = 6.4 Hz); **^{19}F NMR** (376 MHz, $CDCl_3$) δ -107.1 (q, *J* = 6.8 Hz). **HRMS** calculated for $C_{32}H_{28}OPF_4Fe$ $[M+H]^+$ 591.1158, found 591.1154.

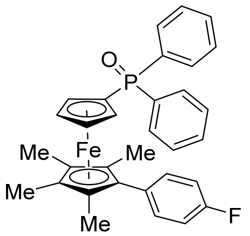

1-Diphenylphosphinyl-1'-(4-flurophenyl)-2',3',4',5'-tetramethylferrocene (8v): Yellow solid, mp 154.2-156.1 °C, 62.4 mg, 58% yield. **^{1}H NMR** (400 MHz, $CDCl_3$) δ 7.67 – 7.57 (m, 4H), 7.49 – 7.41 (m, 2H), 7.41 – 7.33 (m, 6H), 6.97 – 6.90 (m, 2H), 4.10 (q, *J* = 2.0 Hz, 2H), 4.07 (q, *J* = 1.8 Hz, 2H), 1.91 (s, 6H), 1.88 (s, 6H); **^{13}C NMR** (100 MHz, $CDCl_3$) δ 161.4 (d, *J* = 245.1 Hz), 135.2 (d, *J* = 105.0 Hz), 132.6, 132.6 (d, *J* = 7.8 Hz), 131.5 (d, *J* = 9.6 Hz), 131.3 (d, *J* = 2.8 Hz), 128.2 (d, *J* = 12.0 Hz), 114.6 (d, *J* = 21.1 Hz), 87.2, 82.8, 82.1, 76.7 (d, *J* = 10.7 Hz), 75.2 (d, *J* = 12.7 Hz), 73.4 (d, *J* = 119.8 Hz), 12.2, 11.5. **^{31}P NMR** (162 MHz, $CDCl_3$) δ 28.1; **^{19}F NMR** (376 MHz, $CDCl_3$) δ -116.5. **HRMS** calculated for $C_{32}H_{31}OPFFe$ $[M+H]^+$ 537.1440, found 537.1445.

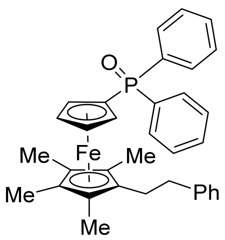

1-Diphenylphosphinyl-1'phenethyl-2',3',4',5'-tetramethylferrocene (8w): Yellow solid, mp 50.8-51.9 °C, 71.4 mg, 65% yield. **^{1}H NMR** (700 MHz, $CDCl_3$) δ 7.64 – 7.53 (m, 3H), 7.42 – 7.36 (m, 2H), 7.36 – 7.30 (m, 3H), 7.28 – 7.22 (m, 2H), 7.20 – 7.12 (m, 1H), 7.08 (d, *J* = 7.5 Hz, 2H), 4.00 (s, 2H), 3.99 (s, 2H), 2.56 (t, *J* = 8.1 Hz, 2H), 2.47 (t, *J* = 8.2 Hz, 2H), 1.83 (s, 6H), 1.77 (s, 6H). **^{13}C NMR** (175 MHz, $CDCl_3$) δ 142.1, 135.3 (d, *J* = 104.7 Hz), 131.4 (d, *J* = 9.6 Hz), 131.2 (d, *J* = 2.6 Hz), 128.6, 128.3, 128.1 (d, *J* = 12.0 Hz), 125.8, 85.3, 82.5, 81.9, 75.6 (d, *J* = 10.8 Hz), 74.0 (d, *J* = 12.8 Hz), 73.0 (d, *J* = 120.8 Hz),

37.4, 29.4, 11.4, 11.2. **^{31}P NMR** (162 MHz, $CDCl_3$) δ 28.3; **HRMS** calculated for $C_{34}H_{35}OPNaFe$ $[M+Na]^+$ 569.1667, found 569.1666.

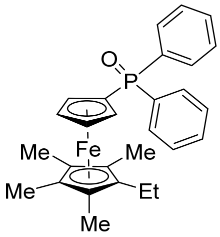

1-Diphenylphosphinyl-1'-ethyl-2',3',4',5'-tetramethylferrocene (8x): Yellow solid, mp 125.6-127.2 °C, 69.5 mg, 74% yield. **^{1}H NMR** (400 MHz, $CDCl_3$) δ 7.65 – 7.57 (m, 4H), 7.45 – 7.39 (m, 2H), 7.39 – 7.33 (m, 4H), 4.00 (s, 2H), 3.99 (s, 2H), 2.35 (q, *J* = 7.6 Hz, 2H), 1.83 (s, 6H), 1.83 (s, 6H), 0.87 (t, *J* = 7.6 Hz, 3H); **^{13}C NMR** (100 MHz, $CDCl_3$) δ 135.5 (d, *J* = 104.7 Hz), 131.5 (d, *J* = 9.9 Hz), 131.1 (d, *J* = 2.8 Hz), 128.0 (d, *J* = 11.9 Hz), 87.7, 82.3, 81.5, 75.6 (d, *J* = 10.9 Hz), 74.0 (d, *J* = 13.0 Hz), 73.1 (d, *J* = 120.7 Hz), 19.9, 15.3, 11.3, 11.1; **^{31}P NMR** (162 MHz, $CDCl_3$) δ 28.1; **HRMS** calculated for $C_{28}H_{31}OPNaFe$ $[M+Na]^+$ 493.1354, found 493.1359.

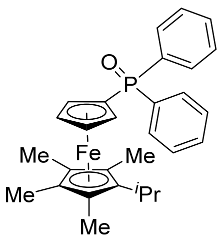

1-Diphenylphosphinyl-1'-isopropyl-2',3',4',5'-tetramethylferrocene (8y): Yellow solid, mp 85.5-87.0 °C, 55.2 mg, 57% yield. **^{1}H NMR** (400 MHz, $CDCl_3$) δ 7.64 – 7.55 (m, 4H), 7.46 – 7.40 (m, 2H), 7.40 – 7.33 (m, 4H), 4.11 (d, 2H), 4.08 (d, *J* = 2.2 Hz, 2H), 2.65 (hept, *J* = 7.1 Hz, 1H), 1.85 (s, 6H), 1.79 (s, 6H), 1.16 (d, *J* = 7.1 Hz, 6H); **^{13}C NMR** (100 MHz, $CDCl_3$) δ 135.3 (d, *J* = 104.8 Hz), 131.4 (d, *J* = 9.5 Hz), 131.2 (d, *J* = 2.9 Hz), 128.1 (d, *J* = 12.1 Hz), 92.0, 82.5, 81.0, 75.4 (d, *J* = 10.9 Hz), 74.0 (d, *J* = 12.9 Hz), 72.7 (d, *J* = 121.3 Hz), 26.8, 23.3, 12.0, 11.3; **^{31}P NMR** (162 MHz, $CDCl_3$) δ 28.9; **HRMS** calculated for $C_{29}H_{33}OPNaFe$ $[M+Na]^+$ 507.1511, found 507.1507.

Unsuccessful substrates for electrochemical C-H phosphorylation

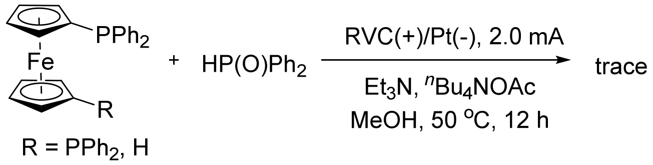

To an oven-dried undivided three-necked flask equipped with a stir bar, tertiary phosphines Fc-PPh_2 or Fc-2PPh_2 (0.20 mmol), diphenyl phosphine oxide (0.40 mmol), ^{n}Bu_4NOAc (0.20 mmol), MeOH (4 mL) and Et_3N (0.40 mmol) were combined and added in a glove-box. The flask was equipped with RVC (15 mm×10 mm×5 mm) as the anode and platinum plate (10 mm×10 mm×0.3 mm) as the cathode and. The reaction mixture was stirred and electrolyzed at a constant current of 2.0 mA under 50 °C for 12 h. However, only trace amount of desired products could be observed.

3.7. Typical procedure for electrochemical C-H phosphorylation of ruthenocene (Method B)

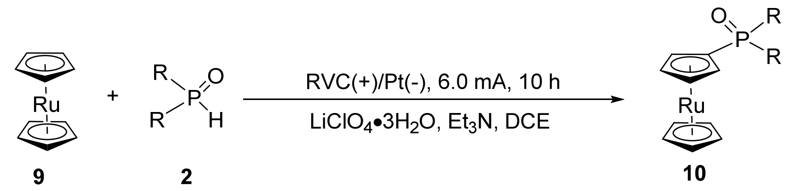

To an oven-dried undivided three-necked flask equipped with a stir bar, ruthenocene (0.20 mmol), diphenyl phosphine oxide (0.40 mmol), $LiClO_4•3H_2O$ (0.30 mmol), DCE (4 mL) and Et_3N (0.40

mmol) were combined and added in a glove-box. The flask was equipped with RVC (15 mm×10 mm×5 mm) as the anode and platinum plate (10 mm×10 mm×0.3 mm) as the cathode and. The reaction mixture was stirred and electrolyzed at a constant current of 6.0 mA under 50 °C for 10 h. When the reaction was finished, after filtered through a silica plug and concentrated in vacuo, the crude product mixture was purified by silica chromatography to afford the product.

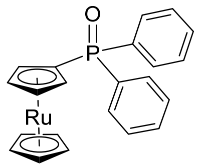

Diphenylphosphinylruthenocene (10a): Light yellow solid, mp 169.9-171.1 °C, 44.0 mg, 51% yield. **^{1}H NMR** (400 MHz, $CDCl_3$) δ 7.72 – 7.65 (m, 4H), 7.52 – 7.46 (m, 2H), 7.45 – 7.39 (m, 4H), 4.77 (q, *J* = 1.7 Hz, 2H), 4.69 (q, *J* = 1.7 Hz, 2H), 4.52 (s, 5H). **^{13}C NMR** (100 MHz, $CDCl_3$) δ 134.3 (d, *J* = 107.0 Hz), 131.6 (d, *J* = 2.9 Hz), 131.6 (d, *J* = 9.9 Hz), 128.2 (d, *J* = 12.1 Hz), 77.1 (d, *J* = 115.0 Hz), 74.0 (d, *J* = 13.4 Hz), 73.4 (d, *J* = 9.9 Hz), 72.4. **^{31}P NMR** (162 MHz, $CDCl_3$) δ 28.0; **HRMS** calculated for $C_{22}H_{20}OPRu$ $[M+H]^+$ 433.0290, found 433.0926.

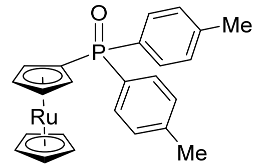

Bis(4-methyl)phenyl)phosphinylruthenocene (10b): Light yellow solid, mp 174.9-175.6 °C, 44.1 mg, 48% yield. **^{1}H NMR** (400 MHz, $CDCl_3$) δ 7.55 (dd, *J* = 12.0, 7.9 Hz, 4H), 7.21 (dd, *J* = 8.1, 2.6 Hz, 4H), 4.74 (q, *J* = 1.6 Hz, 2H), 4.66 (q, *J* = 1.8 Hz, 2H), 4.52 (s, 5H), 2.37 (s, 6H); **^{13}C NMR** (100 MHz, $CDCl_3$) δ 141.9 (d, *J* = 2.6 Hz), 131.6 (d, *J* = 10.2 Hz), 131.1 (d, *J* = 109.9 Hz), 128.9 (d, *J* = 12.5 Hz), 77.7 (d, *J* = 114.9 Hz), 74.0 (d, *J* = 13.4 Hz), 73.2 (d, *J* = 9.9 Hz), 72.3, 21.7. **^{31}P NMR** (162 MHz, $CDCl_3$) δ 28.1; **HRMS** calculated for $C_{24}H_{24}OPRu$ $[M+H]^+$ 461.0603, found 461.0605.

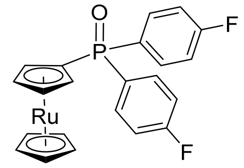

Bis(4-fluoro)phenyl)phosphinylruthenocene (10c): Light yellow solid, 53.3 mg, 57% yield. **^{1}H NMR** (400 MHz, $CDCl_3$) δ 7.66 (ddd, *J* = 11.6, 8.3, 5.5 Hz, 4H), 7.12 (td, *J* = 8.7, 2.1 Hz, 4H), 4.79 (q, *J* = 1.6 Hz, 2H), 4.64 (q, *J* = 1.8 Hz, 2H), 4.55 (s, 5H); **^{13}C NMR** (100 MHz, $CDCl_3$) δ 165.0 (dd, *J* = 253.1, 3.3 Hz), 134.0 (dd, *J* = 11.5, 8.7 Hz), 130.1 (dd, *J* = 110.3, 3.3 Hz), 115.7 (dd, *J* = 21.3, 13.3 Hz), 76.8 (d, *J* = 117.0 Hz), 73.9 (d, *J* = 13.7 Hz), 73.6 (d, *J* = 10.1 Hz), 72.4. **^{31}P NMR** (162 MHz, $CDCl_3$) δ 26.7; **^{19}F NMR** (376 MHz, $CDCl_3$) δ -107.0. **HRMS** calculated for $C_{22}H_{17}OPF_2NaRu$ $[M+Na]^+$ 490.9921, found 490.9921.

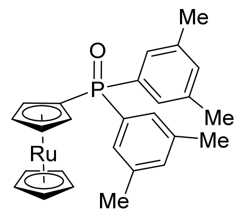

Bis(3,5-dimethyl)phenyl)phosphinylruthenocene (10d): Light yellow solid, mp 213.8-215.3 °C, 39.5 mg, 40% yield. **^{1}H NMR** (400 MHz, $CDCl_3$) δ 7.29 (d, *J* = 12.5 Hz, 4H), 7.11 (s, 2H), 4.76 (q, *J* = 1.6 Hz, 2H), 4.69 (q, *J* = 1.7 Hz, 2H), 4.5 (s, 5H), 2.3 (s, 12H); **^{13}C NMR** (100 MHz, $CDCl_3$) δ 137.7 (d, *J* = 12.8 Hz), 134.0 (d, *J* = 106.3 Hz), 133.3 (d, *J* = 2.8 Hz), 129.2 (d, *J* = 10.0 Hz), 77.7 (d, *J* = 108.7 Hz), 74.1 (d, *J* = 13.3 Hz), 73.2 (d, *J* = 9.8 Hz), 72.3, 21.4. **^{31}P NMR** (162 MHz, $CDCl_3$) δ 28.4; **HRMS** calculated for $C_{26}H_{28}OPRu$ $[M+H]^+$ 489.0916, found 489.0918.

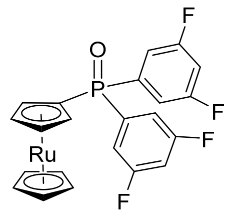

Bis(3,5-difluoro)phenyl)phosphinylruthenocene (10e): Light yellow solid, 36.2 mg, 36% yield. **^{1}H NMR** (400 MHz, $CDCl_3$) δ 7.25 – 7.18 (m, 4H), 7.01 – 6.94 (m, 2H), 4.85 (q, J = 1.7 Hz, 2H), 4.69 (q, J = 1.7 Hz, 2H), 4.60 (s, 5H); **^{13}C NMR** (100 MHz, $CDCl_3$) δ 162.9 (ddd, J = 253.9, 19.9, 11.1 Hz), 137.5 (dt, J = 106.1, 6.8 Hz), 114.7, 114.6, 114.6, 114.5, 114.4, 114.4, 114.3, 107.8 (dt, J =24.9, 2.0 Hz), 74.11 (d, J = 10.6 Hz), 74.09 (d, J = 120.3 Hz), 73.8 (d, J = 14.2 Hz), 72.8. **^{31}P NMR** (162 MHz, $CDCl_3$) δ 25.5 (t, J = 6.5 Hz). **^{19}F NMR** (376 MHz, $CDCl_3$) δ -106.9 (d, J = 6.6 Hz); **HRMS** calculated for $C_{22}H_{15}OPF_4NaRu$ $[M+Na]^+$ 526.9732, found 526.9708.

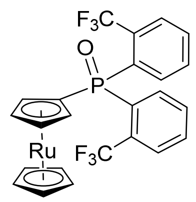

Bis(2-trifluoromethylphenyl)phosphinylruthenocene (10f): Light yellow solid, mp 180.5-182.3 °C, 60.1 mg, 53% yield. **^{1}H NMR** (400 MHz, $CDCl_3$) δ 8.21 (dd, J = 14.7, 7.4 Hz, 2H), 7.75 – 7.69 (m, 2H), 7.69 – 7.59 (m, 4H), 4.80 (q, J = 1.6 Hz, 2H), 4.67 (q, J = 1.8 Hz, 2H), 4.57 (s, 5H). **^{13}C NMR** δ 136.0 (d, J = 8.5 Hz), 132.02, 132.00, 131.9, 131.7 (d, J = 2.6 Hz), 131.7, 131.6, 131.4, 131.3, 131.1, 131.0, 130.8 (d, J = 11.6 Hz), 123.5 (qd, J = 274.6, 2.9 Hz), 78.9 (d, J = 124.0 Hz), 74.8 (d, J = 14.0 Hz), 73.3 (d, J = 10.6 Hz), 72.8. **^{31}P NMR** (162 MHz, $CDCl_3$) δ 29.8; **^{19}F NMR** (376 MHz, $CDCl_3$) δ -56.2. **HRMS** calculated for $C_{24}H_{18}OPF_6Ru$ $[M+H]^+$ 569.0037, found 569.0036.

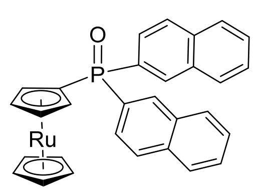

Bis(naphthalen-2-yl)phosphinylruthenocene (10g): Light yellow solid, mp 218.8-219.2 °C, 39.3 mg, 37% yield. **^{1}H NMR** (400 MHz, $CDCl_3$) δ 8.35 (d, J = 14.0 Hz, 2H), 7.93 – 7.84 (m, 6H), 7.70 (ddd, J = 10.0, 8.5, 1.5 Hz, 2H), 7.61 – 7.51 (m, 4H), 4.82 (q, J = 1.7 Hz, 2H), 4.79 (q, J = 1.8 Hz, 2H), 4.54 (s, 4H). **^{13}C NMR** (100 MHz, $CDCl_3$) δ 134.7 (d, J = 2.3 Hz), 133.3 (d, J = 9.5 Hz), 132.4 (d, J = 13.4 Hz), 131.4 (d, J = 107.4 Hz), 129.0, 128.1, 128.0 (d, J = 12.0 Hz), 126.9, 126.8, 77.1 (d, J = 115.4 Hz), 74.2 (d, J = 13.5 Hz), 73.4 (d, J = 10.0 Hz), 72.4; **^{31}P NMR** (162 MHz, $CDCl_3$) δ 28.5; **HRMS** calculated for $C_{30}H_{24}OPRu$ $[M+H]^+$ 533.0603, found 533.0603.

3.8. Derivatizations and scale-up synthesis

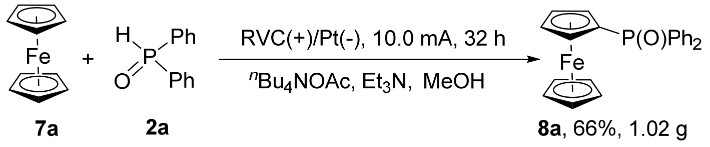

To an oven-dried undivided three-necked flask equipped with a stir bar, ferrocene (744.1 mg, 4.0 mmol), diphenyl phosphine oxide (1.62 g, 8.0 mmol), $^{n}Bu_4NOAc$ (1.21 g, 4.0 mmol), MeOH (80 mL) and Et_3N (809.0 mg, 8.0 mmol) were combined and added in a glove-box. The flask was equipped with RVC (100 PPI, 40 mm×15 mm×15 mm) as the anode and platinum plate (15 mm×15 mm×0.5 mm) as the cathode. The reaction mixture was stirred and electrolyzed at a constant current of 10.0 mA under 50 °C for 32 h. When the reaction was finished, concentrated in vacuo, the crude product mixture was purified by silica chromatography (petroleum ether/ethyl

acetate = 1:2) to afford the product, 1.02 g, 66% yield.

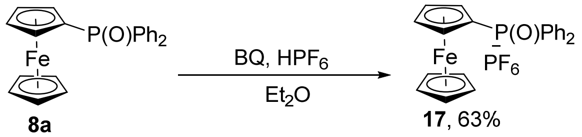

Diphenylphosphinylferrocenium hexaflurophosphate (17): known compound,[9] Benzoquinone (108.1 mg, 1.0 mmol) was added to **8a** (772.4 mg, 2.0 mmol) in diethyl ether (40 mL) at room temperature. The reaction mixture was stirred for 10 min. Then HPF_6 (973.1 mg, 60 wt% solution in H_2O, 4.0 mmol) was added at 0 °C. A greenish blue solid was formed, which was filtered through a sintered glass funnel and washed with ice cold ether until the ether solution became colorless to give product as a greenwish solid, known compound. mp 69.1-69.7 °C, 686.9 mg, 63%.

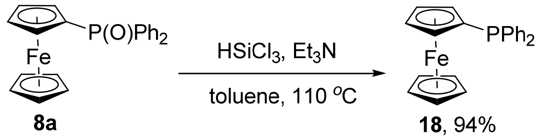

Diphenylphosphinoferrocene (18): To a sealed tube was sequentially added **8a** (77.2 mg, 0.2 mmol), toluene (1.0 mL), Et_3N (111.3 mg, 1.1 mmol), $HSiCl_3$ (108.6 mg, 1.0 mmol). The resulting mixture was stirred at 100 °C for 24 h. This solution was warmed to room temperature and the $NaHCO_3$ was added to the mixture. The mixture was diluted with diethyl ether (10 mL) and washed with water (10 mL×3), and the organic layer was dried over Na_2SO_4. The products were concentrated under reduced pressure and the residue was purified by flash column chromatography (silica gel, petroleum ether: ethyl acetate = 50:1) to afford product as a yellow solid, known compound. mp 111.1-113.0 °C, 69.8 mg, 94% yield. **^{1}H NMR** (400 MHz, $CDCl_3$) δ 7.44 – 7.37 (m, 4H), 7.36 – 7.31 (m, 6H), 4.39 (t, *J* = 1.8 Hz, 2H), 4.13 (q, *J* = 1.9 Hz, 2H), 4.10 (s, 5H). **^{13}C NMR** (175 MHz, $CDCl_3$) δ 139.1 (d, *J* = 9.4 Hz), 133.5 (d, *J* = 19.4 Hz), 128.5, 128.2 (d, *J* = 6.8 Hz), 75.8 (d, *J* = 5.6 Hz), 72.9 (d, *J* = 14.9 Hz), 70.8 (d, *J* = 3.9 Hz), 69.2; **^{31}P NMR** (162 MHz, $CDCl_3$) δ -16.4.

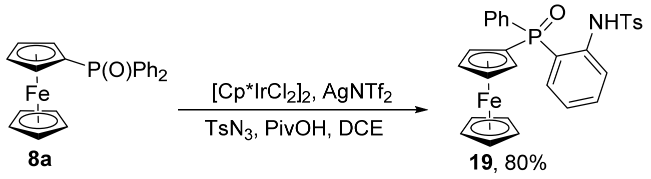

4-Methyl-*N*-(2-(phenyl(ferrocenyl)phosphoryl)phenyl)benzenesulfonamide (19): To a sealed tube was sequentially added $[Cp^*IrCl_2]_2$ (1.6 mg, 0.002 mmol), $AgNTf_2$ (3.2 mg, 0.008 mmol), PivOH (2.5 mg, 1.1 mmol), DCE (1.5 mL) and stirred for 15 min, then **8a** (38.6 mg, 0.1 mmol) and TsN_3 (75%wt in EA, 58 μL, 0.2 mmol) were added. The resulting mixture was stirred at 100 °C for 12 h and concentrated under reduced pressure. The residue was purified by flash column chromatography (silica gel, petroleum ether: ethyl acetate = 1:1) to afford product as a yellow solid, mp 214.3-215.7 °C, 44.2 mg, 80% yield. **^{1}H NMR** (400 MHz, $CDCl_3$) δ 11.30 (s, 1H), 7.79 (dd, *J* = 8.4, 4.4 Hz, 1H), 7.63 (d, *J* = 8.0 Hz, 2H), 7.55 – 7.47 (m, 1H), 7.46 – 7.32 (m, 5H), 7.01

(d, J = 8.1 Hz, 2H), 6.98 – 6.92 (m, 2H), 4.51 (s, 1H), 4.48 (s, 1H), 4.37 (s, 1H), 4.25 (s, 5H), 4.20 (s, 1H), 2.29 (s, 3H). **^{13}C NMR** (100 MHz, $CDCl_3$) δ 143.2, 143.0 (d, J = 3.3 Hz), 136.7, 133.2 (d, J = 107.1 Hz), 133.0 (d, J = 2.2 Hz), 132.81 (d, J = 10.8 Hz), 131.84 (d, J = 2.9 Hz), 131.1 (d, J = 10.3 Hz), 129.4, 128.3 (d, J = 12.4 Hz), 127.3, 122.7 (d, J = 12.3 Hz), 120.0 (d, J = 7.4 Hz), 119.0 (d, J = 102.4 Hz), 73.1 (d, J = 13.8 Hz), 72.6 (d, J = 10.8 Hz), 72.1 (d, J = 120.9 Hz), 71.8 (d, J = 11.0 Hz), 71.4 (d, J = 12.9 Hz), 69.9. **^{31}P NMR** (162 MHz, $CDCl_3$) δ 37.9. **HRMS** calculated for $C_{29}H_{26}O_3NPSNaFe$ [M+Na]$^+$ 578.0613, found 578.0598.

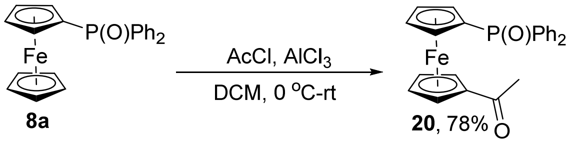

1-Acetyl-1'-diphenylphosphinylferrocene (20): To a solution of **8a** (193.1mg, 0.5 mmol) and $AlCl_3$ (133.3 mg, 1.0 mmol) in anhydrous DCM (2 mL) at 0 °C, was added a solution of AcCl (43μL, 0.60 mmol) in anhydrous DCM (2 mL). And then the resulting mixture was stirred at rt for 3 h. The mixture was washed with water (10 mL×3), and the organic layer was dried over Na_2SO_4 and concentrated under reduced pressure. The residue was purified by flash column chromatography (silica gel, petroleum ether: ethyl acetate = 1:2) to afford product as a yellow gum, 167.3 mg, 78% yield. **^{1}H NMR** (700 MHz, $CDCl_3$) δ 7.68 – 7.62 (m, 4H), 7.54 – 7.48 (m, 2H), 7.47 – 7.41 (m, 4H), 4.81 (m, 2H), 4.49 (m, 4H), 4.40 – 4.30 (m, 2H), 2.33 (s, 3H). **^{13}C NMR** (175 MHz, $CDCl_3$) δ 202.0, 133.7 (d, J = 106.8 Hz), 131.8 (d, J = 2.6 Hz), 131.3 (d, J = 9.9 Hz), 128.3 (d, J = 12.1 Hz), 80.3, 74.8 (d, J = 114.4 Hz), 73.8, 73.7 (d, J = 5.1 Hz), 73.6 (d, J = 7.5 Hz), 71.0, 27.6. **^{31}P NMR** (162 MHz, $CDCl_3$) δ 27.8; **HRMS** calculated for $C_{24}H_{22}O_2PFe$ [M+H]$^+$ 429.0701, found 429.0696.

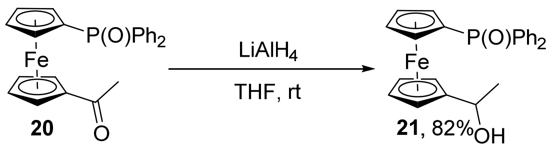

1-(1-Hydroxyethyl)-1'-diphenylphosphinylferrocene (21): To a solution of **20** (171.3mg, 0.4 mmol) in anhydrous THF (2 mL), was added $LiAlH_4$ (1 M in THF, 0.6 mL, 0.6 mmol). The resulting mixture was stirred at rt for 1 h. then cooled to 0 °C and quenched with NH_4Cl. This solution was warmed to room temperature. The mixture was diluted with DCM and washed with water, and the organic layer was dried over Na_2SO_4 and concentrated under reduced pressure. The residue was purified by flash column chromatography (silica gel, petroleum ether: ethyl acetate = 1:2) to afford product as a yellow solid, mp 133.7-134.5 °C, 141.1 mg, 82% yield. **^{1}H NMR** (400 MHz, $CDCl_3$) δ 7.73 – 7.57 (m, 4H), 7.49 – 7.33 (m, 6H), 5.93 (s, 1H), 4.71 – 4.60 (m, 1H), 4.52 (s, 1H), 4.45 (s, 1H), 4.41 (s, 1H), 4.32 (d, J = 5.9 Hz, 2H), 4.18 (s, 1H), 3.99 (s, 1H), 3.90 (s, 1H), 1.32 (d, J = 6.4 Hz, 3H). **^{13}C NMR** (100 MHz, $CDCl_3$) δ 133.6 (d, J = 107.4 Hz), 133.1 (d, J = 107.2 Hz). 131.8, 131.73, 131.69, 131.4 (d, J = 2.1 Hz), 131.3 (d, J = 2.1 Hz), 128.3 (d, J = 12.0 Hz), 98.1, 73.4 (d, J = 12.6 Hz). 72.6 (d, J = 117.0 Hz). 72.2 (d, J = 11.6 Hz), 71.8 (d, J = 10.6 Hz), 68.5, 67.9, 67.3 (d, J = 13.3 Hz), 64.8, 25.7. **^{31}P NMR** (162 MHz, $CDCl_3$) δ 30.9. **HRMS** calculated for $C_{24}H_{24}O_2PFe$ [M+H]$^+$ 431.0858, found 431.0878.

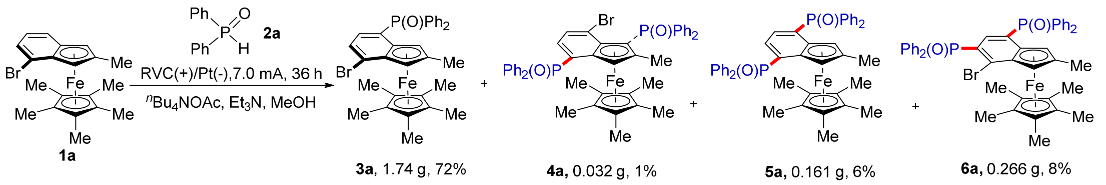

To an oven-dried undivided three-necked flask equipped with a stir bar, benzoferrocene (1.69 g, 4.0 mmol), diphenyl phosphine oxide (1.62 g, 8.0 mmol), $^{n}Bu_4NOAc$ (1.20 g, 4.0 mmol), MeOH (80 mL) and Et_3N (809.0 mg, 8.0 mmol) were combined and added in a glove-box. The flask was equipped with RVC (100 PPI, 40 mm×15 mm×15 mm) as the anode and platinum plate (15 mm×15 mm×0.5 mm) as the cathode. The reaction mixture was stirred and electrolyzed at a constant current of 7.0 mA under 50 °C for 36 h. When the reaction was finished, concentrated in vacuo, the crude product mixture was purified by silica chromatography (petroleum ether/ethyl acetate = 4:1-1:1) to respectively afford **3a** (1.74 g, 72%), **4a** (0.032 g, 1%), **5a** (0.161 g, 6%), **6a** (0.266 g, 8%).

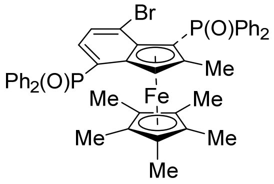

1-Methyl-2,7-diylbis(diphenylphosphinyloxide)4-diphenylphosphinyl-7-bromoindenyl-pentamethyl cyclopentadienyliron (4a): Purple solid, 0.032 g, 1%, **^{1}H NMR** (400 MHz, $CDCl_3$) δ 7.70 – 7.63 (m, 2H), 7.62 – 7.56 (m, 3H), 7.51 (dd, J = 7.7, 2.8 Hz, 3H), 7.45 – 7.31 (m, 8H), 7.25 – 7.20 (m, 2H), 7.18 – 7.11 (m, 2H), 7.09 (dd, J = 7.1, 2.3 Hz, 1H), 6.59 (dd, J = 15.0, 7.1 Hz, 1H), 5.47 (d, J = 2.1 Hz, 1H), 1.73 (s, 15H), 1.11 (s, 3H). **^{31}P NMR** (162 MHz, $CDCl_3$) δ 30.0, 27.1. **HRMS** calculated for $C_{44}H_{42}$O2P2BrFe $[M+H]^+$ 799.1187, found 799.1181.

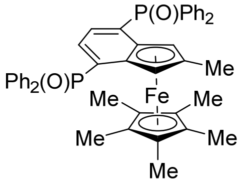

1-Methyl-4,7-diylbis(diphenylphosphineoxide)-pentamethyl cyclopentadienyliron (5a): Green solid, 0.161 g, 6%, **^{1}H NMR** (400 MHz, $CDCl_3$) δ 7.69 (dd, J = 11.7, 7.3 Hz, 4H), 7.57 – 7.42 (m, 12H), 7.43 – 7.35 (m, 4H), 6.73 – 6.62 (m, 2H), 4.80 (s, 2H), 1.78 (s, 3H), 1.71 (s, 15H). **^{31}P NMR** (162 MHz, $CDCl_3$) δ 29.5. **HRMS** calculated for $C_{44}H_{43}O_2P_2Fe$ $[M+H]^+$ 721.2082, found 721.2092.

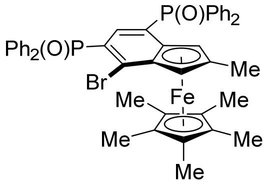

1-Methyl-3-bromo-4,6-diylbis(diphenylphosphinyloxide)-4-diphenyl-phosphinyl-7-bromoindenyl-pentamethyl cyclopentadienyliron (6a): Green solid, 0.266 g, 8%, ^{1}H NMR (400 MHz, $CDCl_3$) δ 7.69 (dd, J = 12.3, 7.5 Hz, 2H), 7.55 – 7.48 (m, 3H), 7.47 – 7.33 (m, 11H), 7.32 – 7.20 (m, 4H), 6.53 (dd, J = 10.6, 15.3 Hz, 1H), 5.04 (s, 1H), 4.47 (s, 1H), 1.90 (s, 3H), 1.67 (s, 15H). **^{31}P NMR** (162 MHz, $CDCl_3$) δ 29.6, 29.2. **HRMS** calculated for $C_{44}H_{42}O_2P_2BrFe$ $[M+H]^+$ 799.1187, found 799.1161.

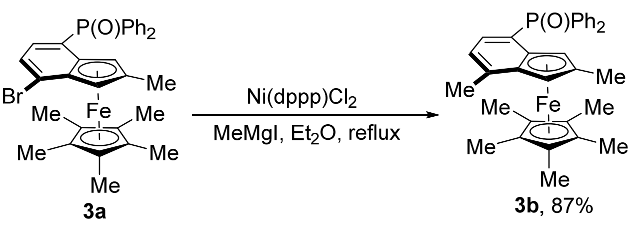

1,7-Dimethyl-4-diphenylphosphinylindenyl-pentamethylcyclopentadienyliron (3b): A solution of the MeMgI (3M in Et_2O, 0.33 mL, 1.0 mmol) was added to a stirred solution of **3a** (120.0 mg, 0.2 mmol) and $NiCl_2$(dppp) (27.1 mg, 0.05 mmol) in anhydrous Et_2O (10 mL). The mixture was refluxed for 3 h, then cooled to 0 °C and quenched with NH_4Cl. This solution was warmed to room temperature. The mixture was diluted with diethyl ether (10 mL) and washed with water (10 mL×3), and the organic layer was dried over Na_2SO_4. The products were concentrated under reduced pressure and the crude product mixture was purified by flash column chromatography (silica gel, petroleum ether/ethyl acetate = 4:1) to afford the product **3b** as a purple solid, 93.0 mg, 87% yield.

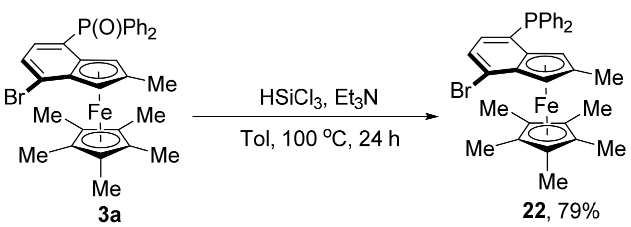

1-Methyl-4-diphenylphosphino-7-bromoindenyl-pentamethylcyclopentadienyliron (22): To a sealed tube was sequentially added **3a** (120.0 mg, 0.2 mmol), toluene (1.0 mL), Et_3N (111.3 mg, 1.1 mmol), $HSiCl_3$ (108.6 mg, 1.0 mmol). The resulting mixture was stirred at 100 ^{o}C for 24 h. The upper organic phase was dried over Na_2SO_4 and concentrated under reduced pressure. The residue was purified by flash column chromatography (silica gel, petroleum ether: ethyl acetate = 50:1) to afford product as a purple solid. mp 205.1-206.7 ^{o}C, 92.9 mg, 79% yield. **^{1}H NMR** (400 MHz, $CDCl_3$) δ 7.40 – 7.31 (m, 5H), 7.26 – 7.15 (m, 5H), 7.03 (d, *J* = 7.1 Hz, 1H), 6.23 – 6.12 (m, 1H), 4.29 (s, 1H), 4.19 (s, 1H), 1.84 (s, 3H), 1.68 (s, 15H). **^{13}C NMR** (100 MHz, $CDCl_3$) δ 136.5 (d, *J* = 14.7 Hz), 136.0 (d, *J* = 11.5 Hz), 135.9 (d, *J* = 10.5 Hz), 134.7 (d, *J* = 19.8 Hz), 133.9 (d, *J* = 19.9 Hz), 129.1, 128.7, 128.6, 128.6, 128.5, 128.4, 127.0, 124.6, 123.4, 92.1 (d, *J* = 24.6 Hz), 89.3 (d, *J* = 6.3 Hz), 88.7, 78.1, 67.6 (d, *J* = 10.6 Hz), 66.8, 13.6, 9.8 (d, *J* = 2.4 Hz). **^{31}P NMR** (162 MHz, $CDCl_3$) δ -12.9. **HRMS** calculated for $C_{32}H_{33}PBrFe$ $[M+H]^+$ 583.0847, found 583.0826.

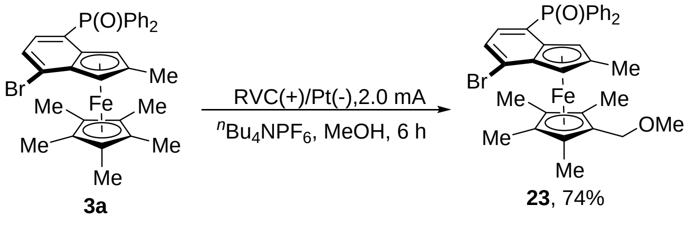

1-Methyl-4-diphenylphosphinyl-7-bromoindenyl-1'-methoxymethyl-2',3',4',5'tetramethyl cyclopentadienyliron (23): In an oven-dried undivided three-necked flask equipped with a stir

bar, **3a** (120.0 mg, 0.20 mmol), $^{n}Bu_4NPF_6$ (38.7 mg, 0.10 mmol), MeOH (5 mL) were combined and added in the air. The flask was equipped with RVC (10 mm×15 mm×5 mm) as the anode and platinum plate (10 mm×10 mm×0.3 mm) as the cathode and. The reaction mixture was stirred and electrolyzed at a constant current of 2.0 mA under 50 °C for 6 h. When the reaction was finished, concentrated in vacuo, the crude product mixture was purified by silica chromatography (petroleum ether/ethyl acetate = 2:1) to afford the purple product, viscous solid, 93.1 mg, 74% yield, **^{1}H NMR** (400 MHz, $CDCl_3$) δ 7.70 (dd, *J* = 11.8, 7.4 Hz, 2H), 7.60 – 7.53 (m, 1H), 7.53 – 7.40 (m, 5H), 7.39 – 7.29 (m, 2H), 7.10 (d, *J* = 7.1 Hz, 1H), 6.56 (dd, *J* = 15.4, 7.1 Hz, 1H), 4.90 (s, 1H), 4.34 (s, 1H), 4.33 – 4.20 (m, 2H), 3.28 (s, 3H), 1.84 (s, 3H), 1.72 (s, 3H), 1.70 (s, 6H), 1.64 (s, 3H). **^{13}C NMR** (100 MHz, $CDCl_3$) δ 132.9 (d, J = 104.46 Hz), 132.7, 132.5 (d, *J* = 9.6 Hz), 131.9 (d, *J* = 2.8 Hz), 131.8, 131.8, 131.7, 131.1 (d, *J* = 3.5 Hz), 130.7, 130.6, 128.5 (d, *J* = 12.2 Hz), 122.1 (d, *J* = 14.8 Hz), 91.7 (d, *J* = 9.8 Hz), 90.6, 89.2 (d, *J* = 8.9 Hz), 80.4, 79.5 (d, *J* = 16.5 Hz), 76.3, 68.6, 67.5, 66.4, 58.0, 13.7, 9.8, 9.6, 9.2, 9.0. **^{31}P NMR** (162 MHz, $CDCl_3$) δ 29.3; **HRMS** calculated for $C_{33}H_{34}O_2PBrNaFe$ $[M+Na]^+$ 651.0721, found 651.0728.

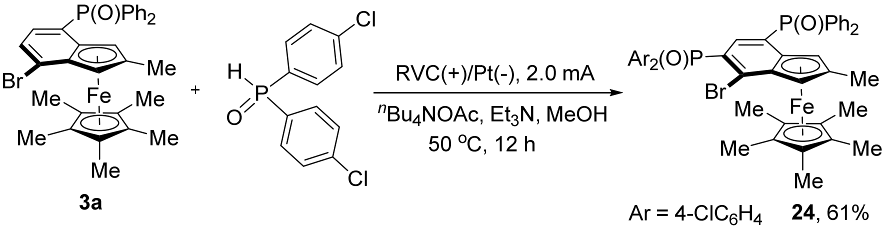

1-Methyl-4-diphenylphosphinyl-6-bis(4-chlorophenyl)phosphinyl-7-bromoindenyl-penta methyl cyclopentadienyliron (24): In an oven-dried undivided three-necked flask equipped with a stir bar, **3a** (120.0 mg, 0.20 mmol), diphenyl phosphine oxide (0.40 mmol), $^{n}Bu_4NOAc$ (60.2 mg, 0.20 mmol), MeOH (5 mL) and Et_3N (56 μL, 0.40 mmol) were combined and added in the glove-box. The flask was equipped with RVC (10 mm×15 mm×5 mm) as the anode and platinum plate (10 mm×10 mm×0.3 mm) as the cathode. The reaction mixture was stirred and electrolyzed at a constant current of 2.0 mA under 50 °C for 12 h. When the reaction was finished, concentrated in vacuo, the crude product mixture was purified by silica chromatography (petroleum ether/ethyl acetate = 1:2) to afford the green product. mp 195.6-197.1 °C, 105.9 mg, 61% yield. **^{1}H NMR** (700 MHz, $CDCl_3$) δ 7.64 – 7.58 (m, 2H), 7.54 (t, *J* = 7.5 Hz, 1H), 7.47 (t, *J* = 7.4 Hz, 1H), 7.44 – 7.35 (m, 10H), 7.31 – 7.27 (m, 2H), 7.25 (dd, *J* = 8.4, 2.2 Hz, 2H), 6.35 (dd, *J* = 15.6, 11.0 Hz, 1H), 5.02 (s, 1H), 4.47 (s, 1H), 1.91 (s, 3H), 1.70 (s, 15H). **^{13}C NMR** (175 MHz, $CDCl_3$) δ 142.3 (t, *J* = 3.5 Hz), 138.5 (d, *J* = 3.8 Hz), 138.4 (d, *J* = 3.2 Hz), 133.0 (d, *J* = 10.8 Hz), 132.9 (d, *J* = 10.2 Hz), 132.5 (d, *J* = 104.9 Hz), 132.2, 132.1, 132.0, 131.84, 131.83, 131.81, 131.76 (d, *J* = 9.5 Hz), 131.5 (d, *J* = 10.2 Hz), 131.2 (d, *J* = 104.4 Hz), 131.0 (d, *J* = 108.1 Hz), 130.9 (d, *J* = 108.6 Hz), 130.0 (dd, *J* = 103.6, 11.4 Hz), 129.1, 129.0, 128.9, 128.6 (d, *J* = 12.1 Hz), 128.4 (d, *J* = 12.1 Hz), 121.3 (dd, *J* = 110.6, 13.4 Hz), 93.6, 92.6 (d, *J* = 9.5 Hz), 89.9 (t, *J* = 8.9 Hz), 79.2, 70.4, 68.2, 13.6. 9.7. **^{31}P NMR** (162 MHz, $CDCl_3$) δ 29.4, 28.3. **HRMS** calculated for $C_{44}H_{40}O_2PCl_2BrFe$ $[M+H]^+$ 867.0408, found 867.0445.

3.9. X-ray Crystal Structures

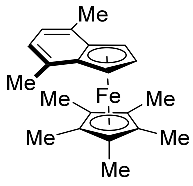

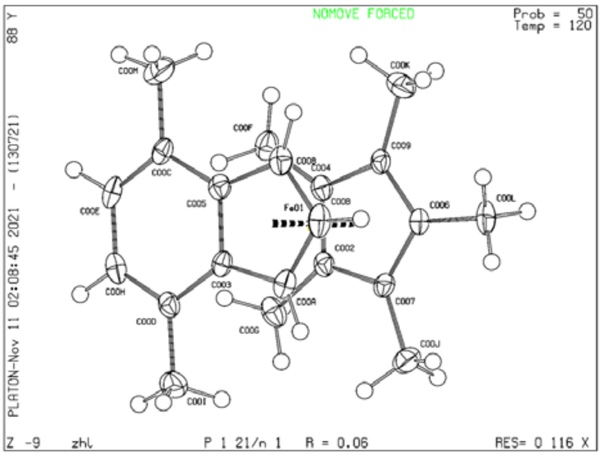

Supplementary Table 3. Crystal data and structure refinement for 1h

Identification code	**1h**
Empirical formula	$C_{21}H_{26}Fe$
Formula weight	334.27
Temperature/K	120.0
Crystal system	monoclinic
Space group	$P2_1/n$
a/Å	8.367(3)
b/Å	13.965(4)
c/Å	14.616(5)
α/°	90
β/°	95.649(12)
γ/°	90
Volume/$Å^3$	1699.5(9)
Z	4
$\rho_{calc}g/cm^3$	1.306
μ/mm^{-1}	0.882
F(000)	712.0
Crystal size/mm^3	0.25 × 0.25 × 0.25
Radiation	MoKα (λ = 0.71073)
2Θ range for data collection/°	5.392 to 55.336
Index ranges	$-10 \le h \le 10$, $-18 \le k \le 18$, $-18 \le l \le 19$
Reflections collected	21531
Independent reflections	3852 [R_{int} = 0.1022, R_{sigma} = 0.0665]
Data/restraints/parameters	3852/0/206
Goodness-of-fit on F^2	1.080
Final R indexes [I>=2σ (I)]	R_1 = 0.0552, wR_2 = 0.1470
Final R indexes [all data]	R_1 = 0.0611, wR_2 = 0.1515
Largest diff. peak/hole / e $Å^{-3}$	0.75/-0.74

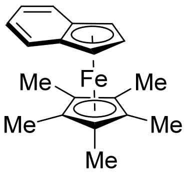

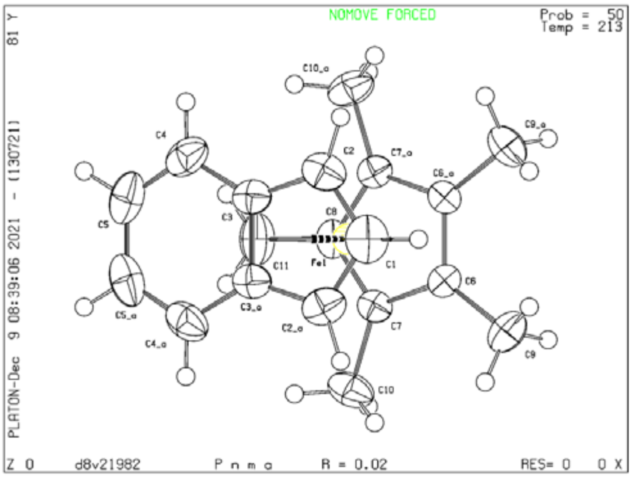

Supplementary Table 4. Crystal data and structure refinement for **1e.**

Identification code	**1e**
Empirical formula	$C_{19}H_{22}Fe$
Formula weight	306.21
Temperature/K	213.15
Crystal system	orthorhombic
Space group	Pnma
a/Å	9.2658(6)
b/Å	12.8517(8)
c/Å	12.8645(8)
α/°	90
β/°	90
γ/°	90
Volume/Å^3	1531.92(17)
Z	4
ρ_{calc}g/cm^3	1.328
μ/mm^{-1}	0.972
F(000)	648.0
Crystal size/mm^3	0.2 × 0.15 × 0.12
Radiation	MoKα (λ = 0.71073)
2Θ range for data collection/°	8.34 to 50.998
Index ranges	$-9 \le h \le 11$, $-13 \le k \le 15$, $-15 \le l \le 15$
Reflections collected	6901
Independent reflections	1482 [R_{int} = 0.0301, R_{sigma} = 0.0214]
Data/restraints/parameters	1482/2/107
Goodness-of-fit on F^2	1.061
Final R indexes [I>=2σ (I)]	R_1 = 0.0237, wR_2 = 0.0648
Final R indexes [all data]	R_1 = 0.0257, wR_2 = 0.0663
Largest diff. peak/hole / e Å^{-3}	0.24/-0.21

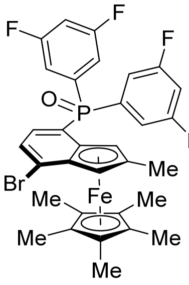

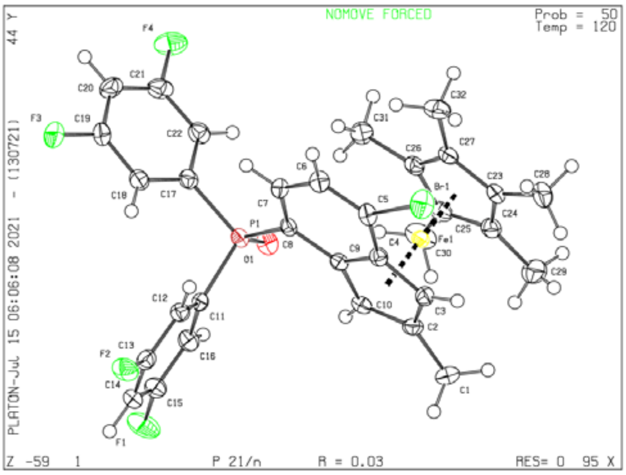

Supplementary Table 5. Crystal data and structure refinement for 3q

Identification code	**3q**	
Empirical formula	$C_{32}H_{28}BrF_4FeOP$	
Formula weight	671.27	
Temperature	120(2) K	
Wavelength	0.71073 Å	
Crystal system	Monoclinic	
Space group	$P2_1/n$	
Unit cell dimensions	a = 11.744(2) Å	a= 90°.
	b = 14.811(2) Å	b= 101.485(6)°.
	c = 16.375(3) Å	g = 90°.
Volume	2791.2(8) $Å^3$	
Z	4	
Density (calculated)	1.597 Mg/m^3	
Absorption coefficient	2.081 mm^{-1}	
F(000)	1360	
Crystal size	0.240 x 0.160 x 0.100 mm^3	
Theta range for data collection	2.241 to 27.542°.	
Index ranges	-15<=h<=15, -19<=k<=18, -19<=l<=21	
Reflections collected	38041	
Independent reflections	6414 [R(int) = 0.0853]	
Completeness to theta = 25.242°	99.9 %	
Absorption correction	Semi-empirical from equivalents	
Max. and min. transmission	0.7456 and 0.6198	
Refinement method	Full-matrix least-squares on F^2	
Data / restraints / parameters	6414 / 0 / 367	
Goodness-of-fit on F^2	1.038	
Final R indices [I>2sigma(I)]	R1 = 0.0321, wR2 = 0.0797	
R indices (all data)	R1 = 0.0451, wR2 = 0.0849	
Extinction coefficient	n/a	
Largest diff. peak and hole	0.435 and -0.596 e.$Å^{-3}$	

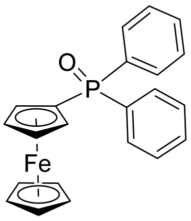

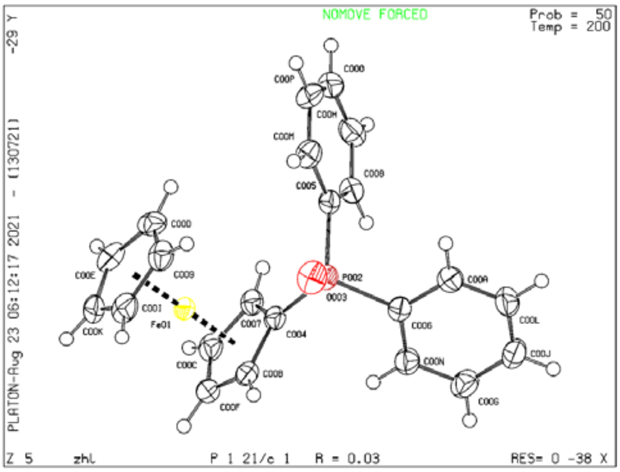

Supplementary Table 6. Crystal data and structure refinement for 8a

Identification code	**8a**
Empirical formula	$C_{22}H_{19}FeOP$
Formula weight	386.19
Temperature/K	200.0
Crystal system	monoclinic
Space group	$P2_1/c$
a/Å	14.1298(5)
b/Å	10.3853(4)
c/Å	11.7967(4)
α/°	90
β/°	90.5450(10)
γ/°	90
Volume/Å^3	1731.00(11)
Z	4
ρ_{calc}g/cm^3	1.482
μ/mm^{-1}	0.970
F(000)	800.0
Crystal size/mm^3	0.25 × 0.2 × 0.2
Radiation	MoKα (λ = 0.71073)
2Θ range for data collection/°	4.868 to 55.004
Index ranges	$-18 \le h \le 18$, $-13 \le k \le 13$, $-15 \le l \le 13$
Reflections collected	26332
Independent reflections	3967 [R_{int} = 0.0457, R_{sigma} = 0.0283]
Data/restraints/parameters	3967/0/226
Goodness-of-fit on F^2	2.112
Final R indexes [I>=2σ (I)]	R_1 = 0.0467, wR_2 = 0.1407
Final R indexes [all data]	R_1 = 0.0546, wR_2 = 0.1427
Largest diff. peak/hole / e Å^{-3}	0.75/-0.30

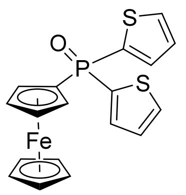

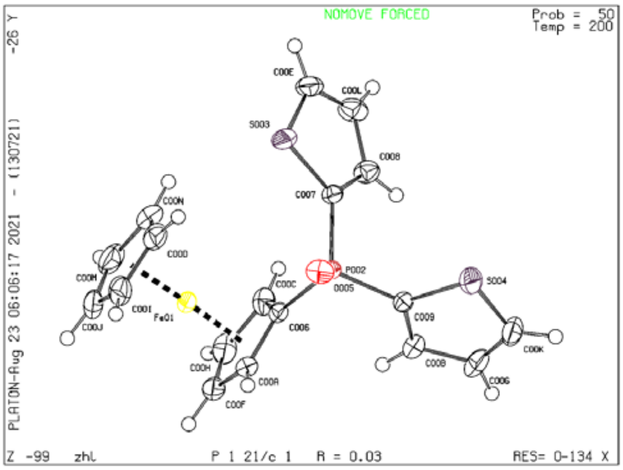

Supplementary Table 7. Crystal data and structure refinement for **8i**

Identification code	**8i**
Empirical formula	$C_{18}H_{15}FeOPS_2$
Formula weight	398.24
Temperature/K	200.0
Crystal system	monoclinic
Space group	$P2_1/c$
a/Å	8.1568(2)
b/Å	16.3835(5)
c/Å	13.0444(4)
α/°	90
β/°	104.9460(10)
γ/°	90
Volume/Å^3	1684.24(8)
Z	4
ρ_{calc}g/cm^3	1.571
μ/mm^{-1}	1.238
F(000)	816.0
Crystal size/mm^3	0.25 × 0.2 × 0.2
Radiation	MoKα (λ = 0.71073)
2Θ range for data collection/°	4.972 to 56.558
Index ranges	$-10 \leq h \leq 10$, $-20 \leq k \leq 21$, $-16 \leq l \leq 17$
Reflections collected	23148
Independent reflections	4151 [R_{int} = 0.0460, R_{sigma} = 0.0301]
Data/restraints/parameters	4151/0/208
Goodness-of-fit on F^2	1.851
Final R indexes [I>=2σ (I)]	R_1 = 0.0391, wR_2 = 0.1176
Final R indexes [all data]	R_1 = 0.0458, wR_2 = 0.1194
Largest diff. peak/hole / e Å^{-3}	0.83/-0.36

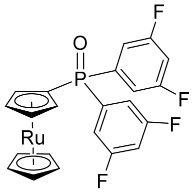

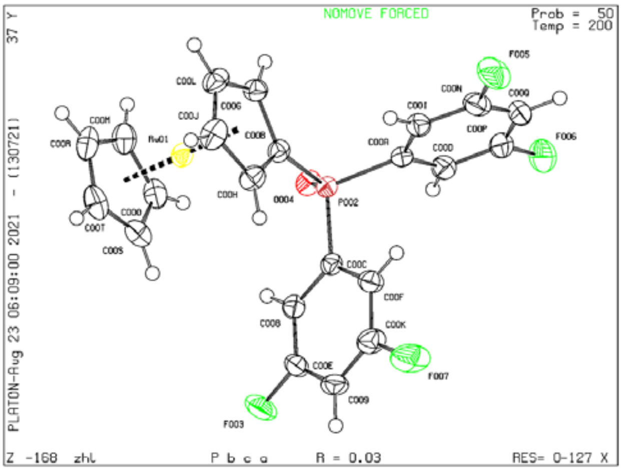

Supplementary Table 8. Crystal data and structure refinement for 10e

Identification code	**10e**
Empirical formula	$C_{22}H_{15}F_4OPRu$
Formula weight	503.38
Temperature/K	200.0
Crystal system	orthorhombic
Space group	Pbca
a/Å	15.5714(7)
b/Å	14.6187(6)
c/Å	16.7288(7)
α/°	90
β/°	90
γ/°	90
Volume/Å^3	3808.0(3)
Z	8
ρ_{calc}g/cm^3	1.756
μ/mm^{-1}	0.956
F(000)	2000.0
Crystal size/mm^3	0.25 × 0.2 × 0.2
Radiation	MoKα (λ = 0.71073)
2Θ range for data collection/°	4.532 to 56.666
Index ranges	$-20 \leq h \leq 20$, $-19 \leq k \leq 18$, $-22 \leq l \leq 22$
Reflections collected	44951
Independent reflections	4730 [R_{int} = 0.0582, R_{sigma} = 0.0395]
Data/restraints/parameters	4730/0/262
Goodness-of-fit on F^2	1.034
Final R indexes [I>=2σ (I)]	R_1 = 0.0291, wR_2 = 0.0627
Final R indexes [all data]	R_1 = 0.0456, wR_2 = 0.0685
Largest diff. peak/hole / e Å^{-3}	0.45/-0.41

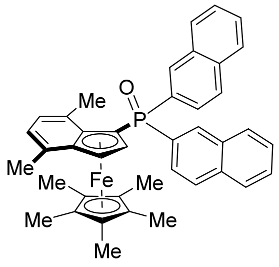

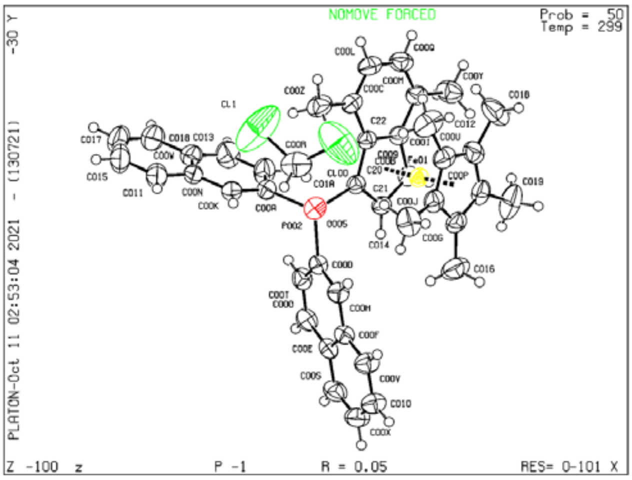

Supplementary Table 9. Crystal data and structure refinement for 3v'

Identification code	**3v'**
Empirical formula	$C_{42}H_{41}Cl_2FeOP$
Formula weight	719.47
Temperature/K	298.6
Crystal system	triclinic
Space group	P-1
a/Å	10.2954(3)
b/Å	13.3864(4)
c/Å	13.7575(4)
α/°	78.7740(10)
β/°	75.4810(10)
γ/°	84.6840(10)
Volume/Å^3	1798.45(9)
Z	2
ρ_{calc}g/cm^3	1.329
μ/mm^{-1}	0.645
F(000)	752.0
Crystal size/mm^3	0.25 × 0.2 × 0.2
Radiation	MoKα (λ = 0.71073)
2Θ range for data collection/°	4.51 to 55.012
Index ranges	$-13 \leq h \leq 11$, $-17 \leq k \leq 17$, $-17 \leq l \leq 17$
Reflections collected	41592
Independent reflections	8271 [R_{int} = 0.0472, R_{sigma} = 0.0382]
Data/restraints/parameters	8271/0/431
Goodness-of-fit on F^2	1.026
Final R indexes [I>=2σ (I)]	R_1 = 0.0460, wR_2 = 0.1069
Final R indexes [all data]	R_1 = 0.0703, wR_2 = 0.1197
Largest diff. peak/hole / e Å^{-3}	0.60/-0.53

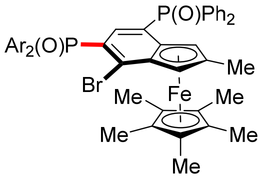

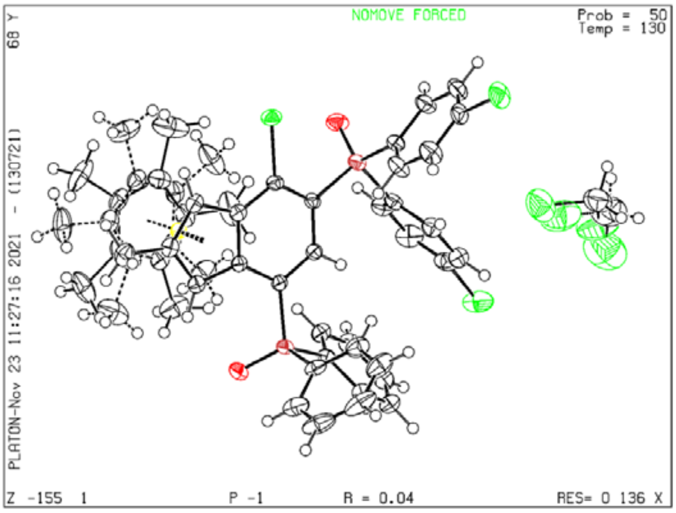

Supplementary Table 10. Crystal data and structure refinement for 24.

Identification code	**24**	
Empirical formula	$C_{45} H_{41} Br Cl_4 Fe O_2 P_2$	
Formula weight	953.28	
Temperature	130(2) K	
Wavelength	0.71073 Å	
Crystal system	Triclinic	
Space group	P-1	
Unit cell dimensions	a = 9.410(3) Å	α= 80.879(12)°.
	b = 11.192(4) Å	β= 82.635(12)°.
	c = 21.451(8) Å	γ = 74.048(12)°.
Volume	2136.2(13) Å^3	
Z	2	
Density (calculated)	1.482 Mg/m^3	
Absorption coefficient	1.651 mm^{-1}	
F(000)	972	
Crystal size	0.150 x 0.120 x 0.120 mm^3	
Theta range for data collection	2.247 to 27.462°.	
Index ranges	-12<=h<=12, -14<=k<=14, -27<=l<=27	
Reflections collected	44557	
Independent reflections	9761 [R(int) = 0.0667]	
Completeness to theta = 25.242°	99.9 %	
Absorption correction	Semi-empirical from equivalents	
Max. and min. transmission	0.7456 and 0.6534	
Refinement method	Full-matrix least-squares on F^2	
Data / restraints / parameters	9761 / 704 / 626	
Goodness-of-fit on F^2	1.027	
Final R indices [I>2sigma(I)]	R1 = 0.0427, wR2 = 0.1041	
R indices (all data)	R1 = 0.0593, wR2 = 0.1141	
Extinction coefficient	n/a	
Largest diff. peak and hole	0.979 and -0.681 e.Å^{-3}	

3.10. Copies of NMR spectra

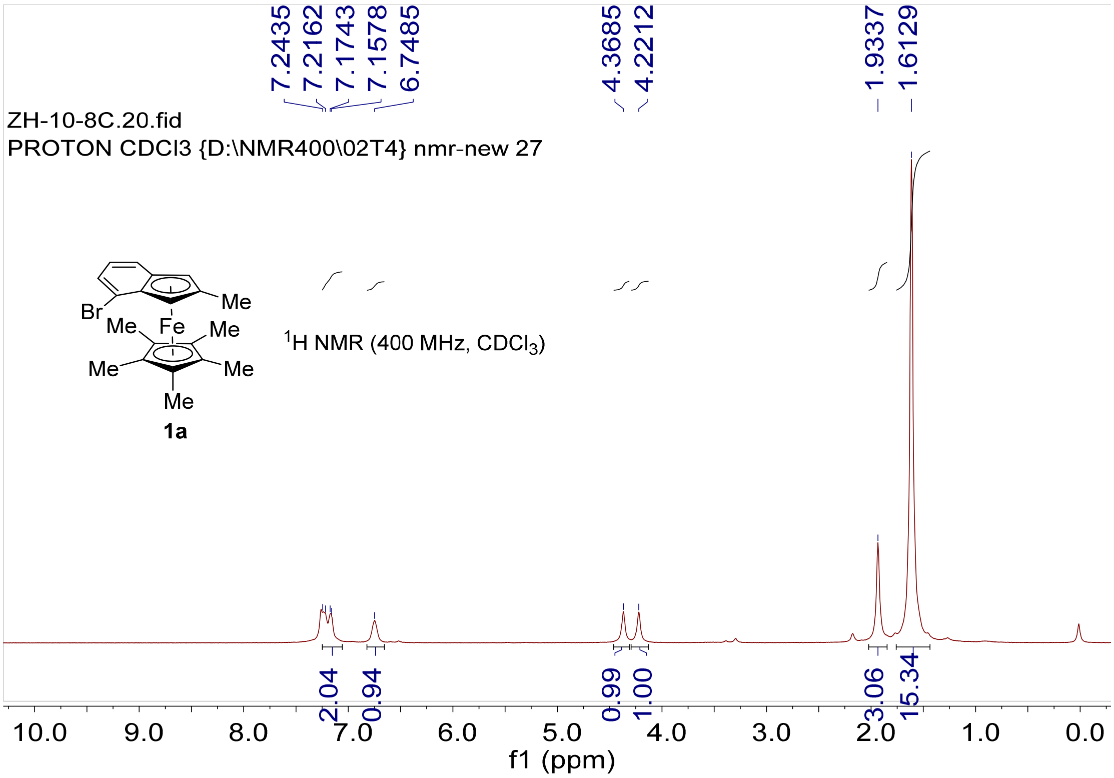

Supplementary Figure 7. ^{1}H NMR spectra of compound 1a

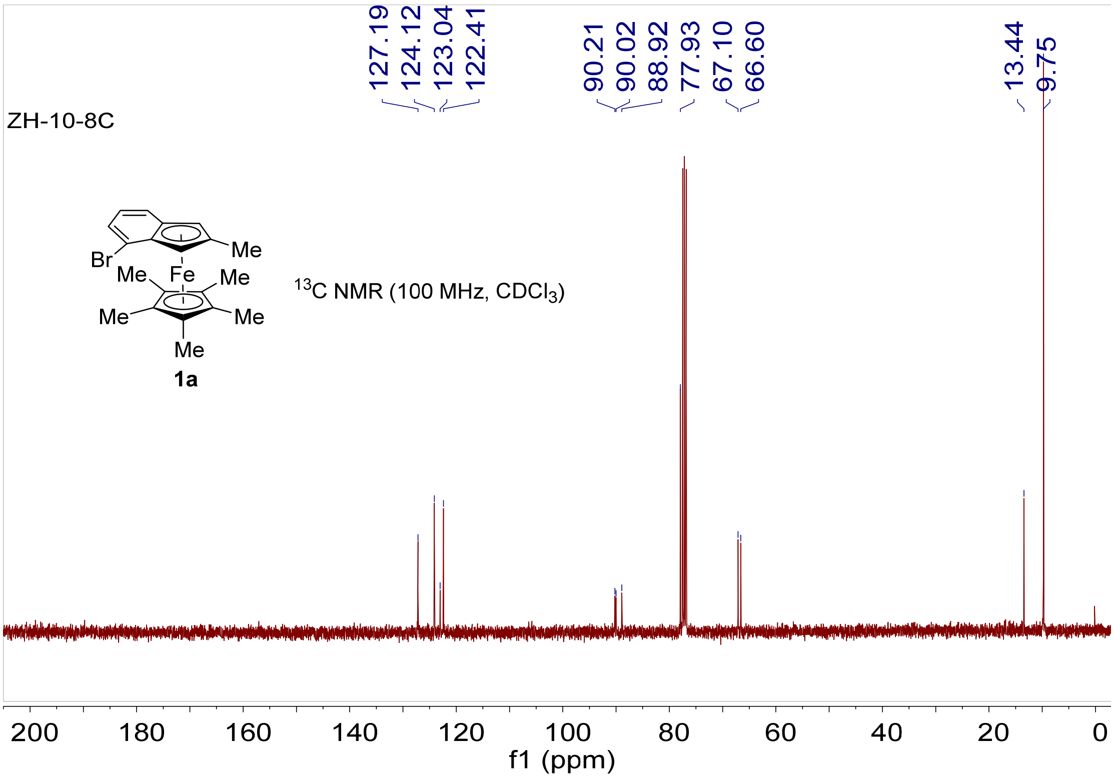

Supplementary Figure 8. ^{13}C NMR spectra of compound 1a

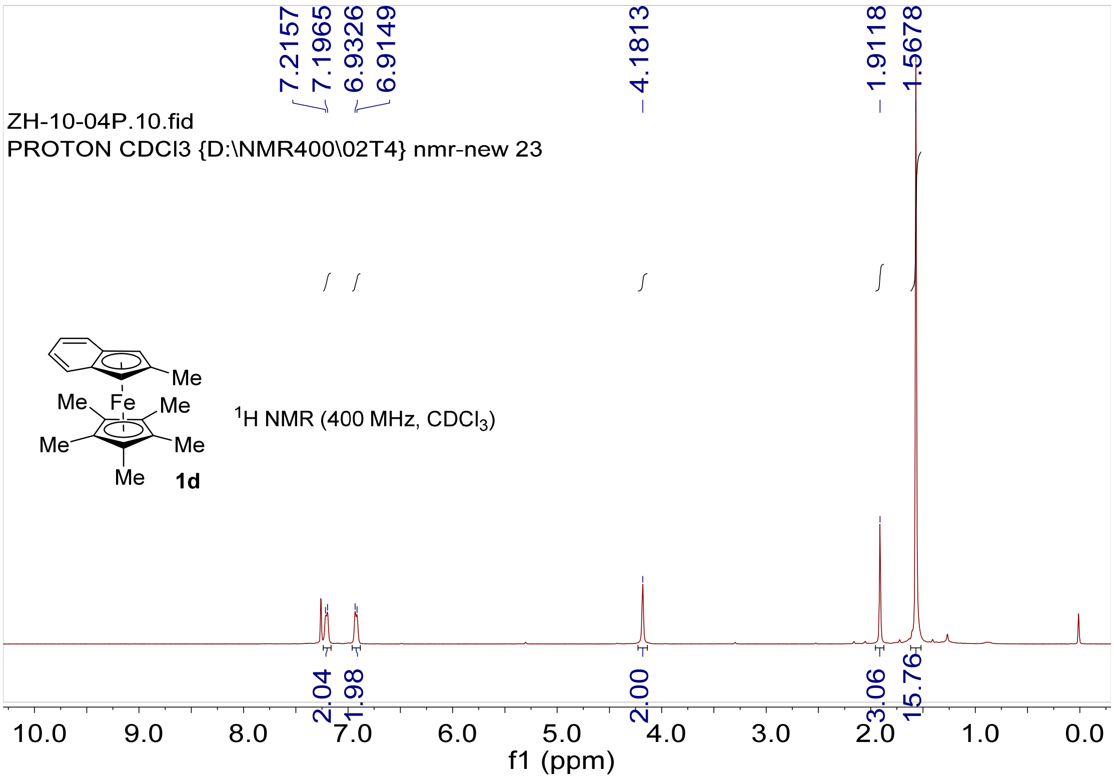

Supplementary Figure 9. ^{1}H NMR spectra of compound 1d

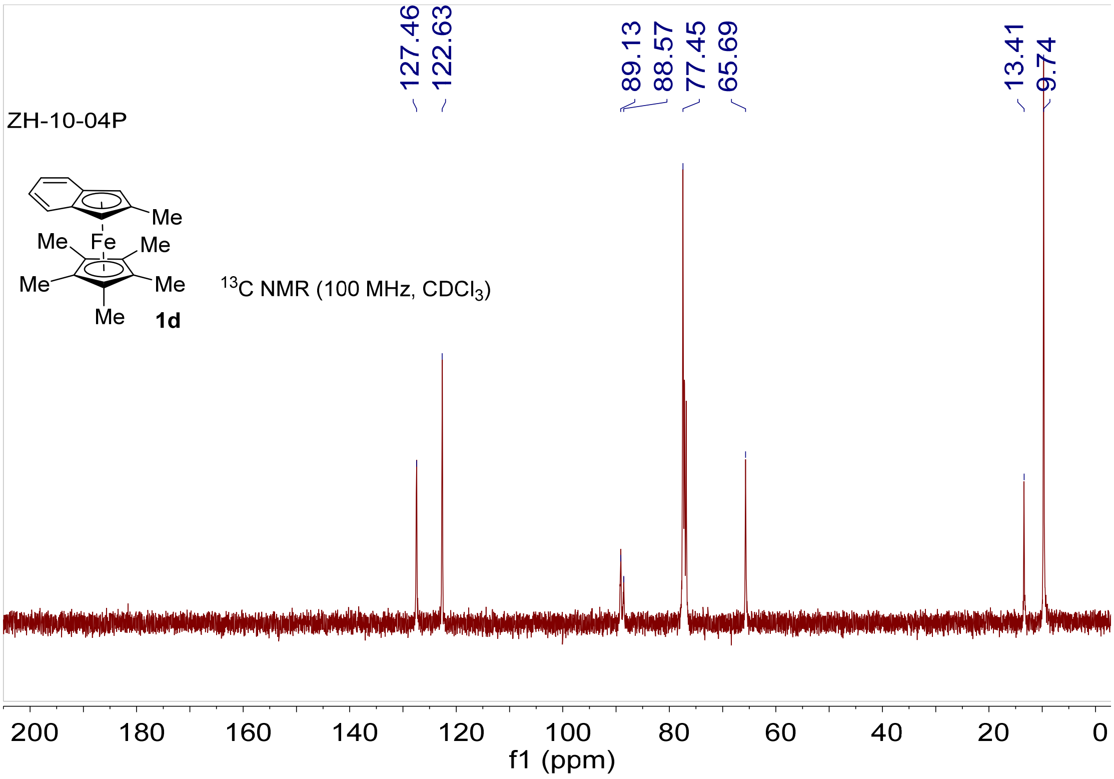

Supplementary Figure 10. ^{13}C NMR spectra of compound 1d

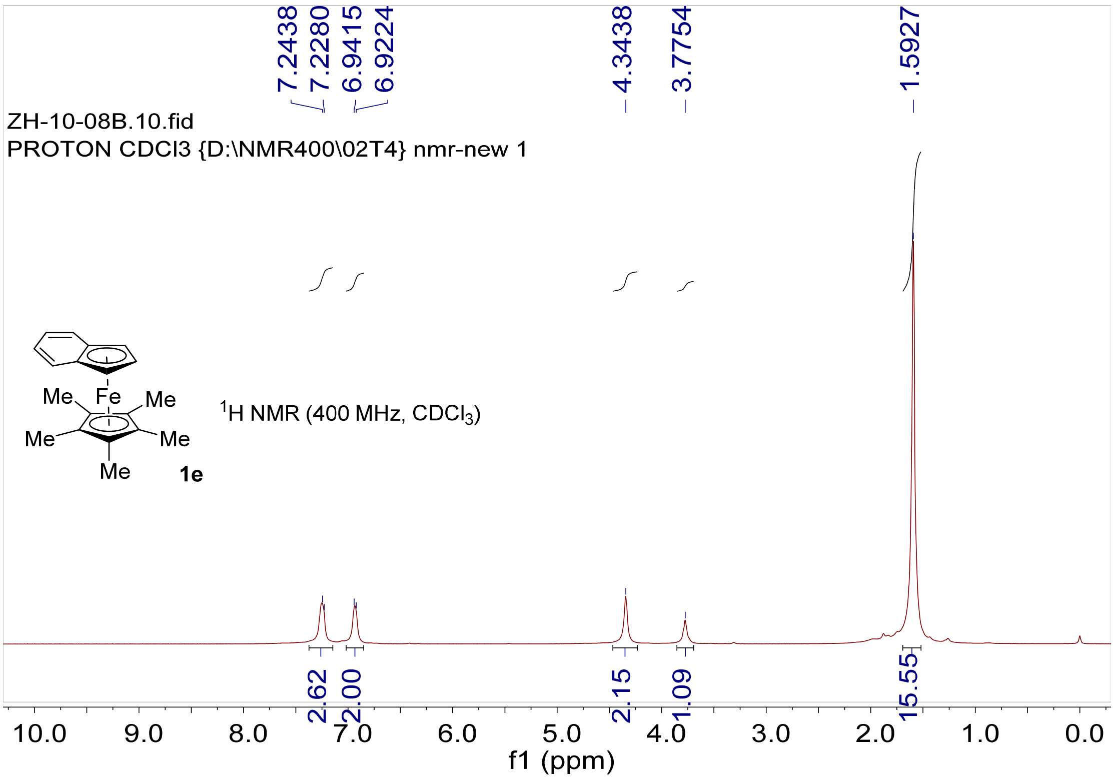

Supplementary Figure 11. ^{1}H NMR spectra of compound 1e

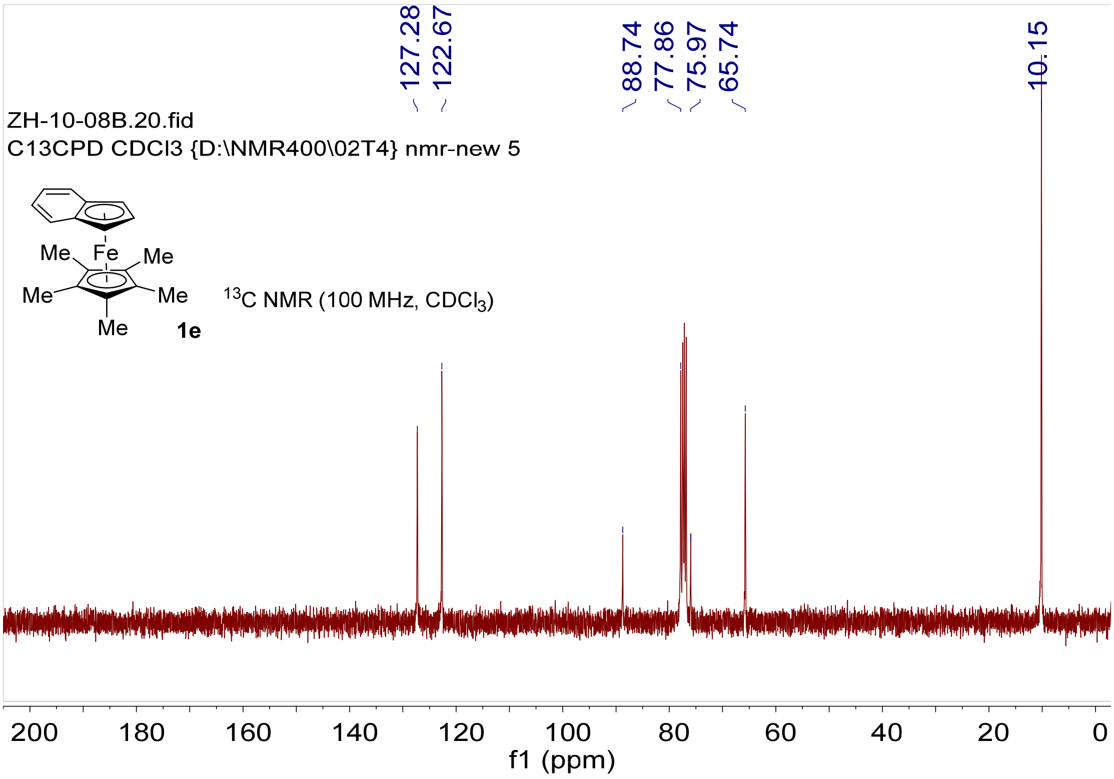

Supplementary Figure 12. ^{13}C NMR spectra of compound 1e

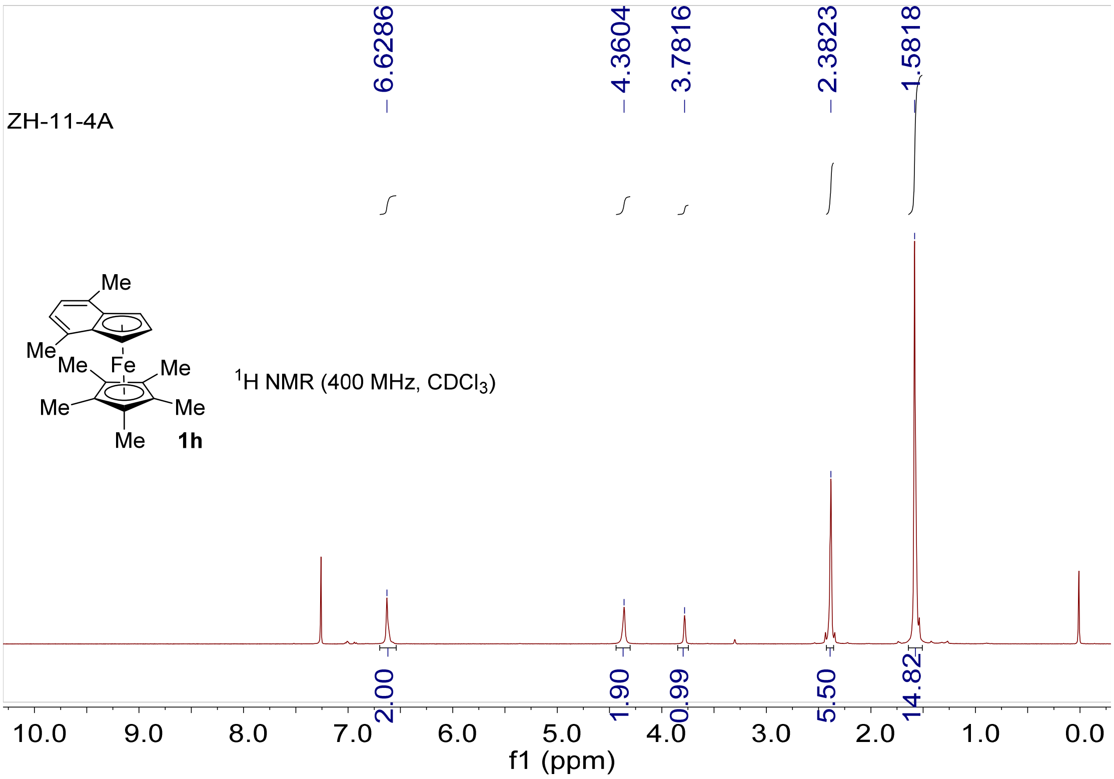

Supplementary Figure 13. ^{1}H NMR spectra of compound 1h

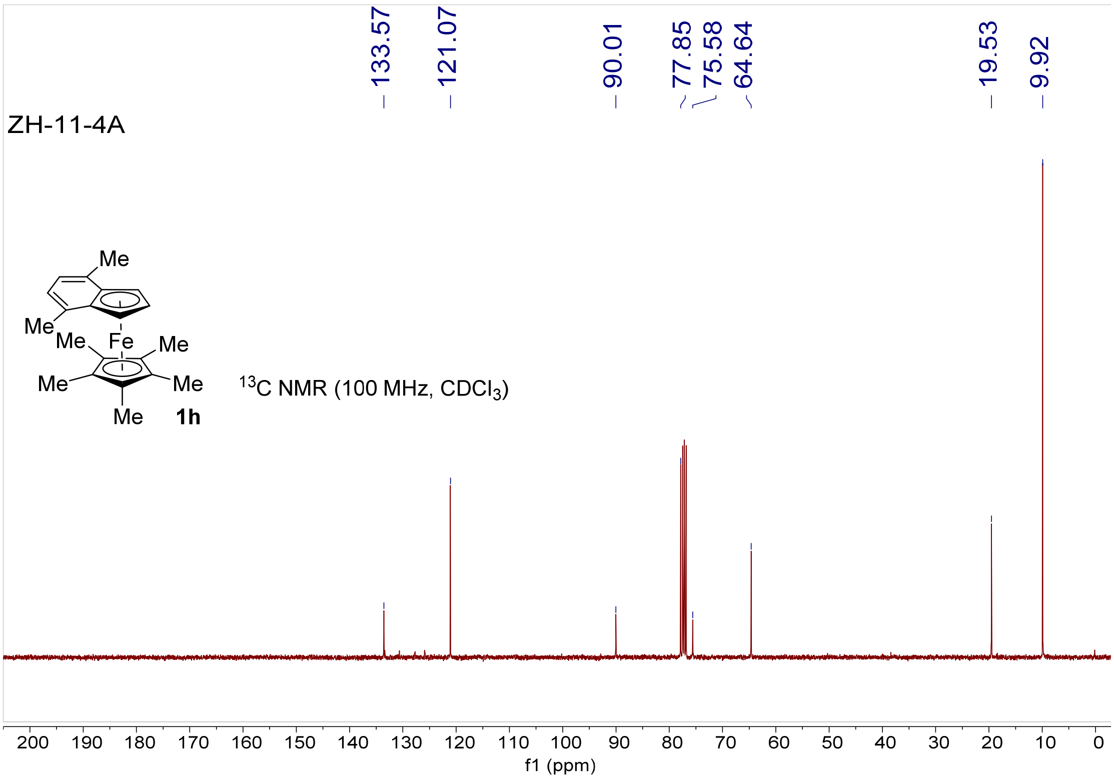

Supplementary Figure 14. ^{13}C NMR spectra of compound 1h

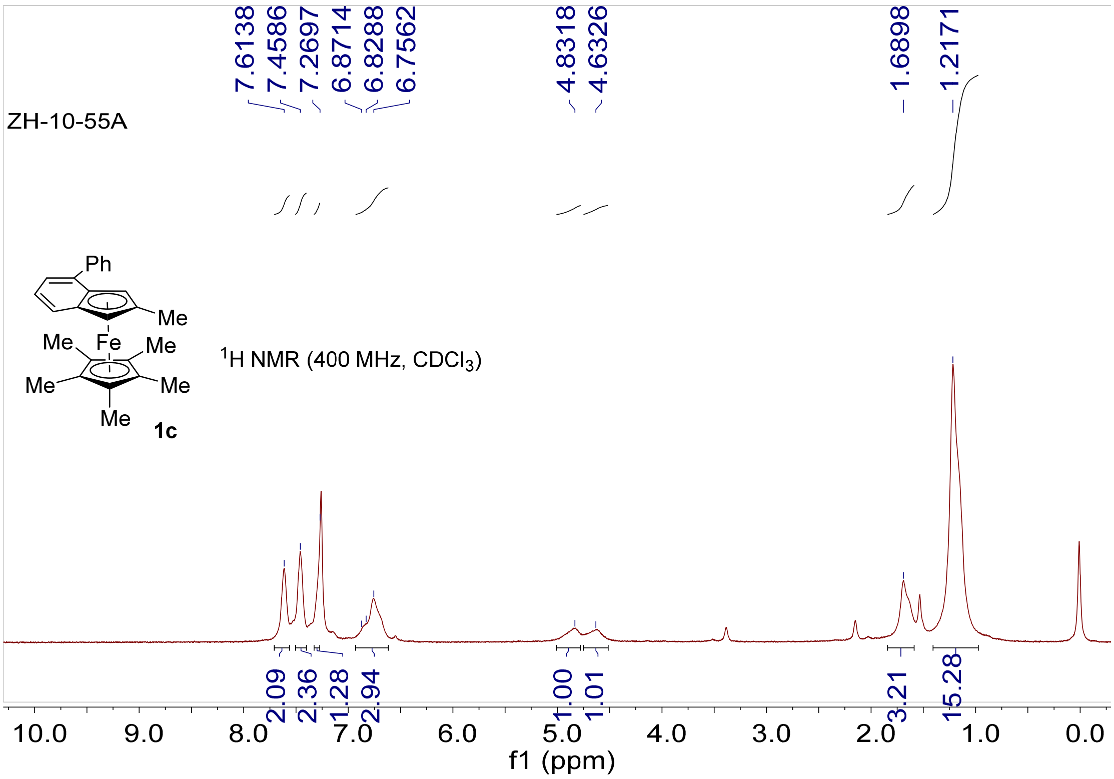

Supplementary Figure 15. ^{1}H NMR spectra of compound 1c

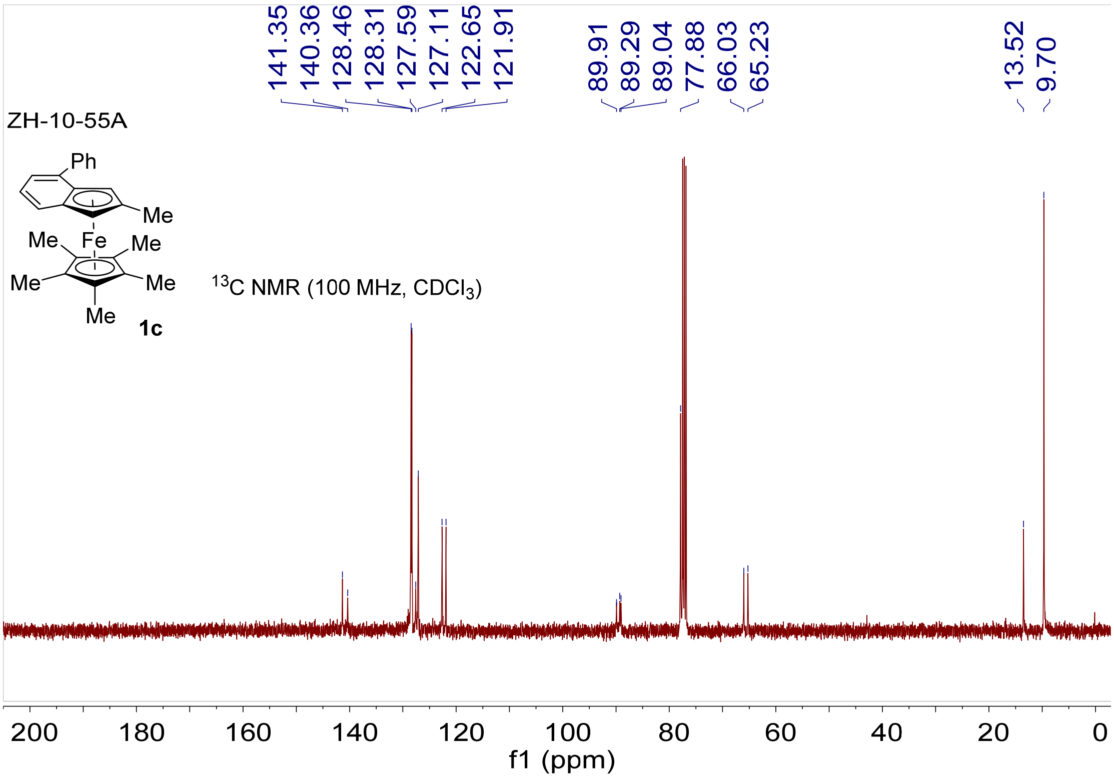

Supplementary Figure 16. ^{13}C NMR spectra of compound 1c

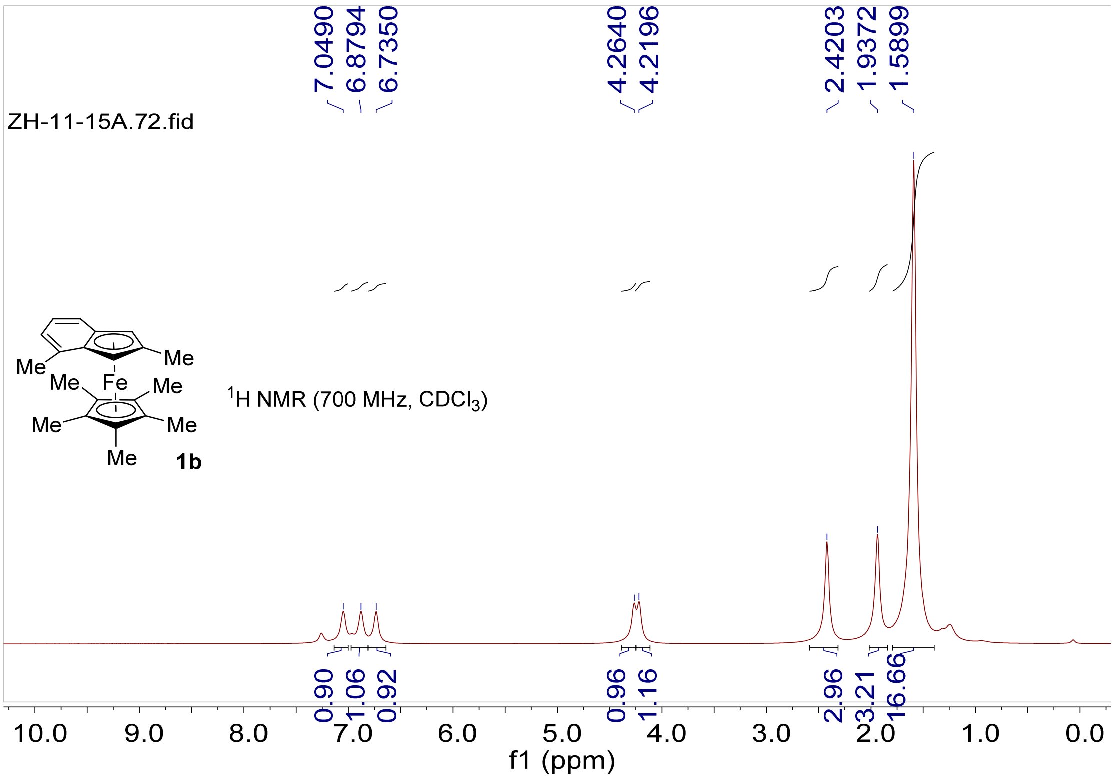

Supplementary Figure 17. ^{1}H NMR spectra of compound 1b

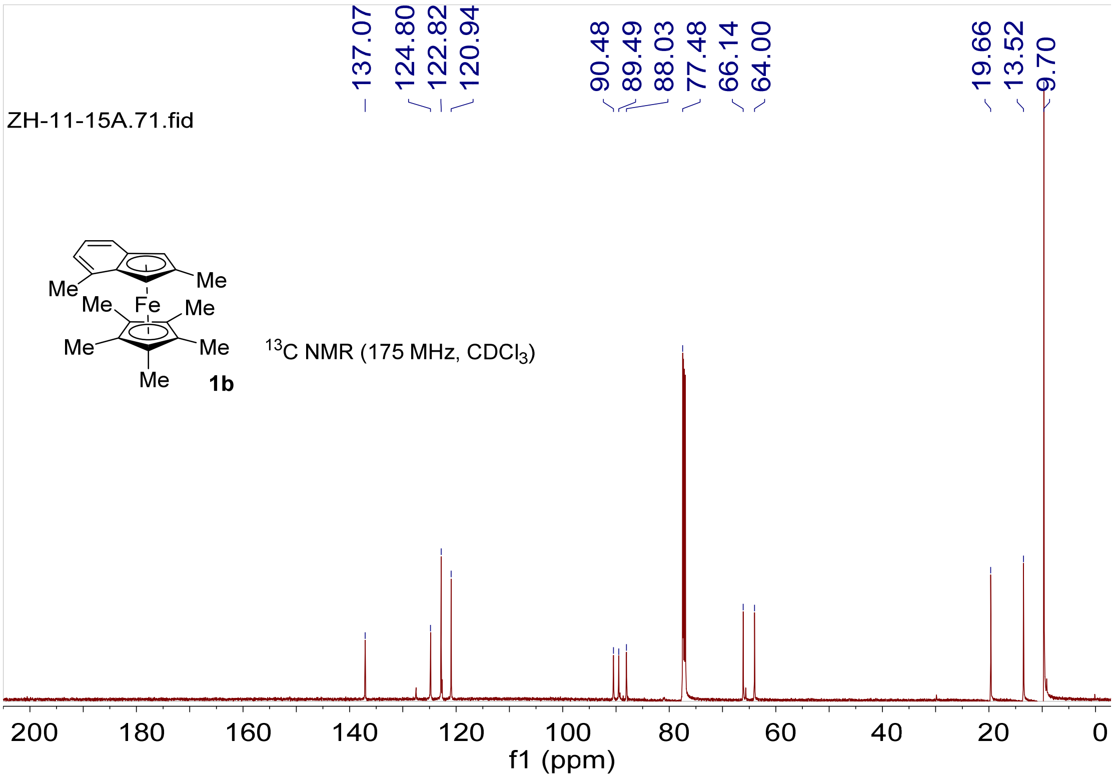

Supplementary Figure 18. ^{13}C NMR spectra of compound 1b

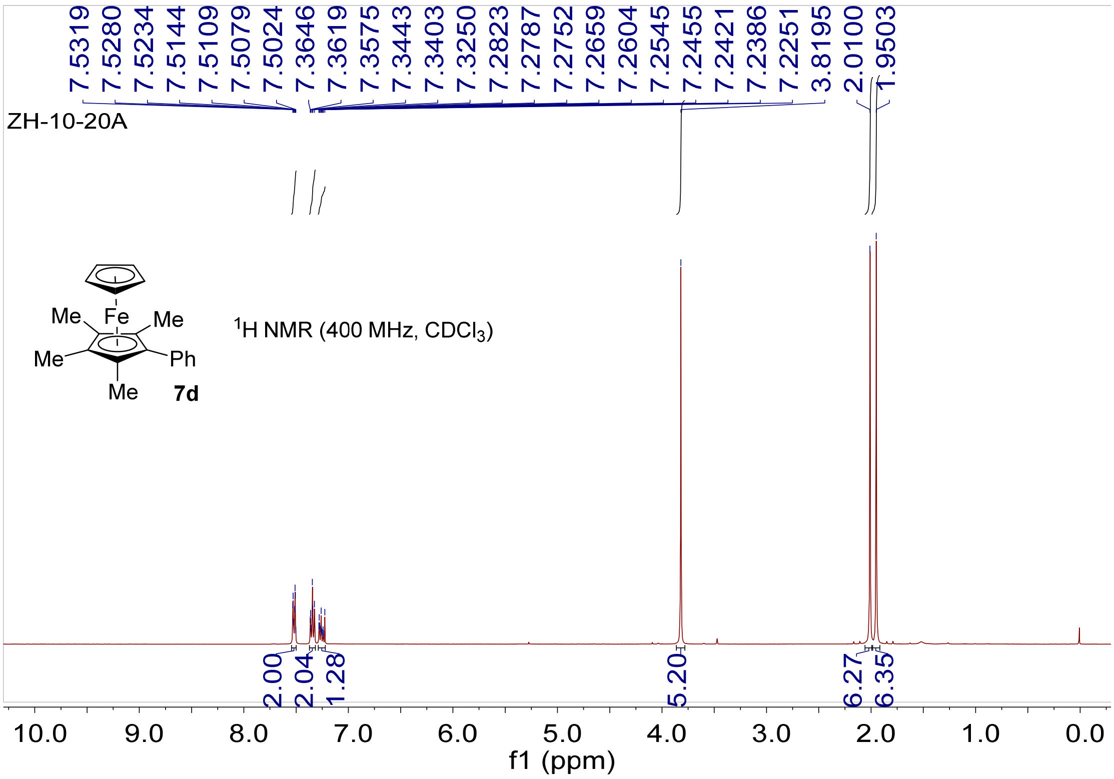

Supplementary Figure 19. ^{1}H NMR spectra of compound 7d

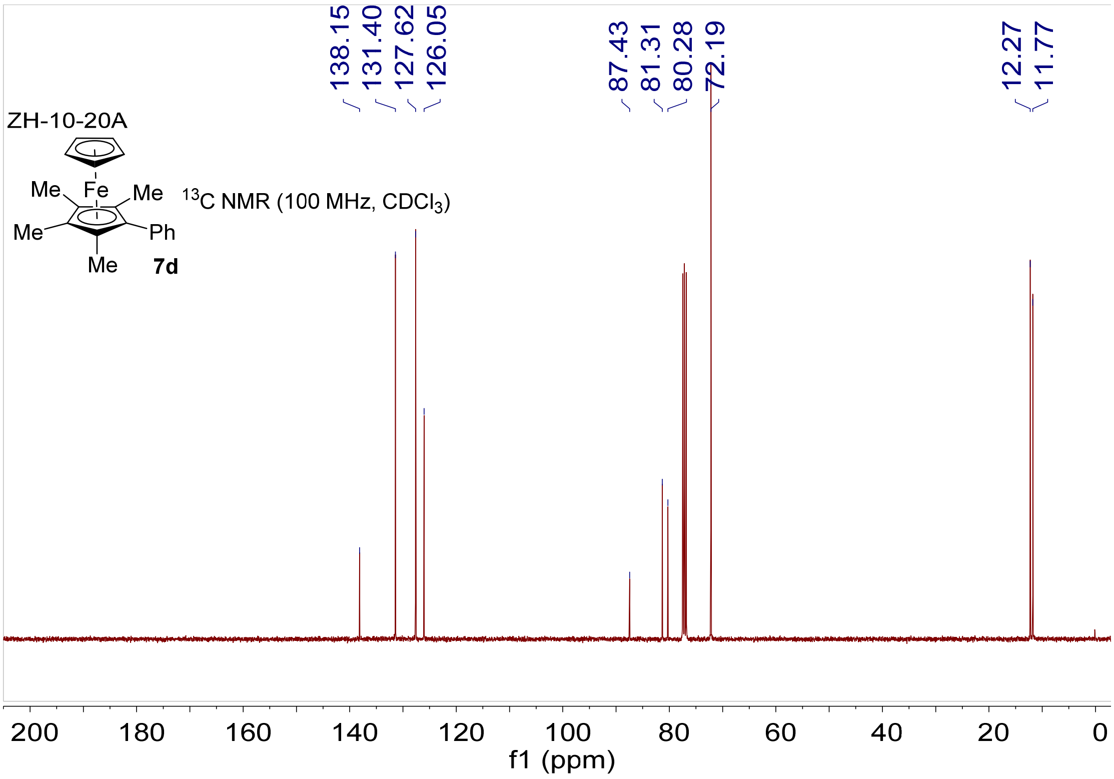

Supplementary Figure 20. ^{13}C NMR spectra of compound 7d

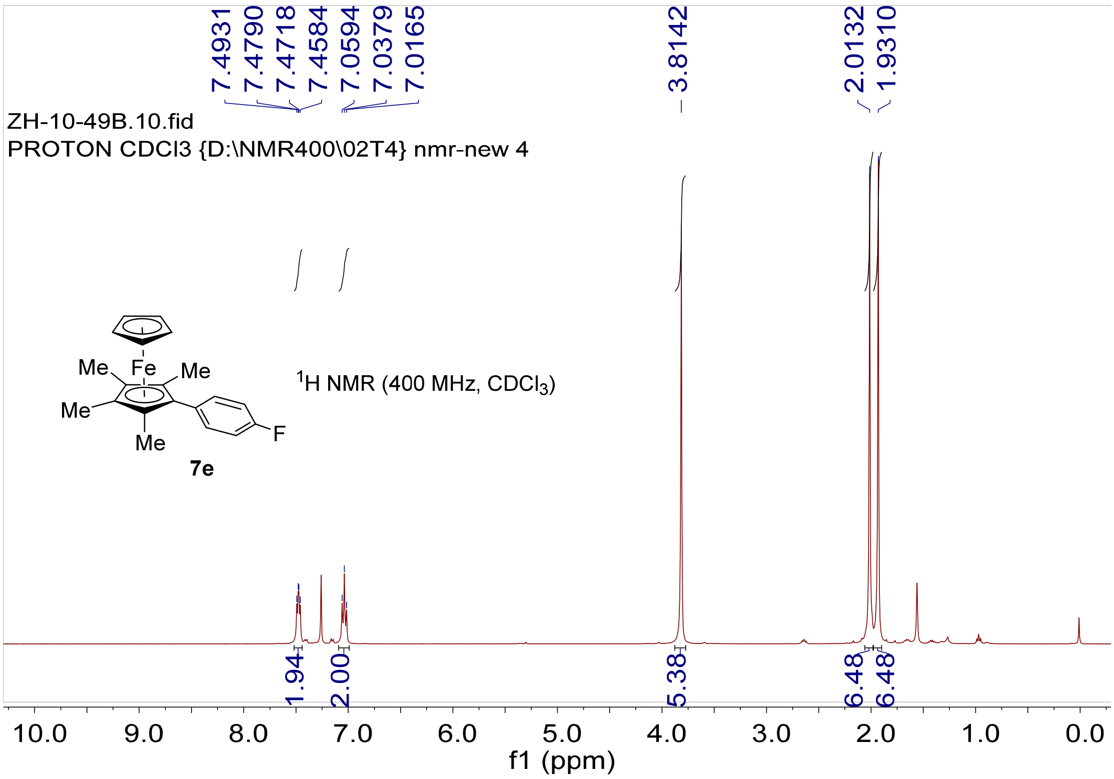

Supplementary Figure 21. ^{1}H NMR spectra of compound 7e

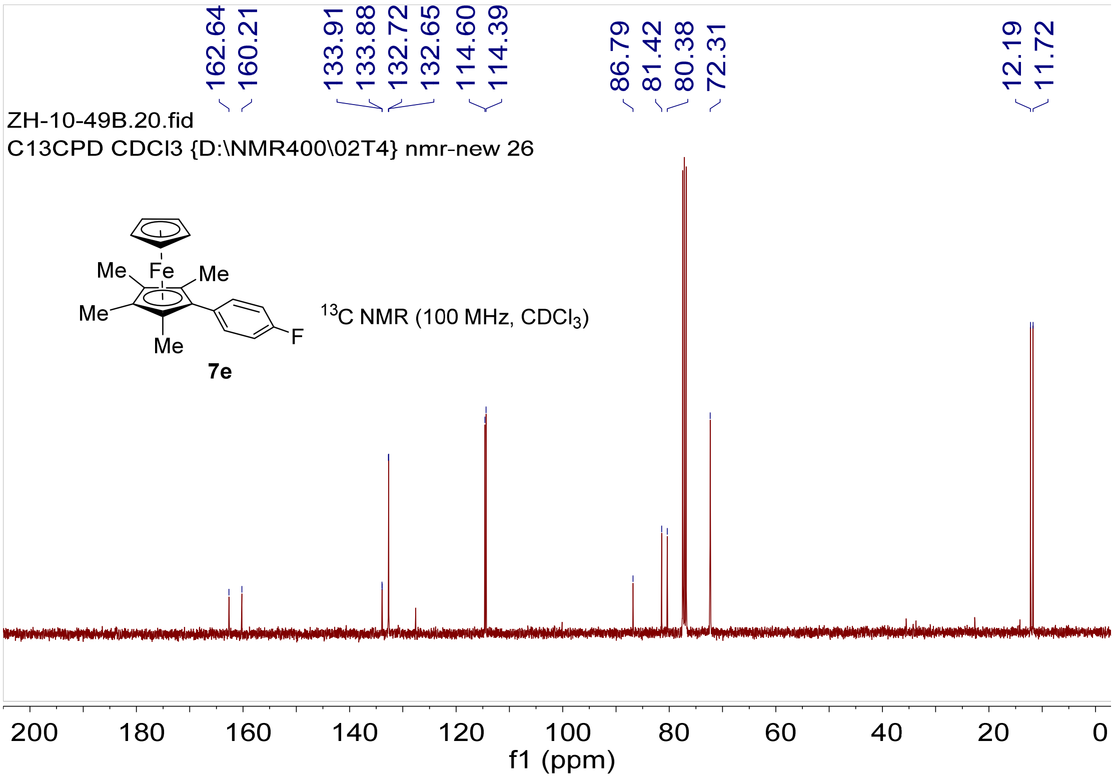

Supplementary Figure 22. ^{13}C NMR spectra of compound 7e

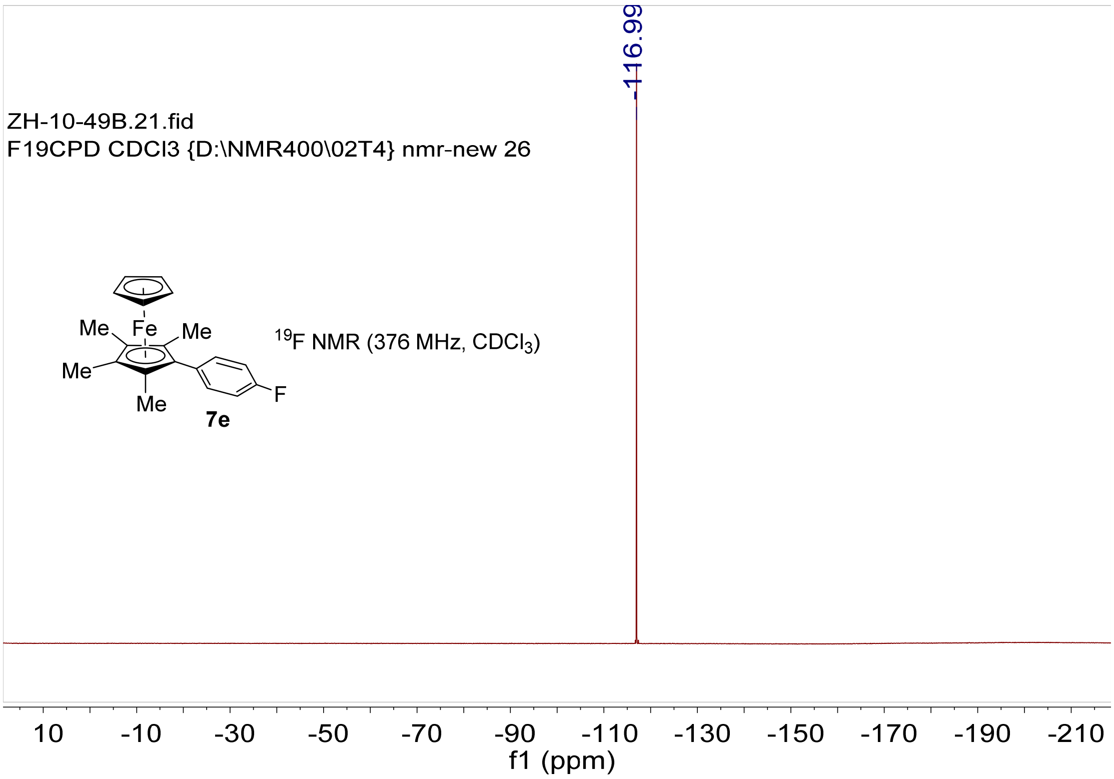

Supplementary Figure 23. ^{19}F NMR spectra of compound 7e

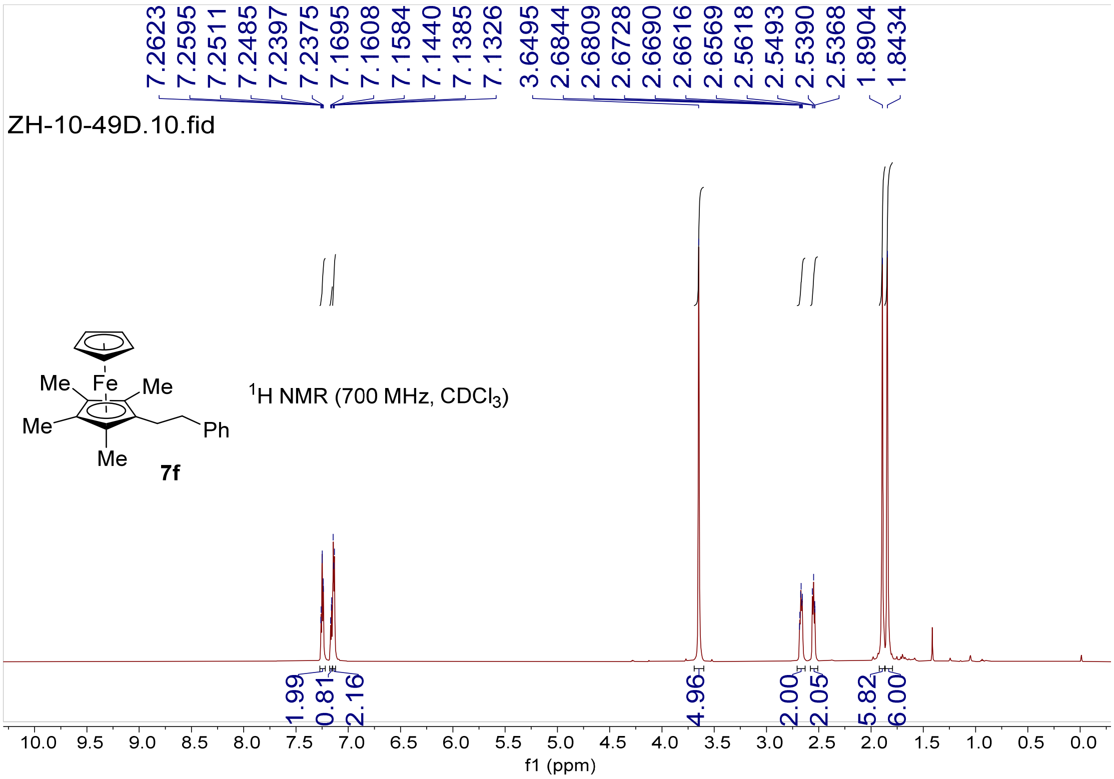

Supplementary Figure 24. ^{1}**H NMR spectra of compound 7f**

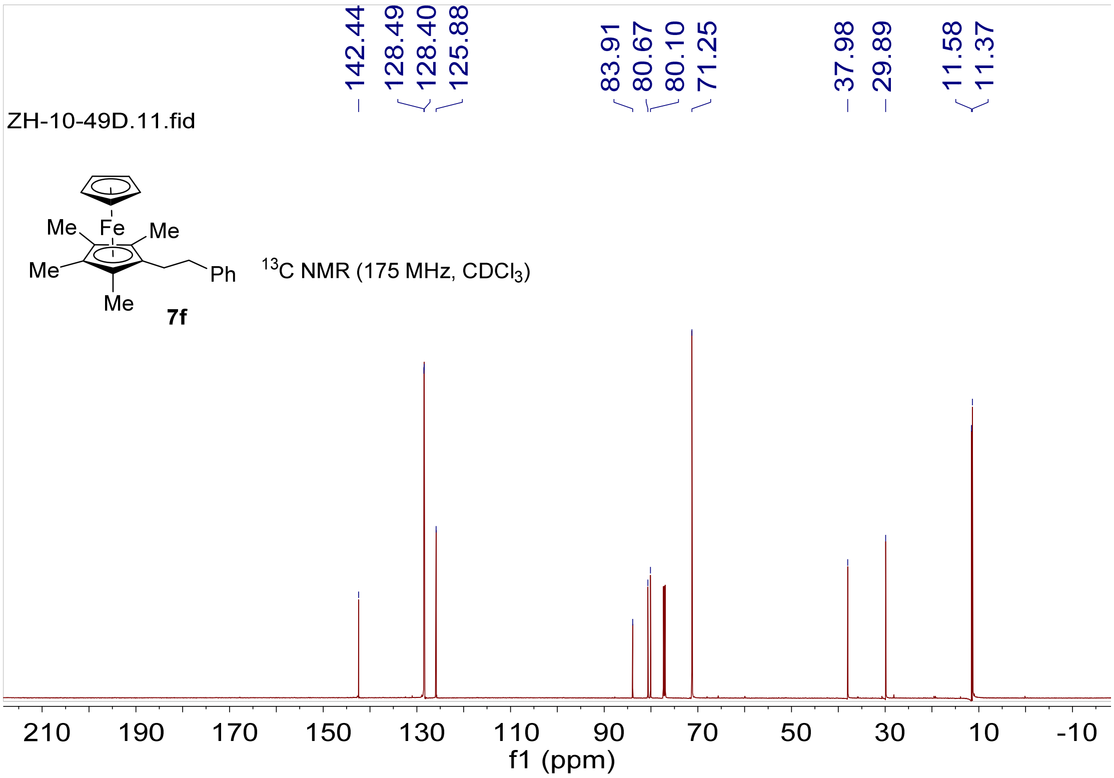

Supplementary Figure 25. ^{13}**C NMR spectra of compound 7f**

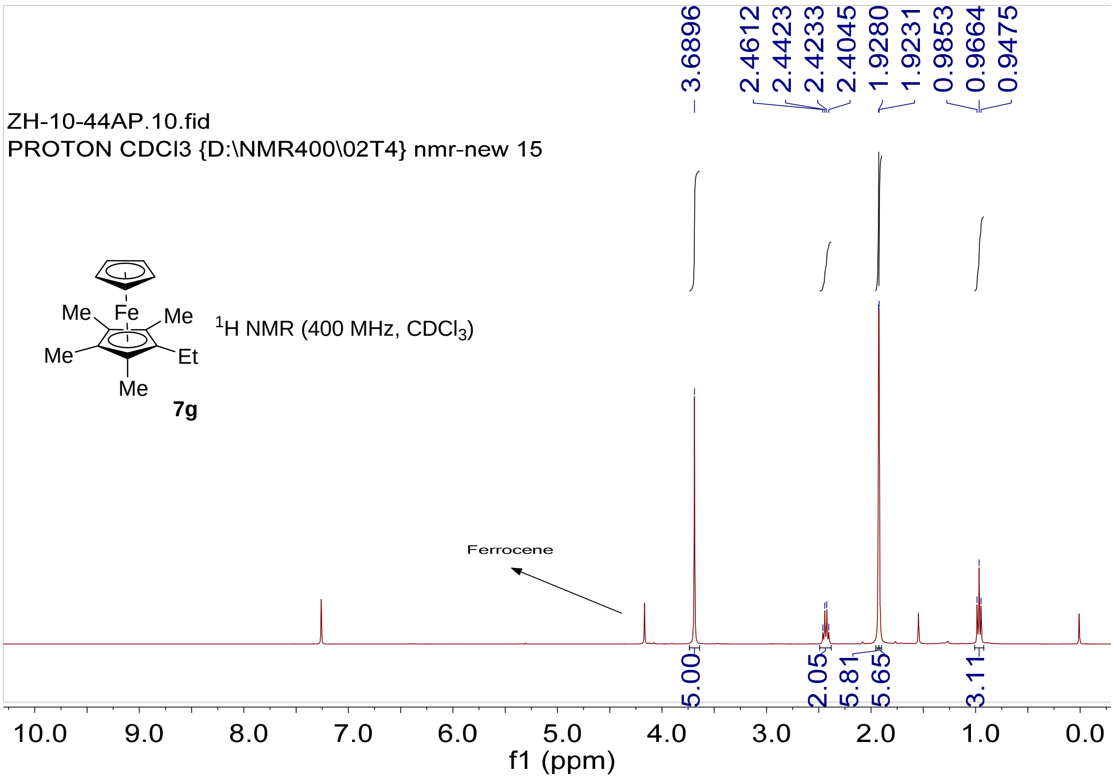

Supplementary Figure 26. ^{1}H NMR spectra of compound 7g

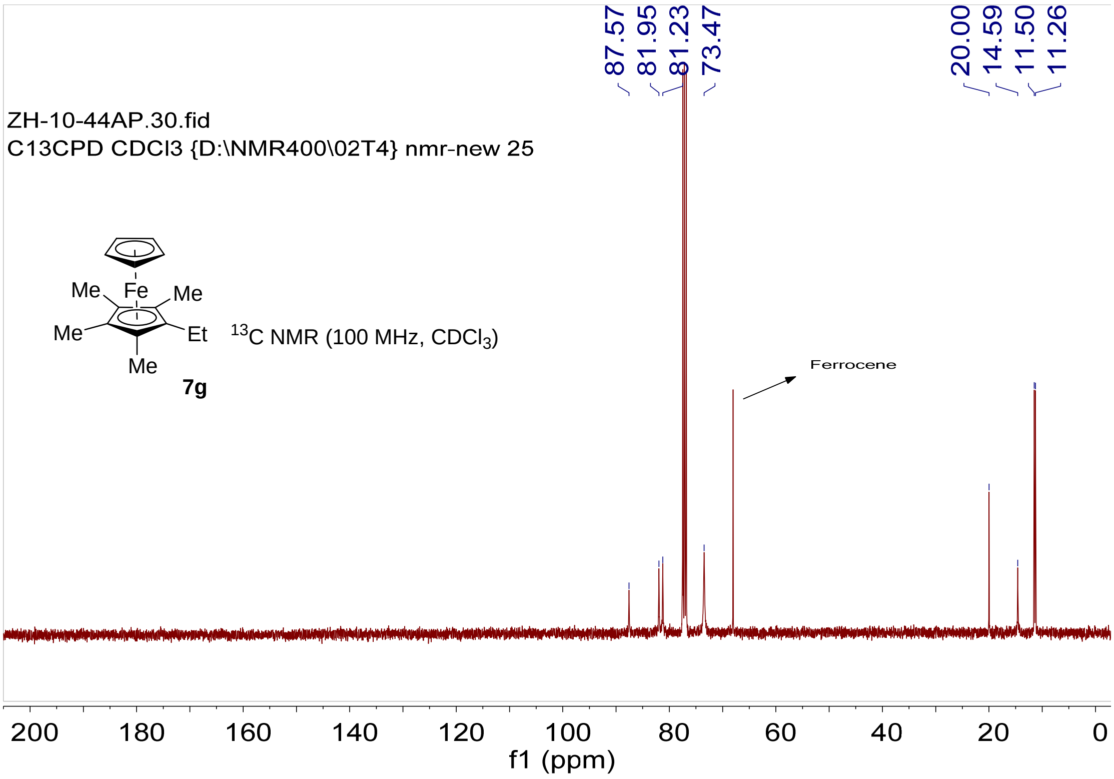

Supplementary Figure 27. ^{13}C NMR spectra of compound 7g

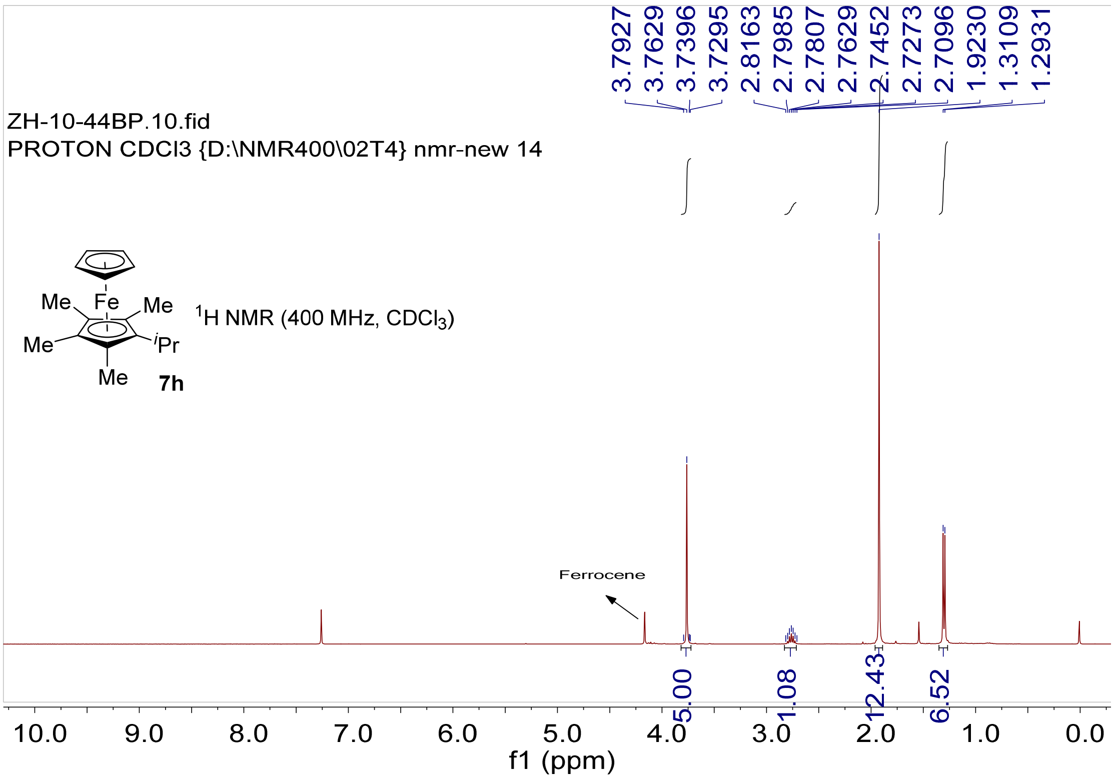

Supplementary Figure 28. ^{1}**H NMR spectra of compound 7h**

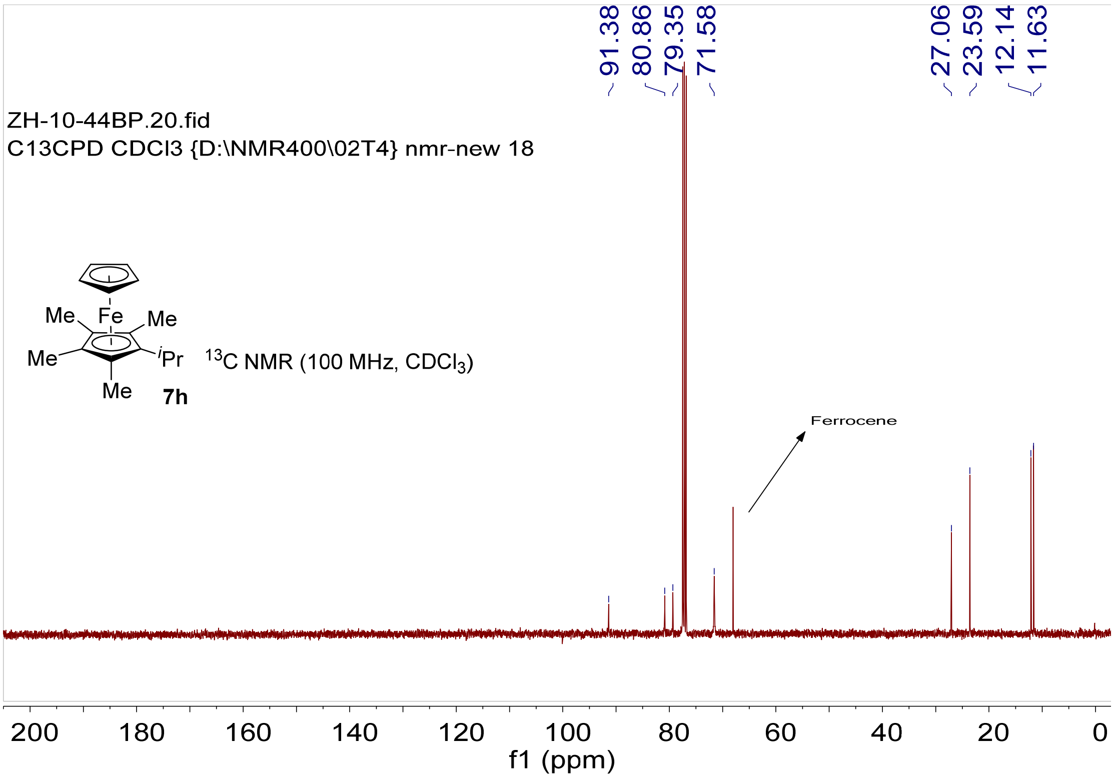

Supplementary Figure 29. ^{13}**C NMR spectra of compound 7h**

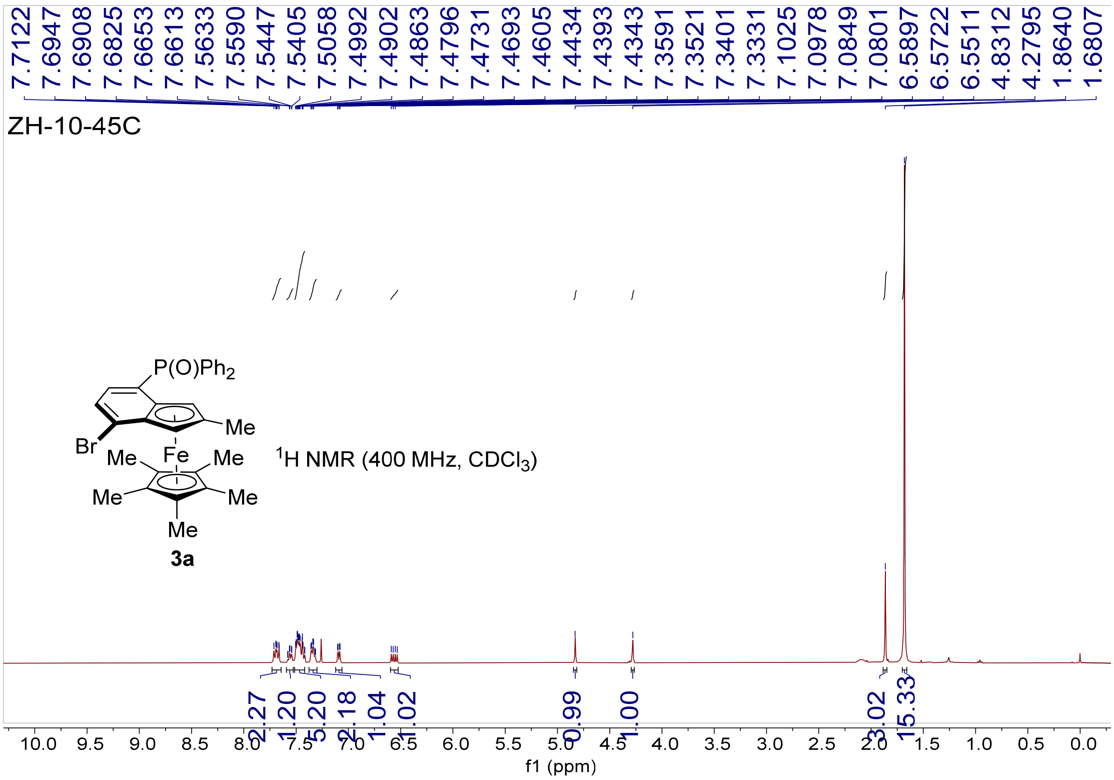

Supplementary Figure 30. ^{1}H NMR spectra of compound 3a

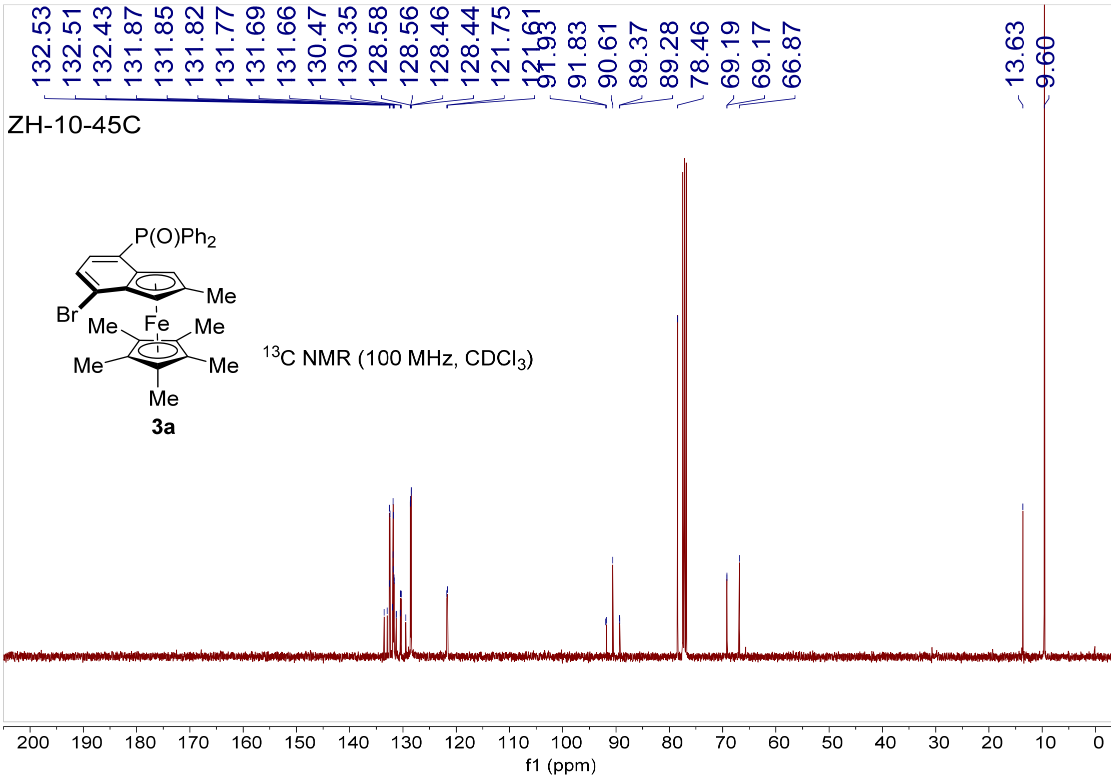

Supplementary Figure 31. ^{13}C NMR spectra of compound 3a

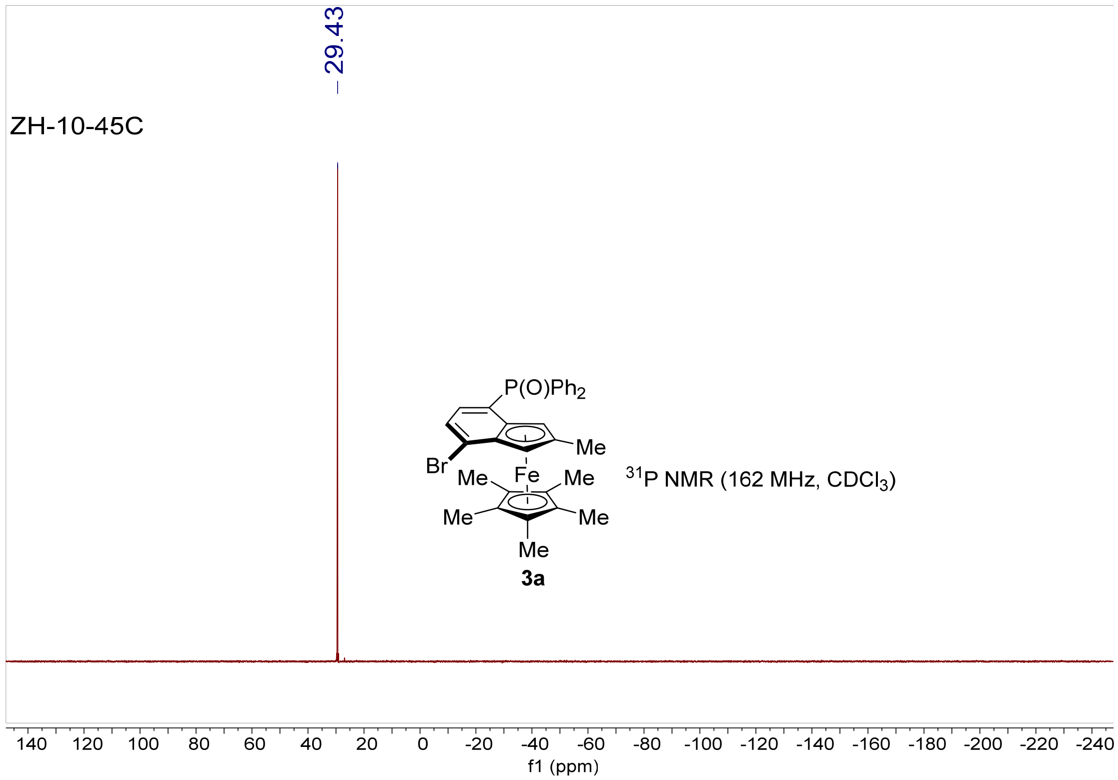

Supplementary Figure 32. ^{31}P NMR spectra of compound 3a

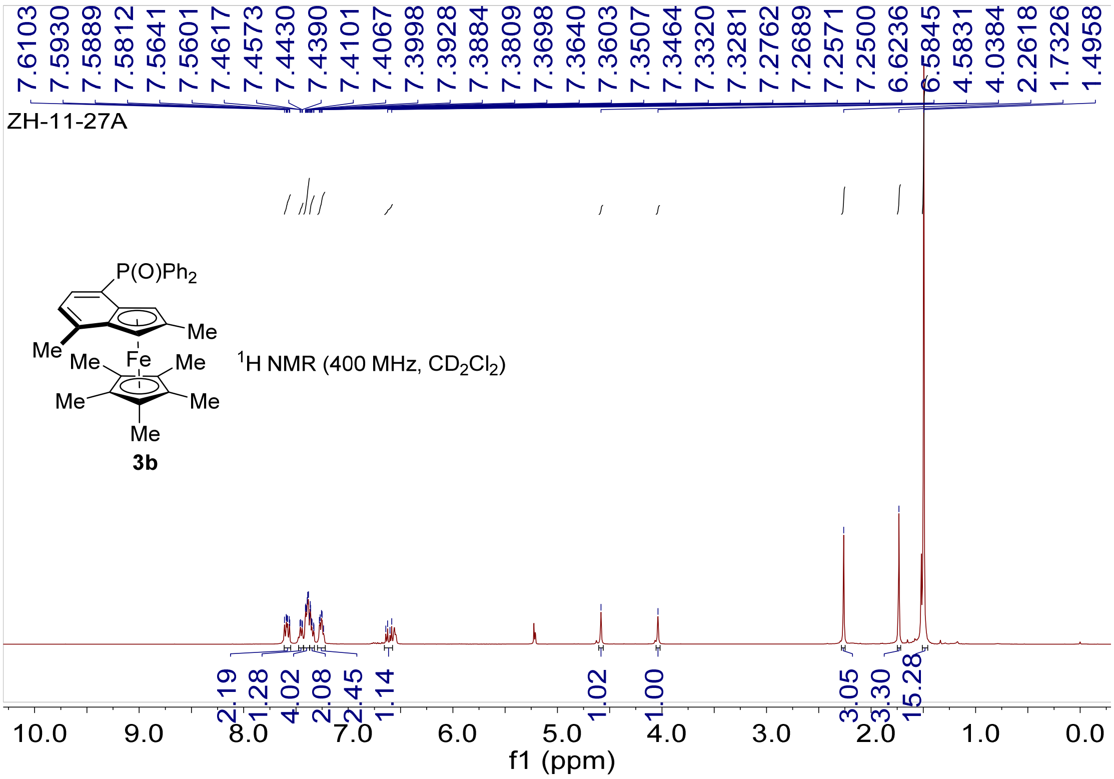

Supplementary Figure 33. ^{1}H NMR spectra of compound 3b

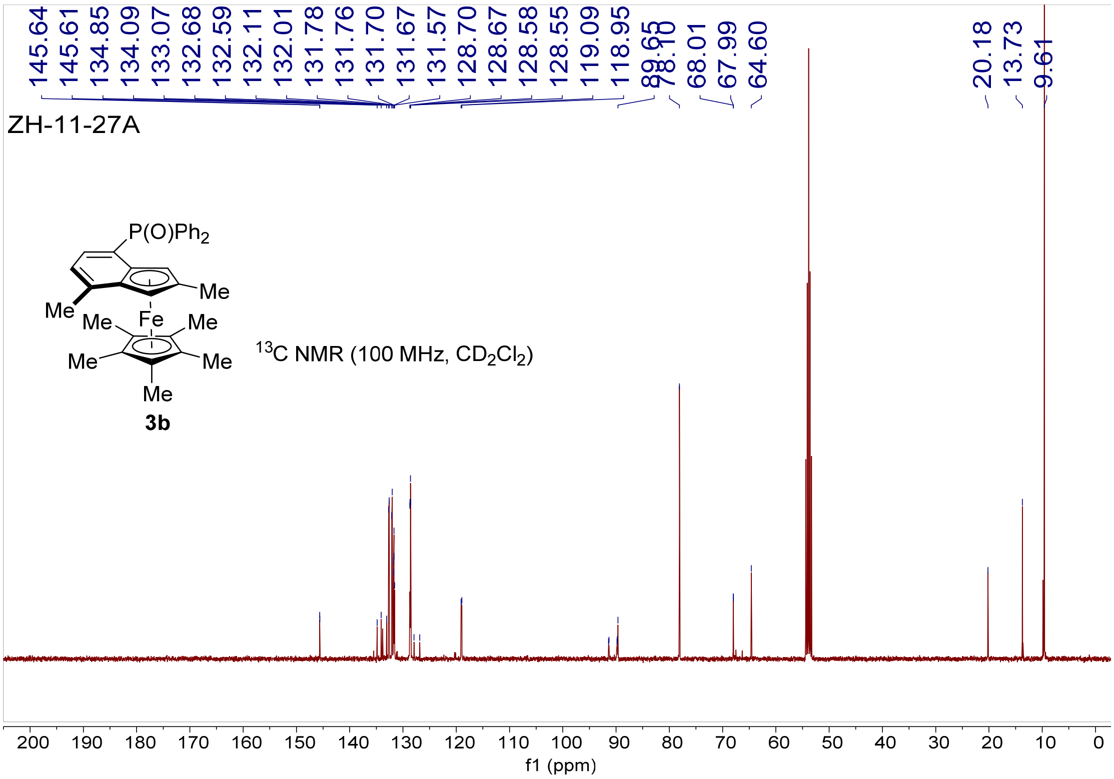

Supplementary Figure 34. ^{13}C NMR spectra of compound 3b

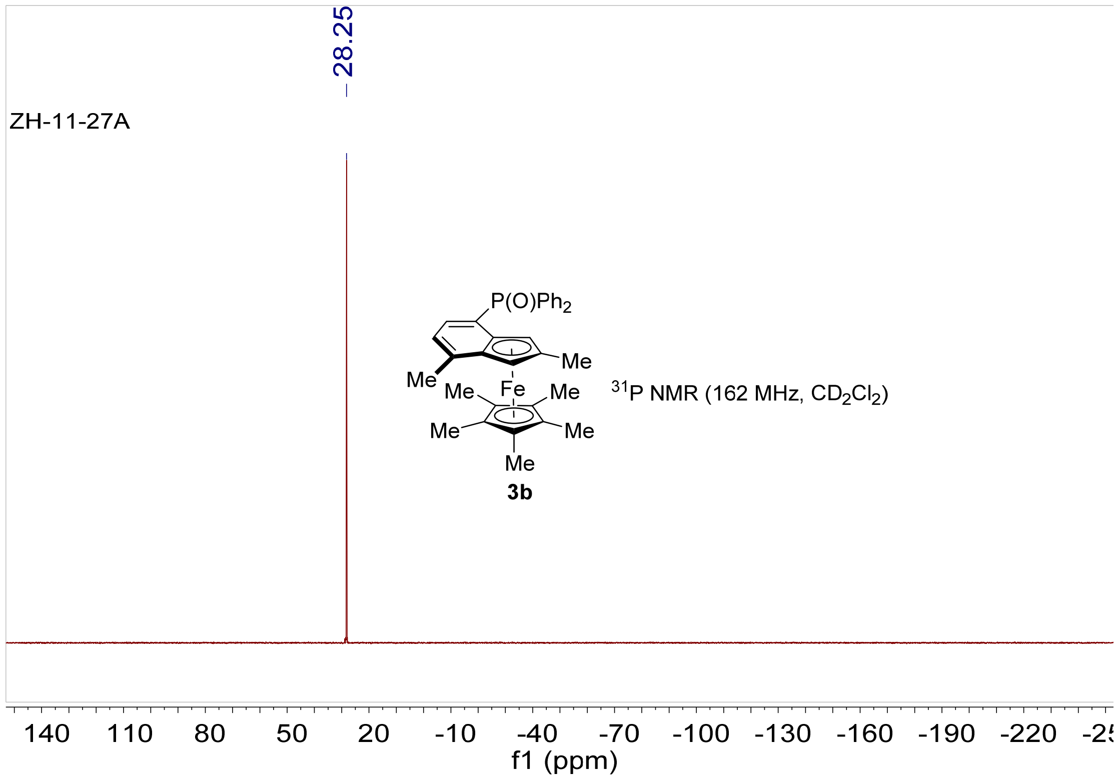

Supplementary Figure 35. ^{31}P NMR spectra of compound 3b

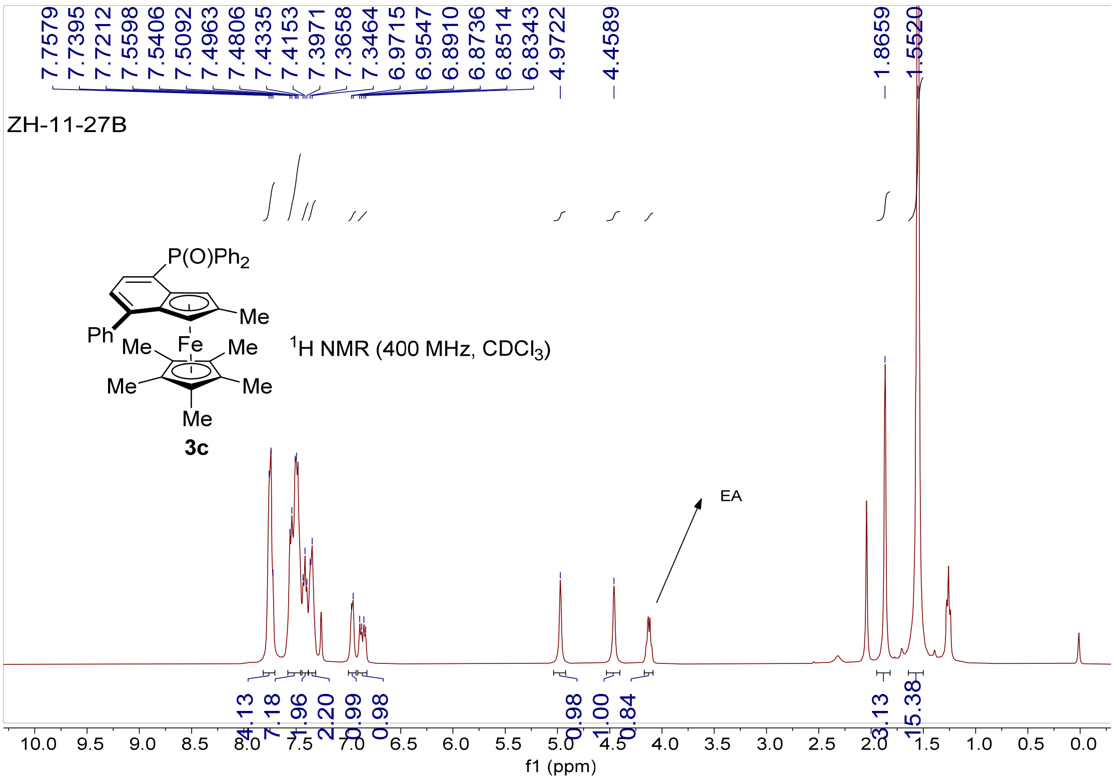

Supplementary Figure 36. ^{1}H NMR spectra of compound 3c

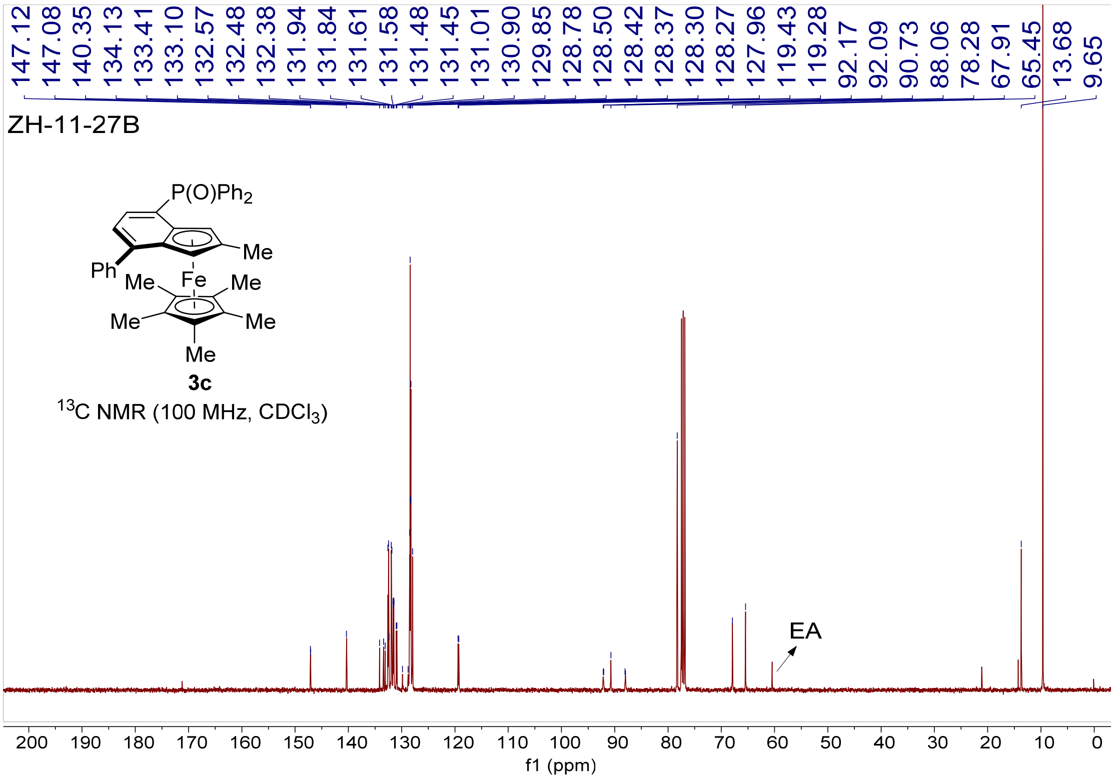

Supplementary Figure 37. ^{13}C NMR spectra of compound 3c

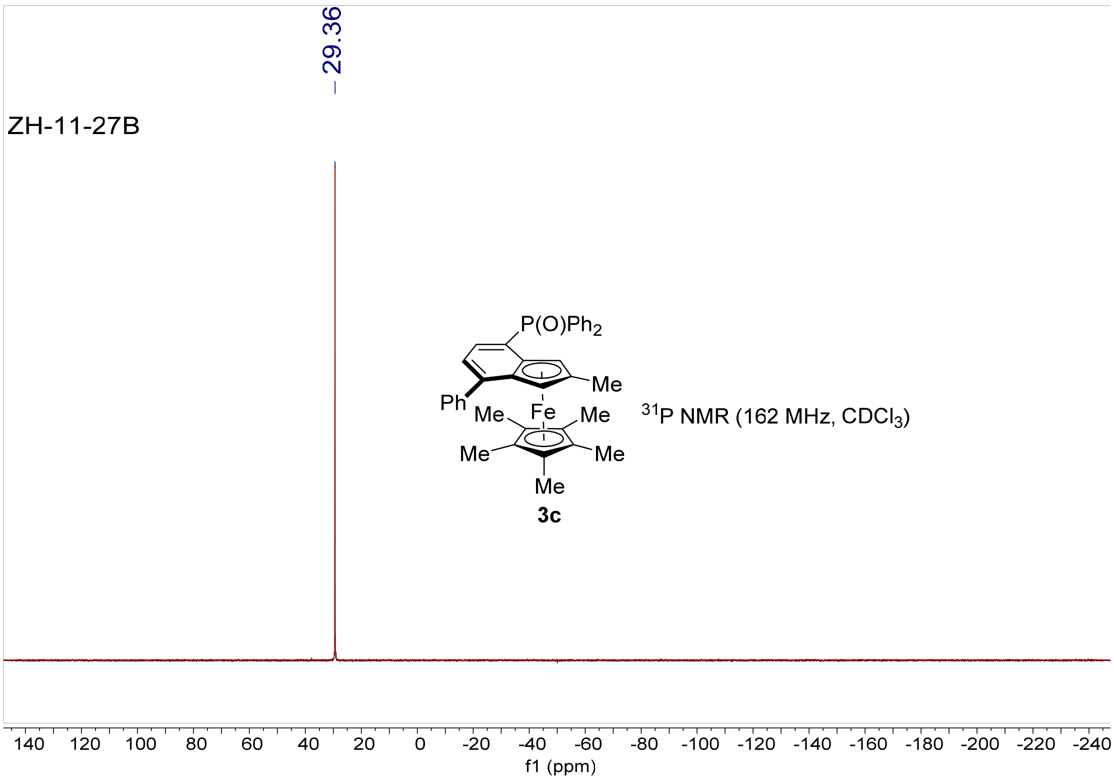

Supplementary Figure 38. ^{31}P NMR spectra of compound 3c

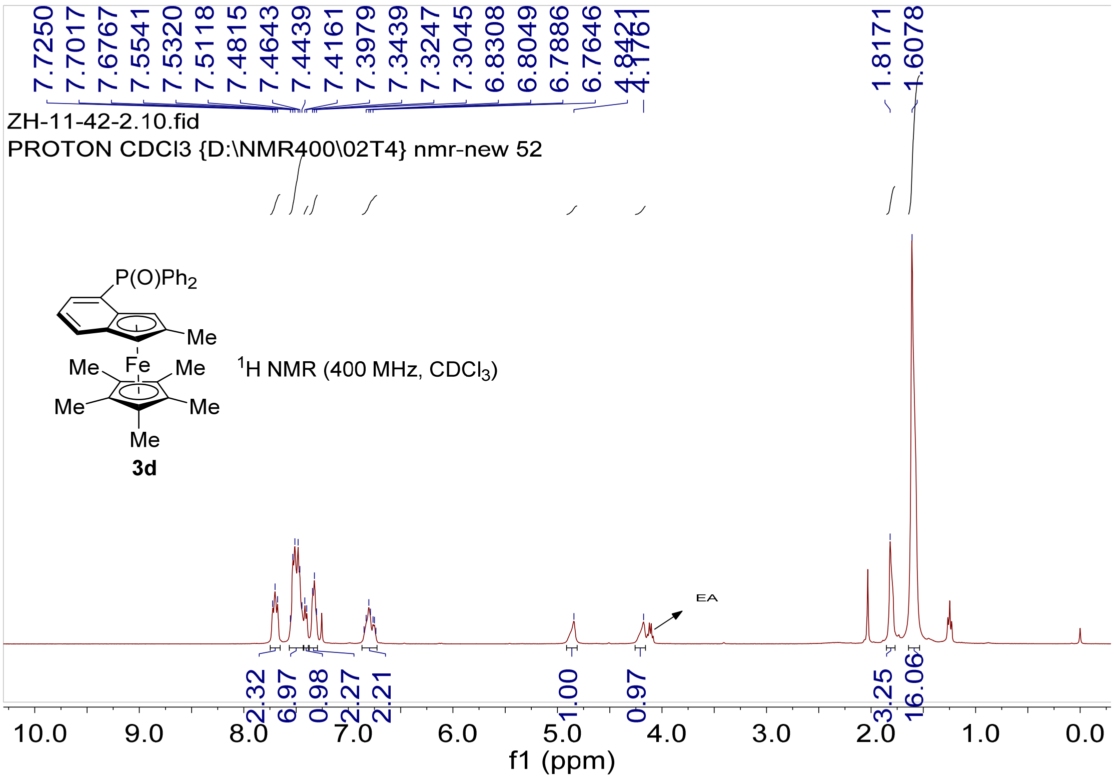

Supplementary Figure 39. ^{1}H NMR spectra of compound 3d

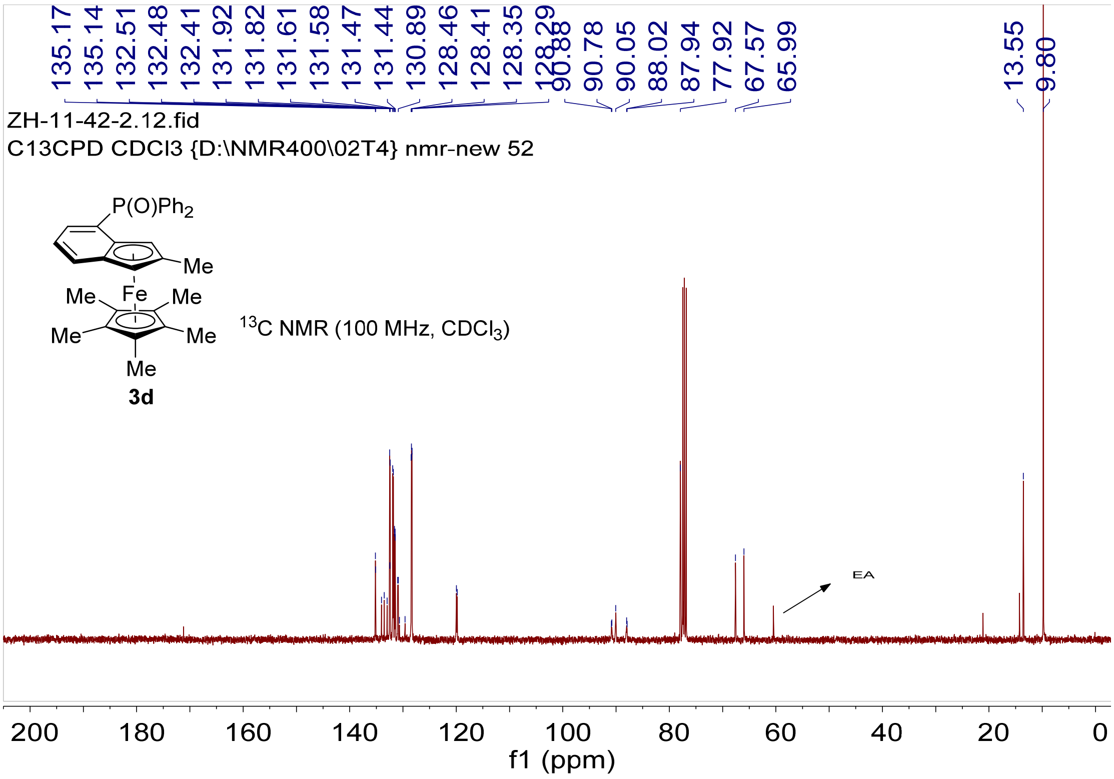

Supplementary Figure 40. ^{13}C NMR spectra of compound 3d

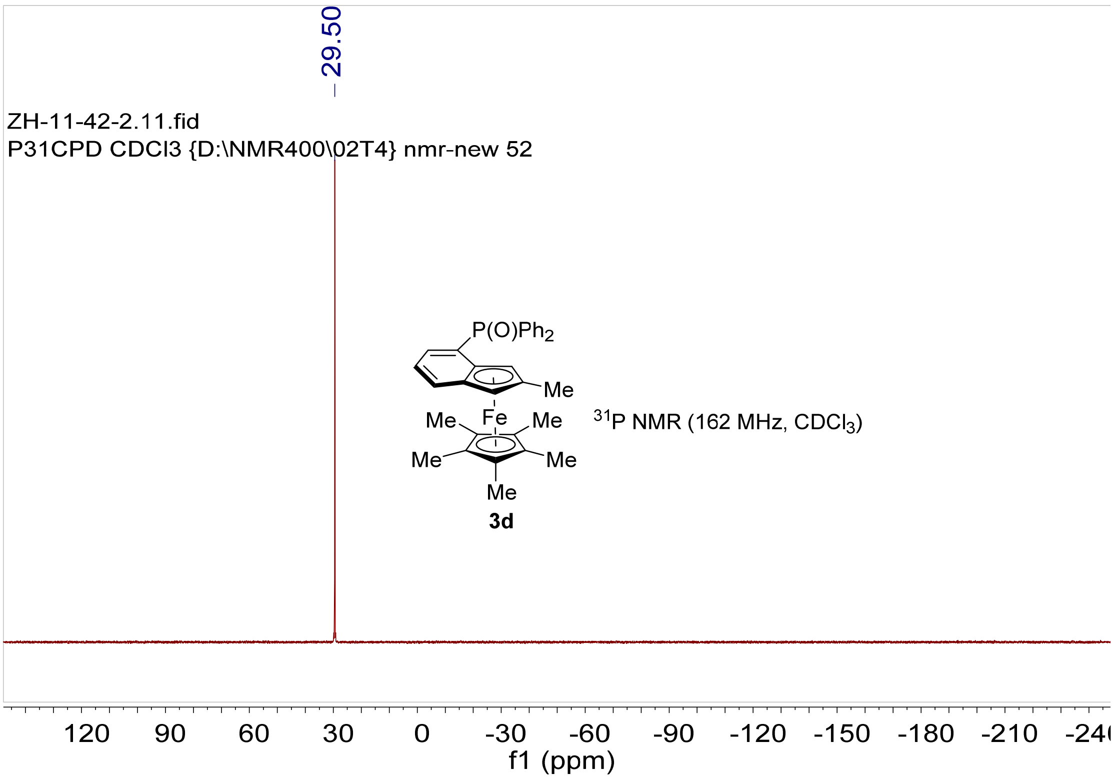

Supplementary Figure 41. ^{31}**P NMR spectra of compound 3d**

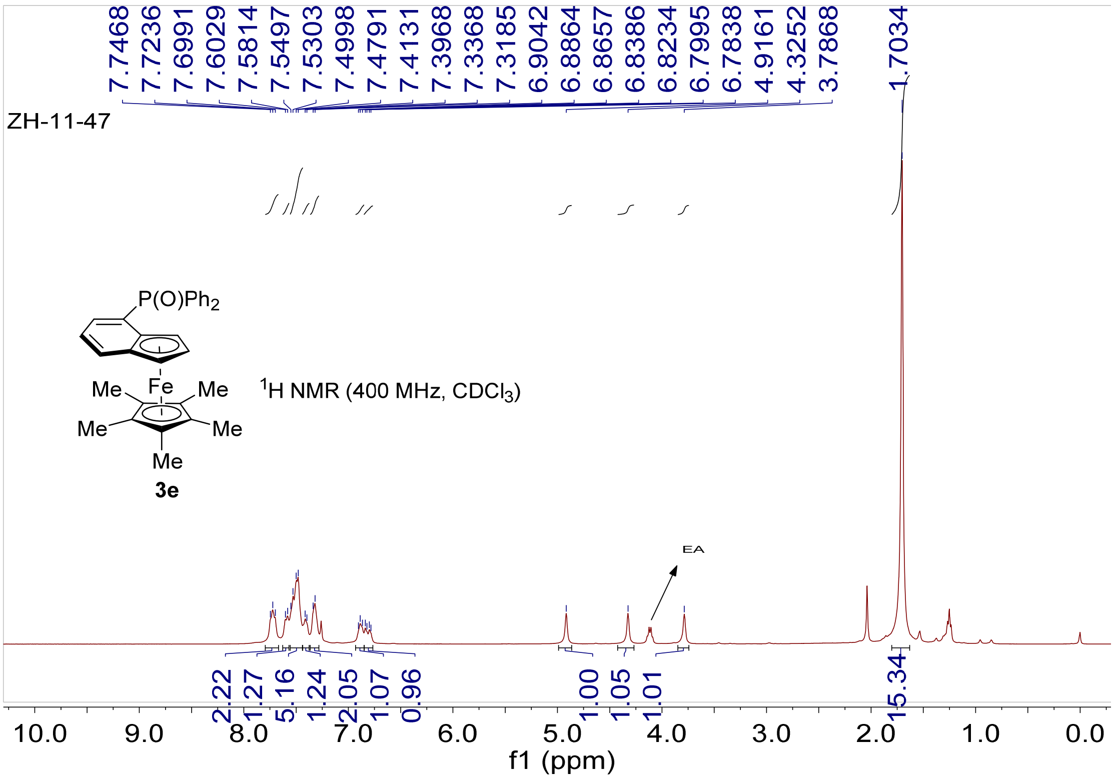

Supplementary Figure 42. ^{1}H NMR spectra of compound 3e

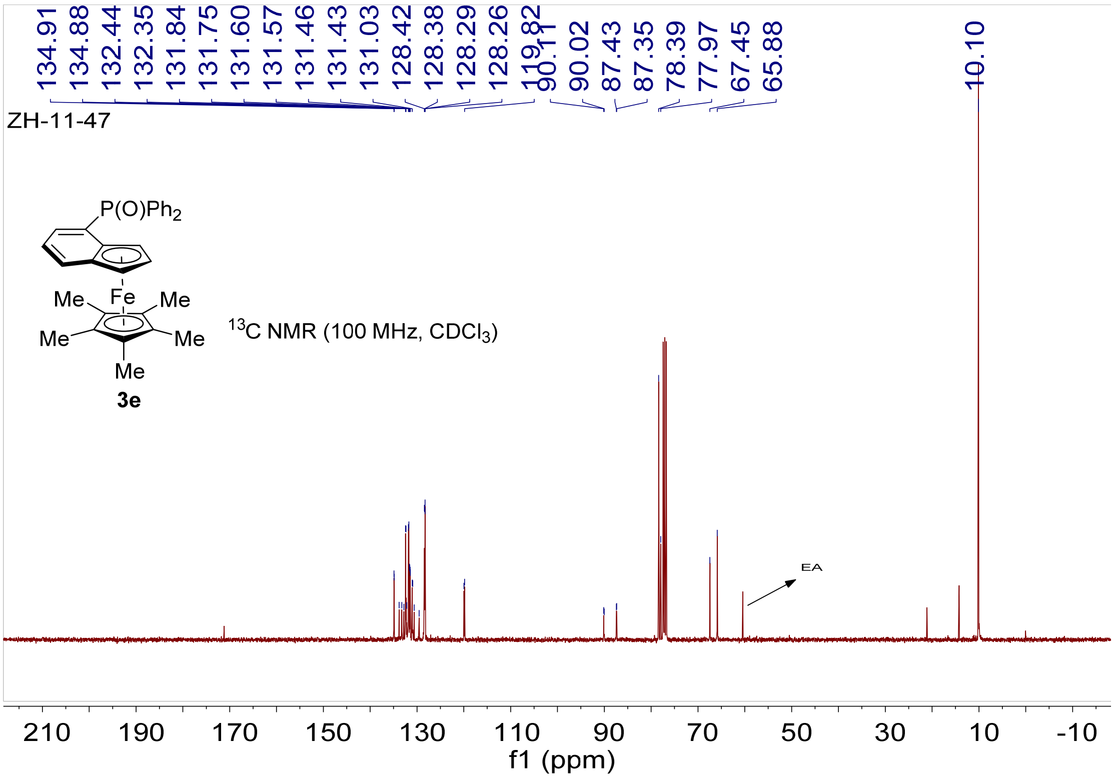

Supplementary Figure 43. ^{13}C NMR spectra of compound 3e

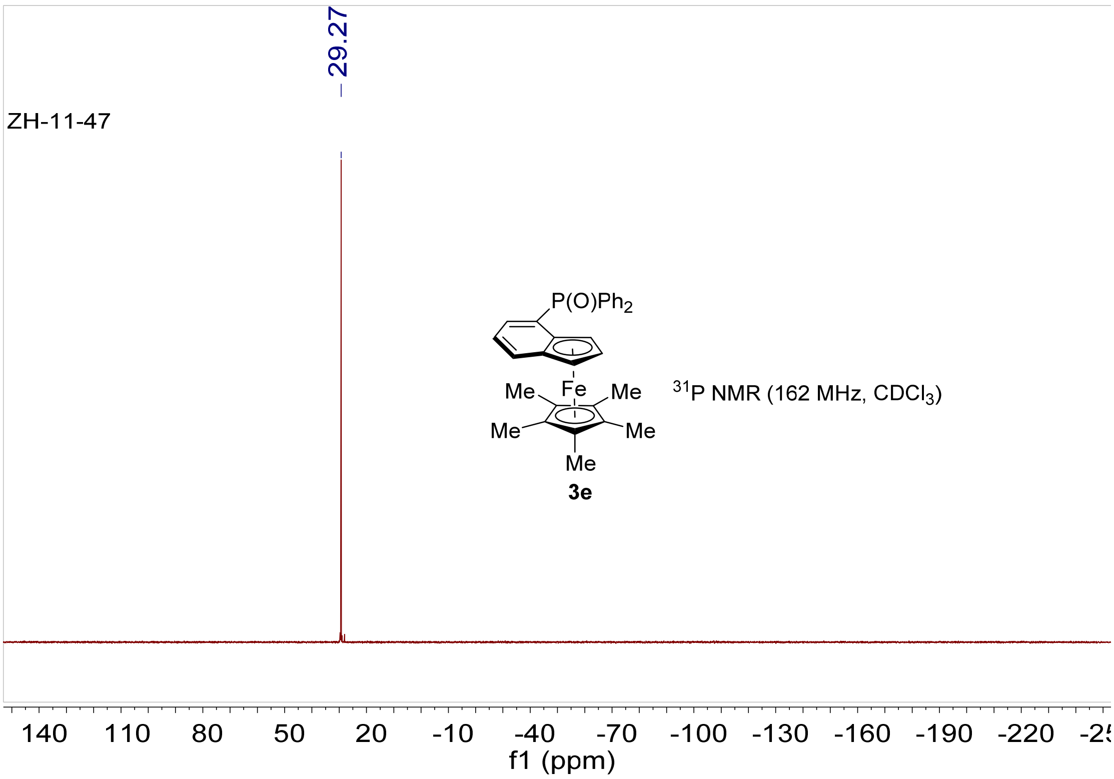

Supplementary Figure 44. ^{31}P NMR spectra of compound 3e

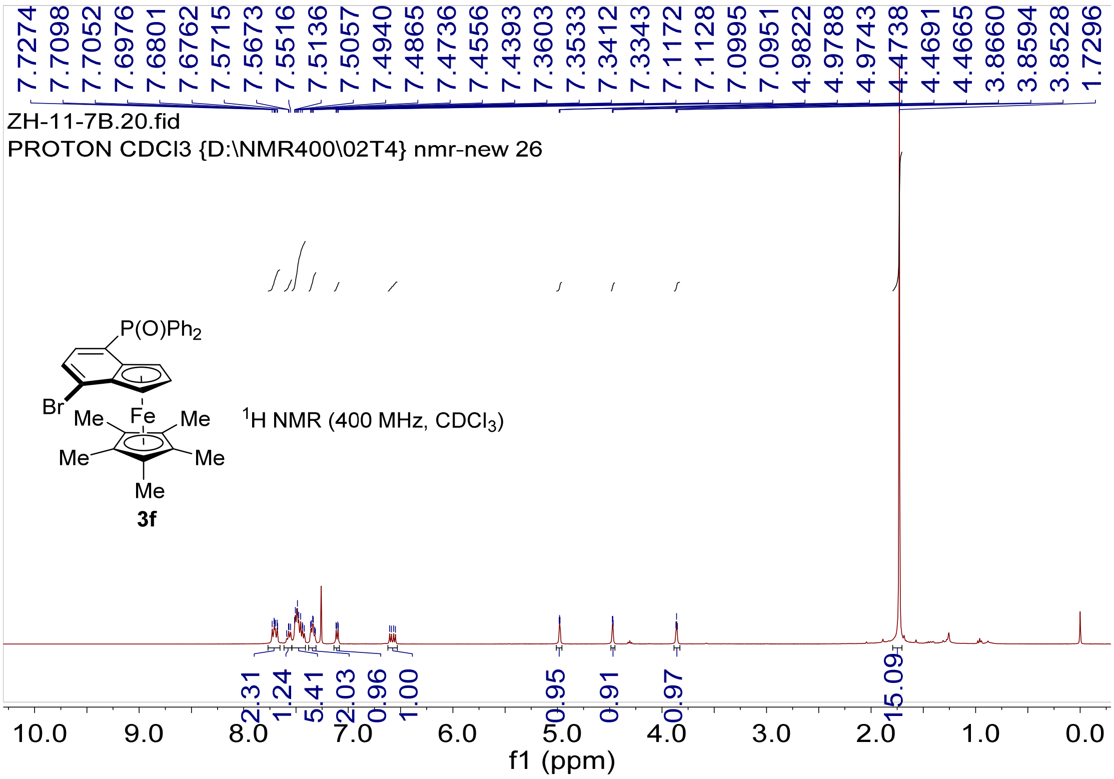

Supplementary Figure 45. ^{1}H NMR spectra of compound 3f

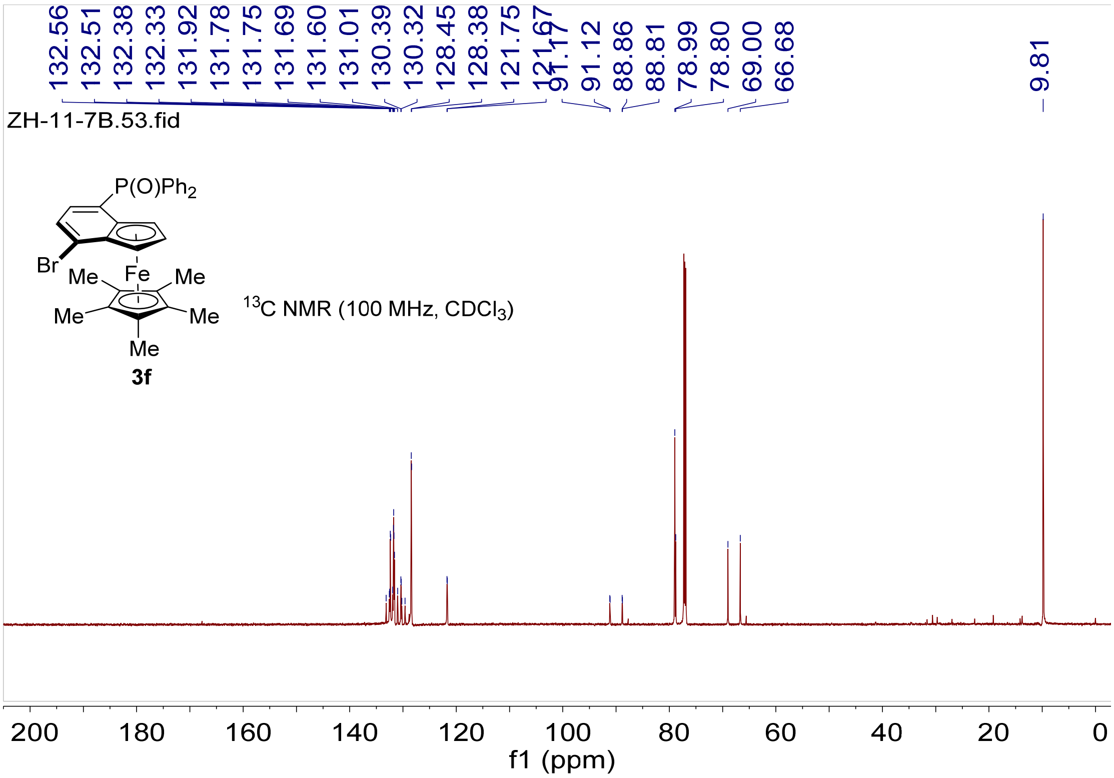

Supplementary Figure 46. ^{13}C NMR spectra of compound 3f

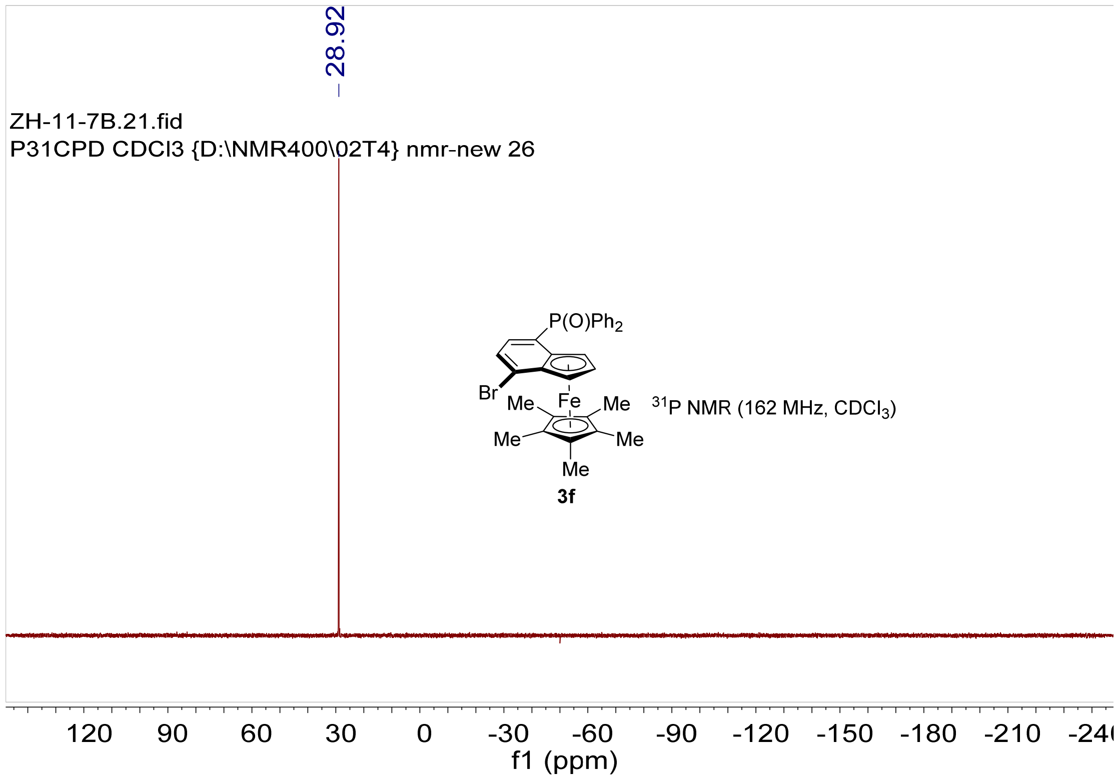

Supplementary Figure 47. ^{31}P NMR spectra of compound 3f

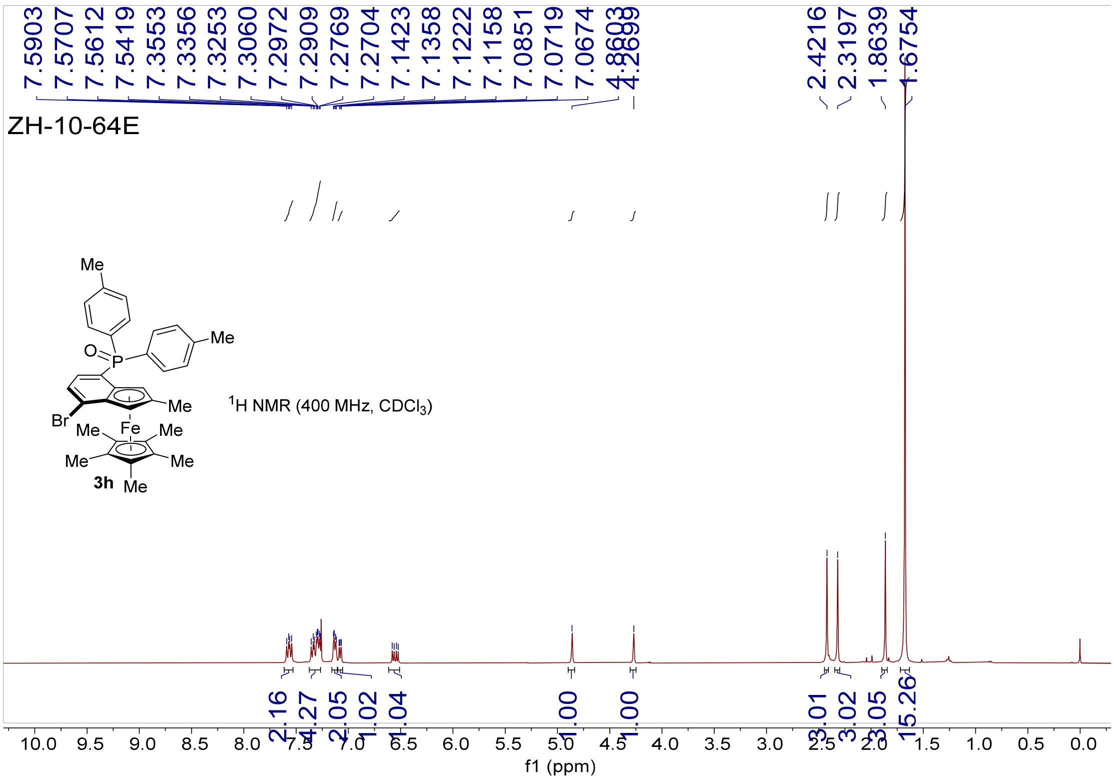

Supplementary Figure 48. ^{1}H NMR spectra of compound 3h

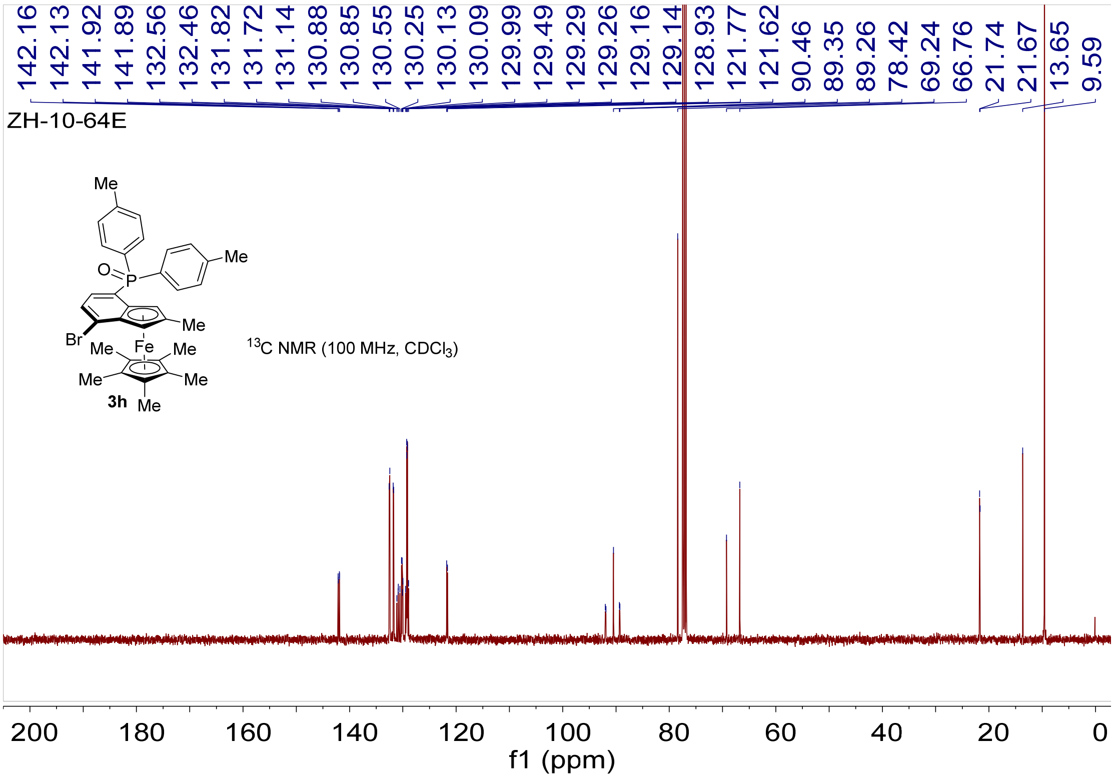

Supplementary Figure 49. ^{13}C NMR spectra of compound 3h

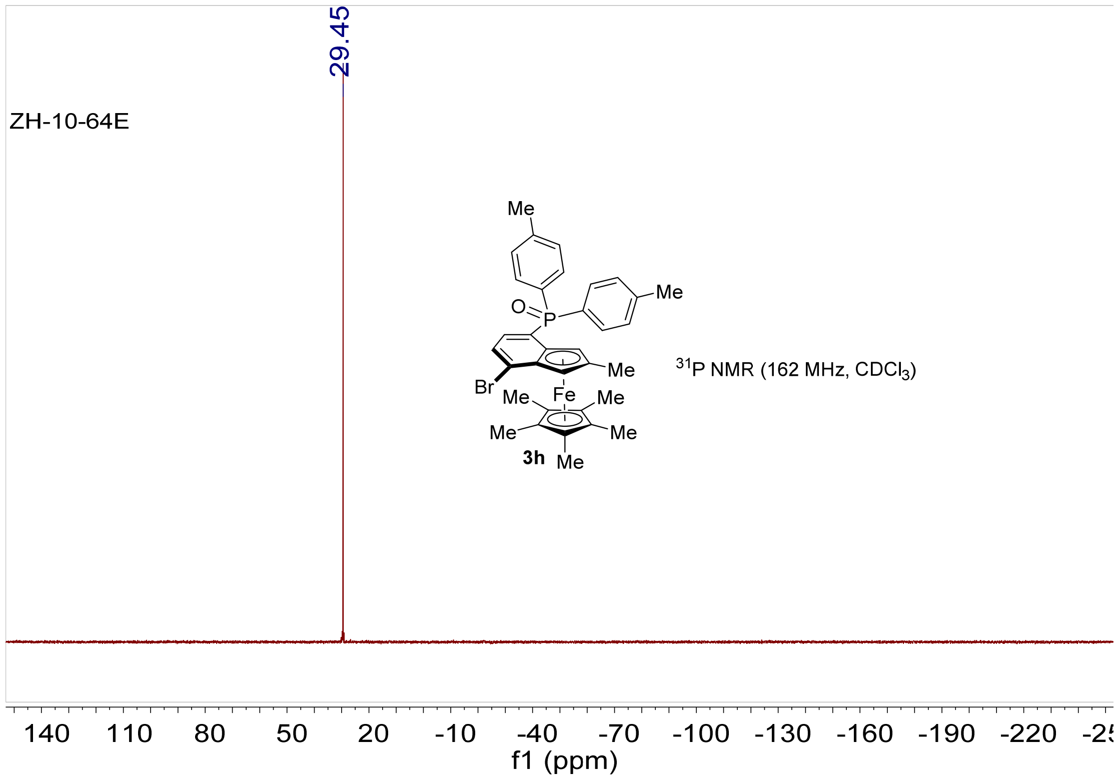

Supplementary Figure 50. ^{31}P NMR spectra of compound 3h

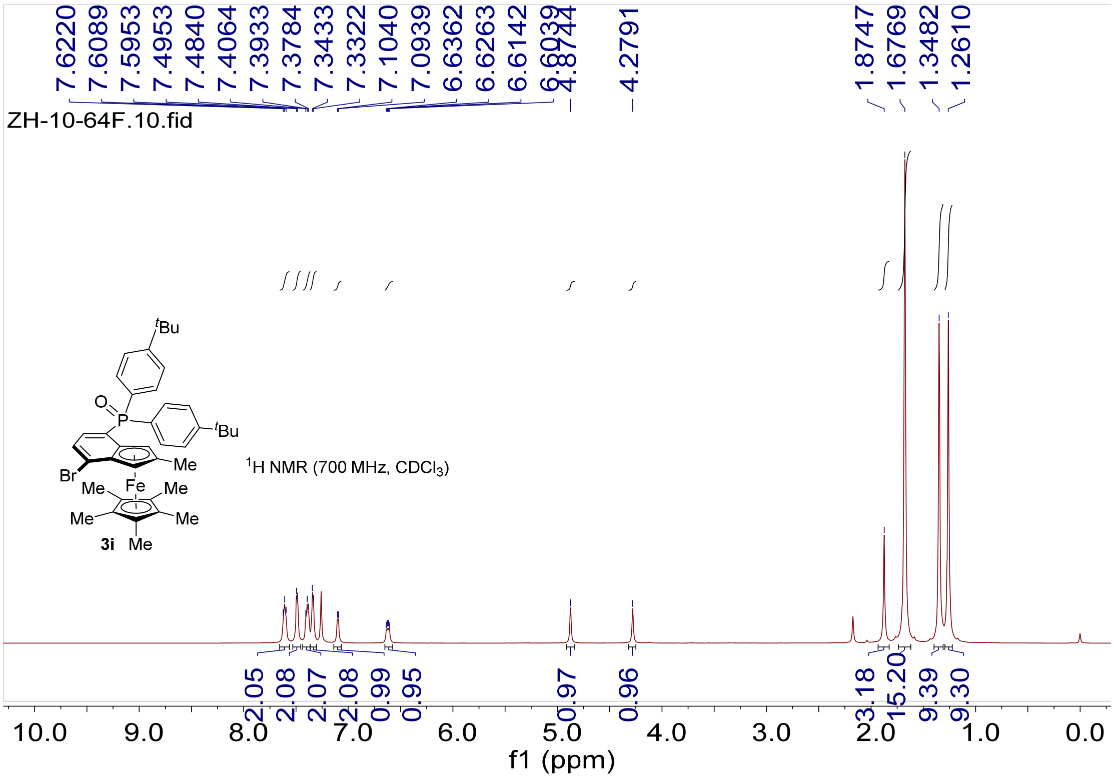

Supplementary Figure 51. ^{1}H NMR spectra of compound 3i

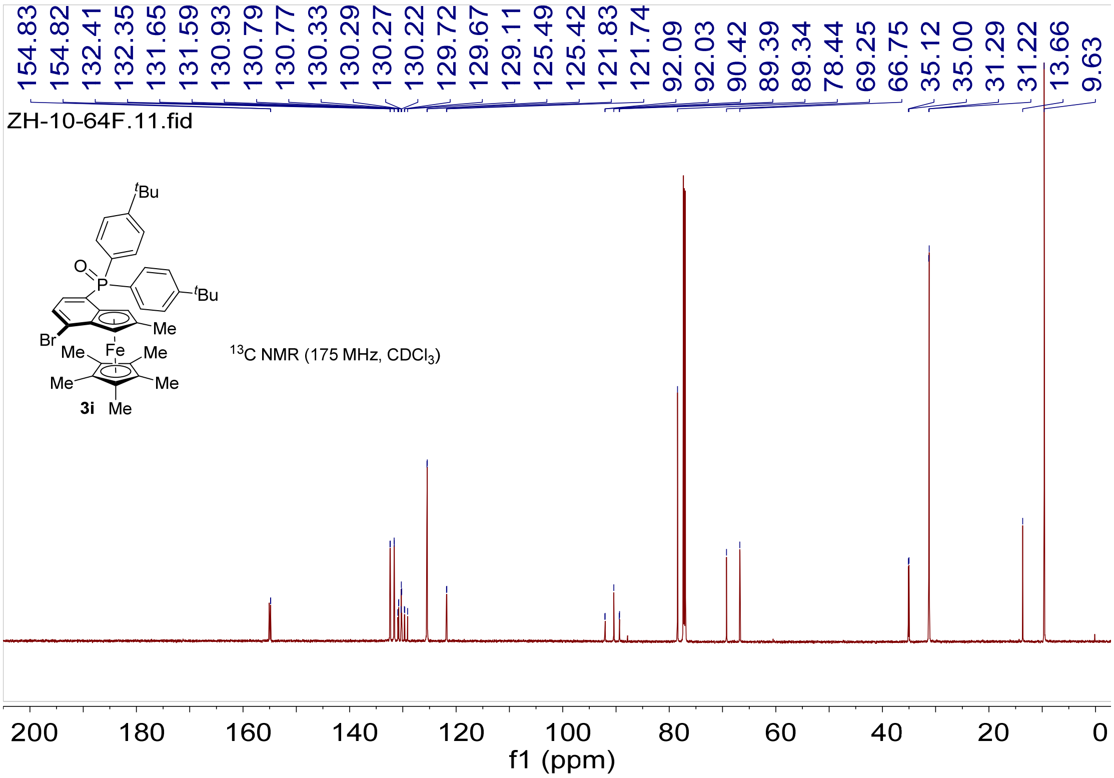

Supplementary Figure 52. ^{13}C NMR spectra of compound 3i

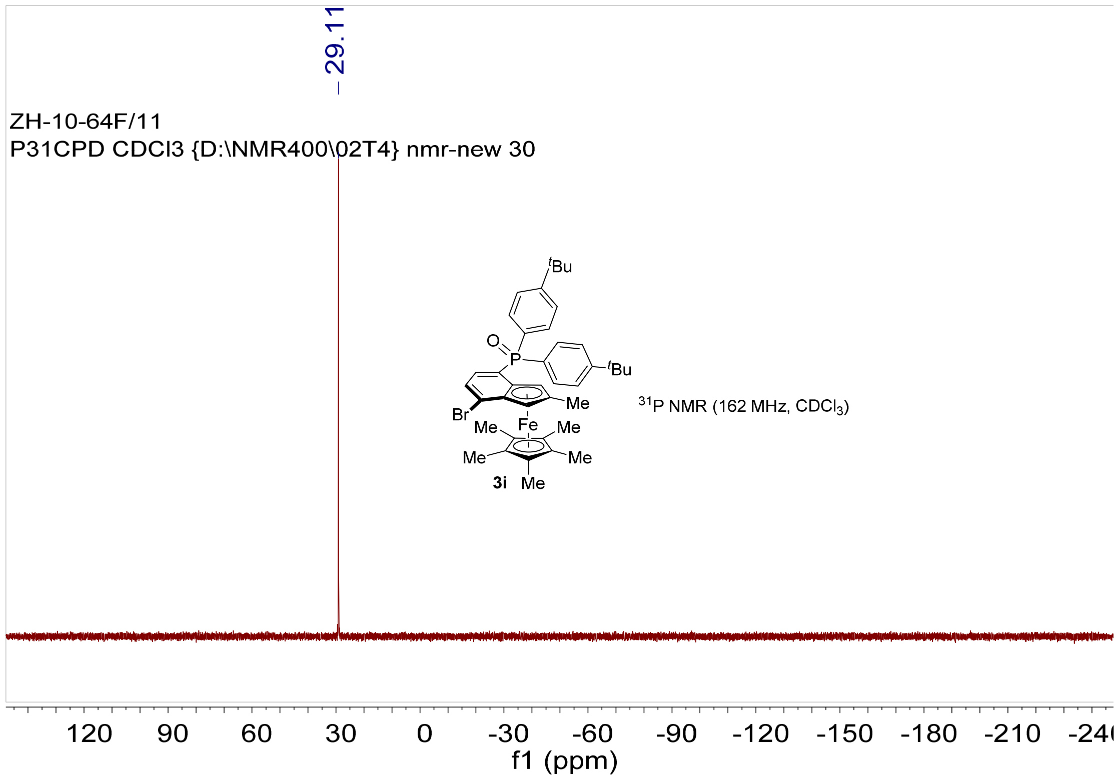

Supplementary Figure 53. ^{31}P NMR spectra of compound 3i

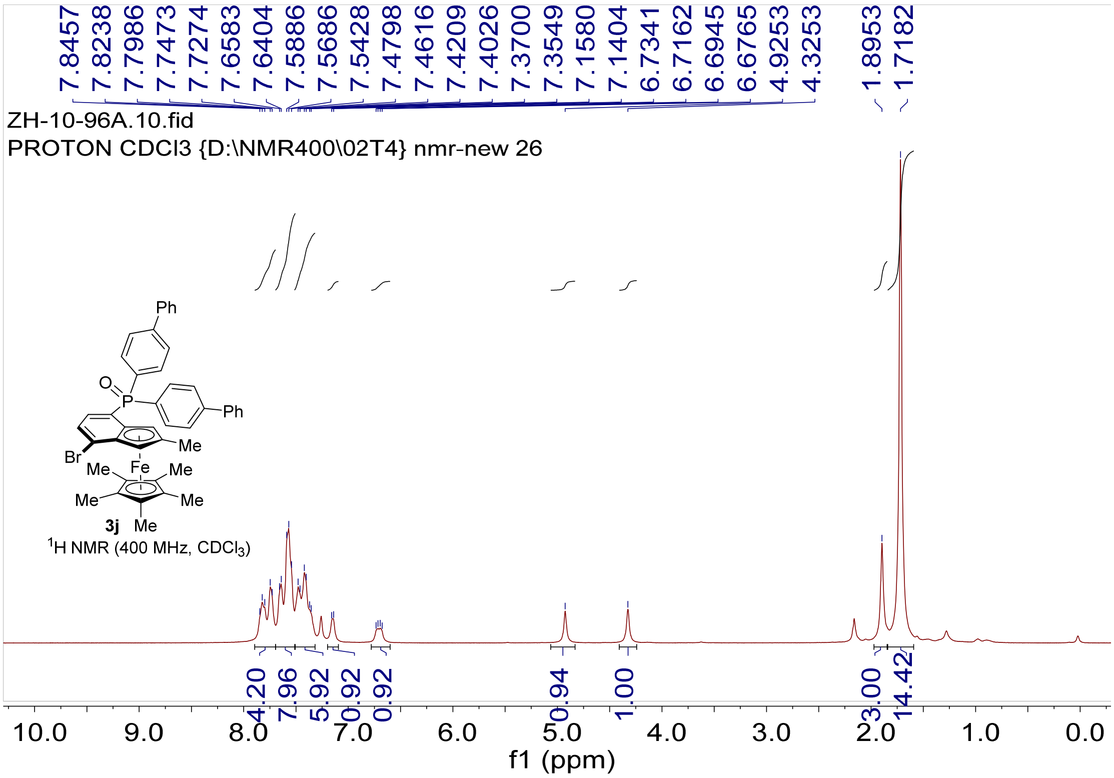

Supplementary Figure 54. ^{1}H NMR spectra of compound 3j

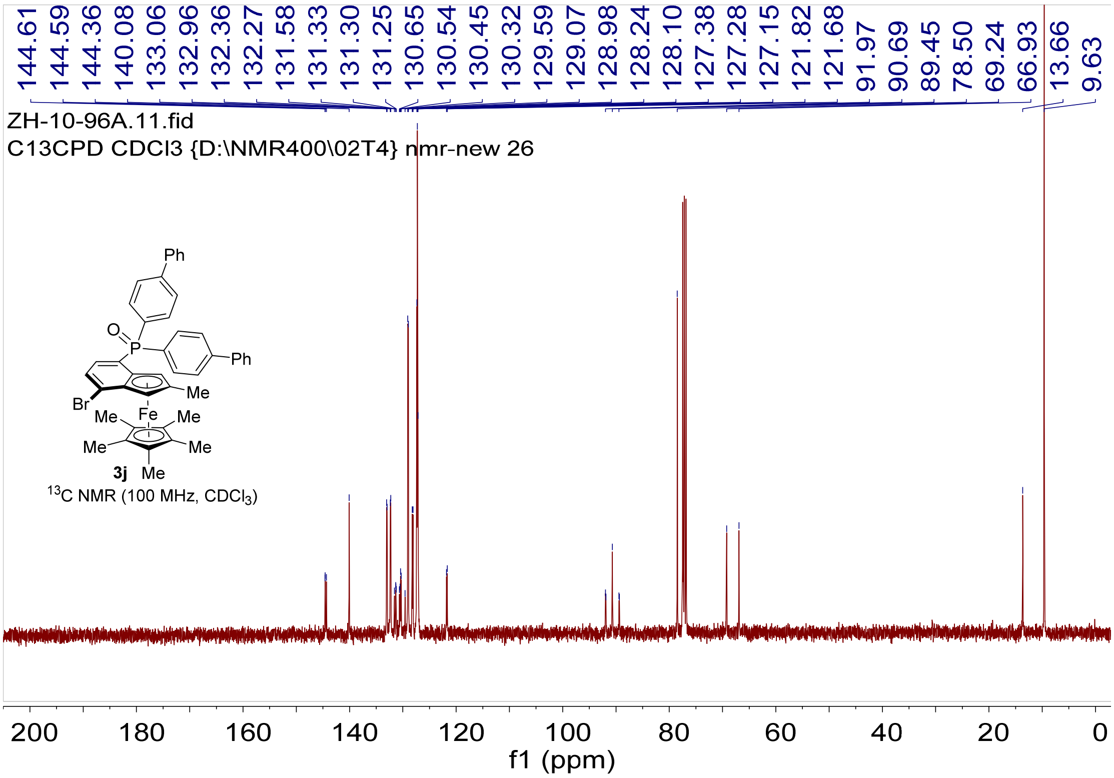

Supplementary Figure 55. ^{13}C NMR spectra of compound 3j

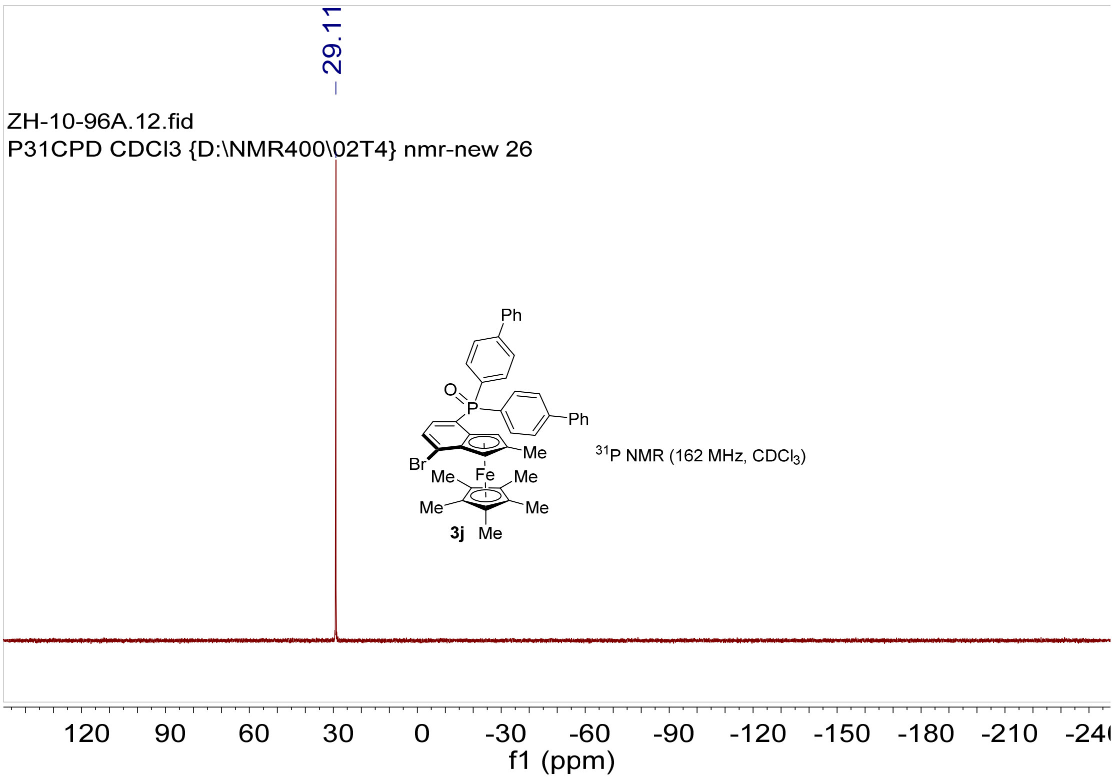

Supplementary Figure 56. ^{31}P NMR spectra of compound 3j

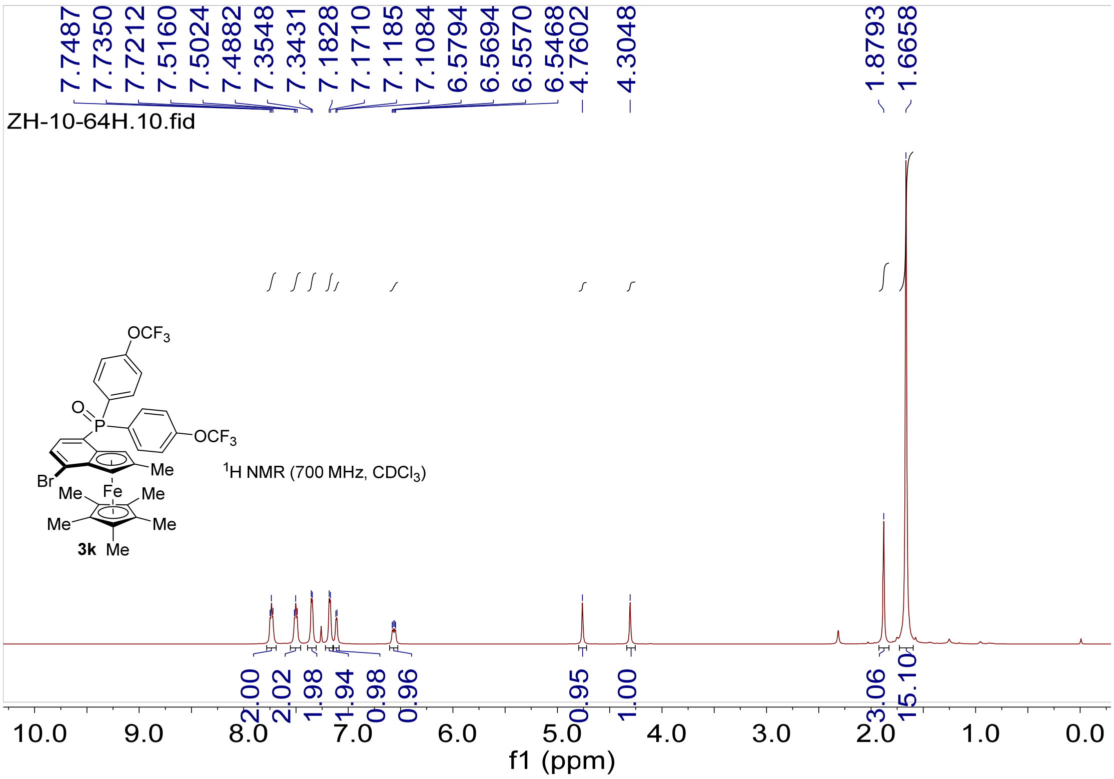

Supplementary Figure 57. ^{1}H NMR spectra of compound 3k

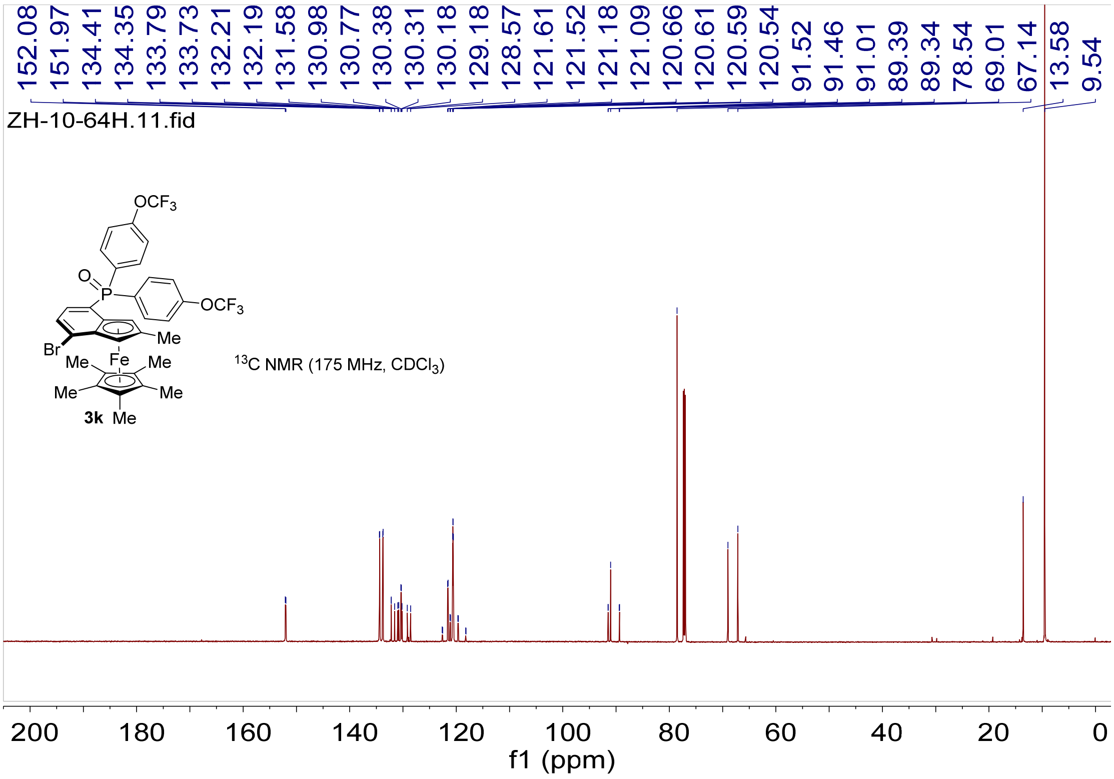

Supplementary Figure 58. ^{13}C NMR spectra of compound 3k

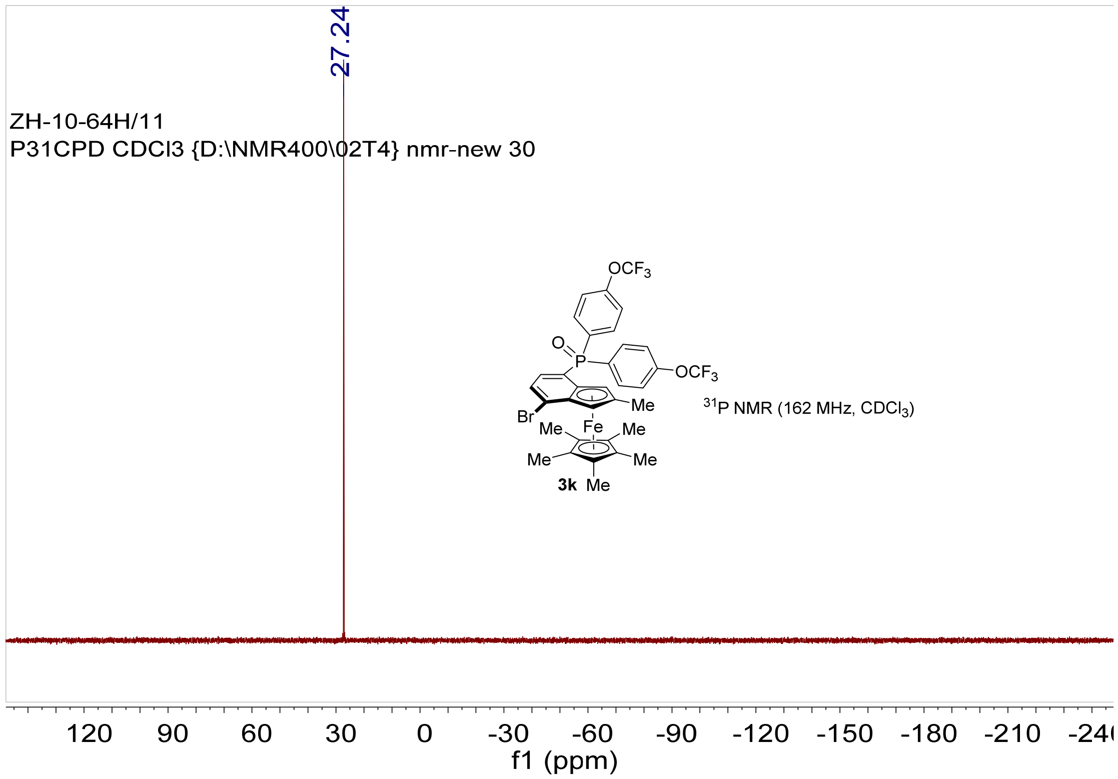

Supplementary Figure 59. ^{31}**P NMR spectra of compound 3k**

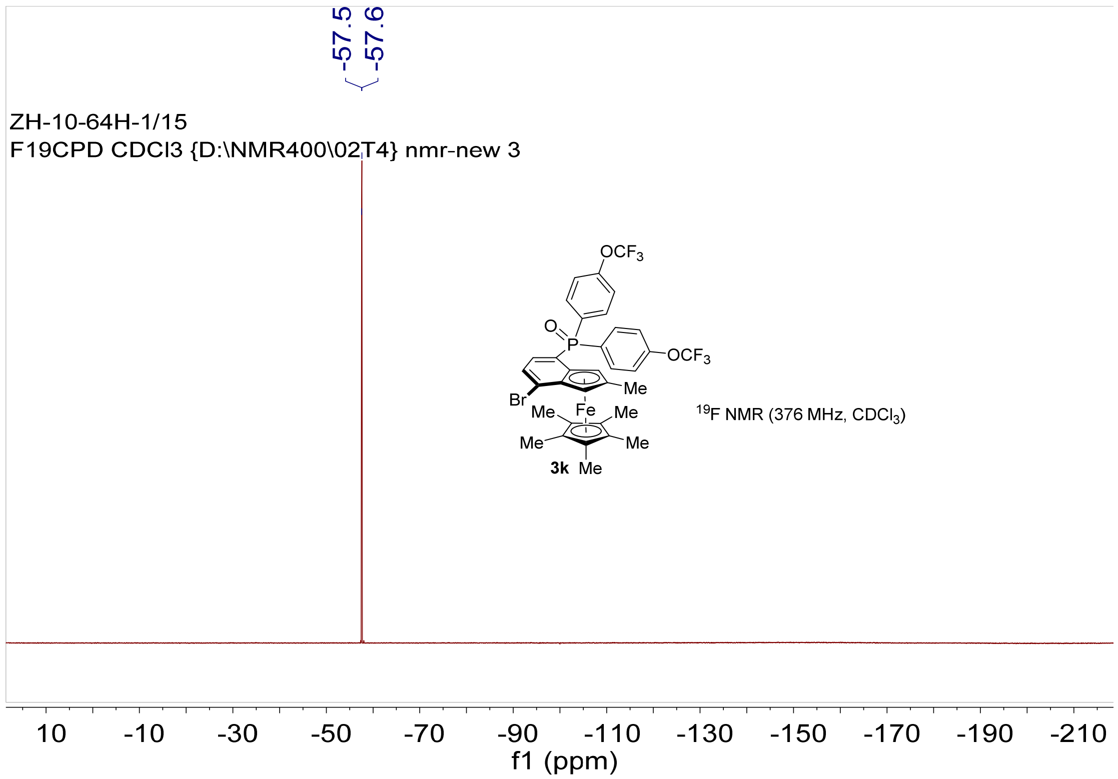

Supplementary Figure 60. ^{19}**F NMR spectra of compound 3k**

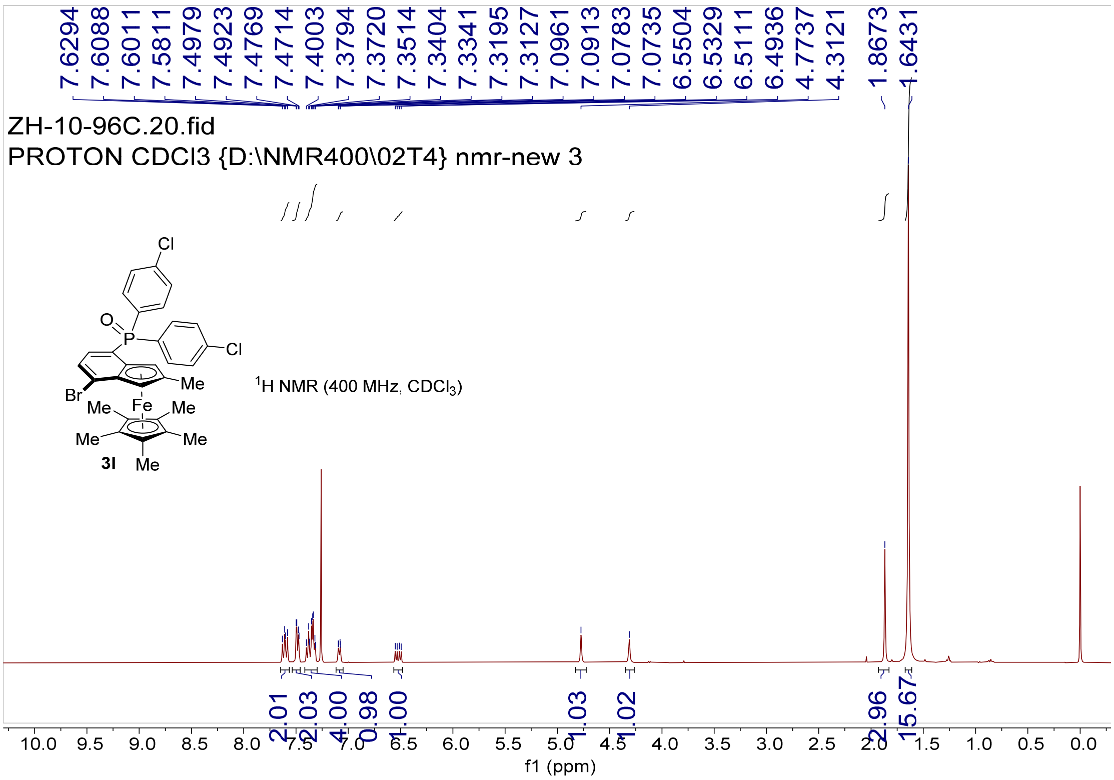

Supplementary Figure 61. ^{1}H NMR spectra of compound 3l

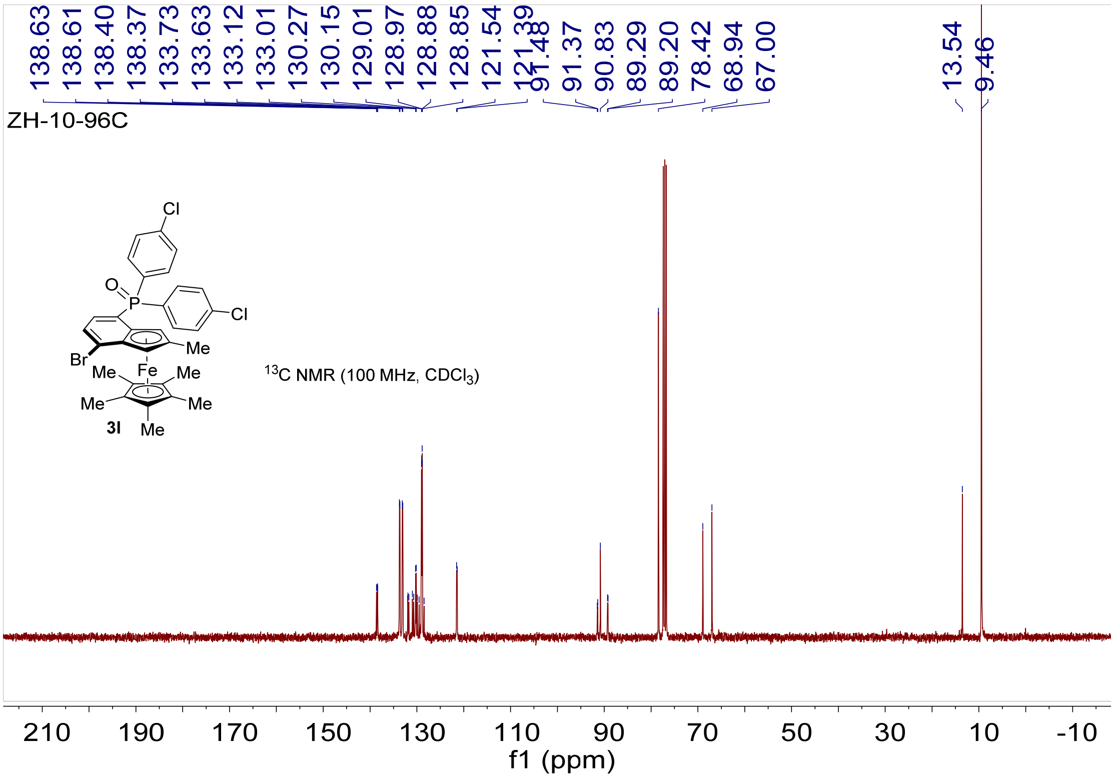

Supplementary Figure 62. ^{13}C NMR spectra of compound 3l

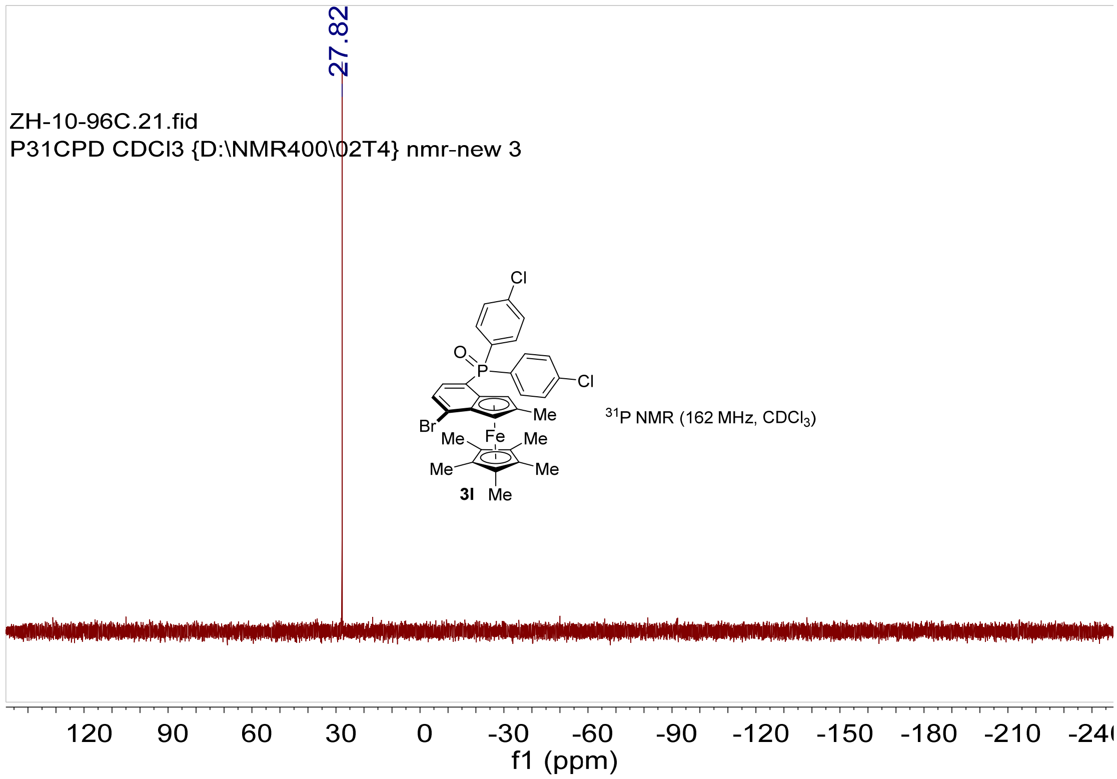

Supplementary Figure 63. ^{31}**P NMR spectra of compound 3l**

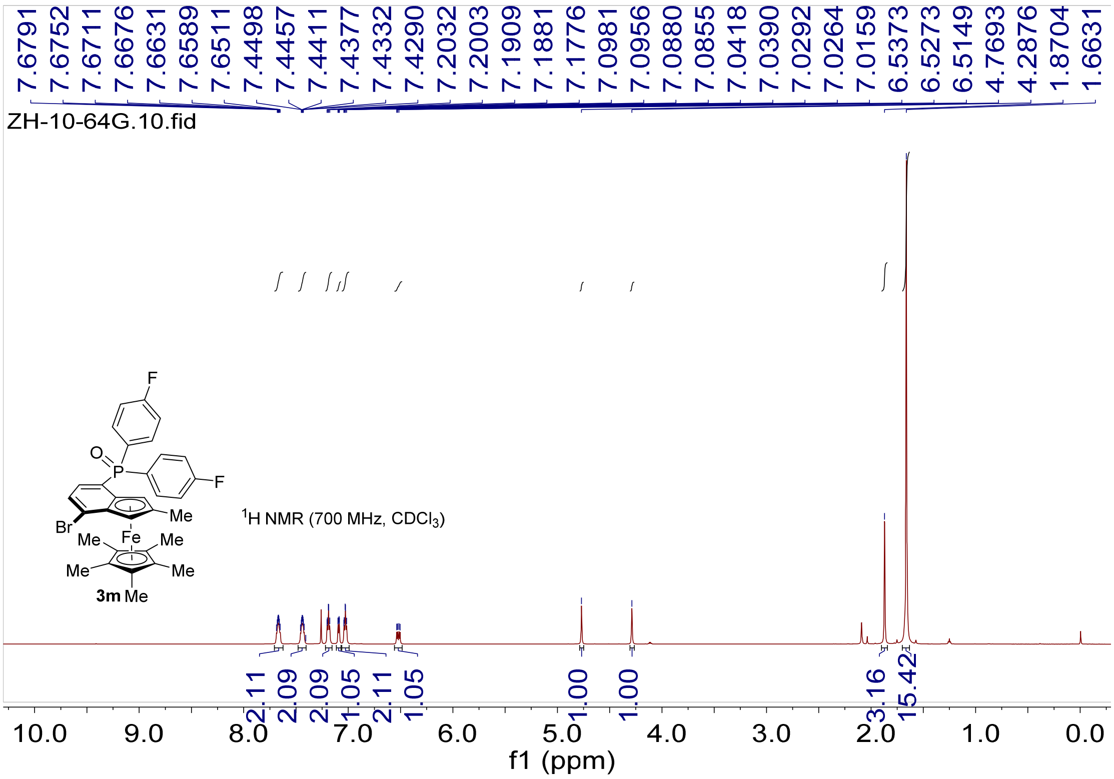

Supplementary Figure 64. ^{1}**H NMR spectra of compound 3m**

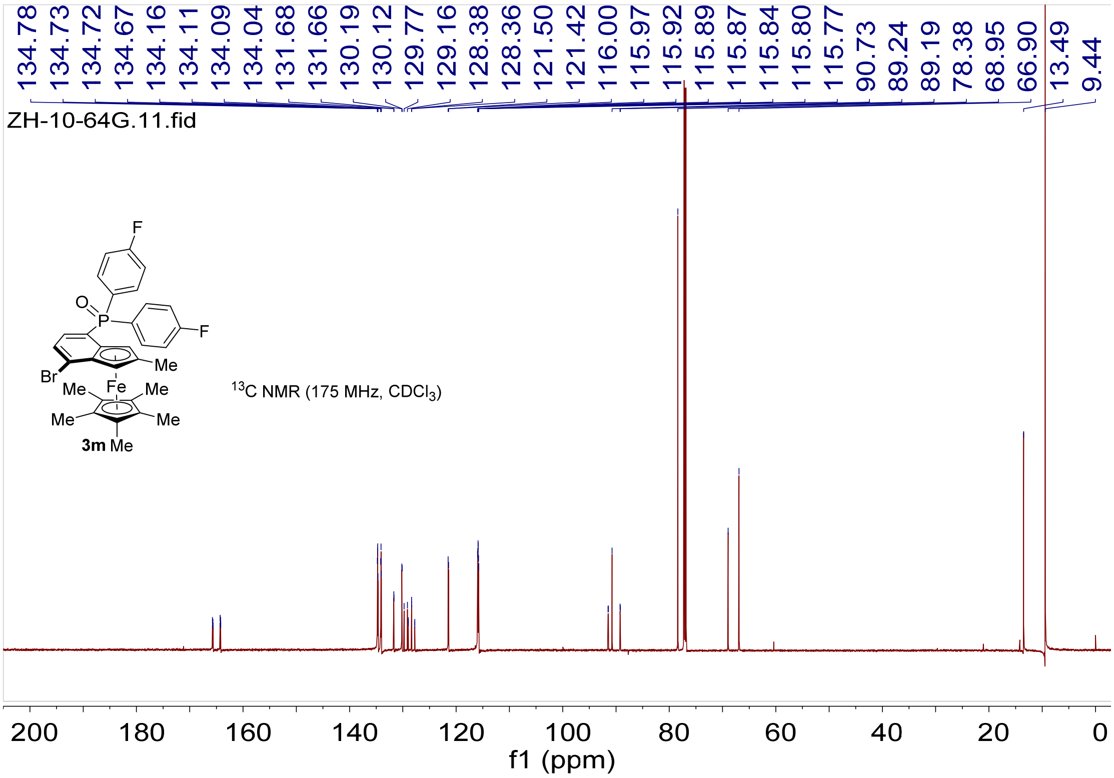

Supplementary Figure 65. ^{13}**C NMR spectra of compound 3m**

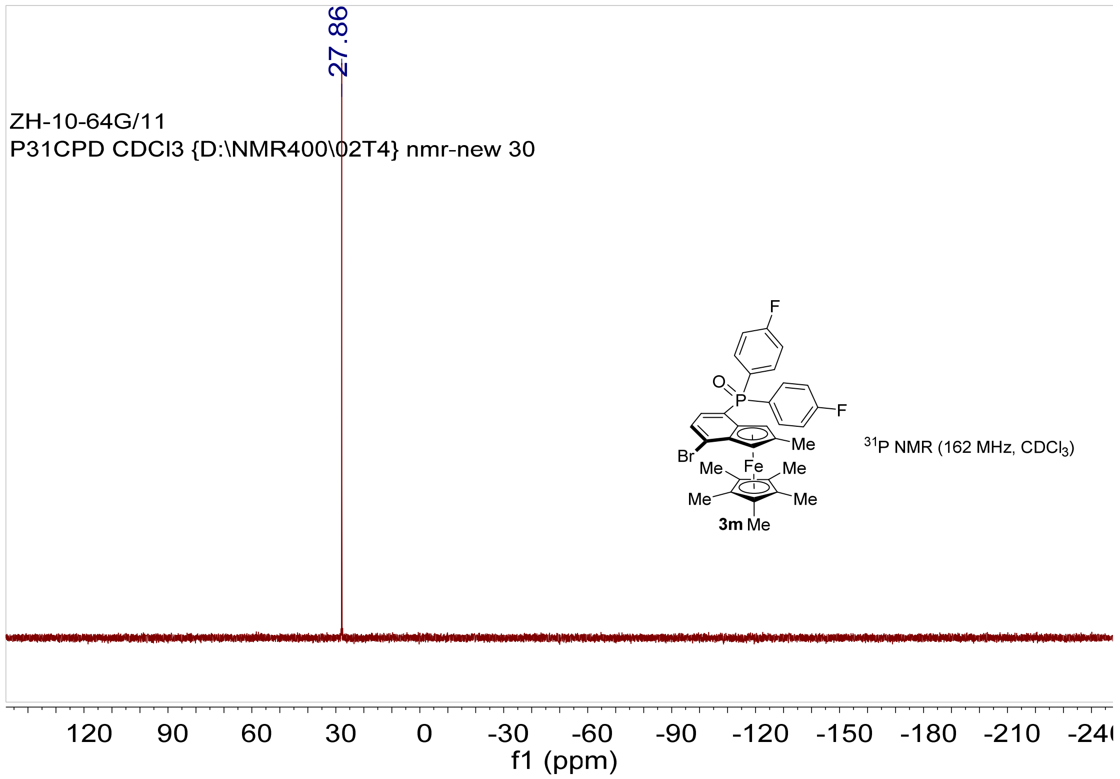

Supplementary Figure 66. ^{31}**P NMR spectra of compound 3m**

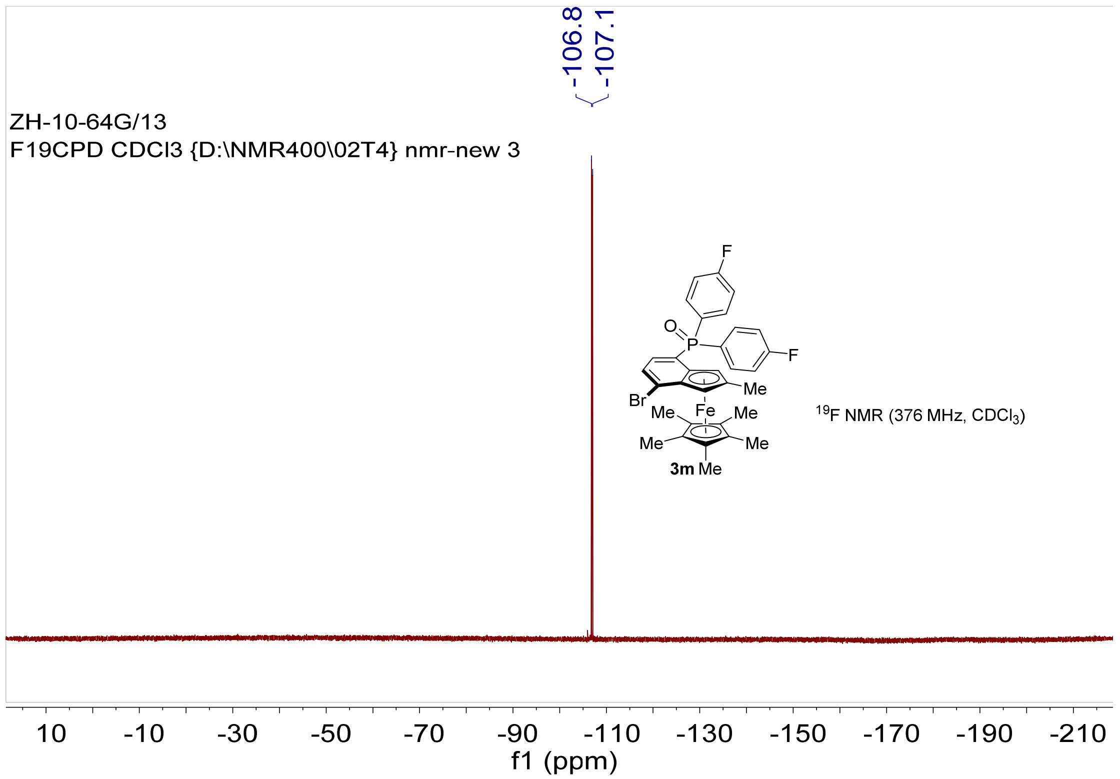

Supplementary Figure 67. ^{19}**F NMR spectra of compound 3m**

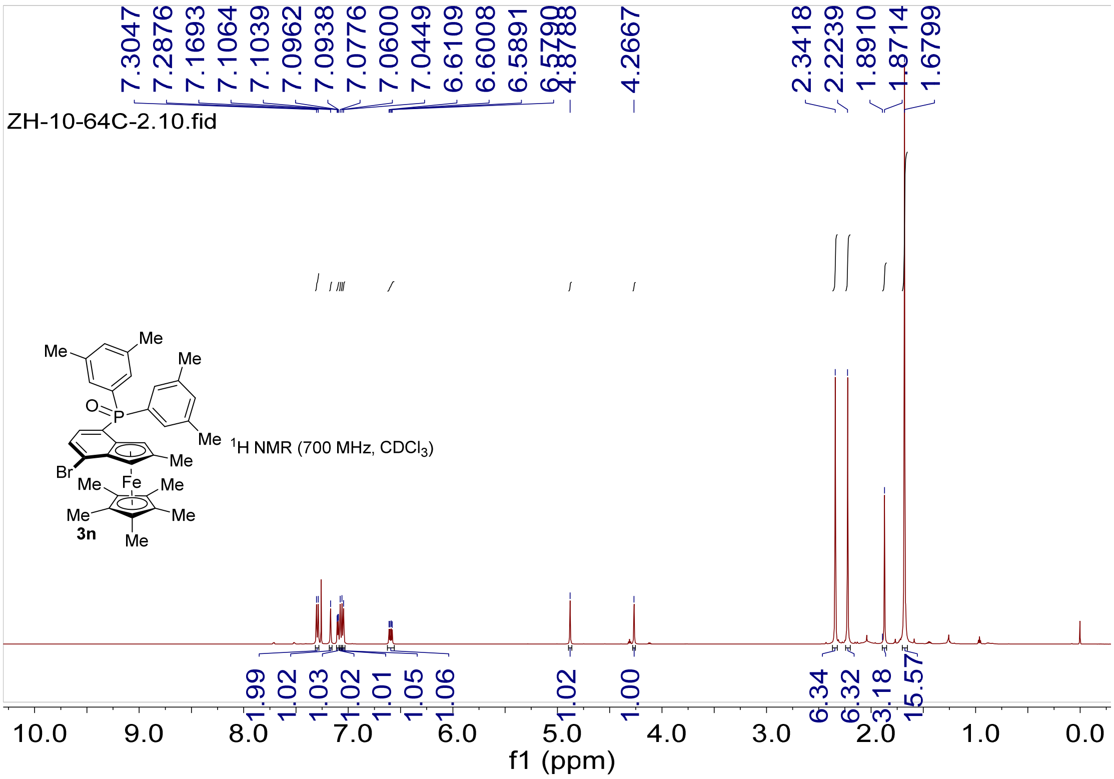

Supplementary Figure 68. ^{1}H NMR spectra of compound 3n

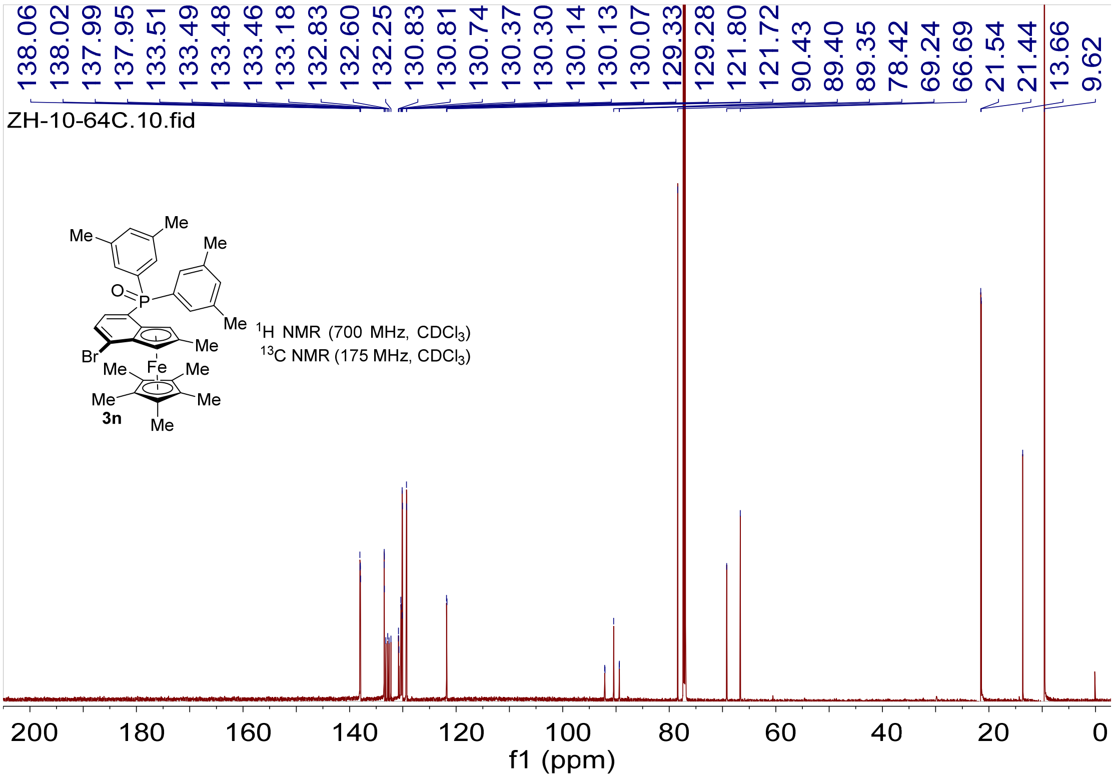

Supplementary Figure 69. ^{13}C NMR spectra of compound 3n

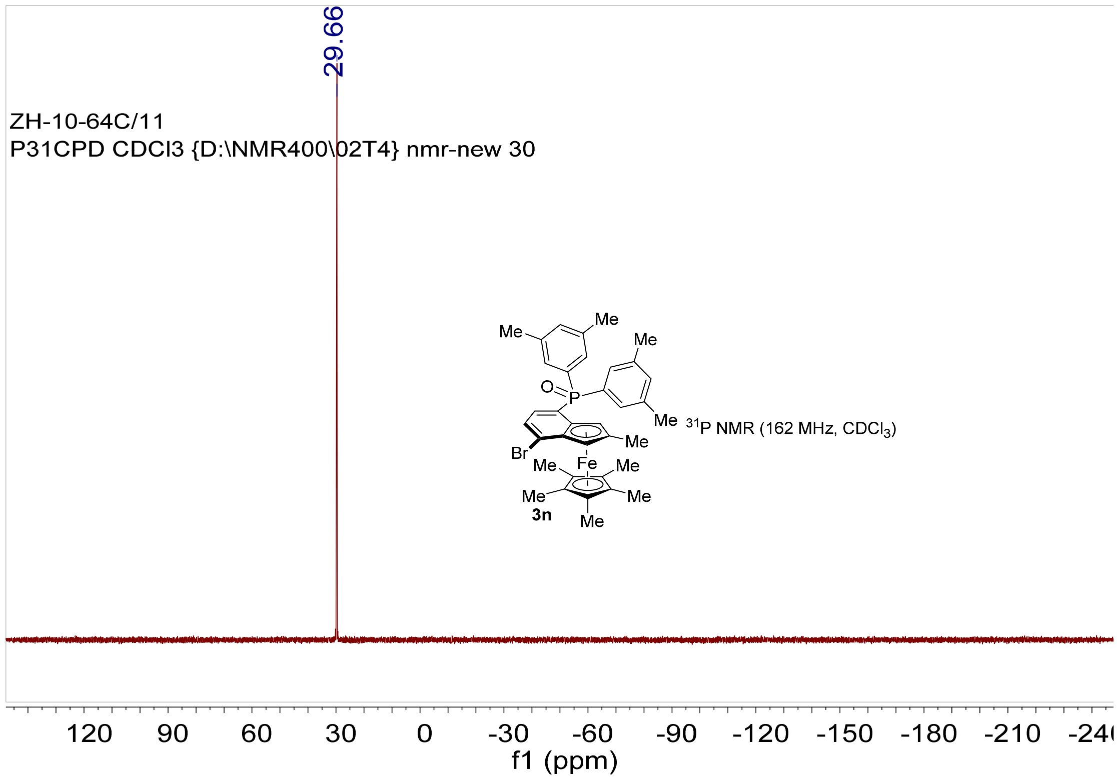

Supplementary Figure 70. ^{31}P NMR spectra of compound 3n

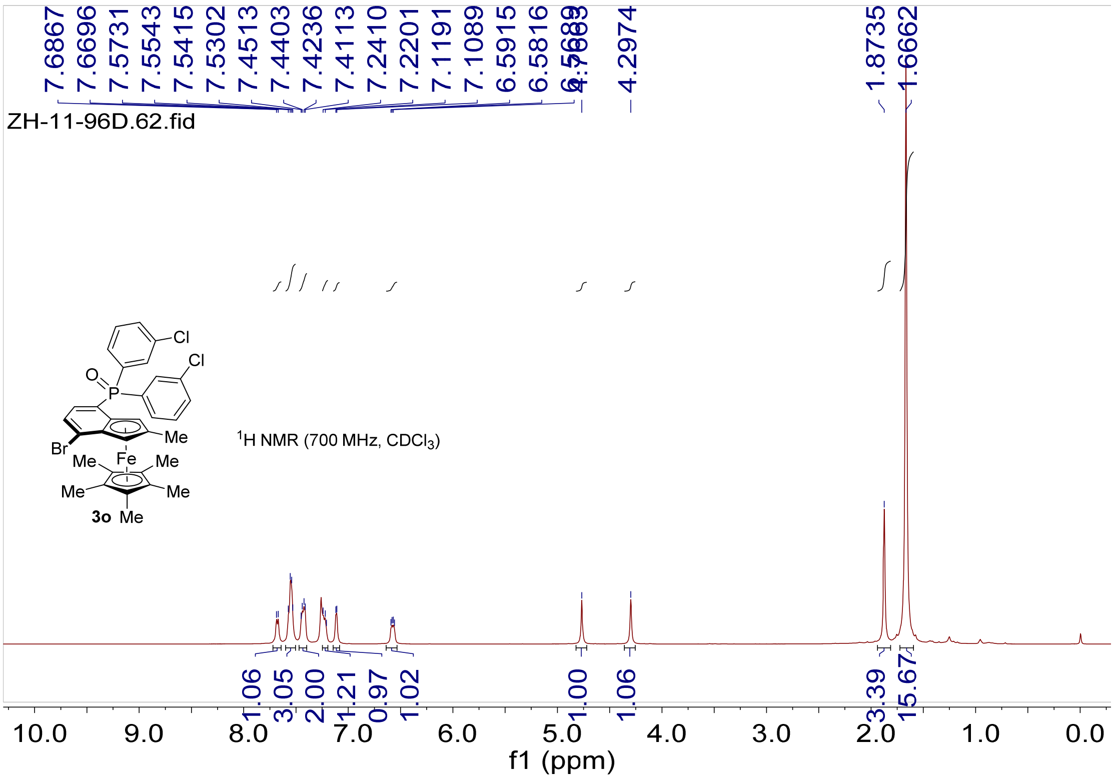

Supplementary Figure 71. ^{1}H NMR spectra of compound 3o

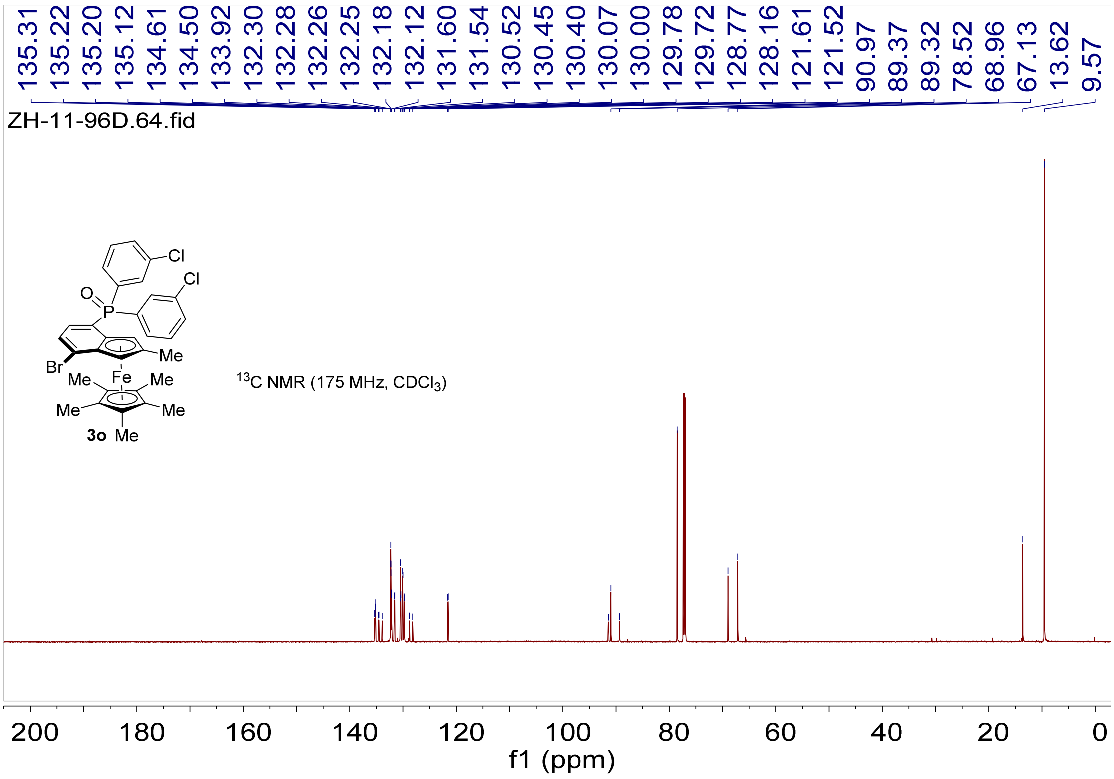

Supplementary Figure 72. ^{13}C NMR spectra of compound 3o

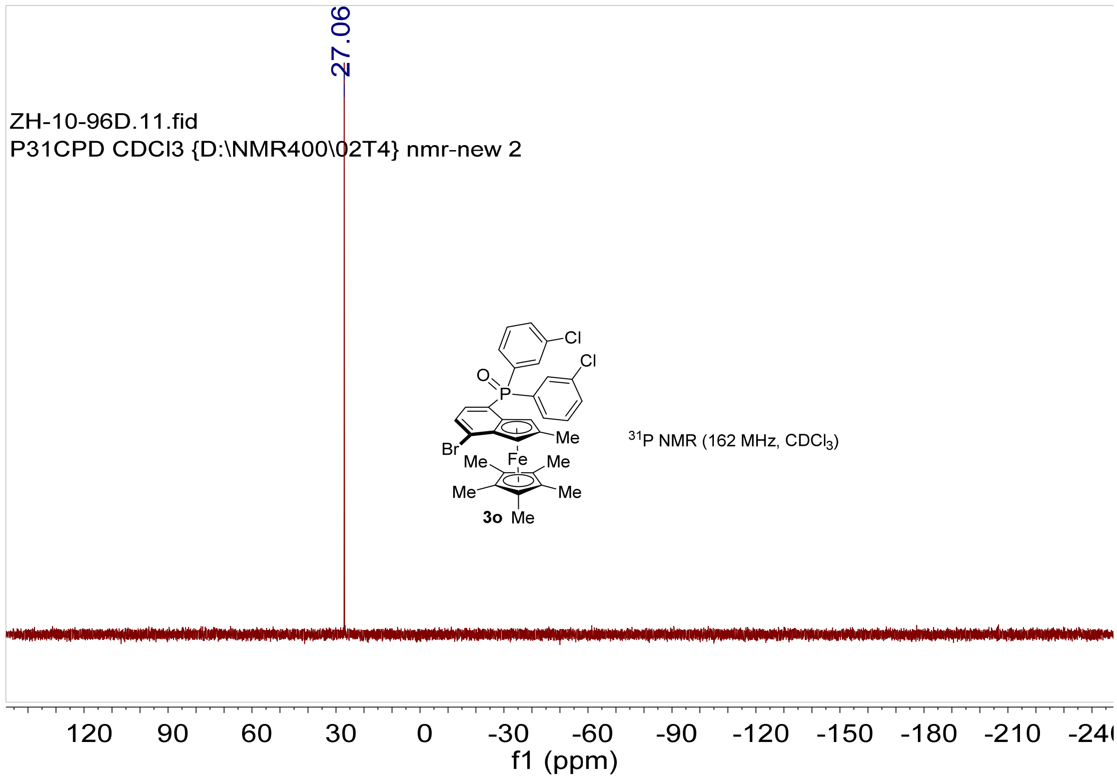

Supplementary Figure 73. ^{31}P NMR spectra of compound 3o

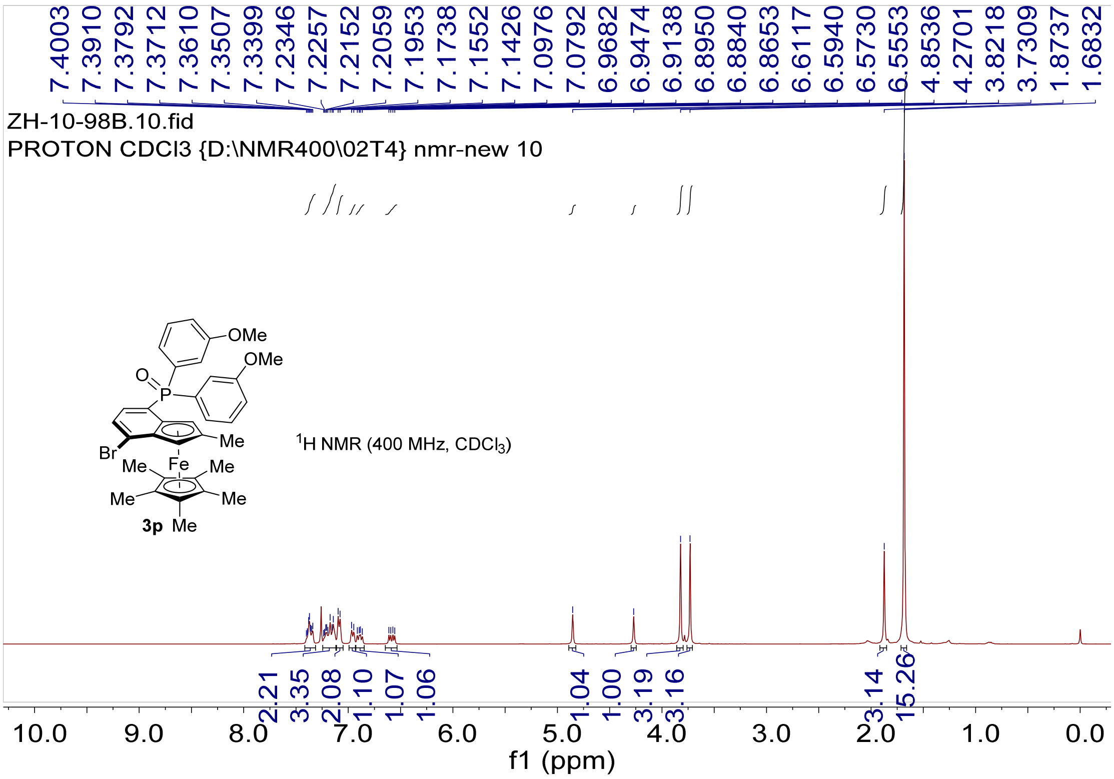

Supplementary Figure 74. ^{1}H NMR spectra of compound 3p

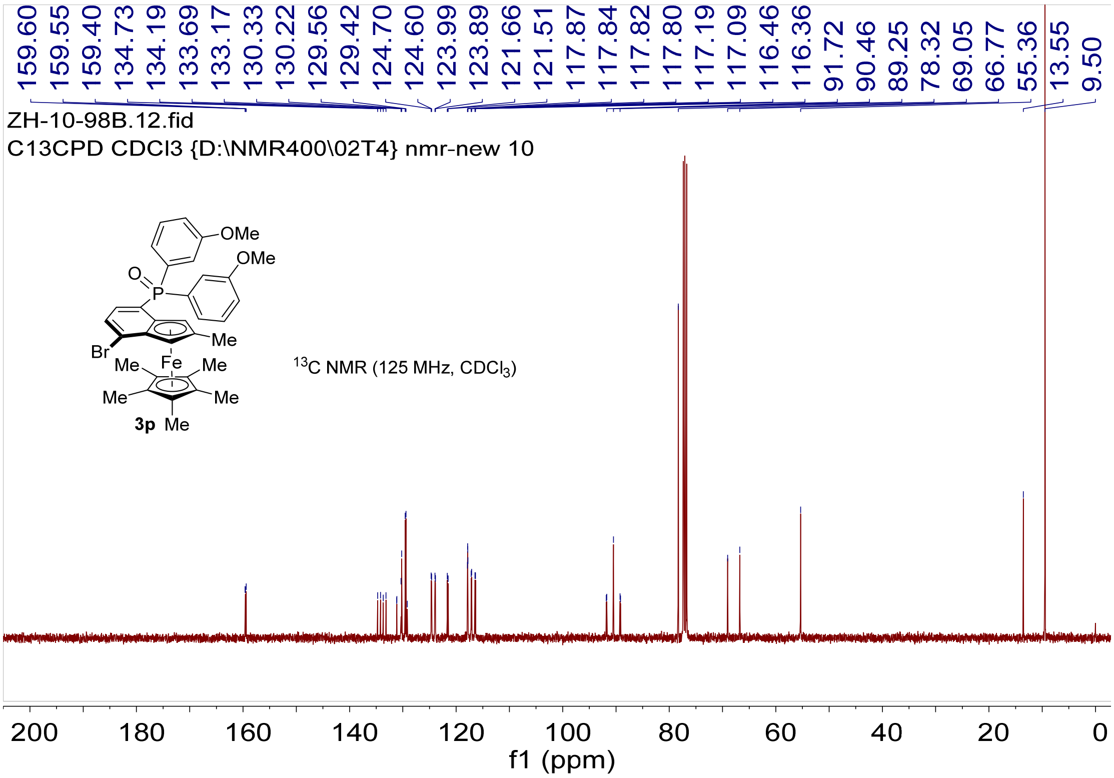

Supplementary Figure 75. ^{13}C NMR spectra of compound 3p

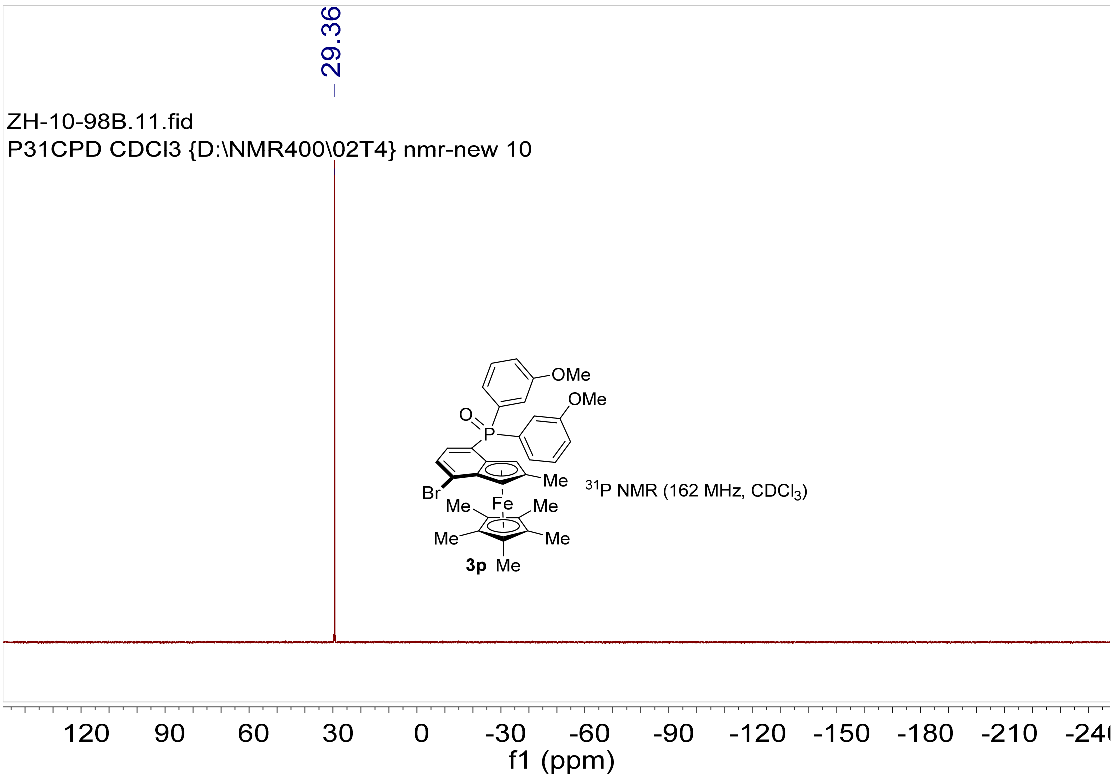

Supplementary Figure 76. ^{31}P NMR spectra of compound 3p

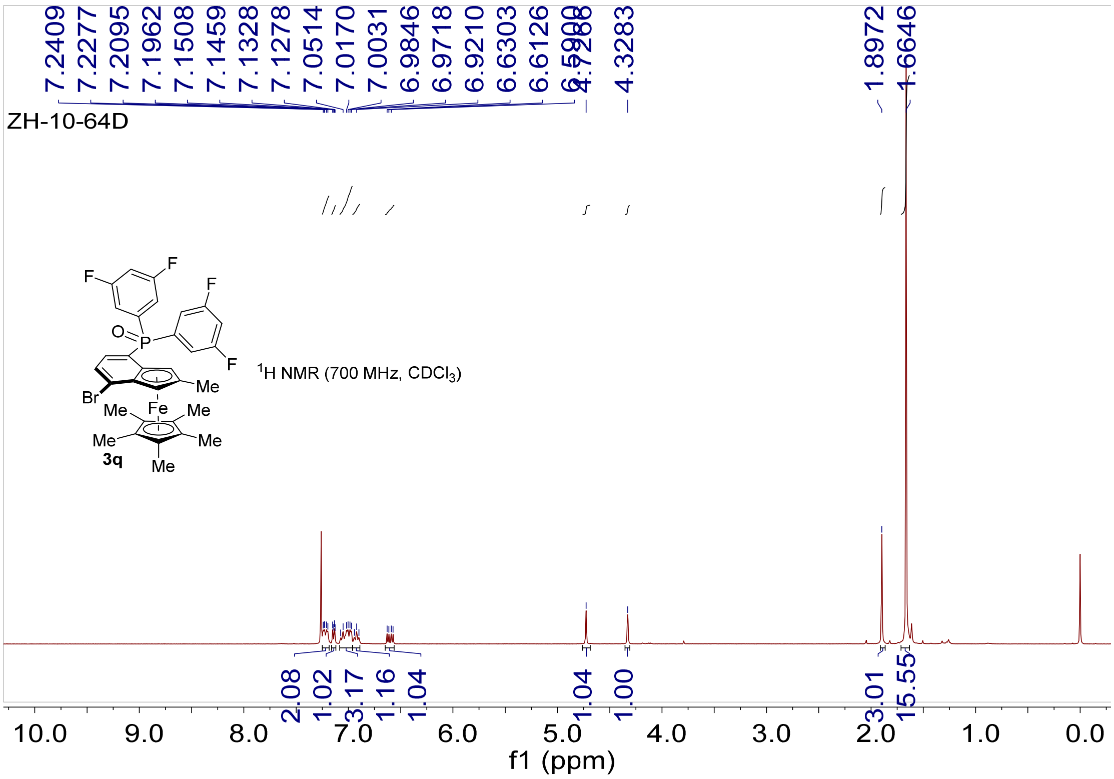

Supplementary Figure 77. ^{1}H NMR spectra of compound 3q

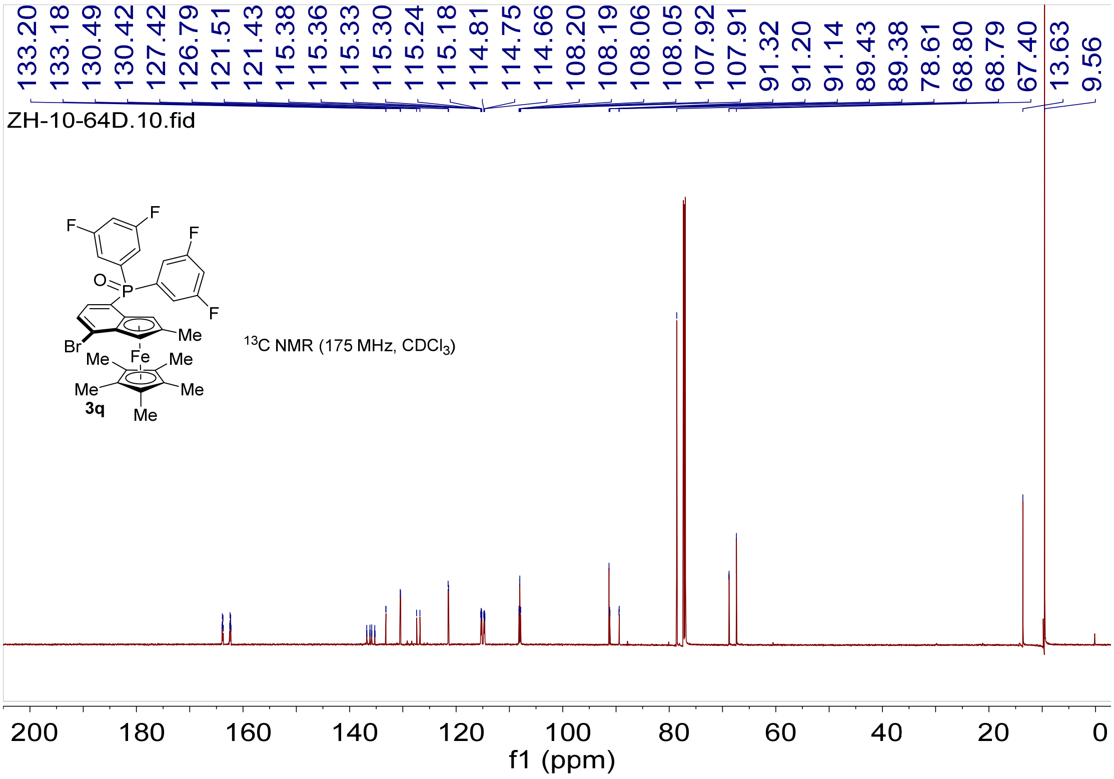

Supplementary Figure 78. ^{13}C NMR spectra of compound 3q

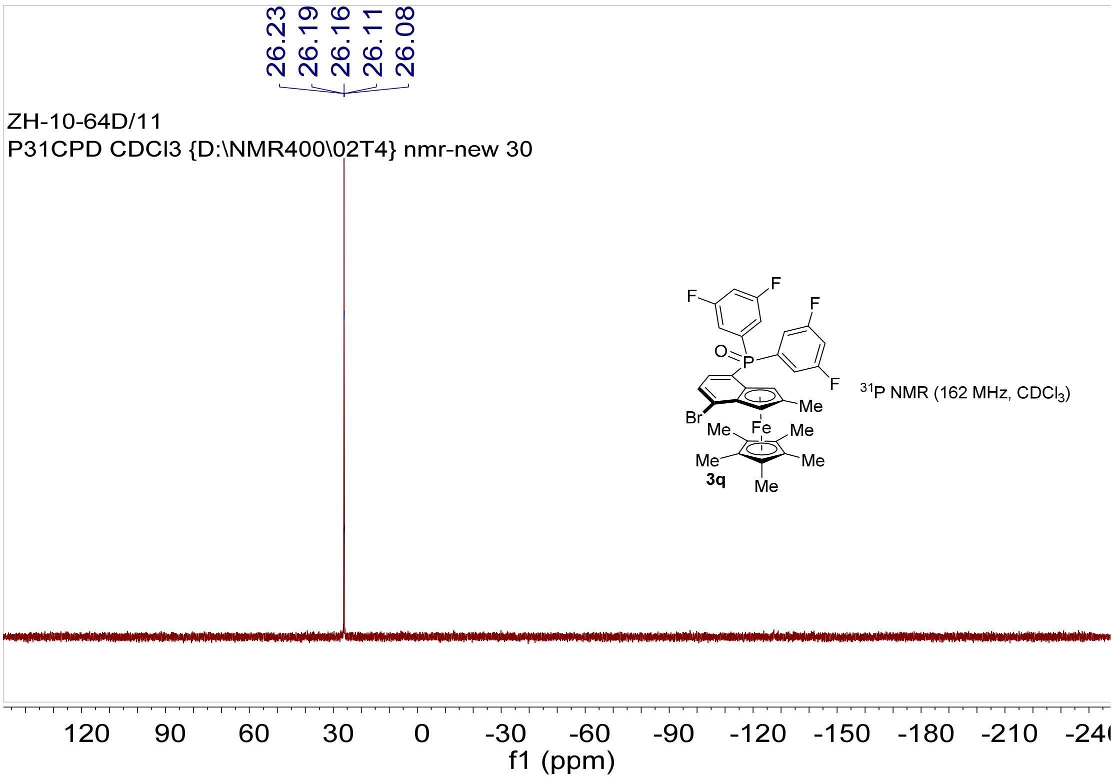

Supplementary Figure 79. ^{13}**P NMR spectra of compound 3q**

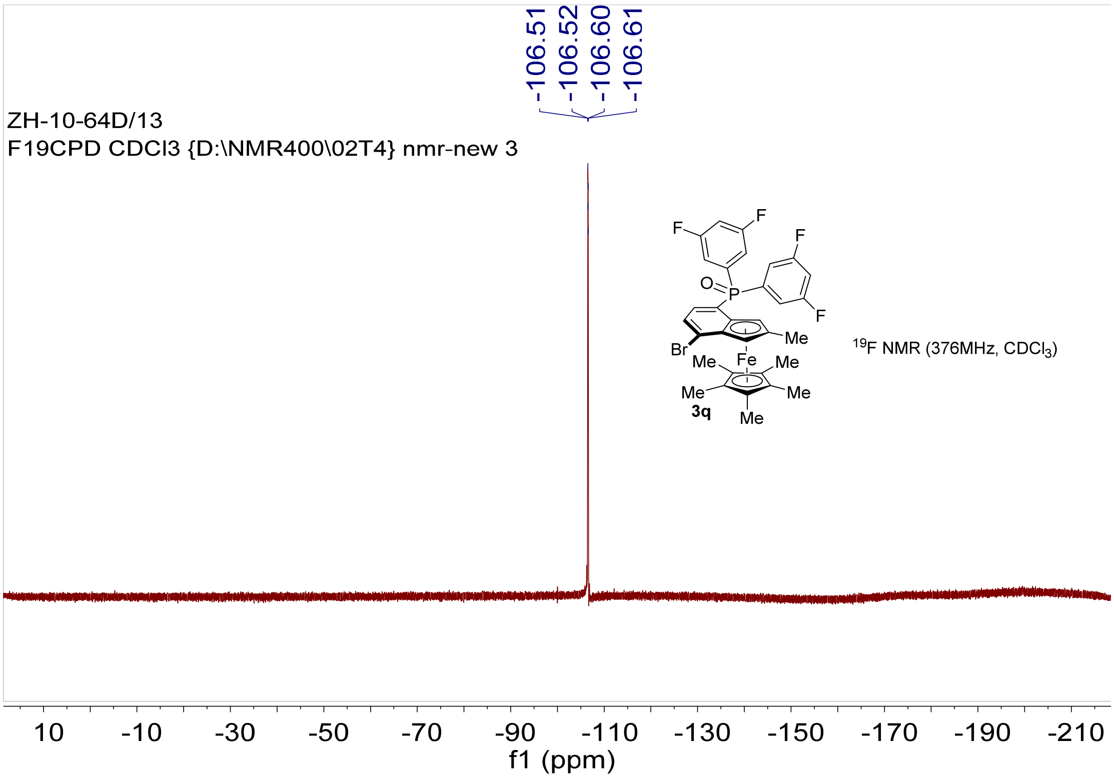

Supplementary Figure 80. ^{19}**F NMR spectra of compound 3q**

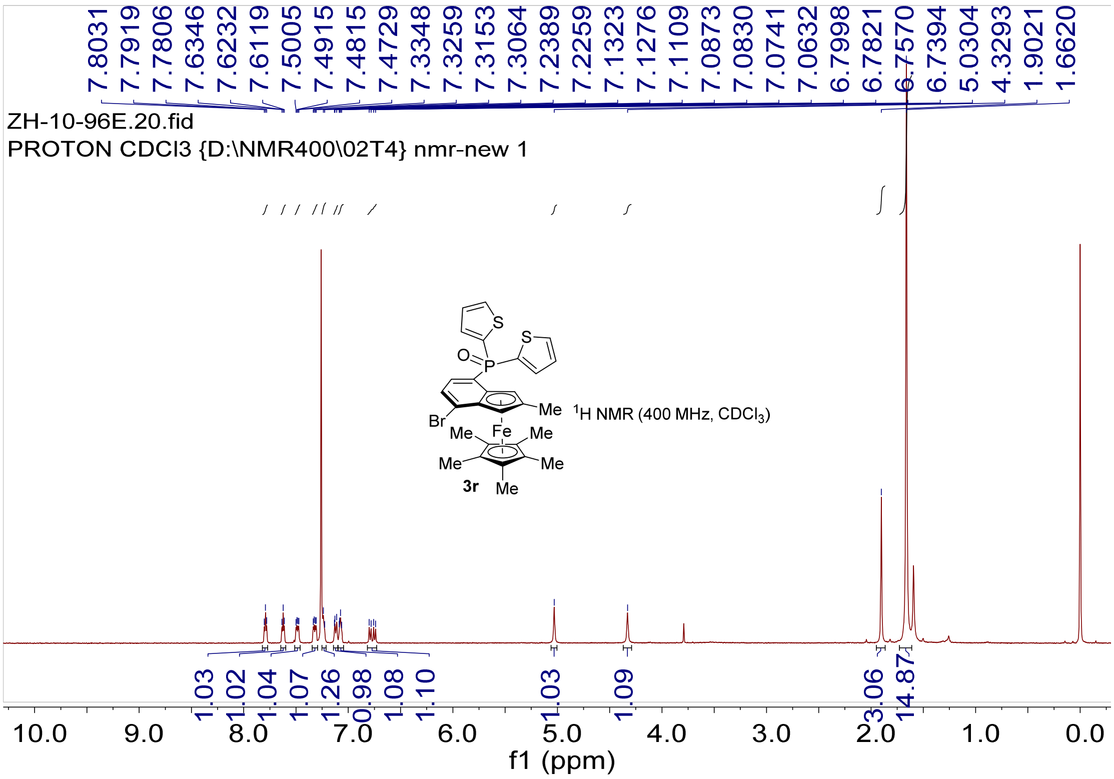

Supplementary Figure 81. ^{1}**H NMR spectra of compound 3r**

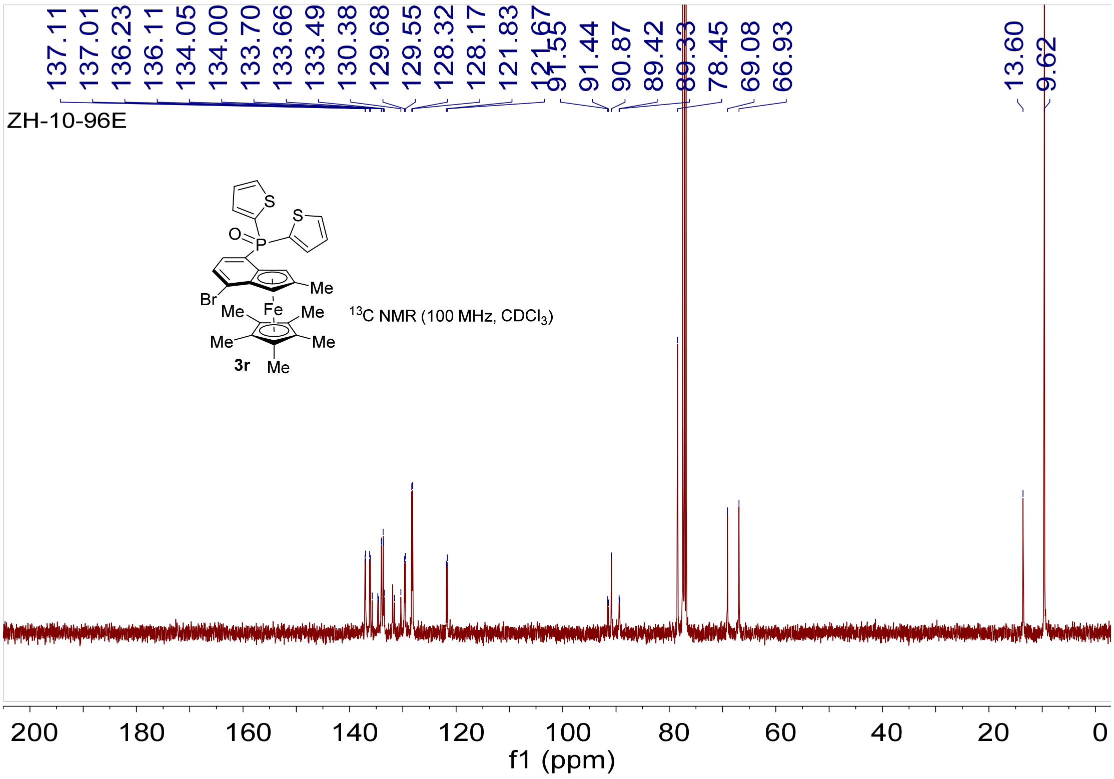

Supplementary Figure 82. ^{13}**C NMR spectra of compound 3r**

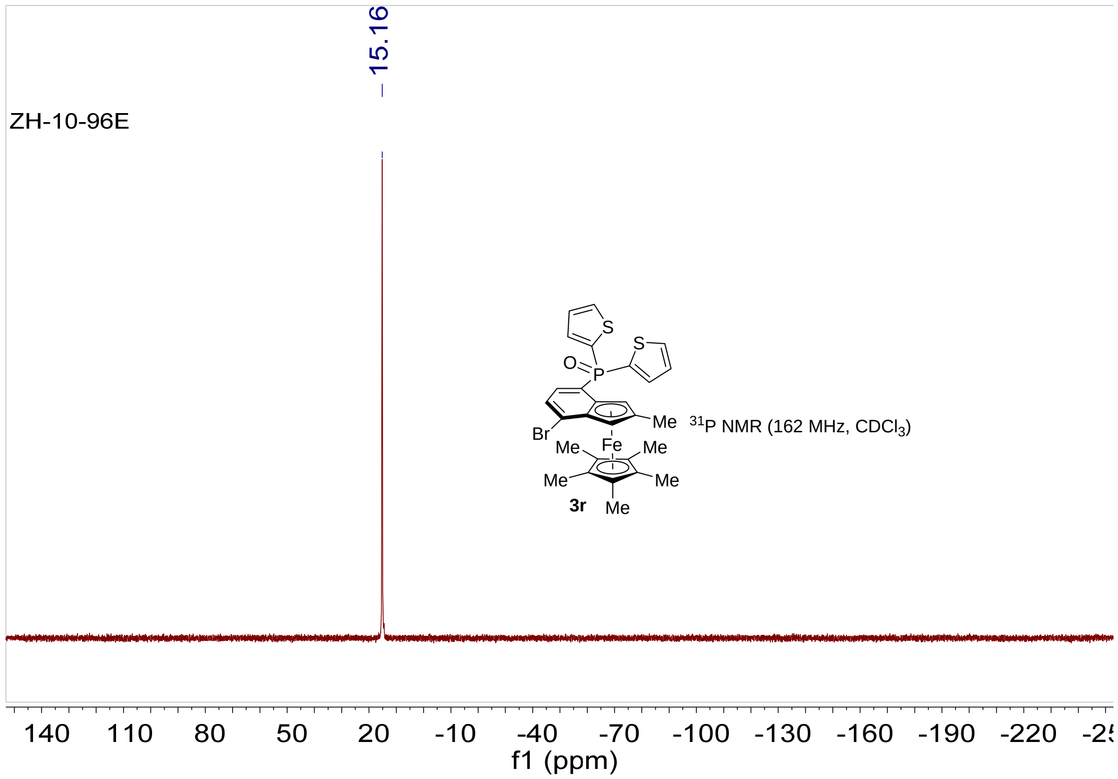

Supplementary Figure 83. ^{13}C NMR spectra of compound 3r

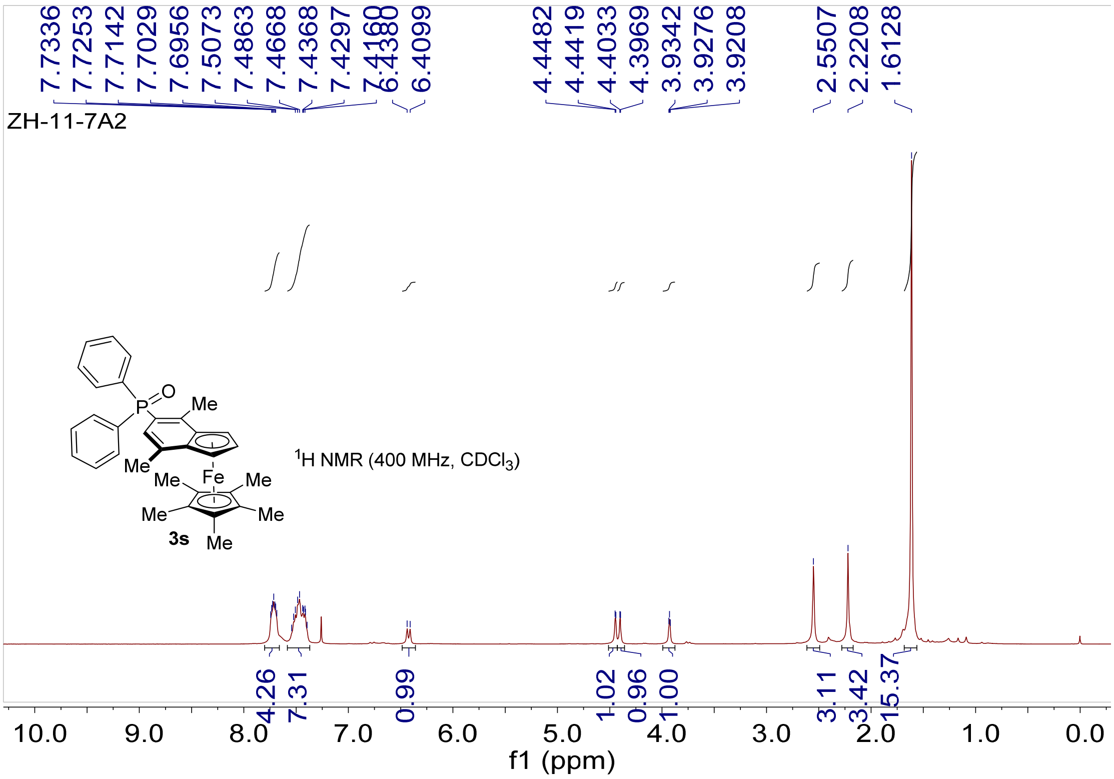

Supplementary Figure 84. ^{1}H NMR spectra of compound 3s

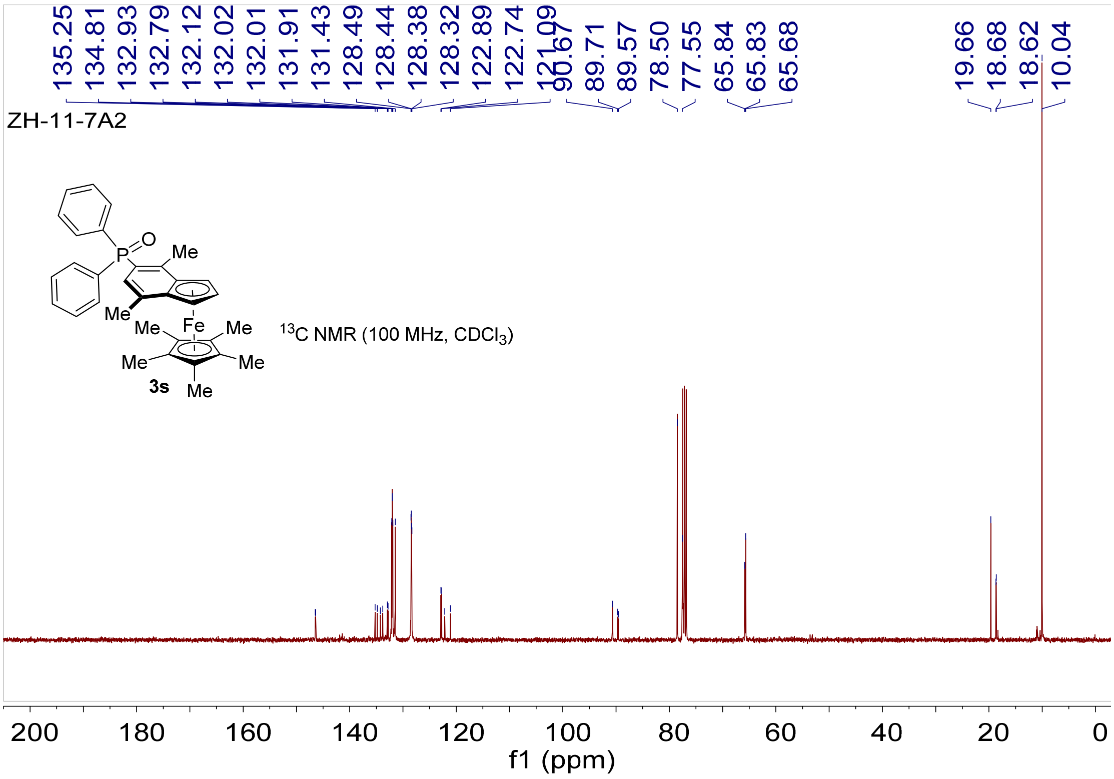

Supplementary Figure 85. ^{13}C NMR spectra of compound 3s

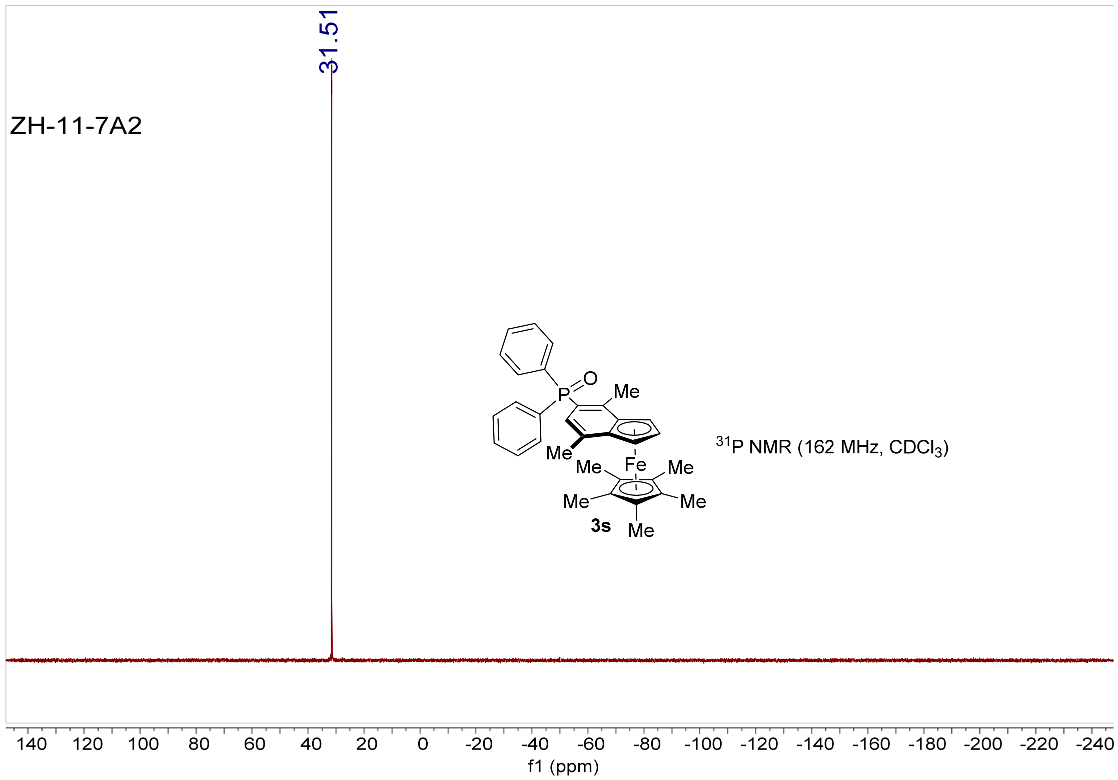

Supplementary Figure 86. ^{31}P NMR spectra of compound 3s

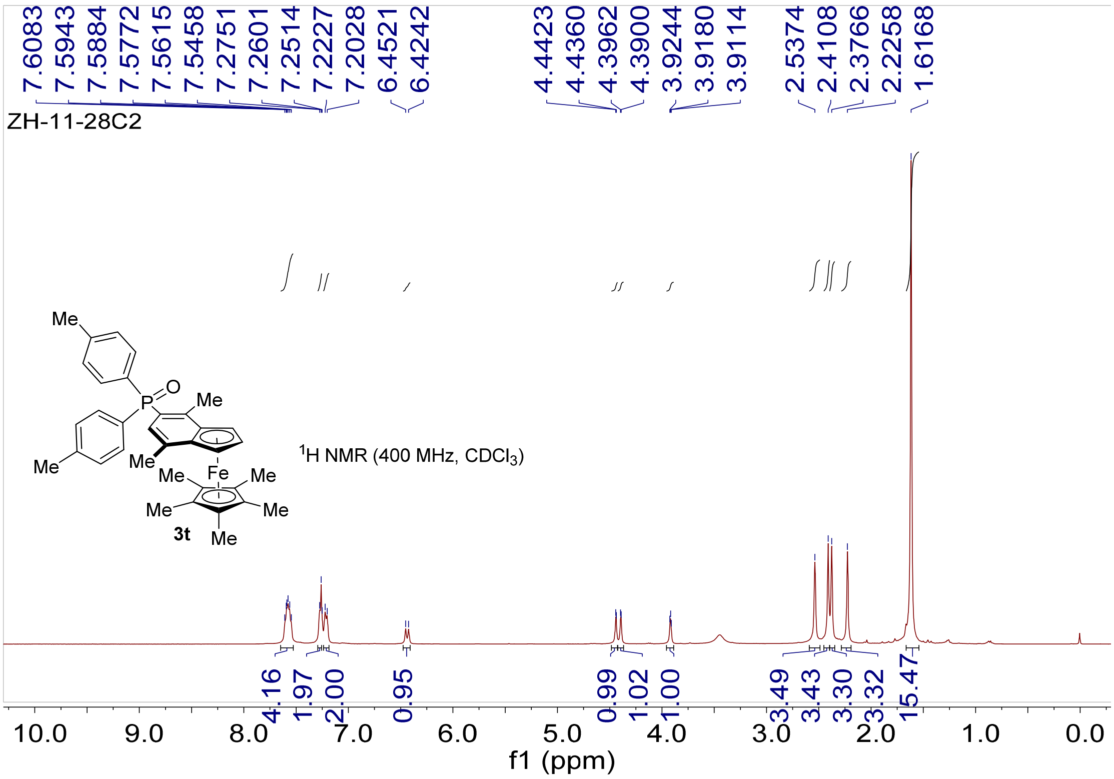

Supplementary Figure 87 ^{1}H NMR spectra of compound 3t

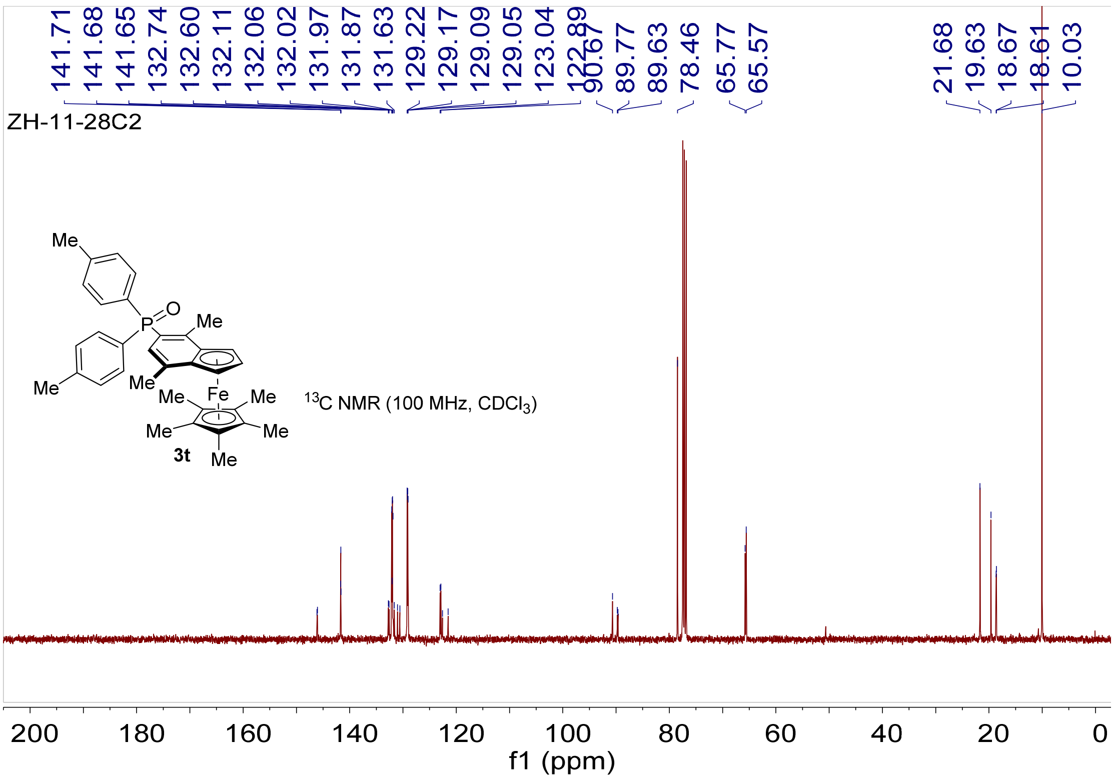

Supplementary Figure 88. ^{13}C NMR spectra of compound 3t

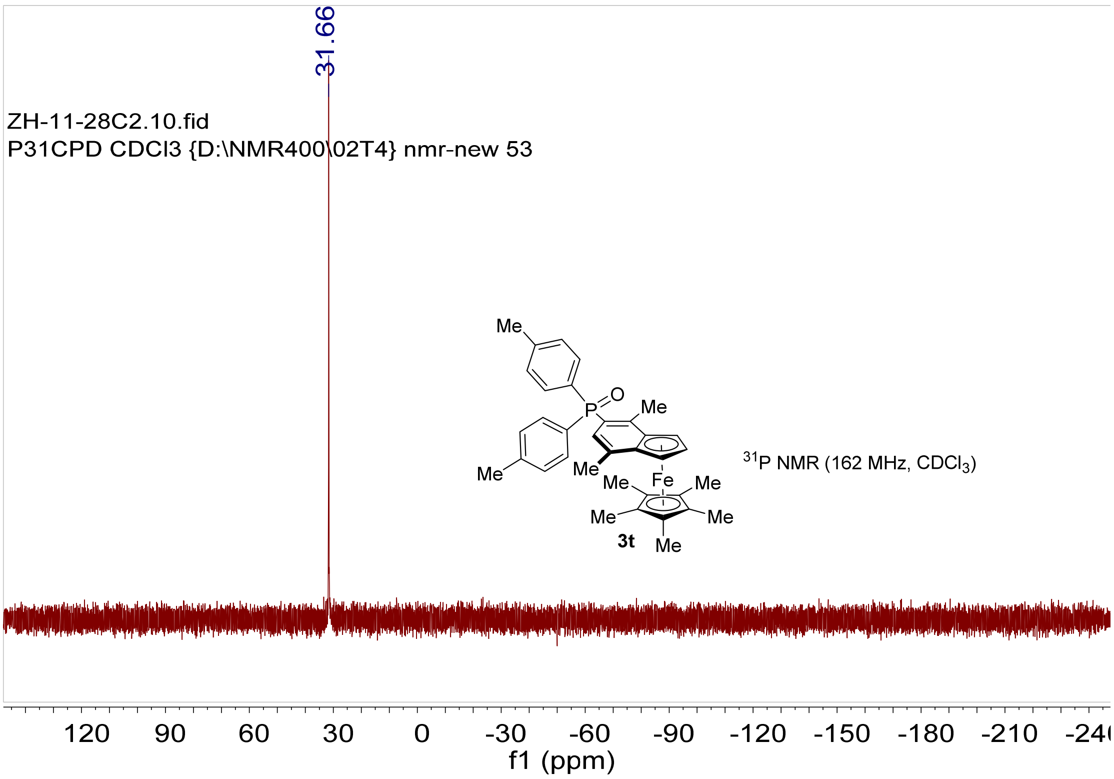

Supplementary Figure 89. ^{31}P NMR spectra of compound 3t

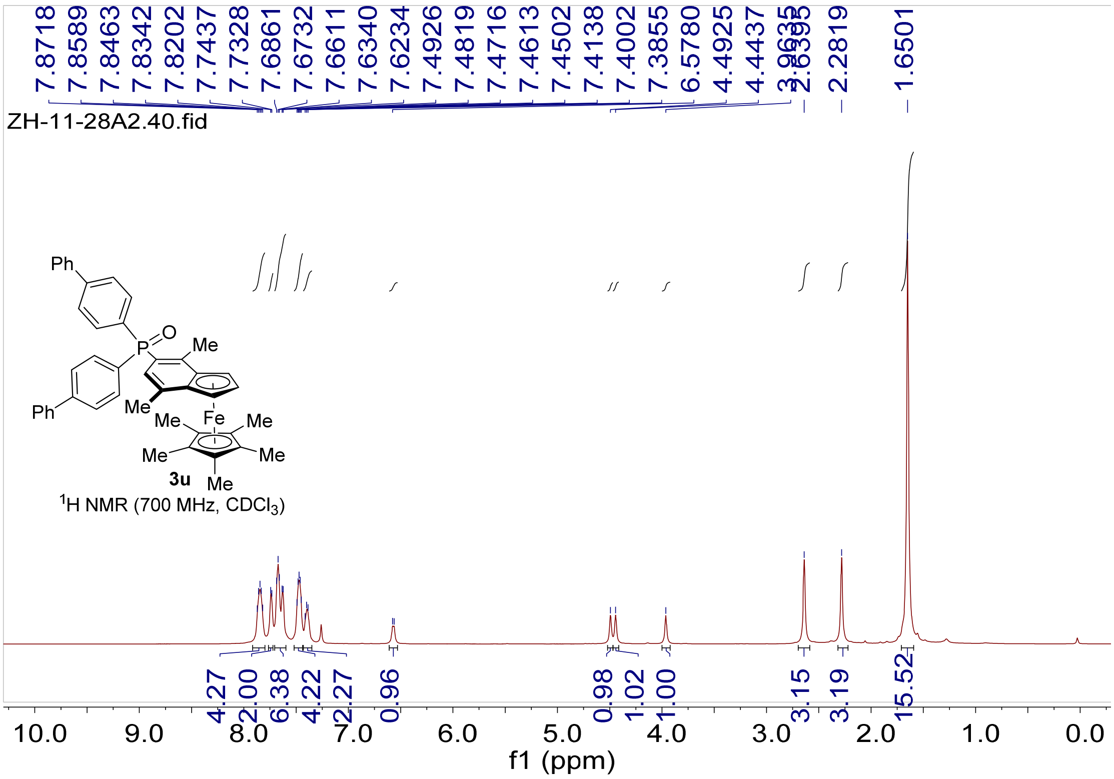

Supplementary Figure 90. ^{1}H NMR spectra of compound 3u

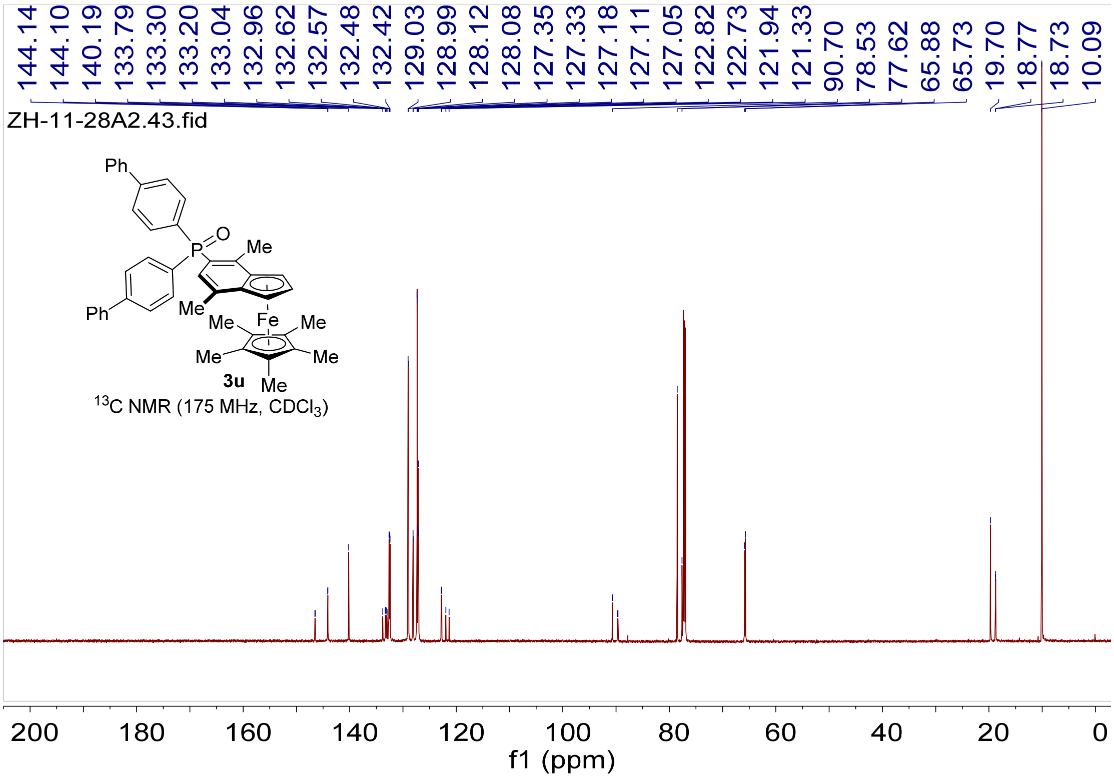

Supplementary Figure 91. ^{13}C NMR spectra of compound 3u

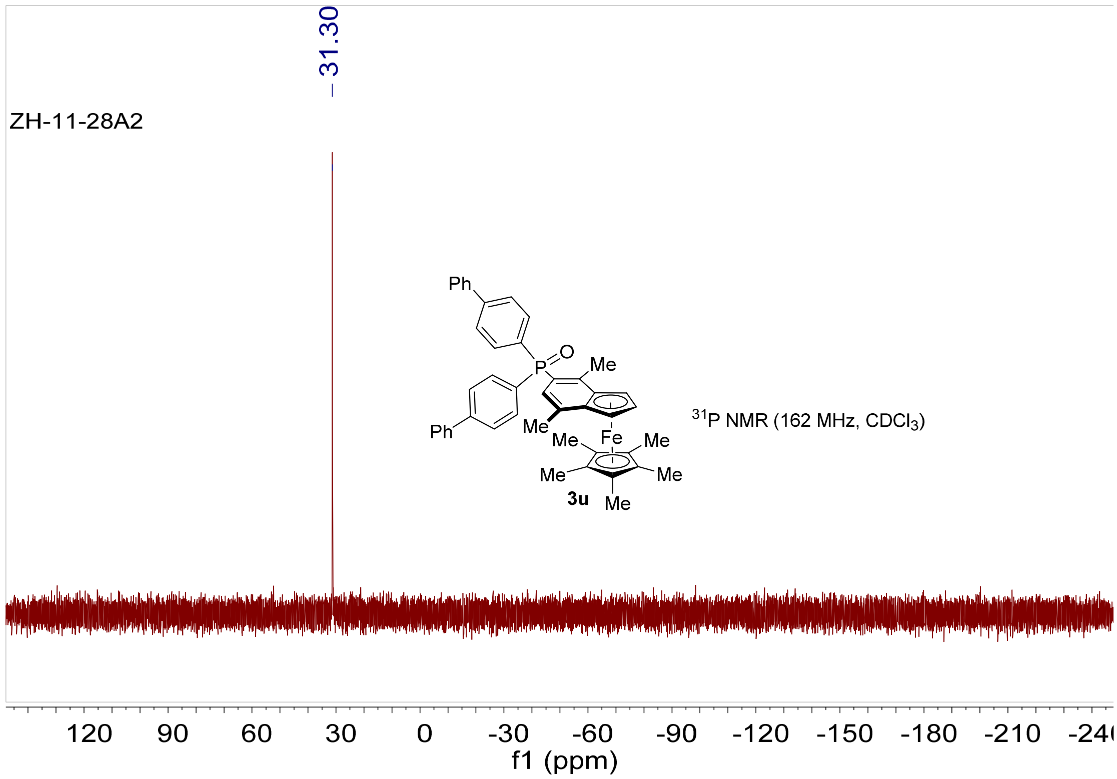

Supplementary Figure 92. ^{31}P NMR spectra of compound 3u

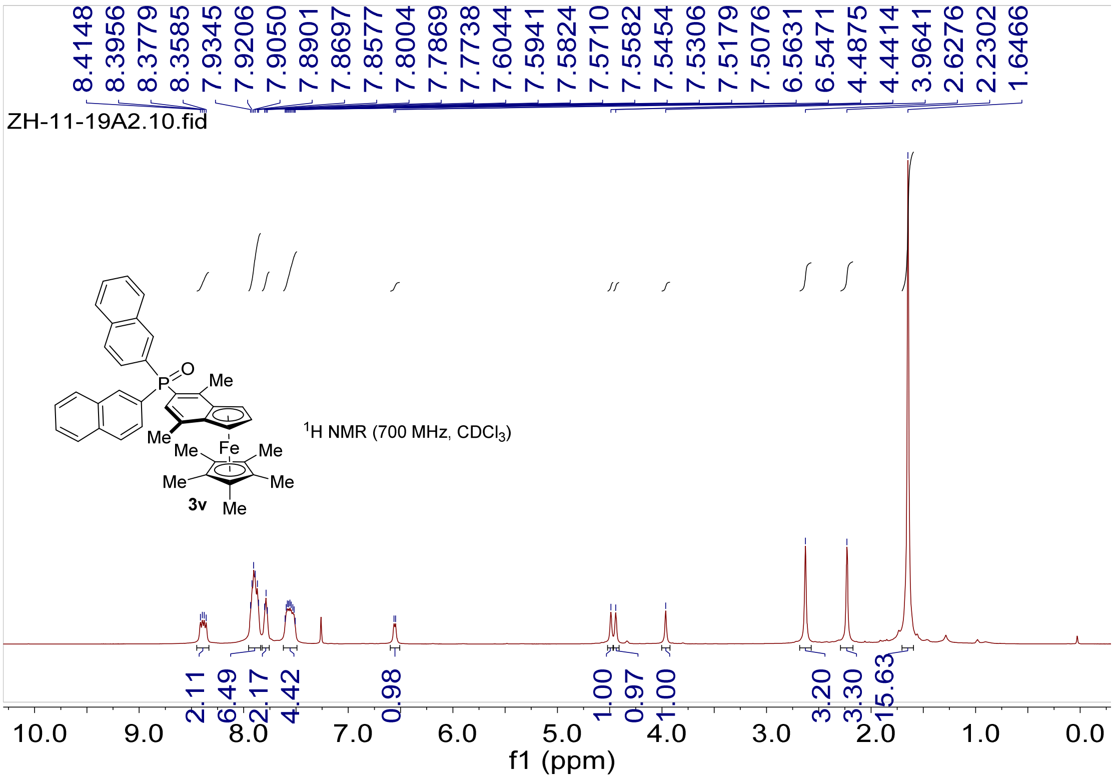

Supplementary Figure 93. ^{1}H NMR spectra of compound 3v

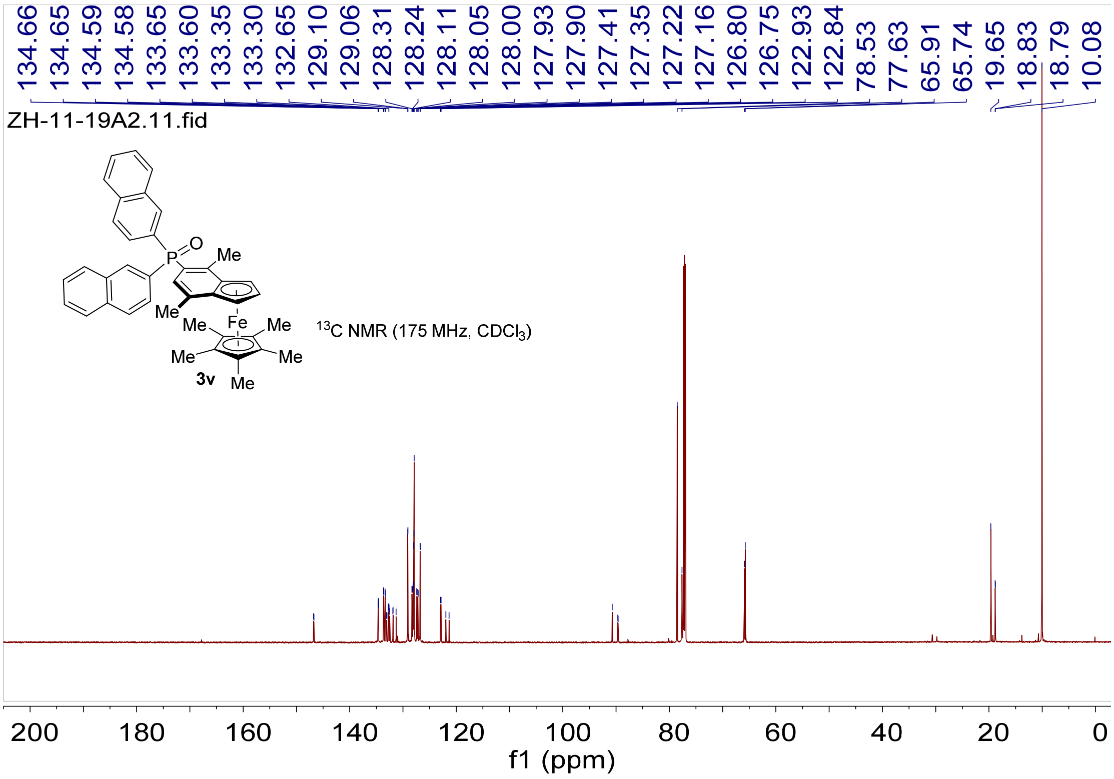

Supplementary Figure 94. ^{13}C NMR spectra of compound 3v

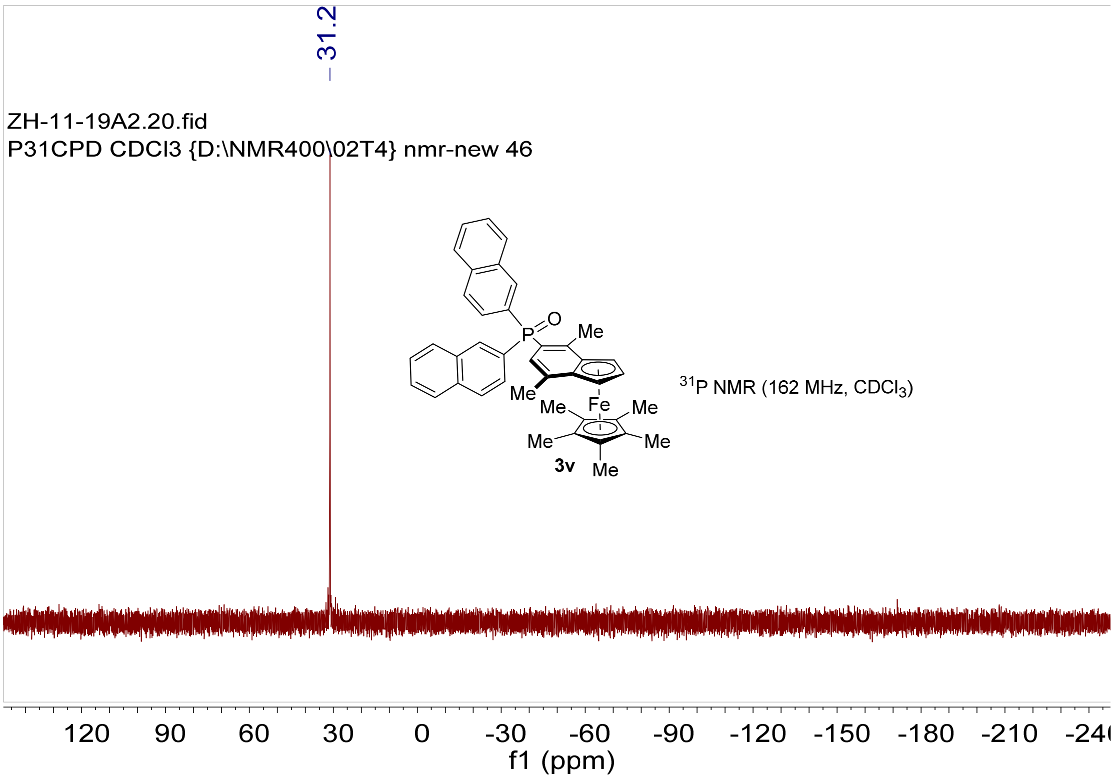

Supplementary Figure 95. ^{31}P NMR spectra of compound 3v

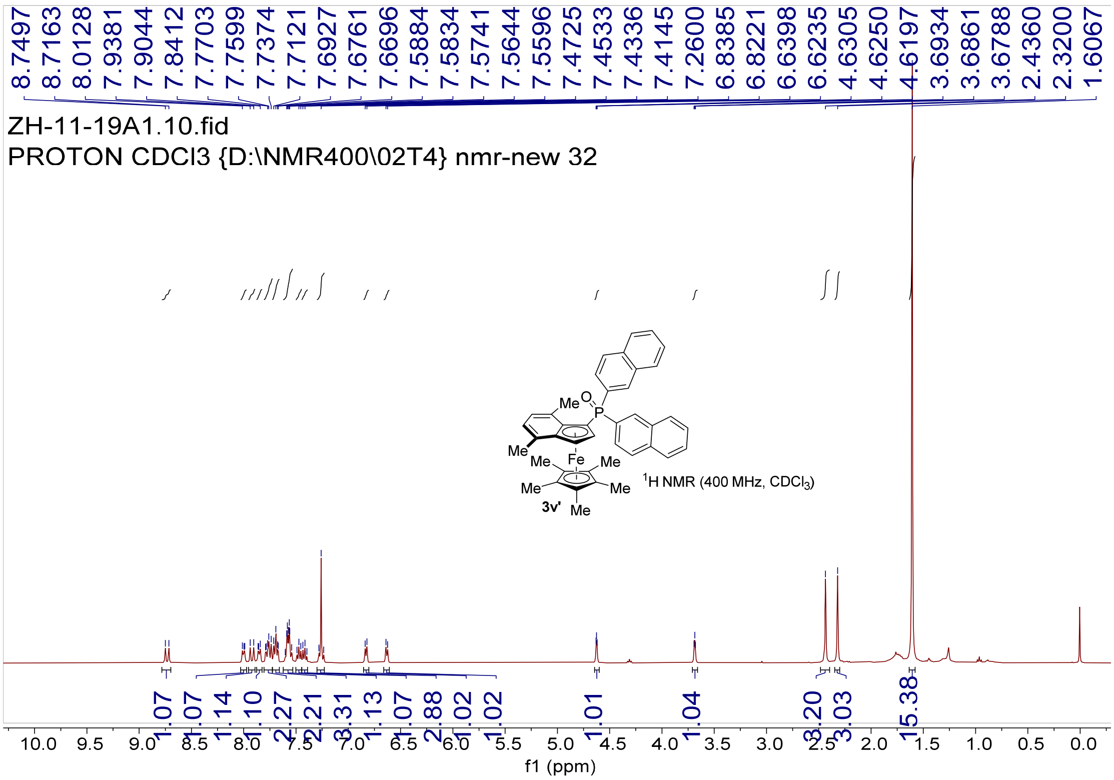

Supplementary Figure 96. ^{1}H NMR spectra of compound 3v'

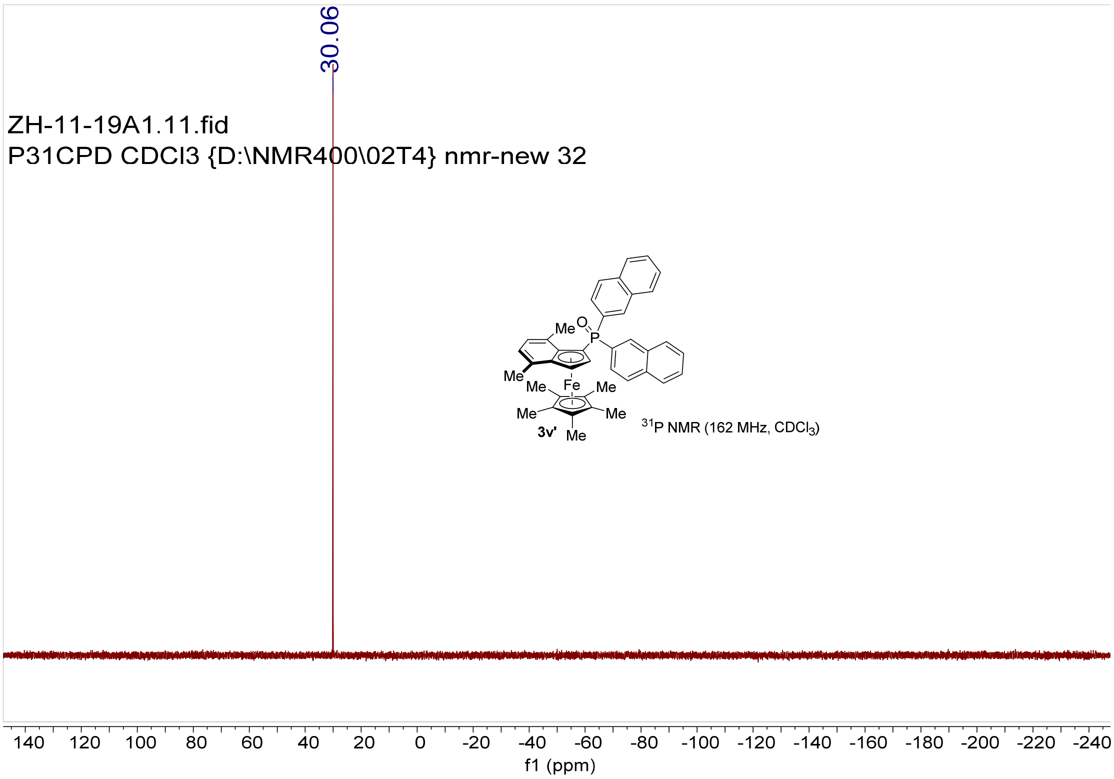

Supplementary Figure 97. ^{13}C NMR spectra of compound 3v'

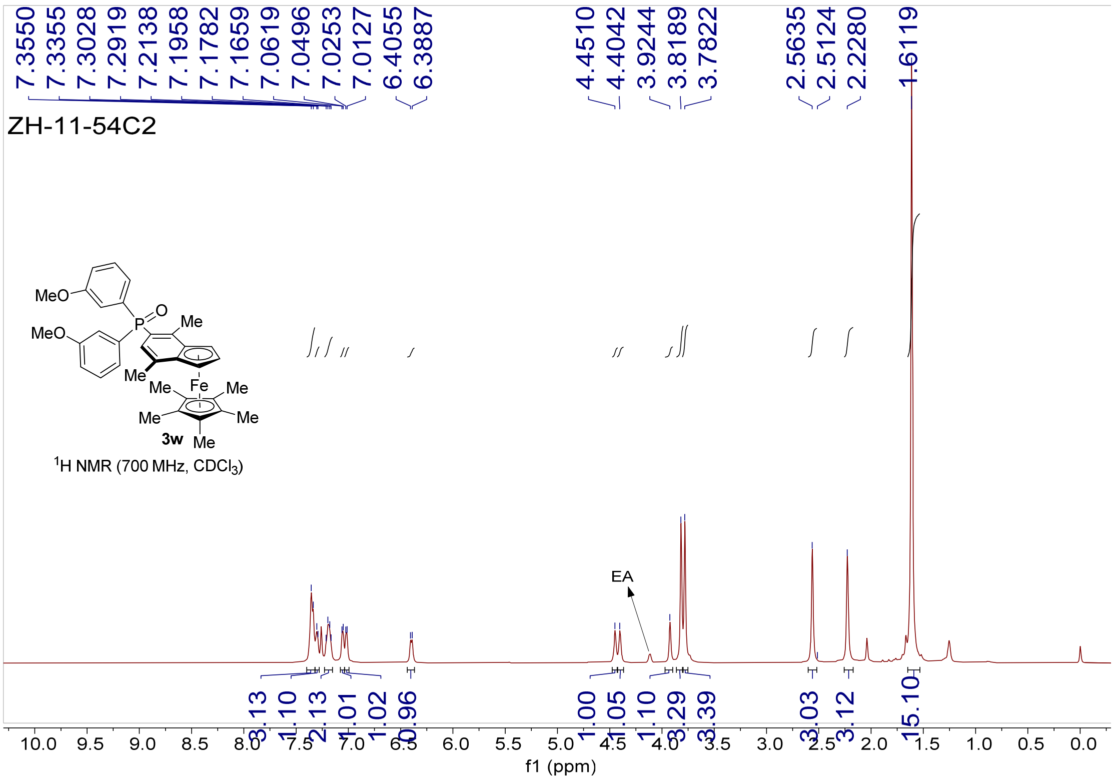

Supplementary Figure 98. ^{1}**H NMR spectra of compound 3w**

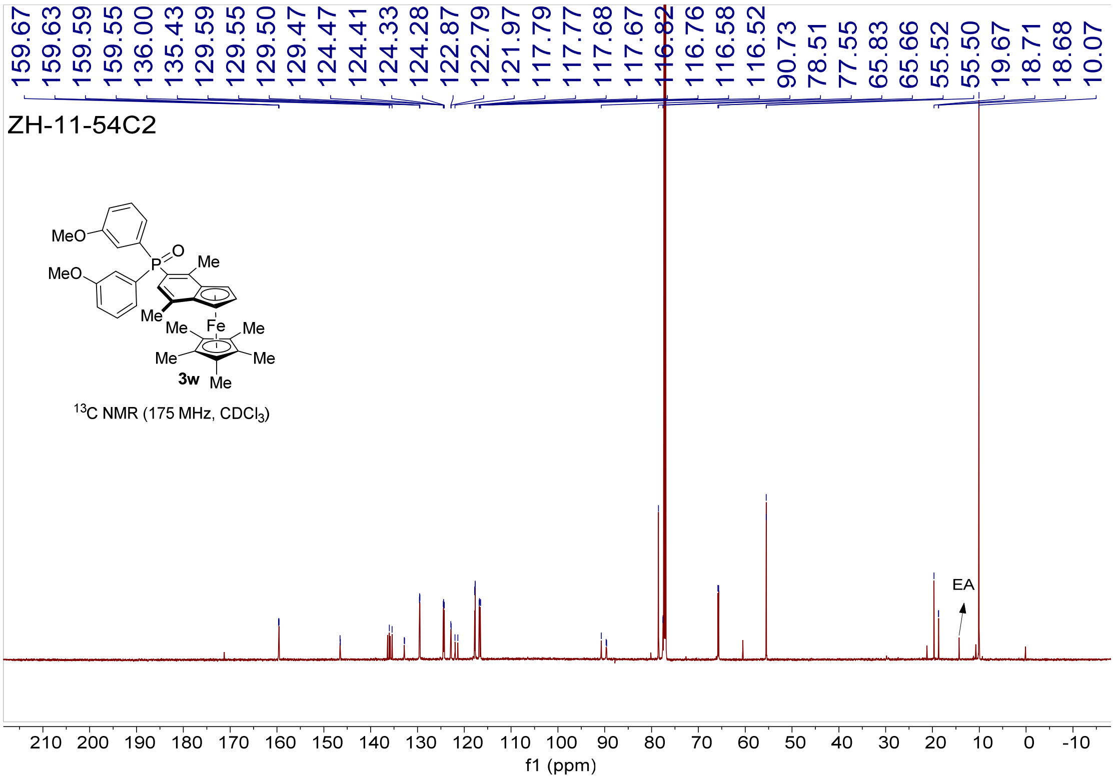

Supplementary Figure 99. ^{13}**C NMR spectra of compound 3w**

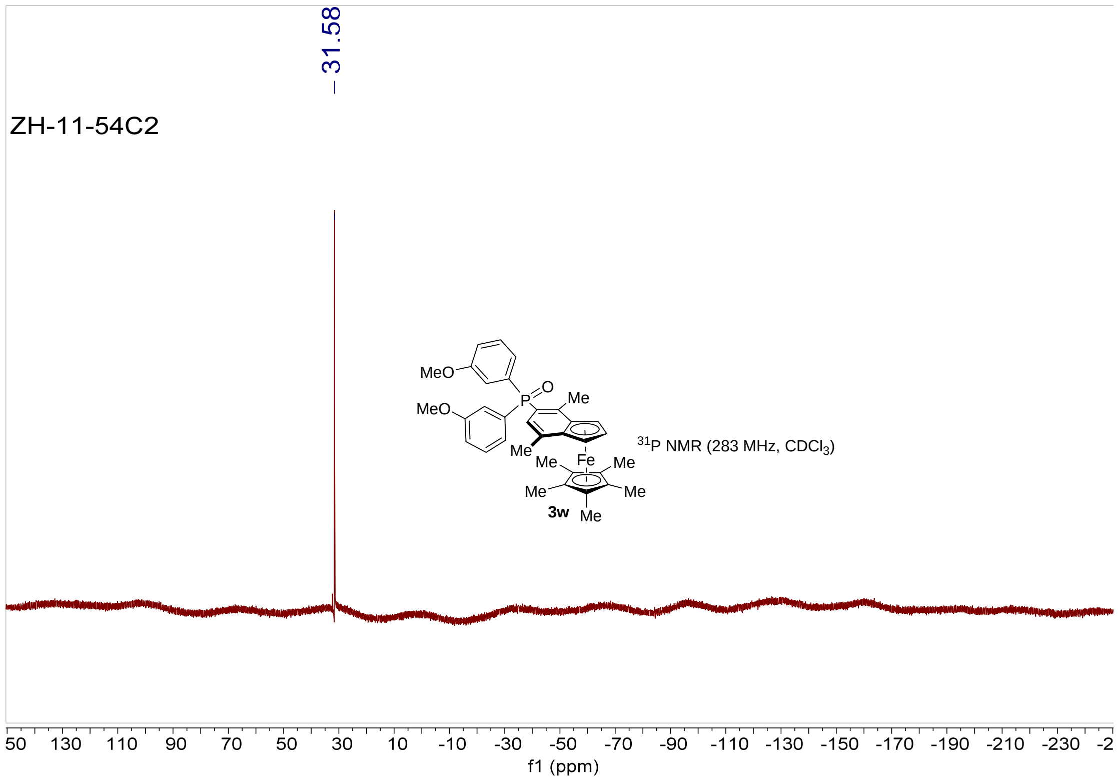

Supplementary Figure 100. ^{31}P NMR spectra of compound 3w

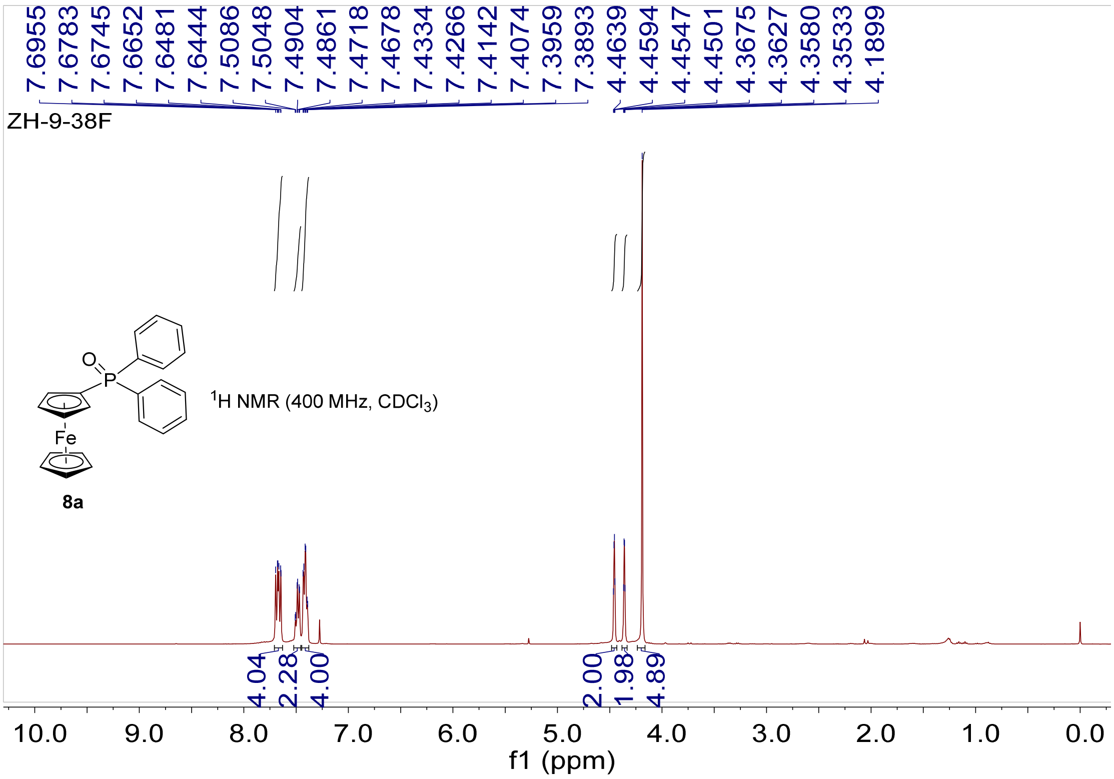

Supplementary Figure 101. ^{1}H NMR spectra of compound 8a

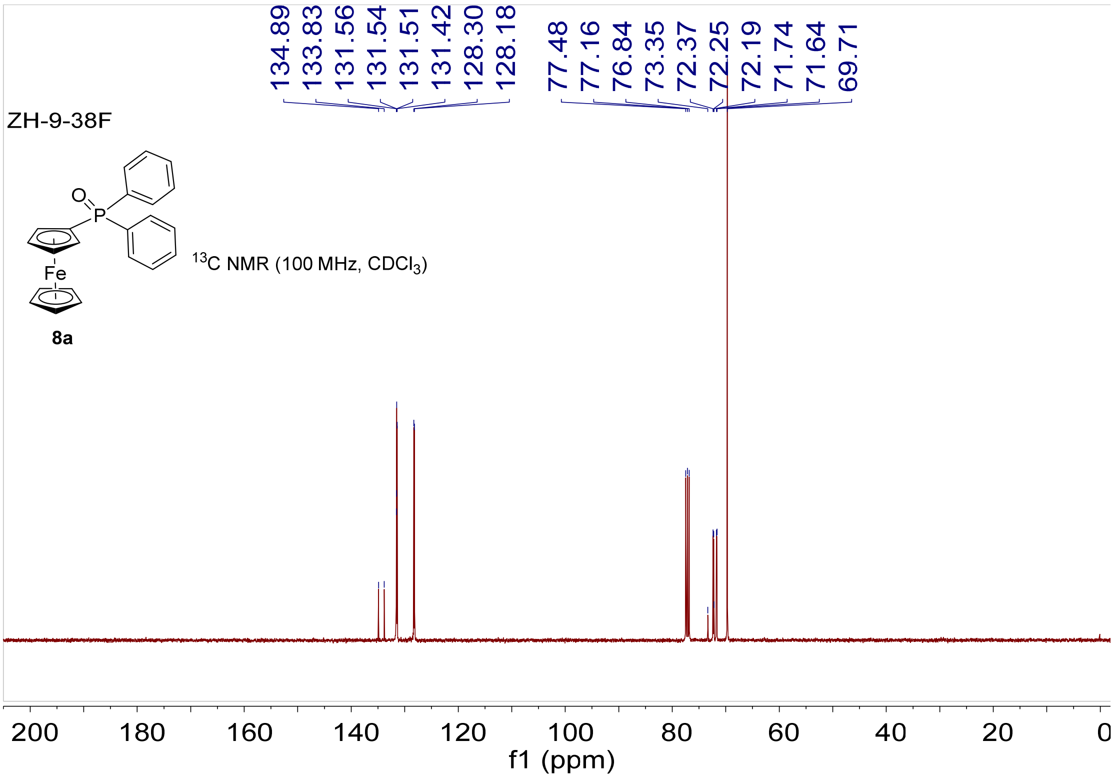

Supplementary Figure 102. ^{13}C NMR spectra of compound 8a

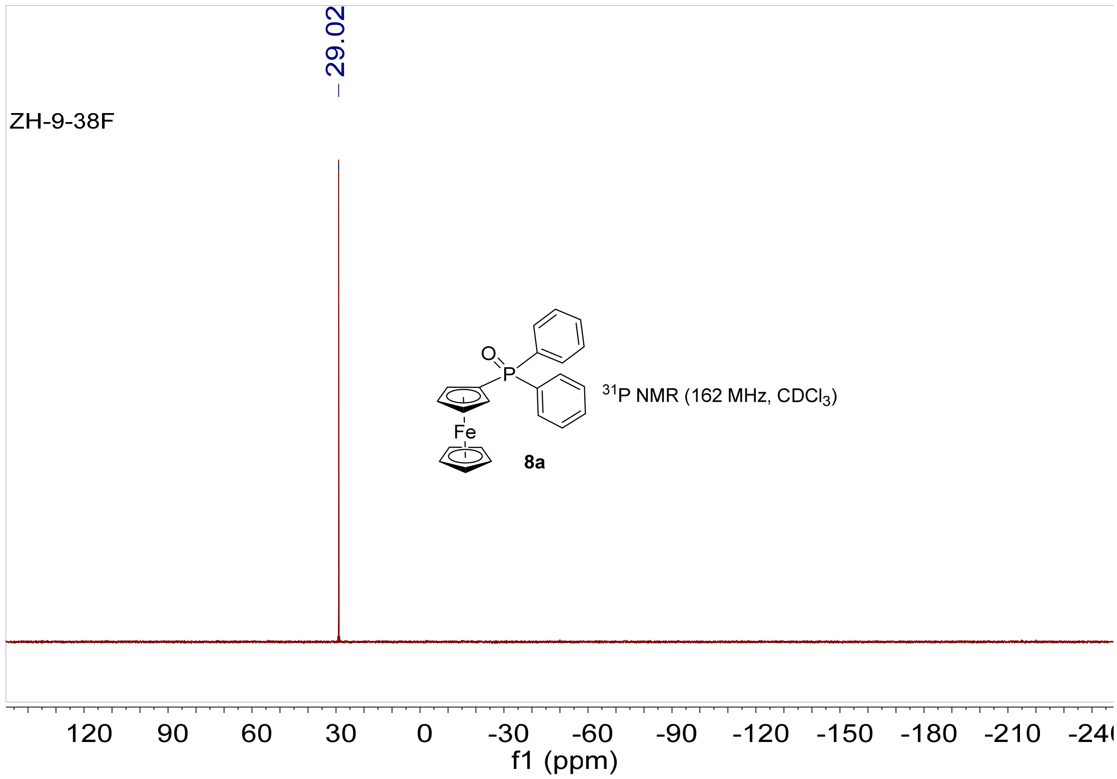

Supplementary Figure 103. ^{31}P NMR spectra of compound 8a

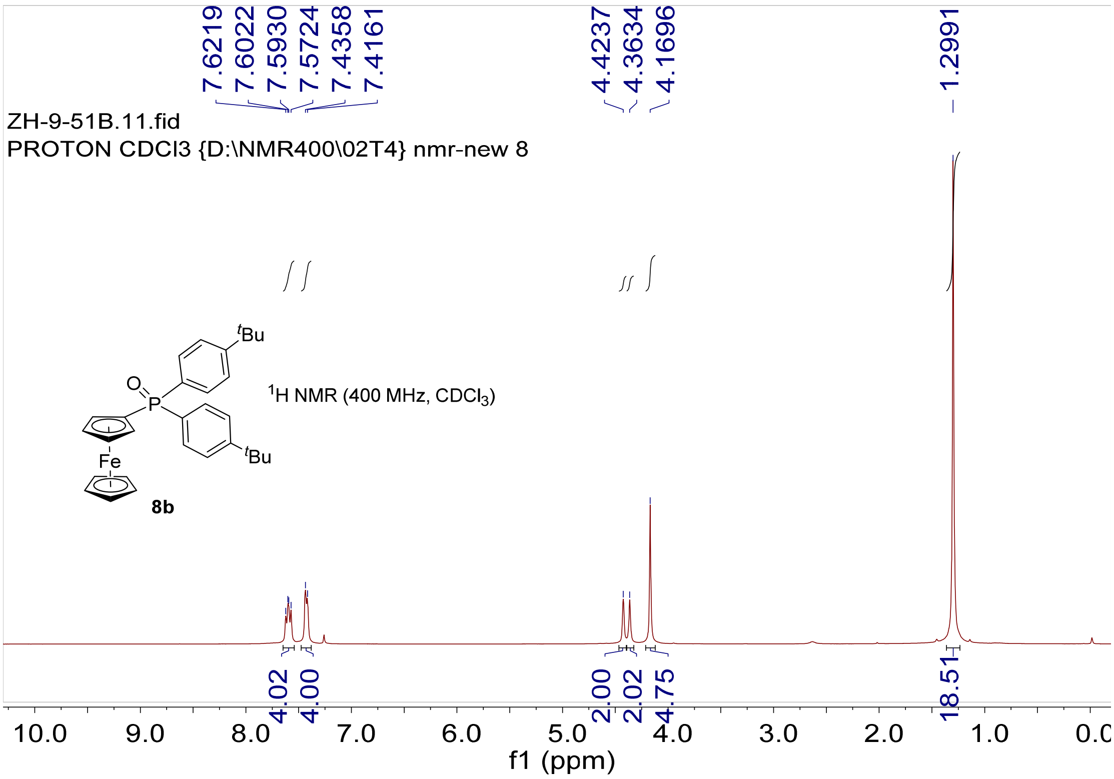

Supplementary Figure 104. ^{1}**H NMR spectra of compound 8b**

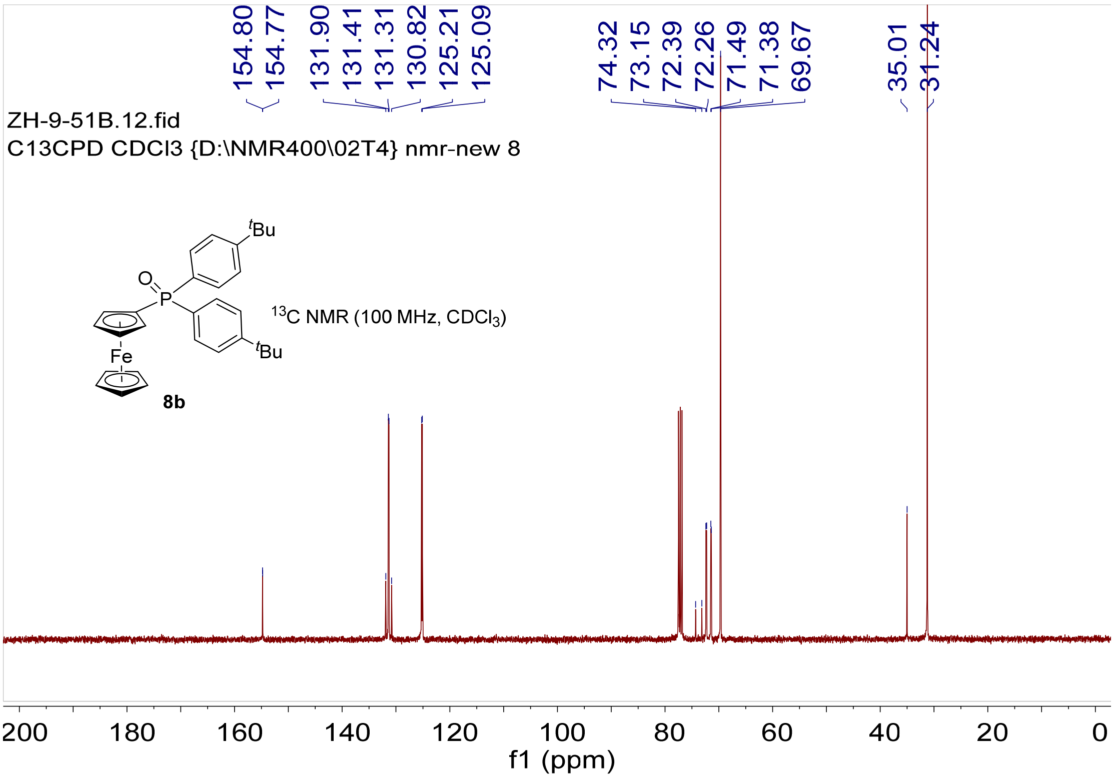

Supplementary Figure 105. ^{13}**C NMR spectra of compound 8b**

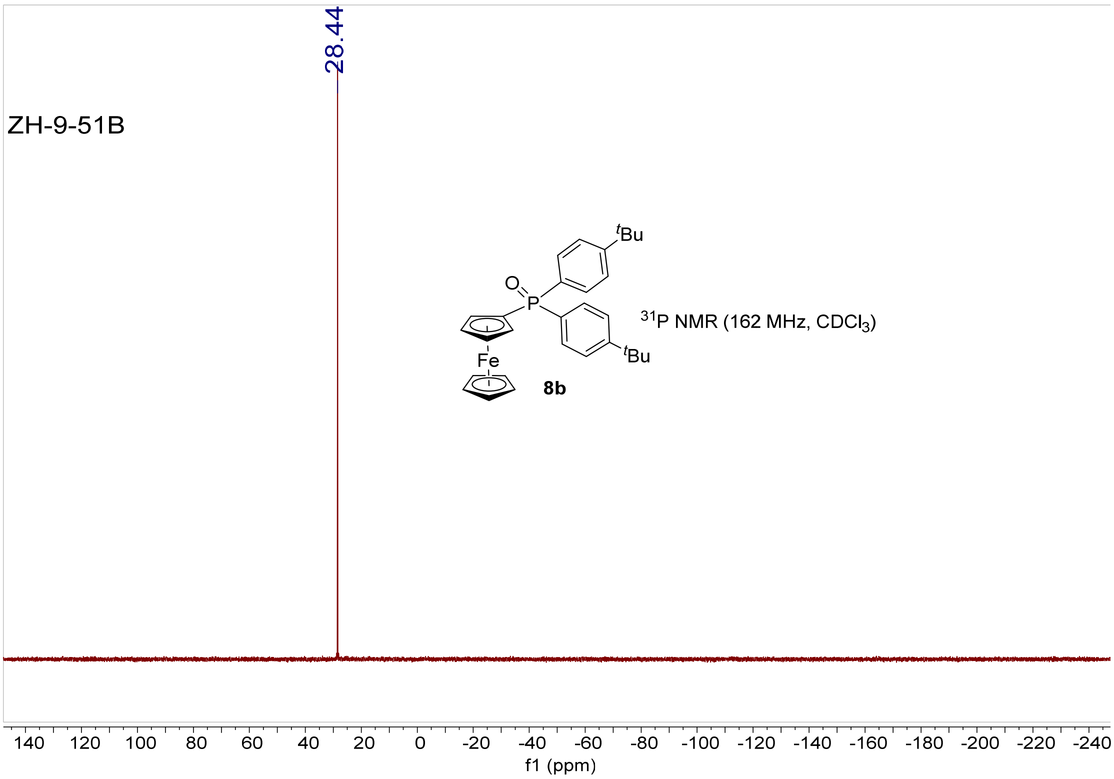

Supplementary Figure 106. ^{31}P NMR spectra of compound 8b

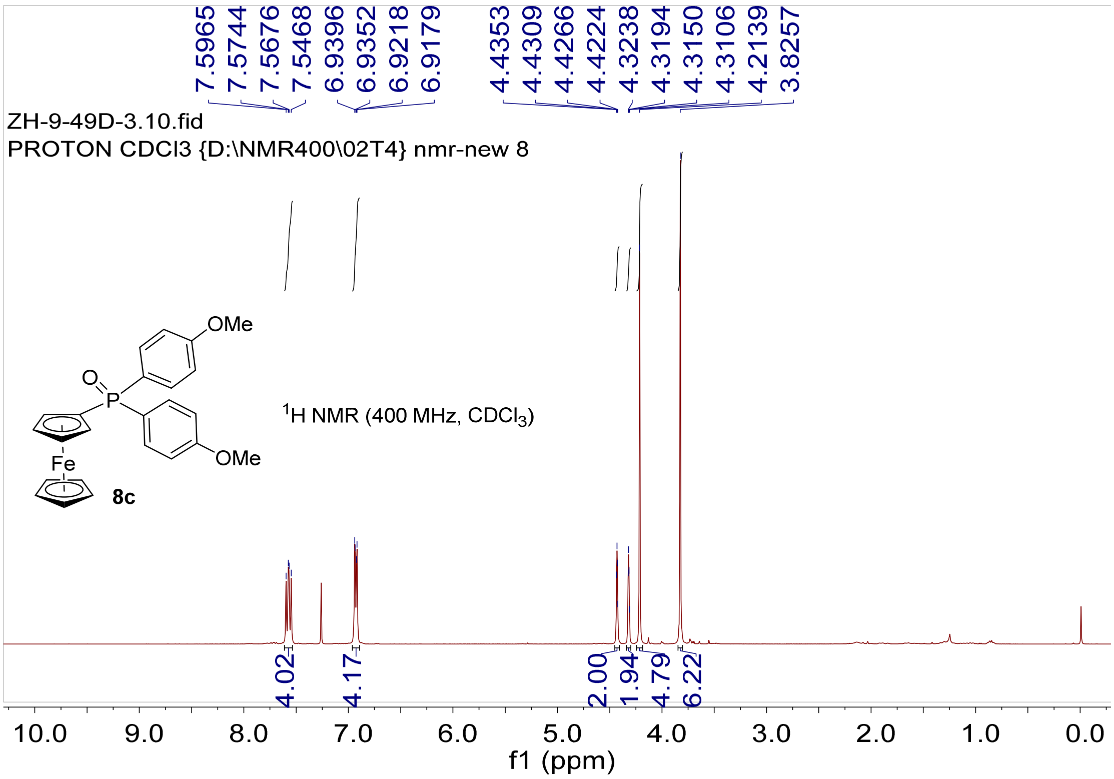

Supplementary Figure 107. ^{1}H NMR spectra of compound 8c

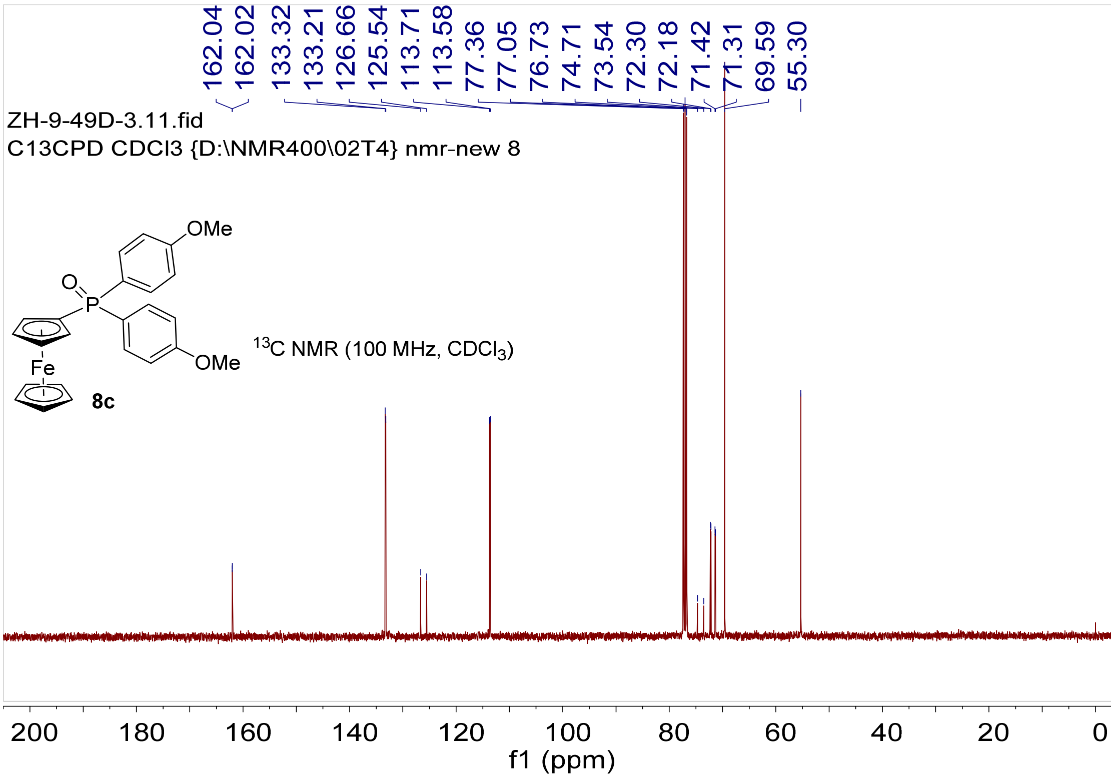

Supplementary Figure 108. ^{13}C NMR spectra of compound 8c

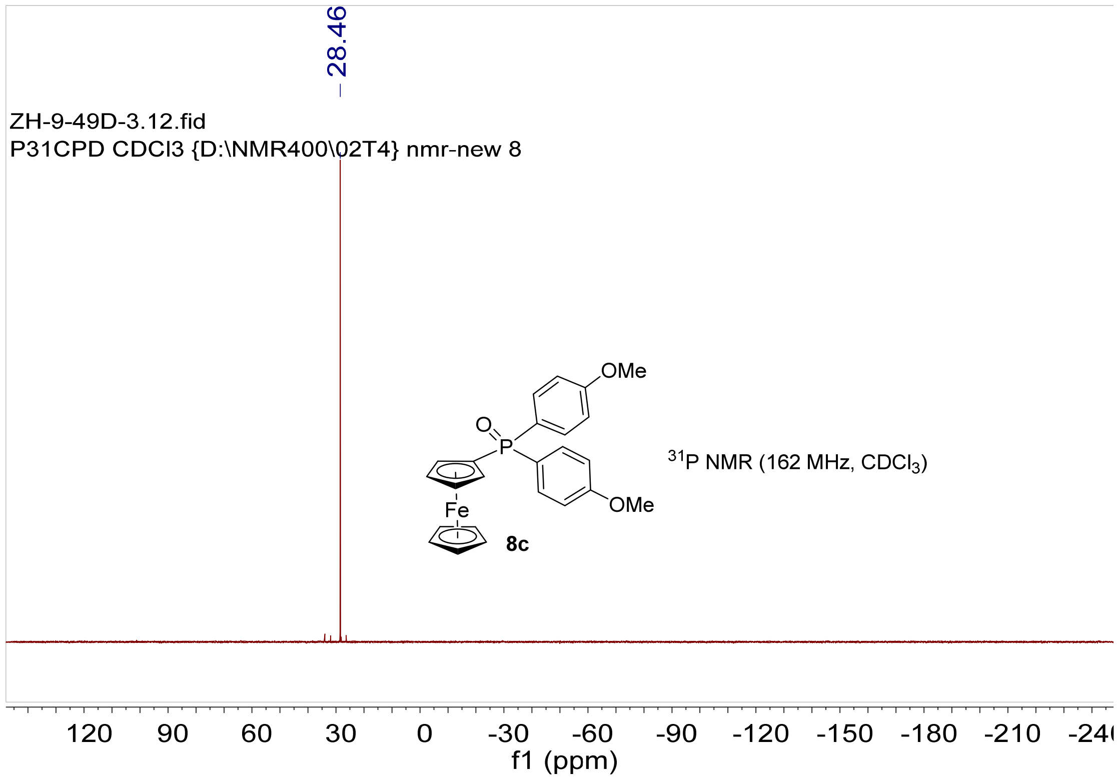

Supplementary Figure 109. ^{31}P NMR spectra of compound 8c

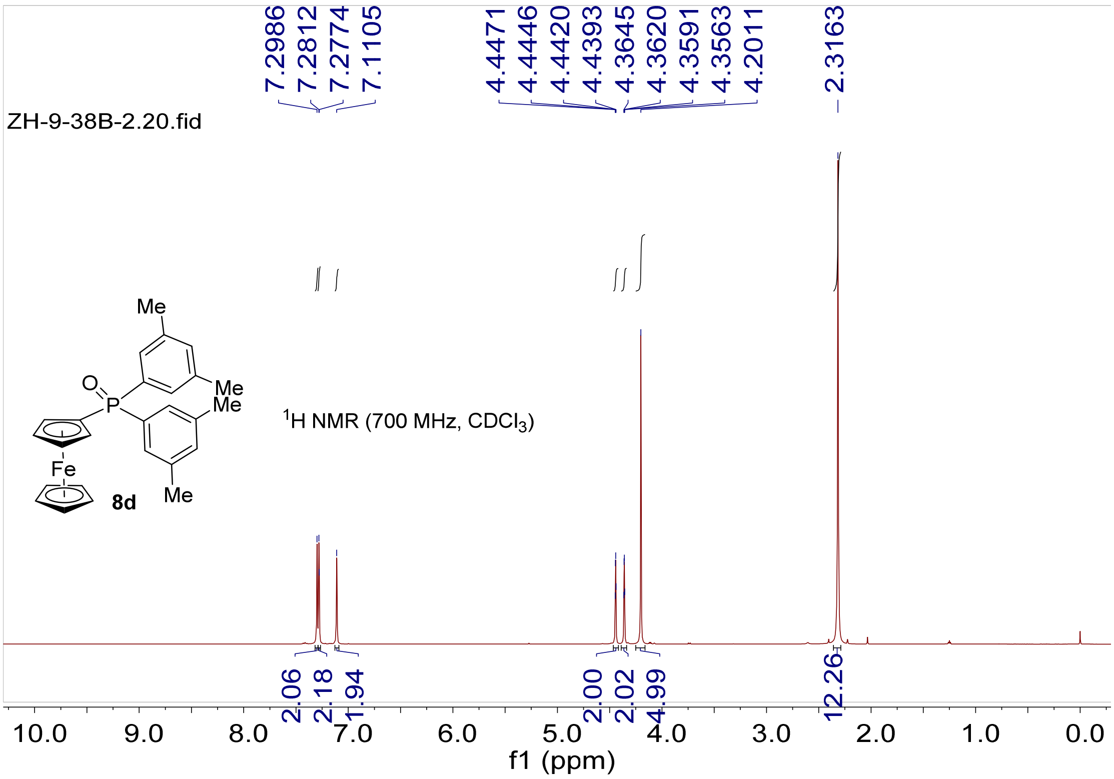

Supplementary Figure 110. ^{1}H NMR spectra of compound 8d

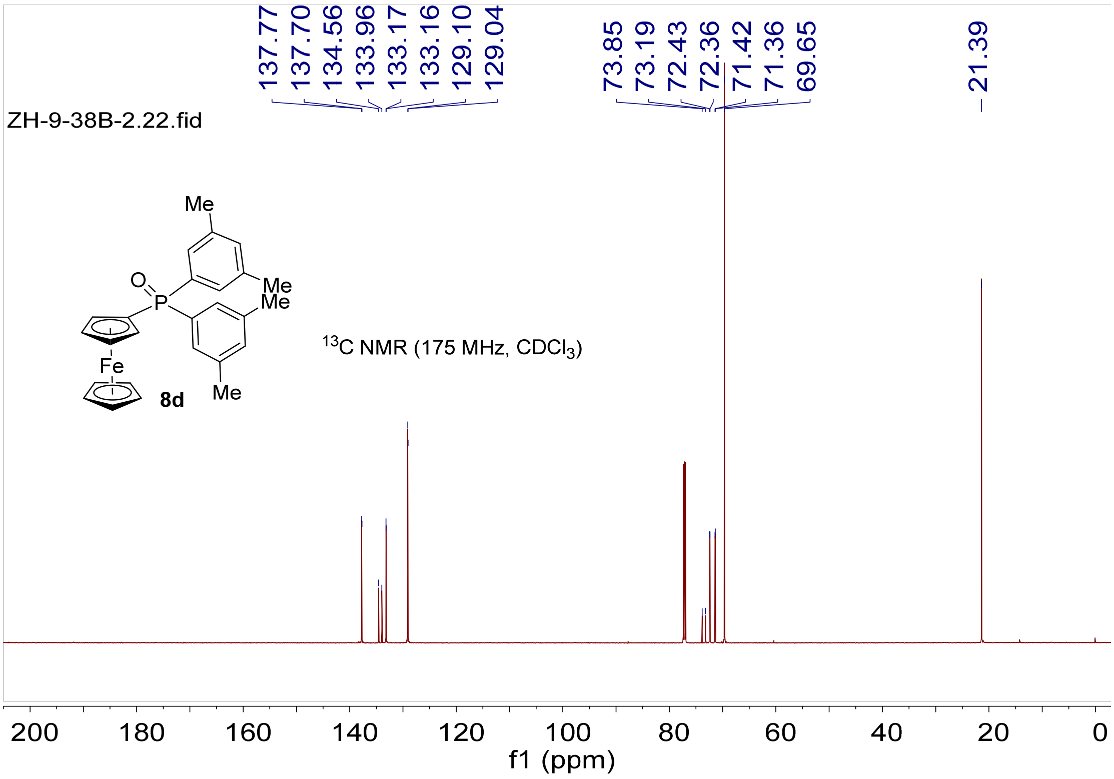

Supplementary Figure 111. ^{13}C NMR spectra of compound 8d

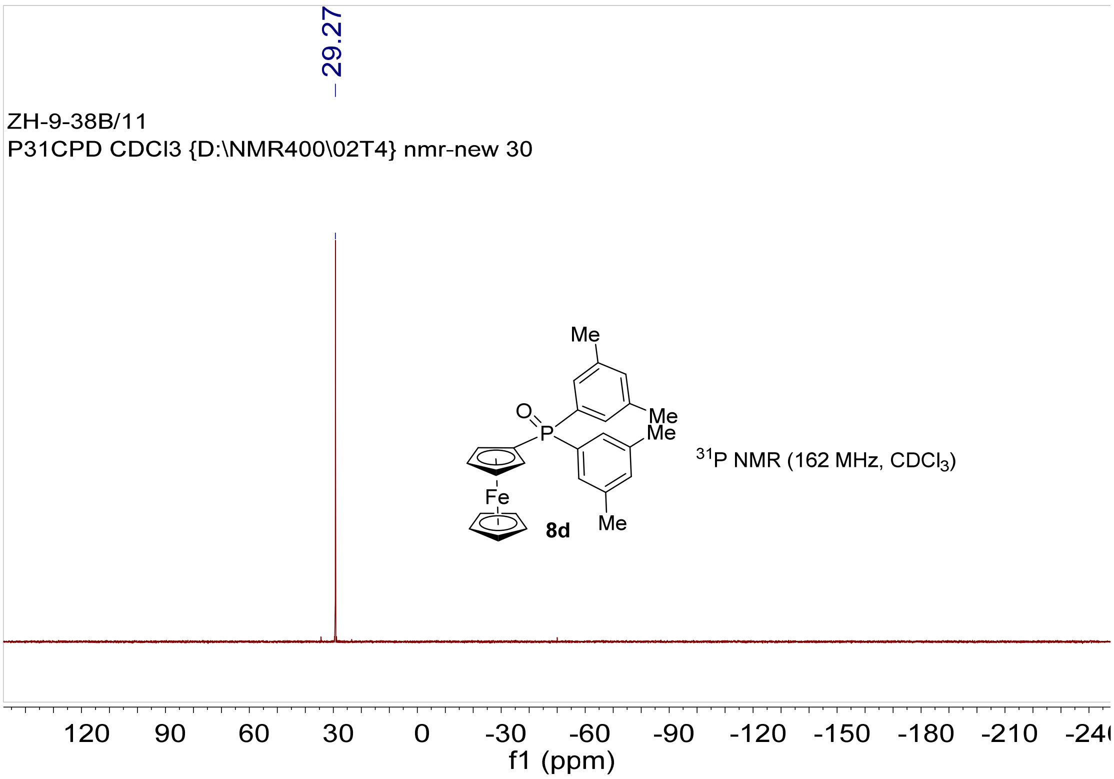

Supplementary Figure 112. ^{31}P NMR spectra of compound 8d

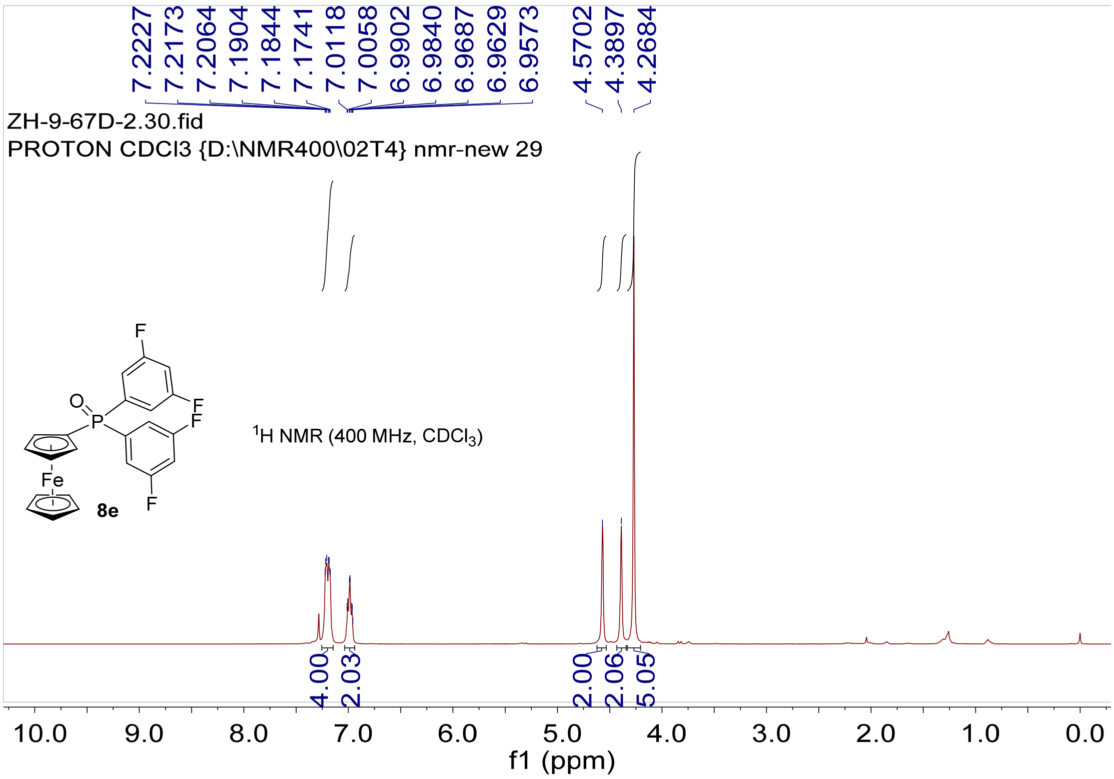

Supplementary Figure 113. ^{1}**H NMR spectra of compound 8e**

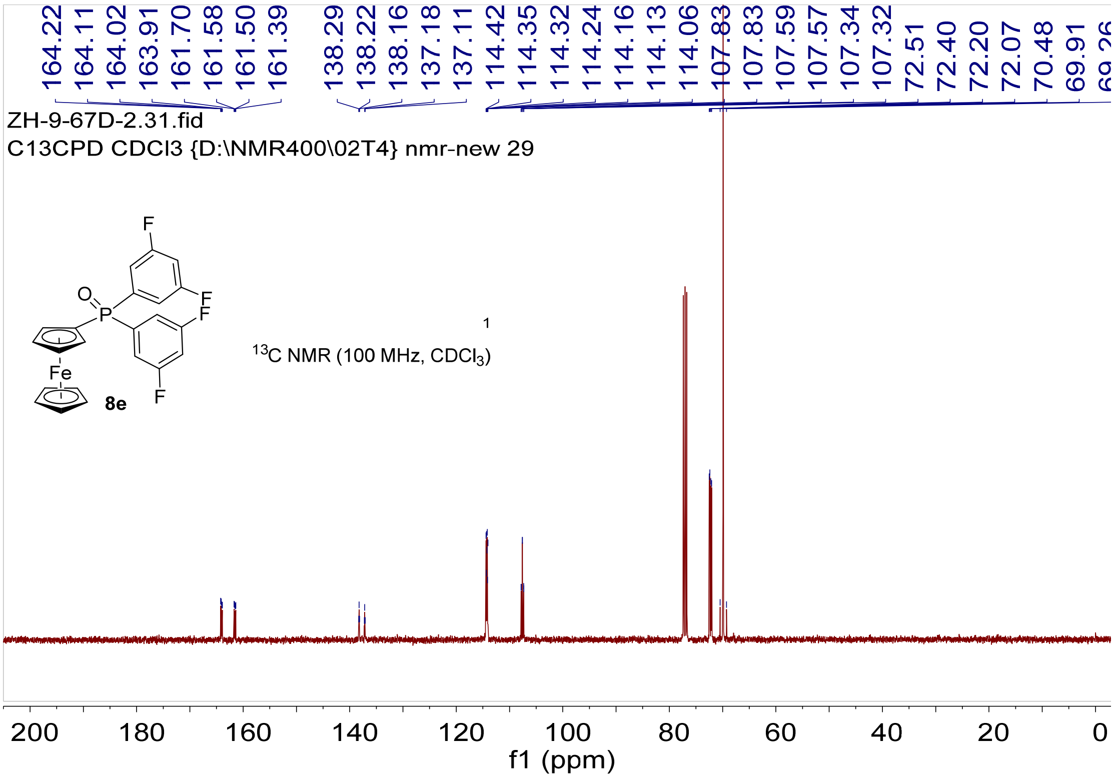

Supplementary Figure 114. ^{13}**C NMR spectra of compound 8e**

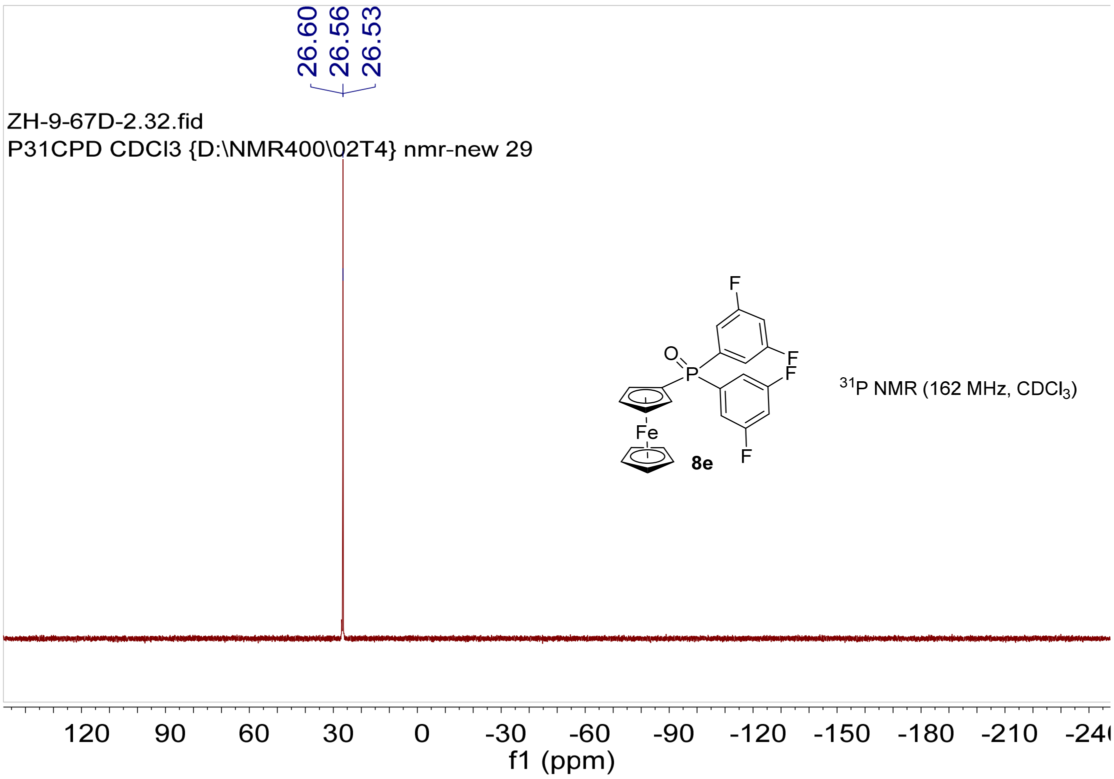

Supplementary Figure 115. ^{31}P NMR spectra of compound 8e

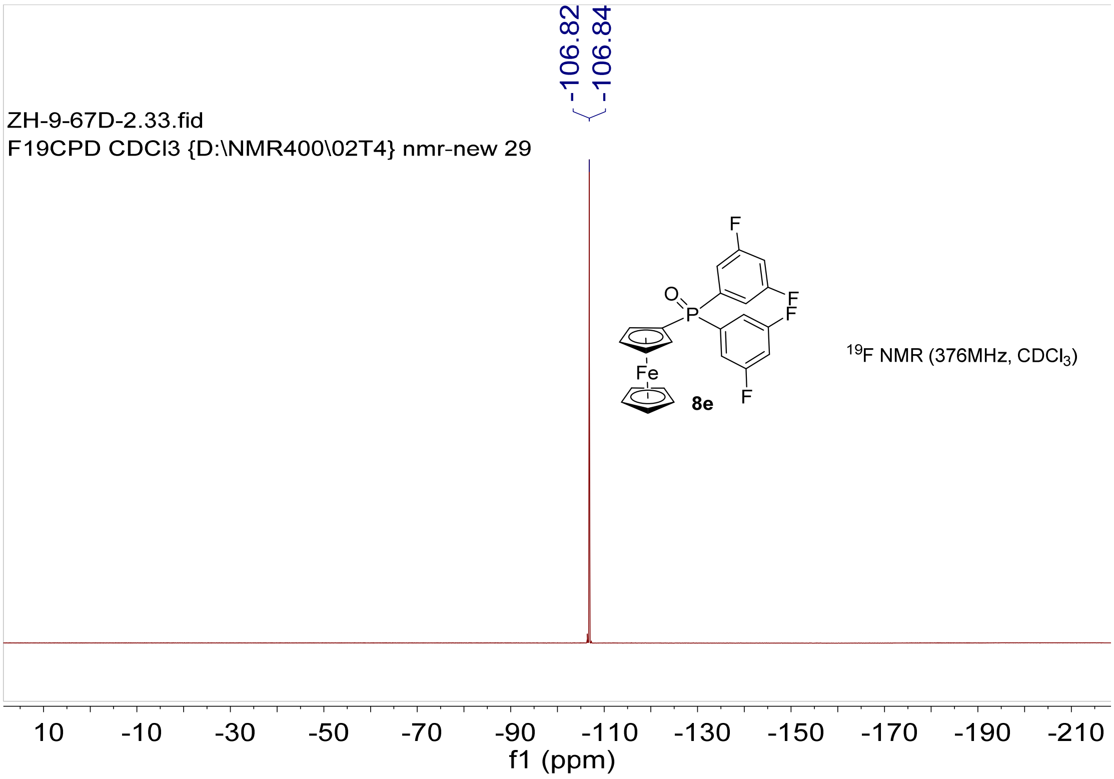

Supplementary Figure 116. ^{31}P NMR spectra of compound 8e

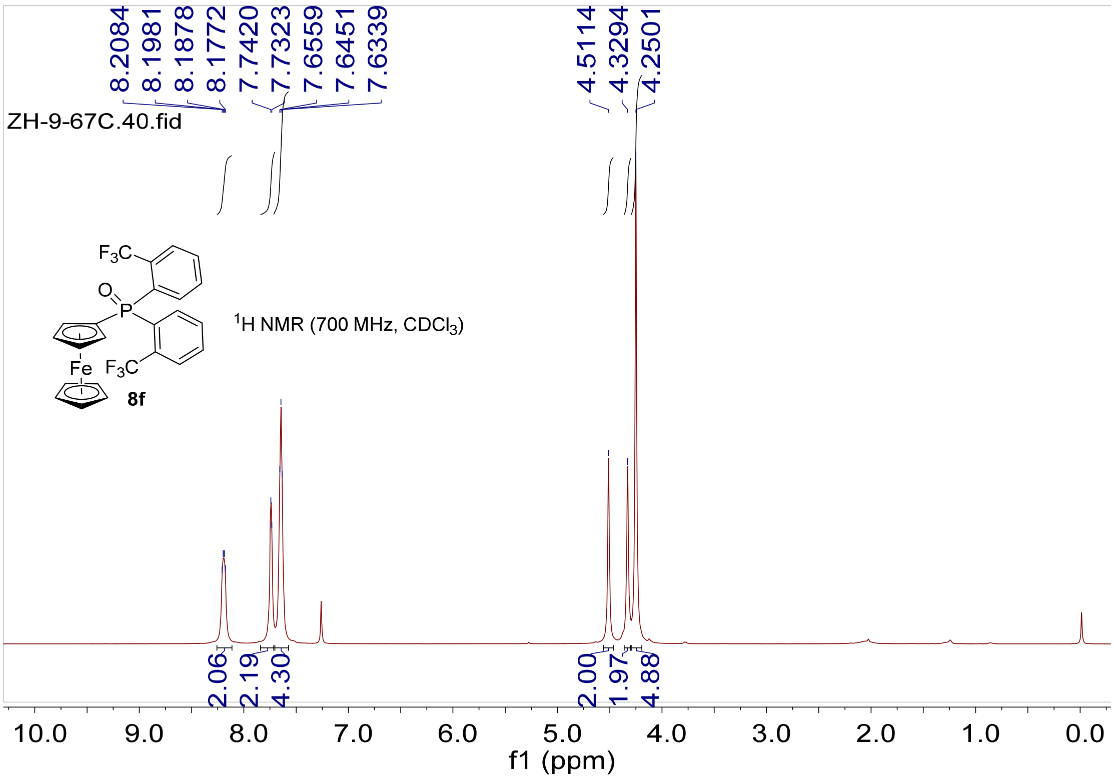

Supplementary Figure 117. ^{1}H NMR spectra of compound 8f

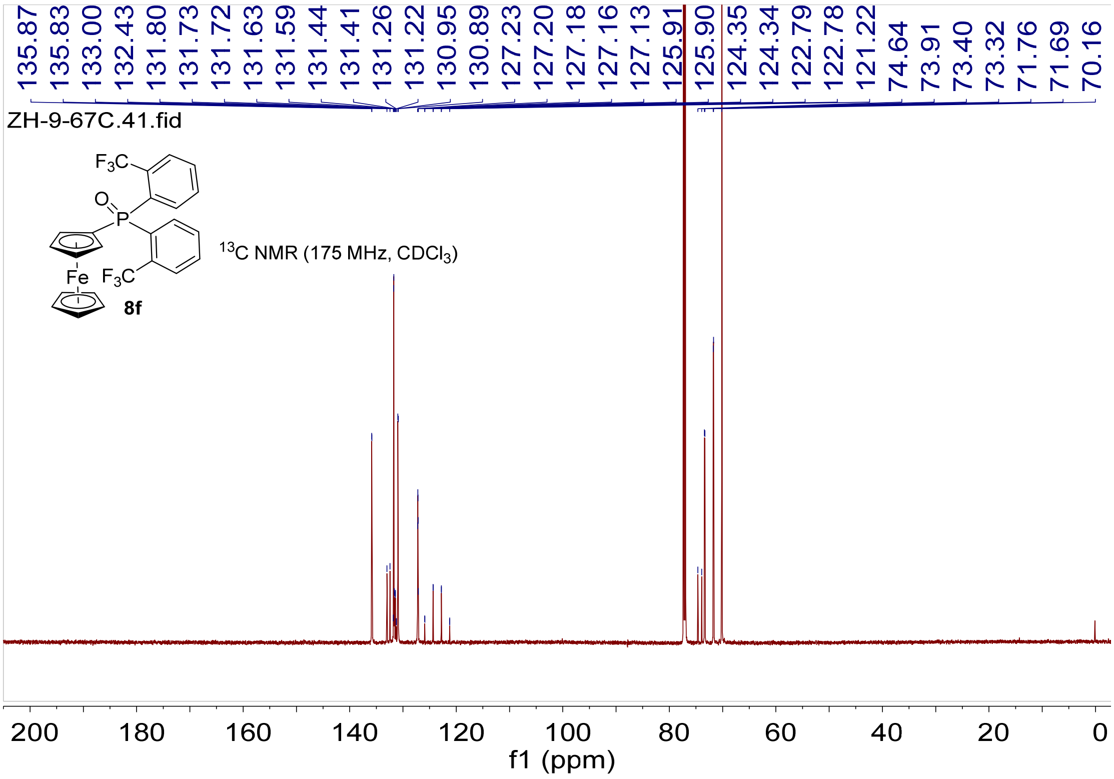

Supplementary Figure 118. ^{13}C NMR spectra of compound 8f

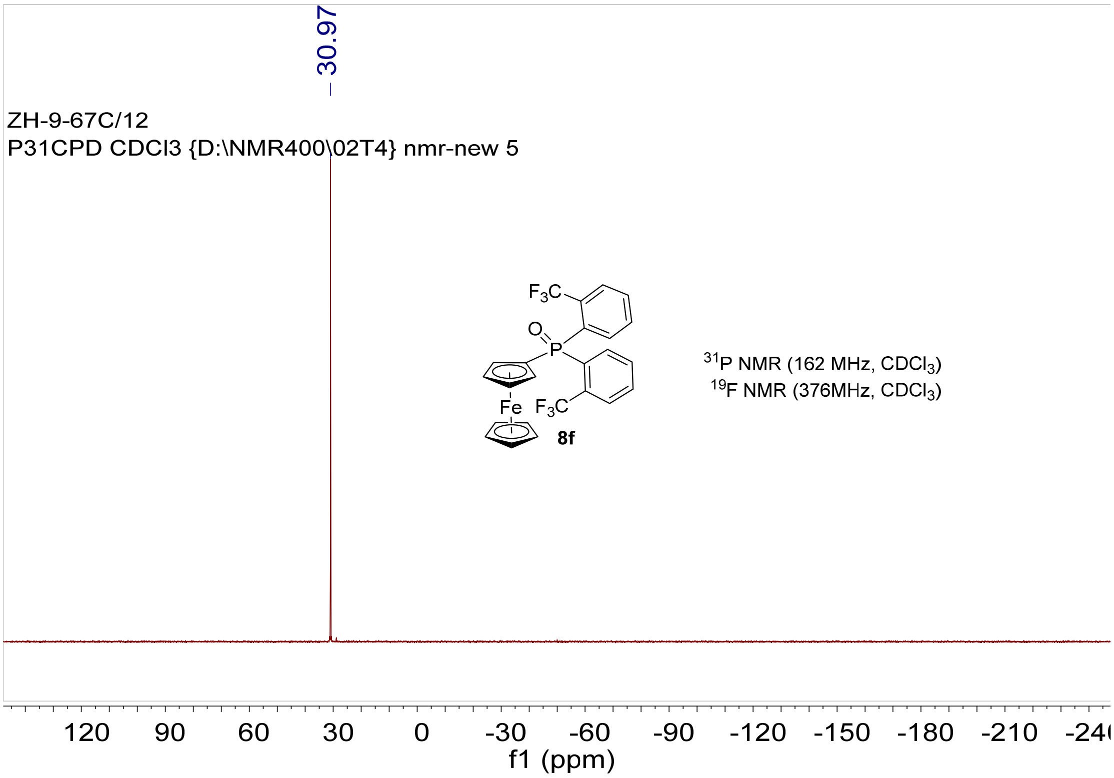

Supplementary Figure 119. ^{31}P NMR spectra of compound 8f

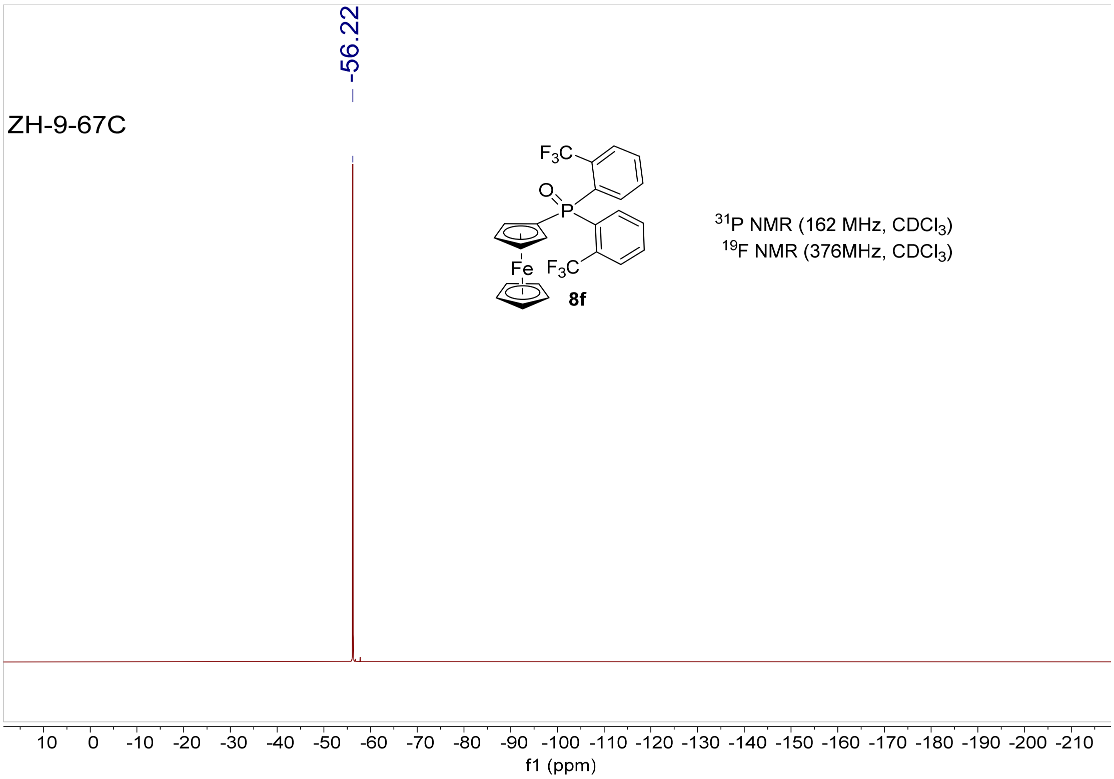

Supplementary Figure 120. ^{19}F NMR spectra of compound 8f

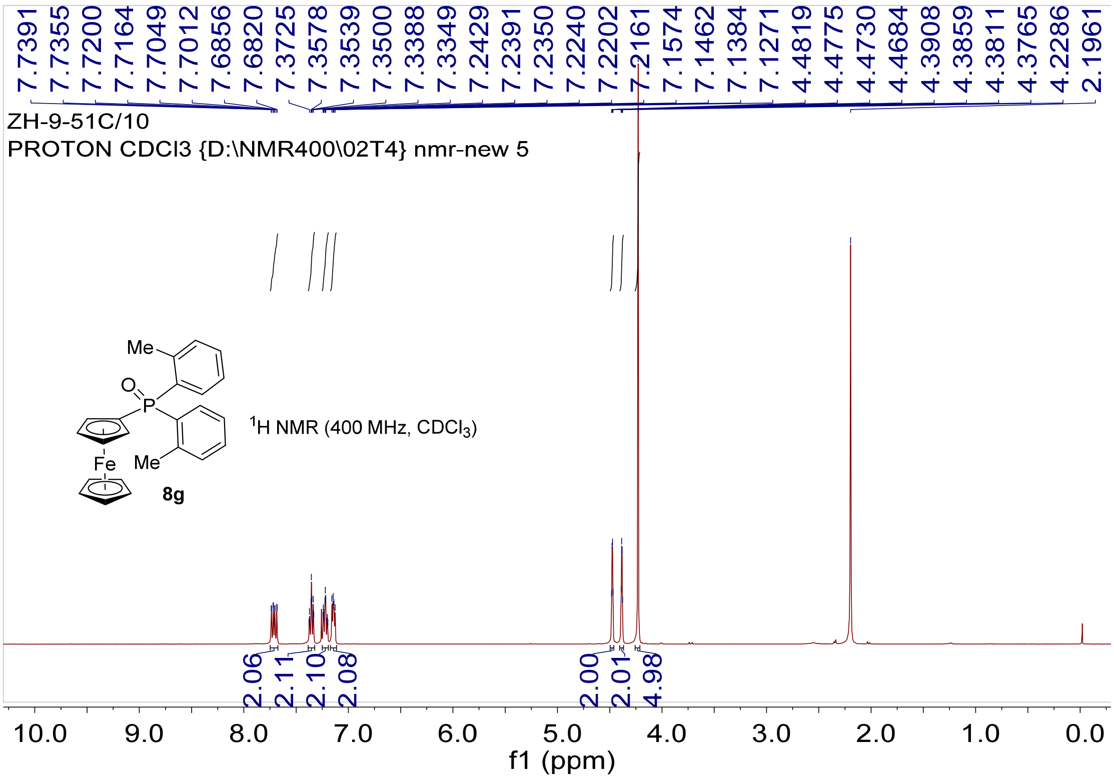

Supplementary Figure 121. ^{1}**H NMR spectra of compound 8g**

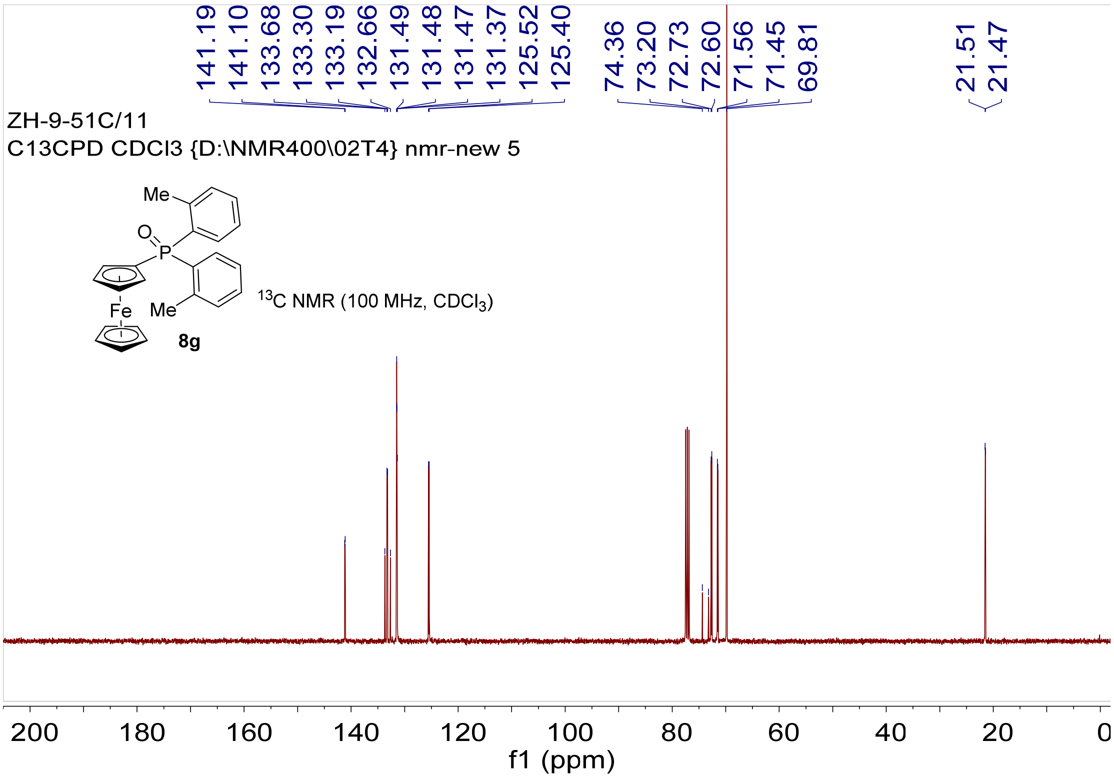

Supplementary Figure 122. ^{13}**C NMR spectra of compound 8g**

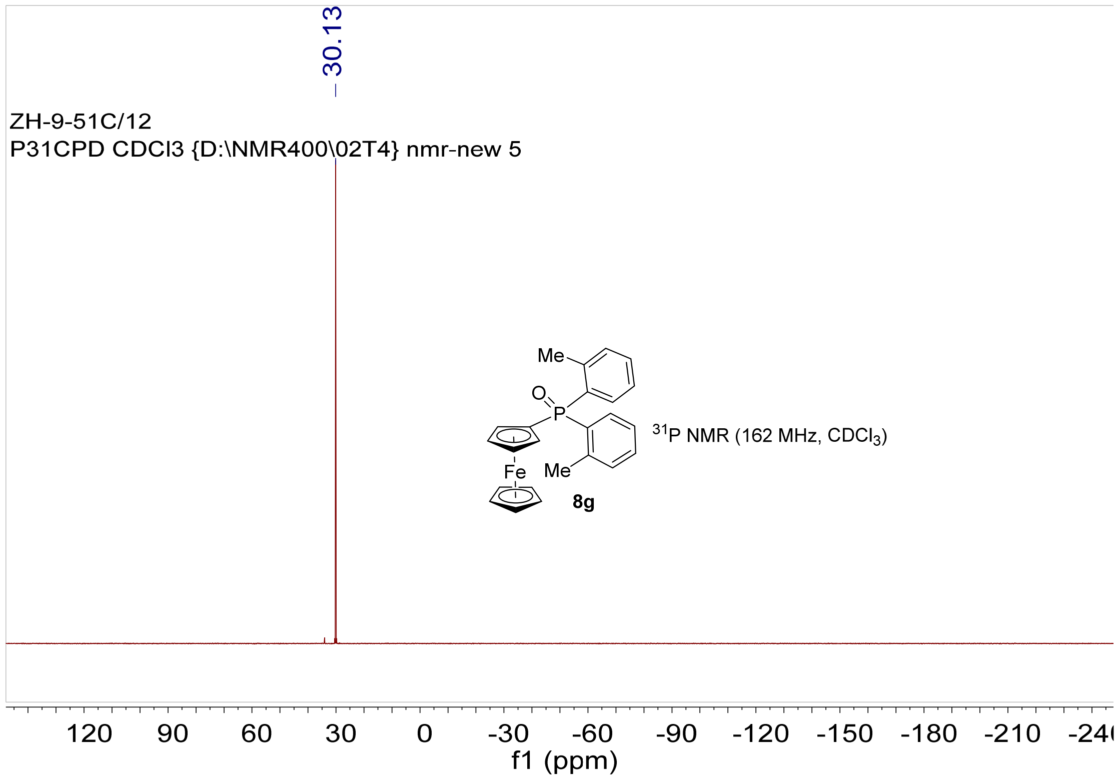

Supplementary Figure 123. ^{31}**P NMR spectra of compound 8g**

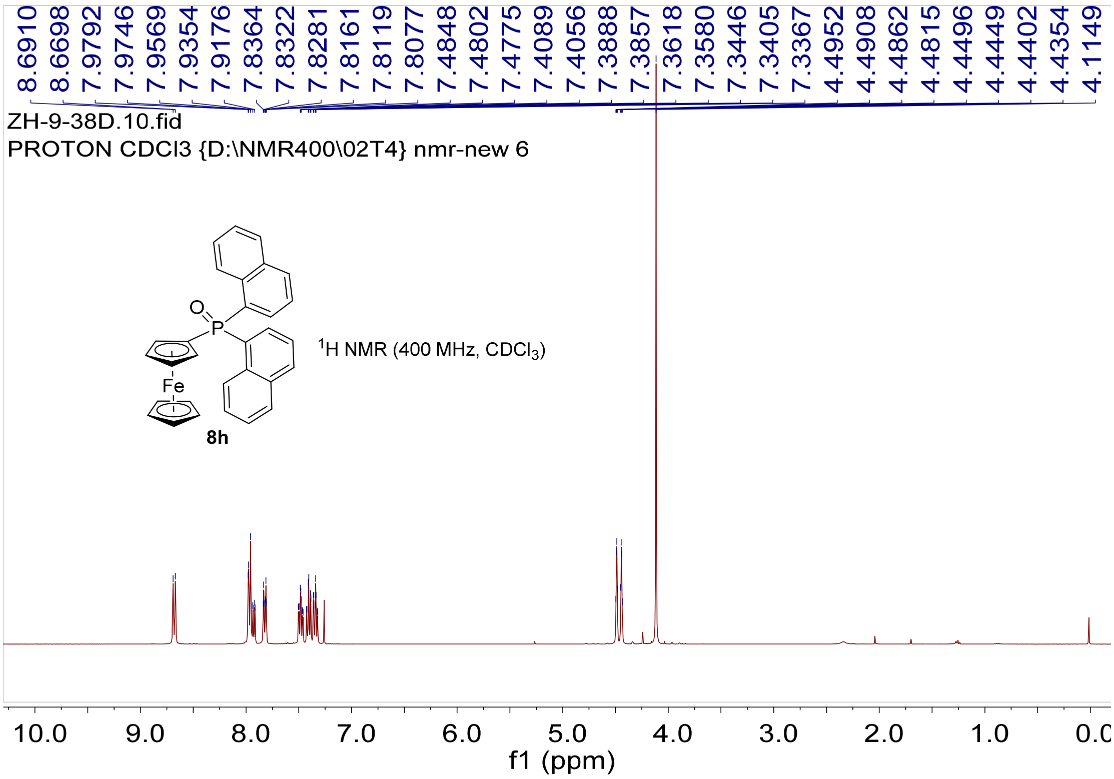

Supplementary Figure 124. ^{1}H NMR spectra of compound 8h

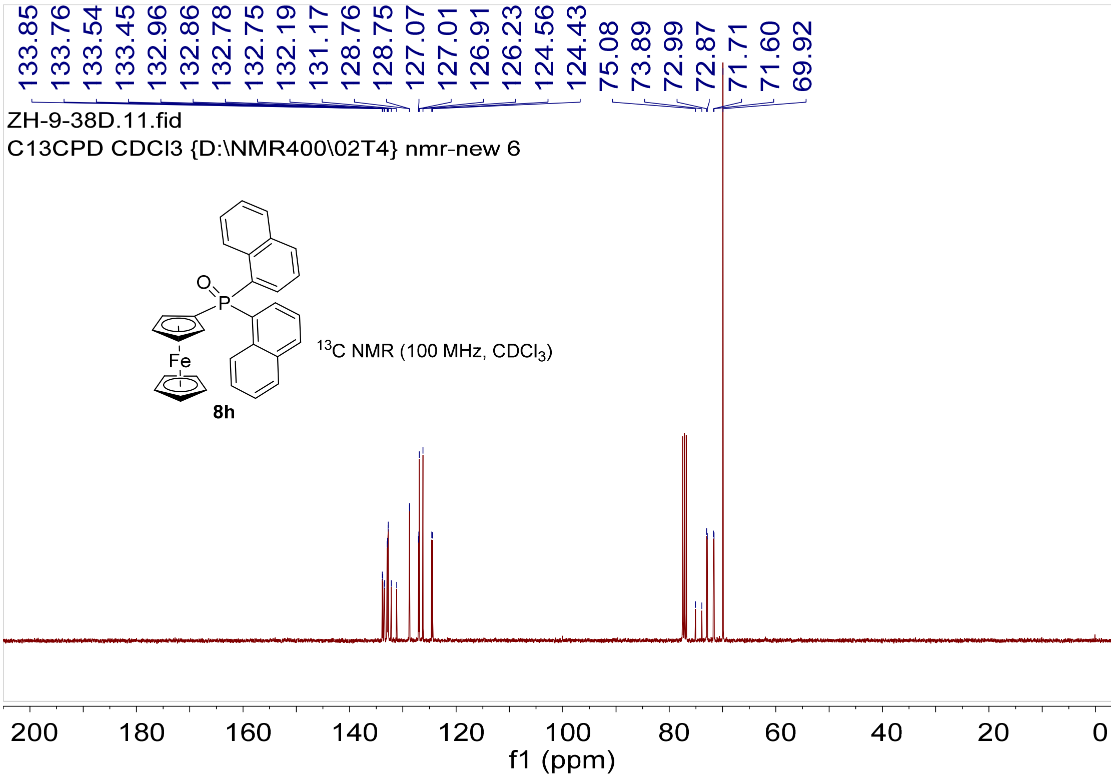

Supplementary Figure 125. ^{13}C NMR spectra of compound 8h

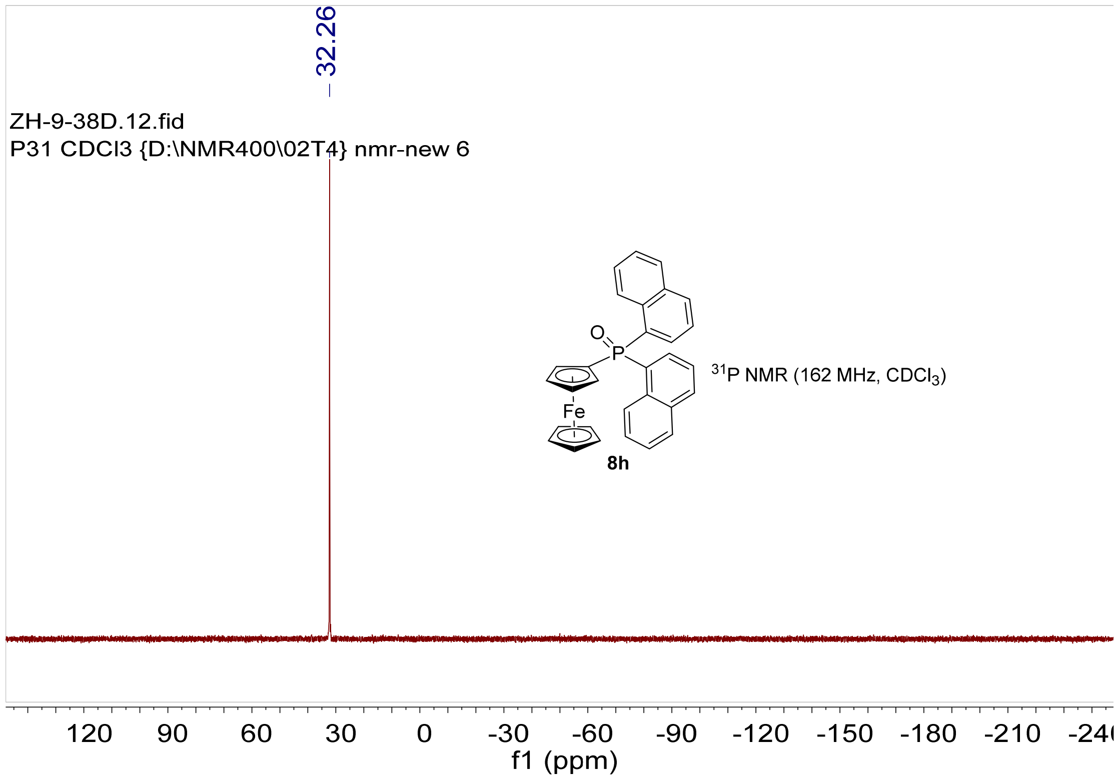

Supplementary Figure 126. ^{31}P NMR spectra of compound 8h

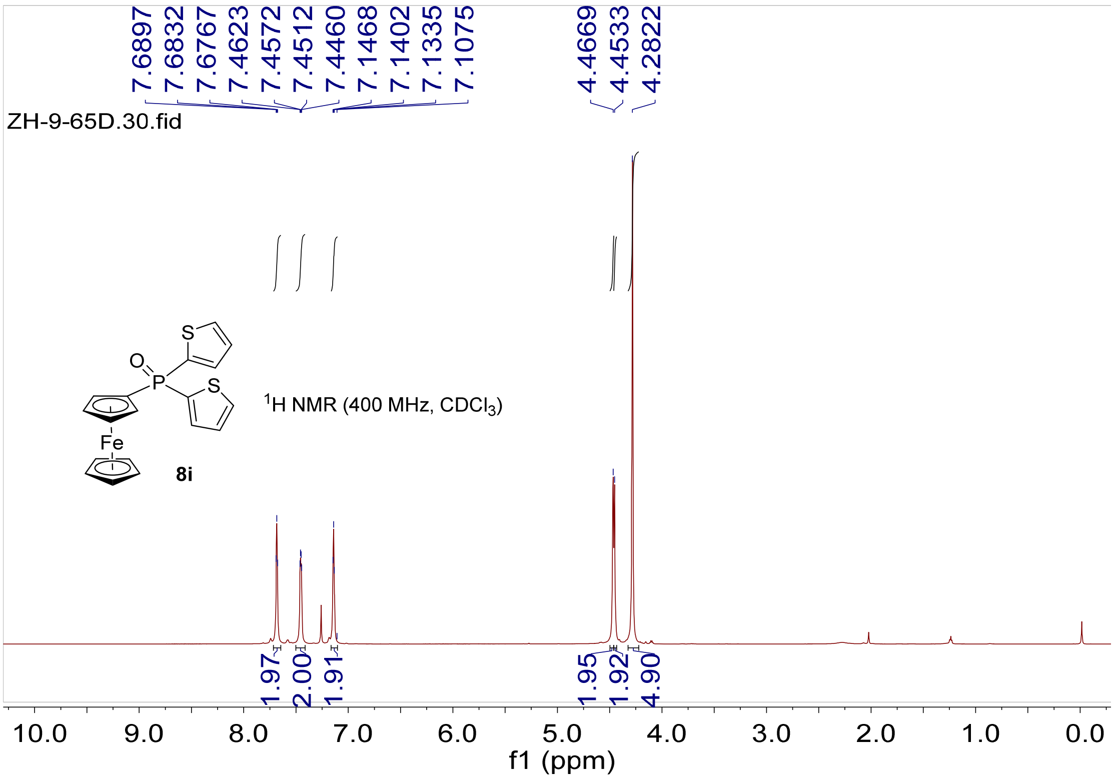

Supplementary Figure 127. ^{1}H NMR spectra of compound 8i

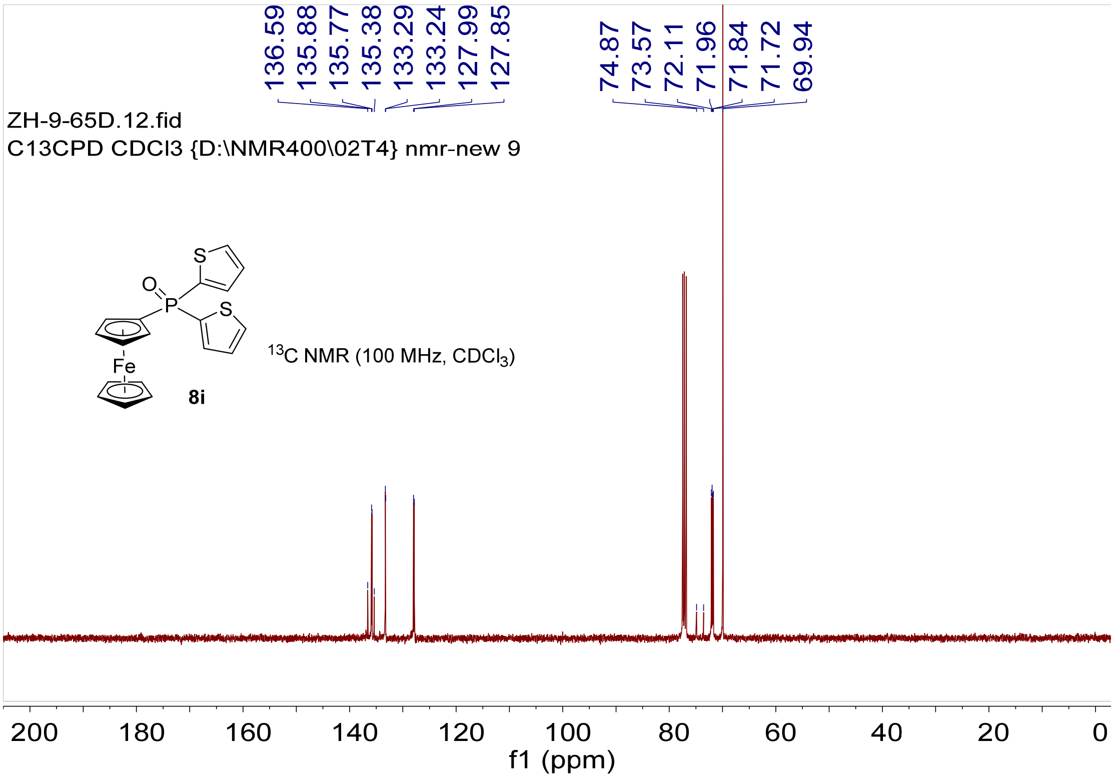

Supplementary Figure 128. ^{13}C NMR spectra of compound 8i

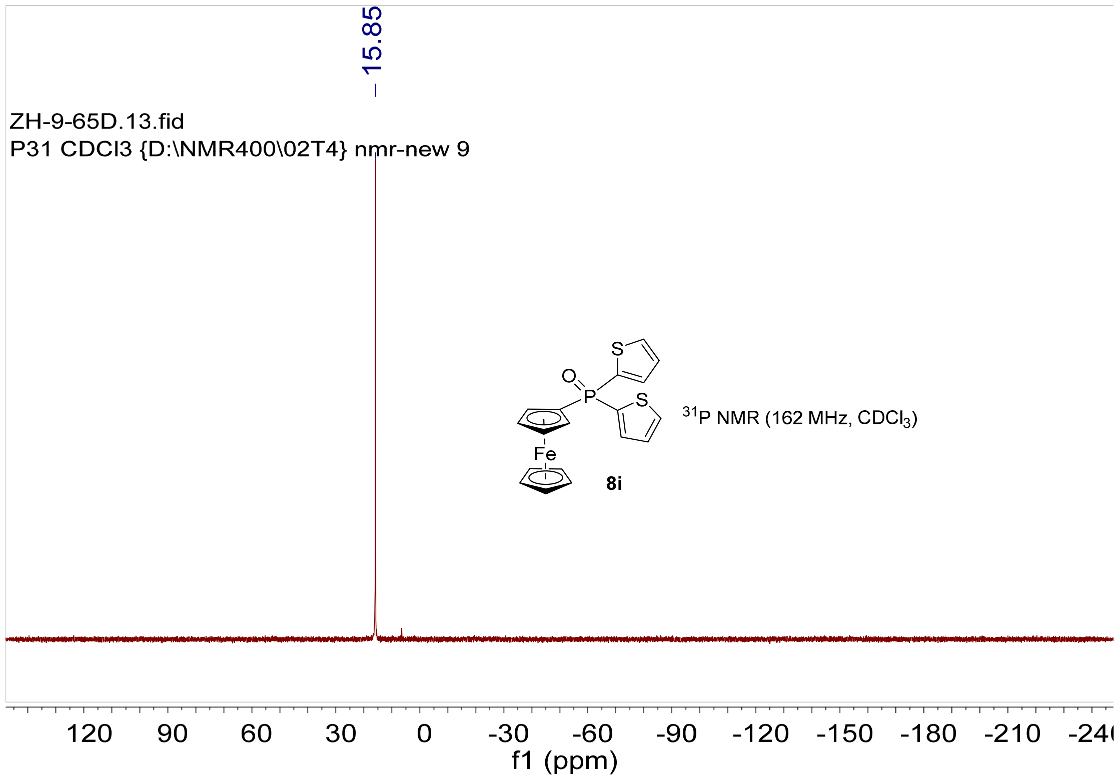

Supplementary Figure 129. ^{31}P NMR spectra of compound 8i

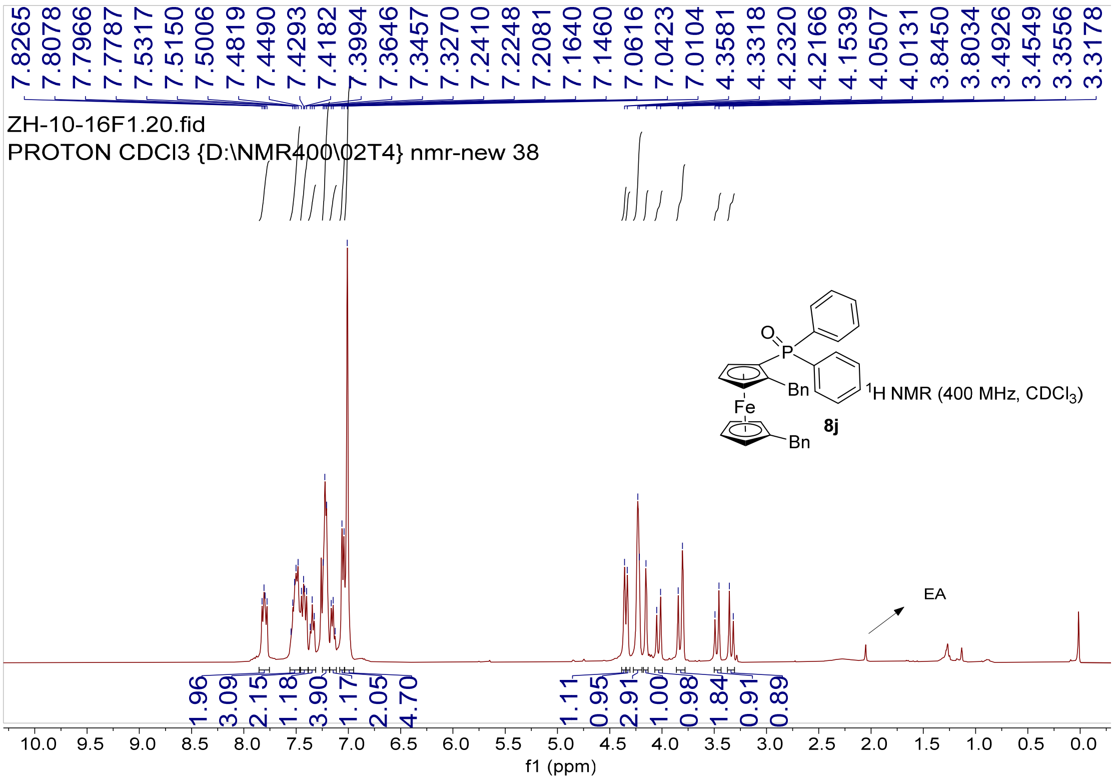

Supplementary Figure 130. ^{1}H NMR spectra of compound 8j

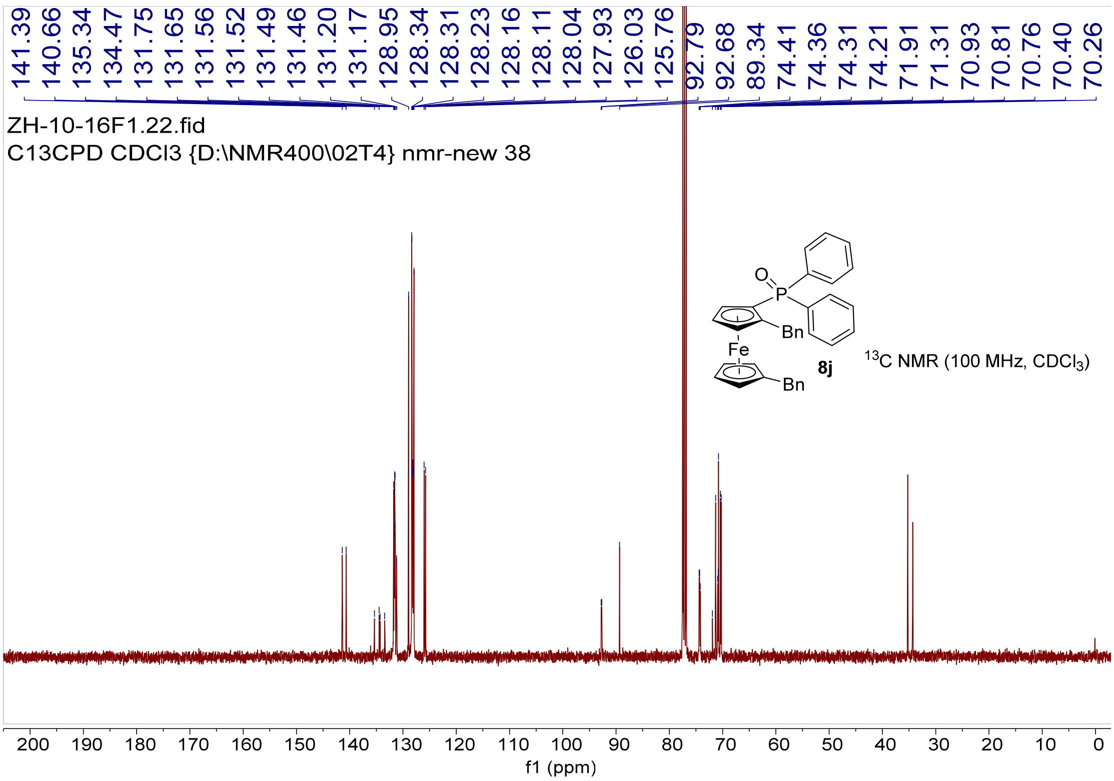

Supplementary Figure 131. ^{13}C NMR spectra of compound 8j

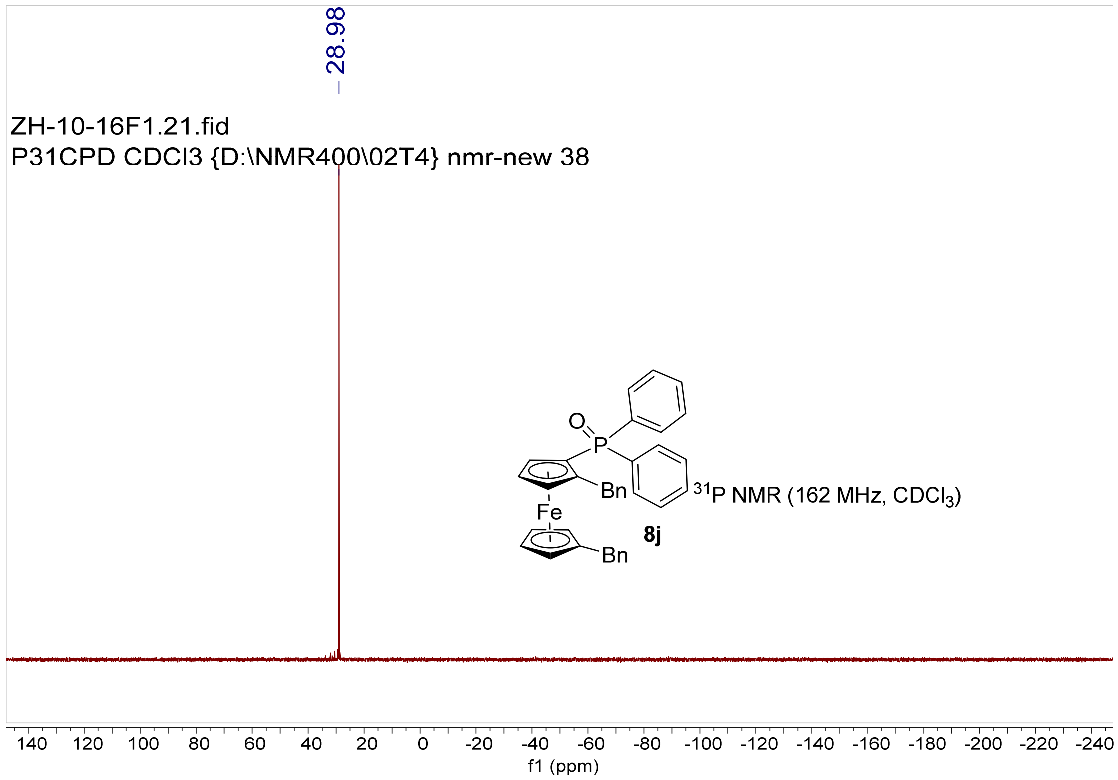

Supplementary Figure 132. ^{31}P NMR spectra of compound 8j

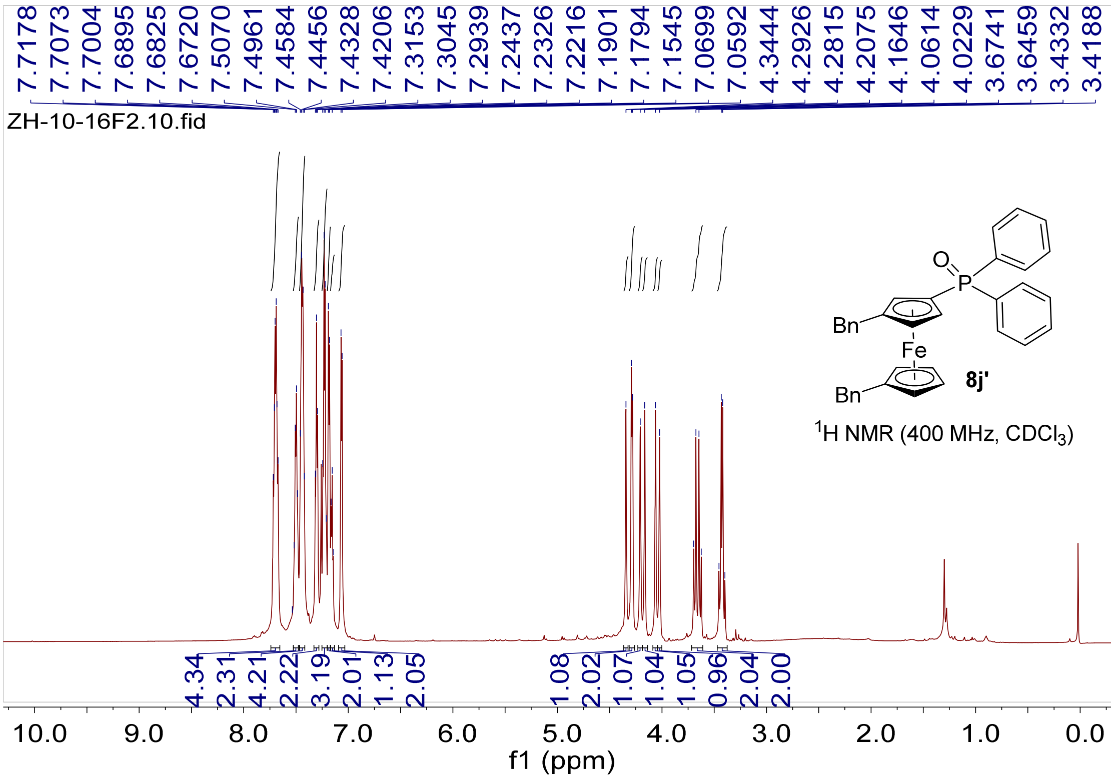

Supplementary Figure 133. ^{1}H NMR spectra of compound 8j'

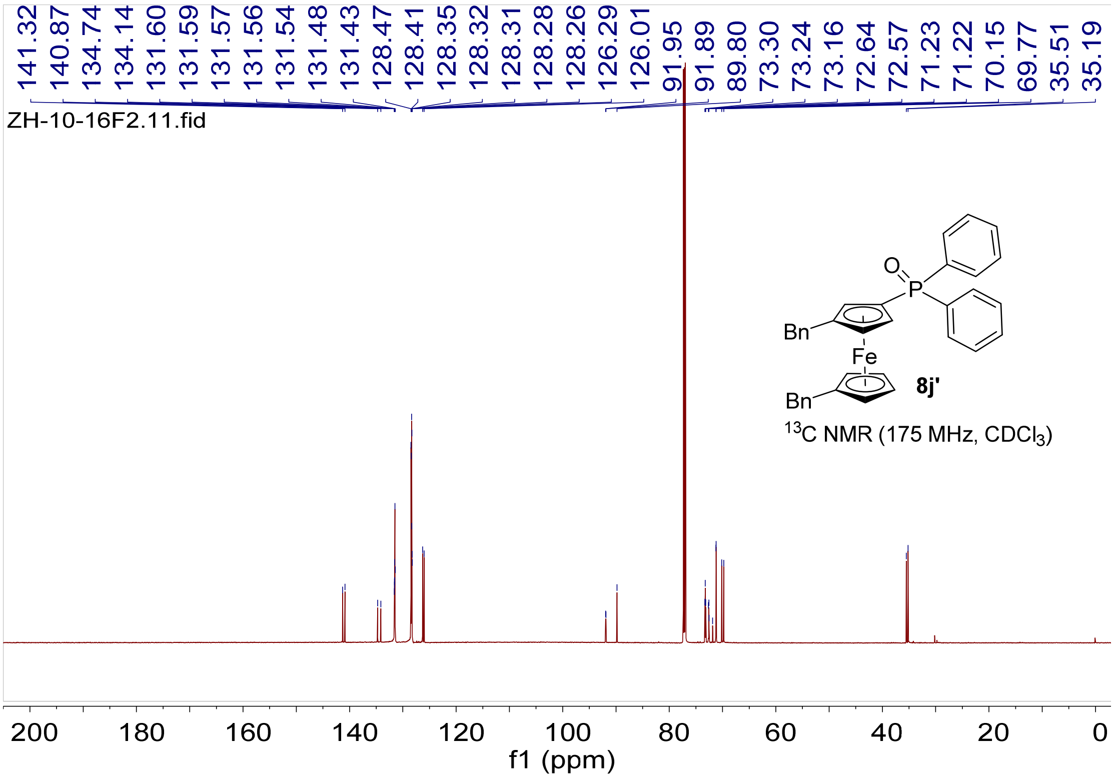

Supplementary Figure 134. ^{13}C NMR spectra of compound 8j'

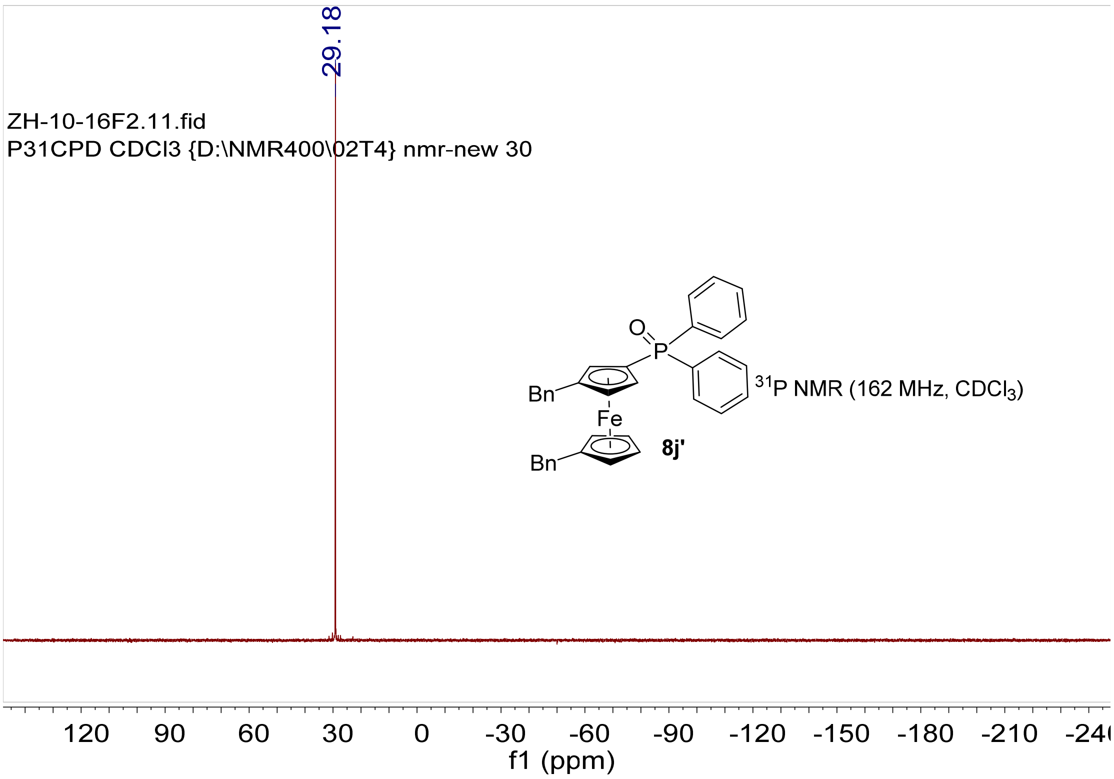

Supplementary Figure 135. ^{31}P NMR spectra of compound 8j'

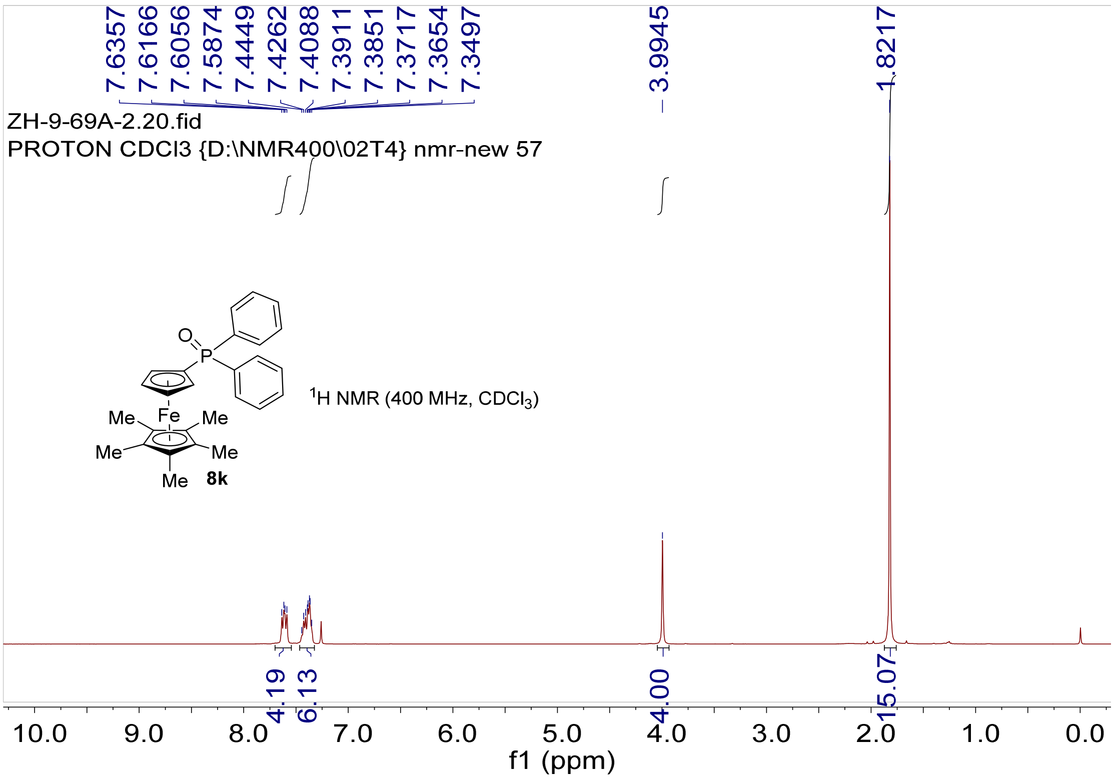

Supplementary Figure 136. ^{1}**H NMR spectra of compound 8k**

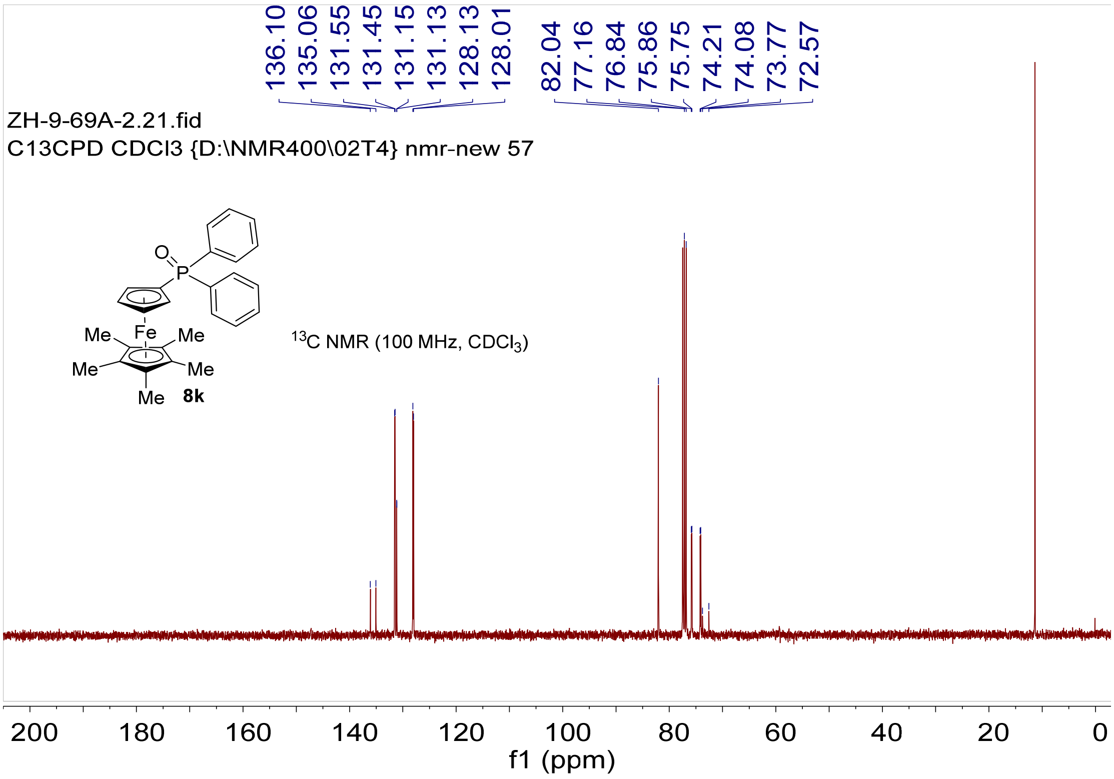

Supplementary Figure 137. ^{13}**C NMR spectra of compound 8k**

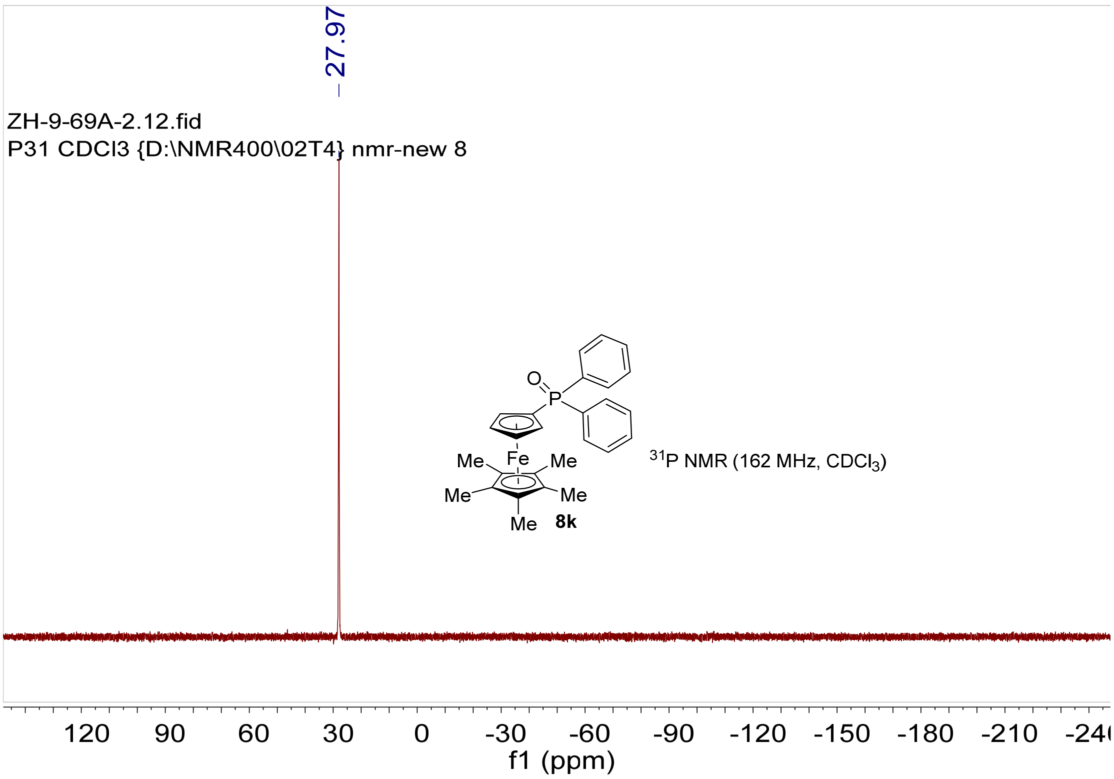

Supplementary Figure 138. ^{31}P NMR spectra of compound 8k

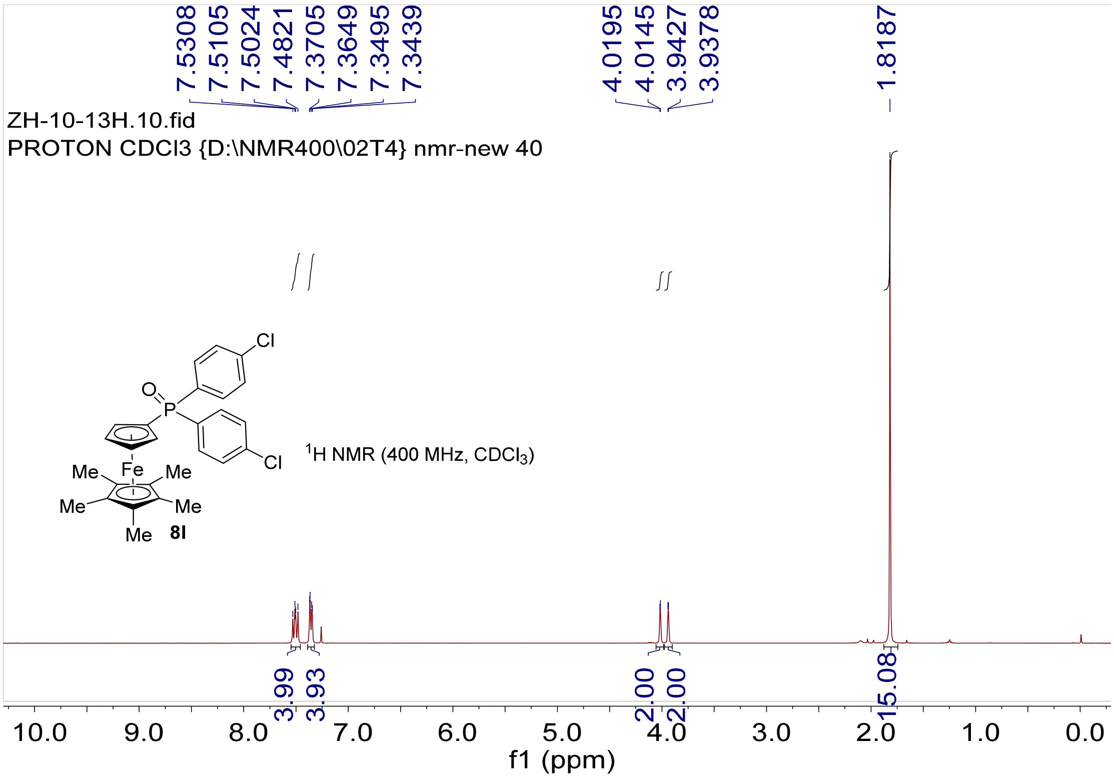

Supplementary Figure 139. ^{1}**H NMR spectra of compound 8l**

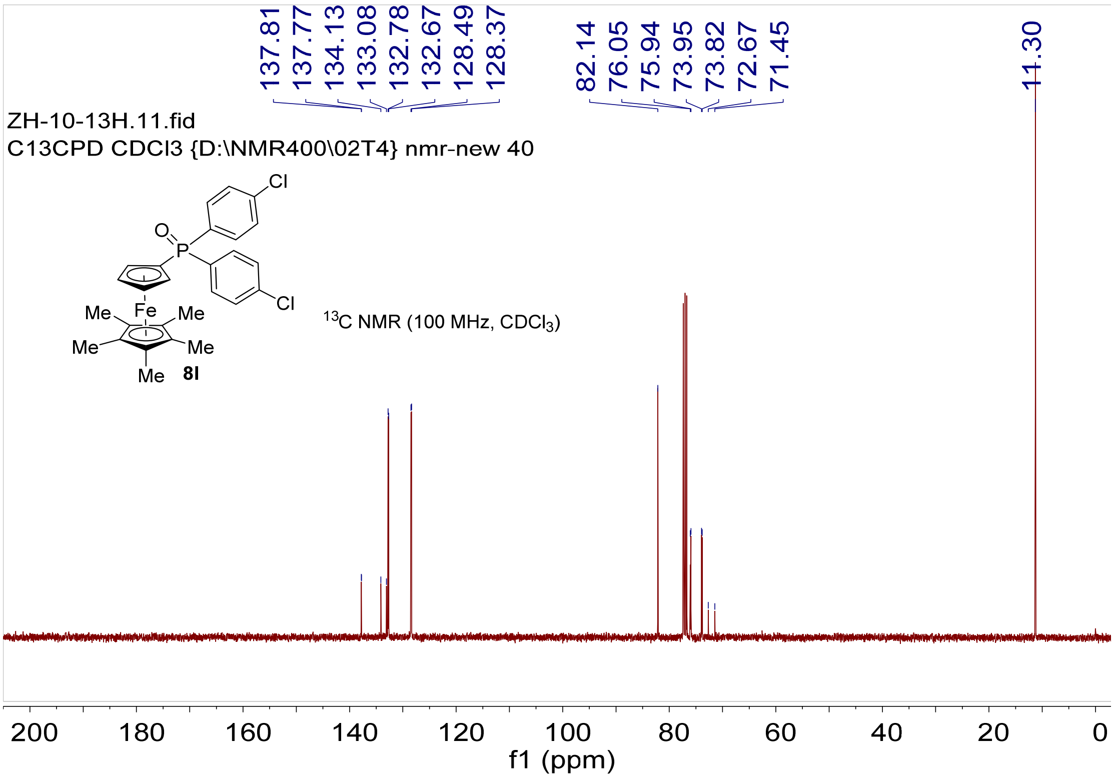

Supplementary Figure 140. ^{13}**C NMR spectra of compound 8l**

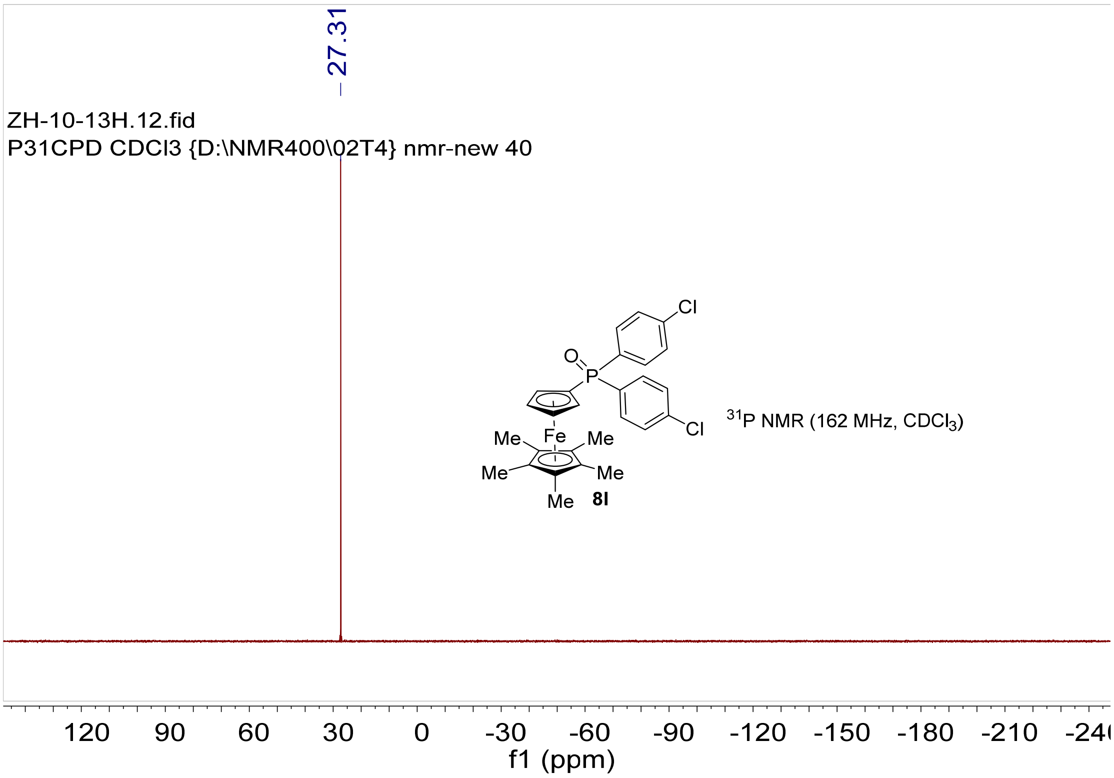

Supplementary Figure 141. ^{31}P NMR spectra of compound 8l

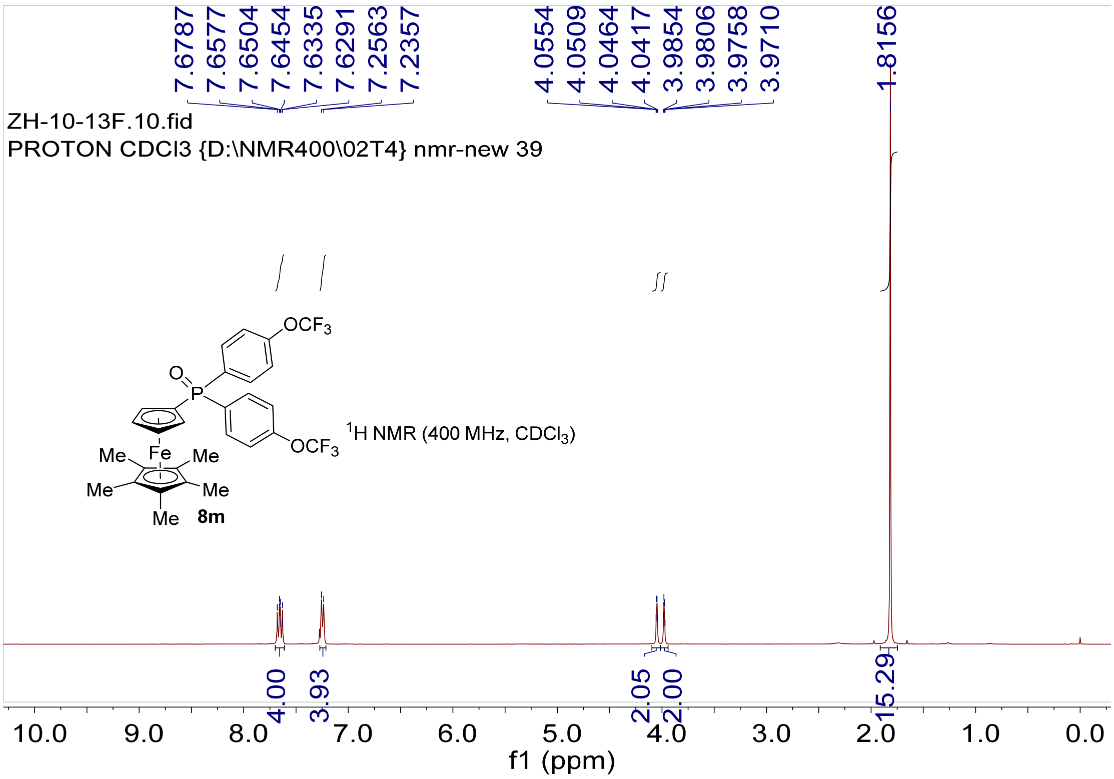

Supplementary Figure 142. ^{1}**H NMR spectra of compound 8m**

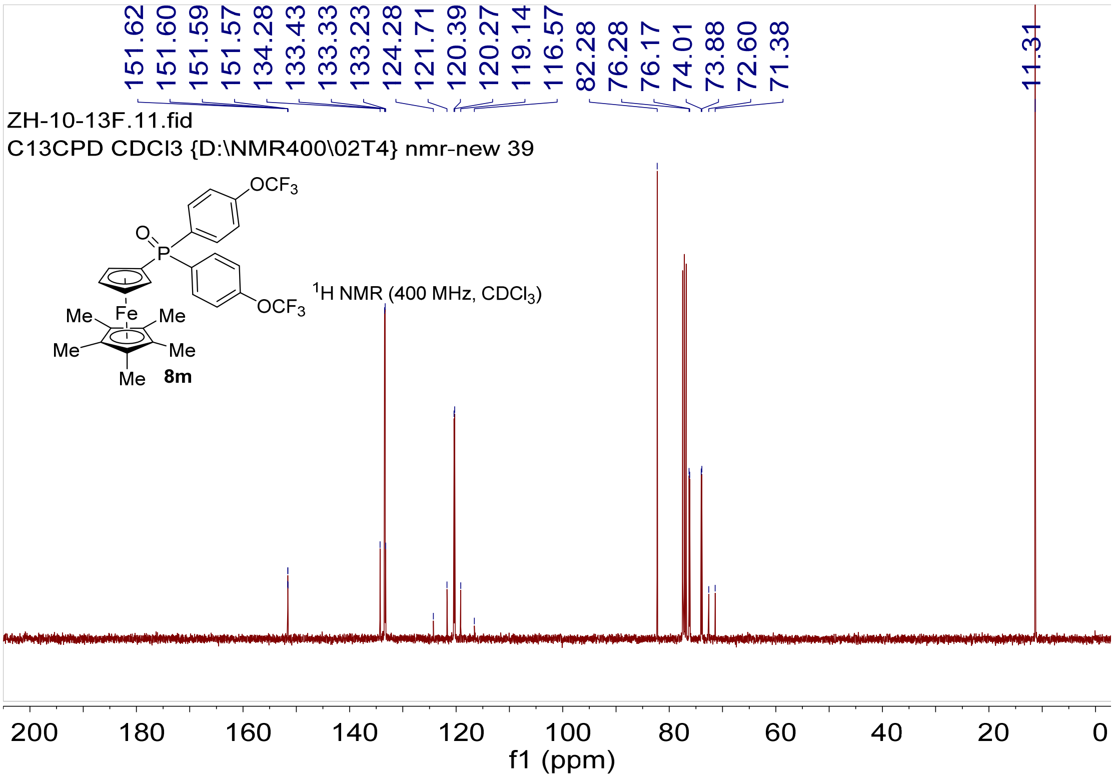

Supplementary Figure 143. ^{13}**C NMR spectra of compound 8m**

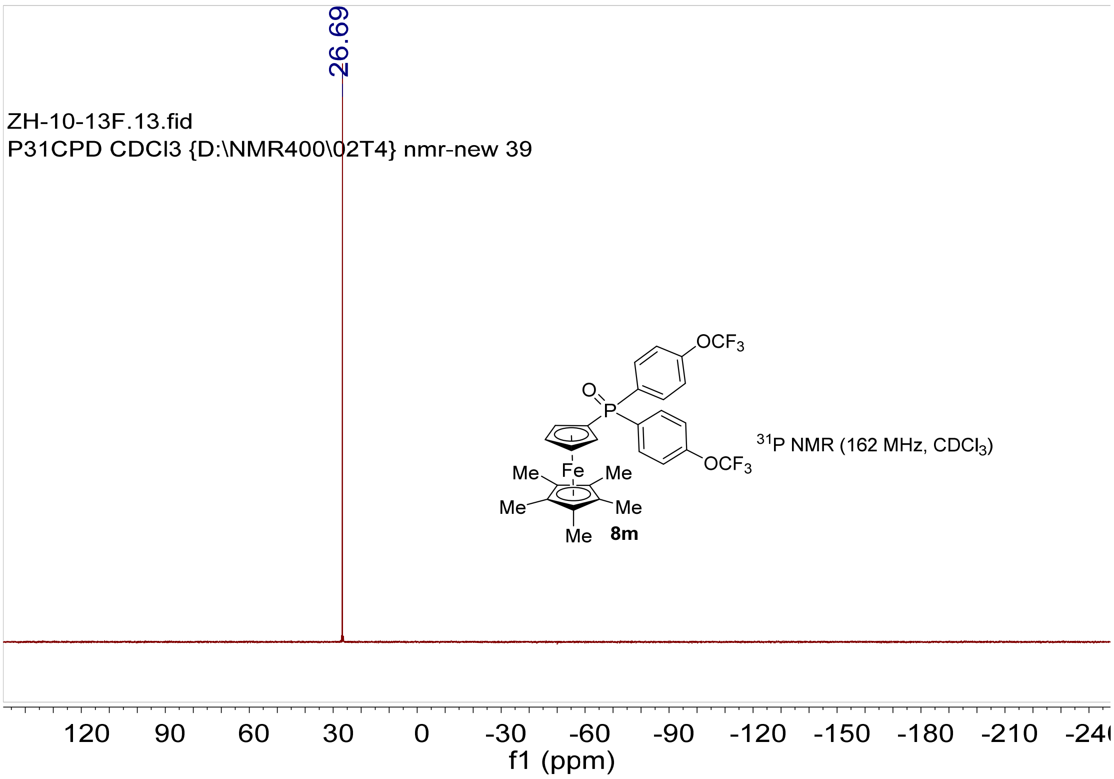

Supplementary Figure 144. ^{31}**P NMR spectra of compound 8m**

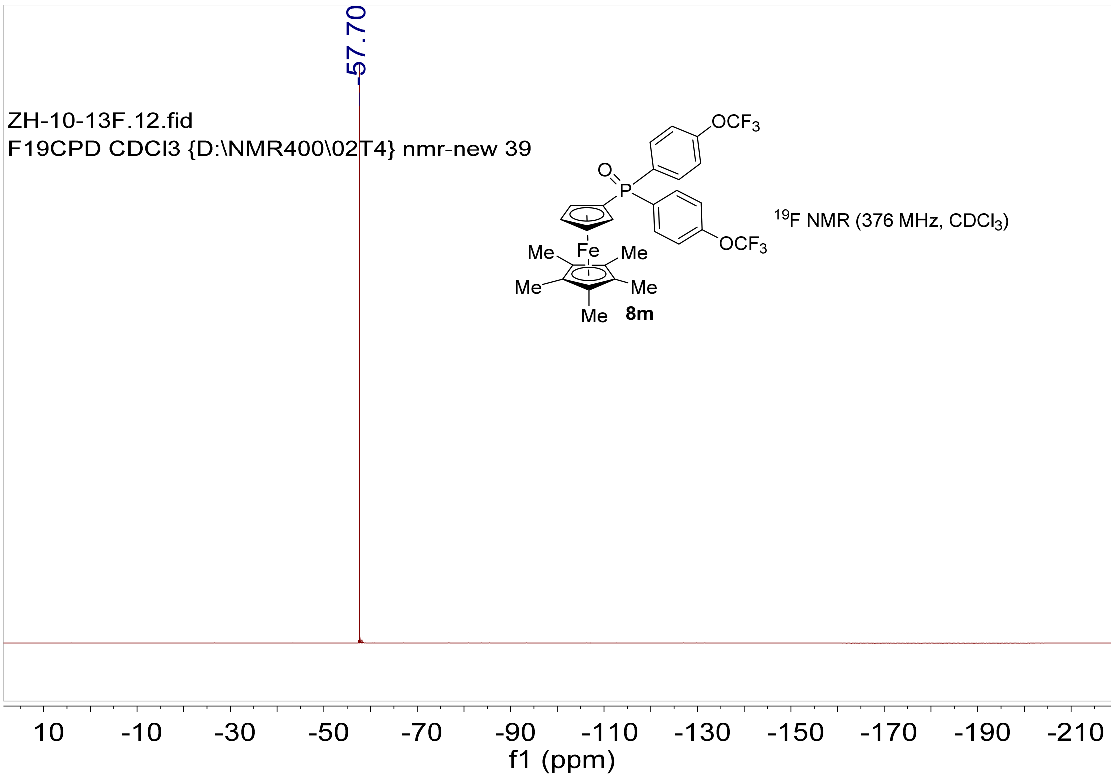

Supplementary Figure 145. ^{19}**F NMR spectra of compound 8m**

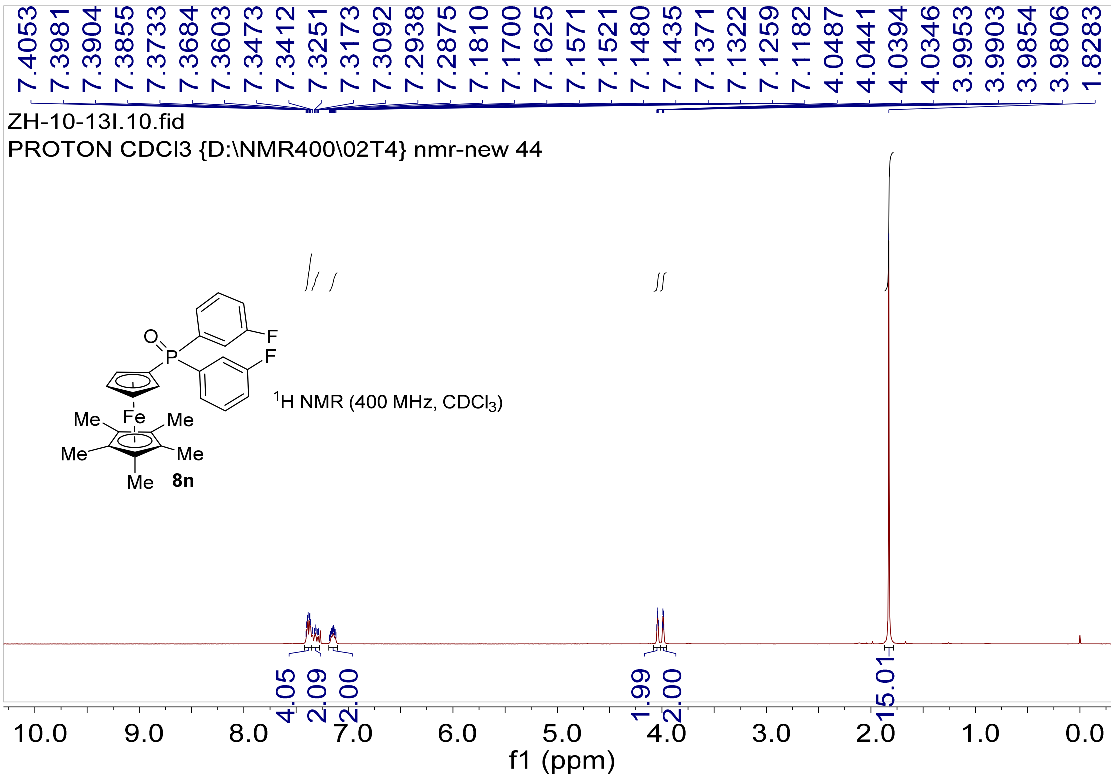

Supplementary Figure 146. ^{1}**H NMR spectra of compound 8n**

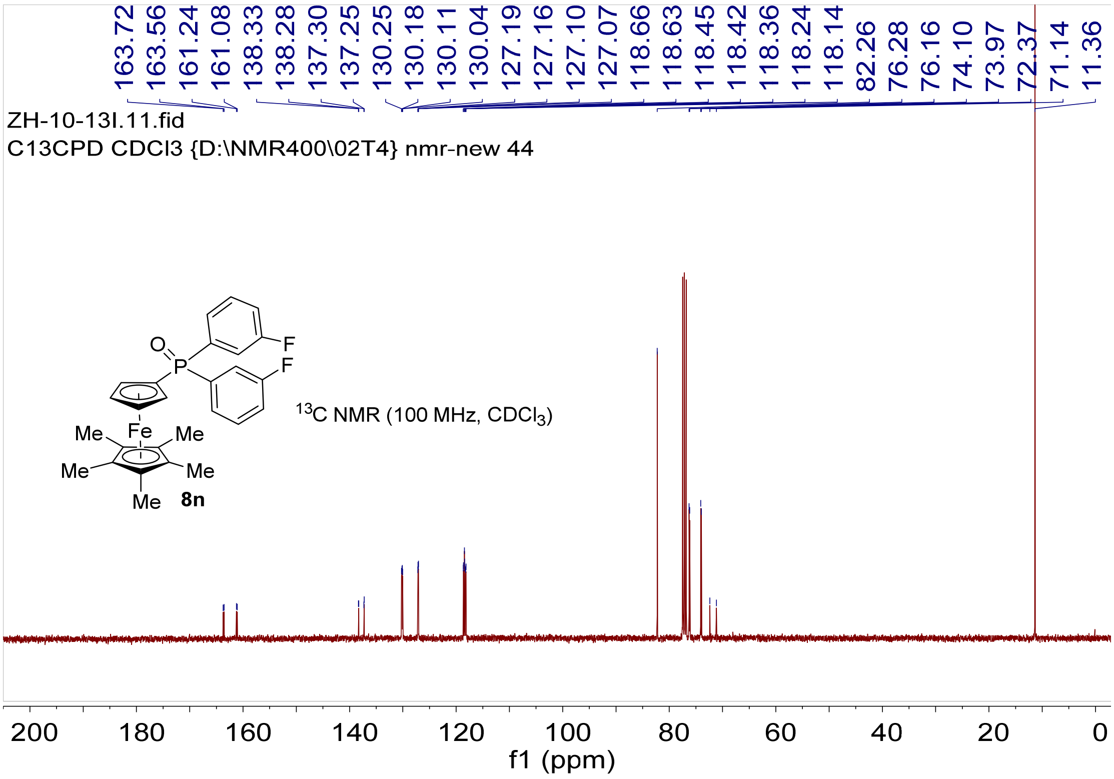

Supplementary Figure 147. ^{13}**C NMR spectra of compound 8n**

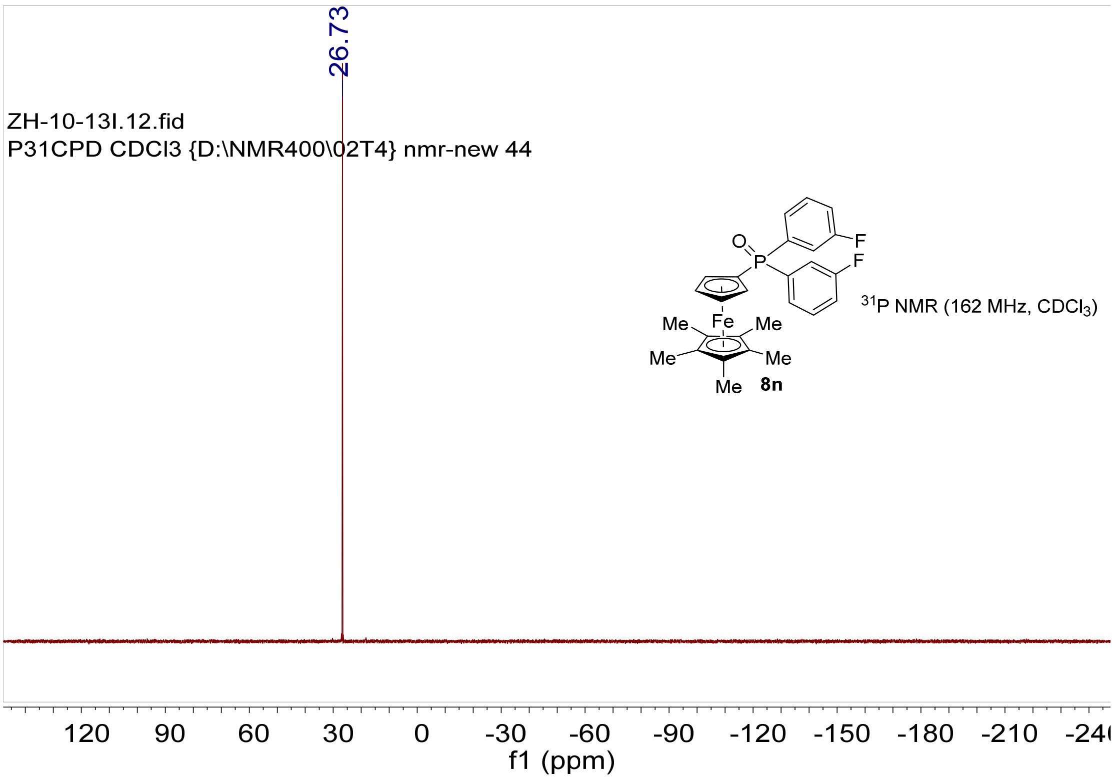

Supplementary Figure 148. ^{31}P NMR spectra of compound 8n

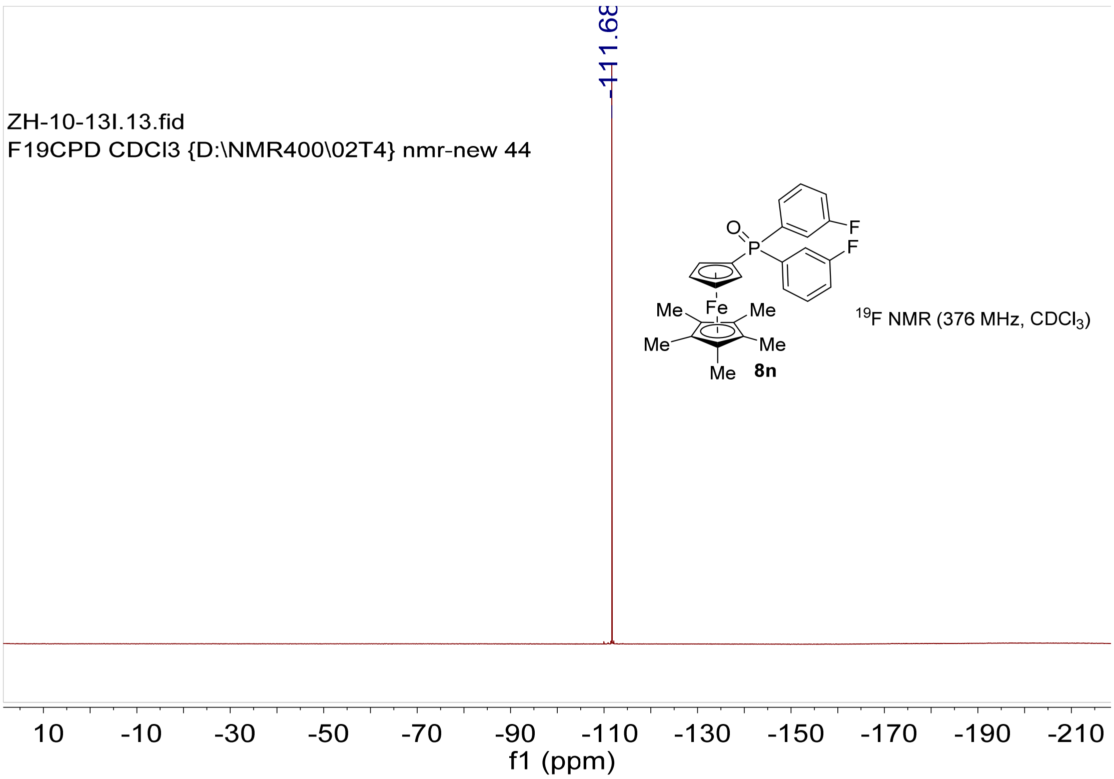

Supplementary Figure 149. ^{19}F NMR spectra of compound 8n

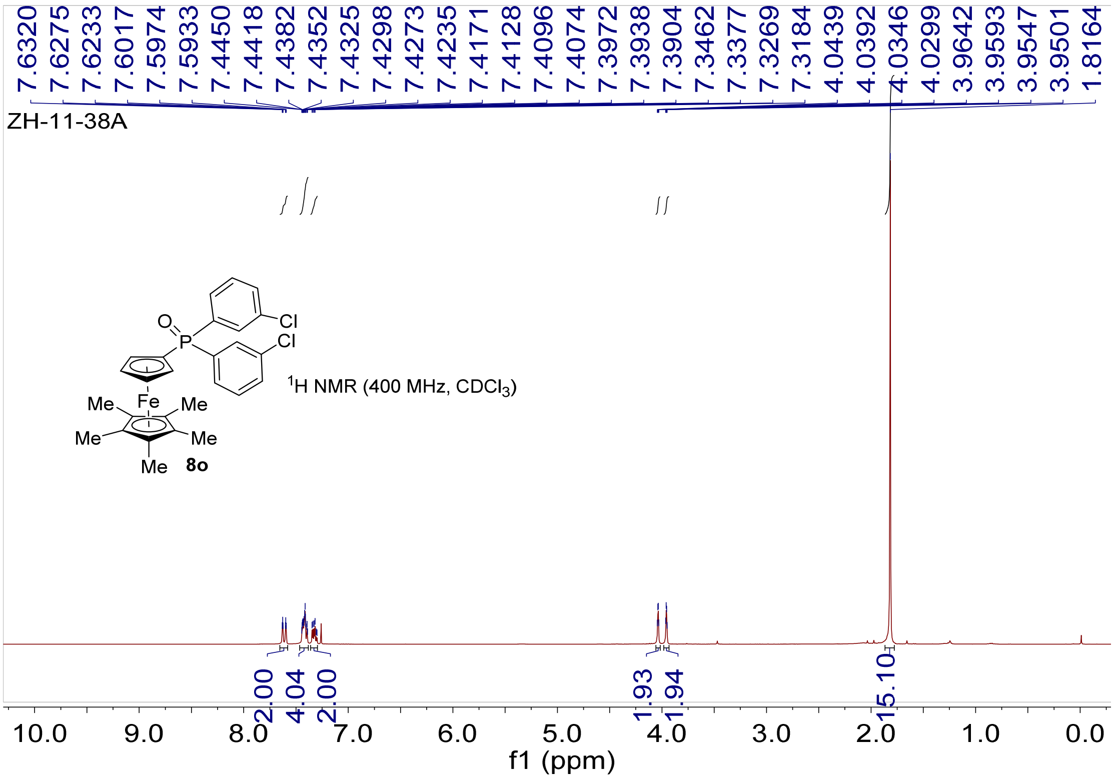

Supplementary Figure 150. ^{1}**H NMR spectra of compound 8o**

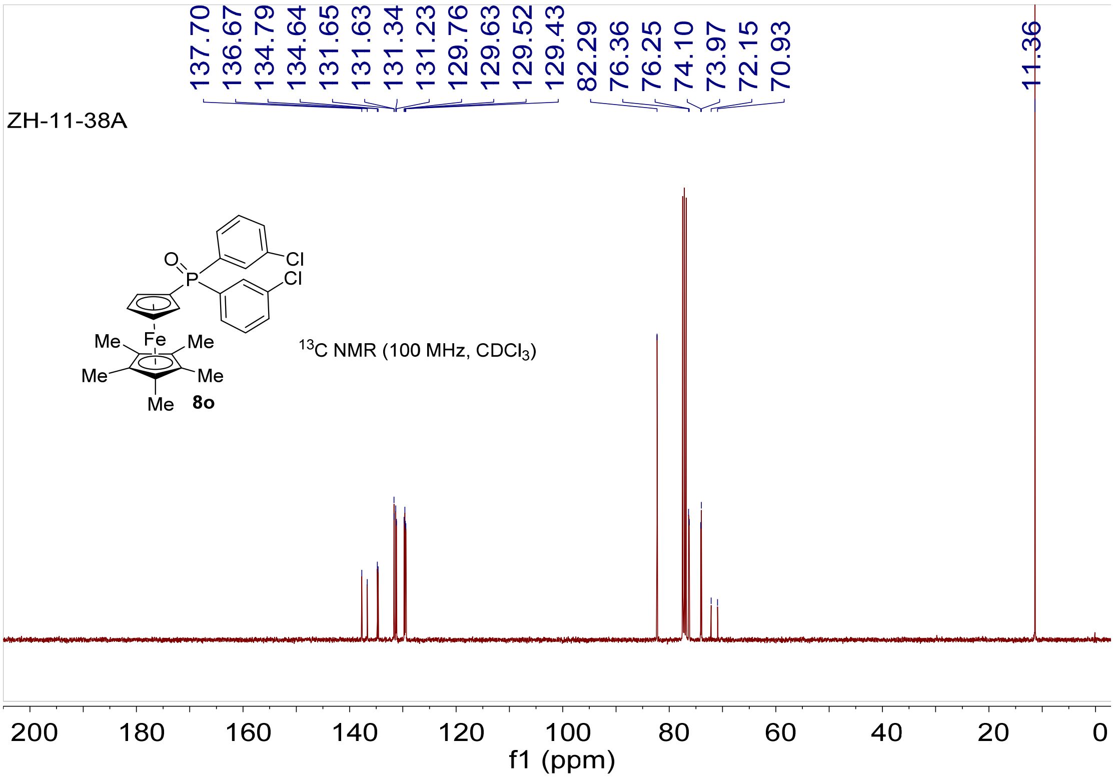

Supplementary Figure 151. ^{13}**C NMR spectra of compound 8o**

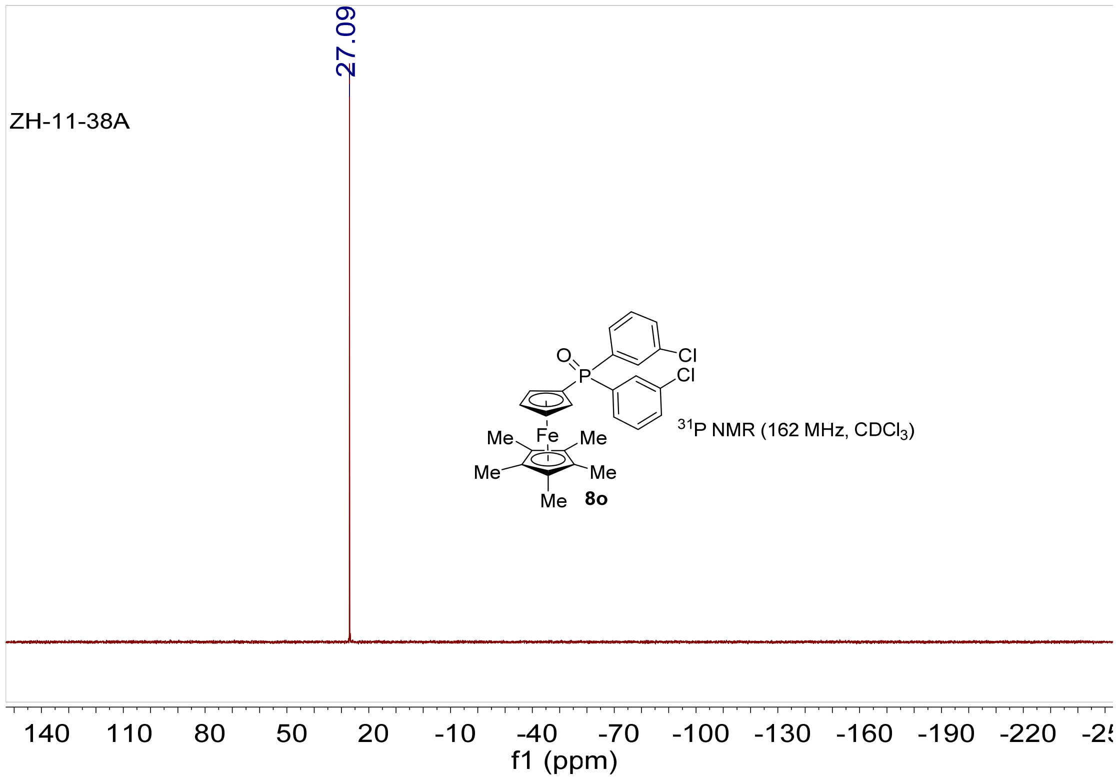

Supplementary Figure 152. ^{31}P NMR spectra of compound 8o

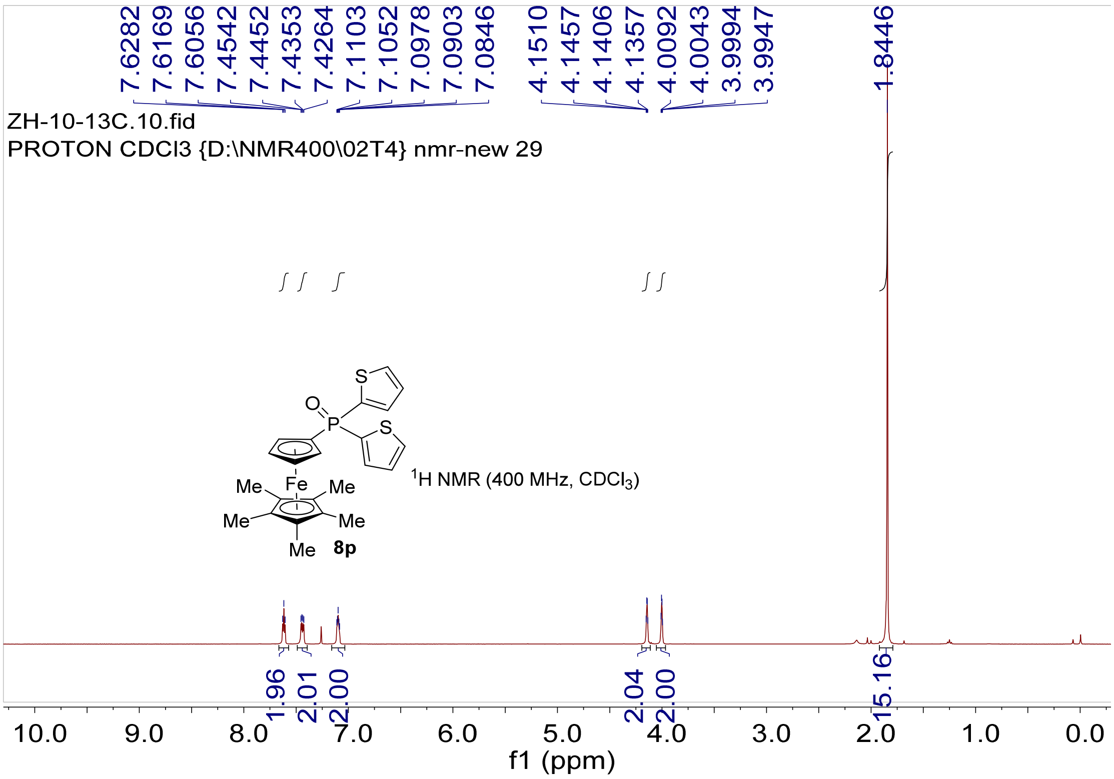

Supplementary Figure 153. ^{1}**H NMR spectra of compound 8p**

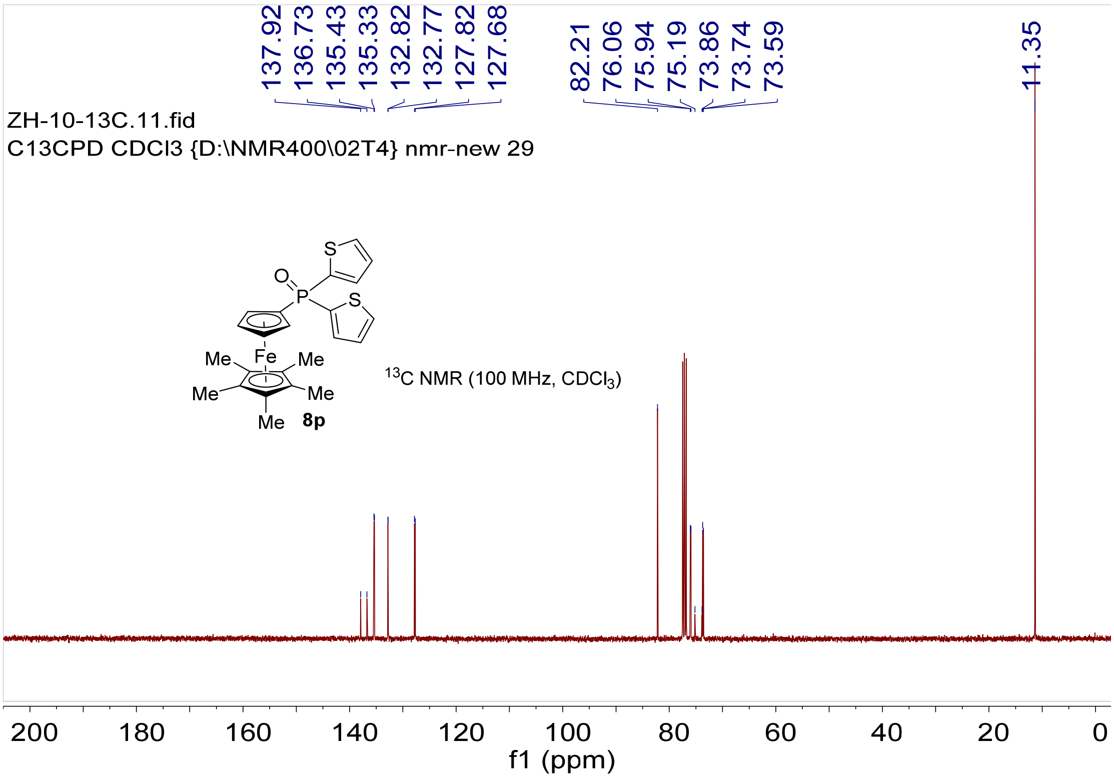

Supplementary Figure 154. ^{13}**C NMR spectra of compound 8p**

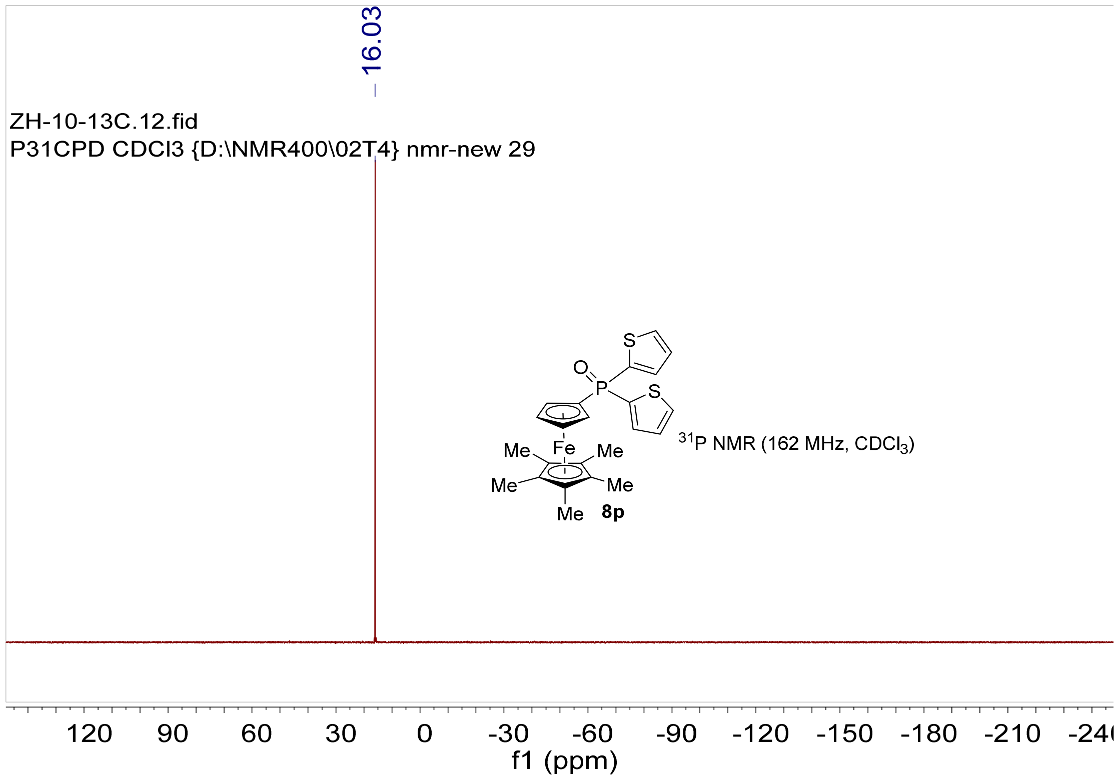

Supplementary Figure 155. ^{31}P NMR spectra of compound 8p

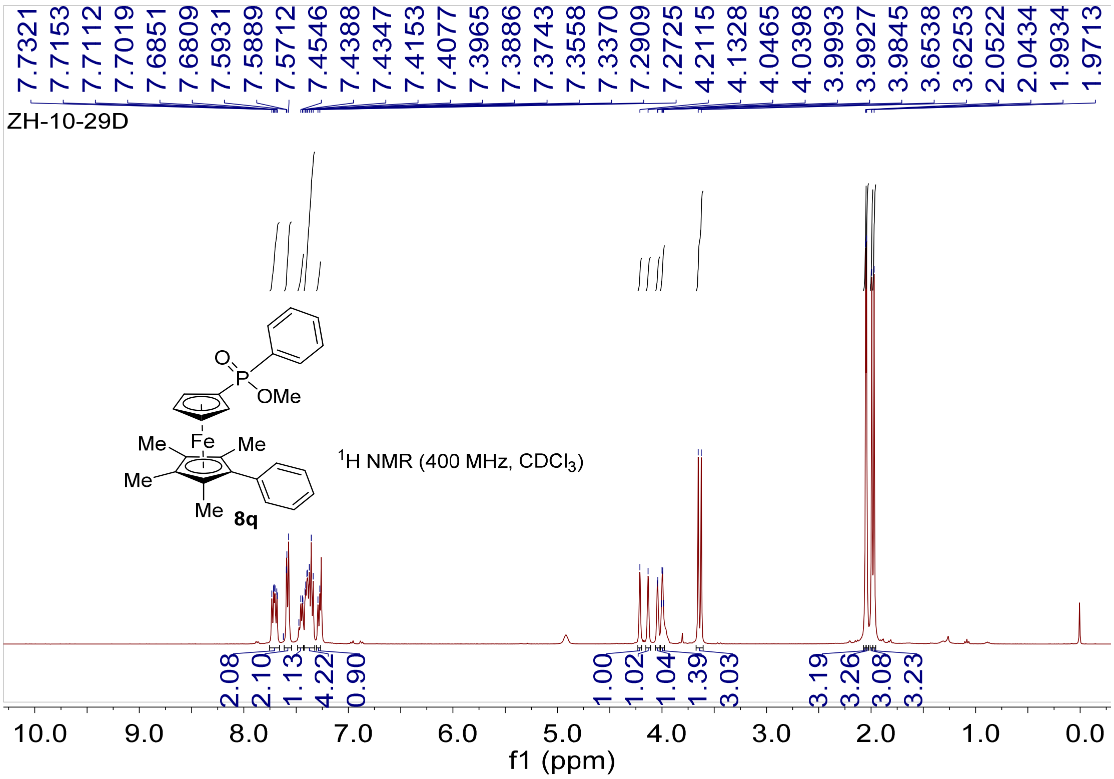

Supplementary Figure 156. ^{1}H NMR spectra of compound 8q

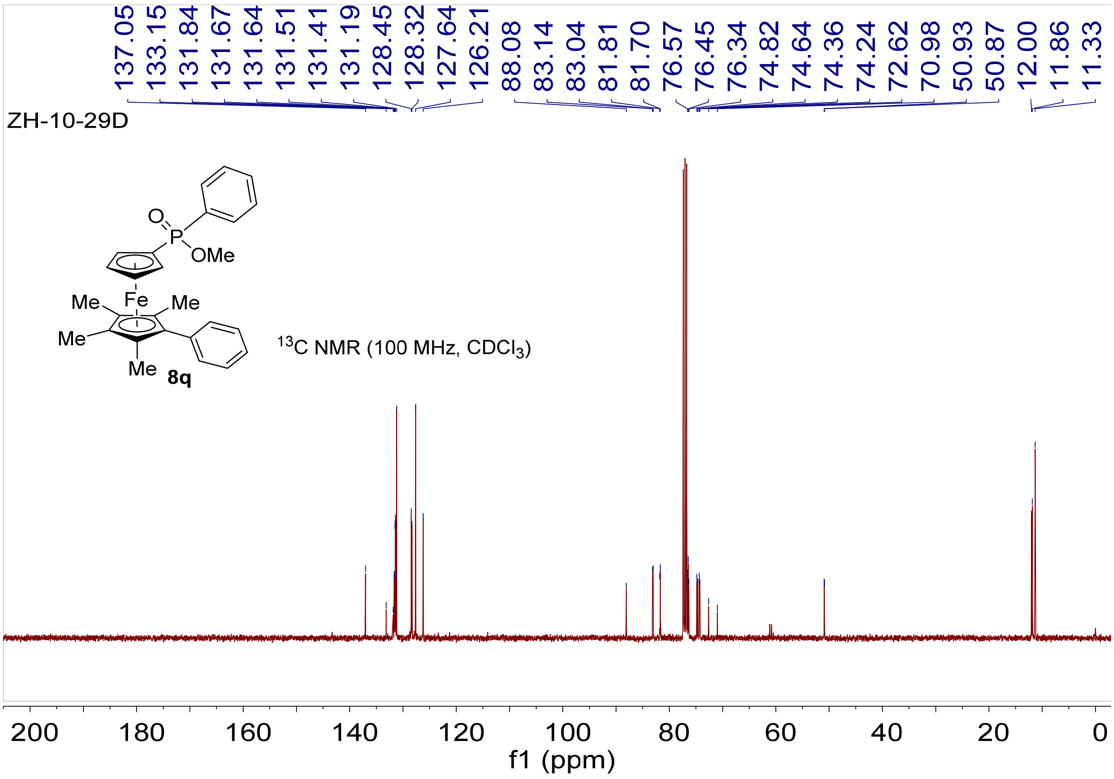

Supplementary Figure 157. ^{13}C NMR spectra of compound 8q

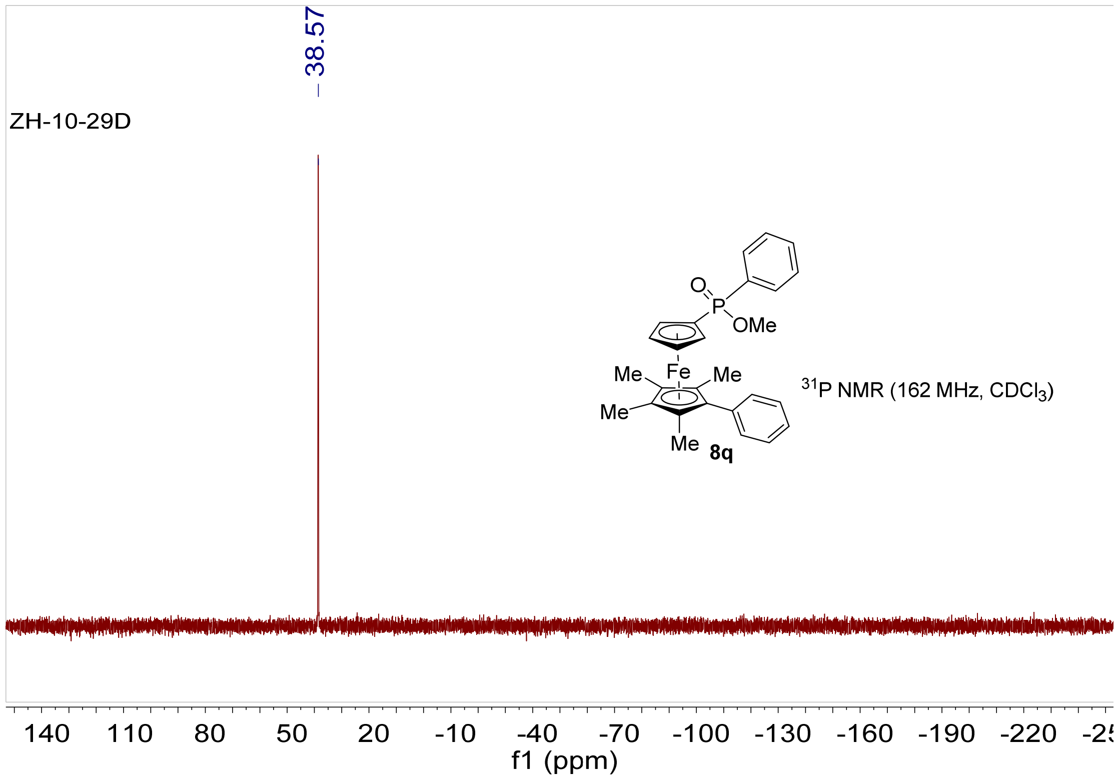

Supplementary Figure 158. ^{31}P NMR spectra of compound 8q

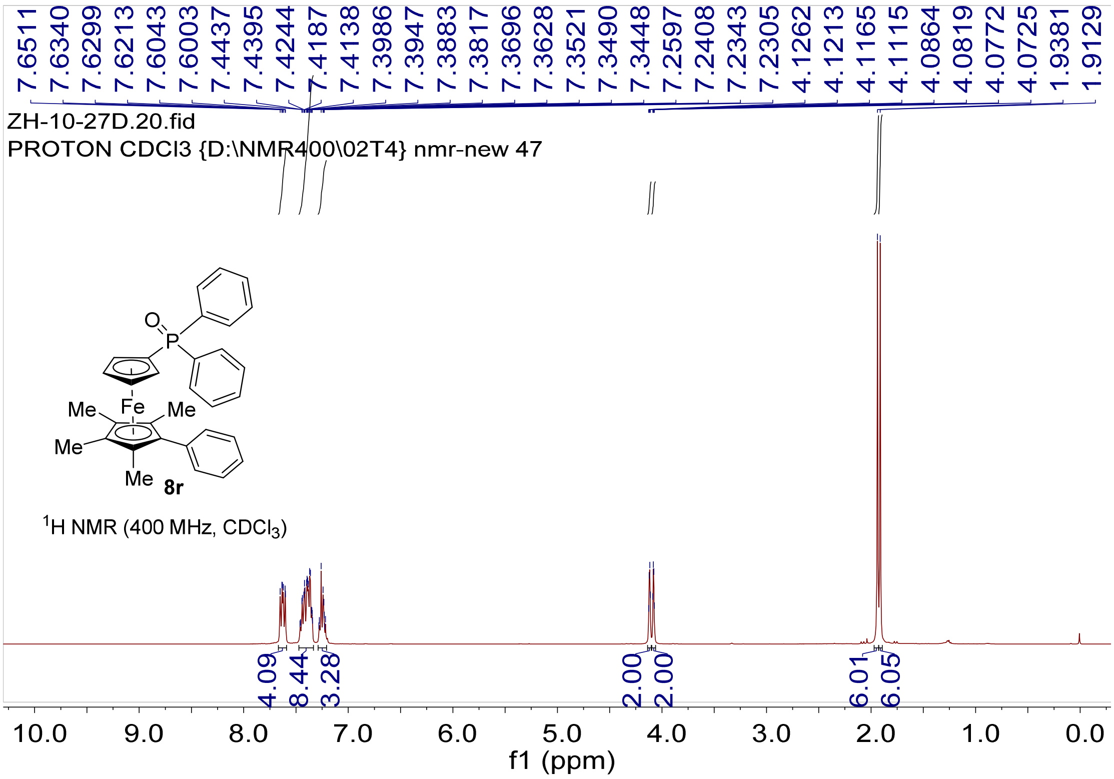

Supplementary Figure 159. ^{1}H NMR spectra of compound 8r

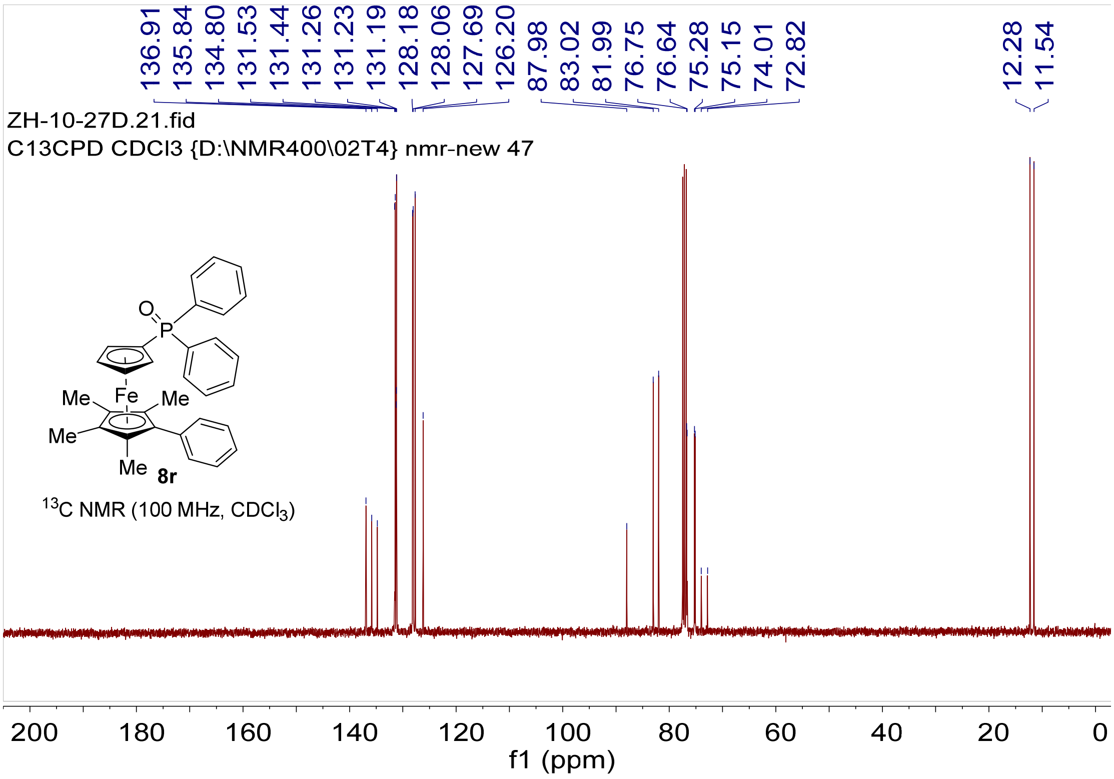

Supplementary Figure 160. ^{13}C NMR spectra of compound 8r

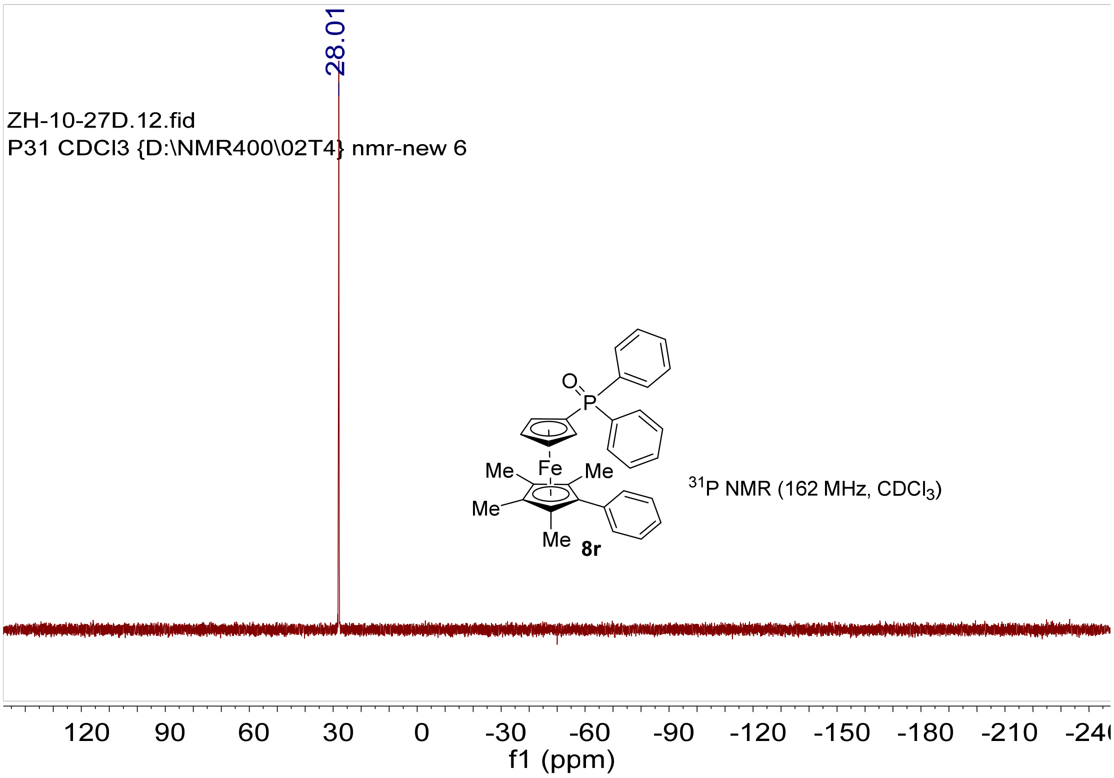

Supplementary Figure 161. ^{31}P NMR spectra of compound 8r

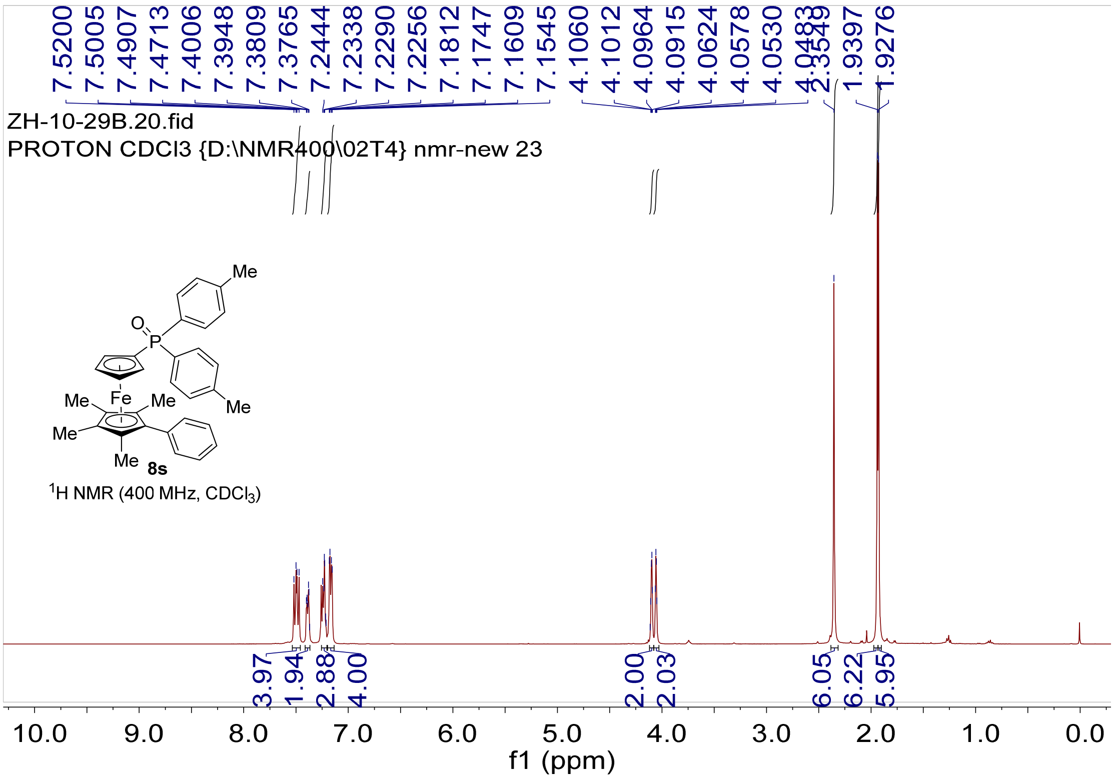

Supplementary Figure 162. ^{1}H NMR spectra of compound 8s

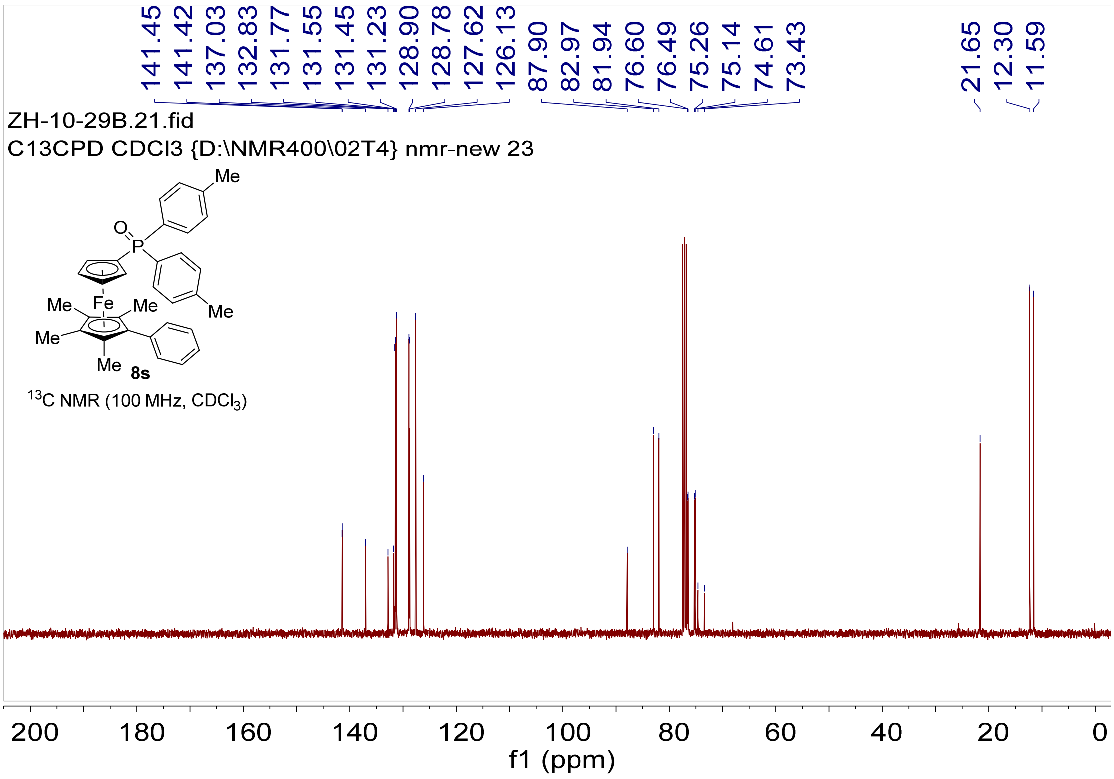

Supplementary Figure 163. ^{13}C NMR spectra of compound 8s

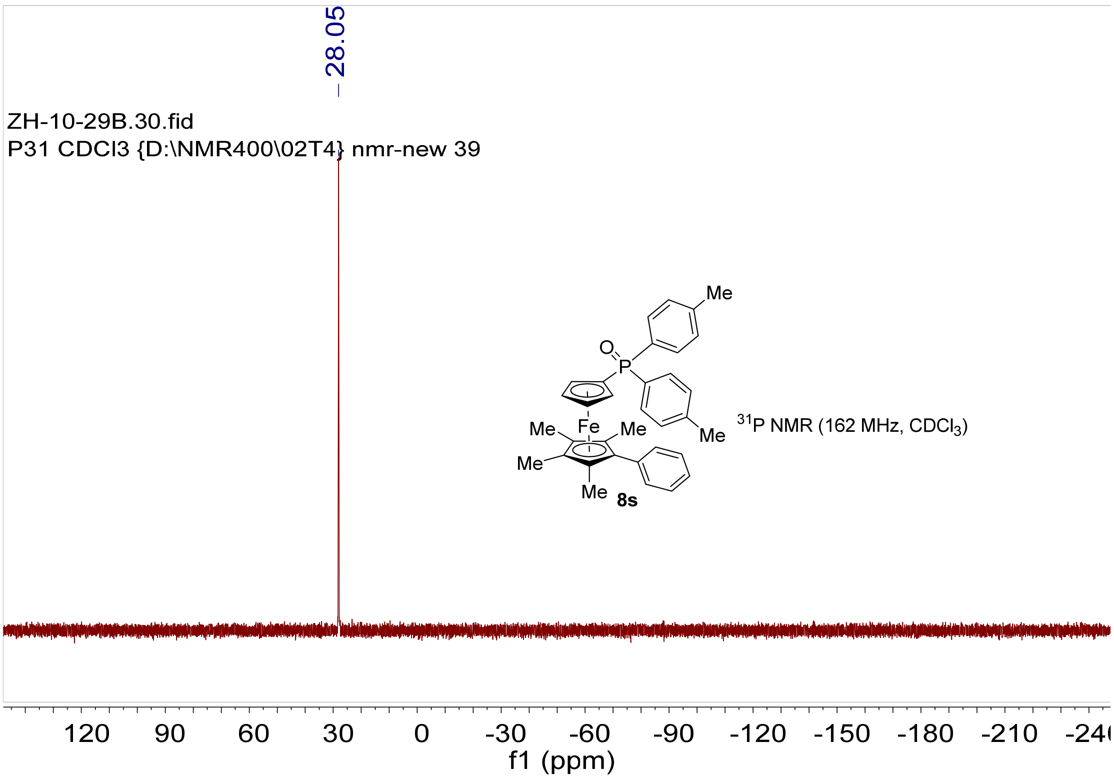

Supplementary Figure 164. ^{31}P NMR spectra of compound 8s

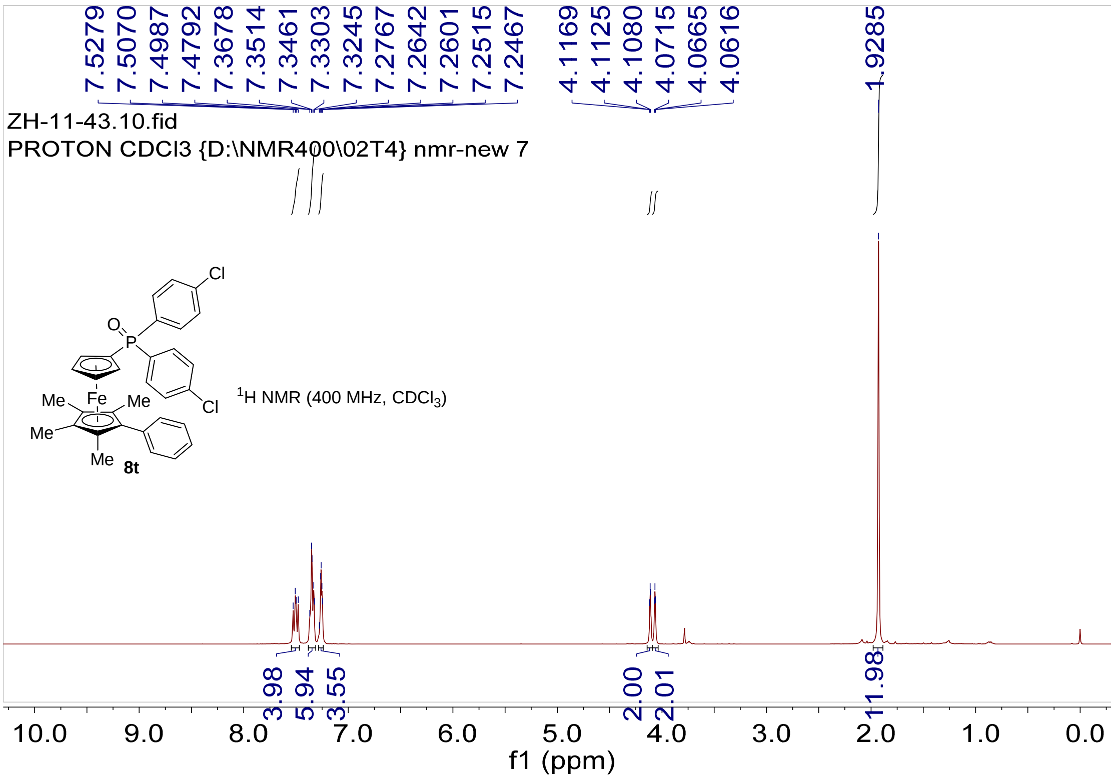

Supplementary Figure 165. ^{1}H NMR spectra of compound 8t

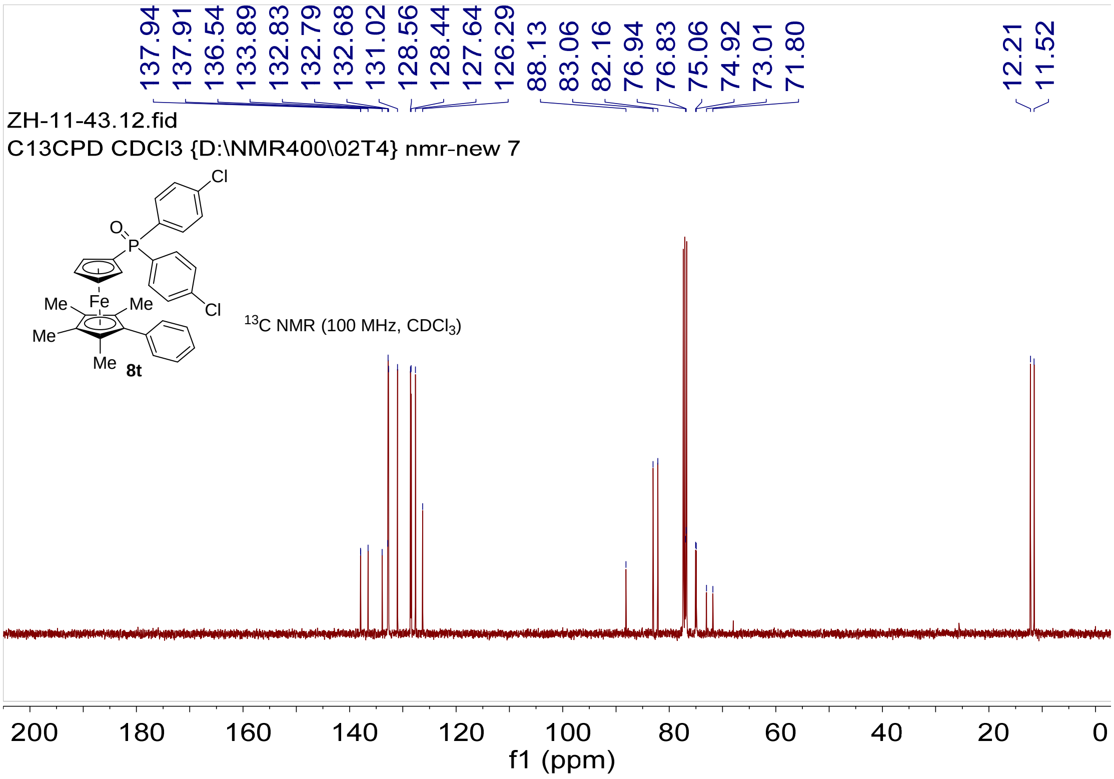

Supplementary Figure 166. ^{13}C NMR spectra of compound 8t

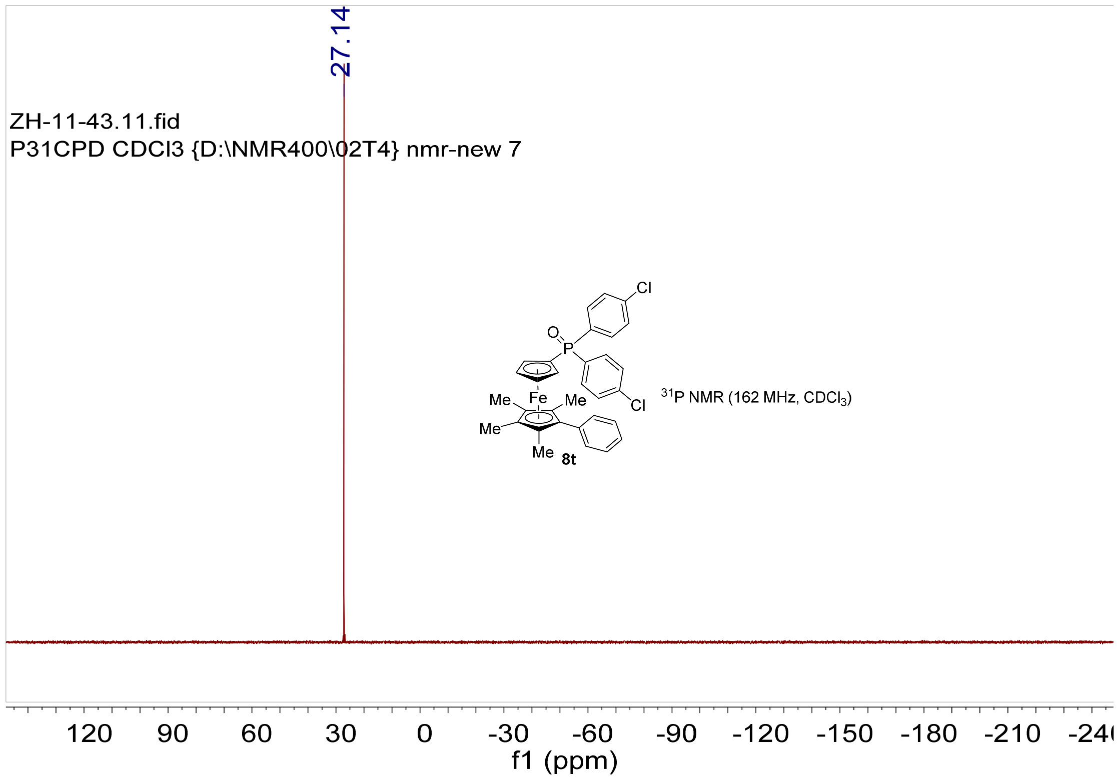

Supplementary Figure 167. ^{31}P NMR spectra of compound 8t

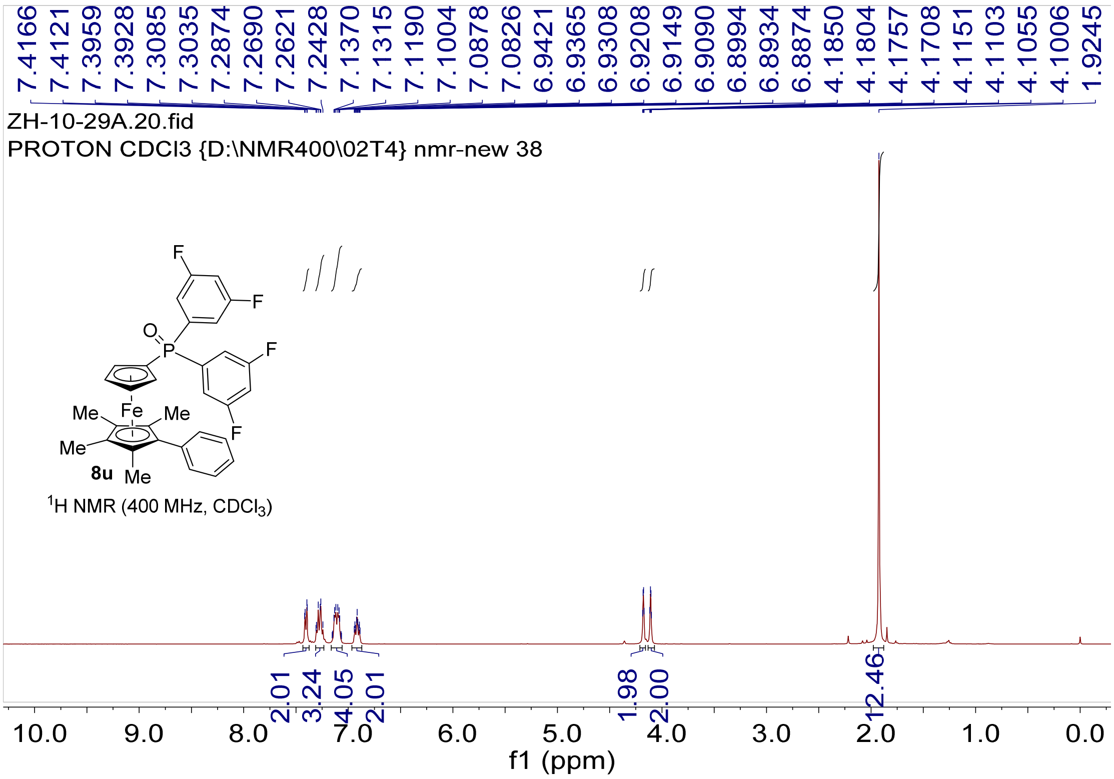

Supplementary Figure 168. ^{1}H NMR spectra of compound 8u

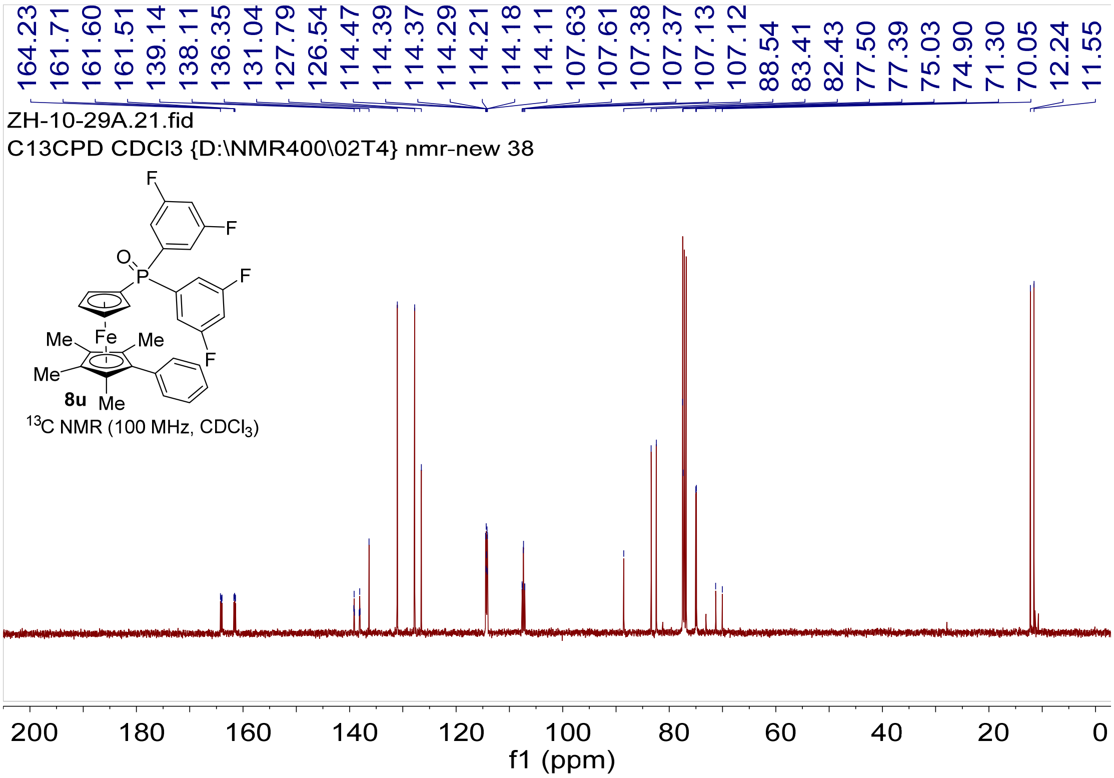

Supplementary Figure 169. ^{13}C NMR spectra of compound 8u

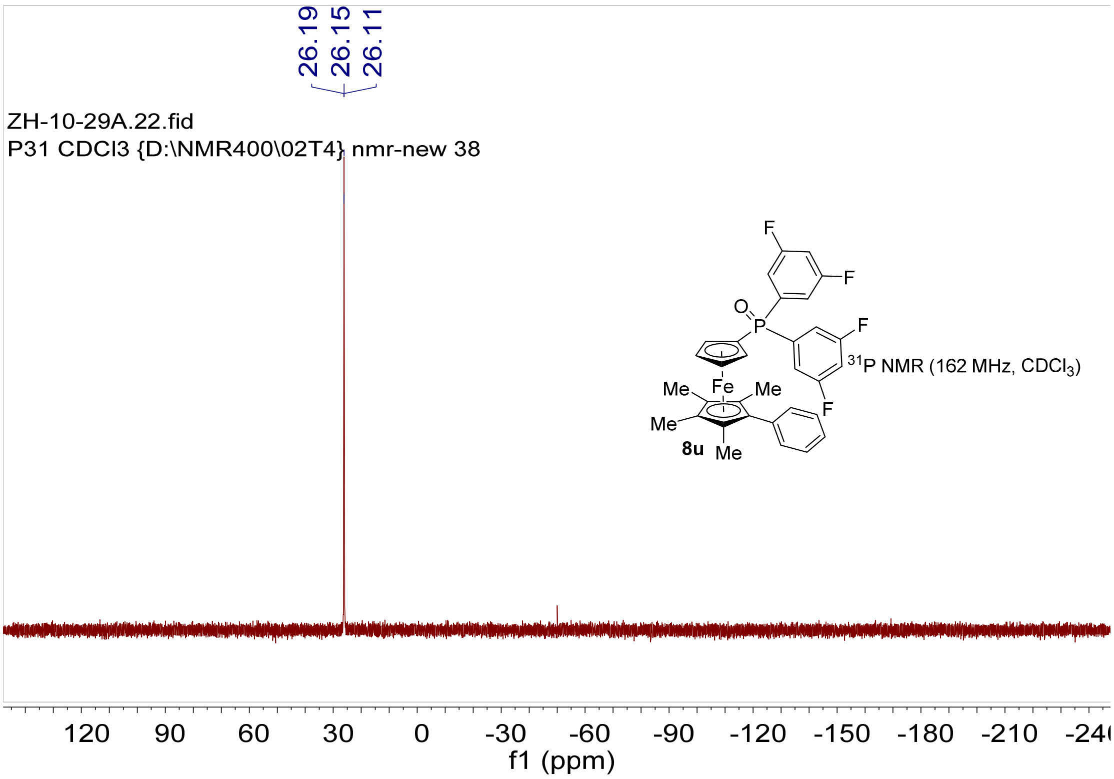

Supplementary Figure 170. ^{31}P NMR spectra of compound 8u

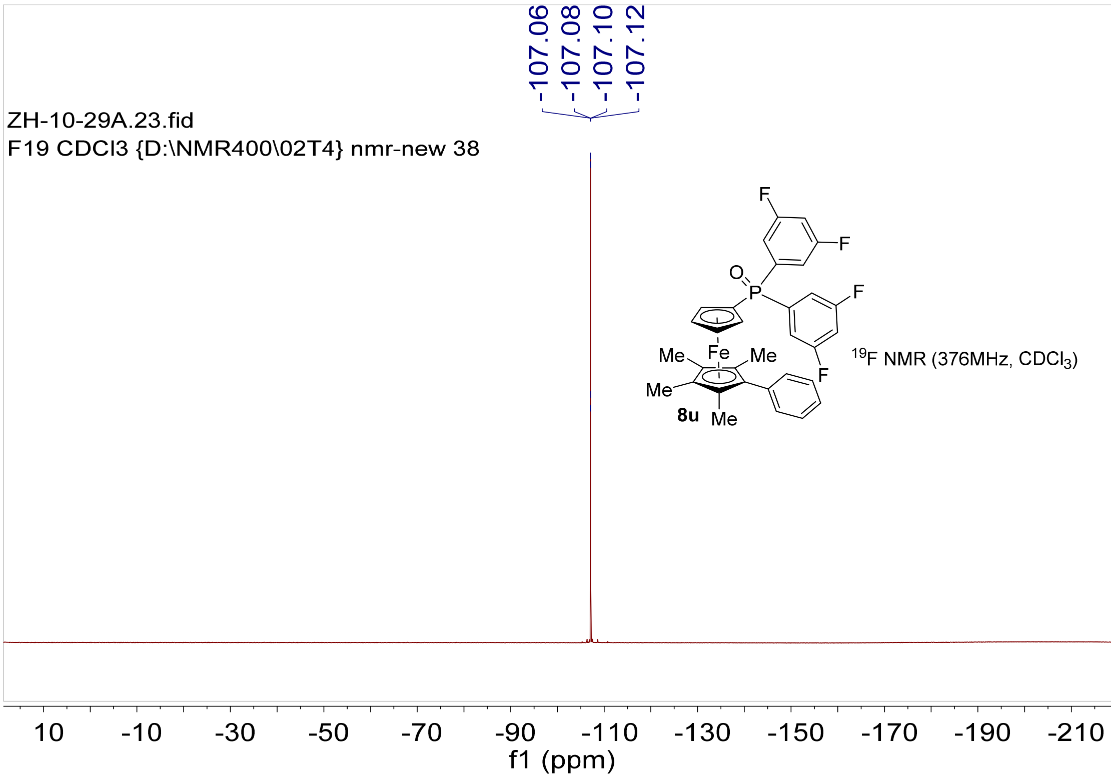

Supplementary Figure 171. ^{19}F NMR spectra of compound 8u

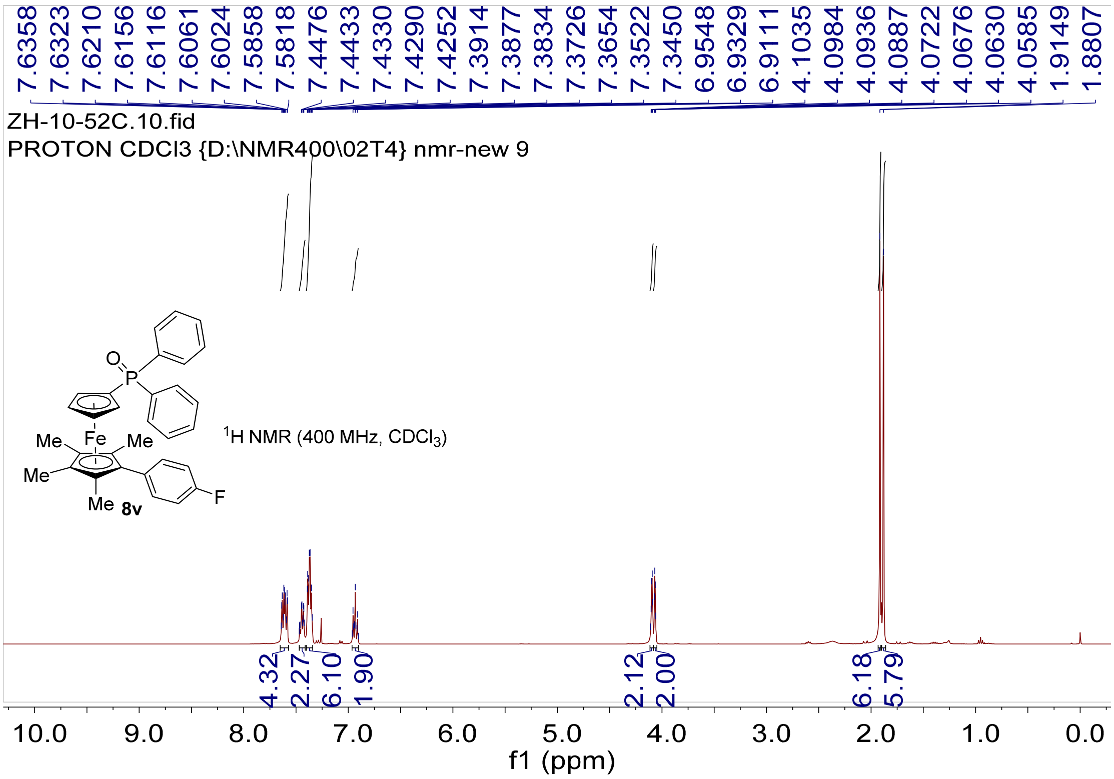

Supplementary Figure 172. ^{1}H NMR spectra of compound 8v

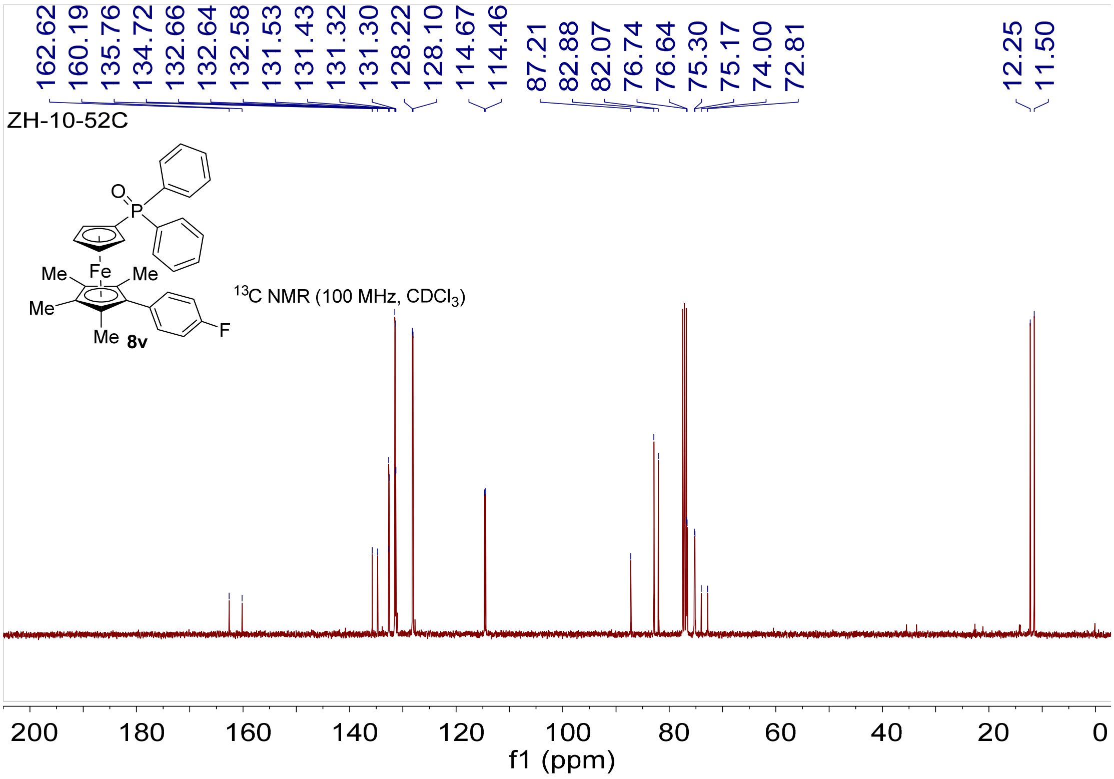

Supplementary Figure 173. ^{13}C NMR spectra of compound 8v

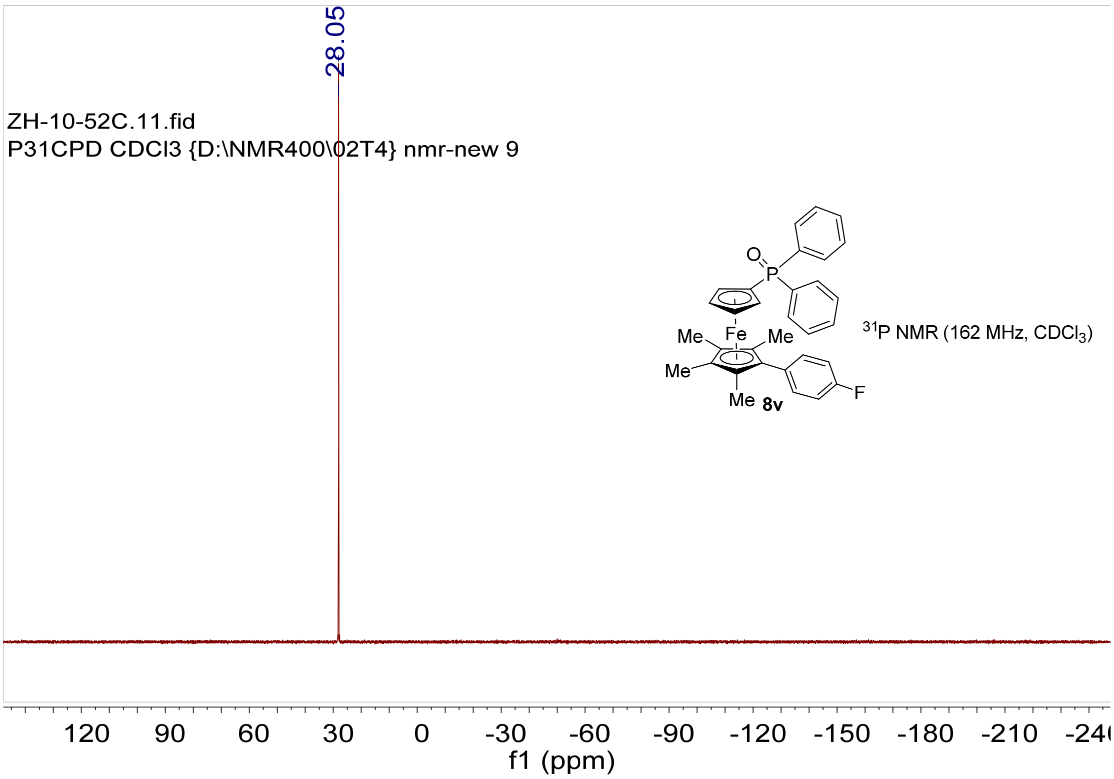

Supplementary Figure 174. ^{31}**P NMR spectra of compound 8v**

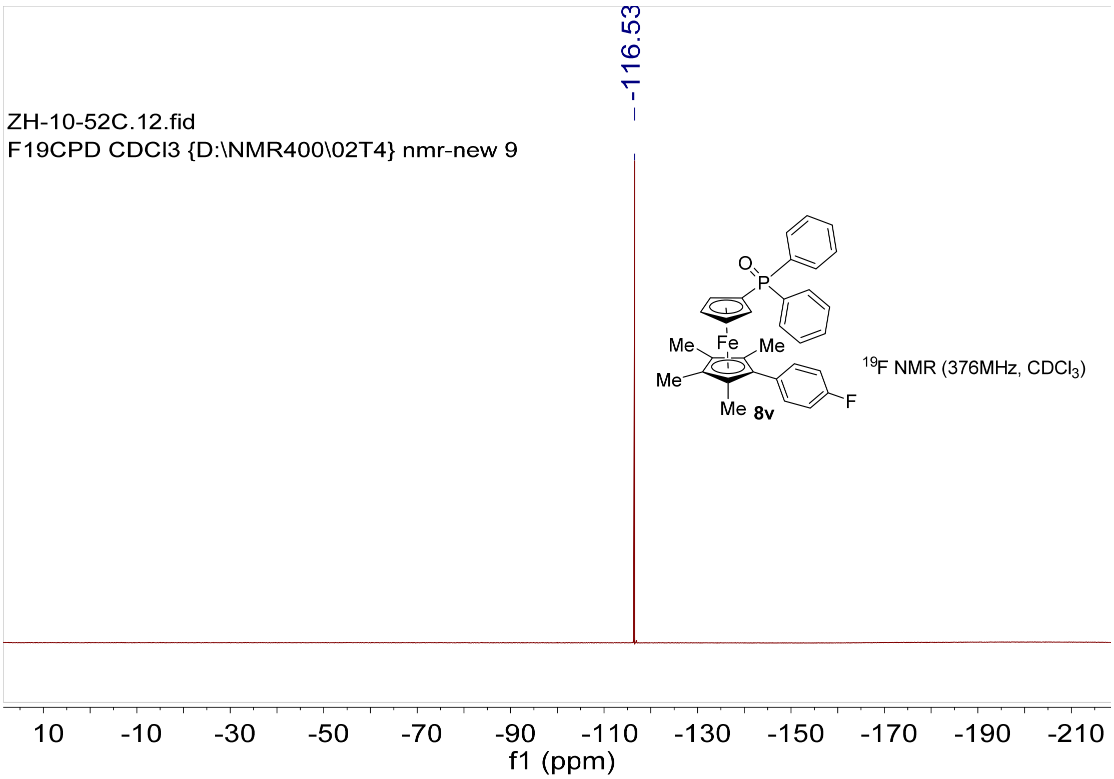

Supplementary Figure 175. ^{19}**F NMR spectra of compound 8v**

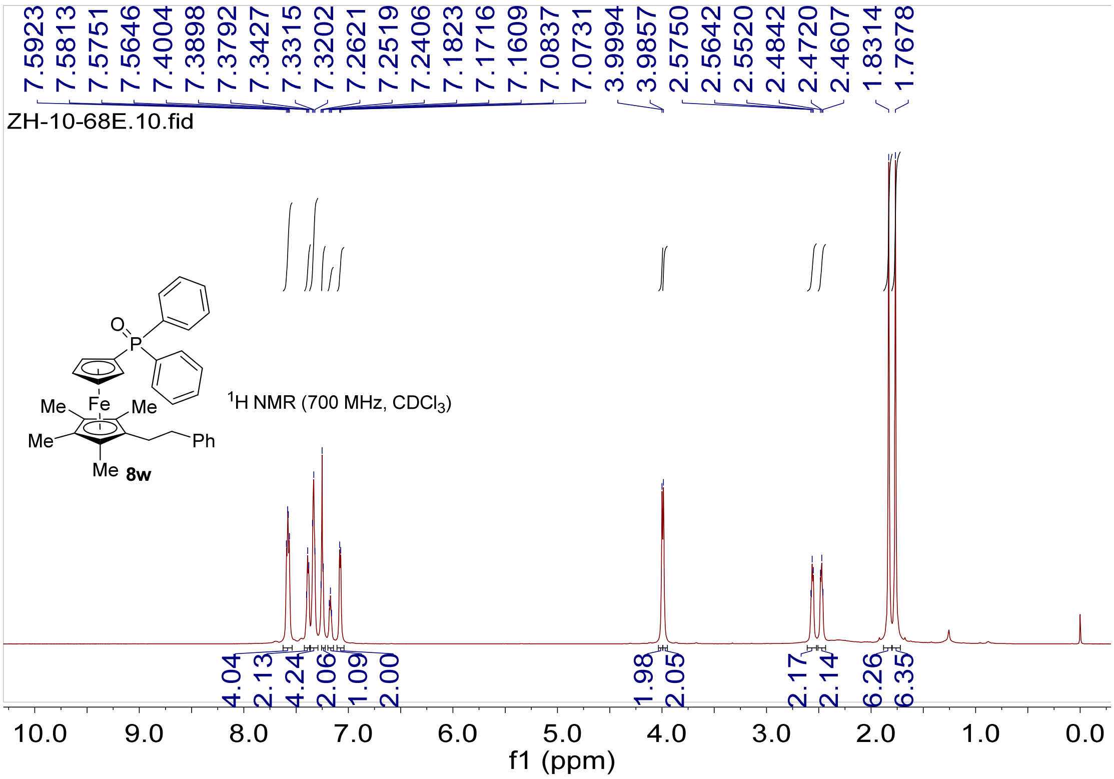

Supplementary Figure 176. ^{1}H NMR spectra of compound 8w

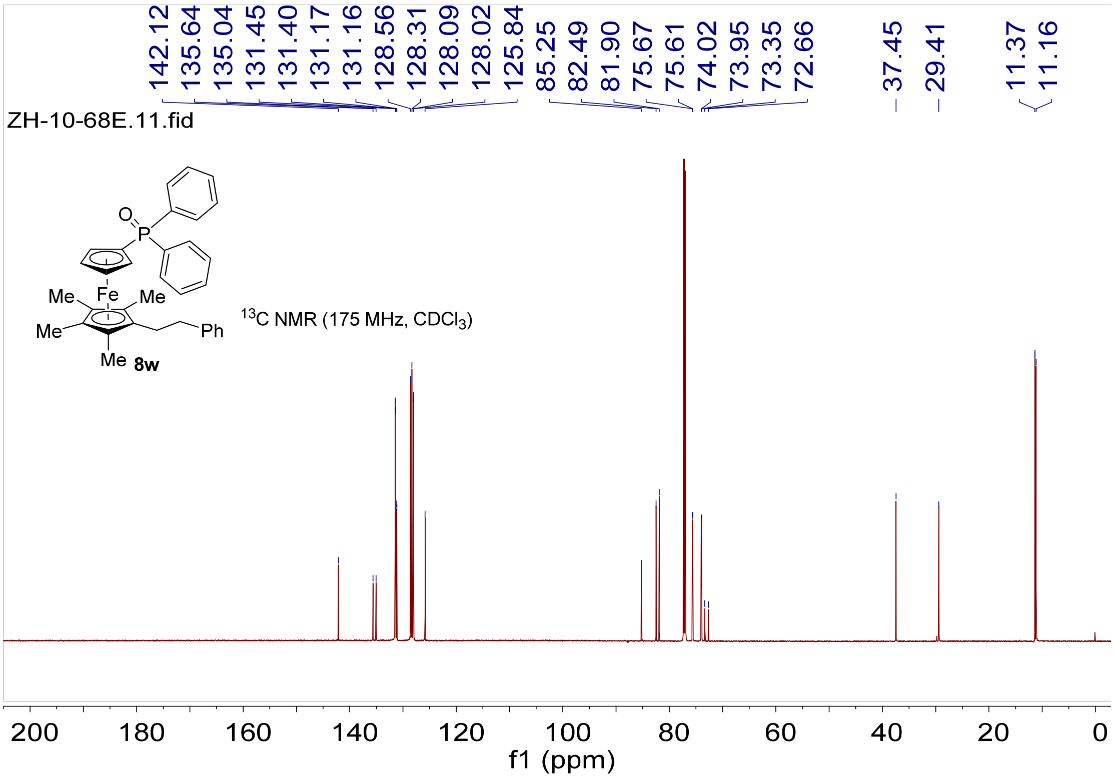

Supplementary Figure 177. ^{13}C NMR spectra of compound 8w

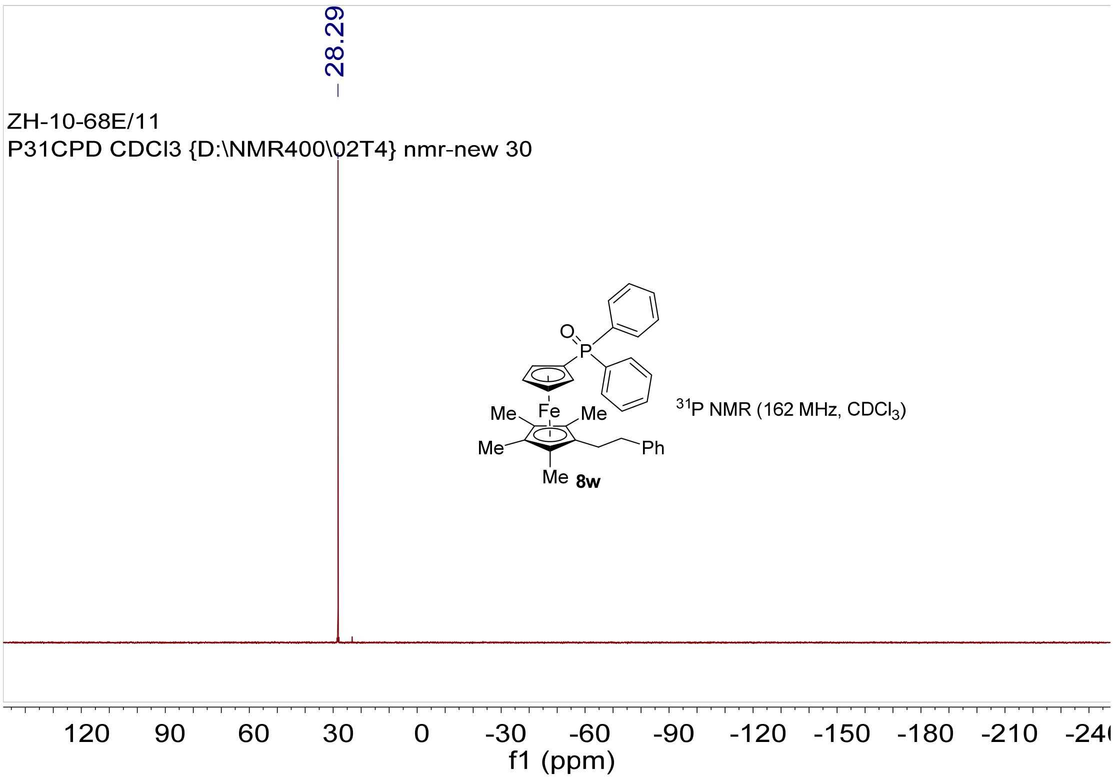

Supplementary Figure 178. ^{31}P NMR spectra of compound 8w

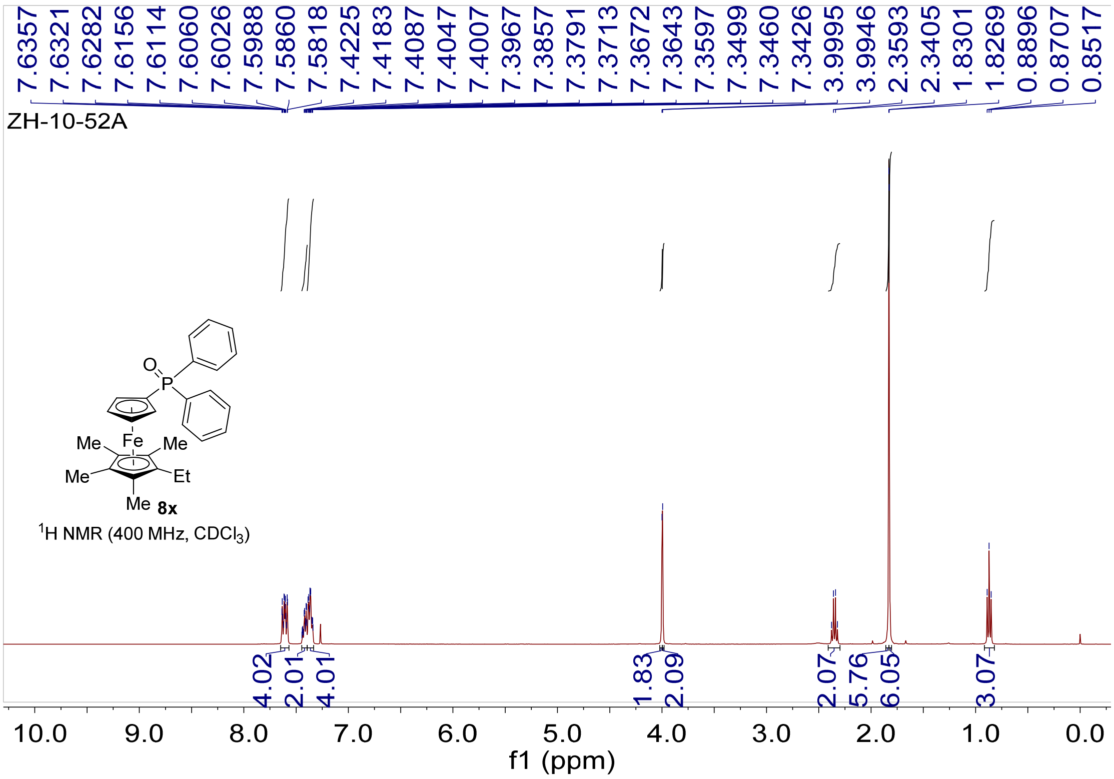

Supplementary Figure 179. ^{1}**H NMR spectra of compound 8x**

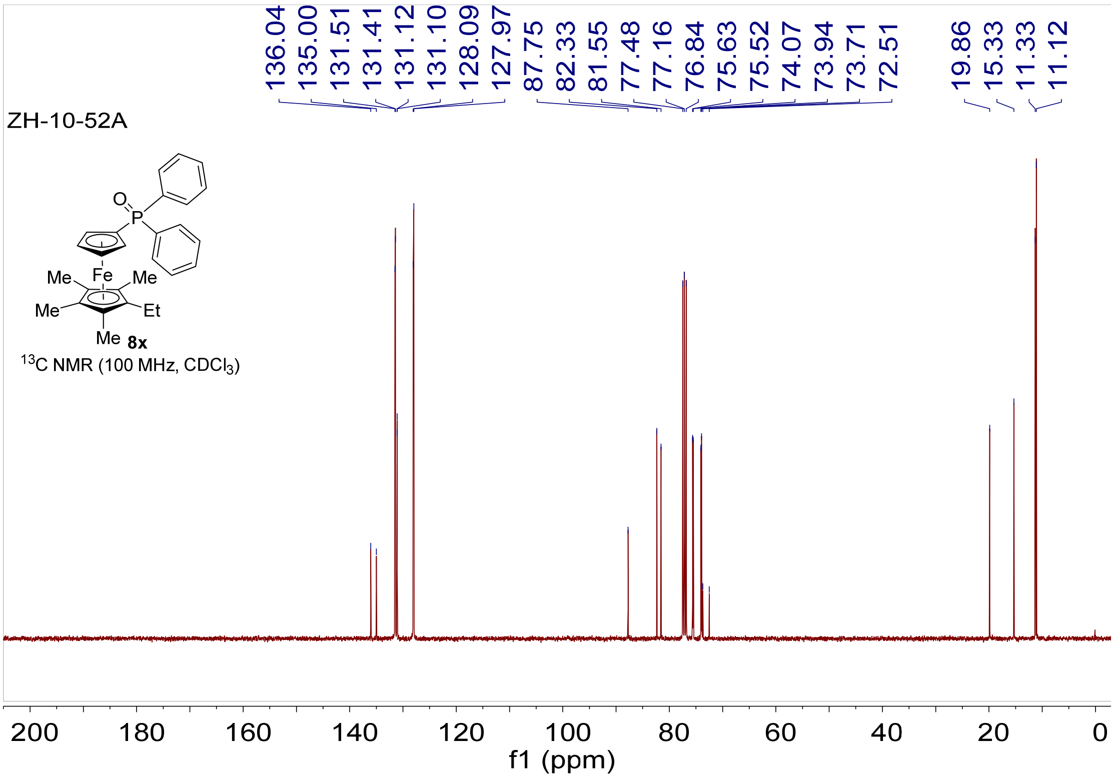

Supplementary Figure 180. ^{13}**C NMR spectra of compound 8x**

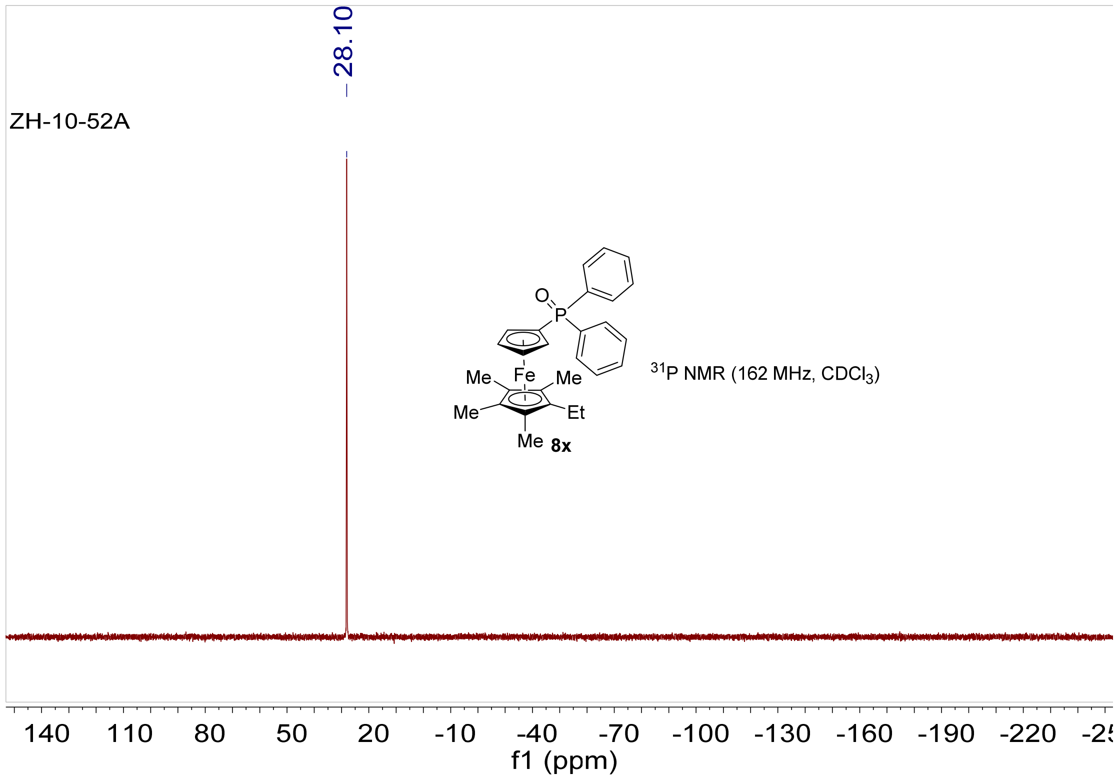

Supplementary Figure 181. ^{31}P NMR spectra of compound 8x

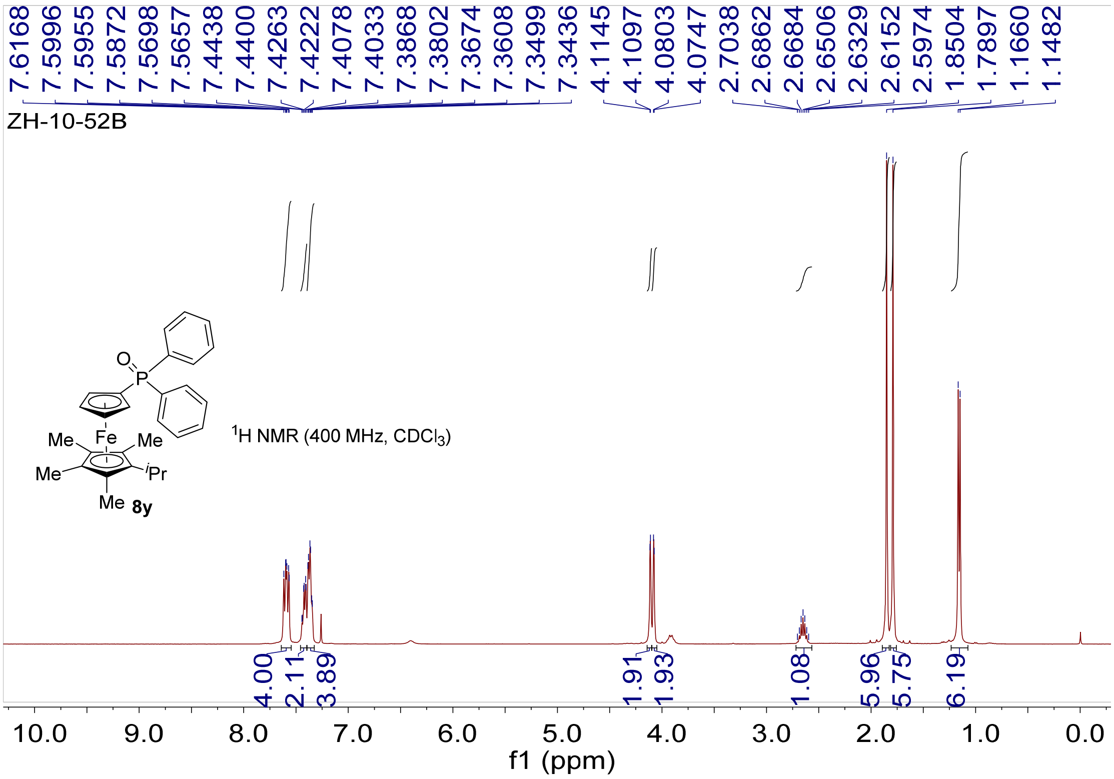

Supplementary Figure 182. ^{1}H NMR spectra of compound 8y

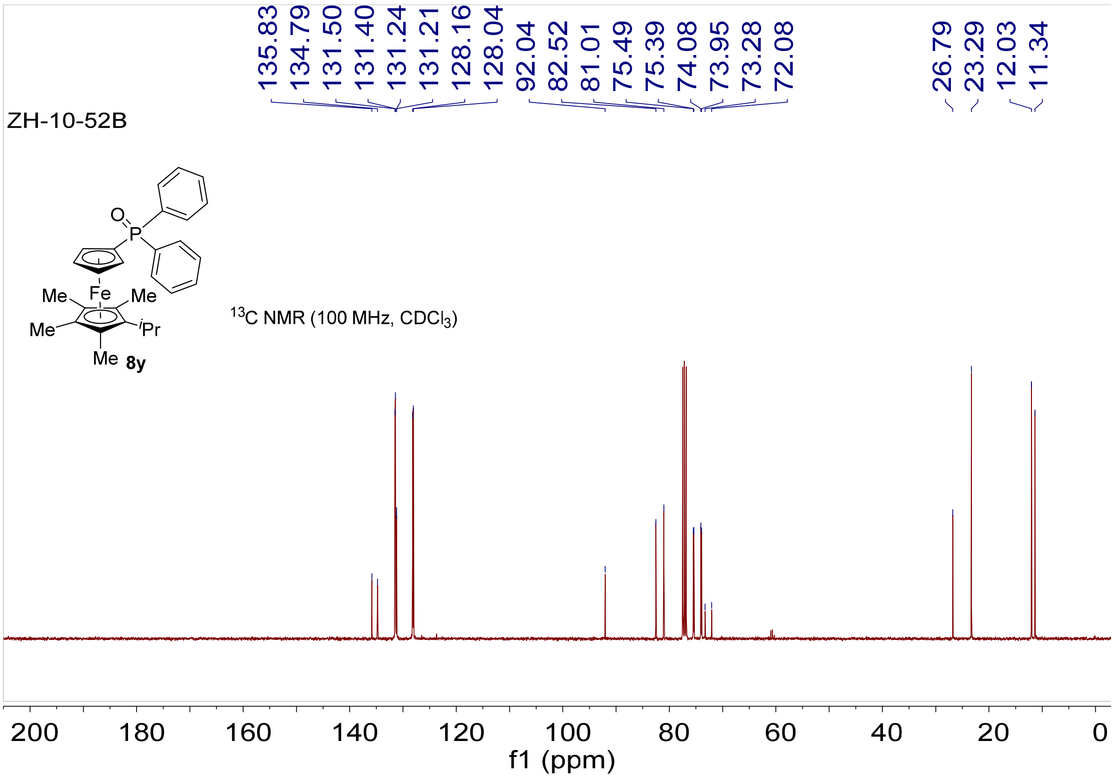

Supplementary Figure 183. ^{13}C NMR spectra of compound 8y

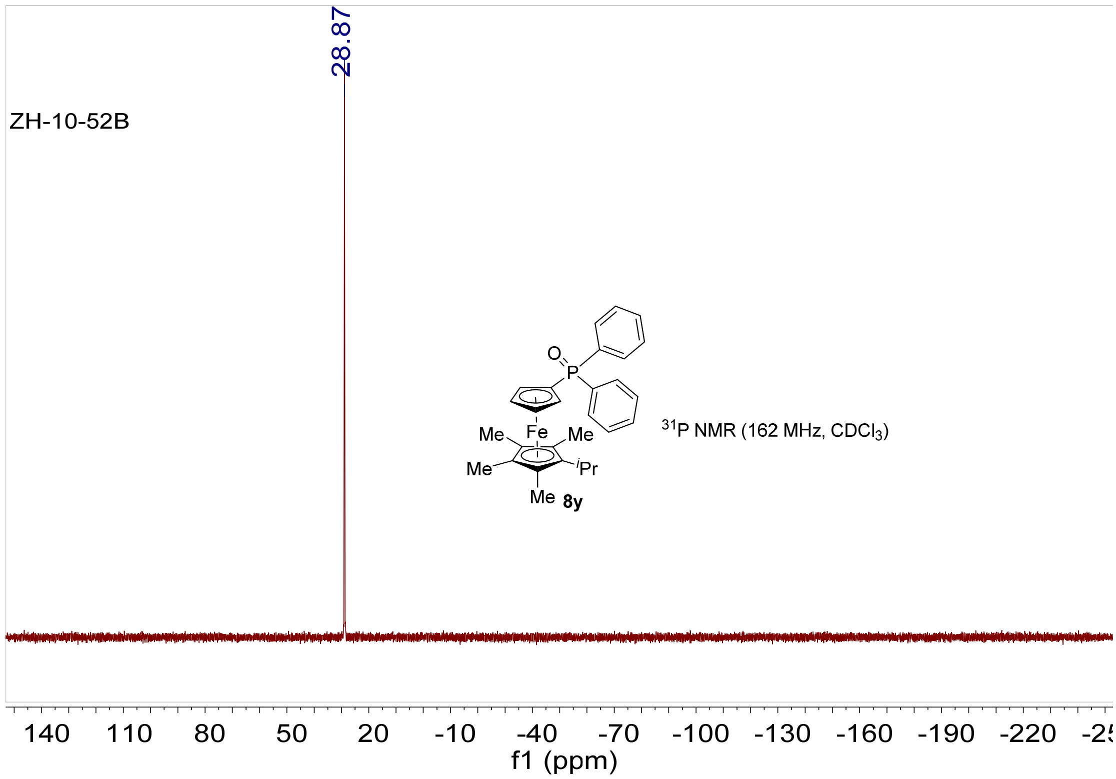

Supplementary Figure 184. ^{31}P NMR spectra of compound 8y

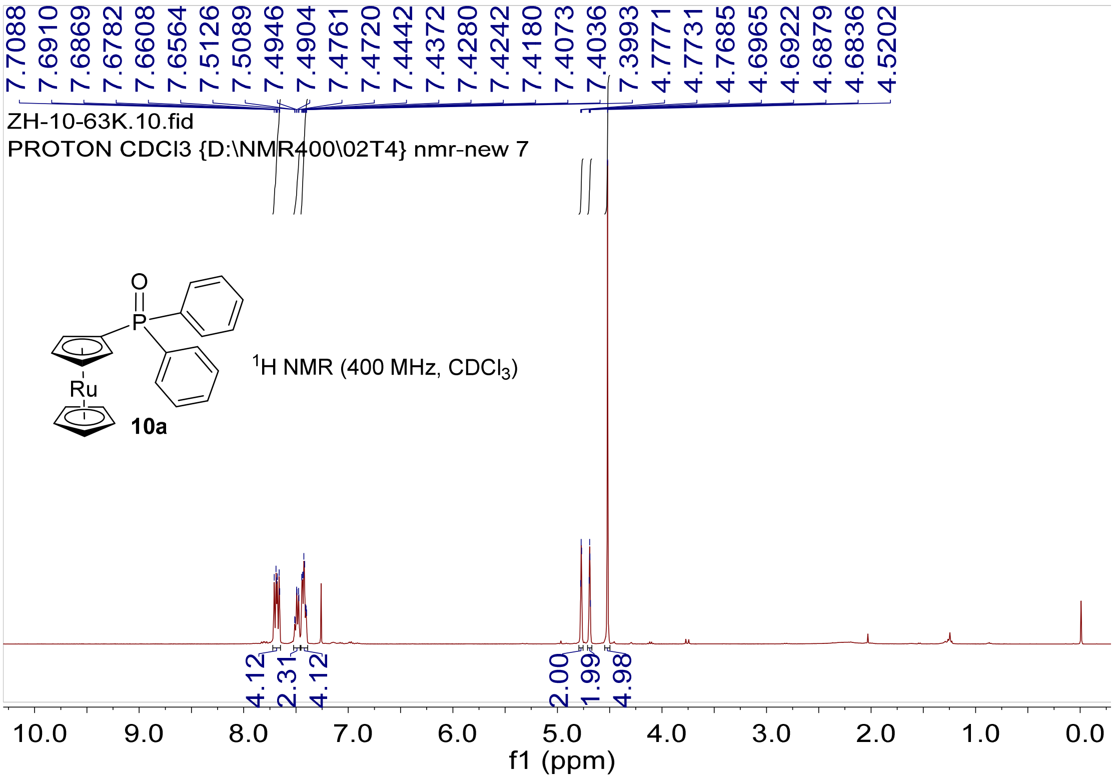

Supplementary Figure 185. ^{1}H NMR spectra of compound 10a

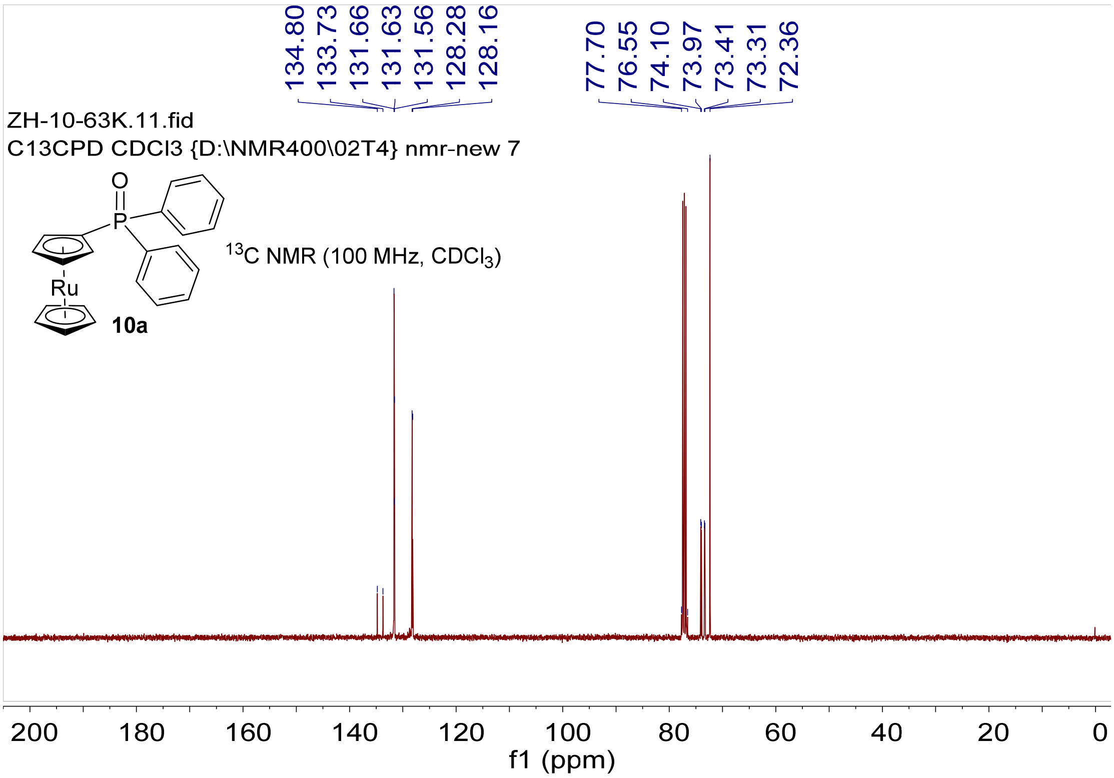

Supplementary Figure 186. ^{13}C NMR spectra of compound 10a

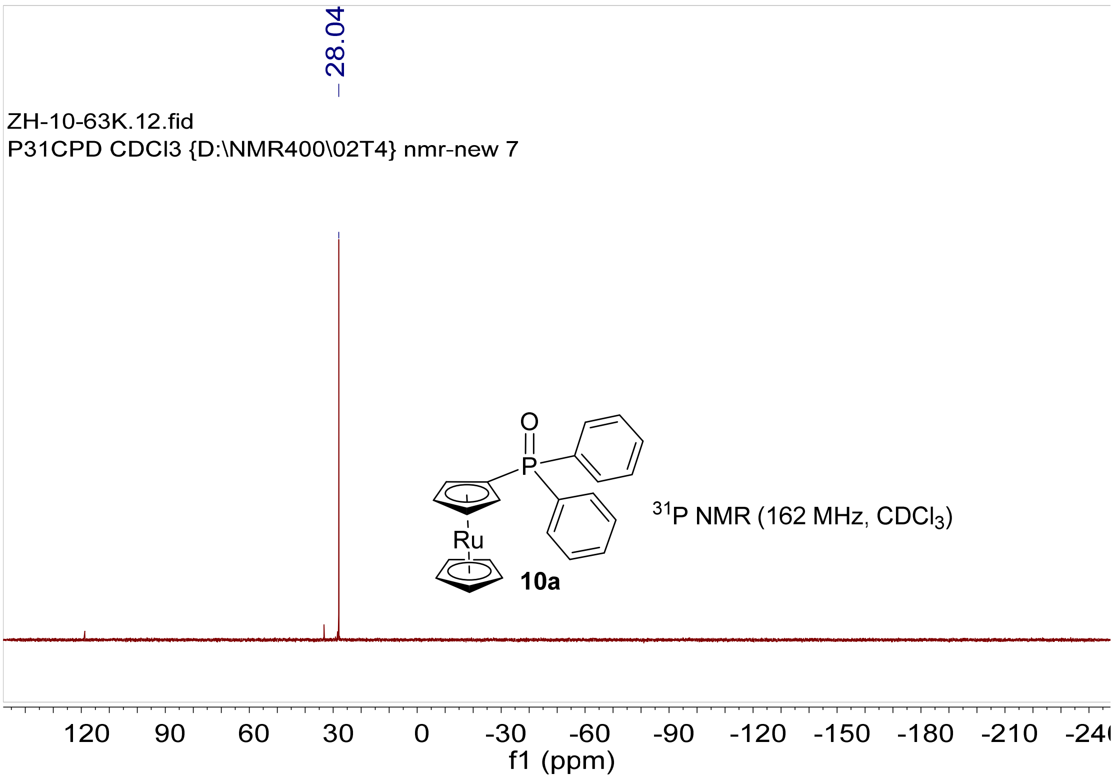

Supplementary Figure 187. ^{31}P NMR spectra of compound 10a

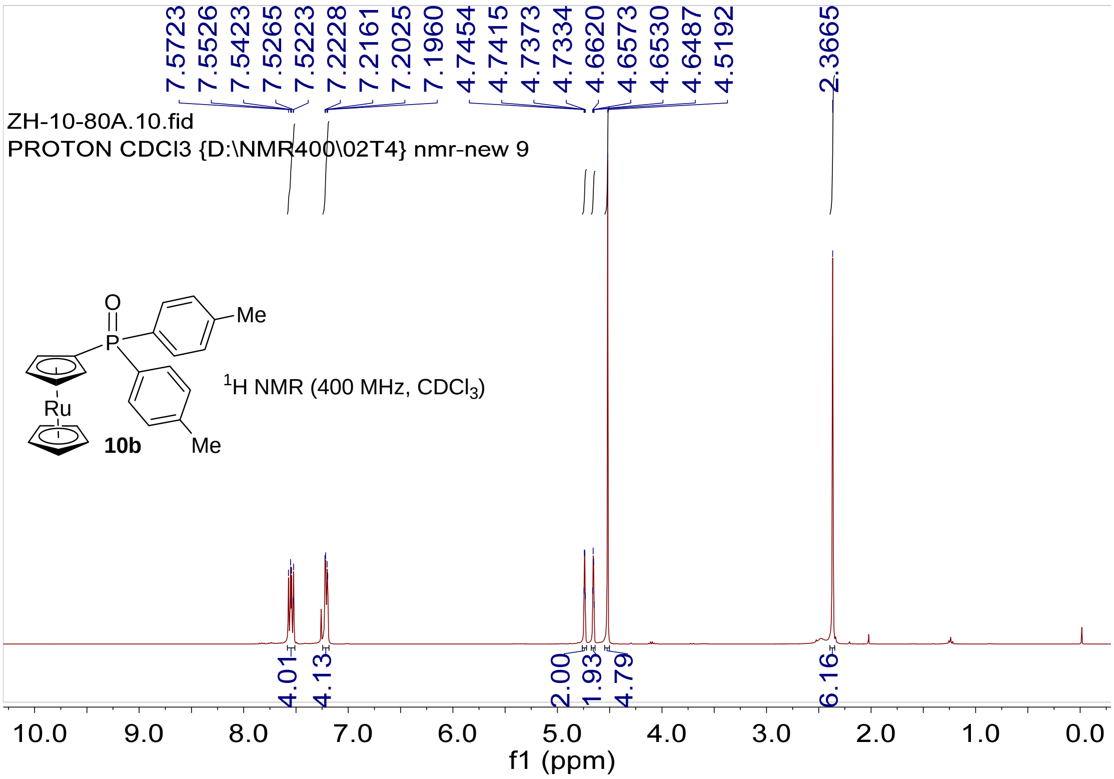

Supplementary Figure 188. ^{1}H NMR spectra of compound 10b

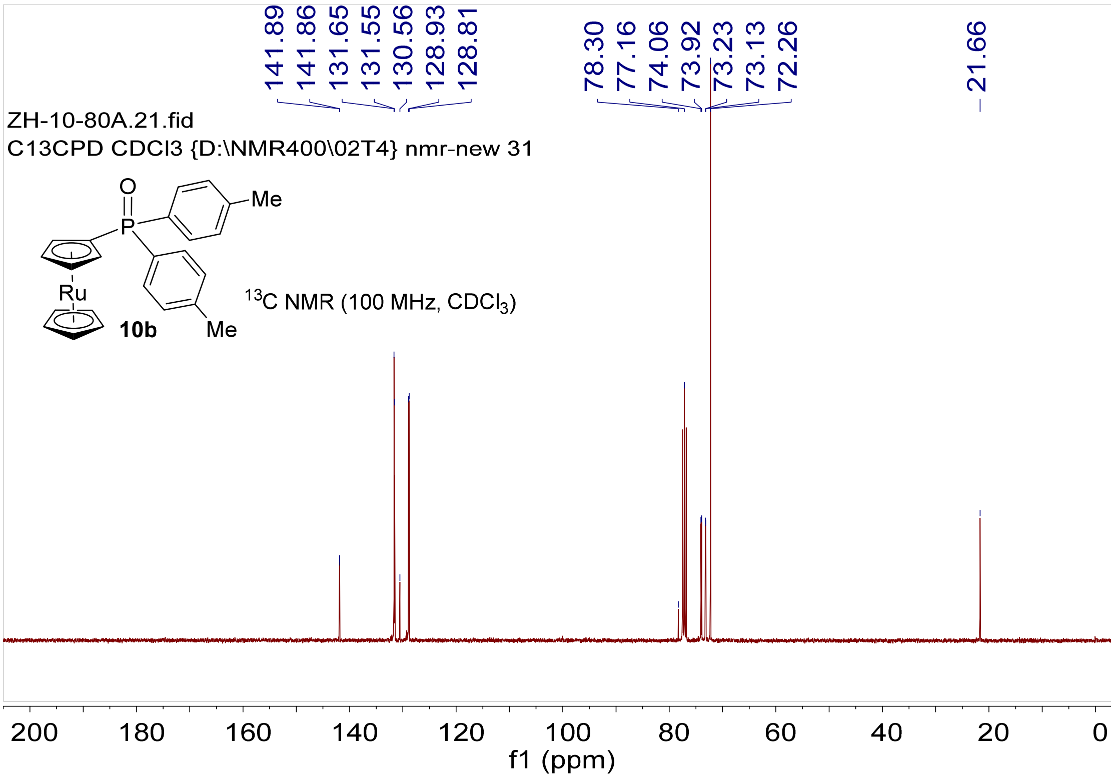

Supplementary Figure 189. ^{13}C NMR spectra of compound 10b

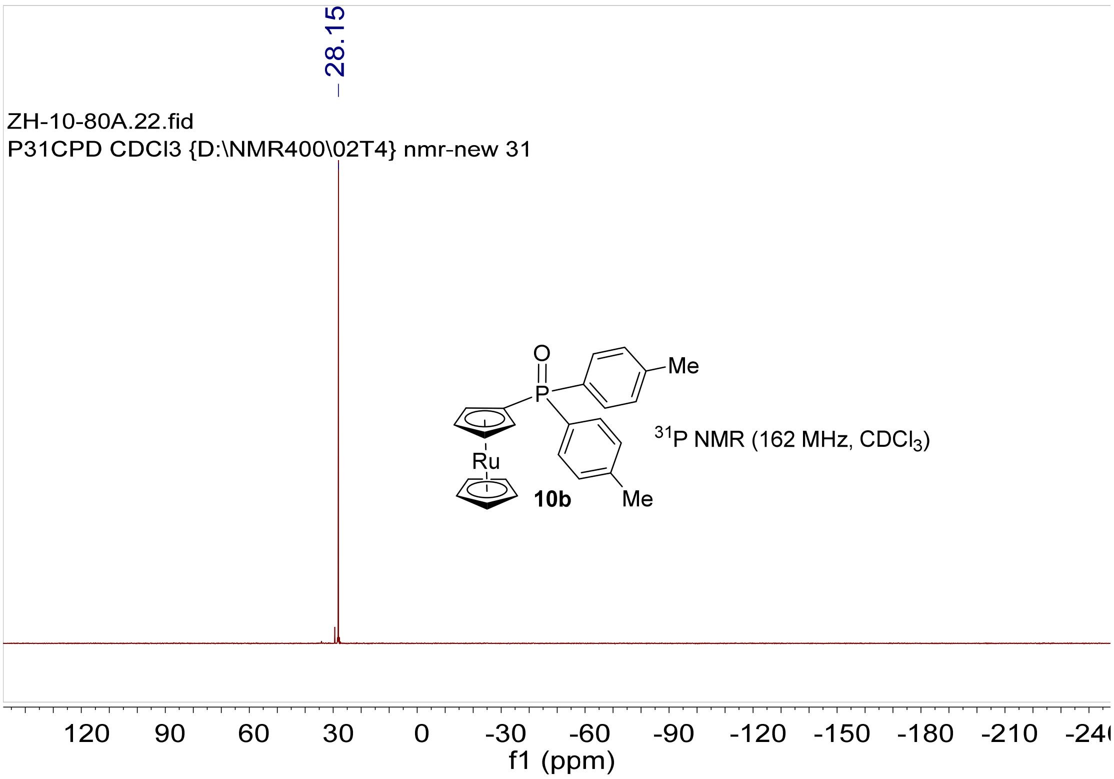

Supplementary Figure 190. ^{31}P NMR spectra of compound 10b

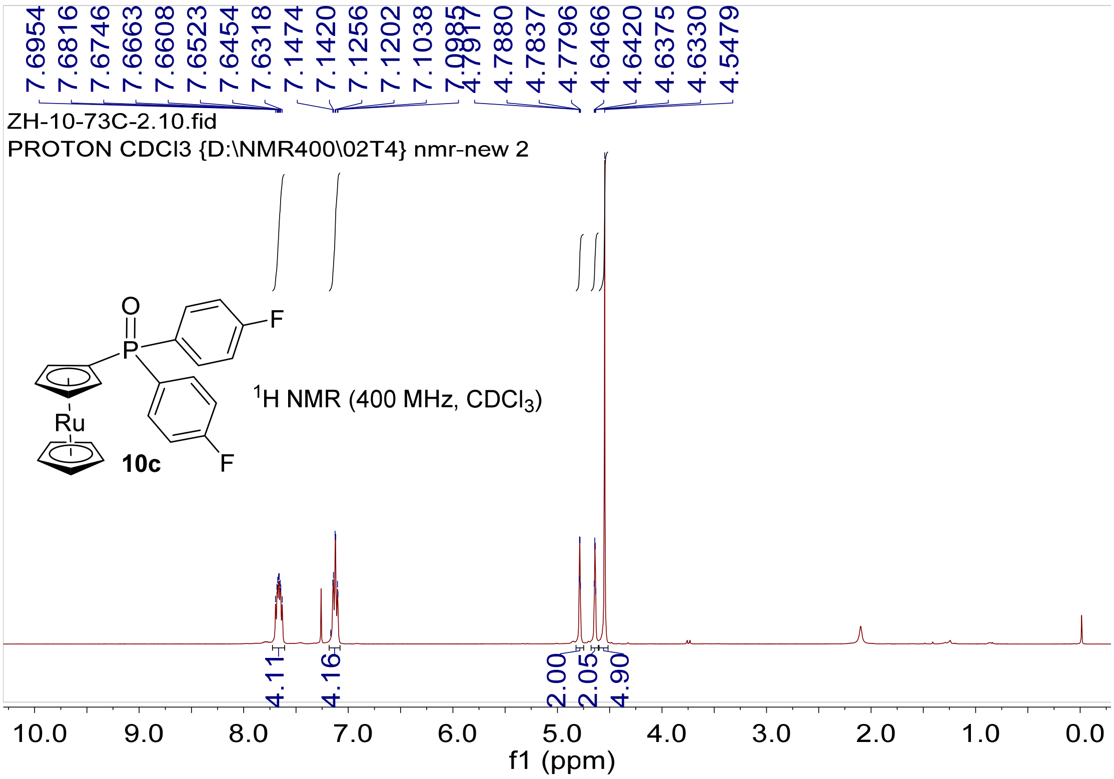

Supplementary Figure 191. ^{1}H NMR spectra of compound 10c

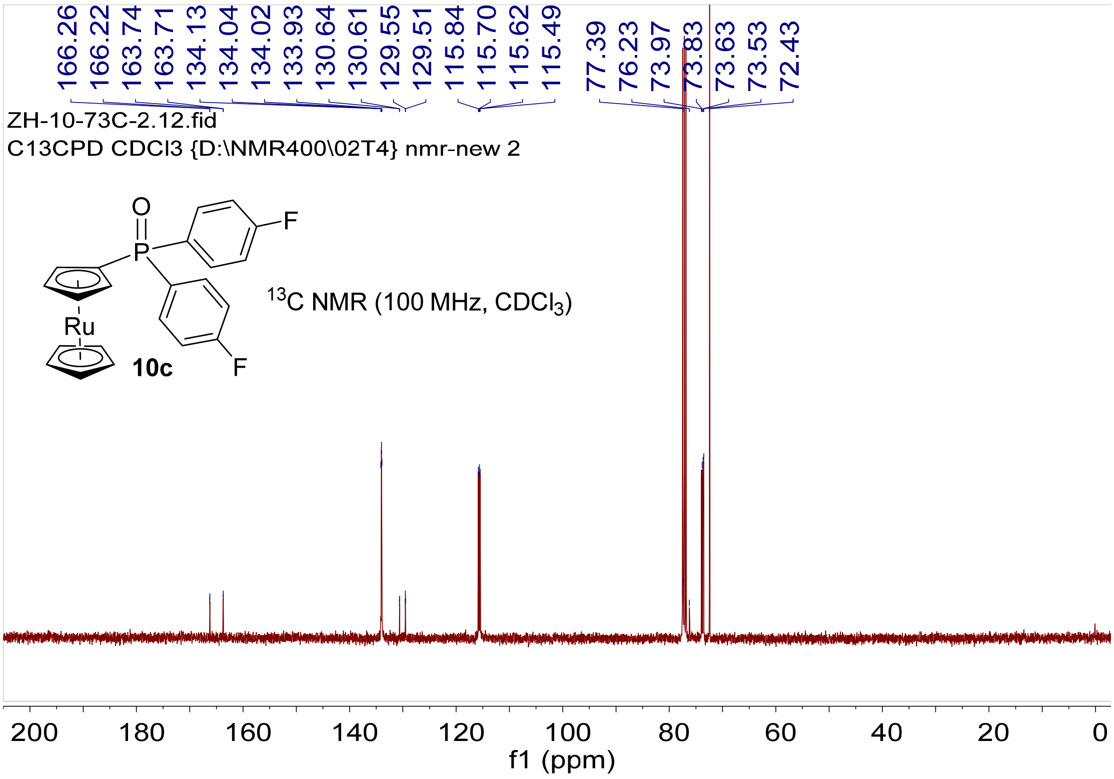

Supplementary Figure 192. ^{13}C NMR spectra of compound 10c

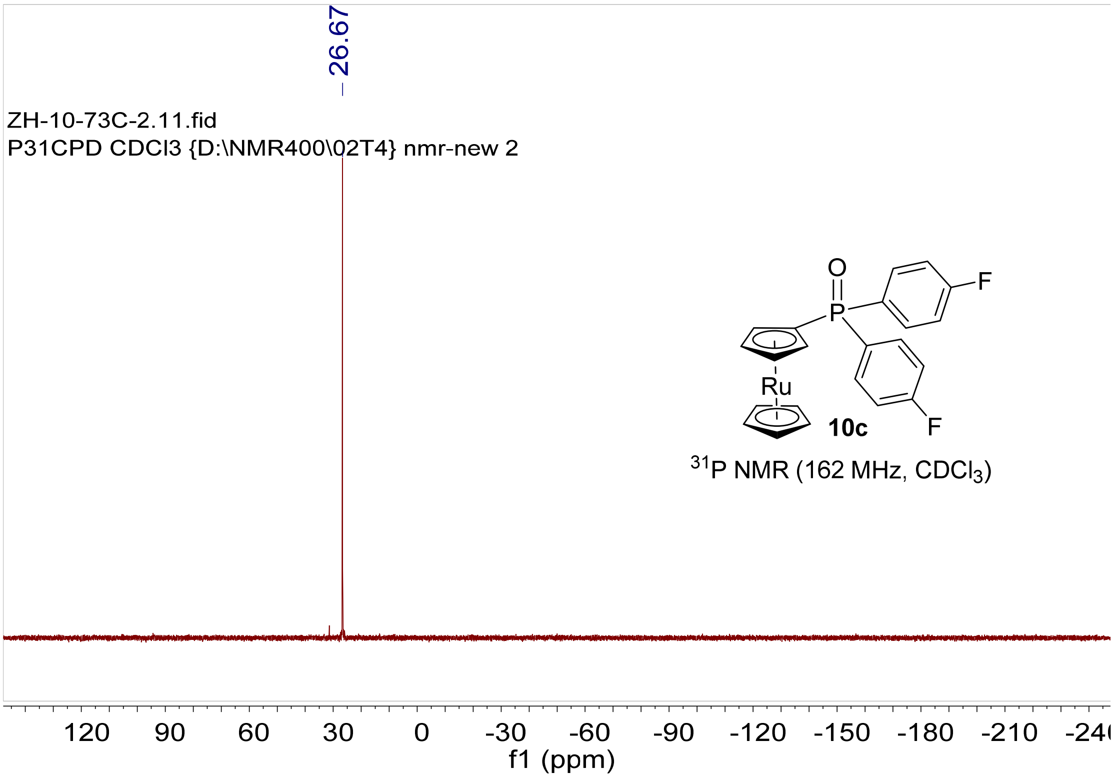

Supplementary Figure 193. ^{31}P NMR spectra of compound 10c

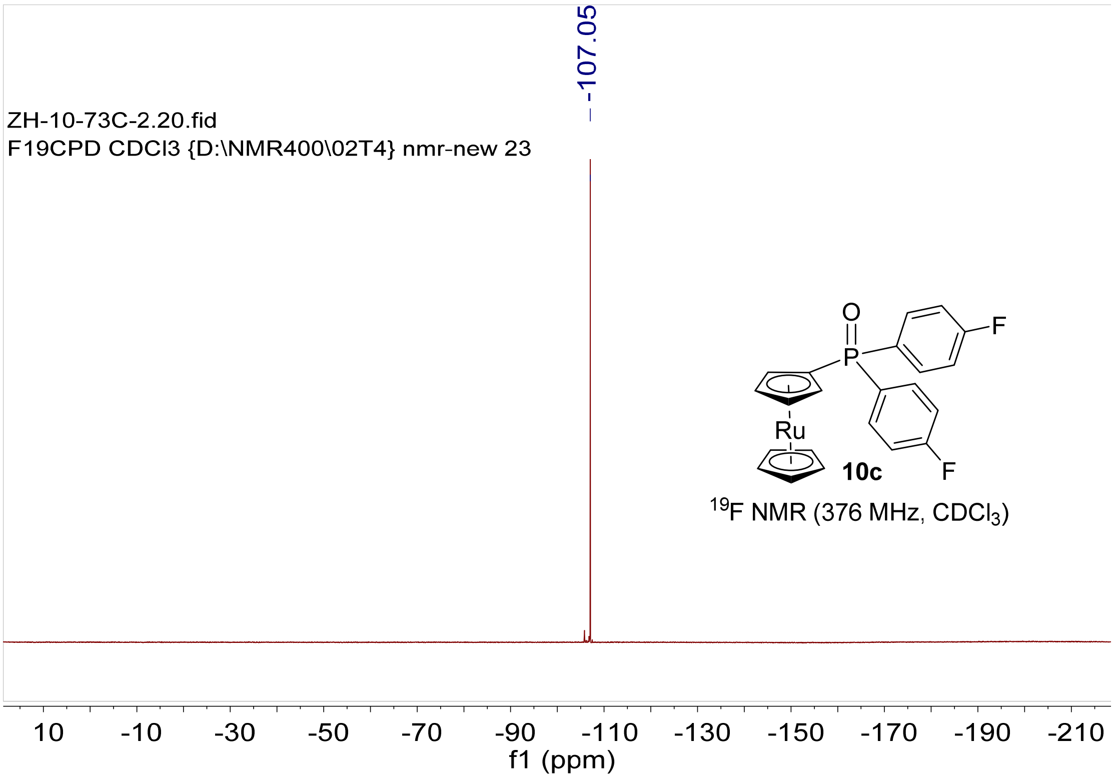

Supplementary Figure 194. ^{19}F NMR spectra of compound 10c

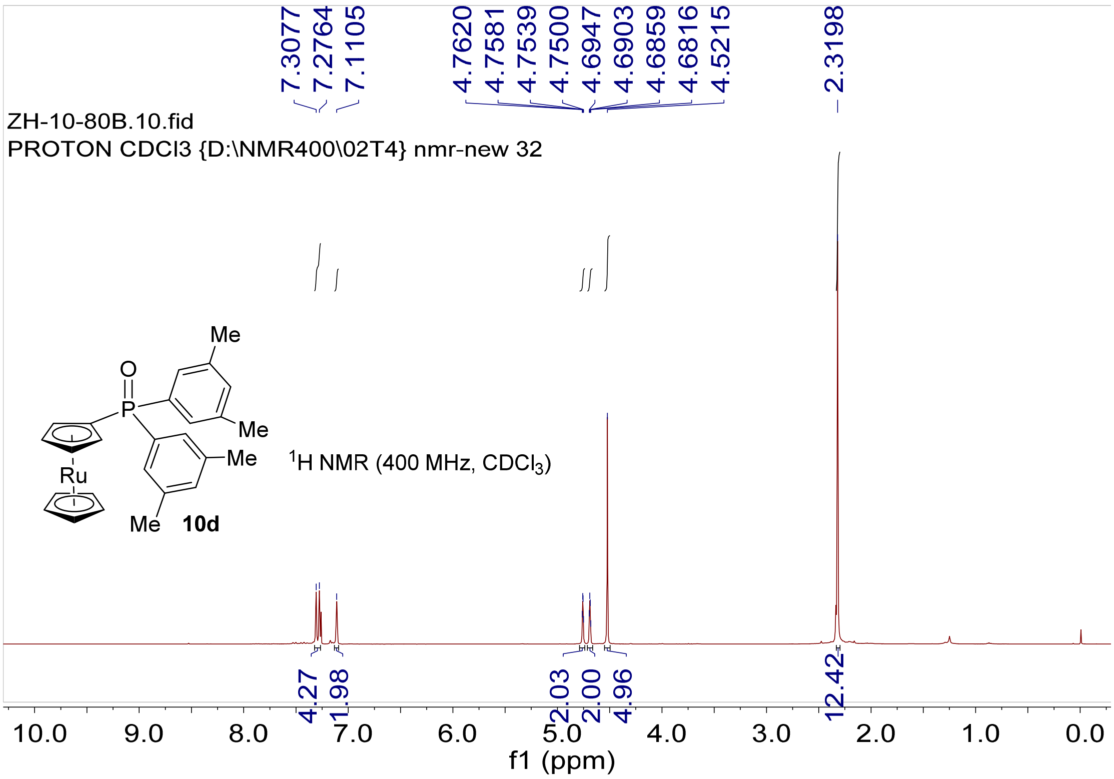

Supplementary Figure 195. ^{1}H NMR spectra of compound 10d

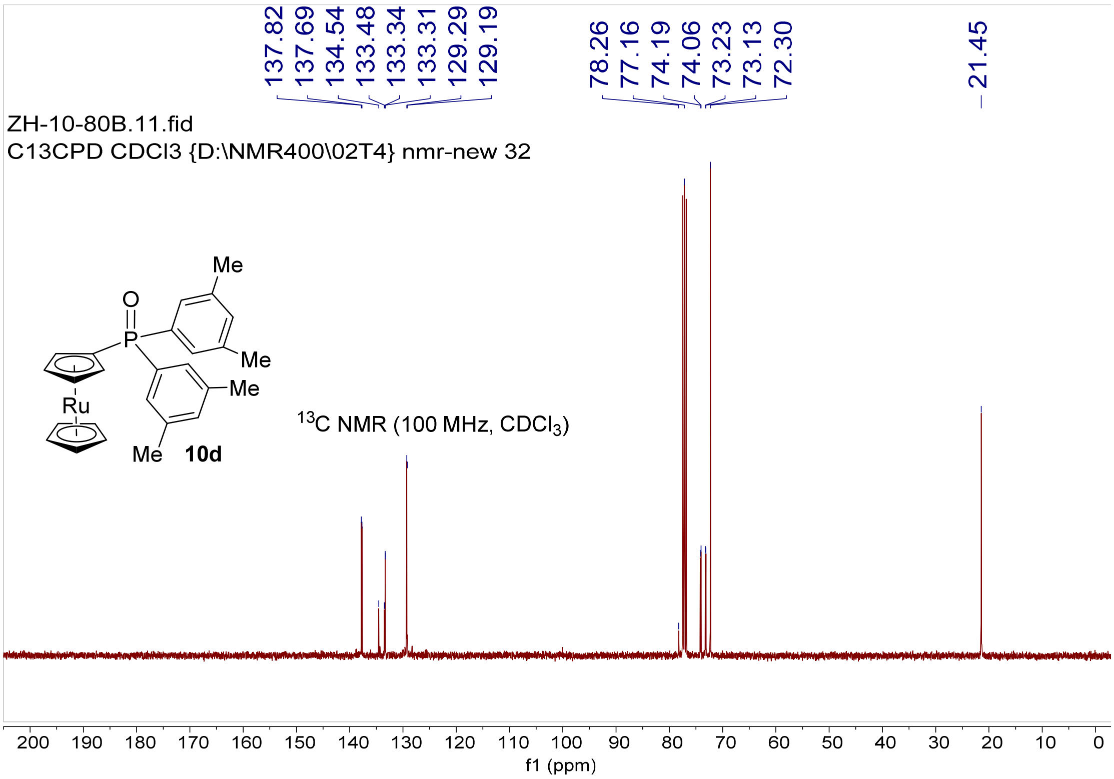

Supplementary Figure 196. ^{13}C NMR spectra of compound 10d

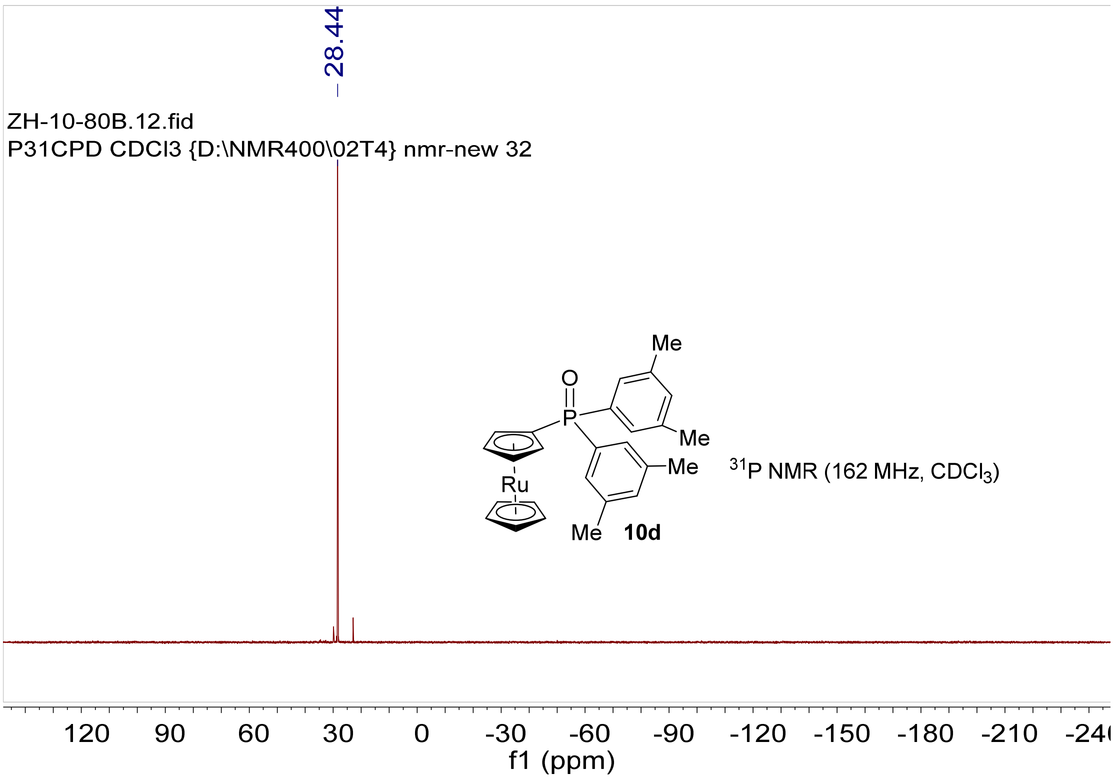

Supplementary Figure 197. ^{31}P NMR spectra of compound 10d

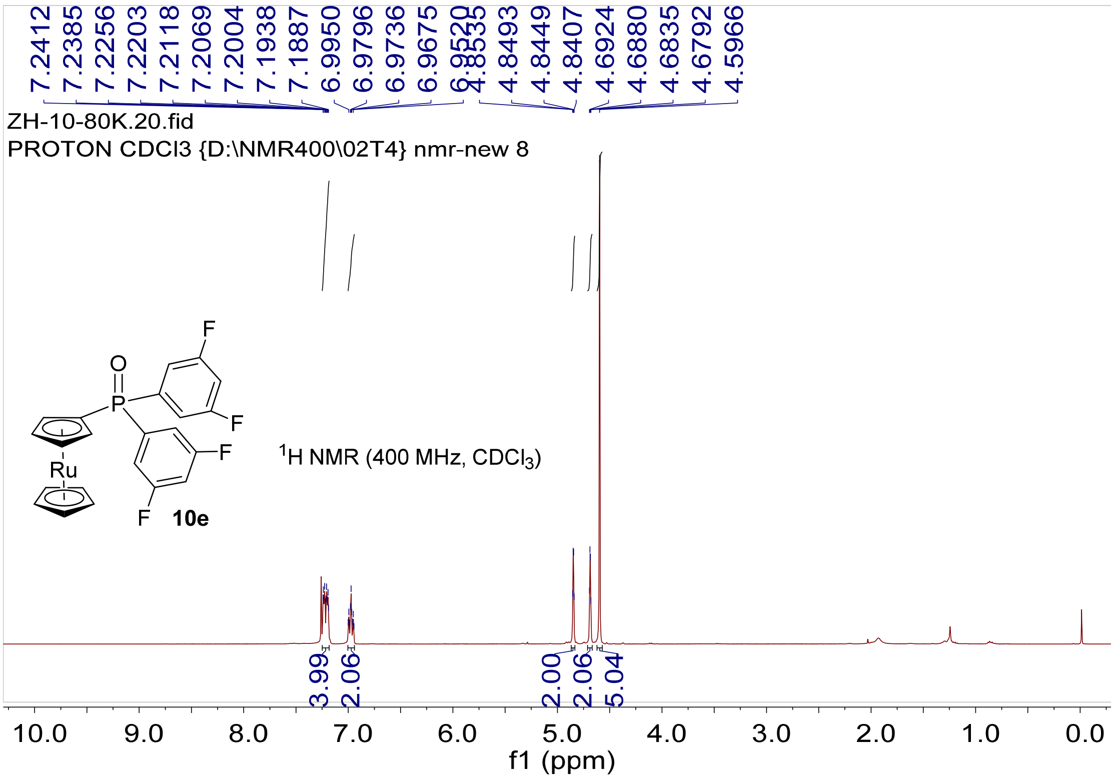

Supplementary Figure 198. ^{1}H NMR spectra of compound 10e

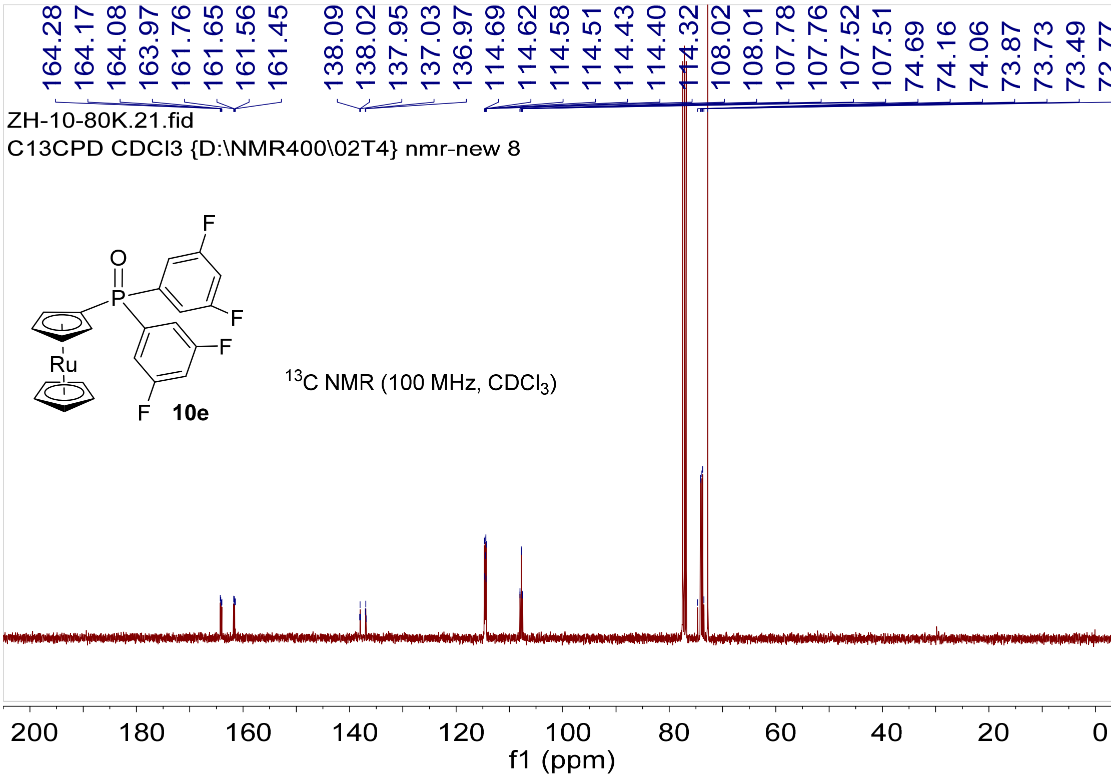

Supplementary Figure 199. ^{13}C NMR spectra of compound 10e

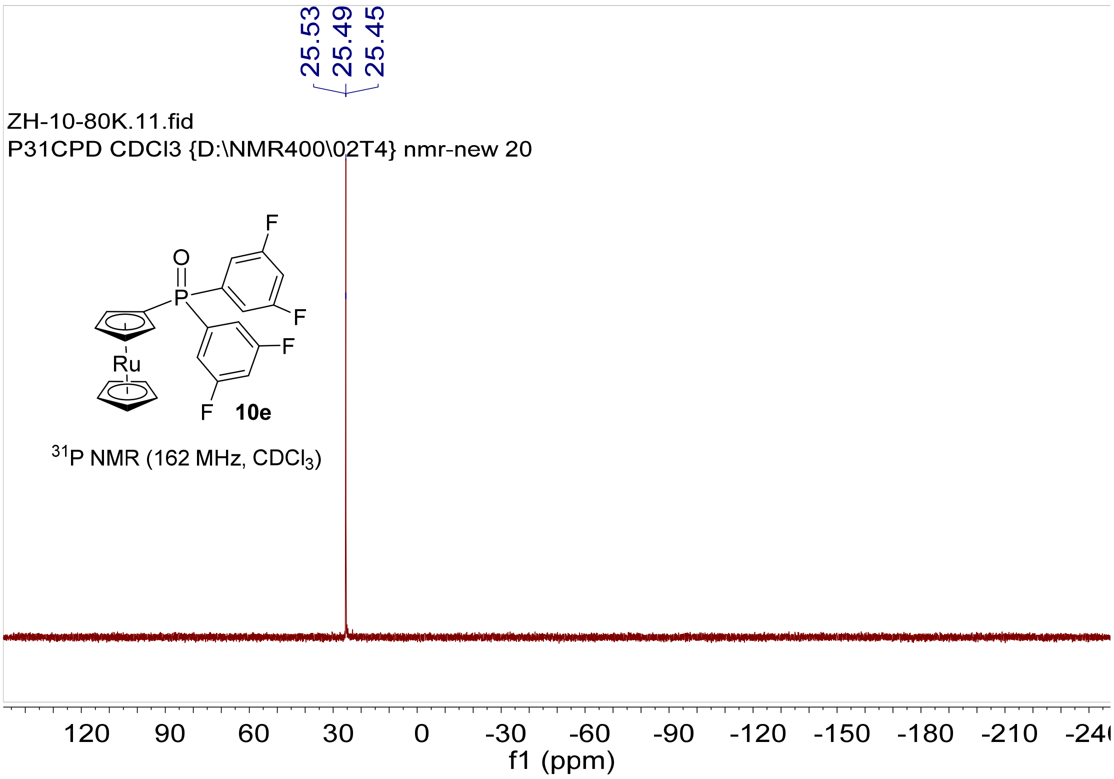

Supplementary Figure 200. ^{31}**P NMR spectra of compound 10e**

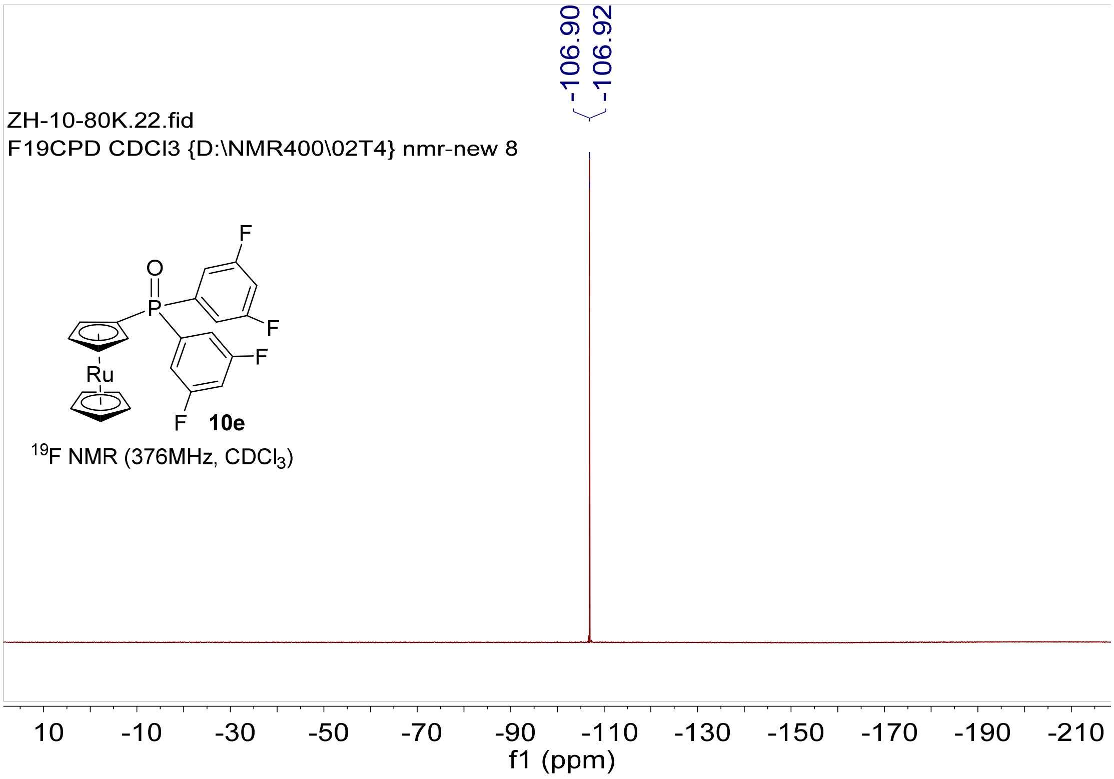

Supplementary Figure 201. ^{19}**F NMR spectra of compound 10e**

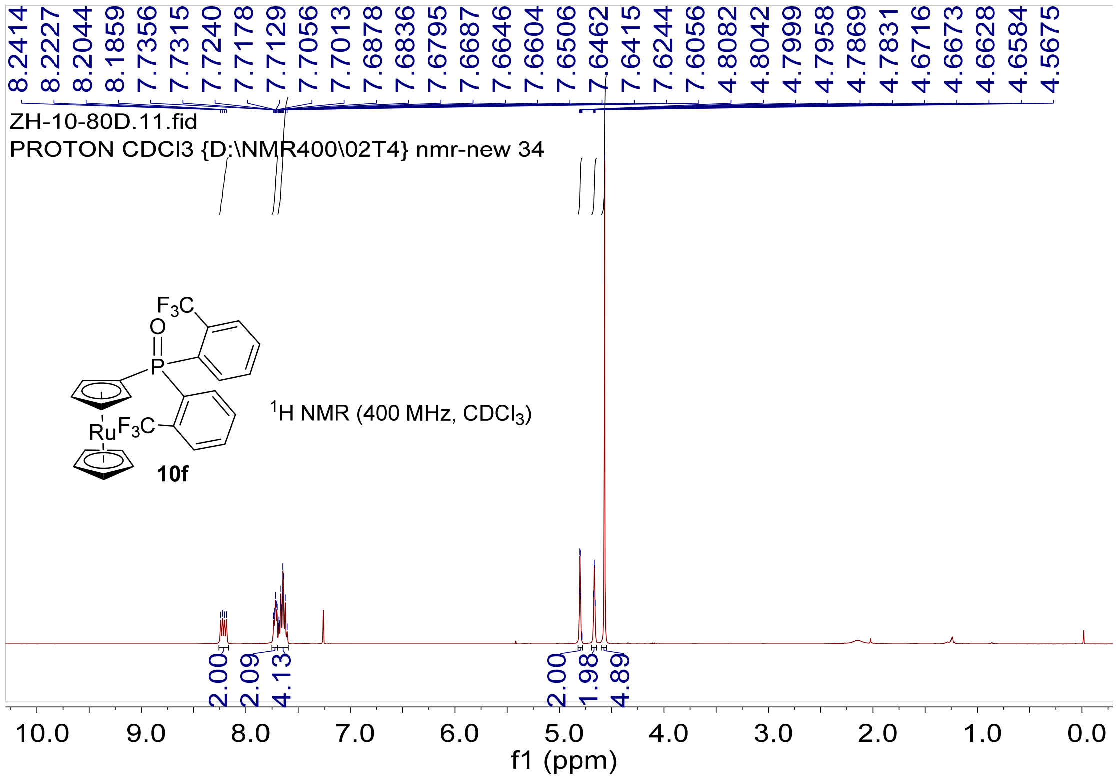

Supplementary Figure 202. ^{1}**H NMR spectra of compound 10f**

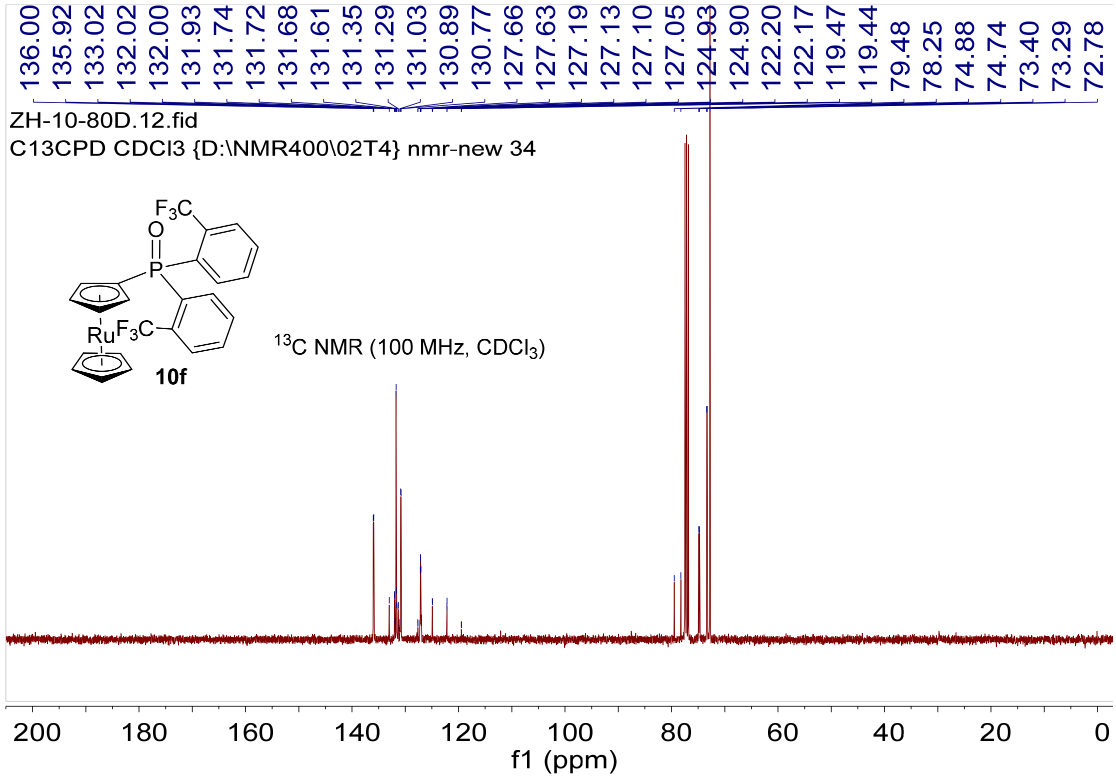

Supplementary Figure 203. ^{13}**C NMR spectra of compound 10f**

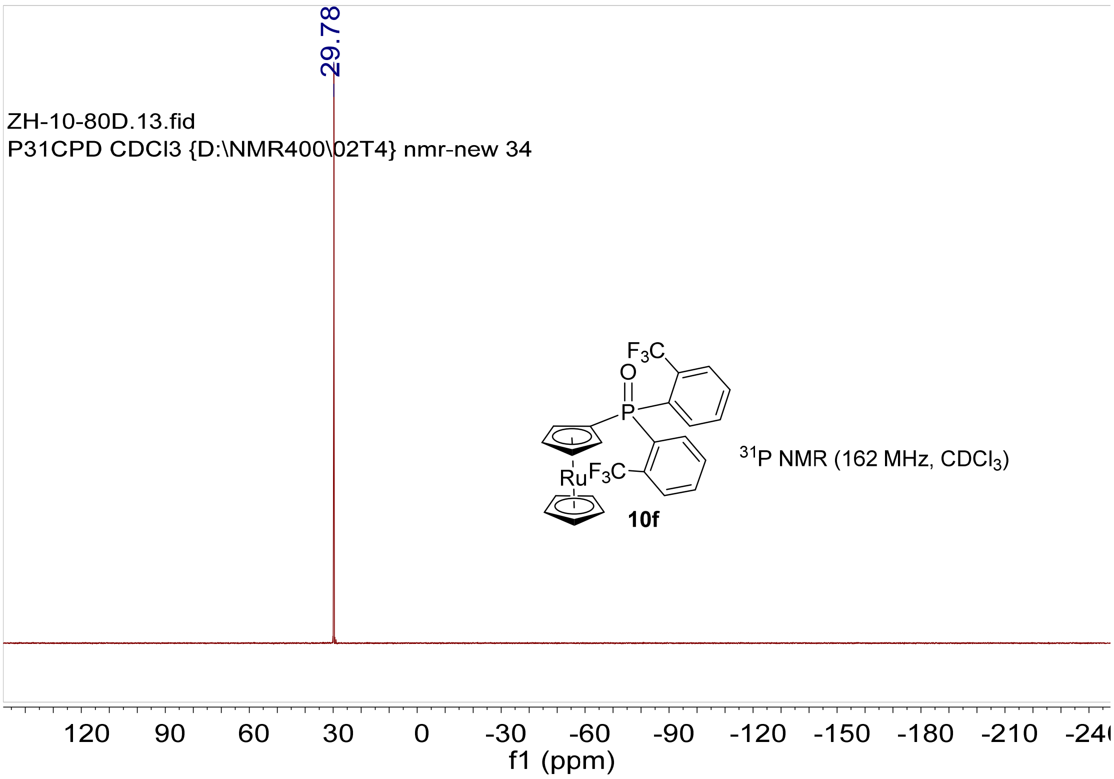

Supplementary Figure 204. ^{31}P NMR spectra of compound 10f

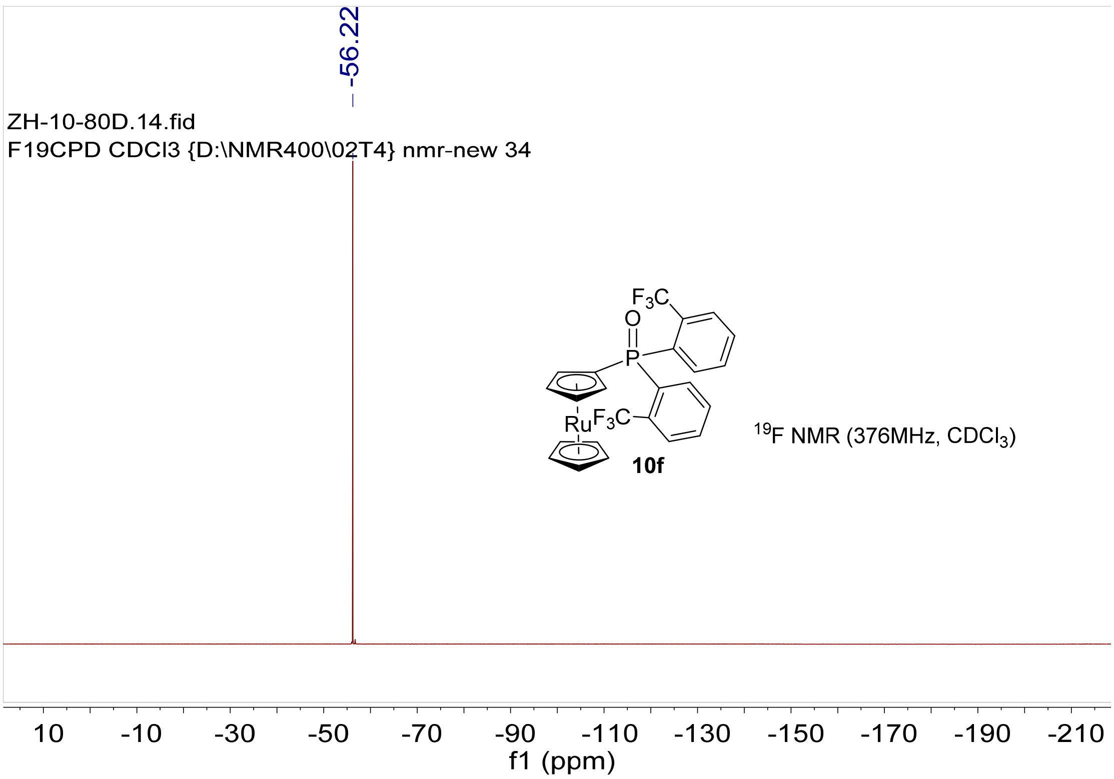

Supplementary Figure 205. ^{19}F NMR spectra of compound 10f

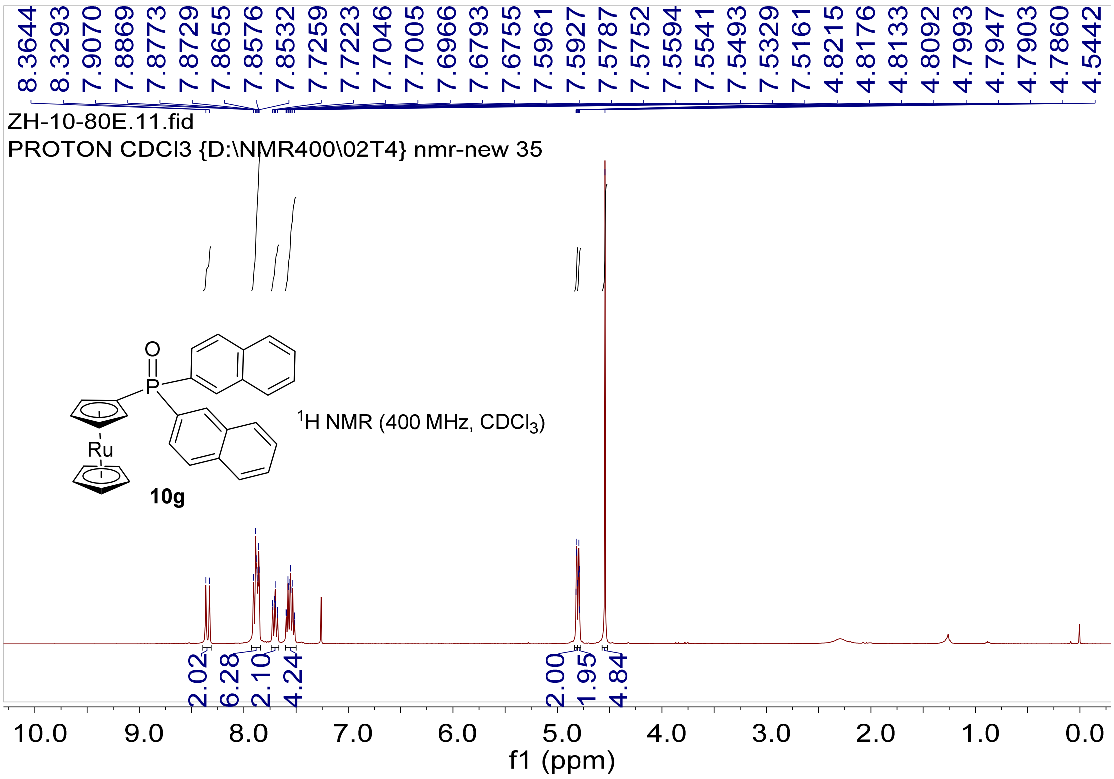

Supplementary Figure 206. ^{1}H NMR spectra of compound 10g

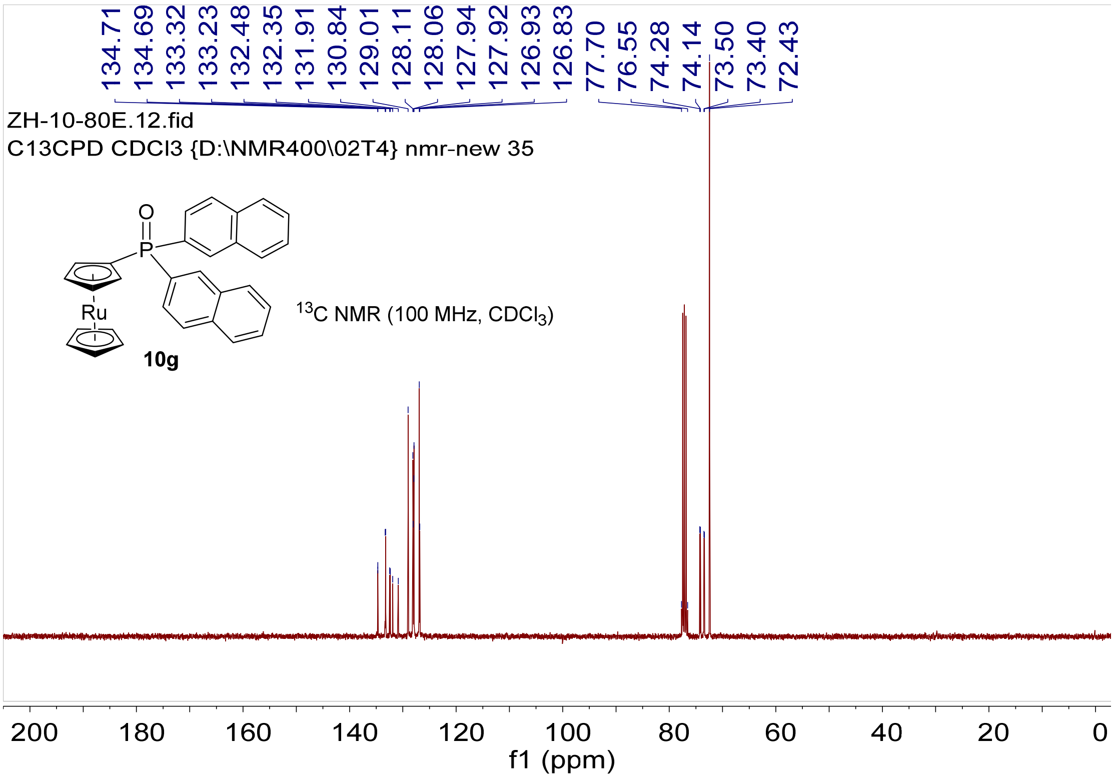

Supplementary Figure 207. ^{13}C NMR spectra of compound 10g

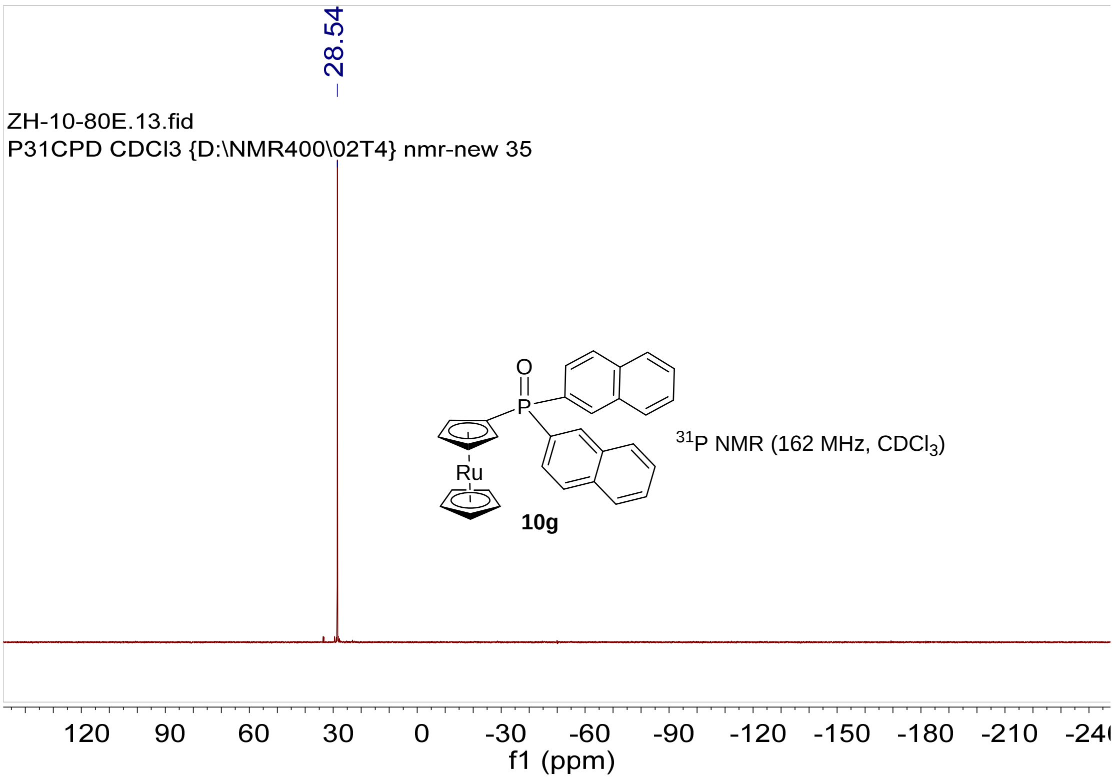

Supplementary Figure 208. ^{31}P NMR spectra of compound 10g

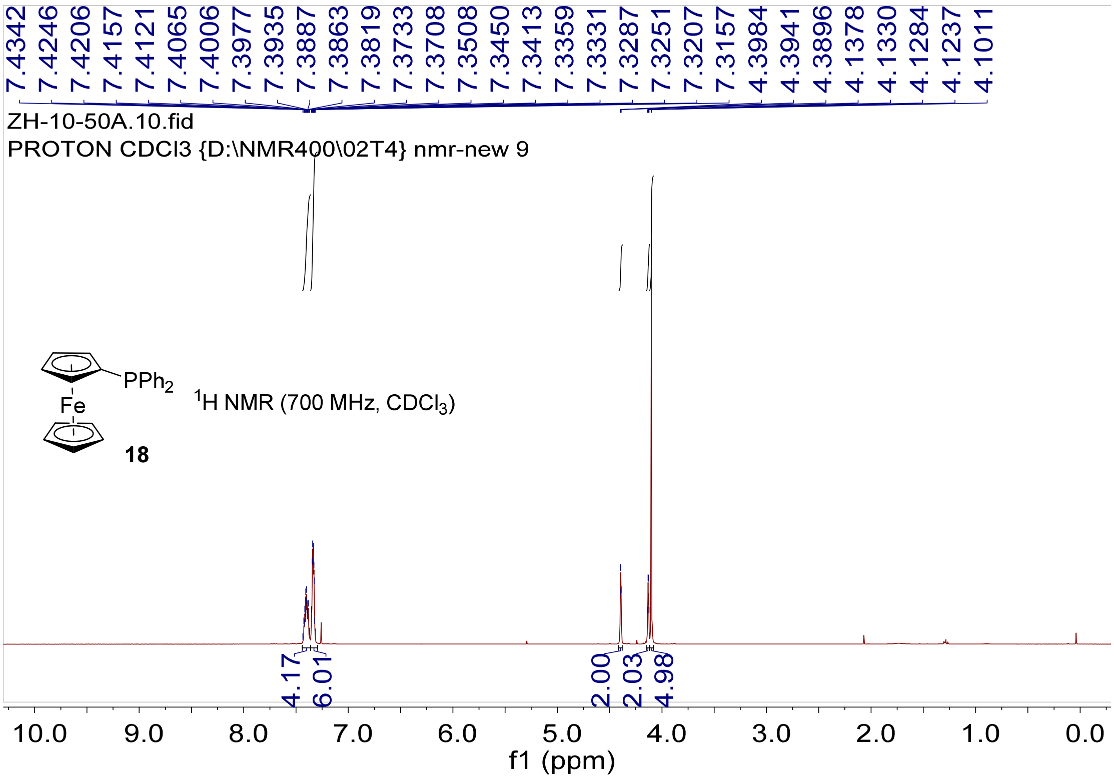

Supplementary Figure 209. ^{1}**H NMR spectra of compound 18**

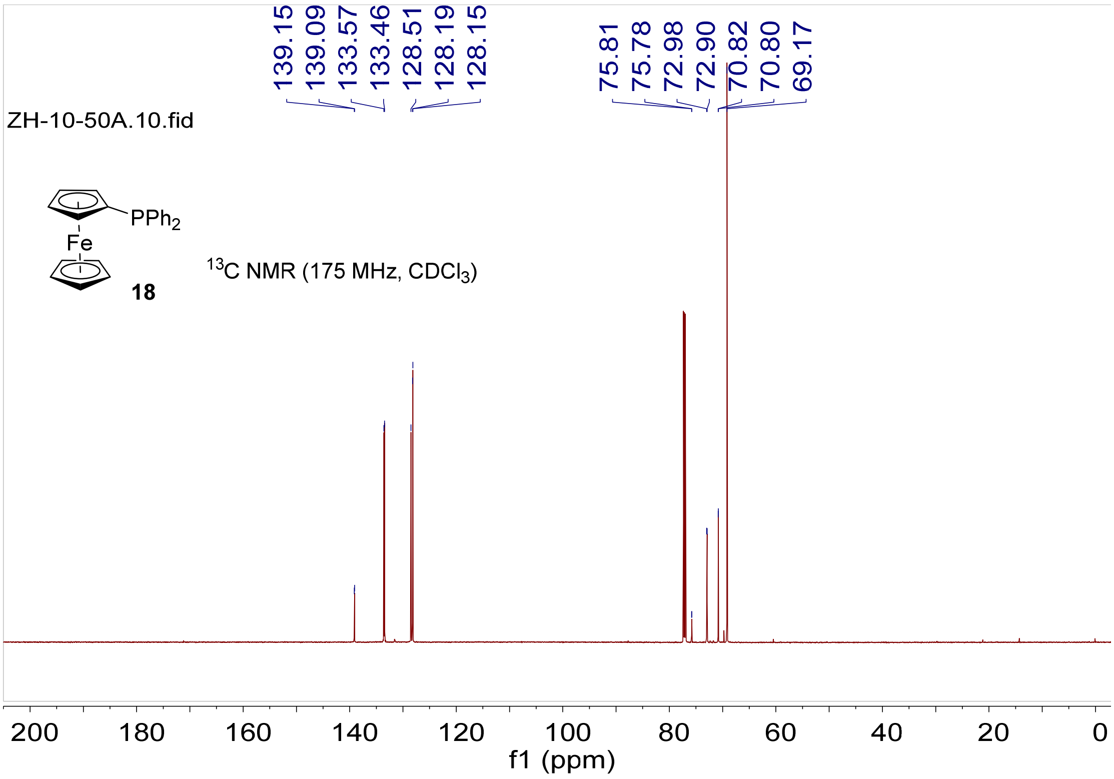

Supplementary Figure 210. ^{13}**C NMR spectra of compound 18**

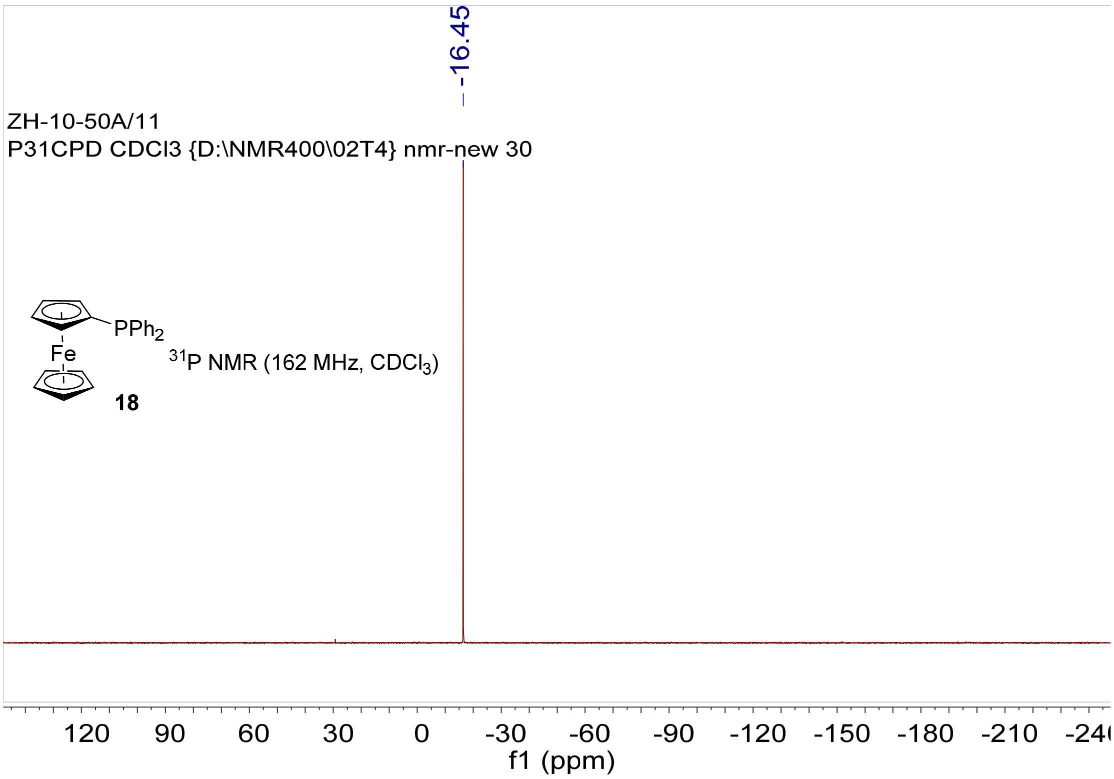

Supplementary Figure 211. ^{31}P NMR spectra of compound 18

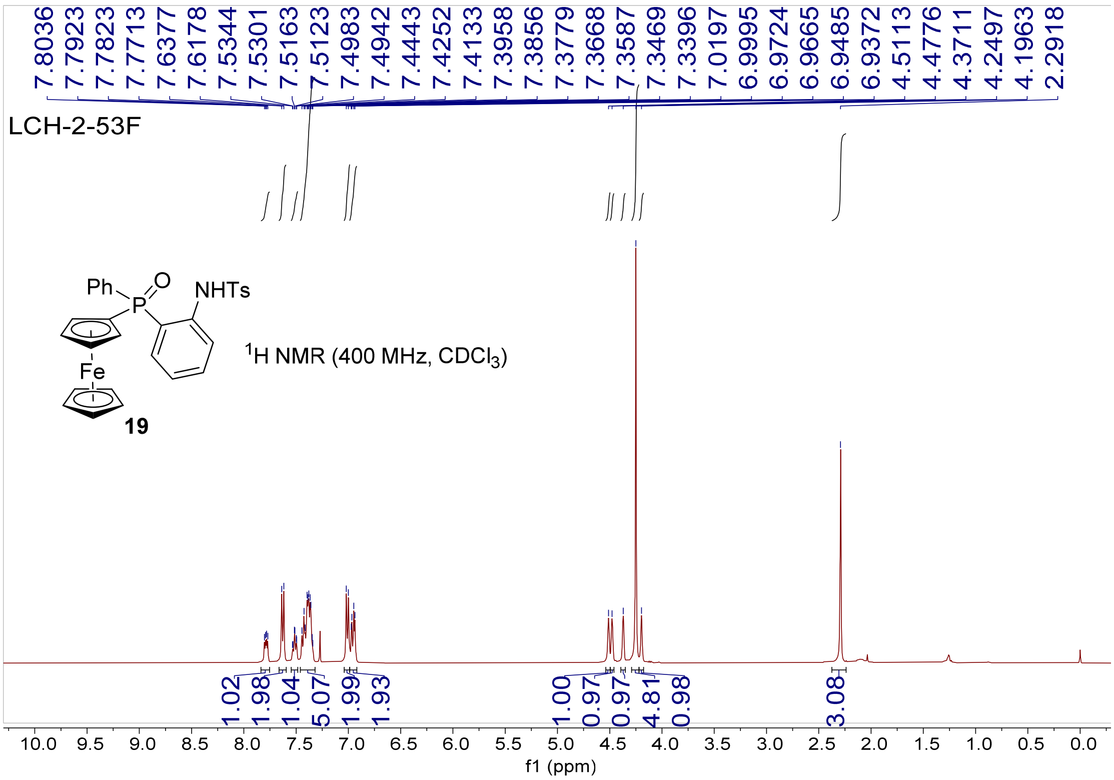

Supplementary Figure 212. ^{1}H NMR spectra of compound 19

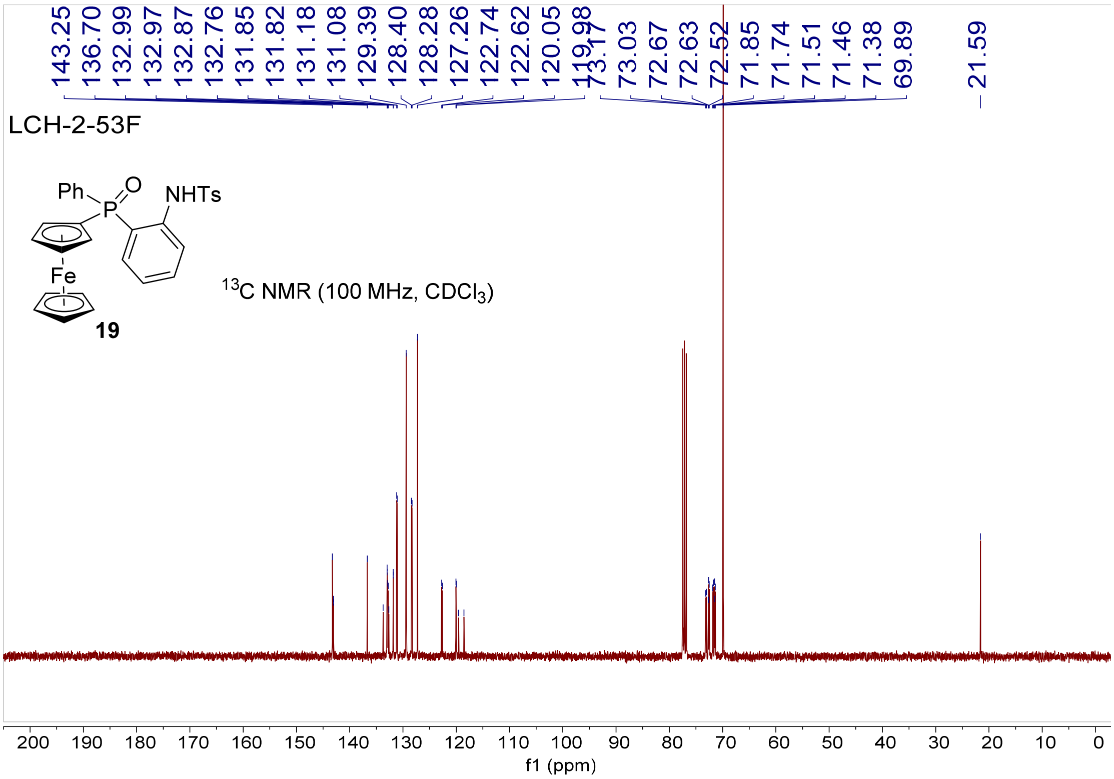

Supplementary Figure 213. ^{13}C NMR spectra of compound 19

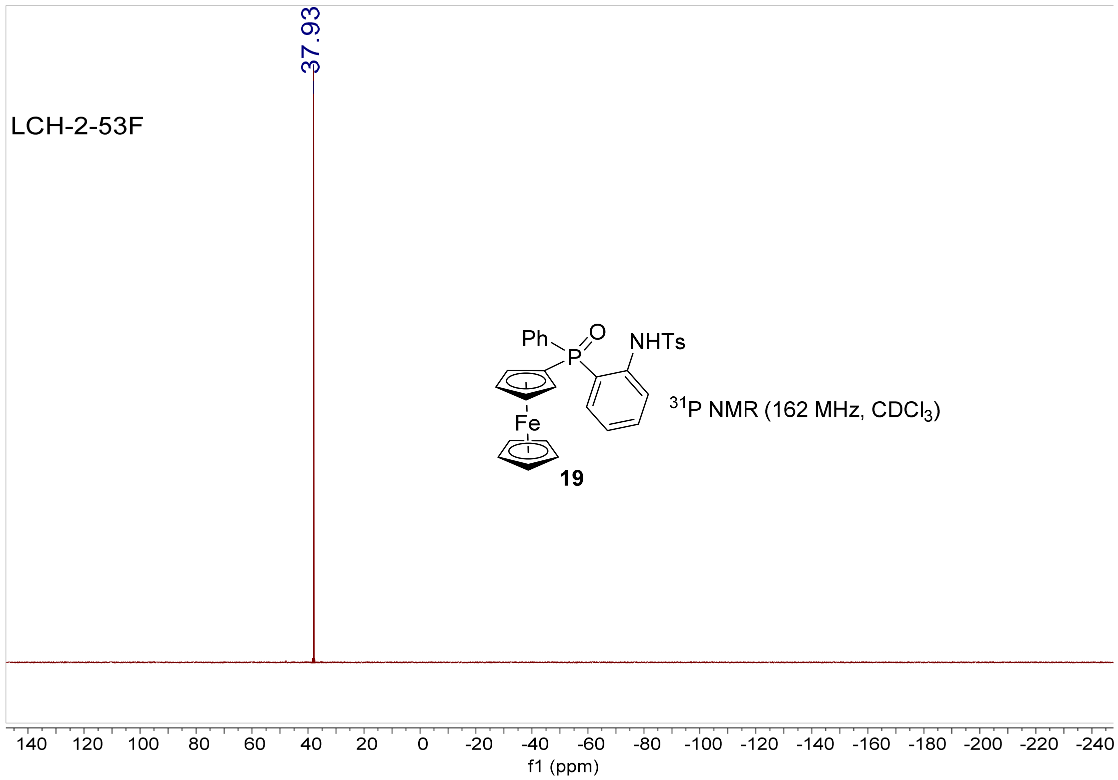

Supplementary Figure 214. ^{31}P NMR spectra of compound 19

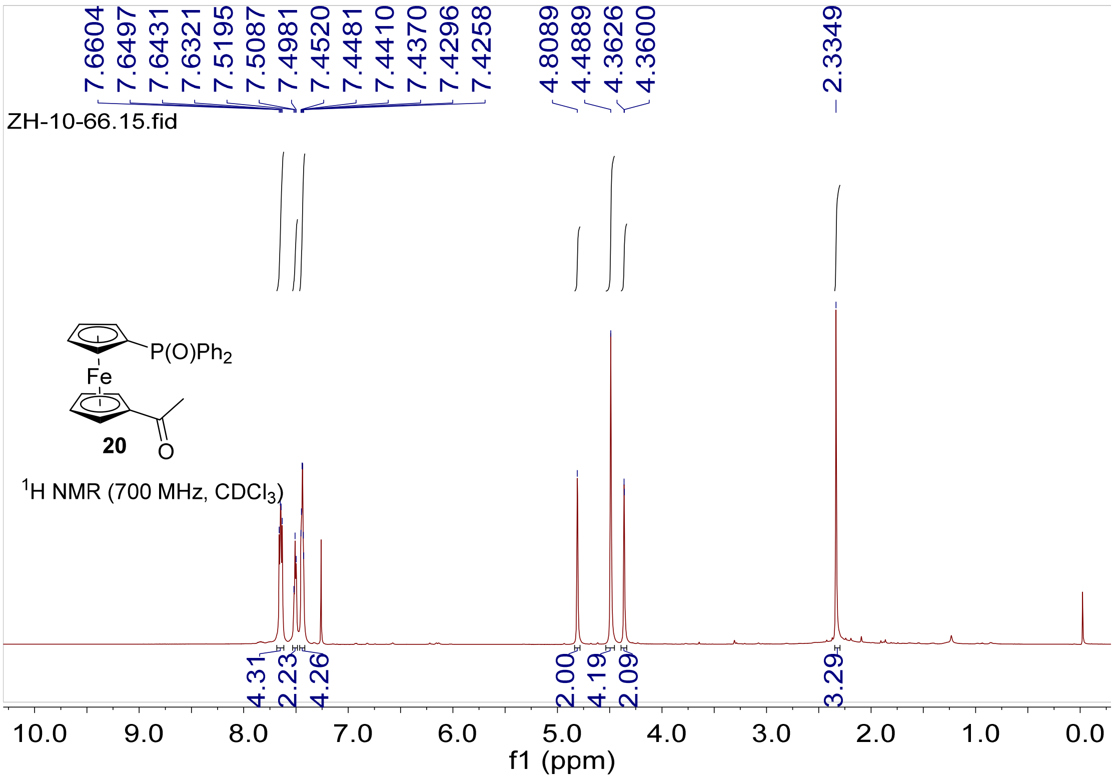

Supplementary Figure 215. ^{1}H NMR spectra of compound 20

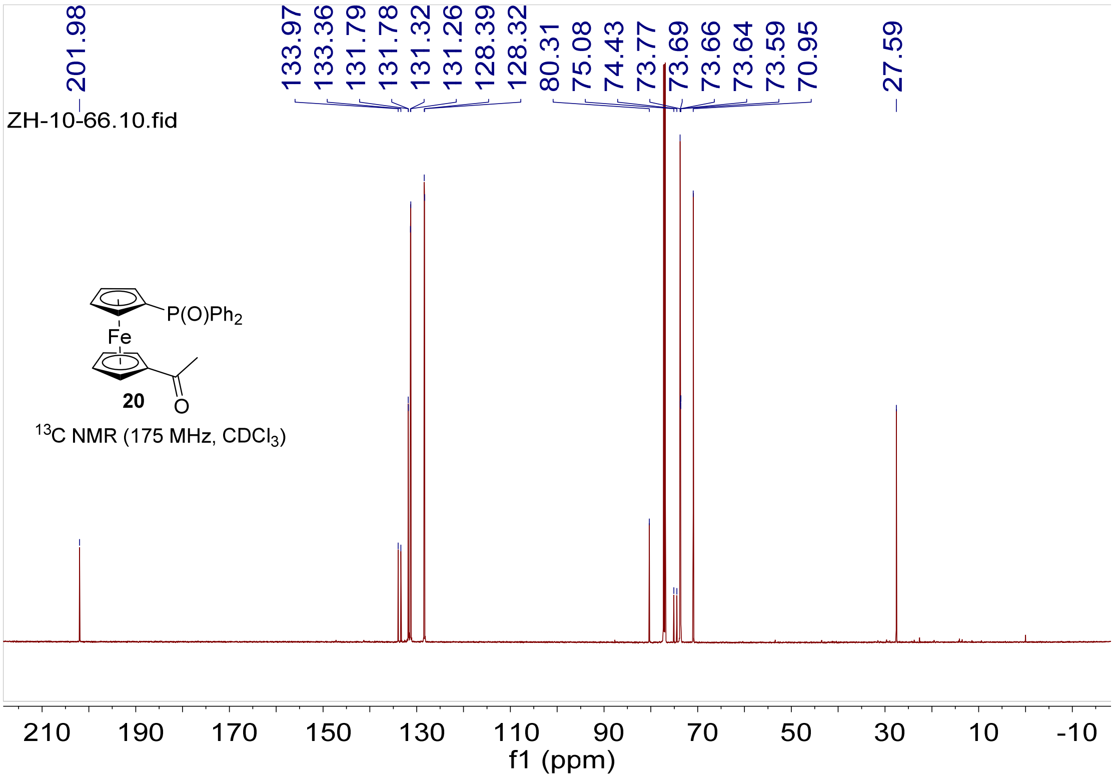

Supplementary Figure 216. ^{13}C NMR spectra of compound 20

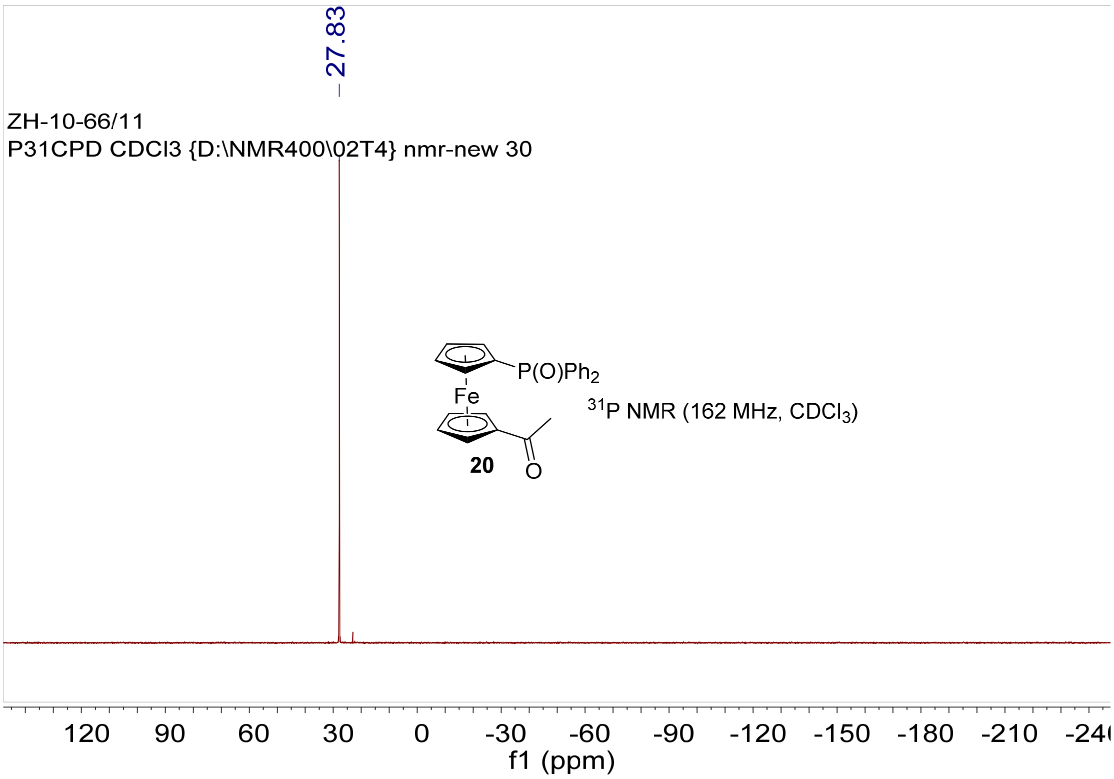

Supplementary Figure 217. ^{3}P NMR spectra of compound 20

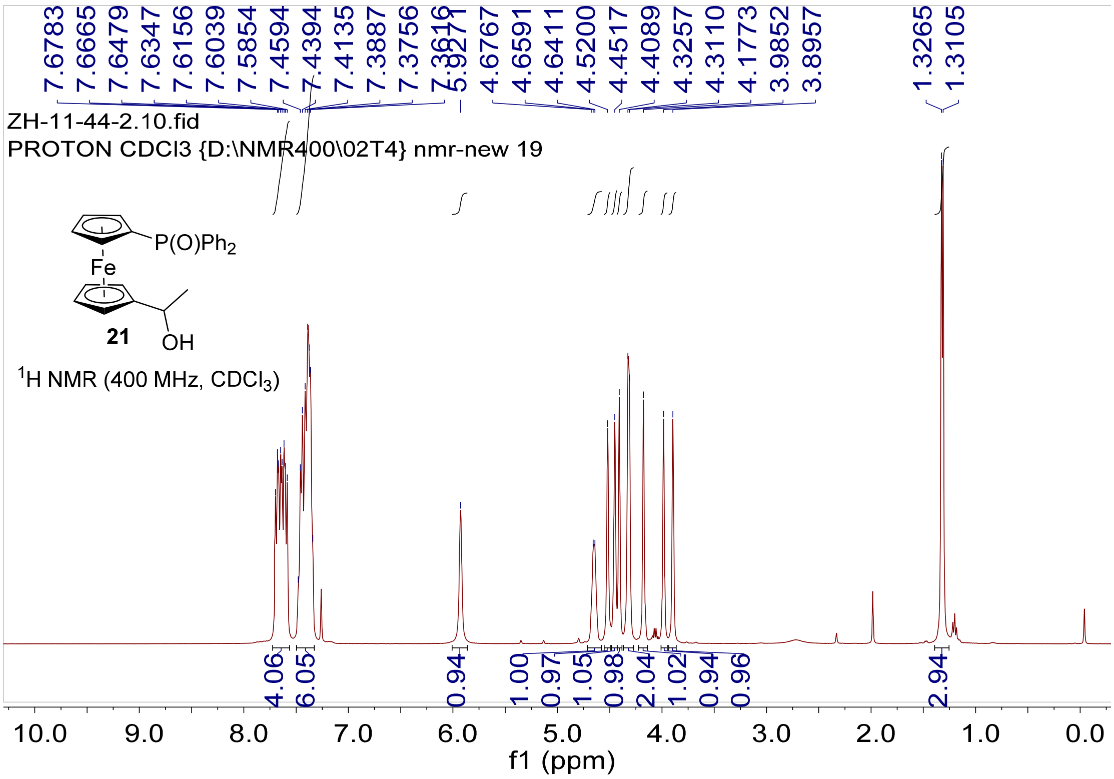

Supplementary Figure 218. ^{1}H NMR spectra of compound 21

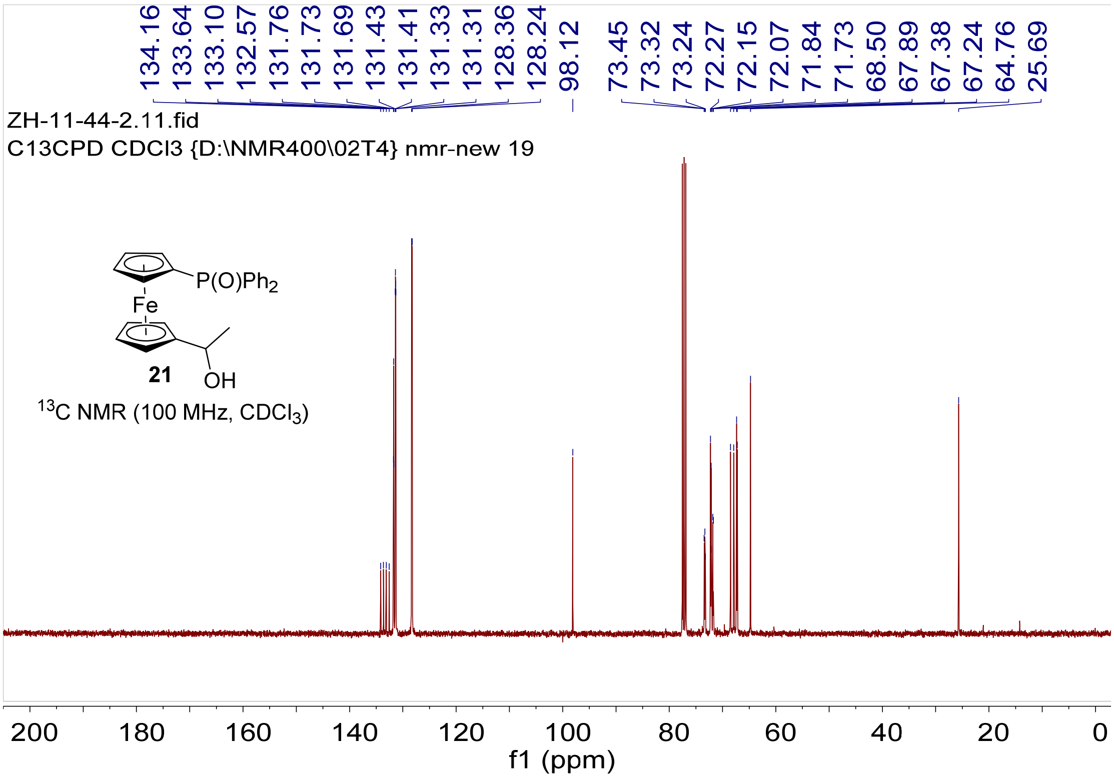

Supplementary Figure 219. ^{13}C NMR spectra of compound 21

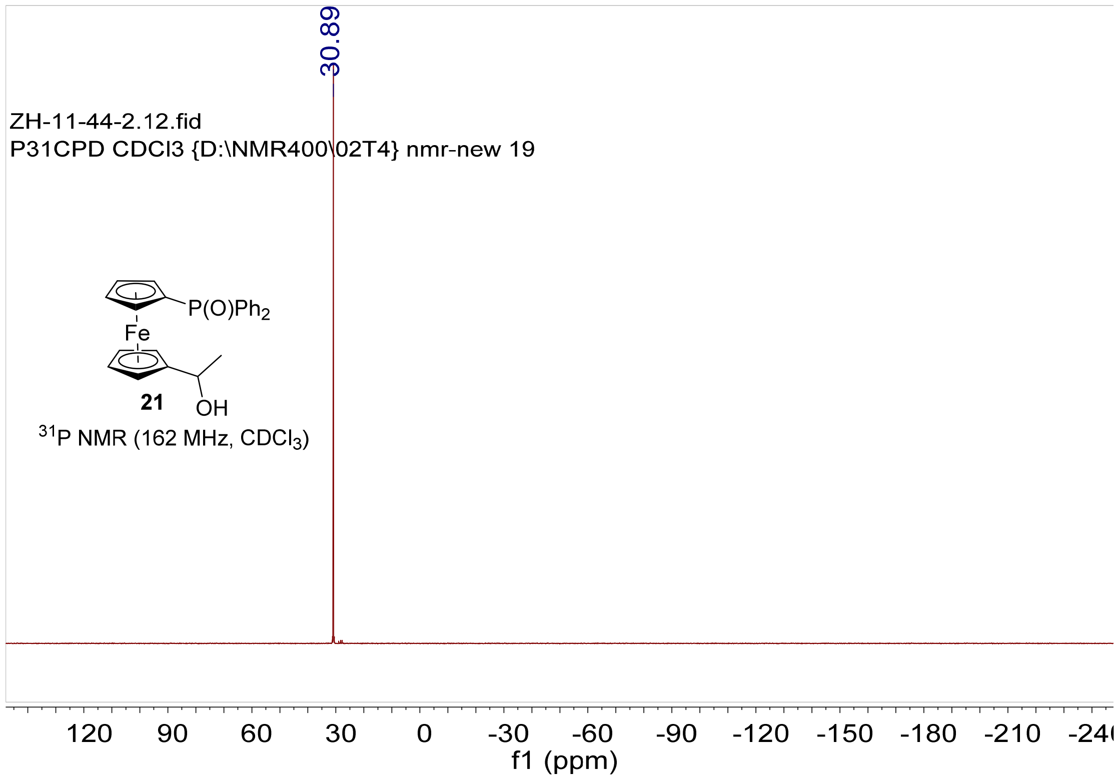

Supplementary Figure 220. ^{31}P NMR spectra of compound 21

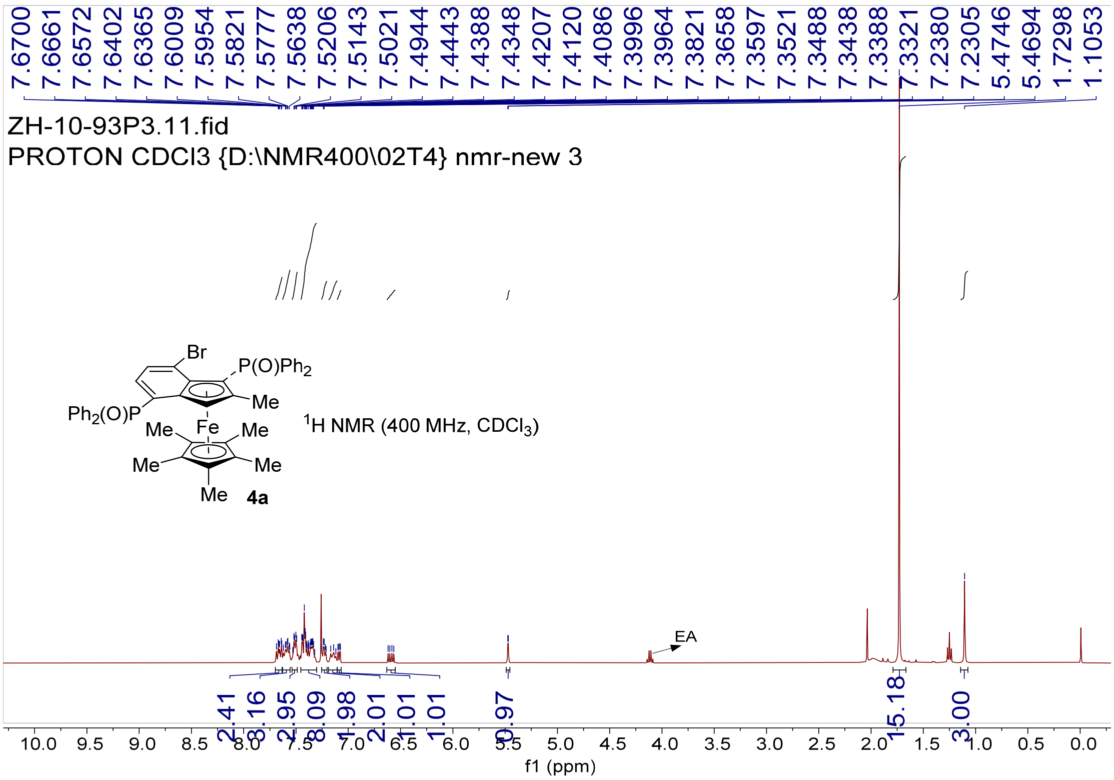

Supplementary Figure 221. ^{1}**H NMR spectra of compound 4a**

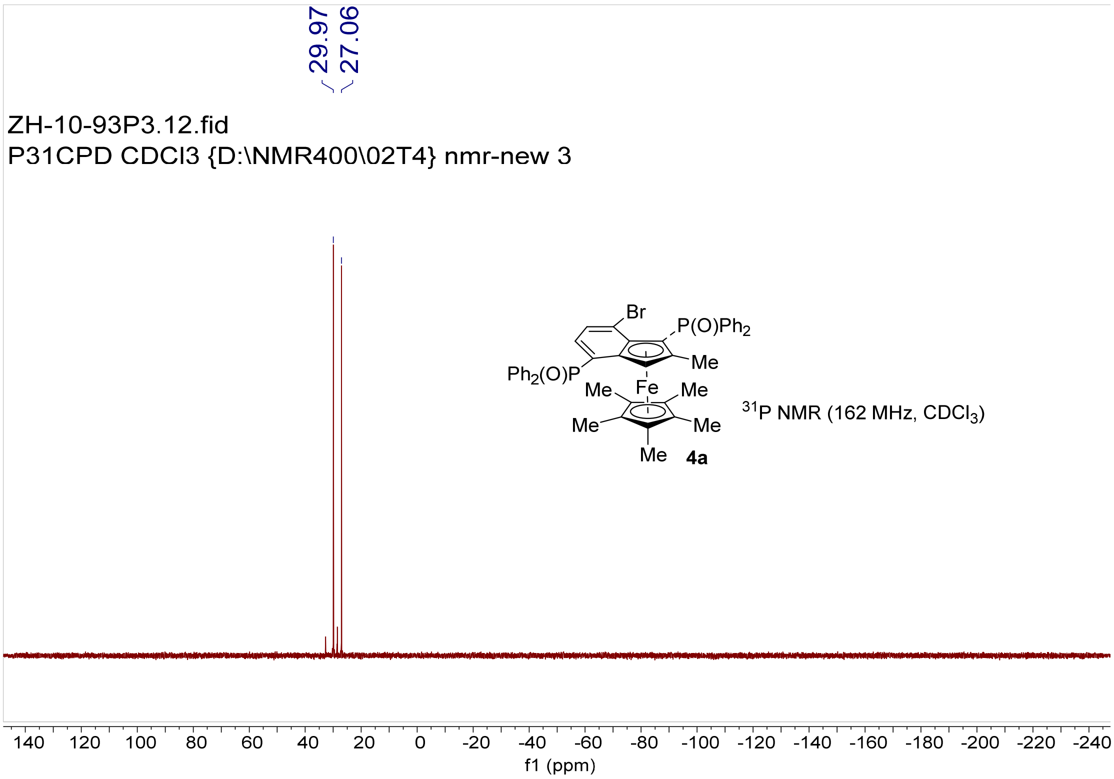

Supplementary Figure 222. ^{31}**P NMR spectra of compound 4a**

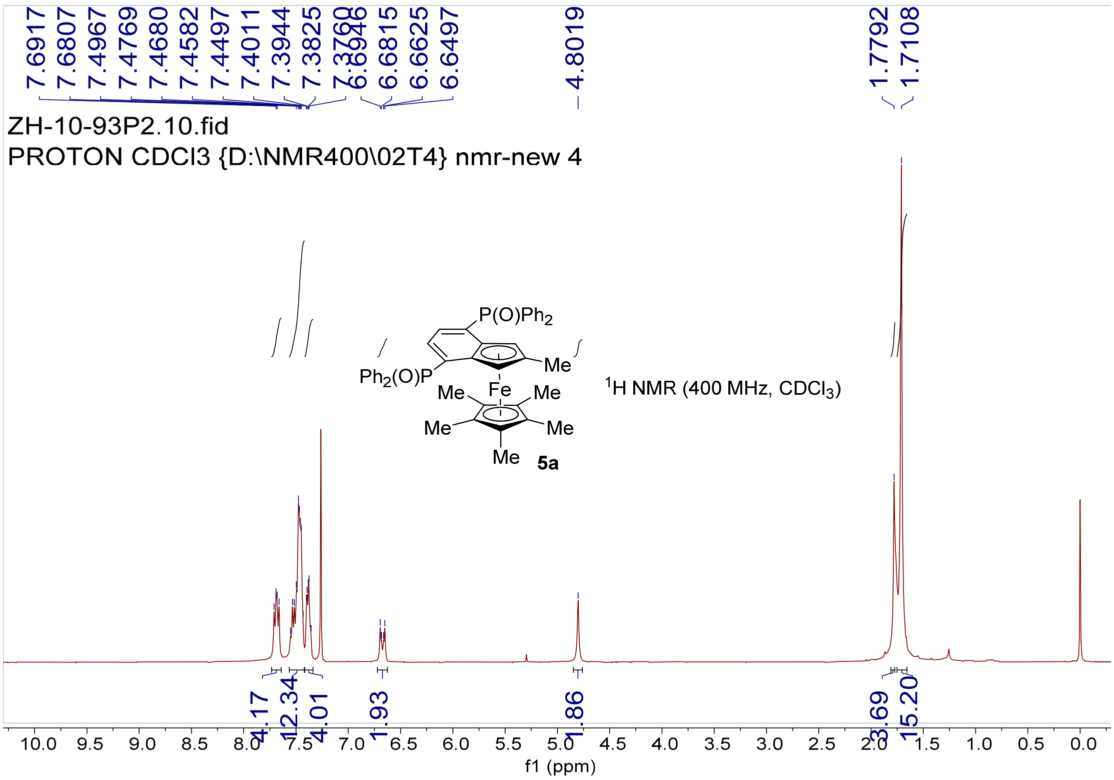

Supplementary Figure 223. ^{1}**H NMR spectra of compound 5a**

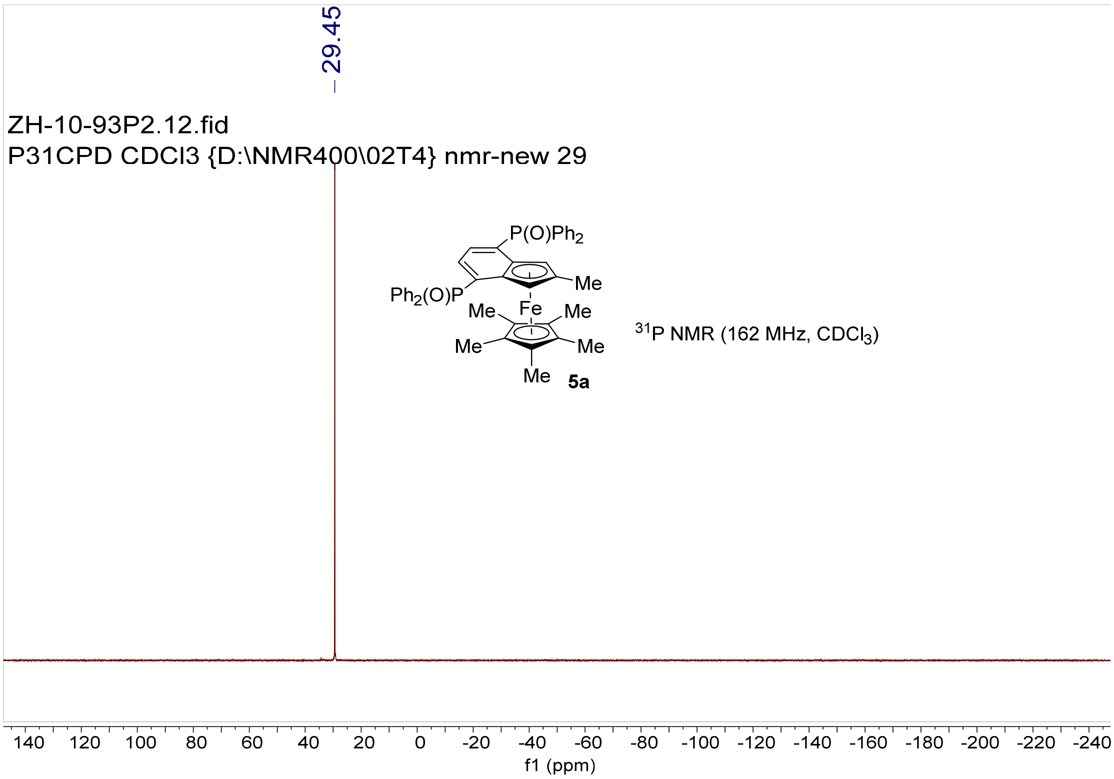

Supplementary Figure 224. ^{31}**P NMR spectra of compound 5a**

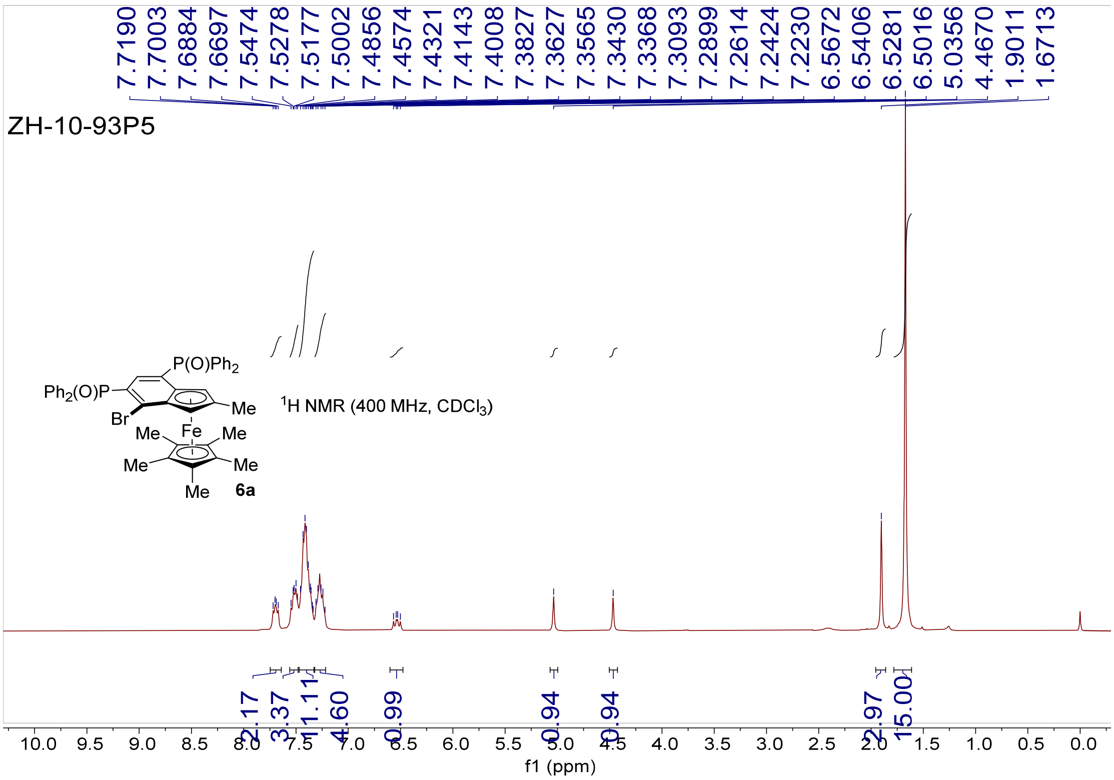

Supplementary Figure 225. ^{1}H NMR spectra of compound 6a

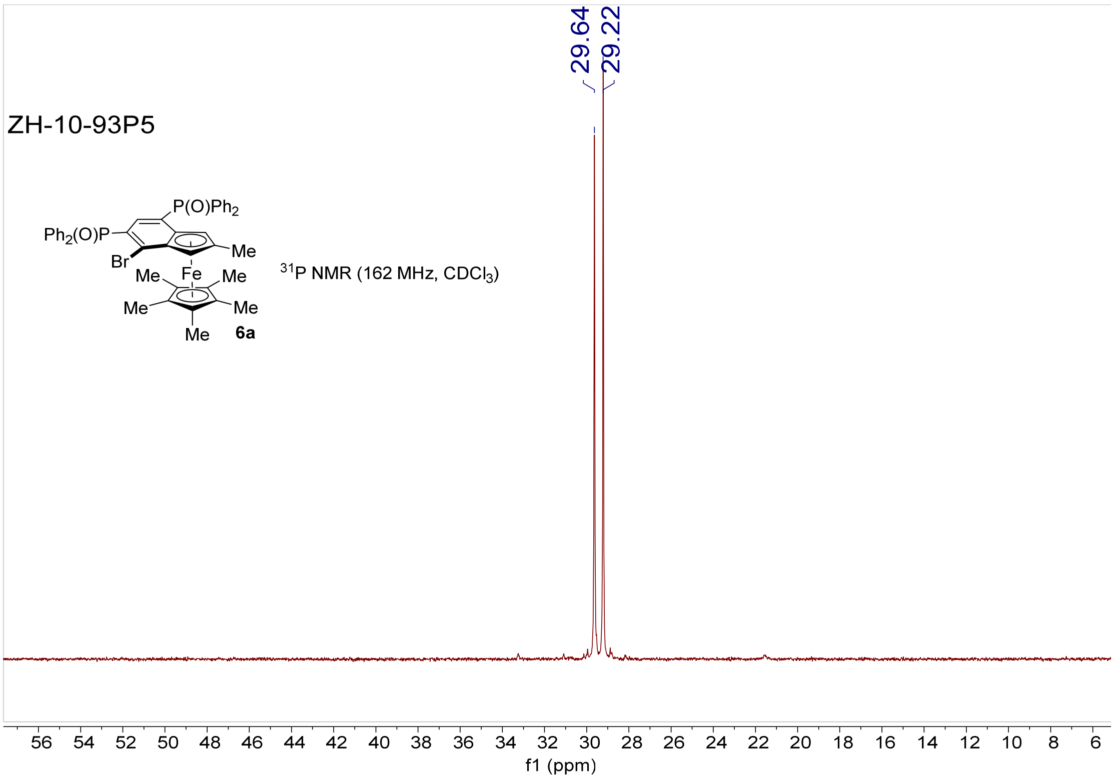

Supplementary Figure 226. ^{31}P NMR spectra of compound 6a

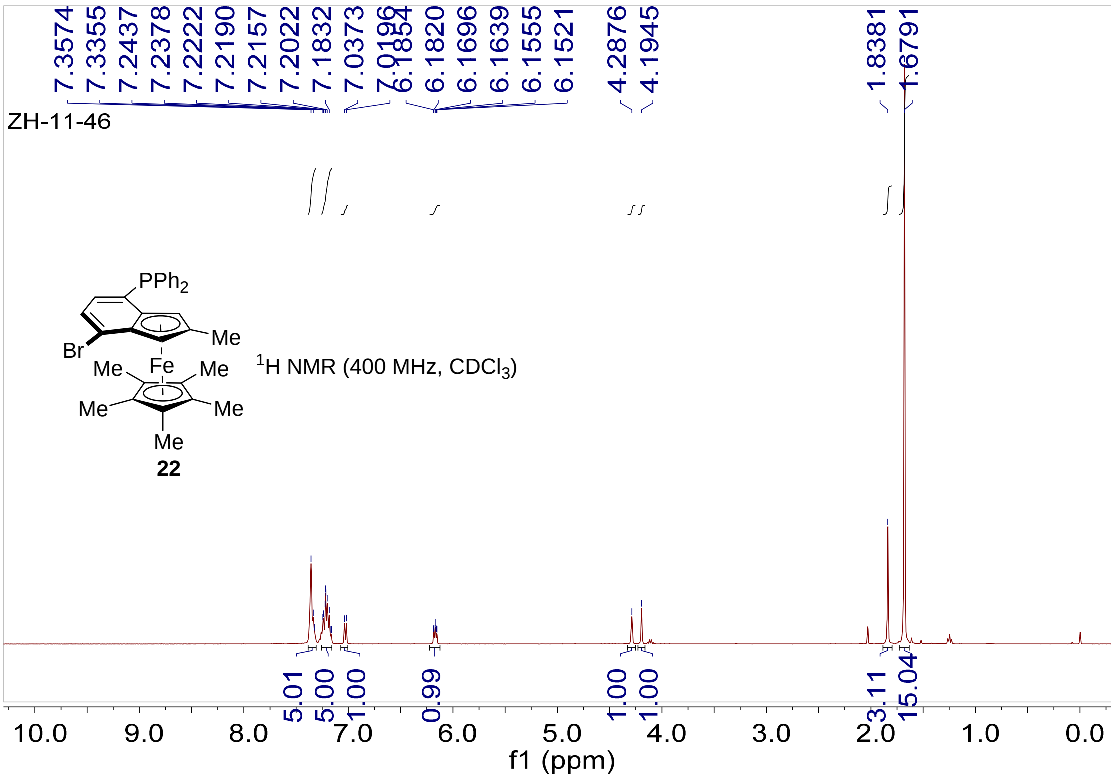

Supplementary Figure 227. ^{1}H NMR spectra of compound 22

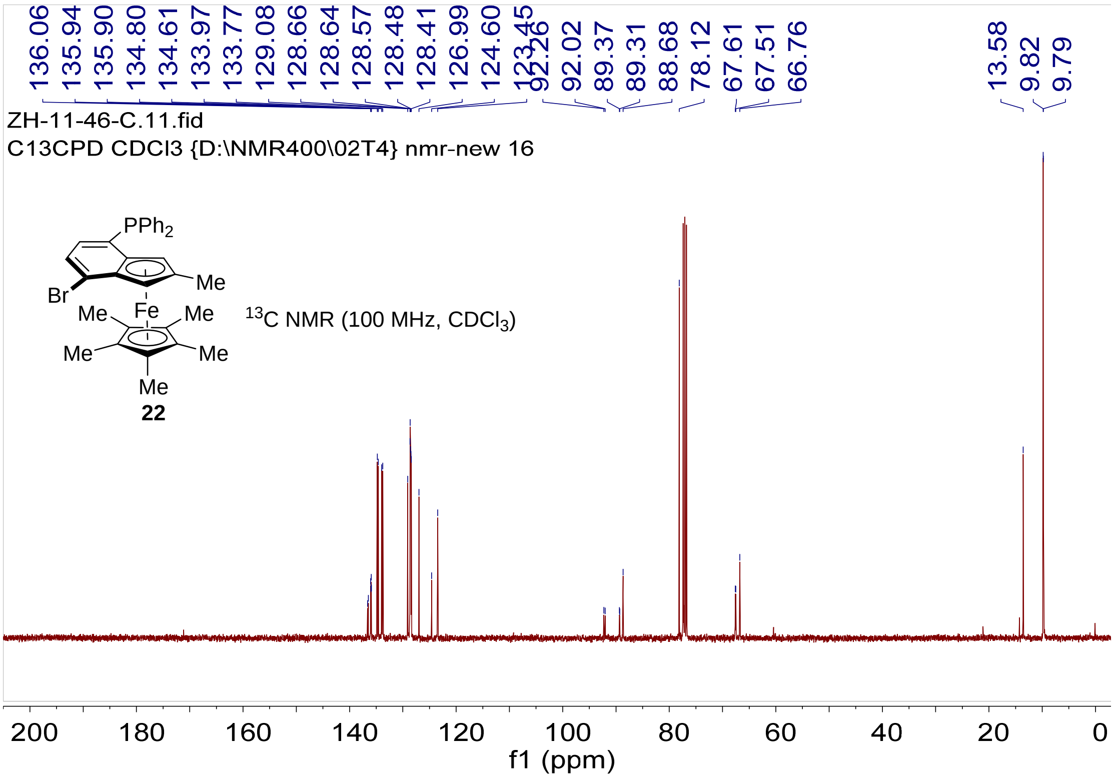

Supplementary Figure 228. ^{13}C NMR spectra of compound 22

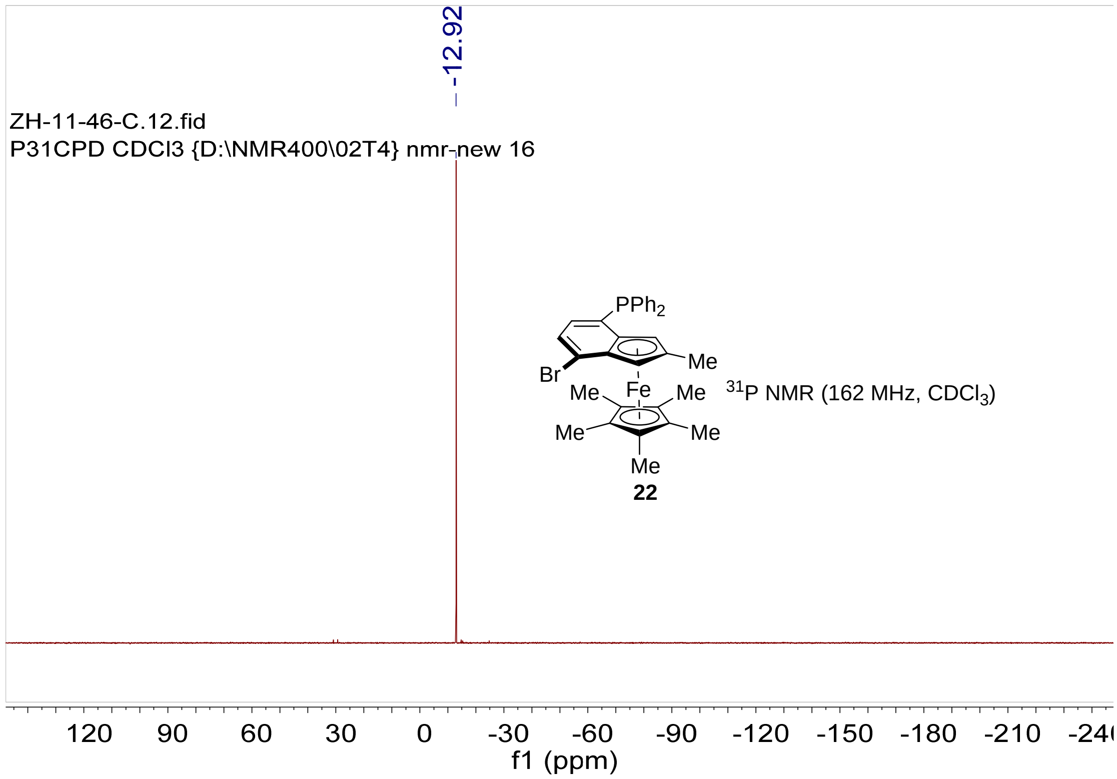

Supplementary Figure 229. ^{31}P NMR spectra of compound 22

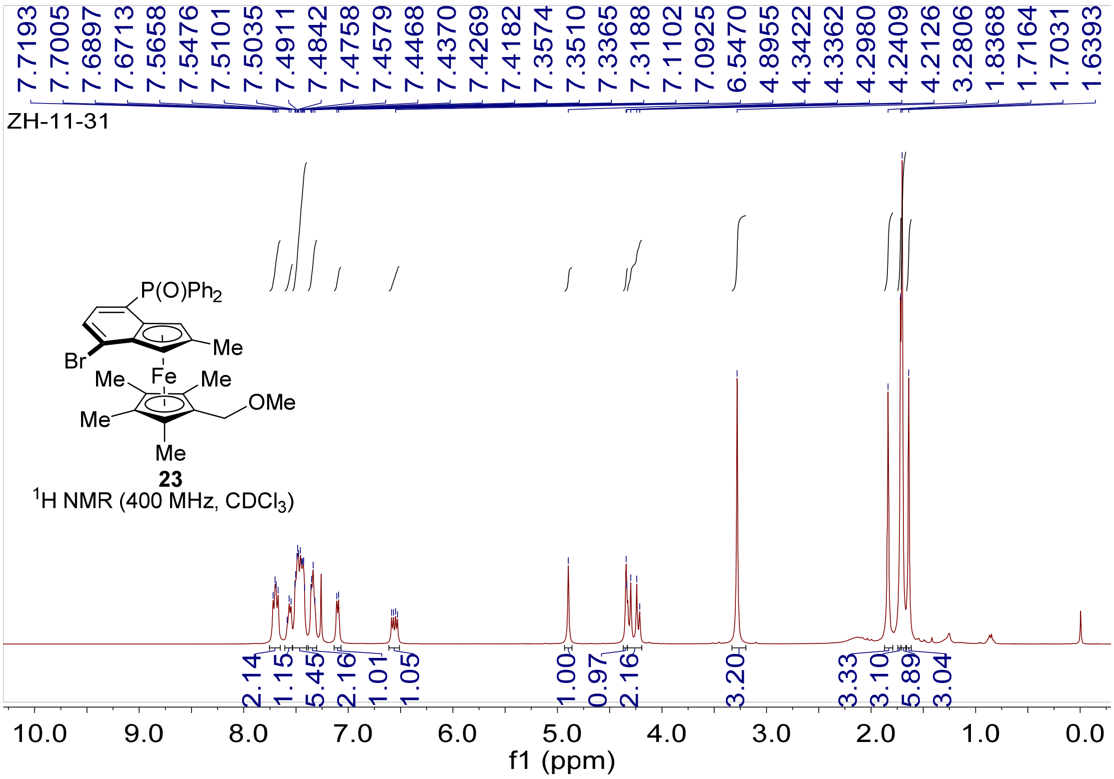

Supplementary Figure 230. ^{1}**H NMR spectra of compound 23**

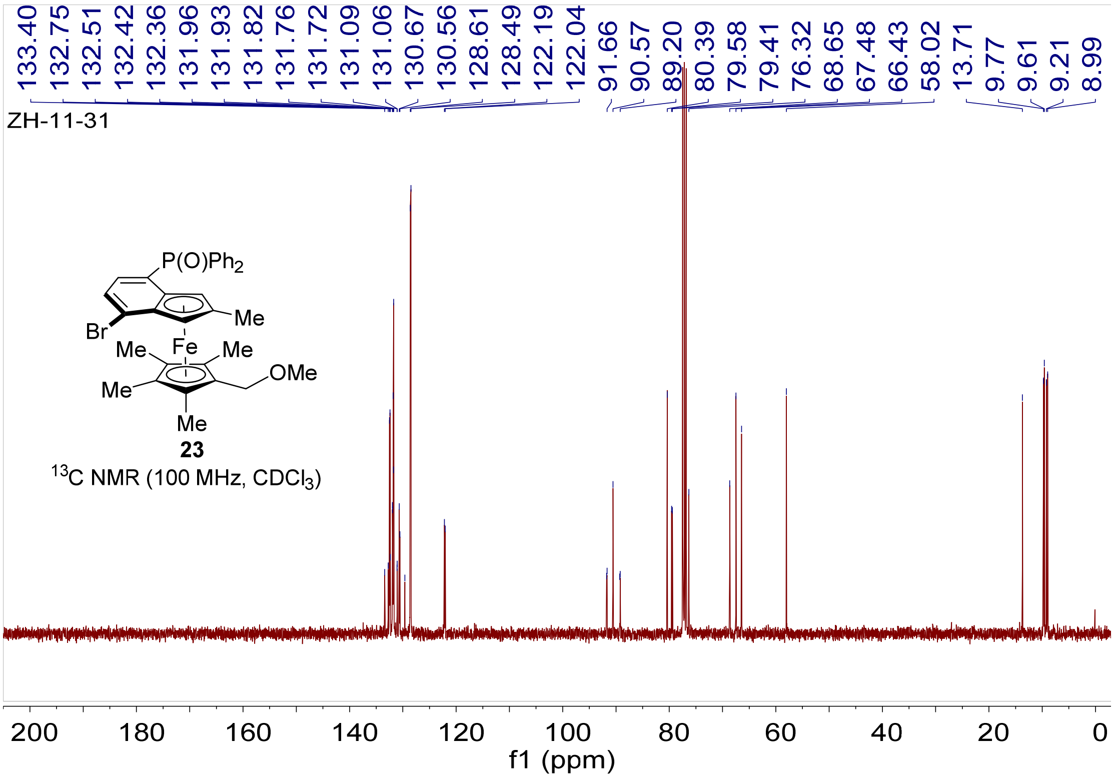

Supplementary Figure 231. ^{13}**C NMR spectra of compound 23**

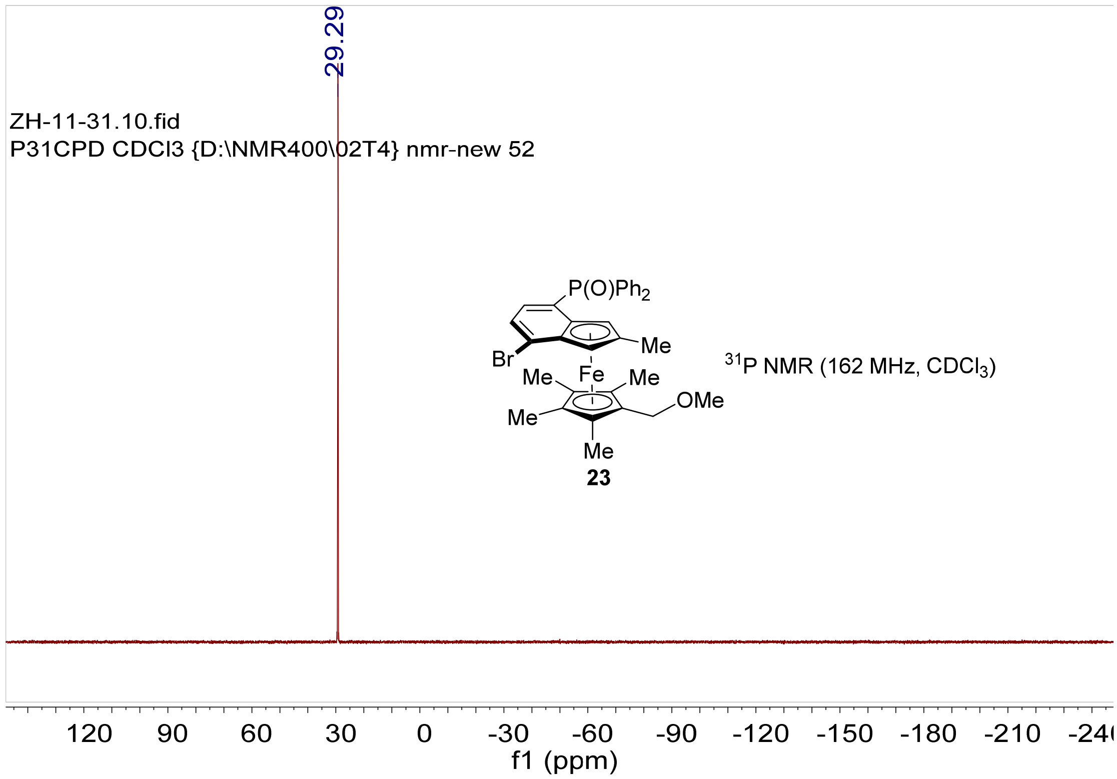

Supplementary Figure 232. ^{31}P NMR spectra of compound 23

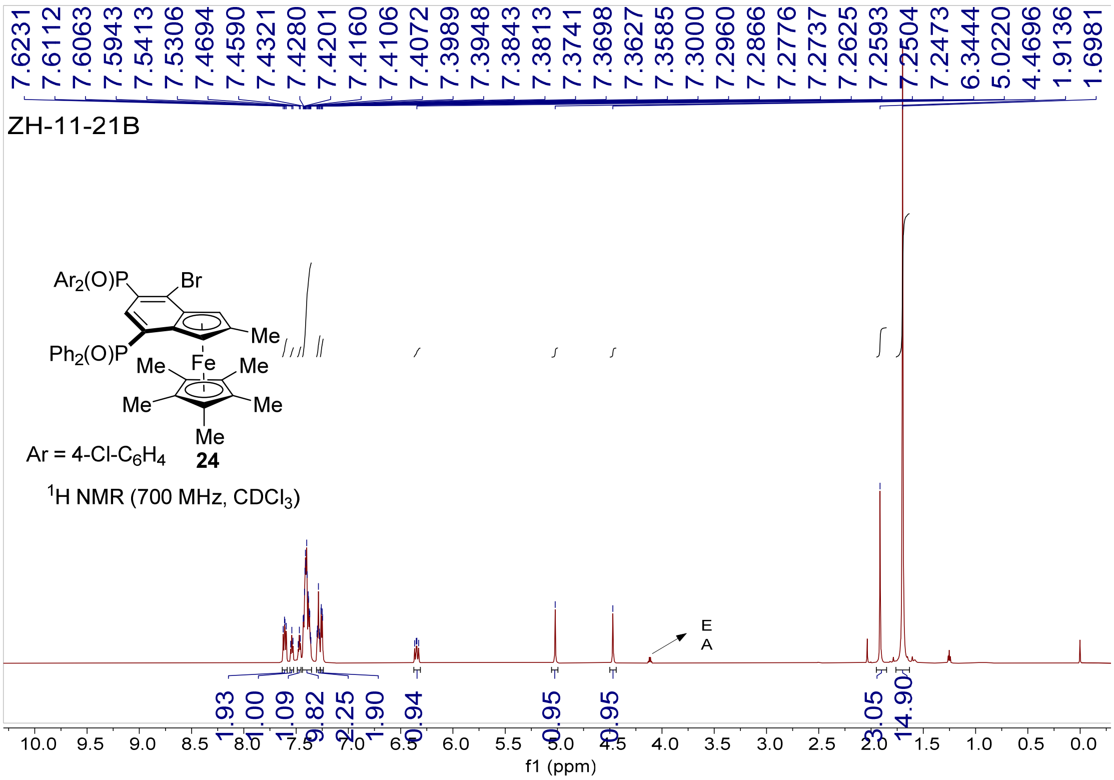

Supplementary Figure 233. ^{1}**H NMR spectra of compound 24**

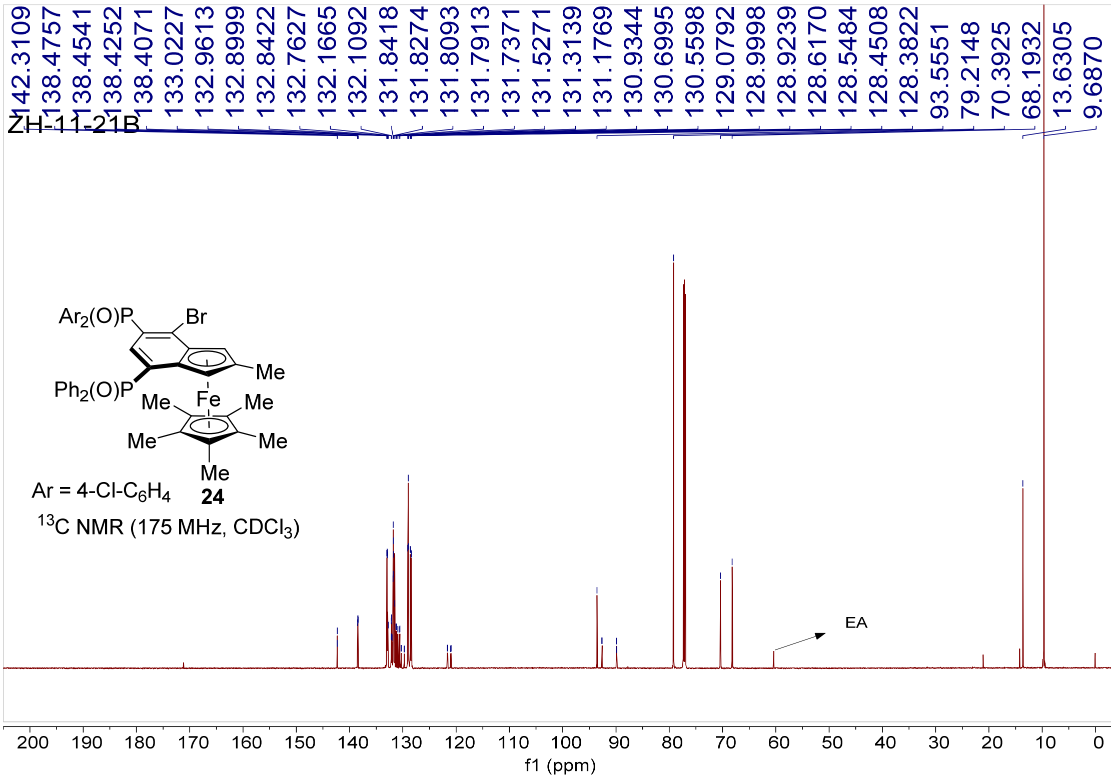

Supplementary Figure 234. ^{13}**C NMR spectra of compound 24**

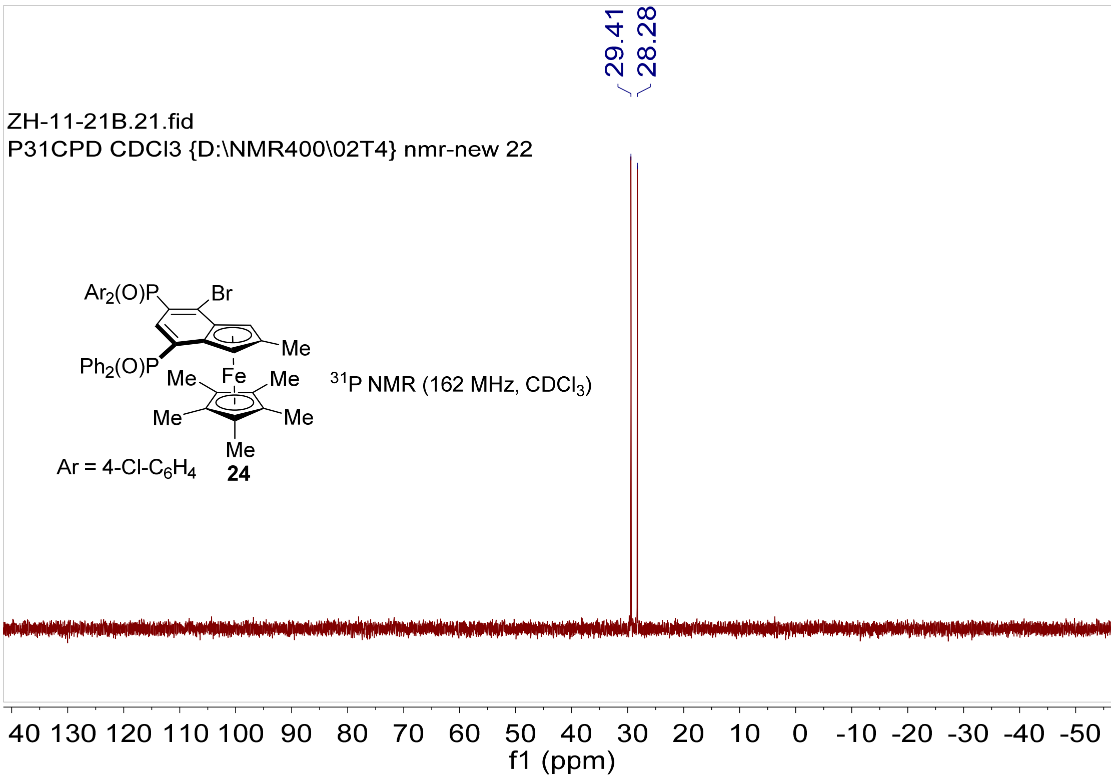

Supplementary Figure 235. ^{31}P NMR spectra of compound 24

4. Supplementary References

1 Gou, X. Y. *et al.* Visible-light-induced ligand-free $RuCl_3$ catalyzed C-H phosphorylation in water. *Chem. Commun.* **56**, 4704-4707 (2020).

2 Lei, T. *et al.* Cobaloxime catalysis for enamine phosphorylation with hydrogen evolution. *Org. Lett.* **22**, 5385-5389 (2020).

3 Busacca, C. A. *et al.* A superior method for the reduction of secondary phosphine oxides. *Org. lett.* **7**, 4277-4280 (2005).

4 Thimmaiah, M., Luck, R. L. & Fang, S. Novel benzoferrocenyl chiral ligands: Synthesis and evaluation of their suitability for asymmetric catalysis. *J. Organomet. Chem.* **692**, 1956-1962 (2007).

5 Bhattacharyya, S. Highly efficient reductive deoxygenation of acylferrocenes and alpha-ferrocenylalcohols using titanium(iv) chloride and sodium cyanoborohydride. *Synlett*, 971-972 (1995).

6 Brown, L. C., Ressegue, E. & Merola, J. S. Rapid access to derivatized, dimeric, ring-substituted dichloro(cyclopentadienyl)rhodium (III) and iridium (III) complexes. *Organometallics* **35**, 4014-4022 (2016).

7 Kang, D., Ricci, F., White, R. J. & Plaxco, K. W. Survey of redox-active moieties for application in multiplexed electrochemical biosensors. *Anal. Chem.* **88**, 10452-10458 (2016).

8 Rickmeier, J. & Ritter, T. Site-specific deoxyfluorination of small peptides with [(18) F] fluoride. *Angew. Chem. Int. Ed.* **57**, 14207-14211 (2018).

9 Khobragade, D. A. *et al.* Acceptor-substituted ferrocenium salts as strong, single-electron oxidants: synthesis, electrochemistry, theoretical investigations, and initial synthetic application. *Chem. Eur. J.* **18**, 12267-12277 (2012).
